# Transcriptome-wide identification of mRNAs and lincRNAs associated with trastuzumab-resistance in HER2-positive breast cancer

**DOI:** 10.18632/oncotarget.10637

**Published:** 2016-07-16

**Authors:** Callie R. Merry, Sarah McMahon, Megan E. Forrest, Cynthia F. Bartels, Alina Saiakhova, Courtney A. Bartel, Peter C. Scacheri, Cheryl L. Thompson, Mark W. Jackson, Lyndsay N. Harris, Ahmad M. Khalil

**Affiliations:** ^1^ Department of Genetics and Genome Sciences, Case Western Reserve University, Cleveland, OH 44106, USA; ^2^ Department of Biochemistry, Case Western Reserve University, Cleveland, OH 44106, USA; ^3^ Case Comprehensive Cancer Center, Case Western Reserve University, Cleveland, OH 44106, USA; ^4^ Department of Medicine and Case Western Reserve University, Cleveland, OH 44106, USA; ^5^ Department of Nutrition, Case Western Reserve University, Cleveland, OH 44106, USA

**Keywords:** breast cancer, HER2, drug resistance, trastuzumab-resistance, cancer therapy

## Abstract

Approximately, 25–30% of early-stage breast tumors are classified at the molecular level as HER2-positive, which is an aggressive subtype of breast cancer. Amplification of the HER2 gene in these tumors results in a substantial increase in HER2 mRNA levels, and consequently, HER2 protein levels. HER2, a transmembrane receptor tyrosine kinase (RTK), is targeted therapeutically by a monoclonal antibody, trastuzumab (Tz), which has dramatically improved the prognosis of HER2-driven breast cancers. However, ~30% of patients develop resistance to trastuzumab and recur; and nearly all patients with advanced disease develop resistance over time and succumb to the disease. Mechanisms of trastuzumab resistance (TzR) are not well understood, although some studies suggest that growth factor signaling through other receptors may be responsible. However, these studies were based on cell culture models of the disease, and thus, it is not known which pathways are driving the resistance *in vivo*. Using an integrative transcriptomic approach of RNA isolated from trastuzumab-sensitive and trastuzumab-resistant HER2+ tumors, and isogenic cell culture models, we identified a small set of mRNAs and lincRNAs that are associated with trastuzumab-resistance (TzR). Functional analysis of a top candidate gene, S100P, demonstrated that inhibition of S100P results in reversing TzR. Mechanistically, S100P activates the RAS/MEK/MAPK pathway to compensate for HER2 inhibition by trastuzumab. Finally, we demonstrated that the upregulation of S100P appears to be driven by epigenomic changes at the enhancer level. Our current findings should pave the path toward new therapies for breast cancer patients.

## INTRODUCTION

Breast cancer is a major health problem affecting millions of patients worldwide, and results in over 500,000 deaths annually. Previous studies have led to the classification of breast tumors into several molecular subtypes, with HER2-positive (HER2+) tumors representing ~25–30% of early-stage breast cancer patients' diagnoses [1–3]. HER2+ tumors are characterized at the molecular level by an amplification of a genomic region encompassing the HER2 gene (also known as HER2/neu and ERBB2), which is a member of the ERBB family of transmembrane receptor tyrosine kinases (RTKs) [1]. Although no known ligands bind to the HER2 receptor itself, a number of ligands bind to other ERBB family members (e.g., HER3), and lead to the heterodimerization of these members with HER2 [4]. Heterodimerization results in autophosphorylation by the intracellular tyrosine kinase domain of HER2, and the initiation of a signaling cascade that results in the activation and repression of specific mRNAs and long intergenic non-coding RNAs (lincRNAs) [5].

Trastuzumab (Herceptin^®^) is a monoclonal antibody that binds to the extracellular domain of the HER2 receptor, and is thought to inhibit its heterodimerization, and consequently, its signaling cascade [6]. Currently, trastuzumab is the standard of care for HER2+ breast cancer patients as 70% of early-stage patients appear to be cured by trastuzumab and chemotherapy. However, a significant percentage of early-stage patients (~30%) relapse after this combination by unclear mechanisms [7]. Furthermore, most patients who relapse will acquire resistance to trastuzumab during therapy and succumb to the disease [6, 8, 9]. Although several mechanisms of TzR have been proposed, it has been difficult to show convincingly that these occur in the clinical setting [6, 9]. For example, it was hypothesized that resistance occurs due to shedding of the extracellular domain of HER2, and thus, trastuzumab is no longer able to bind to HER2 [10], however, no experimental evidence has been presented to support this. Other models proposed that HER3 signaling could compensate for HER2 inhibition by trastuzumab. However, patients who received a combination of trastuzumab and pertuzumab, which blocks HER2-HER3 interaction, have a median progression-free survival of 5.5 months [11]. Thus, it is clear that other mechanisms of resistance are at play in tumors *in vivo*.

**Figure 1:**
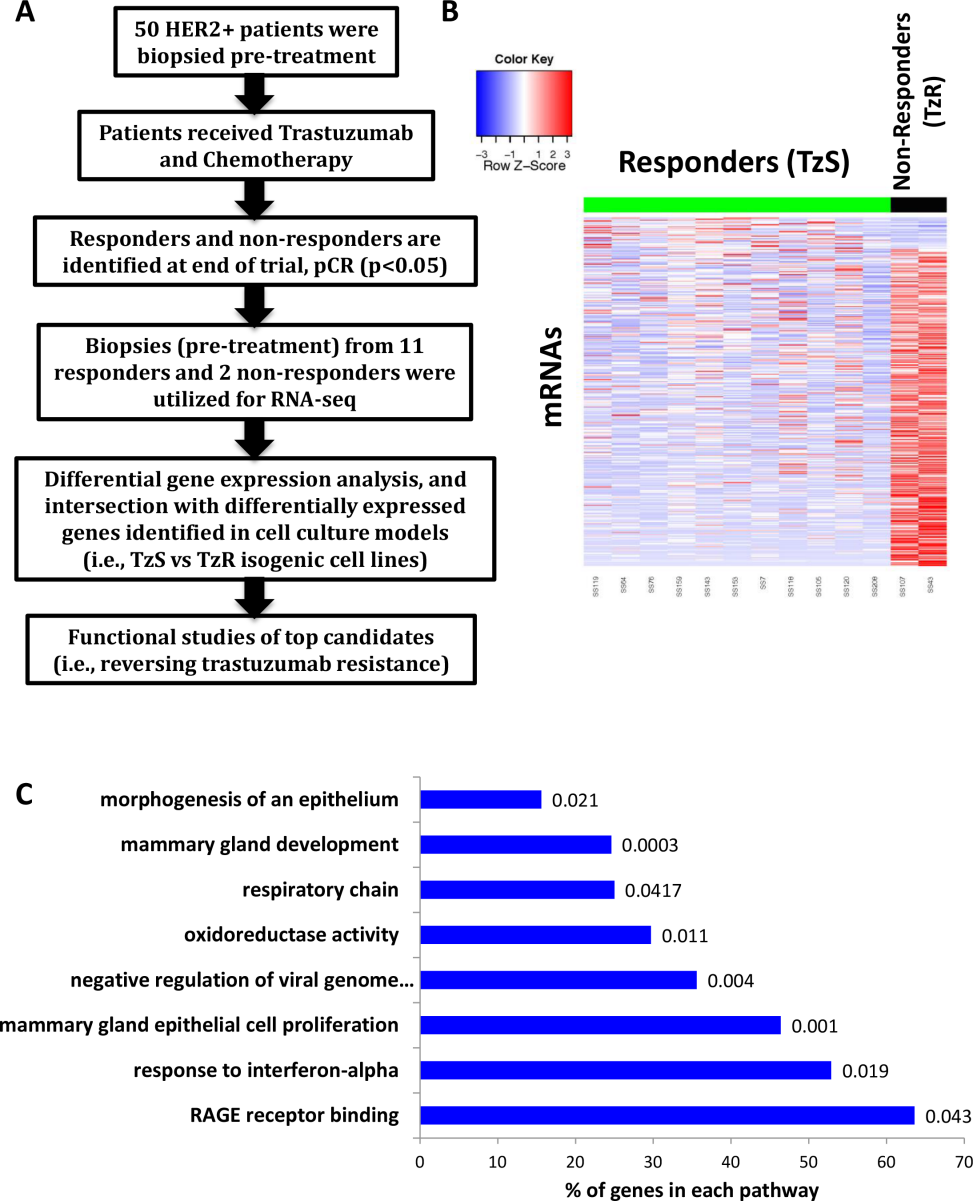
Identification of differentially expressed genes in trastuzumab-resistant (TzR) vs trastuzumab-sensitive (TzS) tumors by RNA-seq (**A**) A schematic outlining overall study design. Differentially expressed genes identified in TzS vs TzR human HER2+ tumors *in vivo* were intersected with differentially expressed genes identified in TzS vs TzR BT474 cell lines, which led to a small list of mRNAs and lincRNAs that are associated with TzR. (**B**) Heatmap representation of the differentially expressed mRNAs in TzS patients (responders) vs TzR patients (non-responders). (**C**) Pathway analysis of differentially expressed mRNAs in tumors *in vivo*. Pathway names are listed on the Y-axis, the percentage of genes affected in each pathway is indicated on the X-axis, and a *p*-value is given at the end of each bar.

Given the immense potential clinical benefits in identifying mechanisms of TzR, we decided to take a comprehensive and unbiased approach to identify the key cellular factors that drive TzR in humans *in vivo*. By applying next generation RNA-sequencing to RNA isolated from human HER2+ tumors and cell culture models of the disease that are either trastuzumab-sensitive (TzS) or trastuzumab-resistant (TzR), we have identified a small set of mRNAs and lincRNAs that are strongly associated with TzR (Figure 1A). Functional studies of a top candidate gene, S100P, demonstrated that inhibition of S100P results in reversal of trastuzumab-resistance (TzR). Our study provides for the first time a clinically relevant mechanism of TzR, and opens the door for exploring novel therapeutic strategies.

## RESULTS

### Identification of differentially expressed mRNAs and lincRNAs in trastuzumab-sensitive (TzS) vs trastuzumab-resistant (TzR) human tumors *in vivo*

We hypothesized that TzR tumors have a distinct gene expression profile compared to TzS tumors, and a subset of these differentially expressed coding and non-coding genes mediate the observed resistance. To that end, we applied RNA-seq to RNA isolated from HER2+ tumor biopsies that were collected during a clinical trial (see methods). In this clinical trial, 50 patient tumors were biopsied at the beginning of the trial prior to receiving trastuzumab (pre-trastuzumab). After receiving a combination of chemotherapy and trastuzumab for 4 months, a subset of thirteen tumors, representing the extremes of response to treatment, were selected for gene expression analysis by RNA-seq. Eleven of those thirteen patients were identified as responders (i.e., trastuzumab-sensitive or TzS) and two patients were identified as non-responders (i.e., trastuzumab-resistant or TzR) as measured by pathological complete response (pCR) or clinical non-response (SD+PD). We compared mRNA gene expression between the responders (TzS) and non-responders (TzR), and identified differentially expressed genes between the two groups prior to receiving treatment (pre-trastuzumab) (Figure 1A). Specifically, we identified ~1,542 mRNAs and 371 lincRNAs that are differentially expressed (Figure 1B, and Supplementary Files S1 and S2). We performed pathway analysis on differentially expressed mRNAs, and found several pathways to be affected including those related to mammary gland cell proliferation and development (Figure 1C). Also, RAGE receptor binding was statistically significant and emerged as a key player (see below). Currently, it is not possible to perform pathway analysis on lincRNAs as only a small percentage of all annotated lincRNAs (~10,000 in the human genome) have been functionally characterized (~200 lincRNAs) [12].

### Characterization of TzS and TzR HER2+ cell culture model

**Figure 2:**
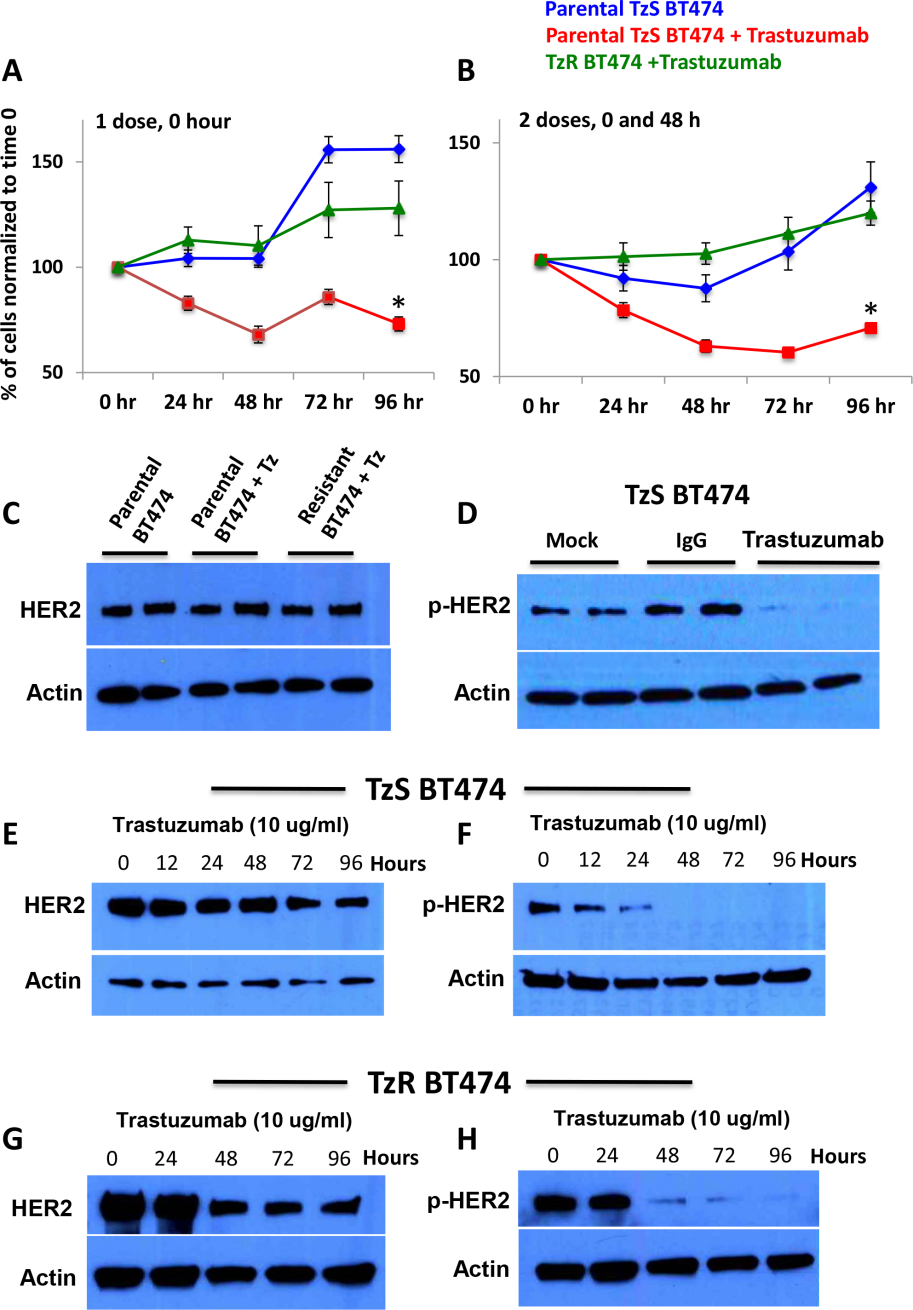
Characterization of HER2-positive trastuzumab-sensitive (TzS) and trastuzumab-resistant (TzR) BT474 breast cancer cell lines (**A**–**B**) Cell proliferation analysis of TzS and TzR BT474 cell lines with either (A) one dose of 10 μg/ml trastuzumab at 0 hr or (B) 10 μg/ml trastuzumab at 0 and 48 hours demonstrated that while TzS BT474 cells is significantly decreased by trastuzumab, TzR cells proliferation is not affected. (**C**) Western blot analysis demonstrates that both TzS and TzR BT474 cells have comparable levels of HER2 protein. (**D**) Trastuzumab (10 μg/ml) treatment significantly affects p-HER2 levels in TzS BT474 cells within 24 hours post treatment. (**E**) HER2 protein levels stay relatively constant in TzS BT474 cells with trastuzumab treatment over 96 hours. (**F**) p-HER2 levels decrease significantly at 24 hours post trastuzumab treatment, and remain undetectable at a 96 h timepoint. (**G**–**H**) Decreased levels of HER2 and p-HER2 levels are observed in TzR cells at 48 h post trastuzumab treatment.

To further pinpoint key mRNAs and lincRNAs that contribute to TzR, we generated TzR HER2+ BT474 cells by chronically exposing the cell line to trastuzumab over six weeks. To confirm that we have generated BT474 cells that are resistant to trastuzumab (TzR), we measured cell proliferation of the parental TzS and isogenic TzR cells under exposure to trastuzumab over a 96 hour time period. We found that the proliferation of parental TzS BT474 cells is significantly affected when exposed to trastuzumab within 48 hours. By contrast, the proliferation of TzR BT474 cells was not affected (Figure 2A–2B).

Next, we examined the effect of trastuzumab on HER2 and phospho-HER2 (p-HER2) protein levels in TzS and TzR cells. We found TzS cells to express comparable levels of HER2 protein to TzR cells (Figure 2C). However, trastuzumab strongly inhibits the phosphorylation of HER2 (p-HER2) in TzS cells within 24 hours of exposure (Figure 2D). Next, we examined the expression of HER2 and p-HER2 levels in both TzS and TzR cells over a 96-hour time course, and found: i. HER2 protein levels are not significantly affected in the parental TzS line by trastuzumab until the 96-hour time point (Figure 2E), ii. p-HER2 levels significantly decrease in the parental TzS line within 12 hours post-exposure to trastuzumab (Figure 2F), iii. Surprisingly, both HER2 and p-HER2 levels are significantly affected in the TzR BT474 cells at 48 hours post exposure to trastuzumab (Figure 2G–2H). The decrease in HER2 and p-HER2 levels in the TzR line demonstrates that trastuzumab can still bind to and inhibit HER2 phosphorylation in TzR cells. These observations demonstrate that the mechanism of TzR, at least in a subset of tumors, is not due to truncation or mutations of HER2 that precludes trastuzumab binding.

### Transcriptome-wide changes in gene expression in a cell culture model of TzR

**Figure 3:**
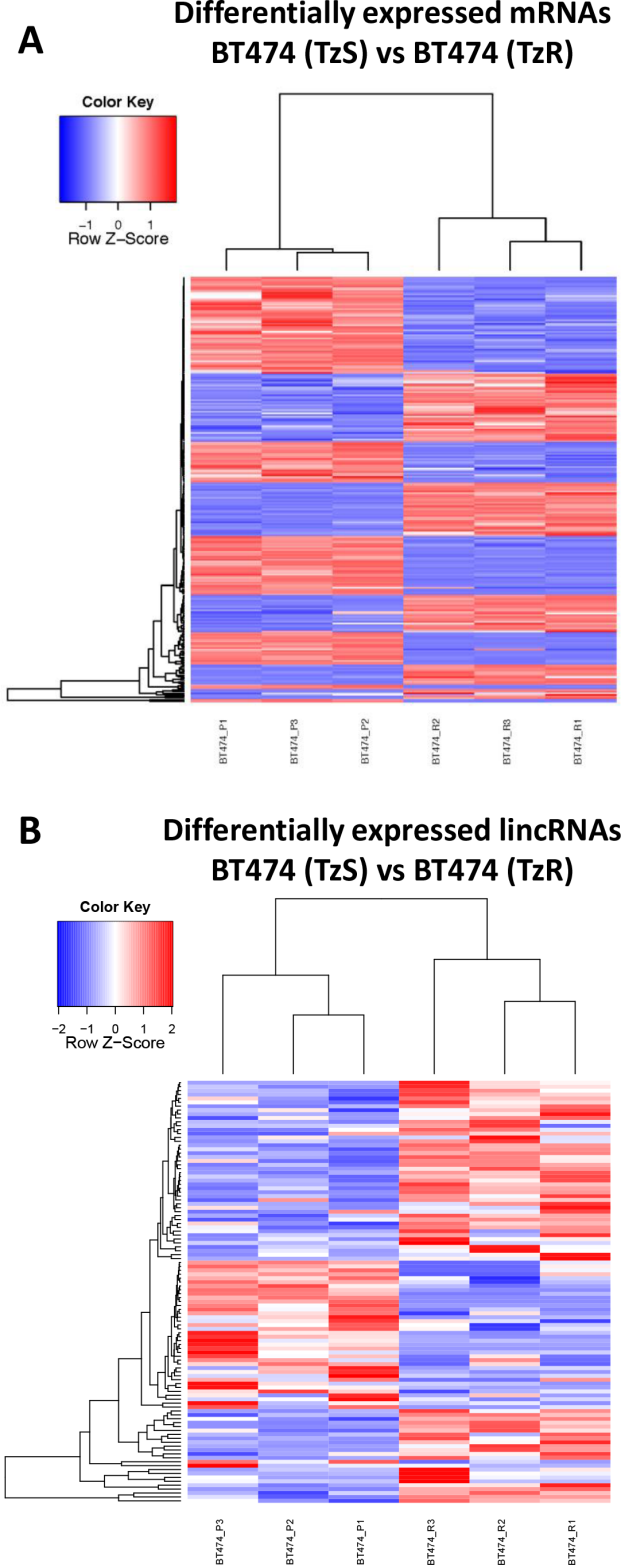
Numerous mRNAs and lincRNAs are differentially expressed in TzS vs TzR BT474 cells Heatmap representation of differentially expressed (**A**) mRNAs and (**B**) lincRNAs in TzS BT474 vs TzR BT474 isogenic cell lines.

To identify genes associated with TzR in our cell culture model described above, we quantified changes in gene expression between parental TzS BT474 and TzR BT474 cells by RNA-seq. We identified 233 mRNAs and 34 lincRNAs as differentially expressed (fold change ≥ 2, *p* ≤ 0.05) (Figure 3A–3B, Supplementary Files S3 and S4). To further refine our list of genes that are specifically associated with TzR, and not due to short-term exposure to trastuzumab, we treated the parental TzS BT474 cells with trastuzumab, and collected RNA at 48 hours post-exposure. We measured changes in gene expression by RNA-seq and identified 242 mRNAs and 27 lincRNAs that are differentially expressed as compared to mock treated cells (≥ 2 fold change and *p* ≤ 0.05) (Supplementary Files S5 and S6). Subsequently, mRNAs and lincRNAs that are affected by short-term exposure to trastuzumab were subtracted from mRNAs and lincRNAs that are differentially expressed in TzR vs TzS cells. This enabled us to further pinpoint mRNAs and lincRNAs that are specifically associated with long-term resistance to trastuzumab in culture. This analysis narrowed down the gene list to 201 mRNAs and 25 lincRNAs.

**Figure 4:**
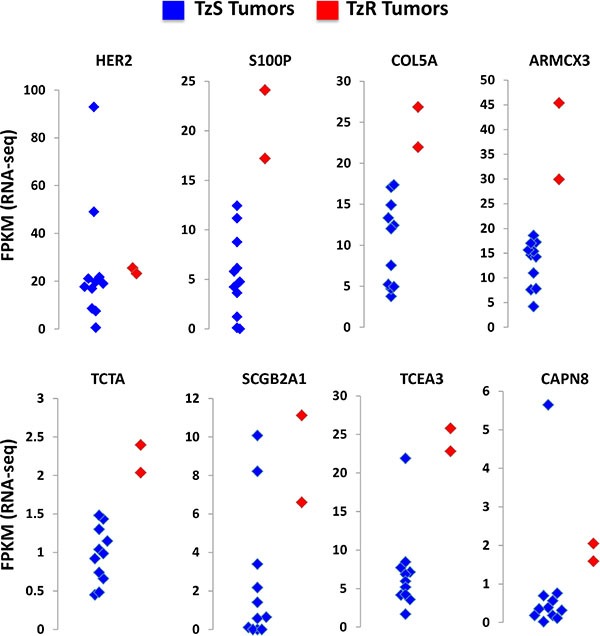
Top candidate mRNAs associated with trastuzumab-resistant (TzR) The expression of each mRNA in each tumor sample (blue dots: TzS, red dots: TzR) is shown as FPKM value based on RNA-seq analysis. HER2 mRNA levels are shown as a control and do not significantly change between TzS vs TzR tumors. By contrast, mRNAs identified in our analysis show striking upregulation in TzR tumors (red) vs the majority of TzS tumors (blue).

**Figure 5:**
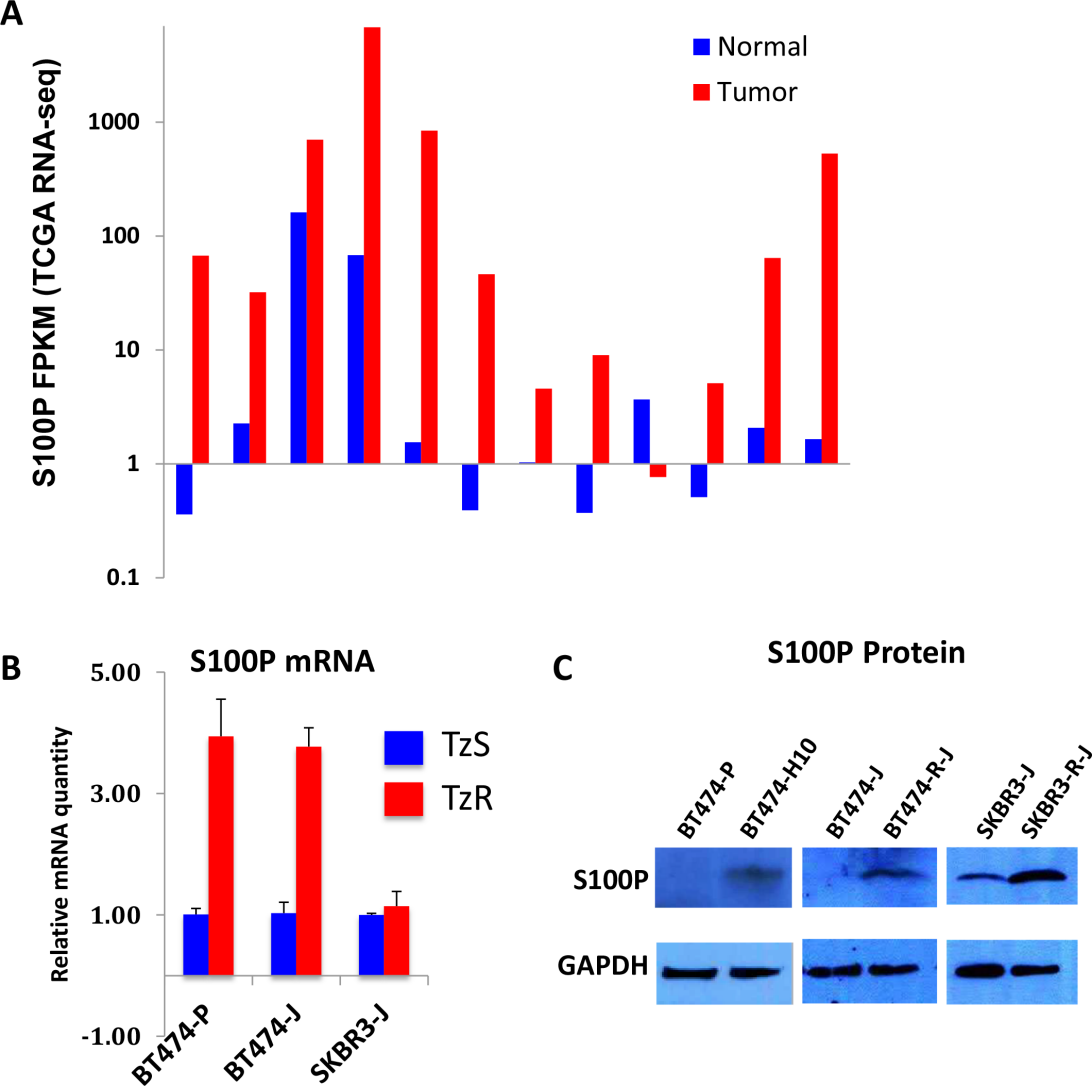
S100P is upregulated in trastuzumab-resistant cells (**A**) S100P is highly upregualted in HER2+ tumors as compared to matched normal breast tissues (TCGA RNA-seq data). (**B**) S100P mRNA is elevated in two independently generated TzR vs TzS BT474 cells. (**C**) S100P protein is elevated in TzR vs TzS BT474 and SKBR3 cells as measured by western blot analysis.

We intersected mRNAs and lincRNAs identified in our cell culture model with differentially expressed mRNAs and lincRNAs identified in our *in vivo* data set (clinical trial). This analysis led to the identification of 18 mRNAs and 7 lincRNAs that are associated with TzR *in vivo* and in cell culture, with concordant expression patterns (*p* < 0.0001, two-tailed Fischer's exact test) (Supplementary File S7). Expression of top mRNA genes identified in our analysis in TzS vs TzR tumors *in vivo* is shown using cluster graphs, HER2 expression is also shown to demonstrate that HER2 levels do not change dramatically between TzS and TzR tumors (Figure 4). Careful examination of these genes, their known roles in breast cancer, and our bioinformatics analysis of key pathways associated with TzR *in vivo* (see Figure 1C), suggested a potential key role of S100P. S100P is part of the S100 protein family, has documented roles in tumorigenesis [13], and is known to bind to the receptor RAGE (see Figure 1C), and activates similar signaling pathways to those activated by HER2 signaling. Furthermore, analysis of S100P in Oncomine demonstrated that S100P is strongly associated with breast cancer (*p* = 1.8E-94, average fold change = 10.9) (Supplementary Figure S1). Lastly, examination of RNA-seq of The Cancer Genome Atlas (TCGA) also demonstrated that S100P is highly upregulated in HER2+ breast cancer (Figure 5A).

To further confirm the upregulation of S100P in TzR cells, we examined S100P mRNA and protein levels in the TzS vs TzR BT474 cell line utilized for our RNA-seq studies, and two independently generated TzS vs TzR BT474 and SKBR3 cell lines (three isogenic TzS vs TzR cell lines total). We found S100P to be upregulated at both the mRNA (Figure 5B) and protein levels (Figure 5C), with potentially increased stability of the S100P mRNA in SKBR3 cells. Importantly, we found S100P to be upregulated in a cohort of breast cancer cell lines as compared to normal human mammary epithelial cells (Supplementary Figure S2), demonstrating that S100P is upregulated from normal tissues to breast tumors, and further upregulated or stabilized in TzR tumors.

### Inhibition of S100P reverses TzR in a cell culture model

**Figure 6:**
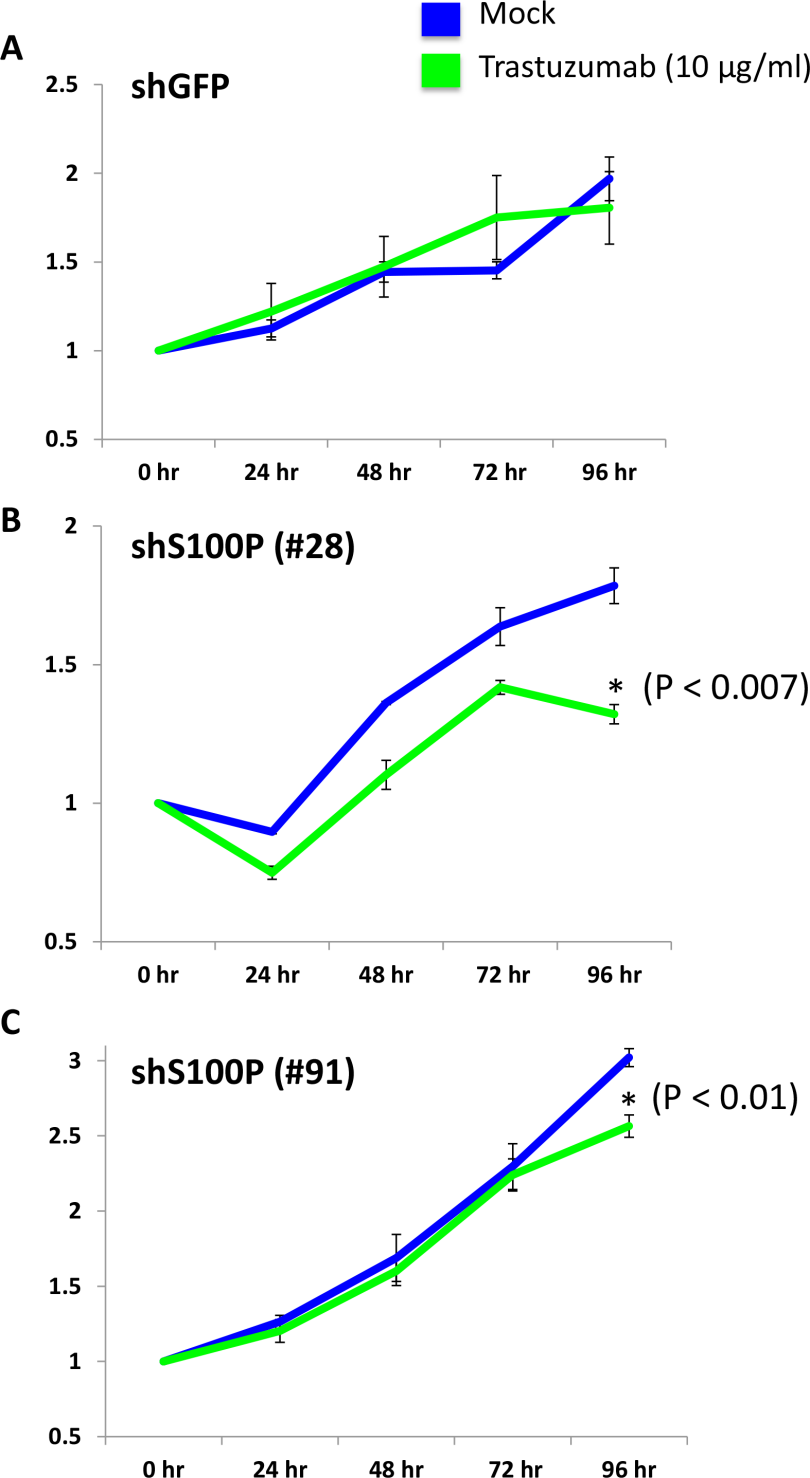
Knock down of S100P partially reverses TzR in cell culture Cell proliferation analysis using MTS colorimetric assay demonstrated that TzR BT474 cells treated with either mock vs trastuzumab (10 μg/ml) for 96 hours show decreased proliferation rate when S100P levels are depleted with two independent shRNAs targeting S100P vs a negative control shRNA targeting GFP.

**Figure 7:**
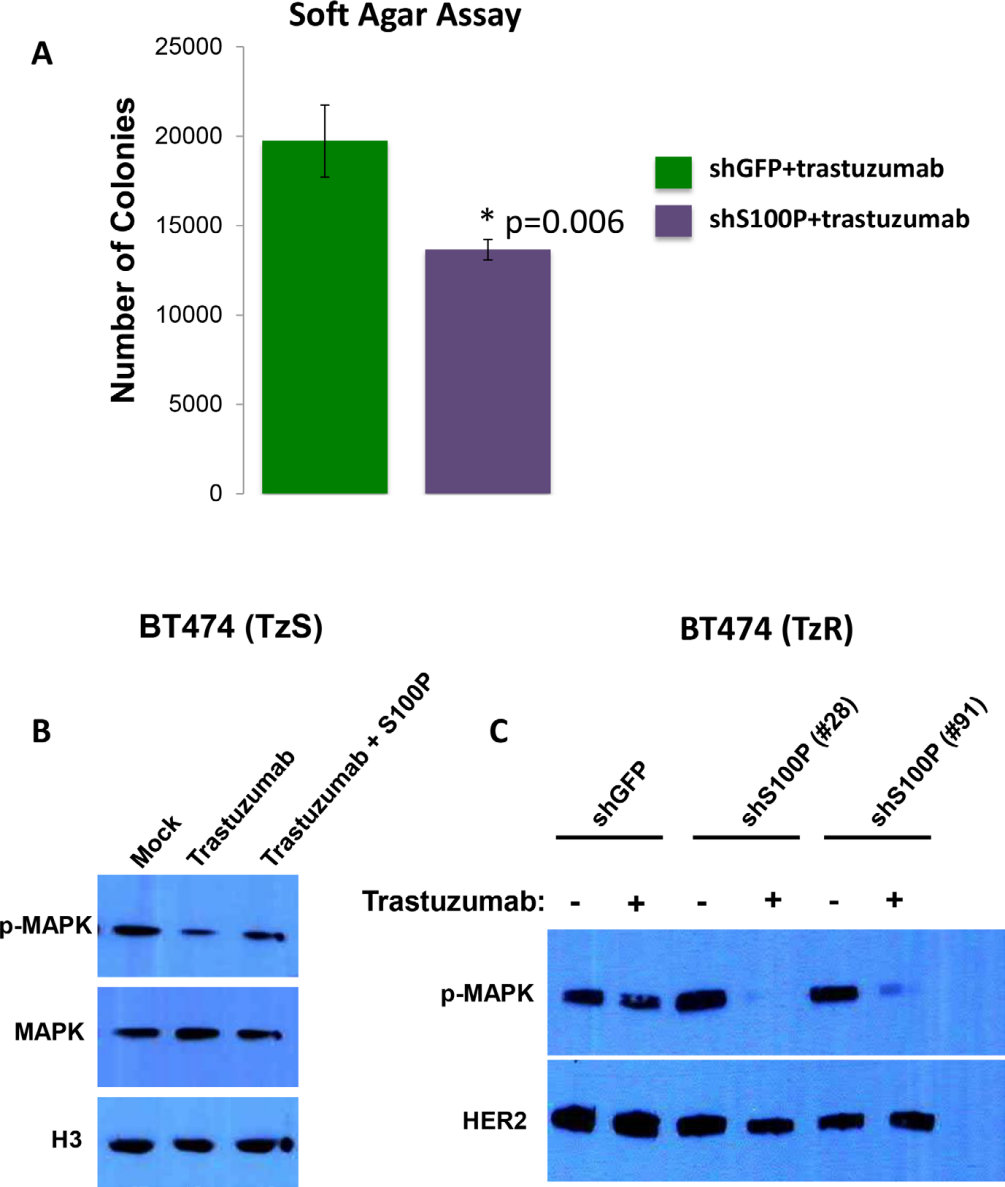
S100P activates Ras/MEK/MAPK pathway (**A**) BT474 cells infected with shS100P lentivirus or control shGFP lentivirus were plated into soft agar to assess anchorage independent growth (AIG). Cells were grown in the presence of 10 μg/mL Trastuzumab for 3 weeks. (*P < 0.01, Student's *t* test). (**B**) Trastuzumab treatment inhibits p-MAPK levels in TzS BT474 cells, but the addition of recombinant S100P protein is sufficient to restore p-MAPK levels. (**C**) Knock down of S100P in TzR cells results in significant decrease in p-MAPK levels when cells are treated with trastuzumab, further supporting a role of S100P in trastuzumab-resistance.

To test the potential role of S100P in TzR, we designed and tested five distinct shRNAs against S100P, and identified two shRNAs that are effective at knocking down S100P (Supplementary Figure S3A). To determine if the depletion of S100P would re-sensitize TzR cells to trastuzumab, we tested the proliferation of TzR BT474 cells that were infected with either shGFP or shS100P in mock vs trastuzumab treatments. BT474 cells infected with shGFP showed no response to trastuzumab (Figure 6A), by contrast, the proliferation of TzR BT474 cells infected with shS100P was significantly impacted when exposed to trastuzumab (Figure 6B–6C). We also examined the rate of apoptosis of TzR cells in response to shRNA-mediated depletion of S100P, and found a significant increase in apoptosis in TzR cells treated with shS100P vs shGFP (Supplementary Figure S4). Lastly, we performed soft agar colony formation assays under trastuzumab treatment, and found that the knock down of S100P results in a significant decrease in number of colonies, as compared to shGFP (Figure 7A). Collectively, these data demonstrate that depletion of S100P partially re-sensitizes TzR cells to trastuzumab.

### S100P activates Ras/MEK/MAPK pathway

S100P is known to bind to the receptor RAGE, leading to activation of the Ras/MEK/MAPK and other signaling pathways [14]. The Ras/MEK/MAPK is a key pathway that is known to be activated by HER2 signaling [1]. To test the hypothesis that S100P leads to activation of Ras/MEK/MAPK, and thus compensates for HER2 inhibition by trastuzumab, we performed the following experiments. First, we examined the effect of recombinant S100P on p-MAPK in TzS cells as follows: BT474 cells (TzS) were treated with trastuzumab alone or with trastuzumab and recombinant S100P protein, and the levels of pMAPK, MAPK and histone H3 (control) were measured by WB. We found that trastuzumab treatment results in reducing p-MAPK levels, but the addition of S100P recombinant protein is sufficient to restore p-MAPK protein levels (Figure 7B). Next, we examined the effect of knocking down S100P on p-MAPK in TzR cells. We found that TzR cells harboring shGFP had minor reduction of p-MAPK when treated with trastuzumab, however, TzR cells harboring shS100P showed significant reduction of p-MAPK when treated with trastuzumab (Figure 7C). These findings demonstrate that the upregulation of S100P provides a mechanism for activating Ras/MEK/MAPK independent of HER2 signaling in TzR cells.

### Upregulation of S100P is driven by epigenetic changes at enhancers

**Figure 8:**
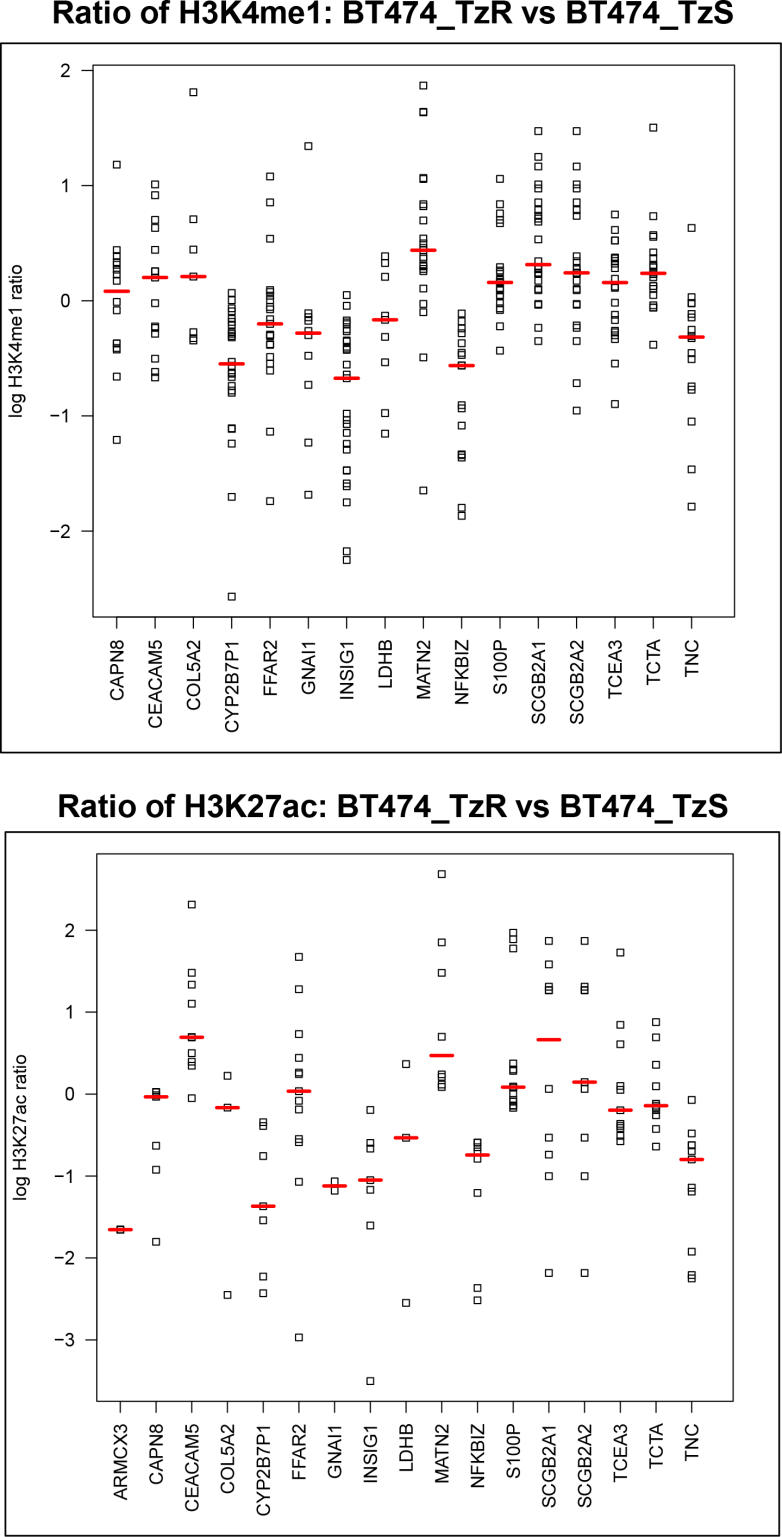
S100P upregulation in TzR cells is wired at the epigenetic level All high confidence H3K4me1 and H3K27ac peaks (MACS, *p*-value < 1E-9) within 150KB of the TSS for top genes associated with trastuzumab-resistance were retrieved and plotted as log2 ratio of the ChIP signal in TzR BT474 vs TzS BT474 cell line. Red lines demarcate the median signal fold change for each gene. (top panel) Each data point corresponds to relative levels of H3K4me1 ChIP-seq signals (trastuzumab-resistant/ trastuzumab-sensitive) located within 150-kb of each of the 16 dysregulated genes. (bottom panel) Same as top, but for H3K27ac.

To identify the potential mechanism(s) that drive increased S100P expression in TzR tumors and cell lines, we examined global changes in the epigenome at enhancer regions. Specifically, we examined changes in histone H3 lysine 4 monomethylation (H3K4me1) and H3 lysine 27 acetylation (H3K27ac), two key histone modifications associated with active enhancers [15]. First, we identified genome-wide changes in H3K4me1 and H3K27ac between TzS and TzR cells (Supplementary Figure S5, Supplementary Files S8 and S9). Next, we specifically examined enhancers surrounding top candidate genes identified in our RNA-seq analysis, including S100P (Figure 8). Both H3K4me1 and H3K27ac profiles indicate that the upregulation of S100P in TzR cells is driven by epigenetic changes at several enhancers regulating S100P (Figure 8, and Supplementary Figure S6). These findings indicate that trastuzumab-resistance is wired at the epigenetic level, at least for a subset of genes identified in our study.

## DISCUSSION

Trastuzumab was originally developed and utilized as an adjuvant therapy for HER2-positive metastatic breast cancer, and currently as an adjuvant therapy in early-stage HER2-positive patients [16–19]. The clinical utilization of trastuzumab has revolutionized the treatment of HER2-positive breast cancer patients, however, a significant percentage of patients (~30%) do not respond to trastuzumab or acquire resistance to it, by largely unknown mechanisms [9, 10]. Because of the immense therapeutic benefits of trastuzumab to HER2-positive breast cancer patients, it is critical to identify the mechanisms of TzR. Although a number of models of TzR have been proposed, none has been proven to be present in clinical specimens from patients treated with Trastuzmab. In this manuscript, we have taken an unbiased genome-wide approach to identify genes and pathways that may drive and/or contribute to TzR. By utilizing next generation RNA-seq (RNA-seq) of RNA isolated from tumors and cell lines that are either TzS or TzR, we identified key genes and pathways that potentially contribute to TzR. Importantly, the utilization of tumor biopsies from patients that are either sensitive or resistant to trastuzumab enabled us to obtain clinically relevant data. However, it is important to point out that the tissue culture models used in our studies do not represent all HER2+ tumors, and thus, we may have missed other key genes and pathways that could also contribute to trastuzumab-resistance.

Many of the mRNAs that we identified as associated with TzR have been previously shown to be dysregulated in various types of cancer. MATN2 and SCGB2A2 are upregulated in ovarian cancer [20, 21]; S100P is upregulated in prostate cancer, invasive ductal carcinomas, and pancreatic cancer [22]; LDHB is a biomarker for triple negative breast cancer [23]; and TNC is associated with Tamoxifen resistance in breast cancer [24]. Although the exact roles of these genes in TzR are yet to be elucidated, previous studies of these genes in cancer should provide some preliminary insights. For example, the role of S100P in the activation of RAGE-mediated Ras/MEK/MAPK signaling provides clues into its role in TzR [14, 25].

S100P emerged as a top candidate for functional studies as it showed strong association with TzR both *in vivo* and *in vitro* studies, it is highly upregulated in many cancer types, and it belongs to the S100 family of proteins with well-documented roles in tumorigenesis [13]. S100 family members, including S100P, function as homodimers with a Ca^2+^-binding EF-hand motif [26]. Calcium binding to S100P promotes a conformational change exposing hydrophobic residues, which enables the interaction with protein binding partners [27]. S100P acts as both an intracellular and extracellular signaling molecule, and has been observed in the nucleus, cytoplasm, and extracellular matrix [28]. Previous studies of S100P in breast cancer have demonstrated that the expression of S100P correlates with breast cancer progression and decreased patient survival [29–32]. Also, S100P roles in tumorigenesis have been well-documented in pancreatic cancer. These studies have demonstrated that S100P exerts its oncogenic activities via interaction and activation of the receptor RAGE [14, 25]. The extracellular ligand-binding domain of RAGE is known to bind to a number of ligands, including S100P, to initiate downstream signaling pathways that promote cell proliferation, survival, and motility [33, 34]. Blocking S100P interaction with RAGE is sufficient to significantly impact the growth of tumors [25, 33]. These findings suggest that S100P-RAGE interaction could be therapeutically blocked to reverse TzR, at least in a subset of HER2+ patients.

To gain insights into the potential mechanisms driving changes in gene expression of S100P and other key genes identified in our study in TzS vs TzR cells, we examined chromatin changes at enhancers. Enhancers play major roles in regulating gene expression in virtually all cell types, and are known to become altered in cancer [15]. Two key chromatin marks, H3K4me1 and H3K27ac, identify active enhancers across the genome, and examining these two marks in TzS vs TzR cells demonstrated changes in enhancer activity at many loci. Enhancers regulating S100P showed higher levels of both H3K4me1 and H3K27ac in TzR cells as compared to TzS cells. These observations suggest that TzR cells acquire epigenetic changes that impact gene expression, and consequently, growth advantages.

Our study also led to the identification of long intergenic non-coding RNAs (lincRNAs) that are dysregulated in TzR tumors and cell lines. We and others previously demonstrated critical roles of lincRNAs in regulating gene expression at the transcriptional and post-transcriptional level in mammalian and other systems [35–44]. Currently, the role of these lincRNAs in TzR is yet to be elucidated, but they could potentially offer novel mechanistic insights and therapeutic targets in cancer biology. A major mechanism utilized by lincRNAs to regulate gene expression is via the recruitment of chromatin-modifying complexes and other proteins to specific gene loci [38, 42, 43, 45–50]. Thus, such lincRNAs, when they become dysregulated in cancer, could have profound effect on the epigenome [42]. By targeting such lincRNAs, we could potentially modulate the epigenetic landscape of cancer cells to a normal state, at least partially [51]. A key advantage of targeting lincRNAs is their tissue-specific expression, potentially reducing negative side effects. In future studies, we will assess the roles of these lincRNAs in TzR, and their potential utility as therapeutic targets.

In summary, we have utilized both clinical HER2+ tumor samples and cell culture models of trastuzumab-resistance to identify key mRNAs and lincRNAs associated with resistance to trastuzumab in tumors *in vivo*. Our findings provide a small list of potential therapeutic targets that can be experimentally tested to develop novel therapies for this aggressive form of breast cancer.

## MATERIALS AND METHODS

### Clinical trial information

Brown University Oncology Group (Brown University, Yale University, Cedar-Sinai Center), PI: William Sikov MD; Correlative Science PI: Lyndsay Harris MD; BrUOG Study ID: BR-211B; Clinical Trials.gov ID NCT00617942.

### RNA isolation and next generation RNA sequencing (RNA-seq) of clinical trial tumor samples

Patient biopsies cores were flash frozen for processing. RNA was isolated from samples using AllPrep (Qiagen) and amplified using Ovation RNA-seq System (NuGen). Library preparation of samples was completed using TruSeq v3 (Illumina) and sequenced on the Illumina HiSeq 2500 platform. Cufflinks V2.0.2 was used to annotate the aligned reads to human mRNA and lincRNA. Expression values were calculated as FPKM (fragment per kilobase of exon per million of mapped fragments). For our analysis, transcript expression was defined as if the FPKM value across either all non-responders (TzR) or all responders (TzS) samples were ≥ 1.0 for mRNAs and ≥ 0.25 for lincRNAs. The mean expression was calculated for TzR and TzS and the fold change was calculated as TzR/TzS to identify differentially expressed transcripts. Transcripts were defined as differentially expressed if the fold change was ≥ 2.0 or ≤ 0.5. Heatmaps were generated in R using heatmap.2 with Z scores scaled by row using standard Z score calculation of log fold change.

### Cell lines

TzS cells were treated with 10 μg/ml trastuzumab for at least six weeks to acquire TzR. Human breast cancer cell lines used in this study were grown in Hybri-Care Medium (ATCC_46-X™) supplemented with 10% fetal bovine serum (Bioexpress) and 100 units/ml penicillin and 100 μg/ml streptomycin (Life Technologies) at 37°C with 5% CO_2._ TzR cell lines are maintained in 10 μg/ml of trastuzumab.

### Cell proliferation assay

Cell proliferation assays were performed using CellTiter 96^®^ AQ_ueous_ One Solution Cell Proliferation Assay (Promega) as indicated by manufacturer's instructions and read as absorbance at 490 nm.

### Western blot analyses

Protein lysates were prepared with Laemmli Sample Buffer (BioRad) and separated on a 4–20% gradient SDS-PAGE Mini-Protean TGX^TM^ Gels (BioRad). Gels were transferred to nitrocellulose membrane (Thermo Scientific) and probed with primary antibodies overnight at the following dilutions: anti-actin (Ambion, AM4302, 3.1 mg/ml) 1:1000, anti-HER2/Erb2 (Cell Signaling, 2242S) 1:1000, anti-HER2-phospho (Fisher, MS-1072-P0, 200 μg/ml) 1:500, anti-p44/42 MAPK (Erk1/2) (3A7) (Cell Signaling, 9107S) 1:1000, anti-phospho-p44/42 MAPK (Erk1/2) (Thr202/Tyr204) (Cell Signaling, 4370P) 1:500, and anti-H3 (Cell Signaling, 9715S) 1:2000. Secondary antibodies anti-mouse HRP (Thermo Scientific, 32230) and anti-rabbit HRP (Abcam, ab6721) were used at 1:10,000 dilutions. HRP was activated using SuperSignal West Pico Chemiluminescent Substrate (Thermo Scientific) for autoradiography.

### RNA isolation from cell lines

RNA was isolated from cell lines using RNeasy^®^ Mini Kit (Qiagen) according to the manufacturer's protocol with an addition of DNase (Qiagen) treatment step after the first wash to remove DNA contamination.

### Next generation RNA-sequencing

RNA isolated from cell lines was assessed for quality using BioRad Experion with an RNA integrity number (RIN) ≥ 8 as threshold for high quality, suitable for RNA-sequencing. Library preparation was performed using Scriptseq™ Complete Gold (Human/Mouse/Rat) (Illumina) and sequenced on Illumina HiSeq 2500 with six samples run per flow cell. The 100 bp paired-end strand-specific sequences were mapped to the human genome release hg19 using TopHat with 2 mismatches allowed for full-length reads. Raw reads were mapped to human mRNAs annotated in the RefSeq database and lincRNAs annotated in Cabili et al. [52] using Cufflinks V2.0.2. Expression values were calculated as FPKM (fragment per kilobase of exon per million of mapped fragments). Transcripts were considered expressed if FPKM values across either all TzR samples or all TzS samples were ≥ 1.0 for mRNA and ≥ 0.25 for lincRNAs. The mean expression values were calculated for expressed transcripts followed by fold change (TzR/TzS) with statistical significance calculated using a paired *t*-test. Transcripts were defined as differentially expressed if the fold change was ≥ 2.0 or ≤ 0.5 and *p* < 0.05. Heatmaps were generated in R using heatmap.2 with Z scores scaled by row using standard Z score calculation of log fold change.

### Quantitative Real-time PCR (qRT-PCR)

RNA was converted to cDNA using RNA to cDNA EcoDry™ Premix Random Hexamers (Clontech). Primer pairs were designed using primer3 software (Untergrasser et al., 2007) with most spanning exon-exon boundaries. Maxima SyBr Green/ROX qPCR Master Mix (Thermo Scientific) was used for qRT-PCR. A comparative C_T_ quantitation was performed with a hold stage of 50°C for 2 min and 95°C for 10 min followed by 40× cycles of 95°C for 15 sec and 60°C for 1 min, and finally a melt curve at 95°C for 15 sec, 60°C for 1 min, and a ramp to 95°C at 0.3°C increments. Analysis was done using the 2ΔΔC_T_ method with GAPDH as the reference gene [53].

### Apoptosis assay

Cells were plated at 5000 cells/well in a 96-well plate. After 5 hours, the media was replaced and trastuzumab (10 μg/ml) was added. After 48 hours, Caspase-Glo^®^ 3/7 Assay (Promega, G8091) reagent was added to the wells. The plate was incubated for 1 hr at room temperature and the luminescence was read by spectrophotometer. The luminescence reading was normalized to the mock treatment of each cell line to calculate relative apoptosis.

### ChIP-seq and analysis

Cells were crosslinked, sonicated, chromatin immunoprecipitated and converted into libraries for deep sequencing. For each ChIP, 6 μg of anti-Histone H3K4 monomethyl (Abcam ab8895) or anti-Histone H3K27 acetyl (Abcam ab4729) were bound to 100 μl of protein G dynabeads (Life Technologies 10004D) and incubated with sonicated lysate from 5 million crosslinked cells. Immunoprecipitated DNA fragments were end-repaired, received an overhanging A base, and then ligated to Illumina TruSeq indexed adapters [700 nM] that were annealed from oligonucleotides (IDT, HPLC-purified). Sample clean-up between steps was performed with PCRCleanDx beads (Aline Biosciences). Adapter-modified DNA fragments were PCR-amplified and size-selected on agarose gel for 250–350 bp. ChIP-seq libraries were sequenced on the HiSeq 2500 platform at the Case Western Reserve University Genomics Core Facility. The FASTX-Toolkit 0.0.13 (http://hannonlab.cshl.edu/fastx_toolkit/) was used to remove adapter sequences and trim read ends using a quality score cutoff of 20. ChIP-seq data were aligned to the hg19 genome assembly (retrieved from http://cufflinks.cbcb.umd.edu/igenomes.html), using Bowtie v0.12.9 [54], allowing reads with ≤ 2 mismatches and discarding reads with > 1 reportable alignment (“-m 1” parameter). PCR duplicates were removed using SAMtools. Peaks were detected with MACS v1.4 with a threshold for significant enrichment of *P* < 1E-9. Wiggle tracks stepped at 25 bp were generated by MACS, normalized to the mean whole-genome WIG signal and visualized on the UCSC Genome Browser.

### Soft agar assays

50,000 BT474 shS100P or BT474 shGFP cells were suspended in 0.6% type VII agarose (Sigma-Aldrich) in 10% FBS RPMI medium and plated onto a bottom layer of 1.2% agar in 10% FBS RPMI medium. Cells were plated onto 60mm plates in quadruplicate. 1mL of 10% FBS RPMI with 10 μg/mL trastuzumab was added 24 hours after plating. The medium was changed every 2 days, with fresh trastuzumab added, until cells were analyzed after 3 weeks. To quantify colonies, each plate was scanned using an automated multipanel scanning microscope and the digital images were analyzed using MetaMorph image quantification software.

## SUPPLEMENTARY MATERIAL FIGURES AND TABLES





















## Abbreviations

TzS: Trastuzumab-sensitive
TzR: Trastuzumab-Resistant.
