# Supplementary material for: Transcriptome-wide identification of mRNAs and lincRNAs associated with trastuzumab-resistance in HER2-positive breast cancer
Source: Oncotarget. 2016 Jul 16;7(33):53230–44. doi: 10.18632/oncotarget.10637 (PMC5288181; doi:10.18632/oncotarget.10637)
Supplement: Supplementary file 1 [file oncotarget-07-53230-s001.pdf]

Transcriptome-wide identification of mRNAs and lincRNAs associated with trastuzumab-resistance in HER2-positive breast cancer

Supplementary Materials

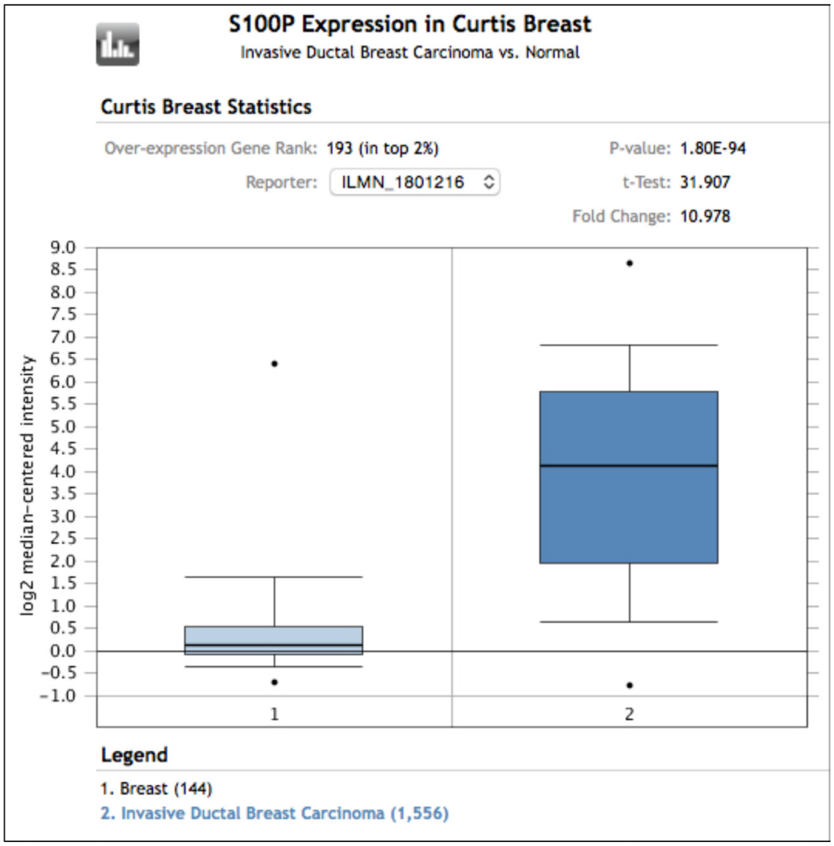

Supplementary Figure S1: S100P is highly up-regulated in breast cancer tissue (*n* = 1,556) when compared to normal breast (*n* = 144), *p* = 1.8E-94. S100P is in the top 2% of up-regulated genes.

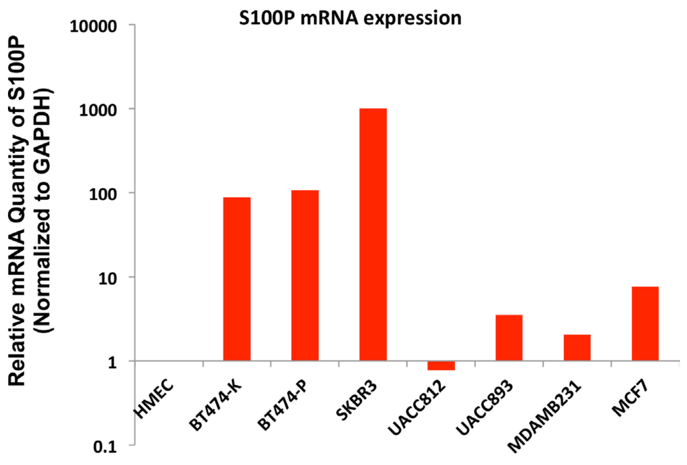

Supplementary Figure S2: S100P is up-regulated in breast cancer cell lines as compared to normal human mammary epithelial cells. The highest expression is observed in BT474 and SKBR3 (both are HER2+ cell lines).

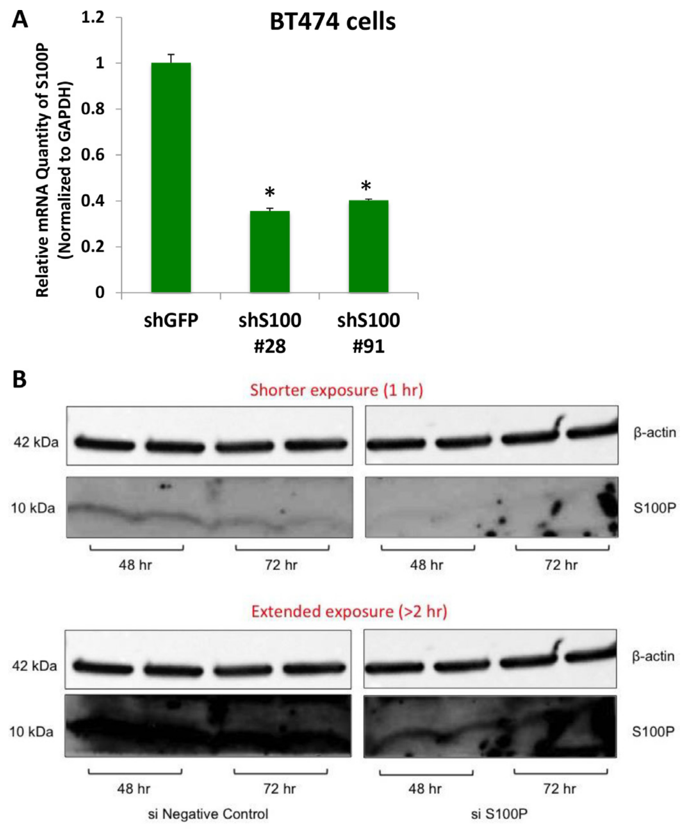

Supplementary Figure S3: Knock down of S100P. (**A**) Using two distinct shRNAs, we were able to deplete S100P mRNA levels by 65% and 60%, respectively, as compared to shRNA targeting GFP. Measurements were done at 48 hours post knock down. (**B**) To determine the time required to deplete S100P protein post mRNA knock down, we performed western blot analysis at 48 and 72 hours post KD and observed some depletion at 48 hours but substantial depletion at 72 hours. Both short and long exposures of the blot are shown, however, the long exposure results in higher background.

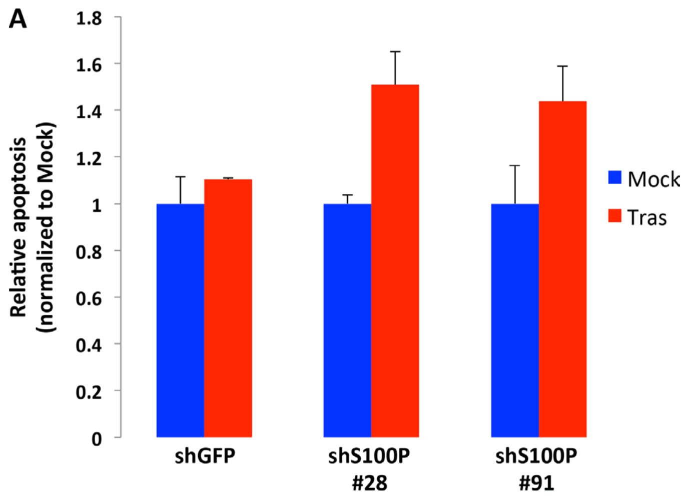

Supplementary Figure S4: (A) Knock down of S100P leads to increased apoptosis in TzR BT474 cells. Utilizing the Caspase 3/7 apoptotic assay, we found that TzR cells show increased apoptosis when treated with trastuzumab (Tras) as compared to mock treated cells ($p < 0.01$).

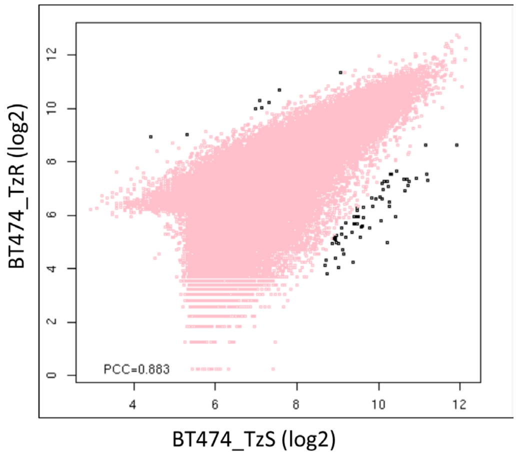

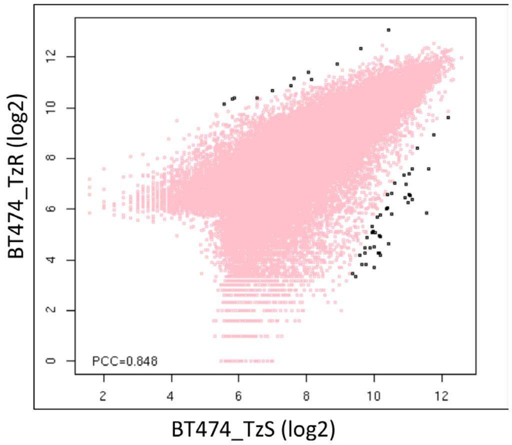

Supplementary Figure S5: Epigenetic changes at enhancers in TzR cells. We examined genome-wide changes in H3K27ac and H3K4me1 (markers of active enhancers) in TzS and TzR BT474, and identified enhancer changes in TzR cells as compared to TzS cells ($p < 0.05$).

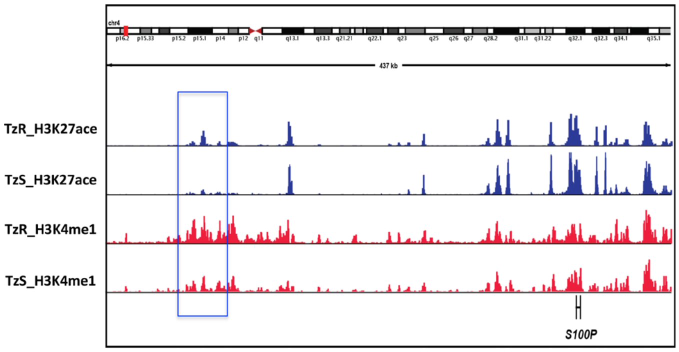

Supplementary Figure S6: A potential key enhancer regulating S100P. A snapshot of chromatin marks, H3K4me1 and H3K27ac, at an active enhancer regulating S100P. Both chromatin marks demonstrate increased enhancer activity in TzR vs TzS cells. The genomic location of S100P gene is indicated.

Supplementary File S1: Diff Expressed mRNAs_TzS vs TzR. See Supplementary_File_S1

Supplementary File S2: Diff Expressed lincRNAs_TzS vs TzR. See Supplementary_File_S2

Supplementary File S3: Diff Expressed mRNAs_BT474 (TzS vs TzR). See Supplementary_File_S3

Supplementary File S4: Diff Expressed lincRNAs_BT474 (TzS vs TzR). See Supplementary_File_S4

Supplementary File S5: Diff Expressed mRNAs_BT474 (TzS)_Mock vs Tras. See Supplementary_File_S5

Supplementary File S6: Diff Expressed lincRNAs_BT474 (TzS)_Mock vs Tras. See Supplementary_File_S6

Supplementary File S7: Top mRNAs and lincRNAs associated with TzR. See Supplementary_File_S7

Supplementary File S8: H3K4me1 ChIP-seq_BT474 cells. See Supplementary_File_S8

Supplementary File S9: H3K27ace_ChIP-seq_BT474 cells. See Supplementary_File_S9
