# Supplementary material for: Transcriptome-wide identification of mRNAs and lincRNAs associated with trastuzumab-resistance in HER2-positive breast cancer
Source: Oncotarget. 2016 Jul 16;7(33):53230–44. doi: 10.18632/oncotarget.10637 (PMC5288181; doi:10.18632/oncotarget.10637)
Supplement: Supplementary file 9 [file oncotarget-07-53230-s009.docx]

id baseMean BT474-J (TzS) BT474-R-J (TzR) foldChange log2FoldChange pval padjchr10_110319638_110320442 31.17783043 62.35566086 0 0 #NAME? 0.02269542 1chr8_8621083_8624109 258.281162 21.35467838 495.2076455 23.189656 4.535409514 1.58E-06 0.005898933chr12_107942761_107944505 104.3184169 11.95861989 196.6782138 16.44656454 4.039714351 0.002379737 0.68600337chr5_171022211_171024515 179.9545942 23.91723978 335.9919486 14.04810721 3.812303855 0.00024501 0.205863413chr8_37335851_37338228 222.5899151 30.75073686 414.4290934 13.47704594 3.752432399 8.46E-05 0.105439082chr1_67858061_67861172 280.1278671 39.29260821 520.9631259 13.25855293 3.72885142 2.16E-05 0.03914869chr4_78147471_78149193 118.5880148 17.0837427 220.0922869 12.88314222 3.687412607 0.002730632 0.723644078chr1_94428729_94430018 57.96504549 9.396058486 106.5340325 11.33816192 3.503114872 0.041378735 1chr7_8316931_8318570 89.05158815 15.37536843 162.7278079 10.58366885 3.403767922 0.013777719 1chr6_39959131_39962743 197.3721054 34.1674854 360.5767254 10.55321225 3.399610296 0.00052967 0.303758941chr11_121383442_121386960 310.6442725 54.66797664 566.6205684 10.36476203 3.373615088 5.07E-05 0.069672958chr1_114552773_114553839 62.33134358 11.10443276 113.5582544 10.22638949 3.354224974 0.041055405 1chr1_48965579_48967632 105.3937581 18.79211697 191.9953992 10.21680524 3.352872236 0.008536516 1chr10_54711279_54712797 70.20975264 12.81280703 127.6066983 9.959308526 3.31604558 0.031685645 1chr6_4829172_4830317 50.94082357 9.396058486 92.48558865 9.843019686 3.29910098 0.069572478 1chrX_152721420_152722681 55.46537992 10.25024562 100.6805142 9.822253822 3.296054104 0.05793644 1chr12_109611153_109612182 40.7210072 7.687684216 73.75433019 9.593829314 3.262106773 0.111354068 1chr1_59697224_59698683 66.69764168 12.81280703 120.5824763 9.411089708 3.234361782 0.04027311 1chr20_60576892_60582120 700.6732021 135.8157545 1265.53065 9.317995947 3.220019703 1.05E-06 0.004542259chr1_76570683_76572199 61.5877335 11.95861989 111.2168471 9.300140662 3.217252537 0.049855652 1chr1_93366949_93368231 61.5877335 11.95861989 111.2168471 9.300140662 3.217252537 0.049855652 1chr2_232347243_232348403 56.47782532 11.10443276 101.8512179 9.172122531 3.197255628 0.062045018 1chr17_64759479_64761502 142.2861378 28.18817546 256.3841002 9.095448571 3.185144791 0.00404899 0.900319516chr7_96631398_96633074 94.85742521 18.79211697 170.9227334 9.095448571 3.185144791 0.016375338 1chr17_9972224_9973248 42.90415625 8.541871351 77.26644115 9.045610496 3.177217875 0.108964765 1chr1_106926530_106928388 78.94234884 16.22955557 141.6551421 8.728220654 3.125687574 0.03036863 1chr8_8922169_8926914 922.7710905 191.3379183 1654.204263 8.645459707 3.111942679 5.18E-07 0.002551931chr3_58992101_58993387 69.89323612 14.5211813 125.265291 8.626384342 3.108755996 0.042315371 1chr19_19389142_19390613 85.65005424 17.93792984 153.3621786 8.549602994 3.095857429 0.025521619 1chr1_118486398_118487428 48.441158 10.25024562 86.63207038 8.451706777 3.079242715 0.095502751 1chr8_111166609_111168476 92.35775964 19.64630411 165.0692152 8.402049275 3.070741247 0.021543792 1chr3_52471089_52473894 243.8035872 52.10541524 435.5017592 8.358090176 3.063173324 0.000593401 0.328153869chr8_11285675_11288826 274.5736134 59.79309945 489.3541273 8.184123782 3.032827966 0.000383168 0.267943121chr1_48959475_48960765 66.38112516 14.5211813 118.241069 8.142661855 3.025500492 0.053169182 1chr8_11279849_11283086 205.8510809 45.27191816 366.4302436 8.093985378 3.016850243 0.001453226 0.554625916chr8_10516358_10520946 576.9904937 127.2738831 1026.707104 8.06691113 3.012016362 1.06E-05 0.02304646chr4_103395802_103397112 71.91812691 16.22955557 127.6066983 7.862611995 2.975008662 0.047329603 1chr14_77049258_77051980 221.2913825 50.39704097 392.185724 7.781919662 2.960126086 0.00128775 0.524256232chr16_967339_969817 235.8774969 53.81378951 417.9412044 7.766433255 2.957252191 0.000993003 0.452229537chr11_125800857_125802389 88.1020386 20.50049124 155.7035859 7.595114874 2.925071785 0.031084076 1chr1_48296074_48298585 212.6693633 49.54285383 375.7958729 7.58526899 2.923200342 0.001707509 0.594931757chr6_164162447_164163825 69.57671961 16.22955557 122.9238836 7.574075775 2.921069855 0.054790781 1chr1_105680031_105682130 142.5072919 33.31329827 251.7012856 7.555579863 2.917542481 0.007625127 1chr1_112221431_112223194 94.22439217 22.20886551 166.2399188 7.485295399 2.904059252 0.027020206 1chr1_86053123_86055703 298.8570882 70.89753221 526.8166442 7.43067675 2.89349361 0.000448669 0.287756246chr8_11442954_11448090 672.2425459 159.7329943 1184.752098 7.417078125 2.890850965 1.05E-05 0.02304646chr1_242007149_242009270 146.87359 35.02167254 258.7255075 7.387582852 2.885102405 0.007444953 1chr1_64139403_64142065 182.1681725 43.56354389 320.7728011 7.363331182 2.880358591 0.00353048 0.846604513chr13_33641514_33642500 49.88069696 11.95861989 87.80277403 7.342216312 2.876215619 0.110317749 1chr8_8681196_8684568 273.4658055 65.7724094 481.1592017 7.315517344 2.870959893 0.000719468 0.371344945chr12_66284846_66291830 588.933899 141.7950644 1036.072734 7.306832137 2.869246064 2.20E-05 0.039152884chr3_72846621_72847813 56.58840236 13.66699416 99.50981057 7.281031176 2.864142787 0.089135801 1chr8_125088915_125090907 176.4729125 42.70935675 310.2364682 7.263899338 2.86074421 0.004187048 0.904043654chr1_84583147_84584209 62.71075594 15.37536843 110.0461435 7.157301235 2.839415701 0.075319035 1chr11_59421934_59423915 146.1299799 35.87585967 256.3841002 7.146423877 2.837221487 0.008489645 1chr8_8656598_8659414 263.6730827 64.91822226 462.4279432 7.123237931 2.83253318 0.000965746 0.443543229chr1_2015719_2018184 214.9630894 52.95960237 376.9665765 7.118002395 2.831472418 0.002188337 0.655225714chr2_218620966_218621833 57.60084776 14.5211813 100.6805142 6.933355639 2.793553764 0.093216147 1chr1_93995430_93997826 135.3248117 34.1674854 236.4821381 6.921262577 2.791035238 0.012026748 1chr8_11163419_11165029 81.07781667 20.50049124 141.6551421 6.909841351 2.788652587 0.046829943 1chr2_173880783_173881903 46.95393783 11.95861989 81.94925576 6.852735225 2.776679945 0.133869887 1chr2_191850592_191851817 73.78475944 18.79211697 128.7774019 6.852735225 2.776679945 0.058537585 1chr3_155926755_155929016 164.3387824 41.85516962 286.8223952 6.852735225 2.776679945 0.006704952 1chr4_73969180_73971158 117.3848446 29.89654973 204.8731394 6.852735225 2.776679945 0.018780197 1chrX_13512531_13514434 114.0309919 29.04236259 199.0196211 6.852735225 2.776679945 0.020353206 1chr7_149359096_149360665 110.0917873 28.18817546 191.9953992 6.811203496 2.767909736 0.022774589 1chr4_158729434_158732469 166.5219314 42.70935675 290.3345061 6.797913343 2.765091971 0.006613031 1chr5_1226561_1229114 338.5808646 87.12708778 590.0346415 6.77211481 2.759606432 0.000452758 0.288670727chr1_51870245_51871287 49.7224387 12.81280703 86.63207038 6.761365422 2.75731462 0.124471497 1chr2_128363126_128365739 217.8421674 56.37635091 379.3079838 6.728140039 2.750207734 0.002689967 0.721690234chr19_16636324_16639614 454.6367473 117.8778246 791.3956699 6.71369422 2.74710683 0.000135934 0.145204764chr10_54699188_54701995 180.9497876 46.98029243 314.9192829 6.703221001 2.744854499 0.005278587 0.9889824chr8_63014186_63015907 124.5196435 32.45911113 216.5801759 6.672400087 2.738205798 0.01720491 1chr1_84689655_84691896 192.609143 50.39704097 334.821245 6.643668726 2.731980138 0.004415573 0.92215716chr3_174200476_174203097 140.1182034 36.73004681 243.50636 6.629622915 2.728926814 0.012454272 1chr12_4699064_4701489 175.8398795 46.12610529 305.5536536 6.624310717 2.727770345 0.006090996 1chr1_60441883_60442977 51.90558775 13.66699416 90.14418134 6.595757654 2.721538391 0.120254254 1chr12_65894033_65896552 223.8062627 58.93891232 388.673613 6.594516216 2.721266825 0.002670556 0.720046924chr8_10405072_10406432 84.27341112 22.20886551 146.3379567 6.589168485 2.720096417 0.047567939 1chr1_73509316_73510992 116.6412345 30.75073686 202.5317321 6.586239966 2.719455077 0.021405707 1chr3_53123282_53125599 178.0230285 46.98029243 309.0657646 6.578625816 2.717786256 0.006005935 1chr7_111609634_111611505 112.70203 29.89654973 195.5075102 6.539467329 2.709173126 0.023930434 1chr7_69562349_69563986 93.16426557 24.77142692 161.5571042 6.521913524 2.705295312 0.038702024 1chr8_2533786_2534836 51.32023592 13.66699416 88.97347769 6.510098463 2.702679364 0.12479377 1chr18_19166411_19169264 172.9131203 46.12610529 299.7001354 6.497408213 2.699864348 0.006936069 1chr7_28580993_28582985 147.2530024 39.29260821 255.2133965 6.495201213 2.699374219 0.011488347 1chr2_8879689_8880780 47.96638322 12.81280703 83.11995942 6.487256013 2.697608374 0.139359477 1chr7_55187297_55190713 322.9346236 86.27290064 559.5963465 6.486351361 2.697407176 0.000710562 0.36850309chr8_93121615_93122648 44.61253052 11.95861989 77.26644115 6.461150355 2.691791048 0.156008337 1chr21_43776434_43779153 251.4912714 67.48078367 435.5017592 6.453715199 2.690129913 0.001924341 0.630143056chr9_16191583_16192668 54.0887368 14.5211813 93.6562923 6.449633153 2.689217104 0.116168634 1chr22_21082386_21084496 123.7760335 33.31329827 214.2387686 6.431028442 2.68504947 0.019525353 1chr1_74082499_74084101 101.4697682 27.33398832 175.6055481 6.424439273 2.683570541 0.032727693 1chrX_4579514_4580537 50.7348841 13.66699416 87.80277403 6.424439273 2.683570541 0.129493113 1chr13_36681112_36683906 180.6332711 48.6886667 312.5778756 6.419930895 2.682557768 0.006300685 1chr11_71106970_71108425 79.16350293 21.35467838 136.9723275 6.41416017 2.68126038 0.057659992 1chr12_59352496_59354509 119.8368289 32.45911113 207.2145467 6.383863867 2.674429887 0.021770315 1chr5_89379628_89381056 72.45579753 19.64630411 125.265291 6.376023209 2.672656881 0.069866842 1chr7_32357658_32359176 72.45579753 19.64630411 125.265291 6.376023209 2.672656881 0.069866842 1chr11_60555698_60558273 179.4625675 48.6886667 310.2364682 6.371841525 2.671710386 0.00662897 1chr3_66347638_66348544 44.02717869 11.95861989 76.09573749 6.363254137 2.669764741 0.162050547 1chr12_70017160_70018668 113.1291235 30.75073686 195.5075102 6.357815458 2.668531141 0.025611254 1chr1_117262271_117263991 100.2990645 27.33398832 173.2641408 6.338780083 2.664205216 0.034815514 1chr1_116634600_116636197 87.46900556 23.91723978 151.0207713 6.314306028 2.658624184 0.048126162 1chr3_31734072_31735865 96.35986001 26.47980119 166.2399188 6.27798969 2.65030266 0.039184972 1chr7_134654411_134656428 133.0939815 36.73004681 229.4579161 6.24714467 2.64319694 0.017467324 1chr6_82543520_82545066 95.77450818 26.47980119 165.0692152 6.233778495 2.640106892 0.040423273 1chr12_65492914_65493818 40.08797416 11.10443276 69.07151557 6.22017505 2.636955182 0.189128305 1chr12_95170588_95172331 80.17594833 22.20886551 138.1430311 6.22017505 2.636955182 0.059840482 1chr8_8607893_8611993 381.9273291 105.9192047 657.9354534 6.211672897 2.63498186 0.000473898 0.293516234chr9_109742362_109743798 98.54300906 27.33398832 169.7520298 6.210291297 2.63466094 0.038190544 1chr1_87983355_87984368 52.33268132 14.5211813 90.14418134 6.207771909 2.63407555 0.129541169 1chr8_15094275_15095379 52.33268132 14.5211813 90.14418134 6.207771909 2.63407555 0.129541169 1chr1_3424804_3428874 325.6554432 90.54383632 560.7670501 6.19332108 2.630713242 0.000899793 0.427752972chr10_48700522_48704399 257.7242021 71.75171934 443.6966848 6.183777738 2.628488466 0.002176278 0.65462988chr5_127193694_127194693 45.62497591 12.81280703 78.4371448 6.121776801 2.613950445 0.16179424 1chr19_5934669_5938516 355.523601 99.9398948 611.1073073 6.114748354 2.612293127 0.000692003 0.364104517chr5_174880852_174882173 72.8828911 20.50049124 125.265291 6.110355575 2.611256336 0.074929599 1chr7_55144782_55148065 279.2868572 78.58521643 479.988498 6.1078727 2.610669994 0.001730342 0.599201256chr8_8846173_8850780 392.6848161 111.0443276 674.3253046 6.072577676 2.602309039 0.000491284 0.295831887chr8_8976615_8980194 388.7456115 110.1901404 667.3010826 6.055905547 2.598342704 0.00052003 0.303040381chr8_8646385_8649835 216.8926178 61.50147372 372.2837619 6.053249448 2.597709804 0.004390844 0.920540035chrX_76986980_76988097 51.16197766 14.5211813 87.80277403 6.04653108 2.5961077 0.139239907 1chr3_23979773_23980892 66.17518569 18.79211697 113.5582544 6.042866516 2.595233074 0.091391832 1chr21_41212089_41214220 171.2676419 48.6886667 293.8466171 6.035215935 2.59340539 0.009451675 1chr1_54579297_54581429 165.1452883 46.98029243 283.3102842 6.030406998 2.592255374 0.010576658 1chr1_110732182_110733826 122.8741651 35.02167254 210.7266577 6.017035807 2.589052942 0.024205188 1chr18_76459007_76460952 196.7695016 56.37635091 337.1626523 5.980568923 2.580282733 0.006358027 1chr11_78643824_78645285 88.90854452 25.62561405 152.191475 5.939037195 2.570229068 0.053292378 1chr13_41126333_41128357 160.0353801 46.12610529 273.944655 5.939037195 2.570229068 0.012255566 1chr20_25070187_25072878 358.402679 103.3566433 613.4487146 5.935261583 2.569311615 0.000804442 0.398139957chr10_62291368_62293598 147.790673 42.70935675 252.8719892 5.920763234 2.565783163 0.015529796 1chr7_52834738_52835804 56.11362759 16.22955557 95.99769961 5.91499251 2.564376342 0.124753184 1chr1_76895544_76897759 150.5591739 43.56354389 257.5548038 5.912163723 2.563686222 0.014817257 1chr9_80259008_80263565 300.5329959 87.12708778 513.938904 5.898726987 2.560403638 0.001596974 0.579556751chr12_109562159_109565726 297.1791432 86.27290064 508.0853857 5.889281361 2.5580916 0.001677449 0.590315097chr2_61508931_61510138 52.75977489 15.37536843 90.14418134 5.862895692 2.55161339 0.138932996 1chr8_11410412_11412208 146.0346175 42.70935675 249.3598782 5.838530411 2.545605281 0.016813317 1chr17_61063138_61066016 256.5058172 75.16846788 437.8431665 5.824824941 2.542214692 0.002976504 0.753785607chr5_141066091_141068109 139.9122639 41.00098248 238.8235454 5.824824941 2.542214692 0.019019475 1chr2_152846459_152853094 527.4394906 154.6078714 900.2711097 5.82293192 2.541745751 0.00021042 0.189981773chr6_89661397_89663037 110.4711997 32.45911113 188.4832883 5.806791427 2.537741215 0.034706143 1chr14_21559752_21561647 150.9862674 44.41773102 257.5548038 5.798468267 2.535671846 0.015699114 1chr16_3988786_3989620 37.74656686 11.10443276 64.38870096 5.798468267 2.535671846 0.220290063 1chr5_72187820_72190749 203.1606905 59.79309945 346.5282815 5.795456076 2.534922199 0.006548689 1chr1_93414325_93418474 269.7629697 79.43940356 460.0865359 5.791666545 2.533978542 0.00257559 0.70682191chr7_37550656_37552417 144.8639138 42.70935675 247.0184709 5.78370853 2.531994849 0.017726102 1chr4_124321986_124324832 188.1474825 55.52216378 320.7728011 5.777382928 2.53041612 0.008407027 1chr16_20624719_20626744 106.5319952 31.604924 181.4590663 5.741480864 2.52142289 0.038878896 1chr9_86158924_86159922 46.05206948 13.66699416 78.4371448 5.739165751 2.520841041 0.173457565 1chr8_11272347_11276700 347.2809942 103.3566433 591.2053451 5.720051716 2.516028191 0.001127461 0.49246144chrX_149406610_149409016 149.2302119 44.41773102 254.0426929 5.719398245 2.515863365 0.016974586 1chr10_46513177_46517050 249.6398535 74.31428075 424.9654263 5.718489394 2.515634093 0.003567484 0.850907414chr8_10492197_10495089 266.8362106 79.43940356 454.2330177 5.71798122 2.515505882 0.002851997 0.740026776chr7_68838136_68839152 51.58907123 15.37536843 87.80277403 5.710612687 2.51364554 0.149079576 1chr12_9011776_9013612 123.1430004 36.73004681 209.555954 5.705300489 2.512302873 0.028083244 1chr3_66605346_66606831 91.51878714 27.33398832 155.7035859 5.696336155 2.510034286 0.055123012 1chr15_82232325_82234698 111.4836451 33.31329827 189.6539919 5.693041571 2.509199634 0.035812342 1chrX_54037155_54038352 57.12607298 17.0837427 97.16840326 5.687770236 2.507863187 0.128822816 1chr8_36281062_36282379 62.66307473 18.79211697 106.5340325 5.669080959 2.503114872 0.111857967 1chr13_100619577_100622671 168.0243662 50.39704097 285.6516915 5.668025067 2.502846139 0.012603683 1chr3_66886025_66888123 128.0946503 38.43842108 217.7508796 5.664927786 2.502057565 0.026013211 1chr12_75549159_75550415 65.43157561 19.64630411 111.2168471 5.660955186 2.501045503 0.104406473 1chr18_74203620_74204879 113.6667941 34.1674854 193.1661029 5.65350656 2.49914597 0.03488137 1chr2_65412530_65414713 164.6705135 49.54285383 279.7981732 5.64759903 2.497637663 0.013516676 1chr8_123744727_123746797 144.7056556 43.56354389 245.8477673 5.643429008 2.496572026 0.019260985 1chr2_143498887_143500578 84.81108173 25.62561405 143.9965494 5.619242884 2.49037576 0.066201996 1chr7_122342795_122343912 56.54072115 17.0837427 95.99769961 5.619242884 2.49037576 0.133309036 1chr10_43428337_43430890 242.7738899 73.46009361 412.0876861 5.609680928 2.487918714 0.004283459 0.91245684chr1_59431456_59433028 93.11658436 28.18817546 158.0449933 5.606783366 2.487173328 0.055151559 1chrX_91034812_91036324 62.0777229 18.79211697 105.3633288 5.606783366 2.487173328 0.11566138 1chr9_110556271_110557598 67.61472465 20.50049124 114.7289581 5.596400433 2.484499194 0.100796551 1chr8_21198348_21201278 219.4551792 66.62659653 372.2837619 5.587614875 2.482232587 0.006014043 1chr4_1957992_1961135 210.5643248 64.06403513 357.0646144 5.573557983 2.478598592 0.006909957 1chr12_71742608_71743615 47.6498667 14.5211813 80.77855211 5.562808594 2.475813466 0.172526631 1chr16_8737886_8739035 53.18686845 16.22955557 90.14418134 5.554322235 2.473610878 0.148420885 1chr8_111154148_111156853 212.7474738 64.91822226 360.5767254 5.554322235 2.473610878 0.00679415 1chr1_48151768_48153384 117.4477404 35.87585967 199.0196211 5.547452325 2.471825364 0.034038608 1chr2_139571346_139572671 61.49237108 18.79211697 104.1926252 5.544485773 2.471053663 0.119585478 1chr5_169149797_169152727 181.1232605 55.52216378 306.7243573 5.524358858 2.465807039 0.011195683 1chr17_17779522_17782455 235.9079262 72.60590648 399.2099459 5.498312263 2.458988843 0.005152232 0.984912365chr8_8890729_8892947 717.0417266 221.234468 1212.848985 5.48218818 2.45475185 0.000110575 0.125978377chr1_92051262_92052266 49.83301575 15.37536843 84.29066307 5.48218818 2.45475185 0.165591085 1chr18_34054119_34055621 96.89753063 29.89654973 163.8985115 5.48218818 2.45475185 0.053535368 1chr2_157963682_157965100 83.05502625 25.62561405 140.4844385 5.48218818 2.45475185 0.072603031 1chr5_21078328_21079711 91.36052888 28.18817546 154.5328823 5.48218818 2.45475185 0.060336196 1chr6_125873187_125874305 55.3700175 17.0837427 93.6562923 5.48218818 2.45475185 0.142720388 1chr9_110904692_110905656 44.296014 13.66699416 74.92503384 5.48218818 2.45475185 0.19322597 1chrX_115008236_115011233 168.2932016 52.10541524 284.4809879 5.459720195 2.448827016 0.014370733 1chr5_139598591_139600579 121.2286867 37.58423394 204.8731394 5.451039383 2.446531344 0.033201278 1chr2_3501908_3503555 96.3121788 29.89654973 162.7278079 5.443029693 2.444409906 0.055130858 1chr1_51337820_51340574 170.4763506 52.95960237 287.9930988 5.437976985 2.443070045 0.014073105 1chr11_118249495_118250854 76.93267267 23.91723978 129.9481056 5.433240071 2.441812795 0.085090974 1chr3_50515321_50517618 145.5598427 45.27191816 245.8477673 5.430469423 2.441076914 0.021491438 1chr5_170131351_170132535 60.32166742 18.79211697 101.8512179 5.419890587 2.438263728 0.127809287 1chr1_73311141_73312273 54.78466567 17.0837427 92.48558865 5.413660827 2.436604504 0.147653734 1chr1_55614992_55618457 210.8331601 65.7724094 355.8939107 5.410990931 2.435892823 0.007803426 1chr10_45347552_45349509 104.0323296 32.45911113 175.6055481 5.410054125 2.435643028 0.047632146 1chr2_167828237_167829223 46.47916305 14.5211813 78.4371448 5.401567765 2.4333782 0.185157657 1chr17_77301316_77304145 308.7431361 96.52314626 520.9631259 5.397287035 2.432234414 0.002356865 0.681221876chr1_84555789_84557101 87.42132435 27.33398832 147.5086604 5.396528989 2.432031774 0.068105348 1chr8_28531369_28532950 87.42132435 27.33398832 147.5086604 5.396528989 2.432031774 0.068105348 1chr2_218889032_218892536 250.6046177 78.58521643 422.624019 5.377907426 2.427044921 0.004722214 0.9476376chr12_65297813_65298983 65.27331735 20.50049124 110.0461435 5.367975926 2.424378201 0.114810216 1chr3_18465204_18467692 146.5722881 46.12610529 247.0184709 5.355285675 2.420963537 0.02209259 1chr4_62134130_62135450 56.96781472 17.93792984 95.99769961 5.35165989 2.419986432 0.141968763 1chr1_95722438_95724371 135.4982846 42.70935675 228.2872125 5.345133475 2.418225974 0.026993689 1chr2_228785965_228787131 51.43081297 16.22955557 86.63207038 5.33792007 2.416277703 0.16455214 1chr7_22339761_22343287 278.71672 87.98127491 469.4521652 5.335819078 2.41570975 0.003499216 0.84471392chr20_24722095_24724562 227.2859071 71.75171934 382.8200948 5.335343853 2.415581253 0.00661828 1chr3_63984665_63986057 75.76196902 23.91723978 127.6066983 5.335343853 2.415581253 0.09056342 1chr1_113808938_113810403 100.0931251 31.604924 168.5813261 5.334020932 2.415223486 0.053460259 1chr19_16773598_16775689 145.9869363 46.12610529 245.8477673 5.329905175 2.414109866 0.022661954 1chr1_113090066_113092856 213.4434027 67.48078367 359.4060217 5.326049909 2.413065948 0.008044838 1chr17_60757415_60759872 162.0125897 51.2512281 272.7739513 5.322291024 2.412047399 0.017423525 1chr8_8894616_8896400 531.9315804 168.2748656 895.5882951 5.322175073 2.412015969 0.000395698 0.271451331chr12_56671741_56673759 172.5012414 54.66797664 290.3345061 5.310869799 2.408948161 0.014854018 1chr14_40528385_40529240 43.12531035 13.66699416 72.58362653 5.310869799 2.408948161 0.207526351 1chr2_1901974_1903915 139.8645827 44.41773102 235.3114344 5.297691462 2.405363823 0.025686854 1chrX_153080068_153081355 77.94511807 24.77142692 131.1188092 5.293147208 2.404125777 0.087516033 1chr6_38839121_38840250 56.3824629 17.93792984 94.82699595 5.286395745 2.402284431 0.146794775 1chr8_8877986_8884501 694.2130054 221.234468 1167.191543 5.27581237 2.399393257 0.000169923 0.165926142chr1_61706390_61709008 192.8931929 61.50147372 324.2849121 5.272799048 2.398569015 0.011220004 1chr2_220130367_220131624 69.63961544 22.20886551 117.0703654 5.271334788 2.398168322 0.106756677 1chr8_11330883_11332970 706.2994543 225.5054037 1187.093505 5.264146604 2.396199668 0.000163308 0.163591821chr10_17750483_17754534 307.2559159 98.23152053 516.2803113 5.255749972 2.393896645 0.002762925 0.726001089chr14_34398702_34403646 355.3328762 113.606889 597.0588634 5.25548115 2.393822852 0.001712523 0.594931757chr3_65956817_65958203 80.12826712 25.62561405 134.6309202 5.253763672 2.393351306 0.084594789 1chr7_55104452_55105165 170.7451859 54.66797664 286.8223952 5.246625406 2.391389789 0.015955978 1chr8_11458313_11459447 61.33411282 19.64630411 103.0219215 5.243832172 2.390621513 0.1314039 1chr12_65700178_65703063 226.542297 72.60590648 380.4786875 5.240326936 2.389656822 0.007235042 1chr3_71374943_71376551 87.84841791 28.18817546 147.5086604 5.232997808 2.387637655 0.07213289 1chr8_126943418_126945250 101.1055705 32.45911113 169.7520298 5.229718987 2.386733427 0.054934371 1chr12_110349996_110354462 334.7826664 107.627579 561.9377538 5.2211316 2.384362523 0.002165492 0.65427745chr1_64324930_64326161 55.79711107 17.93792984 93.6562923 5.2211316 2.384362523 0.151772184 1chr14_33378004_33379097 69.05426362 22.20886551 115.8996617 5.21862144 2.383668752 0.110185326 1chr7_52549026_52552046 241.3972467 77.73102929 405.0634642 5.211090962 2.381585438 0.006136421 1chr8_11308534_11310454 678.8832808 218.6719066 1139.094655 5.209149511 2.381047845 0.000203098 0.186555962chr13_100535606_100536638 53.02861019 17.0837427 88.97347769 5.208078771 2.380751269 0.163415418 1chr16_2103761_2106018 235.860245 76.02265502 395.697835 5.20499889 2.379897856 0.006613608 1chr1_14034381_14036265 103.2887195 33.31329827 173.2641408 5.201050324 2.378802997 0.053308201 1chr8_111714610_111716035 76.77441441 24.77142692 128.7774019 5.198626722 2.378130569 0.093061431 1chr1_59649494_59651867 161.2689796 52.10541524 270.432544 5.190104383 2.375763554 0.019240807 1chr12_105383317_105385670 148.0118271 47.83447956 248.1891746 5.188499527 2.375317383 0.023890007 1chr1_93422289_93425081 171.7576313 55.52216378 287.9930988 5.186993432 2.374898543 0.016370504 1chr12_49896641_49898384 108.2403694 35.02167254 181.4590663 5.181336389 2.373324251 0.048857552 1chr14_30205059_30206460 89.44621514 29.04236259 149.8500677 5.159706522 2.367289009 0.07192016 1chr9_117574216_117575539 65.70041091 21.35467838 110.0461435 5.153256889 2.365484512 0.121815414 1chr4_158647707_158651175 207.5898844 67.48078367 347.6989852 5.152562941 2.365290223 0.010012184 1chr3_194536928_194538763 86.67771426 28.18817546 145.1672531 5.149934351 2.364554041 0.076530333 1chr8_11499165_11500595 97.16636593 31.604924 162.7278079 5.148811871 2.364239558 0.061702775 1chr7_68912742_68913833 52.44325837 17.0837427 87.80277403 5.139551418 2.361642446 0.169004479 1chr12_97818700_97821418 235.7019867 76.87684216 394.5271313 5.131937268 2.359503535 0.007061832 1chr7_155745828_155747833 225.2133351 73.46009361 376.9665765 5.131583122 2.359403974 0.008068116 1chr3_59660449_59662892 172.7700767 56.37635091 289.1638025 5.129168486 2.358724963 0.016787053 1chr8_8952710_8955252 842.2877283 275.0482575 1409.527199 5.124654168 2.357454649 0.00011589 0.129497163chr5_134590808_134591882 49.67475749 16.22955557 83.11995942 5.121517905 2.356571457 0.182283262 1chr7_27187473_27189655 138.5356208 45.27191816 231.7993234 5.120156885 2.356188016 0.029208643 1chr5_56725196_56726446 78.37221164 25.62561405 131.1188092 5.116708968 2.355216177 0.092664331 1chr21_47028094_47030300 193.1620282 63.20984799 323.1142084 5.111770059 2.353822942 0.012624802 1chr14_22889918_22892098 182.6733765 59.79309945 305.5536536 5.110182553 2.35337483 0.014691904 1chr1_55036756_55038559 86.09236243 28.18817546 143.9965494 5.108402622 2.352872236 0.078823203 1chr7_55174966_55179212 380.7870547 124.7113217 636.8627876 5.106695839 2.352390133 0.001615654 0.579556751chr5_17128649_17130652 151.2074215 49.54285383 252.8719892 5.104106236 2.351658357 0.023915403 1chr16_936958_937979 46.90625662 15.37536843 78.4371448 5.101480667 2.350916039 0.196887926 1chr5_159601930_159604126 130.2301182 42.70935675 217.7508796 5.098435007 2.350054472 0.034125494 1chr1_50946855_50947648 36.41760494 11.95861989 60.87659 5.09060331 2.347836647 0.262737193 1chr12_64664507_64666100 98.76416316 32.45911113 165.0692152 5.085450877 2.346375689 0.061535482 1chr8_19400292_19401751 80.55536068 26.47980119 134.6309202 5.084287425 2.346045591 0.089512984 1chr21_37794196_37795809 106.484314 35.02167254 177.9469554 5.081052459 2.345127359 0.053090647 1chr8_8931494_8933404 617.9285804 203.2965381 1032.560623 5.079086108 2.344568933 0.000338428 0.249536908chr1_15141228_15142730 70.06670901 23.06305265 117.0703654 5.076100166 2.343720538 0.113098068 1chr10_74740555_74742492 147.8535688 48.6886667 247.0184709 5.073428535 2.342961025 0.025751798 1chr1_64148896_64149987 51.85790654 17.0837427 86.63207038 5.071024066 2.342277121 0.174768627 1chr11_119540724_119544233 417.0464014 137.5241287 696.568674 5.065065166 2.340580831 0.001261641 0.524256232chr5_73212864_73214014 56.80955646 18.79211697 94.82699595 5.046105029 2.335170235 0.155742244 1chr6_43357494_43359633 170.4286694 56.37635091 284.4809879 5.046105029 2.335170235 0.018439365 1chr4_146223269_146224506 64.52970726 21.35467838 107.7047361 5.043613125 2.334457617 0.129800938 1chr2_235303363_235305722 213.9810733 70.89753221 357.0646144 5.036347575 2.332377852 0.01010844 1chr19_4634070_4635323 77.20150798 25.62561405 128.7774019 5.025339165 2.329220968 0.098441064 1chr20_18523981_18525165 61.76120639 20.50049124 103.0219215 5.025339165 2.329220968 0.139278045 1chr16_89594414_89596976 174.7949675 58.08472518 291.5052098 5.018620797 2.327290941 0.01762586 1chr8_9007784_9010900 280.1085778 93.10639772 467.1107578 5.016956614 2.326812462 0.004695232 0.94552154chr1_167879866_167882116 141.1458634 46.98029243 235.3114344 5.008726473 2.324443828 0.029927169 1chr10_69231551_69232861 66.71285631 22.20886551 111.2168471 5.007768049 2.324167741 0.124956084 1chr8_103198925_103200623 110.2652602 36.73004681 183.8004736 5.004090373 2.323107845 0.051402504 1chr6_143940605_143943834 174.2096157 58.08472518 290.3345061 4.998465693 2.32148532 0.018038752 1chr1_84424731_84429199 342.8822296 114.4610761 571.303383 4.991245955 2.319399998 0.002584061 0.70682191chr1_9495050_9497020 97.00810768 32.45911113 161.5571042 4.977249795 2.315348794 0.066975781 1chr12_110133357_110134436 48.50405384 16.22955557 80.77855211 4.977249795 2.315348794 0.195057802 1chr1_86466010_86467120 61.17585456 20.50049124 101.8512179 4.968233038 2.312732846 0.143798776 1chr2_203637835_203638987 61.17585456 20.50049124 101.8512179 4.968233038 2.312732846 0.143798776 1chr8_8914255_8918170 376.9584272 126.419696 627.4971584 4.963602811 2.311387675 0.001978283 0.635654193chr7_9129068_9131430 201.1510143 67.48078367 334.821245 4.961727277 2.310842439 0.012726853 1chr1_71096329_71097826 86.519456 29.04236259 143.9965494 4.958155486 2.309803515 0.083223315 1chr2_223954165_223955944 86.519456 29.04236259 143.9965494 4.958155486 2.309803515 0.083223315 1chr8_41218519_41219629 66.12750448 22.20886551 110.0461435 4.955054701 2.308900984 0.128926847 1chr8_110462877_110464179 66.12750448 22.20886551 110.0461435 4.955054701 2.308900984 0.128926847 1chr1_57300969_57304373 312.4287199 105.0650176 519.7924223 4.947340552 2.306653211 0.003618981 0.856457846chr2_224816588_224817870 58.40735368 19.64630411 97.16840326 4.945887162 2.306229326 0.154520466 1chr14_34739354_34741217 109.0945566 36.73004681 181.4590663 4.940343999 2.304611501 0.054269536 1chr2_202871795_202873537 109.0945566 36.73004681 181.4590663 4.940343999 2.304611501 0.054269536 1chr1_59429738_59431219 88.70260505 29.89654973 147.5086604 4.933969362 2.302748757 0.080469139 1chr1_84347981_84350272 190.0770108 64.06403513 316.0899865 4.933969362 2.302748757 0.015114885 1chr18_68180574_68181997 76.03080433 25.62561405 126.4359946 4.933969362 2.302748757 0.104554884 1chr1_114117052_114120158 321.746668 108.4817662 535.0115698 4.93181102 2.302117519 0.003363595 0.824834202chr10_12553487_12557246 263.3393143 88.83546205 437.8431665 4.928698027 2.301206592 0.006150645 1chr9_116755841_116757254 80.98245425 27.33398832 134.6309202 4.925403443 2.300241901 0.094531527 1chr10_28688910_28690259 68.31065353 23.06305265 113.5582544 4.923817161 2.29977719 0.124122026 1chr11_78452983_78454751 98.6059049 33.31329827 163.8985115 4.919912469 2.298632649 0.066719049 1chr5_31711025_31712413 98.6059049 33.31329827 163.8985115 4.919912469 2.298632649 0.066719049 1chr7_68352045_68354865 197.2118098 66.62659653 327.7970231 4.919912469 2.298632649 0.013872597 1chr10_26726552_26728406 141.572957 47.83447956 235.3114344 4.919284929 2.29844862 0.031424266 1chr8_70655377_70656621 65.54215266 22.20886551 108.8754398 4.902341353 2.293470943 0.133014644 1chr2_205650862_205652478 83.1656033 28.18817546 138.1430311 4.900743979 2.29300078 0.091295288 1chr12_65911189_65912977 100.7890539 34.1674854 167.4106225 4.899705686 2.292695092 0.064635076 1chr1_111017687_111023273 632.2610742 214.4009709 1050.121177 4.897931073 2.292172471 0.000415646 0.276389353chr14_28314096_28315865 88.11725322 29.89654973 146.3379567 4.894810875 2.291253118 0.08281965 1chr5_28077615_28078711 52.87035193 17.93792984 87.80277403 4.894810875 2.291253118 0.179075336 1chr6_39005156_39006257 52.87035193 17.93792984 87.80277403 4.894810875 2.291253118 0.179075336 1chr6_40974931_40976169 70.49380258 23.91723978 117.0703654 4.894810875 2.291253118 0.119545133 1chr12_109941345_109942981 145.9392551 49.54285383 242.3356563 4.891435143 2.290257813 0.029833935 1chr8_8935203_8939042 1579.402853 536.4295208 2622.376184 4.888575447 2.289414119 3.21E-05 0.049747096chr16_15125831_15130818 507.726216 172.5458013 842.9066307 4.88511818 2.288393464 0.000848821 0.412569008chr1_7690223_7692105 98.02055307 33.31329827 162.7278079 4.884770237 2.288290704 0.068605656 1chr1_80489839_80491183 62.77365178 21.35467838 104.1926252 4.87914748 2.286629092 0.142695312 1chr8_99236820_99240284 359.01846 122.1487603 595.8881598 4.87838074 2.28640236 0.002549765 0.70682191chr4_158752713_158754536 107.9238529 36.73004681 179.117659 4.876597625 2.285874938 0.05728918 1chr1_61743761_61744942 72.67695163 24.77142692 120.5824763 4.867805022 2.283271383 0.115182967 1chr3_194704725_194708152 295.6594564 100.7940819 490.5248309 4.86660349 2.282915235 0.00465599 0.941730535chr3_168951792_168953576 110.107002 37.58423394 182.62977 4.85921225 2.280722451 0.055569241 1chr8_129871154_129872305 55.05350098 18.79211697 91.31488499 4.85921225 2.280722451 0.171730122 1chr2_118806981_118808888 115.0586519 39.29260821 190.8246956 4.856503659 2.279918049 0.050955984 1chr1_55061515_55063099 87.5319014 29.89654973 145.1672531 4.855652388 2.279665144 0.085234932 1chr19_16785602_16788248 157.4403521 53.81378951 261.0669148 4.851301445 2.278371827 0.025666369 1chr3_41903987_41905268 64.95680083 22.20886551 107.7047361 4.849628005 2.277874088 0.137222473 1chr3_126576592_126579323 262.0103524 89.68964918 434.3310555 4.842599559 2.275781709 0.006785723 1chr5_65538139_65540435 127.1451008 43.56354389 210.7266577 4.837224864 2.274179605 0.04186311 1chr6_42352219_42355010 254.2902016 87.12708778 421.4533154 4.837224864 2.274179605 0.007427478 1chr1_65321565_65323596 176.6616 60.64728659 292.6759134 4.825869876 2.270789016 0.019773703 1chr8_124852171_124853948 129.3282498 44.41773102 214.2387686 4.823271331 2.270011971 0.040704391 1chr12_13527462_13532181 395.1195485 135.8157545 654.4233424 4.818464139 2.268573368 0.002039161 0.636342509chr5_71085990_71087344 81.99489965 28.18817546 135.8016238 4.817680522 2.268338726 0.096786946 1chr6_52950441_52952322 141.4146987 48.6886667 234.1407308 4.808937 2.265718026 0.033783955 1chr7_55152435_55155025 161.2212984 55.52216378 266.9204331 4.807457327 2.265274052 0.025013584 1chr1_64312279_64313572 69.32309892 23.91723978 114.7289581 4.796914657 2.262106773 0.127067699 1chr12_30683973_30685591 89.12969862 30.75073686 147.5086604 4.796914657 2.262106773 0.084774249 1chr2_44553551_44555123 84.17804869 29.04236259 139.3137348 4.796914657 2.262106773 0.093453896 1chr3_12563579_12564768 69.32309892 23.91723978 114.7289581 4.796914657 2.262106773 0.127067699 1chr7_20425852_20428383 183.2110472 63.20984799 303.2122463 4.796914657 2.262106773 0.018463108 1chr8_11637887_11639928 113.8879482 39.29260821 188.4832883 4.796914657 2.262106773 0.053721355 1chr21_47812471_47815284 264.620595 91.39802345 437.8431665 4.790510232 2.260179324 0.006943119 1chr7_28569060_28570921 121.0227472 41.85516962 200.1903248 4.782929483 2.257894521 0.047887186 1chr2_236049286_236051075 106.1677974 36.73004681 175.6055481 4.780978064 2.257305786 0.062121267 1chr13_43208469_43211619 197.4806451 68.3349708 326.6263194 4.779782819 2.256945067 0.015477511 1chr21_44186615_44188679 197.4806451 68.3349708 326.6263194 4.779782819 2.256945067 0.015477511 1chr1_24981041_24982847 91.31284767 31.604924 151.0207713 4.778393751 2.25652574 0.081956719 1chr14_41193406_41195004 91.31284767 31.604924 151.0207713 4.778393751 2.25652574 0.081956719 1chr8_8693066_8696238 273.938543 94.81477199 453.062314 4.778393751 2.25652574 0.006368527 1chr14_33811100_33813360 224.4220438 77.73102929 371.1130582 4.774323222 2.25529624 0.011113532 1chr14_40208662_40209870 66.55459805 23.06305265 110.0461435 4.771534156 2.2544532 0.136139724 1chr8_8860896_8865605 517.5818346 179.3792984 855.7843709 4.770808999 2.254233929 0.000941468 0.436088674chr16_29175835_29178248 184.2234926 64.06403513 304.38295 4.751229756 2.248300973 0.018868408 1chr2_152747464_152749499 132.5238443 46.12610529 218.9215833 4.746153656 2.246758808 0.040481611 1chr3_87788983_87791060 164.4168929 57.23053805 271.6032477 4.745774842 2.246643655 0.02494983 1chr22_27233637_27234849 63.78609717 22.20886551 105.3633288 4.744201309 2.246165229 0.146010487 1chr1_48391658_48394042 240.2894389 83.71033924 396.8685386 4.740973961 2.245183469 0.009523362 1chr2_189463718_189464705 48.93114741 17.0837427 80.77855211 4.728387305 2.241348212 0.20637983 1chr22_31335251_31338126 239.7040871 83.71033924 395.697835 4.726988788 2.240921443 0.009713087 1chr15_58933878_58934766 43.97949748 15.37536843 72.58362653 4.720773155 2.239023159 0.233483657 1chr3_59590698_59593258 219.8974874 76.87684216 362.9181327 4.720773155 2.239023159 0.01230321 1chr4_187660650_187662173 109.9487437 38.43842108 181.4590663 4.720773155 2.239023159 0.059958085 1chr3_65964903_65966236 78.05569512 27.33398832 128.7774019 4.711255467 2.236111564 0.109503465 1chr14_39614431_39615899 141.2564405 49.54285383 232.9700271 4.702394171 2.233395476 0.036269394 1chr6_9243159_9244635 80.23884417 28.18817546 132.2895129 4.693085336 2.230536694 0.105609224 1chr14_78828610_78831028 179.6989362 63.20984799 296.1880244 4.685789221 2.22829206 0.02109912 1chr1_2367991_2370786 237.3626798 83.71033924 391.0150204 4.671048092 2.223746299 0.010510046 1chr7_22213263_22215123 118.6813399 41.85516962 195.5075102 4.671048092 2.223746299 0.053091668 1chr15_39608640_39610160 77.47034329 27.33398832 127.6066983 4.668425872 2.222936175 0.112752754 1chr2_239124065_239125988 132.9509378 46.98029243 218.9215833 4.659859953 2.220286597 0.04241192 1chr21_47299775_47300771 48.34579558 17.0837427 79.60784846 4.659859953 2.220286597 0.213294001 1chr6_73375000_73375961 48.34579558 17.0837427 79.60784846 4.659859953 2.220286597 0.213294001 1chr10_3997539_3999388 164.2586346 58.08472518 270.432544 4.655828932 2.219038051 0.026689102 1chr12_105356133_105357576 91.73994123 32.45911113 151.0207713 4.652646547 2.218051592 0.086170607 1chr1_75463731_75465155 79.65349234 28.18817546 131.1188092 4.651553607 2.217712653 0.108713699 1chr1_81943520_81944905 79.65349234 28.18817546 131.1188092 4.651553607 2.217712653 0.108713699 1chr2_128348403_128349917 79.65349234 28.18817546 131.1188092 4.651553607 2.217712653 0.108713699 1chr9_101600169_101601766 98.8747402 35.02167254 162.7278079 4.646488762 2.216140919 0.075830862 1chr6_89371660_89372901 62.61539352 22.20886551 103.0219215 4.638774614 2.213743751 0.155315961 1chr5_146471316_146475082 293.2703679 104.2108305 482.3299053 4.628404775 2.21051504 0.006141892 1chrX_152710758_152713385 187.2608287 66.62659653 307.8950609 4.621203498 2.208268621 0.020092516 1chr9_117238441_117242191 376.7048065 134.1073802 619.3022328 4.617957877 2.207255014 0.003026622 0.757640236chr12_95948254_95949179 52.71209367 18.79211697 86.63207038 4.610021878 2.204773597 0.195388028 1chr5_29015926_29016966 52.71209367 18.79211697 86.63207038 4.610021878 2.204773597 0.195388028 1chr7_21903823_21910317 696.172963 248.5684563 1143.77747 4.601458635 2.20209126 0.000504196 0.297007319chr11_91691961_91693557 74.11649059 26.47980119 121.75318 4.59796428 2.200995258 0.124080548 1chr8_10678042_10679807 188.858626 67.48078367 310.2364682 4.597404644 2.200819652 0.020065462 1chr18_1559171_1560270 54.89524272 19.64630411 90.14418134 4.58835315 2.197976435 0.187185557 1chr10_13395927_13397332 90.56923758 32.45911113 148.679364 4.580512492 2.195509024 0.091091227 1chr2_2024480_2026448 111.9736345 40.14679535 183.8004736 4.578210342 2.194783748 0.062584575 1chr2_171852093_171854236 133.3780314 47.83447956 218.9215833 4.576648168 2.194291388 0.044391283 1chr7_22171309_22173285 204.7260211 73.46009361 335.9919486 4.573802348 2.193394022 0.016794276 1chr1_73988467_73990389 128.4263815 46.12610529 210.7266577 4.56849015 2.191717445 0.04819174 1chr10_3990368_3991580 64.21319074 23.06305265 105.3633288 4.56849015 2.191717445 0.153843309 1chr12_109570874_109573047 142.6959794 51.2512281 234.1407308 4.56849015 2.191717445 0.038751478 1chr2_67788206_67789542 64.21319074 23.06305265 105.3633288 4.56849015 2.191717445 0.153843309 1chr4_123623290_123624779 71.34798971 25.62561405 117.0703654 4.56849015 2.191717445 0.132740313 1chr7_140950689_140952175 85.61758766 30.75073686 140.4844385 4.56849015 2.191717445 0.100453637 1chr8_123248630_123251464 214.0439691 76.87684216 351.2110961 4.56849015 2.191717445 0.015095493 1chrX_109029035_109030073 49.9435928 17.93792984 81.94925576 4.56849015 2.191717445 0.2108051 1chr10_46982679_46986533 441.7721844 158.8788071 724.6655617 4.561121617 2.189388639 0.002042988 0.636342509chr1_73296214_73297666 87.8007367 31.604924 143.9965494 4.556142879 2.18781299 0.096974867 1chr22_23619297_23622508 334.164848 120.440386 547.88931 4.549049766 2.185565218 0.004649147 0.941730535chr6_39024850_39030591 523.4505675 188.7753568 858.1257782 4.545751058 2.184518679 0.001269219 0.524256232chr12_31174104_31177060 250.7304094 90.54383632 410.9169825 4.538320875 2.182158616 0.010328446 1chr7_68894953_68898249 281.4527543 101.6482691 461.2572396 4.537777611 2.181985907 0.007583829 1chr5_169486750_169491416 503.6439678 181.9418598 825.3460759 4.536317684 2.181521678 0.001441193 0.554625916chr12_105078213_105080015 99.30183377 35.87585967 162.7278079 4.535858077 2.1813755 0.079561626 1chr14_105003021_105004507 99.30183377 35.87585967 162.7278079 4.535858077 2.1813755 0.079561626 1chrX_2429861_2432099 130.0241787 46.98029243 213.068065 4.535264767 2.181186777 0.047985686 1chr7_25353085_25355868 245.7787595 88.83546205 402.7220569 4.533347918 2.180576887 0.010936929 1chr15_101688487_101690012 115.7545808 41.85516962 189.6539919 4.531196353 2.179892009 0.060361024 1chr1_70633331_70635338 148.6600747 53.81378951 243.50636 4.52498072 2.177911645 0.036575043 1chr22_47080393_47082946 243.0102586 87.98127491 398.0392423 4.524135876 2.177642259 0.011366505 1chr16_85009858_85011875 134.3904768 48.6886667 220.0922869 4.52040078 2.176450688 0.045304841 1chr8_8960141_8963944 1598.416124 579.1388776 2617.69337 4.51997521 2.17631486 7.05E-05 0.090970878chr18_54798127_54800316 198.0183157 71.75171934 324.2849121 4.519542041 2.176176594 0.019087894 1chr3_40772772_40774034 87.21538488 31.604924 142.8258458 4.519101067 2.176035822 0.099716732 1chr3_63949684_63951510 127.2556778 46.12610529 208.3852504 4.517729148 2.175597779 0.050641913 1chr14_35618704_35621060 207.3362637 75.16846788 339.5040596 4.516575489 2.175229322 0.017106394 1chr12_104979391_104981308 145.8915739 52.95960237 238.8235454 4.50954189 2.172980882 0.038475152 1chr12_32645629_32647021 89.39853393 32.45911113 146.3379567 4.508378437 2.172608622 0.096275507 1chr10_28939678_28942039 122.3040279 44.41773102 200.1903248 4.506991244 2.172164647 0.055097297 1chr2_160678094_160679641 98.71648195 35.87585967 161.5571042 4.503226005 2.170958884 0.081725795 1chr14_35582275_35585450 265.4271009 96.52314626 434.3310555 4.499760652 2.169848265 0.009238377 1chr1_48149830_48151416 108.03443 39.29260821 176.7762517 4.498969647 2.169594634 0.069838441 1chr8_144249893_144253949 551.6732469 200.7339767 902.612517 4.49656073 2.168821954 0.00117441 0.499188681chr6_47772400_47773755 67.99413701 24.77142692 111.2168471 4.489723078 2.166626464 0.1464358 1chr21_17613641_17614764 51.54139002 18.79211697 84.29066307 4.485426692 2.165245233 0.208297108 1chr7_27923545_27926609 257.7069501 93.96058486 421.4533154 4.485426692 2.165245233 0.010133783 1chr7_52109592_52111280 128.8534751 46.98029243 210.7266577 4.485426692 2.165245233 0.050406644 1chr14_35601957_35605460 473.1904582 172.5458013 773.8351151 4.484809885 2.165046829 0.001862755 0.61933162chr12_110197201_110202150 463.2871584 169.1290527 757.445264 4.478504738 2.163017132 0.001997916 0.636342509chr2_33267307_33268670 70.17728606 25.62561405 114.7289581 4.477120347 2.162571099 0.140815123 1chr10_12030183_12032209 123.9018251 45.27191816 202.5317321 4.47367243 2.161459624 0.054821883 1chr19_16883595_16884705 53.72453907 19.64630411 87.80277403 4.469175146 2.160008585 0.199403142 1chr22_49013695_49014982 63.04248709 23.06305265 103.0219215 4.466968146 2.159295967 0.163469618 1chr7_33620136_33622452 154.0388182 56.37635091 251.7012856 4.464660828 2.158550581 0.035359817 1chr5_59742487_59744087 90.99633115 33.31329827 148.679364 4.463063454 2.158034318 0.095567446 1chrX_131796980_131799903 181.9926623 66.62659653 297.3587281 4.463063454 2.158034318 0.024416366 1chr1_248854777_248855843 46.5897401 17.0837427 76.09573749 4.454277896 2.155191569 0.23531041 1chr12_109541851_109544256 223.6307525 82.00196497 365.25954 4.454277896 2.155191569 0.015007707 1chr2_38931208_38933101 139.7692203 51.2512281 228.2872125 4.454277896 2.155191569 0.043666134 1chr2_120715701_120716816 55.90768812 20.50049124 91.31488499 4.454277896 2.155191569 0.191010085 1chr3_42594205_42595081 46.5897401 17.0837427 76.09573749 4.454277896 2.155191569 0.23531041 1chr9_97495988_97497248 83.86153218 30.75073686 136.9723275 4.454277896 2.155191569 0.109278048 1chr9_117443854_117444907 55.90768812 20.50049124 91.31488499 4.454277896 2.155191569 0.191010085 1chr13_83822174_83823925 104.6805773 38.43842108 170.9227334 4.446663746 2.152723313 0.076003567 1chr20_60679182_60680844 76.7267332 28.18817546 125.265291 4.443894964 2.151824717 0.125555959 1chr1_217768456_217769561 58.09083717 21.35467838 94.82699595 4.440572426 2.150745664 0.183081368 1chr14_78812848_78814492 106.8637263 39.29260821 174.4348444 4.439380645 2.150358415 0.073571851 1chr12_66500673_66501756 48.77288915 17.93792984 79.60784846 4.43796186 2.149897269 0.224864723 1chr8_58547606_58551225 257.5486918 94.81477199 420.2826117 4.432670172 2.148176018 0.010711176 1chr20_5908137_5909358 69.59193423 25.62561405 113.5582544 4.431435445 2.147774097 0.145020767 1chr4_14182199_14183442 60.27398621 22.20886551 98.33910692 4.427921222 2.146629555 0.175583858 1chr18_63433618_63436670 192.3230557 70.89753221 313.7485792 4.42538082 2.14580161 0.022156337 1chr12_66304717_66305831 50.95603819 18.79211697 83.11995942 4.423129099 2.145067351 0.215037181 1chr21_42705981_42707264 71.77508328 26.47980119 117.0703654 4.4211195 2.14441173 0.139480898 1chr1_98096295_98097173 41.63809017 15.37536843 67.90081192 4.416207145 2.142807844 0.267050488 1chr2_134534325_134535811 104.0952254 38.43842108 169.7520298 4.416207145 2.142807844 0.078019065 1chr3_127324381_127327265 270.6475861 99.9398948 441.3552775 4.416207145 2.142807844 0.00957859 1chr5_29112797_29116577 388.4270577 143.5034387 633.3506767 4.413487805 2.141919212 0.003603793 0.856457846chr2_203226247_203227715 94.77727742 35.02167254 154.5328823 4.412492925 2.141593965 0.091634949 1chr8_68139998_68141758 106.2783745 39.29260821 173.2641408 4.409586145 2.14064326 0.075508787 1chr11_1000378_1003039 267.8790853 99.08570767 436.6724629 4.407017653 2.139802676 0.009934476 1chr13_27579481_27581719 150.0996137 55.52216378 244.6770636 4.406835883 2.13974317 0.038927373 1chr1_11368319_11369694 76.14138138 28.18817546 124.0945873 4.402363235 2.138278186 0.129203363 1chr2_206584906_206587237 188.969203 70.04334507 307.8950609 4.395778937 2.136118835 0.023660882 1chr7_131939372_131942811 389.4395031 144.3576258 634.5213803 4.395482239 2.136021456 0.003663457 0.861341436chr20_23213060_23214268 66.82343336 24.77142692 108.8754398 4.395202592 2.135929666 0.155390575 1chr1_120353332_120354660 78.32453043 29.04236259 127.6066983 4.393812585 2.135473334 0.124433252 1chr16_11172139_11173421 78.32453043 29.04236259 127.6066983 4.393812585 2.135473334 0.124433252 1chr3_56973108_56975749 179.651255 66.62659653 292.6759134 4.39277899 2.135133916 0.026617579 1chr2_208279935_208281587 101.3267246 37.58423394 165.0692152 4.391980303 2.134871584 0.082788459 1chr21_42744705_42748428 262.3420835 97.3773334 427.3068336 4.388155012 2.13361449 0.010694661 1chr1_57166955_57168047 57.50548534 21.35467838 93.6562923 4.385750544 2.132823756 0.18880609 1chr19_5101545_5104478 317.079068 117.8778246 516.2803113 4.379791644 2.130862239 0.006557944 1chr18_45764327_45766050 165.381657 61.50147372 269.2618404 4.378136393 2.1303169 0.032337026 1chr13_100574005_100576082 153.88056 57.23053805 250.5305819 4.377568173 2.130129646 0.037733277 1chr18_55056108_55058032 153.88056 57.23053805 250.5305819 4.377568173 2.130129646 0.037733277 1chr18_33777214_33778343 59.68863439 22.20886551 97.16840326 4.375207874 2.129351564 0.181015784 1chr12_104273231_104275102 107.8761717 40.14679535 175.6055481 4.374086314 2.128981689 0.075009453 1chr19_7917356_7919223 133.0615149 49.54285383 216.5801759 4.371572471 2.128152316 0.050887072 1chr18_942328_945513 231.6197386 86.27290064 376.9665765 4.369466816 2.127457246 0.014910015 1chr6_78083035_78084306 73.3728805 27.33398832 119.4117727 4.368618706 2.127177192 0.138139872 1chr4_186421585_186422715 61.87178343 23.06305265 100.6805142 4.365446143 2.126129103 0.173646063 1chr1_174177979_174179377 87.05712662 32.45911113 141.6551421 4.364110327 2.125687574 0.107485905 1chr1_48228806_48230263 87.05712662 32.45911113 141.6551421 4.364110327 2.125687574 0.107485905 1chr14_40982043_40983289 87.05712662 32.45911113 141.6551421 4.364110327 2.125687574 0.107485905 1chr7_111789996_111791993 199.2995964 74.31428075 324.2849121 4.363695764 2.125550521 0.021495618 1chr8_11394442_11396213 137.427813 51.2512281 223.6043979 4.362908093 2.125290083 0.048024401 1chr5_15488830_15490870 176.2974023 65.7724094 286.8223952 4.360831507 2.124603249 0.028487438 1chrX_152785346_152787856 215.1669916 80.2935907 350.0403925 4.359506026 2.124164673 0.018001513 1chr12_109701852_109703280 89.24027567 33.31329827 145.1672531 4.357636758 2.123545942 0.103722514 1chr7_134892053_134893965 153.2952081 57.23053805 249.3598782 4.357112247 2.12337228 0.038604343 1chrX_148881346_148882504 64.05493248 23.91723978 104.1926252 4.356381678 2.123130359 0.16666783 1chr1_89201934_89203995 105.1076708 39.29260821 170.9227334 4.349997143 2.121014453 0.079528471 1chr21_46115022_46118129 171.3457524 64.06403513 278.6274696 4.349202623 2.120750923 0.030614591 1chr1_90228060_90229324 66.23808153 24.77142692 107.7047361 4.347942349 2.120332811 0.160054545 1chr12_55233362_55235221 146.1604092 54.66797664 237.6528417 4.347203908 2.120087768 0.042890859 1chr1_53647825_53649873 120.975066 45.27191816 196.6782138 4.344375539 2.119148819 0.062062894 1chr2_219108642_219110700 148.3435582 55.52216378 241.1649527 4.343579865 2.118884565 0.041718666 1chr8_56624324_56626670 244.133281 91.39802345 396.8685386 4.342200451 2.118426327 0.013445156 1chr19_16170696_16172845 205.2636917 76.87684216 333.6505413 4.340065642 2.117716863 0.020486474 1chr2_160966593_160968062 111.657118 41.85516962 181.4590663 4.335403918 2.116166412 0.072147632 1chr21_45970160_45972359 239.1816311 89.68964918 388.673613 4.333539228 2.115545764 0.014267503 1chr8_8992917_8994268 298.2849137 111.8985147 484.6713127 4.33134715 2.114815806 0.008154781 1chr17_75659557_75660847 56.92013351 21.35467838 92.48558865 4.330928662 2.114676409 0.194693343 1chr3_64205558_64206932 86.47177479 32.45911113 140.4844385 4.3280433 2.113714933 0.110473546 1chr13_114739813_114741700 131.8908112 49.54285383 214.2387686 4.324312228 2.112470694 0.053389741 1chr15_82705934_82707256 59.10328256 22.20886551 95.99769961 4.322494526 2.111864137 0.186600668 1chr4_48677092_48678140 59.10328256 22.20886551 95.99769961 4.322494526 2.111864137 0.186600668 1chr1_53100536_53104147 268.1479206 100.7940819 435.5017592 4.320707633 2.111267612 0.010858943 1chr6_112375085_112376832 104.522319 39.29260821 169.7520298 4.320202642 2.111098984 0.081613418 1chr2_136548497_136551246 224.9120332 84.56452637 365.25954 4.319299778 2.110797449 0.016805518 1chr2_162049757_162052267 120.3897142 45.27191816 195.5075102 4.31851616 2.110535688 0.063616649 1chr3_60336850_60337997 61.28643161 23.06305265 99.50981057 4.314685141 2.109255284 0.178948785 1chr5_141313406_141314627 61.28643161 23.06305265 99.50981057 4.314685141 2.109255284 0.178948785 1chr5_77267553_77269043 93.02122194 35.02167254 151.0207713 4.312208995 2.108427101 0.099316905 1chr21_30303937_30306303 124.7560123 46.98029243 202.5317321 4.310993432 2.108020365 0.059834763 1chr10_36177259_36180779 297.11421 111.8985147 482.3299053 4.310422767 2.107829376 0.008437597 1chr3_16473957_16477445 299.2973591 112.7527018 485.8420163 4.308916846 2.107325257 0.008292751 1chr1_199945830_199947079 63.46958066 23.91723978 103.0219215 4.30743357 2.106828547 0.171706859 1chr12_56830421_56832898 174.5413468 65.7724094 283.3102842 4.30743357 2.106828547 0.030396432 1chr2_222306057_222307515 95.20437098 35.87585967 154.5328823 4.30743357 2.106828547 0.095923044 1chr9_80149108_80150578 79.33697582 29.89654973 128.7774019 4.30743357 2.106828547 0.126811275 1chr14_30739375_30740539 65.6527297 24.77142692 106.5340325 4.300682106 2.104565496 0.164846805 1chr13_31403084_31410716 860.0349333 324.5911113 1395.478755 4.299189678 2.104064763 0.000489353 0.295831887chr18_34079623_34081959 201.3244872 76.02265502 326.6263194 4.296433995 2.103139731 0.022278752 1chr1_50737010_50739137 101.7538181 38.43842108 165.0692152 4.294380741 2.102450107 0.086597683 1chr1_64224049_64225237 67.83587875 25.62561405 110.0461435 4.294380741 2.102450107 0.158342978 1chr6_50154523_50156064 135.6717575 51.2512281 220.0922869 4.294380741 2.102450107 0.051564037 1chr1_84459277_84461572 221.5581805 83.71033924 359.4060217 4.293448396 2.102136852 0.017854092 1chr6_42218862_42221554 377.4636312 142.6492516 612.2780109 4.292192242 2.101714694 0.004572063 0.933260509chr7_21896789_21899445 257.6592689 97.3773334 417.9412044 4.291976272 2.1016421 0.012376115 1chr3_65887699_65889177 70.0190278 26.47980119 113.5582544 4.288485915 2.100468382 0.152171907 1chr5_168586892_168589828 230.2907767 87.12708778 373.4544655 4.286318699 2.099739122 0.016388765 1chr1_117064438_117066080 90.25272106 34.1674854 146.3379567 4.282959515 2.09860804 0.105676903 1chr2_208301541_208303410 144.4043537 54.66797664 234.1407308 4.282959515 2.09860804 0.045976446 1chr8_128073747_128078770 618.0848013 234.047275 1002.122328 4.281709016 2.098186754 0.001210048 0.512327749chr10_43473318_43478167 540.9309745 205.0049124 876.8570367 4.277248903 2.096683163 0.001751604 0.599554696chr22_22035054_22037869 356.0592343 134.9615673 577.1569013 4.276453754 2.096414939 0.005456883 0.996107968chr1_2408567_2415390 975.7418329 369.8630295 1581.620636 4.27623339 2.096340595 0.000372964 0.266432385chr2_128379165_128381868 322.1412949 122.1487603 522.1338296 4.274573301 2.095780414 0.007125638 1chr13_33634047_33635778 132.9032566 50.39704097 215.4094723 4.274248411 2.095670757 0.05437389 1chr8_11246209_11251411 725.2173629 275.0482575 1175.386468 4.273382711 2.095378526 0.000800546 0.398029403chr8_119586629_119588149 96.80216821 36.73004681 156.8742896 4.27100707 2.094576286 0.095152237 1chr1_48099553_48100624 58.51793073 22.20886551 94.82699595 4.269781178 2.094162135 0.192342087 1chr5_80716598_80721005 274.5391095 104.2108305 444.8673884 4.268917025 2.093870121 0.010819121 1chr10_50224385_50227328 179.9200903 68.3349708 291.5052098 4.265827677 2.092825688 0.029428094 1chr11_1039142_1042658 463.1917959 175.9625498 750.4210421 4.264663378 2.09243187 0.00272626 0.723644078chr2_220396221_220400153 445.1412517 169.1290527 721.1534507 4.26392414 2.092181771 0.003040642 0.759382921chr19_16373024_16374508 121.4021596 46.12610529 196.6782138 4.26392414 2.092181771 0.064790159 1chr4_115514149_115516905 202.3369326 76.87684216 327.7970231 4.26392414 2.092181771 0.02267161 1chr8_123757498_123758662 80.93477304 30.75073686 131.1188092 4.26392414 2.092181771 0.125612065 1chr13_46389789_46391572 143.8190019 54.66797664 232.9700271 4.261544718 2.091376471 0.047051587 1chr1_87218976_87225988 927.9813891 352.7792868 1503.183491 4.260974348 2.091183366 0.000435315 0.282542518chr8_8866437_8873506 1136.867769 432.2186903 1841.516847 4.26061364 2.091061231 0.000267379 0.214673844chr8_19455236_19456667 62.88422883 23.91723978 101.8512179 4.258485461 2.090340424 0.176884956 1chr1_88682086_88682999 42.65053557 16.22955557 69.07151557 4.255909245 2.089467386 0.271939902 1chr1_58783340_58784693 67.25052693 25.62561405 108.8754398 4.248695839 2.087020066 0.163028036 1chr18_933325_934786 112.0842115 42.70935675 181.4590663 4.248695839 2.087020066 0.07536095 1chr18_44210928_44212337 67.25052693 25.62561405 108.8754398 4.248695839 2.087020066 0.163028036 1chr5_68066007_68067717 89.66736923 34.1674854 145.1672531 4.248695839 2.087020066 0.108558389 1chr21_45922353_45924801 138.8673519 52.95960237 224.7751015 4.24427472 2.085518041 0.050996568 1chr4_75928813_75931304 208.3010279 79.43940356 337.1626523 4.24427472 2.085518041 0.021592204 1chr4_187631417_187635043 257.5010106 98.23152053 416.7705007 4.242736939 2.08499523 0.013052204 1chr1_65329260_65332003 188.0673347 71.75171934 304.38295 4.242169425 2.084802241 0.027235455 1chr1_73986278_73988041 118.6336587 45.27191816 191.9953992 4.240938026 2.084383401 0.068503165 1chr1_85399294_85402430 241.6336155 92.25221059 391.0150204 4.238543639 2.083568641 0.015305239 1chr7_28753341_28754986 120.8168077 46.12610529 195.5075102 4.238543639 2.083568641 0.066396067 1chr8_19323884_19326412 120.8168077 46.12610529 195.5075102 4.238543639 2.083568641 0.066396067 1chr1_85914448_85916099 147.5999481 56.37635091 238.8235454 4.236236321 2.082783073 0.045505347 1chr3_8775612_8776816 73.79997407 28.18817546 119.4117727 4.236236321 2.082783073 0.144797587 1chr1_93455400_93456960 100.5831145 38.43842108 162.7278079 4.233467539 2.081839827 0.091213522 1chr8_129506186_129507516 100.5831145 38.43842108 162.7278079 4.233467539 2.081839827 0.091213522 1chr11_15391316_15392874 75.98312312 29.04236259 122.9238836 4.232571756 2.081534527 0.139316643 1chr12_109929852_109931402 75.98312312 29.04236259 122.9238836 4.232571756 2.081534527 0.139316643 1chr15_79365407_79368340 232.3156675 88.83546205 375.7958729 4.230246167 2.080741619 0.016955938 1chr1_64156610_64158907 129.5494039 49.54285383 209.555954 4.229791742 2.080586633 0.058752847 1chr5_42677132_42678348 78.16627217 29.89654973 126.4359946 4.229116596 2.080356336 0.13410293 1chr17_74040029_74041628 104.9494126 40.14679535 169.7520298 4.228283436 2.080072089 0.085279303 1chr11_128960588_128961632 53.56628081 20.50049124 86.63207038 4.225853388 2.079242715 0.2162193 1chr14_23351287_23352964 457.4965359 175.1083627 739.8847092 4.225296256 2.079052499 0.00298727 0.75474877chr8_63059733_63061350 109.3157107 41.85516962 176.7762517 4.223522526 2.078446746 0.079831859 1chr12_109995864_109997128 82.53257026 31.604924 133.4602165 4.222766571 2.078188499 0.124413244 1chr12_104991250_104994374 276.5640002 105.9192047 447.2087957 4.222169122 2.077984368 0.011181304 1chr3_58149194_58152891 390.246009 149.4827486 631.0092694 4.221284898 2.077682201 0.004596883 0.936562992chr4_68600388_68601916 111.4988597 42.70935675 180.2883627 4.221284898 2.077682201 0.077275356 1chrX_116037148_116038348 55.74942986 21.35467838 90.14418134 4.221284898 2.077682201 0.206970719 1chr5_170888478_170891701 365.6460176 140.0866901 591.2053451 4.220282059 2.077339423 0.005468122 0.996107968chr6_38636802_38637879 57.93257891 22.20886551 93.6562923 4.217067831 2.076240227 0.198243663 1chr7_27728635_27730446 173.7977367 66.62659653 280.9688769 4.217067831 2.076240227 0.032998258 1chrX_104165630_104167308 115.8651578 44.41773102 187.3125846 4.217067831 2.076240227 0.072469344 1chr18_72178542_72179959 89.08201741 34.1674854 143.9965494 4.214432163 2.075338261 0.111513196 1chr10_118031208_118033869 149.1977454 57.23053805 241.1649527 4.213920765 2.075163187 0.045267893 1chr1_118592195_118593395 62.298877 23.91723978 100.6805142 4.209537352 2.073661683 0.182205332 1chr12_15751490_15753182 93.4483155 35.87585967 151.0207713 4.209537352 2.073661683 0.103862117 1chr1_168302184_168303699 97.8146136 37.58423394 158.0449933 4.205087524 2.072135829 0.096874562 1chr6_24960612_24962608 131.1472011 50.39704097 211.8973613 4.204559578 2.071954688 0.058389407 1chr19_17041119_17042658 133.3303502 51.2512281 215.4094723 4.203010938 2.071423211 0.056673924 1chr5_34755081_34758256 299.9932879 115.3152632 484.6713127 4.203010938 2.071423211 0.009316024 1chr4_16079382_16080592 68.84832415 26.47980119 111.2168471 4.200063525 2.070411148 0.161214653 1chr13_74898025_74900029 104.3640607 40.14679535 168.5813261 4.199122861 2.070088 0.087486631 1chr1_51086242_51087718 71.03147319 27.33398832 114.7289581 4.197300325 2.069461695 0.154928309 1chr14_52961622_52962953 71.03147319 27.33398832 114.7289581 4.197300325 2.069461695 0.154928309 1chr21_30243531_30244893 71.03147319 27.33398832 114.7289581 4.197300325 2.069461695 0.154928309 1chr2_153013101_153015199 144.2460954 55.52216378 232.9700271 4.195982491 2.069008658 0.049000861 1chr2_98652637_98657238 397.2225497 152.8994972 641.5456023 4.195864696 2.068968156 0.004540631 0.932110698chrX_24669578_24672488 217.4607177 83.71033924 351.2110961 4.195552178 2.068860697 0.020466523 1chr11_2801007_2802525 75.39777129 29.04236259 121.75318 4.192261549 2.067728727 0.143286678 1chr6_40522587_40523741 75.39777129 29.04236259 121.75318 4.192261549 2.067728727 0.143286678 1chr20_43021142_43024642 414.1023903 159.7329943 668.4717863 4.184932421 2.065204326 0.004129914 0.901748681chrX_74327653_74328423 42.06518374 16.22955557 67.90081192 4.18377519 2.064805332 0.280951111 1chr12_104775207_104776755 132.7449984 51.2512281 214.2387686 4.180168487 2.063561093 0.058025101 1chr8_70793437_70795409 134.9281474 52.10541524 217.7508796 4.179045088 2.063173324 0.056326568 1chr10_13445668_13447585 139.2944455 53.81378951 224.7751015 4.17690528 2.062434428 0.053116011 1chr6_84173999_84175417 92.86296368 35.87585967 149.8500677 4.17690528 2.062434428 0.1066425 1chr7_77484277_77485713 92.86296368 35.87585967 149.8500677 4.17690528 2.062434428 0.1066425 1chr9_82414117_82415909 92.86296368 35.87585967 149.8500677 4.17690528 2.062434428 0.1066425 1chr4_95600981_95602467 95.04611272 36.73004681 153.3621786 4.175387509 2.061910097 0.102955394 1chr8_8334551_8337967 287.3214872 111.0443276 463.5986469 4.174897152 2.061740658 0.010722067 1chr1_5751513_5754271 152.3933398 58.93891232 245.8477673 4.171230137 2.060472911 0.044790126 1chr18_949566_952041 167.6753831 64.91822226 270.432544 4.165741676 2.058573378 0.037072545 1chr4_149091210_149093005 112.5113051 43.56354389 181.4590663 4.165388078 2.058450914 0.078639452 1chr8_117640986_117645701 569.1059727 220.3802808 917.8316645 4.164763113 2.058234439 0.001834038 0.615533758chr21_17517571_17518747 57.34722708 22.20886551 92.48558865 4.164354483 2.05809288 0.20430907 1chr2_234422350_234423499 59.53037613 23.06305265 95.99769961 4.162402136 2.057416353 0.195755441 1chr1_89123656_89124842 63.89667422 24.77142692 103.0219215 4.158901378 2.056202474 0.18002337 1chr2_139840462_139841583 66.07982327 25.62561405 106.5340325 4.157326036 2.055655895 0.172781695 1chr8_14121273_14122703 66.07982327 25.62561405 106.5340325 4.157326036 2.055655895 0.172781695 1chr5_141834881_141836248 68.26297232 26.47980119 110.0461435 4.15585233 2.055144392 0.165918727 1chr5_15341875_15345152 209.1552151 81.14777783 337.1626523 4.154921568 2.054821244 0.02323243 1chr1_116580856_116582189 72.62927042 28.18817546 117.0703654 4.153172863 2.054213921 0.153230455 1chr15_64421921_64428177 763.406238 296.4029359 1230.40954 4.151138168 2.053506952 0.000878445 0.421299696chr3_72278208_72282216 323.2643174 125.5655089 520.9631259 4.148934932 2.052741031 0.008227092 1chr5_31806888_31809991 246.2687489 95.66895913 396.8685386 4.148352216 2.052538392 0.016042301 1chr7_27864405_27866639 164.9068823 64.06403513 265.7497294 4.148189056 2.052481647 0.038899375 1chr2_110477407_110480858 298.664326 116.1694504 481.1592017 4.14187379 2.050283593 0.01011786 1chr2_206813005_206814496 105.3765061 41.00098248 169.7520298 4.140194198 2.04969844 0.08901584 1chr10_35296176_35297776 111.9259533 43.56354389 180.2883627 4.138514606 2.049113049 0.080616105 1chr7_140907788_140909472 118.4754004 46.12610529 190.8246956 4.137021636 2.048592503 0.073206046 1chr14_99654355_99656742 205.8013624 80.2935907 331.309134 4.126221422 2.044821242 0.024735374 1chr2_96362797_96365609 216.7171076 84.56452637 348.8696888 4.125485044 2.044563751 0.022052521 1chr1_53966064_53967267 58.9450243 23.06305265 94.82699595 4.111641135 2.039714351 0.201668677 1chr1_75437091_75438789 96.05855812 37.58423394 154.5328823 4.111641135 2.039714351 0.104734455 1chr14_77009740_77010967 80.77651478 31.604924 129.9481056 4.111641135 2.039714351 0.135061225 1chr2_157085763_157087107 67.67762049 26.47980119 108.8754398 4.111641135 2.039714351 0.170748287 1chr2_228284777_228286163 65.49447144 25.62561405 105.3633288 4.111641135 2.039714351 0.177856127 1chr3_65863335_65865283 146.2709862 57.23053805 235.3114344 4.111641135 2.039714351 0.050690811 1chr3_16566071_16567292 72.04391859 28.18817546 115.8996617 4.111641135 2.039714351 0.157614339 1chr3_66853149_66854416 78.59336573 30.75073686 126.4359946 4.111641135 2.039714351 0.140283336 1chr3_140746455_140747971 65.49447144 25.62561405 105.3633288 4.111641135 2.039714351 0.177856127 1chr6_113627349_113628403 45.84613001 17.93792984 73.75433019 4.111641135 2.039714351 0.263771831 1chr8_74437237_74438360 69.86076954 27.33398832 112.3875508 4.111641135 2.039714351 0.164009093 1chr21_33735079_33742418 872.5160092 342.5290412 1402.502977 4.094552019 2.033705617 0.000705024 0.367388428chr1_24752769_24755010 169.7002739 66.62659653 272.7739513 4.094070019 2.033535776 0.038322073 1chr10_28489700_28492421 156.6013796 61.50147372 251.7012856 4.092605759 2.033019699 0.045048169 1chr12_3196261_3198230 152.2350815 59.79309945 244.6770636 4.092061891 2.032827966 0.047622418 1chr1_116815109_116817001 128.220442 50.39704097 206.0438431 4.088411524 2.03154042 0.065717979 1chr7_141135322_141136903 123.8541439 48.6886667 199.0196211 4.08759645 2.031252772 0.069907302 1chr8_8760876_8762674 108.5721006 42.70935675 174.4348444 4.084230194 2.030064181 0.087557184 1chr1_84652026_84653704 106.3889515 41.85516962 170.9227334 4.083670787 2.029866565 0.090527063 1chr1_168036925_168038622 104.2058025 41.00098248 167.4106225 4.083088071 2.029660687 0.093627444 1chr8_101861933_101864829 199.6790088 78.58521643 320.7728011 4.081846634 2.029221977 0.027525251 1chr12_94211799_94213187 97.65635534 38.43842108 156.8742896 4.081184534 2.028987945 0.10378556 1chr9_117417439_117419075 97.65635534 38.43842108 156.8742896 4.081184534 2.028987945 0.10378556 1chr13_84765172_84766804 91.1069082 35.87585967 146.3379567 4.079009062 2.028218712 0.115406846 1chr2_45365013_45366524 91.1069082 35.87585967 146.3379567 4.079009062 2.028218712 0.115406846 1chr8_36347074_36348459 86.7406101 34.1674854 139.3137348 4.077377459 2.027641519 0.124098955 1chr4_189153172_189154606 80.19116296 31.604924 128.7774019 4.074599323 2.026658198 0.138794141 1chr12_12238107_12241647 379.1243243 149.4827486 608.7659 4.072482648 2.025908552 0.006063586 1chr4_39296766_39298037 75.82486486 29.89654973 121.75318 4.072482648 2.025908552 0.149864148 1chr2_203220424_203221597 71.45856676 28.18817546 114.7289581 4.070109406 2.025067575 0.162113196 1chr3_169240494_169241746 69.27541771 27.33398832 111.2168471 4.06881154 2.024607459 0.168730265 1chr5_31687374_31689557 274.9185218 108.4817662 441.3552775 4.068474299 2.024487877 0.013467129 1chr2_217035029_217036292 67.09226867 26.47980119 107.7047361 4.06742994 2.024117496 0.175706145 1chr7_68359067_68360468 67.09226867 26.47980119 107.7047361 4.06742994 2.024117496 0.175706145 1chr5_138656514_138659933 199.093657 78.58521643 319.6020975 4.066949383 2.023947035 0.028079335 1chr17_75719085_75720178 64.90911962 25.62561405 104.1926252 4.065956233 2.023594686 0.183066252 1chr6_101576907_101581460 317.9961509 125.5655089 510.426793 4.065023888 2.02326383 0.009502877 1chr13_31782814_31784752 123.2687921 48.6886667 197.8489175 4.063551765 2.022741273 0.071594117 1chr5_179639053_179640999 244.3544351 96.52314626 392.185724 4.063126195 2.022590173 0.01785453 1chr1_87282676_87283673 60.54282152 23.91723978 97.16840326 4.062693026 2.02243636 0.199052909 1chr12_9039472_9040632 60.54282152 23.91723978 97.16840326 4.062693026 2.02243636 0.199052909 1chr5_90742498_90743695 60.54282152 23.91723978 97.16840326 4.062693026 2.02243636 0.199052909 1chr8_37360050_37363683 363.2569291 143.5034387 583.0104196 4.062693026 2.02243636 0.006844322 1chr12_105458101_105459266 58.35967247 23.06305265 93.6562923 4.060880133 2.021792443 0.207743291 1chr1_116261699_116264354 172.8958684 68.3349708 277.4567659 4.060245621 2.021567004 0.037935873 1chr6_125592552_125596796 453.7784855 179.3792984 728.1776726 4.059429819 2.021277103 0.003905158 0.89336316chr15_51775391_51776577 56.17652343 22.20886551 90.14418134 4.058927787 2.021098673 0.216946283 1chr2_206829530_206831815 166.3464212 65.7724094 266.9204331 4.058243198 2.020855324 0.041070103 1chr19_16363772_16365345 110.1698978 43.56354389 176.7762517 4.057894192 2.020731248 0.086831472 1chr19_5138440_5145597 1055.852848 417.697509 1694.008187 4.055586041 2.019910402 0.00049034 0.295831887chr12_87363932_87365055 51.81022533 20.50049124 83.11995942 4.054535008 2.019536469 0.237056825 1chr8_127522263_127523197 51.81022533 20.50049124 83.11995942 4.054535008 2.019536469 0.237056825 1chr12_3297024_3299377 200.6914542 79.43940356 321.9435048 4.052692875 2.018880848 0.027963945 1chr6_41578449_41579351 250.3185305 99.08570767 401.5513532 4.052565831 2.018835621 0.017077565 1chr12_123880162_123882384 198.5083051 78.58521643 318.4313938 4.052052133 2.018652736 0.028644258 1chr5_19479412_19480370 49.62707628 19.64630411 79.60784846 4.052052133 2.018652736 0.248059243 1chr7_110813943_110814979 49.62707628 19.64630411 79.60784846 4.052052133 2.018652736 0.248059243 1chr20_23836035_23838643 345.2063849 136.6699416 553.7428282 4.051679702 2.018520129 0.007887813 1chr14_61918020_61919704 88.33840732 35.02167254 141.6551421 4.044785181 2.016063083 0.122854372 1chr14_89228752_89230411 88.33840732 35.02167254 141.6551421 4.044785181 2.016063083 0.122854372 1chr12_95896881_95898970 129.2328874 51.2512281 207.2145467 4.043113782 2.015466805 0.066800439 1chr8_56628687_56630449 170.1273675 67.48078367 272.7739513 4.042246348 2.015157247 0.039778745 1chr22_43272862_43280124 876.2492742 347.654164 1404.844385 4.040924948 2.014685557 0.000776943 0.391683987chr1_86881282_86883185 165.7610694 65.7724094 265.7497294 4.040443886 2.014513797 0.041957606 1chr1_61005221_61008603 206.6555495 82.00196497 331.309134 4.040258476 2.014447593 0.026558204 1chr4_7290630_7292274 120.5002912 47.83447956 193.1661029 4.038218972 2.013719143 0.075672185 1chr2_208355590_208358103 197.9229533 78.58521643 317.2606902 4.037154882 2.013338936 0.029220222 1chr8_19971680_19973177 75.23951303 29.89654973 120.5824763 4.033324161 2.011969361 0.154062844 1chr11_16515671_16517577 111.767695 44.41773102 179.117659 4.032571113 2.011699975 0.086108791 1chr8_27778884_27780412 111.767695 44.41773102 179.117659 4.032571113 2.011699975 0.086108791 1chr12_59404366_59405456 70.87321494 28.18817546 113.5582544 4.028577677 2.010270573 0.166729578 1chr2_3611491_3613433 105.2182479 41.85516962 168.5813261 4.027730091 2.009967008 0.095171861 1chr11_119609544_119611661 135.1969827 53.81378951 216.5801759 4.024622275 2.008853387 0.062423287 1chr13_31383577_31385240 100.8519498 40.14679535 161.5571042 4.024159408 2.008687456 0.101901581 1chr7_26546337_26549532 334.7177332 133.2531931 536.1822734 4.023785555 2.00855342 0.008823213 1chr2_66699100_66700322 66.50691684 26.47980119 106.5340325 4.023218745 2.00835018 0.180795153 1chr19_2184887_2191145 636.6882308 253.6935791 1019.682882 4.019348404 2.006961638 0.001763761 0.601170731chr1_47732291_47733860 94.30250264 37.58423394 151.0207713 4.018194745 2.006547487 0.113188217 1chr20_57387343_57395713 1725.240633 687.6206437 2762.860623 4.018001275 2.006478022 0.000198516 0.185674834chr13_91348509_91349740 62.14061874 24.77142692 99.50981057 4.017120649 2.006161791 0.196464507 1chr1_71150576_71151959 92.11935359 36.73004681 147.5086604 4.016021573 2.005767019 0.117305597 1chr1_214449459_214451149 122.0980884 48.6886667 195.5075102 4.015462395 2.005566129 0.075084276 1chr3_45955574_45957086 89.93620454 35.87585967 143.9965494 4.013744917 2.004948933 0.121618073 1chr8_8741943_8743463 387.540404 154.6078714 620.4729365 4.013204054 2.004754513 0.006224876 1chr12_90353019_90355064 147.7105252 58.93891232 236.4821381 4.012326131 2.004438876 0.053544993 1chr5_57071796_57075458 235.4635807 93.96058486 376.9665765 4.011964986 2.004309015 0.020482684 1chr20_23374188_23375512 87.75305549 35.02167254 140.4844385 4.011357205 2.004090441 0.126137312 1chr7_51156016_51159199 321.0334871 128.1280703 513.938904 4.011134351 2.004010289 0.009926211 1chr2_152803399_152805577 115.5486413 46.12610529 184.9711773 4.010119131 2.003645096 0.082651638 1chr3_34436352_34438060 115.5486413 46.12610529 184.9711773 4.010119131 2.003645096 0.082651638 1chr6_160416980_160418791 115.5486413 46.12610529 184.9711773 4.010119131 2.003645096 0.082651638 1chr7_134863257_134865109 115.5486413 46.12610529 184.9711773 4.010119131 2.003645096 0.082651638 1chr8_11201536_11207164 562.4611631 224.6512165 900.2711097 4.007417025 2.002672649 0.002456919 0.693873364chr10_112281615_112282895 83.3867574 33.31329827 133.4602165 4.006214439 2.002239646 0.135847204 1chr3_66800822_66804413 305.7514438 122.1487603 489.3541273 4.006214439 2.002239646 0.01123306 1chr7_67106231_67107718 111.1823432 44.41773102 177.9469554 4.006214439 2.002239646 0.088244642 1chr9_126466223_126467256 55.5911716 22.20886551 88.97347769 4.006214439 2.002239646 0.223525655 1chrX_149252649_149253735 55.5911716 22.20886551 88.97347769 4.006214439 2.002239646 0.223525655 1chr1_247693821_247694813 53.40802255 21.35467838 85.46136672 4.001997371 2.00072022 0.233646512 1chr11_88046522_88047564 53.40802255 21.35467838 85.46136672 4.001997371 2.00072022 0.233646512 1chr3_59670757_59671742 53.40802255 21.35467838 85.46136672 4.001997371 2.00072022 0.233646512 1chr4_149813804_149814789 53.40802255 21.35467838 85.46136672 4.001997371 2.00072022 0.233646512 1chr3_72612353_72614240 132.4284819 52.95960237 211.8973613 4.001113147 2.000401427 0.065835664 1chr1_59339244_59342259 288.2862514 115.3152632 461.2572396 3.999966931 1.999988073 0.013017758 1chr10_32467666_32471494 409.798988 164.0039299 655.5940461 3.997428881 1.999072367 0.005536379 0.996814992chr8_63035633_63038891 352.0246674 140.9408773 563.1084575 3.995352295 1.99832272 0.008090092 1chr9_116691780_116693595 100.266598 40.14679535 160.3864006 3.994998833 1.998195082 0.104510688 1chr1_196692298_196693292 49.04172445 19.64630411 78.4371448 3.992463131 1.997279085 0.255817352 1chr11_74147198_74150880 292.0671977 117.0236375 467.1107578 3.991593218 1.996964704 0.012748174 1chr14_24960421_24961602 72.47101216 29.04236259 115.8996617 3.990710513 1.996645629 0.164747851 1chr8_121062689_121064698 144.9420243 58.08472518 231.7993234 3.990710513 1.996645629 0.056354814 1chr3_66580381_66582001 95.90029986 38.43842108 153.3621786 3.989814731 1.996321756 0.112089752 1chr1_18906196_18908147 119.3295876 47.83447956 190.8246956 3.989270863 1.996125083 0.079378431 1chr2_192102595_192104820 119.3295876 47.83447956 190.8246956 3.989270863 1.996125083 0.079378431 1chr12_3458524_3460116 93.71715081 37.58423394 149.8500677 3.987045949 1.995320232 0.116144854 1chr8_67507879_67509347 70.28786311 28.18817546 112.3875508 3.987045949 1.995320232 0.171466072 1chrX_153045078_153048937 513.2611804 205.8590995 820.6632613 3.986528956 1.995133148 0.003200546 0.794420405chr5_33235810_33237550 91.53400176 36.73004681 146.3379567 3.984148386 1.99427138 0.120390837 1chr20_36514891_36516179 68.10471406 27.33398832 108.8754398 3.983152349 1.993910662 0.178547653 1chr10_118934394_118935693 89.35085272 35.87585967 142.8258458 3.981112845 1.993171765 0.124839019 1chr3_114864873_114865791 44.67542636 17.93792984 71.41292288 3.981112845 1.993171765 0.280943265 1chrX_67807947_67809610 110.5969914 44.41773102 176.7762517 3.979857765 1.992716872 0.090430335 1chr5_133129745_133131532 131.84313 52.95960237 210.7266577 3.97900755 1.992408636 0.067372098 1chr2_151634668_151636113 87.16770367 35.02167254 139.3137348 3.977929228 1.992017609 0.12950156 1chr5_118695437_118697990 174.3354073 70.04334507 278.6274696 3.977929228 1.992017609 0.039969793 1chr2_9584334_9585498 63.73841596 25.62561405 101.8512179 3.97458643 1.990804751 0.193905686 1chr4_84187874_84189365 84.98455462 34.1674854 135.8016238 3.97458643 1.990804751 0.134391533 1chr5_35598438_35599890 84.98455462 34.1674854 135.8016238 3.97458643 1.990804751 0.134391533 1chr2_161036781_161038806 208.0950885 83.71033924 332.4798377 3.971789395 1.989789126 0.027919492 1chr17_78249474_78254650 558.3637003 224.6512165 892.0761842 3.970938586 1.989480049 0.002660576 0.719295323chr13_37388136_37389755 101.8643952 41.00098248 162.7278079 3.968875818 1.988730422 0.103532044 1chr12_109506206_109508032 142.1735234 57.23053805 227.1165088 3.968449652 1.988575502 0.059338444 1chr2_164610772_164617035 424.3374213 170.837427 677.8374155 3.967733695 1.988315199 0.005299671 0.9889824chr15_48028114_48031662 201.5456413 81.14777783 321.9435048 3.967373025 1.988184051 0.029995088 1chr7_121717366_121719069 99.68124613 40.14679535 159.2156969 3.965838258 1.98762584 0.107182223 1chr1_236481908_236485007 218.4254819 87.98127491 348.8696888 3.965272033 1.987419844 0.025327681 1chr1_47296065_47297186 59.37211787 23.91723978 94.82699595 3.964796808 1.987246931 0.211056858 1chr10_43464564_43467242 197.1793432 79.43940356 314.9192829 3.964270485 1.987055402 0.031489755 1chr6_42522584_42526167 351.8664091 141.7950644 561.9377538 3.9630276 1.986603015 0.008452794 1chr3_64198974_64200525 97.49809708 39.29260821 155.7035859 3.96266863 1.98647233 0.110999343 1chr8_102817134_102818722 97.49809708 39.29260821 155.7035859 3.96266863 1.98647233 0.110999343 1chr5_175286207_175290738 574.0728372 231.4847136 916.6609609 3.95992006 1.985471306 0.00253038 0.705052251chr1_101022135_101023262 57.18896882 23.06305265 91.31488499 3.95935813 1.985266567 0.220391043 1chr14_103443411_103445153 133.4409272 53.81378951 213.068065 3.95935813 1.985266567 0.066871248 1chr4_141516046_141516797 38.12597921 15.37536843 60.87659 3.95935813 1.985266567 0.325445429 1chr3_66675658_66677329 112.1947886 45.27191816 179.117659 3.956484866 1.984219239 0.089642469 1chr18_46427623_46429442 186.263598 75.16846788 297.3587281 3.955897152 1.984004919 0.035667257 1chr5_77290327_77291995 93.13179898 37.58423394 148.679364 3.955897152 1.984004919 0.11917334 1chr9_80157445_80158910 93.13179898 37.58423394 148.679364 3.955897152 1.984004919 0.11917334 1chr8_99372944_99375395 241.2694177 97.3773334 385.1615021 3.955350682 1.98380561 0.020578191 1chr14_87650417_87653244 145.9544697 58.93891232 232.9700271 3.95273713 1.982852014 0.057233029 1chr12_3434520_3439090 636.6405496 257.1103277 1016.170771 3.952275199 1.982683406 0.001988087 0.635654193chr20_62072279_62073697 90.94864994 36.73004681 145.1672531 3.952275199 1.982683406 0.123551443 1chr2_202848823_202850579 107.8284905 43.56354389 172.0934371 3.950400306 1.981998853 0.095823226 1chr6_55666558_55667607 71.88566033 29.04236259 114.7289581 3.950400306 1.981998853 0.169379189 1chr8_74393897_74396723 177.5310018 71.75171934 283.3102842 3.948480772 1.981297665 0.039532363 1chrX_4722699_4726069 322.9001197 130.6906317 515.1096077 3.941442482 1.978723721 0.010685123 1chr5_31682428_31686180 320.7169706 129.8364445 511.5974967 3.940322754 1.978313807 0.010873998 1chrY_2379871_2382151 132.8555754 53.81378951 211.8973613 3.937603415 1.977317814 0.068419762 1chr1_93282195_93283932 115.9757349 46.98029243 184.9711773 3.937207874 1.977172885 0.085999561 1chr12_95817541_95818951 82.21605374 33.31329827 131.1188092 3.935929975 1.976704554 0.143290559 1chr3_9029122_9031514 147.5522669 59.79309945 235.3114344 3.935427943 1.976520525 0.056841094 1chr20_18368361_18369378 48.45637263 19.64630411 77.26644115 3.932874129 1.975584014 0.263790854 1chr5_149940613_149942712 143.1859688 58.08472518 228.2872125 3.930245202 1.974619323 0.060253224 1chr15_79358400_79360856 126.3061283 51.2512281 201.3610284 3.928901529 1.97412601 0.075063693 1chr6_143118887_143120091 63.15306414 25.62561405 100.6805142 3.928901529 1.97412601 0.199541147 1chr14_101302635_101305501 218.8525754 88.83546205 348.8696888 3.927144417 1.973480653 0.02619275 1chr15_101666695_101669572 187.2760434 76.02265502 298.5294317 3.926848275 1.973371856 0.036203848 1chr7_18358841_18360989 124.1229792 50.39704097 197.8489175 3.925804247 1.972988237 0.077460149 1chr22_32533577_32534613 46.27322358 18.79211697 73.75433019 3.924748356 1.972600155 0.276421984 1chr4_186675672_186677298 92.54644716 37.58423394 147.5086604 3.924748356 1.972600155 0.122275201 1chr6_79553474_79555485 138.8196707 56.37635091 221.2629906 3.924748356 1.972600155 0.063930969 1chrX_106161017_106162423 92.54644716 37.58423394 147.5086604 3.924748356 1.972600155 0.122275201 1chr11_60647791_60649407 153.5163622 62.35566086 244.6770636 3.923894964 1.972286424 0.053315725 1chr8_11319735_11325312 2529.745254 1027.587123 4031.903384 3.923660867 1.972200351 0.000138688 0.145883627chr4_111331699_111334141 182.9097453 74.31428075 291.5052098 3.922600163 1.971810287 0.038101005 1chr7_73306917_73308653 174.1771491 70.89753221 277.4567659 3.913489755 1.968455668 0.042293018 1chr17_74824829_74828522 245.4774576 99.9398948 391.0150204 3.912501821 1.968091423 0.020730396 1chr17_70422492_70424974 243.2943085 99.08570767 387.5029094 3.910785102 1.967458262 0.021180648 1chr2_207645221_207648206 211.7177765 86.27290064 337.1626523 3.908094544 1.966465369 0.028627356 1chr8_10707511_10708990 83.81385097 34.1674854 133.4602165 3.906059078 1.96571377 0.141686538 1chr10_65370214_65371382 69.11715945 28.18817546 110.0461435 3.903982492 1.964946583 0.181309926 1chr6_106200205_106202328 150.7478614 61.50147372 239.994249 3.902252003 1.964306949 0.056062916 1chr5_150674550_150678748 349.3667435 142.6492516 556.0842355 3.898262553 1.962831261 0.009372008 1chr20_32677663_32679097 66.93401041 27.33398832 106.5340325 3.897493159 1.962546491 0.188883505 1chr2_203613561_203617493 328.1206049 134.1073802 522.1338296 3.893401159 1.961031001 0.010938418 1chr14_40189844_40190958 52.2373189 21.35467838 83.11995942 3.892353608 1.96064278 0.248109139 1chr6_145087148_145088211 52.2373189 21.35467838 83.11995942 3.892353608 1.96064278 0.248109139 1chr7_19558781_19559710 52.2373189 21.35467838 83.11995942 3.892353608 1.96064278 0.248109139 1chr18_77192753_77195513 336.2678493 137.5241287 535.0115698 3.890310556 1.959885327 0.010366902 1chr1_50794477_50795795 77.26440382 31.604924 122.9238836 3.889390263 1.959544002 0.158928818 1chr3_8565495_8568138 167.0423501 68.3349708 265.7497294 3.88892724 1.959372243 0.046799 1chr1_66745889_66748603 217.09652 88.83546205 345.3575779 3.887609406 1.958883277 0.027712264 1chrX_149476099_149477893 152.3456586 62.35566086 242.3356563 3.88634573 1.958414249 0.055676884 1chr7_151306218_151308119 189.886286 77.73102929 302.0415427 3.885726787 1.958184466 0.036542477 1chr1_28115130_28116968 100.1083397 41.00098248 159.2156969 3.883216627 1.957252191 0.111595582 1chr1_62050701_62052046 75.08125477 30.75073686 119.4117727 3.883216627 1.957252191 0.165277696 1chr18_34017971_34019175 62.56771231 25.62561405 99.50981057 3.883216627 1.957252191 0.205324602 1chr6_91387604_91389022 87.59479723 35.87585967 139.3137348 3.883216627 1.957252191 0.13498322 1chr8_9571095_9572109 50.05416985 20.50049124 79.60784846 3.883216627 1.957252191 0.259676025 1chr8_11528357_11529812 75.08125477 30.75073686 119.4117727 3.883216627 1.957252191 0.165277696 1chrX_131892138_131893443 75.08125477 30.75073686 119.4117727 3.883216627 1.957252191 0.165277696 1chr11_118168406_118169968 122.9522756 50.39704097 195.5075102 3.879345025 1.955813094 0.081164187 1chr4_26239393_26241599 183.3368388 75.16846788 291.5052098 3.878025161 1.955322164 0.039451744 1chr15_64414204_64415504 72.89810572 29.89654973 115.8996617 3.876690213 1.954825454 0.171960829 1chr2_171163088_171164920 120.7691265 49.54285383 191.9953992 3.87533992 1.95432286 0.083800246 1chr19_403015_404028 47.8710208 19.64630411 76.09573749 3.873285127 1.953557707 0.27198437 1chr6_89706511_89708004 95.7420416 39.29260821 152.191475 3.873285127 1.953557707 0.119758764 1chr7_18204136_18205901 131.0995199 53.81378951 208.3852504 3.87233927 1.953205358 0.073272288 1chr10_12576085_12579615 345.427539 141.7950644 549.0600136 3.872208217 1.953156532 0.009966443 1chr1_56991071_56995525 422.106591 173.3999884 670.8131936 3.868588457 1.951807262 0.006215109 1chr2_121431973_121433917 128.9163709 52.95960237 204.8731394 3.868479562 1.951766652 0.075579042 1chr2_233296895_233299399 232.8056569 95.66895913 369.9423546 3.866900591 1.951177677 0.024408374 1chr21_46056252_46058354 197.4481785 81.14777783 313.7485792 3.866385348 1.950985433 0.034392814 1chr8_11508019_11511330 359.5388787 147.7743744 571.303383 3.86605178 1.95086096 0.009144494 1chr5_150691151_150692753 81.04535009 33.31329827 128.7774019 3.865645511 1.950709345 0.15110873 1chr18_18771184_18772623 103.889286 42.70935675 165.0692152 3.864942667 1.950447013 0.106727678 1chr2_202866257_202869399 207.7785719 85.41871351 330.1384304 3.864942667 1.950447013 0.031041939 1chr9_126387456_126388979 137.0636153 56.37635091 217.7508796 3.862450763 1.949516542 0.068386636 1chr1_85237881_85242990 658.1078423 270.7773218 1045.438363 3.860878584 1.948929185 0.002167061 0.65427745chr1_7426431_7428223 101.7061369 41.85516962 161.5571042 3.859908004 1.948566463 0.11048392 1chr2_241911933_241913466 101.7061369 41.85516962 161.5571042 3.859908004 1.948566463 0.11048392 1chr3_61119942_61121255 78.86220104 32.45911113 125.265291 3.859171942 1.948291323 0.157034262 1chr8_120277894_120280125 157.7244021 64.91822226 250.5305819 3.859171942 1.948291323 0.053383605 1chr8_11301854_11303507 437.8157279 180.2334855 695.3979703 3.858317273 1.947971783 0.005781302 1chr5_59928512_59929693 56.01826517 23.06305265 88.97347769 3.857836126 1.947791862 0.233727873 1chr6_12163820_12165003 56.01826517 23.06305265 88.97347769 3.857836126 1.947791862 0.233727873 1chr21_32856166_32857562 89.19259446 36.73004681 141.6551421 3.856655638 1.947350333 0.133501644 1chr22_44807074_44808576 99.52298787 41.00098248 158.0449933 3.854663564 1.946604947 0.114411249 1chr2_236671123_236672751 87.00944541 35.87585967 138.1430311 3.850584555 1.945077477 0.138530789 1chr3_33187420_33188831 87.00944541 35.87585967 138.1430311 3.850584555 1.945077477 0.138530789 1chr5_149564551_149565451 43.5047227 17.93792984 69.07151557 3.850584555 1.945077477 0.299095426 1chr11_68967284_68968812 107.6702322 44.41773102 170.9227334 3.848074395 1.944136691 0.102143521 1chr11_126115811_126116793 53.83511612 22.20886551 85.46136672 3.848074395 1.944136691 0.244353072 1chr8_121176777_121181435 579.6727348 239.1723978 920.1730719 3.847321348 1.943854336 0.003001301 0.756530385chr2_122426945_122428087 64.16550953 26.47980119 101.8512179 3.846373965 1.943499036 0.202521047 1chr8_11421383_11422220 223.4877088 92.25221059 354.7232071 3.845145876 1.943038332 0.02721624 1chr8_121997308_121998561 74.49590295 30.75073686 118.241069 3.845145876 1.943038332 0.169820501 1chr8_19072598_19074616 159.3221993 65.7724094 252.8719892 3.844651451 1.942852812 0.053020983 1chr12_29935950_29937780 95.15668977 39.29260821 151.0207713 3.843490626 1.94241715 0.122818783 1chr1_3281355_3284237 241.9653466 99.9398948 383.9907984 3.842217357 1.941939135 0.023068839 1chr17_5374653_5377038 136.4782634 56.37635091 216.5801759 3.841684899 1.941739192 0.069936371 1chr3_66664311_66667764 270.7733778 111.8985147 429.6482409 3.839624164 1.940965102 0.018106094 1chr12_95910221_95911659 72.3127539 29.89654973 114.7289581 3.837531726 1.940178678 0.176723145 1chr22_36944134_36946027 196.2774749 81.14777783 311.4071719 3.837531726 1.940178678 0.035760061 1chr5_54920325_54921897 103.3039341 42.70935675 163.8985115 3.837531726 1.940178678 0.109383197 1chr9_71424075_71426202 144.6255078 59.79309945 229.4579161 3.837531726 1.940178678 0.063444591 1chr22_45039843_45045572 605.1269133 250.2768306 959.9769961 3.835660672 1.939475096 0.002766318 0.726001089chr7_131719297_131722466 204.4247192 84.56452637 324.2849121 3.834762944 1.939137396 0.033040022 1chr3_48573914_48577956 377.8582582 156.3162457 599.4002707 3.834535995 1.939052012 0.008496432 1chr20_19079184_19081501 183.7639324 76.02265502 291.5052098 3.834451845 1.939020352 0.040830786 1chr1_168130199_168131825 171.2503899 70.89753221 271.6032477 3.83092668 1.937693414 0.046881755 1chr5_14108007_14109338 70.12960485 29.04236259 111.2168471 3.829469684 1.937144618 0.183996437 1chr8_102854219_102855975 129.9288163 53.81378951 206.0438431 3.82882984 1.936903546 0.076686764 1chr6_150912923_150914317 59.79921144 24.77142692 94.82699595 3.828079677 1.936620858 0.220502631 1chr7_69385367_69388050 179.3976343 74.31428075 284.4809879 3.828079677 1.936620858 0.043016617 1chr14_51407278_51408912 109.2680295 45.27191816 173.2641408 3.827187974 1.936284762 0.101163072 1chr2_147797349_147798826 78.27684922 32.45911113 124.0945873 3.823104915 1.934744792 0.161278023 1chr11_128876218_128878820 224.5001542 93.10639772 355.8939107 3.822443134 1.934495039 0.02759512 1chr7_6064823_6067751 360.3930658 149.4827486 571.303383 3.821868331 1.934278076 0.009659989 1chr3_14399347_14401060 135.8929116 56.37635091 215.4094723 3.820919034 1.933919687 0.071519424 1chr7_17048653_17051212 240.794643 99.9398948 381.6493911 3.818789202 1.933115285 0.023904162 1chr1_68010576_68011927 86.42409358 35.87585967 136.9723275 3.817952482 1.932799147 0.142164363 1chr14_90811097_90814675 403.3124367 167.4206785 639.2041949 3.817952482 1.932799147 0.007463094 1chr5_74268533_74269732 57.61606239 23.91723978 91.31488499 3.817952482 1.932799147 0.230289997 1chr5_6648762_6650066 86.42409358 35.87585967 136.9723275 3.817952482 1.932799147 0.142164363 1chr8_119360248_119364208 516.3614124 214.4009709 818.321854 3.816782408 1.932356942 0.004163045 0.902456599chr2_176971505_176972365 47.28566897 19.64630411 74.92503384 3.813696125 1.931189894 0.280402556 1chr5_11556664_11558184 94.57133795 39.29260821 149.8500677 3.813696125 1.931189894 0.125951347 1chr5_65504208_65506459 174.4459844 72.60590648 276.2860623 3.80528356 1.928003964 0.046286373 1chrX_74007215_74008816 82.05779548 34.1674854 129.9481056 3.80326805 1.927239622 0.153316612 1chr1_76046857_76048717 145.6379532 60.64728659 230.6286198 3.802785463 1.92705655 0.064354654 1chr8_11653841_11660105 861.3141767 358.7585967 1363.869757 3.801636446 1.926620572 0.001315669 0.526214153chr14_36396613_36398963 278.7623639 116.1694504 441.3552775 3.799237029 1.925709722 0.017802941 1chr22_27726596_27728632 188.5573241 78.58521643 298.5294317 3.798798874 1.925543331 0.04011555 1chr6_164550140_164552016 143.4548041 59.79309945 227.1165088 3.798373239 1.925381676 0.066285599 1chr5_56750667_56753155 170.0796863 70.89753221 269.2618404 3.79790145 1.92520247 0.048848198 1chr12_104974341_104975893 98.35228422 41.00098248 155.7035859 3.797557437 1.925071785 0.12024233 1chr3_17770683_17773286 194.5214194 81.14777783 307.8950609 3.794251293 1.923815231 0.037910022 1chr10_49652199_49654691 352.0875632 146.9201872 557.2549392 3.792909264 1.923304859 0.010606763 1chr5_171855098_171857209 149.4188995 62.35566086 236.4821381 3.792472645 1.923138774 0.062025034 1chr1_58659308_58660657 61.39700866 25.62561405 97.16840326 3.791846824 1.922900686 0.217348202 1chr2_2235533_2237110 96.16913517 40.14679535 152.191475 3.790874805 1.922530812 0.12459792 1chr3_123056486_123058214 130.9412617 54.66797664 207.2145467 3.790419171 1.922357401 0.077785738 1chrX_106719818_106722859 216.7800034 90.54383632 343.0161705 3.788398907 1.92158825 0.030703178 1chr1_100469322_100470556 85.83874175 35.87585967 135.8016238 3.78532041 1.920415423 0.145885736 1chr2_220424723_220428011 273.810714 114.4610761 433.1603519 3.784346318 1.920044121 0.018842929 1chr1_116166671_116168503 145.0526014 60.64728659 229.4579161 3.783481983 1.919714575 0.065770473 1chr21_33667298_33668382 51.06661524 21.35467838 80.77855211 3.782709844 1.919420117 0.263365457 1chr8_37476031_37479659 432.974655 181.0876726 684.8616374 3.781934063 1.91912421 0.006666991 1chr12_104581186_104582287 59.21385961 24.77142692 93.6562923 3.780819434 1.91869895 0.226912196 1chr6_150676480_150677569 59.21385961 24.77142692 93.6562923 3.780819434 1.91869895 0.226912196 1chr8_119556149_119558022 177.6415788 74.31428075 280.9688769 3.780819434 1.91869895 0.045698251 1chr22_43569841_43572028 193.9360676 81.14777783 306.7243573 3.779824482 1.918319244 0.038654054 1chr7_6077970_6079800 142.8694523 59.79309945 225.9458052 3.778793995 1.917925871 0.067751109 1chr4_89645843_89647148 83.65559271 35.02167254 132.2895129 3.777361368 1.917378808 0.151494313 1chr8_11731913_11733337 99.95008144 41.85516962 158.0449933 3.77599696 1.916857603 0.118976019 1chr2_220565891_220568918 340.5864661 142.6492516 538.5236807 3.775159525 1.916537609 0.011720461 1chr21_45485764_45488584 281.3726065 117.8778246 444.8673884 3.773970124 1.916083002 0.017975173 1chr8_8558220_8560964 230.3059913 96.52314626 364.0888363 3.772036557 1.915343658 0.027581972 1chr1_57180871_57181886 48.88346619 20.50049124 77.26644115 3.769004374 1.914183469 0.275803308 1chr1_102554469_102556096 97.76693239 41.00098248 154.5328823 3.769004374 1.914183469 0.123260488 1chr10_29506013_29507204 65.17795493 27.33398832 103.0219215 3.769004374 1.914183469 0.205408976 1chr2_206750002_206751238 57.03071056 23.91723978 90.14418134 3.769004374 1.914183469 0.237042415 1chr20_24747955_24749850 195.5338648 82.00196497 309.0657646 3.769004374 1.914183469 0.038430409 1chr21_42568301_42569603 73.32519929 30.75073686 115.8996617 3.769004374 1.914183469 0.179250269 1chr7_155616726_155618109 73.32519929 30.75073686 115.8996617 3.769004374 1.914183469 0.179250269 1chrX_4910301_4911638 81.47244366 34.1674854 128.7774019 3.769004374 1.914183469 0.157384428 1chr2_152864654_152868585 356.295603 149.4827486 563.1084575 3.767046449 1.913433822 0.010693768 1chr1_64281876_64285225 282.9704038 118.7320118 447.2087957 3.766539361 1.913239606 0.017913012 1chr2_44231166_44234115 274.8231594 115.3152632 434.3310555 3.766466323 1.91321163 0.019089826 1chr12_299689_303131 297.0817434 124.7113217 469.4521652 3.764310719 1.912385718 0.016142535 1chr6_43488871_43494416 681.6000258 286.1526902 1077.047361 3.763890392 1.912224616 0.00239435 0.688385255chr3_40760986_40762945 128.1727608 53.81378951 202.5317321 3.763565695 1.912100155 0.082092133 1chr1_85355825_85357781 120.0255164 50.39704097 189.6539919 3.763196971 1.911958804 0.091169503 1chr2_203671369_203673744 240.0510329 100.7940819 379.3079838 3.763196971 1.911958804 0.025549316 1chr1_46719958_46721514 87.43653898 36.73004681 138.1430311 3.761036077 1.911130145 0.14418946 1chr8_113341355_113342682 79.28929461 33.31329827 125.265291 3.760218816 1.910816618 0.16357404 1chr10_62017589_62018817 62.99480588 26.47980119 99.50981057 3.757951575 1.909946476 0.21424778 1chr3_51535442_51538349 188.9844176 79.43940356 298.5294317 3.757951575 1.909946476 0.041473559 1chr5_174469180_174470497 62.99480588 26.47980119 99.50981057 3.757951575 1.909946476 0.21424778 1chr7_28475345_28476407 62.99480588 26.47980119 99.50981057 3.757951575 1.909946476 0.21424778 1chr11_520322_521939 109.695123 46.12610529 173.2641408 3.756314123 1.909317714 0.105094081 1chr5_168558536_168561084 164.5426845 69.18915794 259.8962111 3.756314123 1.909317714 0.053773627 1chr5_99397462_99398478 54.84756151 23.06305265 86.63207038 3.756314123 1.909317714 0.24778356 1chr10_116352121_116354012 101.5478787 42.70935675 160.3864006 3.755298903 1.908927744 0.117724552 1chr3_14089103_14090643 77.10614556 32.45911113 121.75318 3.75097086 1.907264055 0.170082552 1chr4_124523467_124524947 115.6592183 48.6886667 182.62977 3.75097086 1.907264055 0.097339321 1chr1_111156922_111160387 369.2362391 155.4620586 583.0104196 3.750178178 1.906959142 0.010092069 1chr3_34148366_34149884 68.9589012 29.04236259 108.8754398 3.74884927 1.90644782 0.194369902 1chr20_23222119_23223855 99.36472961 41.85516962 156.8742896 3.748026613 1.906131197 0.12193921 1chr10_125661954_125663074 60.81165683 25.62561405 95.99769961 3.746161923 1.905413259 0.223594789 1chr2_215571153_215572356 60.81165683 25.62561405 95.99769961 3.746161923 1.905413259 0.223594789 1chr9_16150092_16151681 91.21748524 38.43842108 143.9965494 3.746161923 1.905413259 0.137359413 1chr7_27902217_27904558 279.6165511 117.8778246 441.3552775 3.744175623 1.904648107 0.018882233 1chr9_16704666_16705777 52.66441246 22.20886551 83.11995942 3.7426477 1.904059252 0.259185058 1chrX_69715640_69718530 210.6576499 88.83546205 332.4798377 3.7426477 1.904059252 0.034052181 1chr2_234388161_234390013 224.7689895 94.81477199 354.7232071 3.741223014 1.903509968 0.029941609 1chr3_51611983_51613824 119.4401646 50.39704097 188.4832883 3.73996736 1.903025679 0.093305224 1chr13_28122835_28124683 141.6987487 59.79309945 223.6043979 3.739635508 1.902897662 0.070775044 1chr8_28402259_28404214 141.6987487 59.79309945 223.6043979 3.739635508 1.902897662 0.070775044 1chr15_51657482_51662903 483.7247538 204.1507253 763.2987822 3.738898215 1.902613197 0.005512744 0.996814992chr12_65323526_65326892 534.2060172 225.5054037 842.9066307 3.737855577 1.902210827 0.004390424 0.920540035chr6_67922584_67923864 89.0343362 37.58423394 140.4844385 3.737855577 1.902210827 0.142515906 1chr2_1910559_1916265 584.6872806 246.860082 922.5144792 3.736993327 1.901877987 0.003571976 0.850907414chr10_13451201_13452969 139.5155996 58.93891232 220.0922869 3.734244122 1.900816245 0.072939476 1chr2_239090906_239092305 117.2570156 49.54285383 184.9711773 3.733559191 1.900551604 0.096408512 1chr2_203438847_203439971 58.62850778 24.77142692 92.48558865 3.733559191 1.900551604 0.233488556 1chr3_58520602_58524513 388.1409704 164.0039299 612.2780109 3.733313044 1.900456486 0.009225706 1chr2_54918476_54920304 109.1097712 46.12610529 172.0934371 3.730933622 1.899536693 0.107628314 1chr2_184799450_184800747 72.73984746 30.75073686 114.7289581 3.730933622 1.899536693 0.184142043 1chr3_57892562_57894478 109.1097712 46.12610529 172.0934371 3.730933622 1.899536693 0.107628314 1chr5_71168543_71171476 181.8496187 76.87684216 286.8223952 3.730933622 1.899536693 0.045727836 1chr9_96405101_96406046 50.48126342 21.35467838 79.60784846 3.727887962 1.898358502 0.271301729 1chr5_137580876_137583279 230.147733 97.3773334 362.9181327 3.726926175 1.897986242 0.028970029 1chr7_27881158_27883188 115.0738665 48.6886667 181.4590663 3.726926175 1.897986242 0.099644383 1chr7_98217956_98220163 129.1852062 54.66797664 203.7024358 3.726174778 1.897695346 0.08322565 1chr8_72999934_73000895 64.5926031 27.33398832 101.8512179 3.726174778 1.897695346 0.211201524 1chr1_110808805_110810179 92.81528247 39.29260821 146.3379567 3.724312622 1.896974179 0.135799397 1chr4_3877145_3879260 199.7419046 84.56452637 314.9192829 3.724011667 1.896857593 0.038493991 1chr7_98326195_98327916 121.0379618 51.2512281 190.8246956 3.723319472 1.896589409 0.092429796 1chr8_10555052_10556771 121.0379618 51.2512281 190.8246956 3.723319472 1.896589409 0.092429796 1chr5_174756638_174759503 262.1513587 111.0443276 413.2583898 3.72156236 1.895908411 0.022252485 1chr2_191082804_191086795 290.374038 123.0029474 457.7451286 3.721415934 1.895851646 0.017889562 1chr1_215967038_215968555 98.77937778 41.85516962 155.7035859 3.720056265 1.895324442 0.124970895 1chr15_58520042_58521596 112.8907175 47.83447956 177.9469554 3.720056265 1.895324442 0.103019818 1chr20_24665409_24667204 127.0020572 53.81378951 200.1903248 3.720056265 1.895324442 0.085893601 1chr4_59852439_59853755 56.44535873 23.91723978 88.97347769 3.720056265 1.895324442 0.243971502 1chr5_148340393_148342929 195.3756065 82.8561521 307.8950609 3.716019307 1.893757998 0.040476501 1chr2_7134520_7136013 90.63213342 38.43842108 142.8258458 3.715705322 1.893636092 0.140865183 1chr6_3098119_3099549 62.40945405 26.47980119 98.33910692 3.71374038 1.892872963 0.220337804 1chrX_74030891_74032056 62.40945405 26.47980119 98.33910692 3.71374038 1.892872963 0.220337804 1chr18_77378265_77381058 173.1170225 73.46009361 272.7739513 3.713226296 1.89267324 0.050879007 1chr22_24496360_24499140 193.1924575 82.00196497 304.38295 3.711898247 1.892157163 0.041516519 1chr1_81195512_81198018 227.3792322 96.52314626 358.235318 3.711392883 1.891960731 0.030180339 1chr12_26675880_26677466 102.5603241 43.56354389 161.5571042 3.708539063 1.890850965 0.119345527 1chr14_39785462_39787650 102.5603241 43.56354389 161.5571042 3.708539063 1.890850965 0.119345527 1chr5_74950757_74953162 136.7470987 58.08472518 215.4094723 3.708539063 1.890850965 0.07686184 1chr3_58100876_58107286 599.0674556 254.5477662 943.5871449 3.706915833 1.890219359 0.003566623 0.850907414chr14_87588926_87589971 54.26220969 23.06305265 85.46136672 3.705553121 1.889688907 0.255090431 1chr2_140126877_140128780 114.4885147 48.6886667 180.2883627 3.70288149 1.888648377 0.102000557 1chr8_9448675_9450739 134.5639497 57.23053805 211.8973613 3.702522614 1.888508547 0.079260392 1chr2_205752092_205753600 80.30174 34.1674854 126.4359946 3.700477021 1.887711258 0.165818739 1chr2_192042770_192044468 106.3412703 45.27191816 167.4106225 3.697891083 1.886702733 0.11406025 1chr8_131626930_131629404 152.4562356 64.91822226 239.994249 3.696870319 1.886304437 0.064609244 1chr5_15259230_15262588 310.8765666 132.3990059 489.3541273 3.696055902 1.885986577 0.015928118 1chr2_240290091_240293956 336.9160969 143.5034387 530.3287552 3.69558221 1.885801668 0.013362222 1chr8_117602138_117604127 112.3053656 47.83447956 176.7762517 3.69558221 1.885801668 0.105469087 1chr15_58570835_58572643 118.269461 50.39704097 186.1418809 3.693508138 1.884991756 0.097718505 1chr6_139764264_139765674 72.15449564 30.75073686 113.5582544 3.692862871 1.884739691 0.189155 1chr7_151402126_151404145 150.2730866 64.06403513 236.4821381 3.691340041 1.884144643 0.066517955 1chr2_153622880_153623966 52.07906064 22.20886551 81.94925576 3.689934352 1.883595149 0.266897127 1chr3_155323122_155327009 312.4743638 133.2531931 491.6955346 3.689934352 1.883595149 0.015876198 1chrX_133895718_133896681 52.07906064 22.20886551 81.94925576 3.689934352 1.883595149 0.266897127 1chr10_24598412_24600192 116.0863119 49.54285383 182.62977 3.686298948 1.882173074 0.100993256 1chr16_24654909_24656063 58.04315595 24.77142692 91.31488499 3.686298948 1.882173074 0.240235161 1chr21_26521015_26522167 58.04315595 24.77142692 91.31488499 3.686298948 1.882173074 0.240235161 1chr15_52553125_52557285 302.1439704 128.9822574 475.3056834 3.685047021 1.881683028 0.017180861 1chr3_174941528_174942704 64.00725127 27.33398832 100.6805142 3.683345183 1.881016605 0.217141031 1chr20_35983348_35988388 395.9716983 169.1290527 622.8143438 3.682479939 1.880677666 0.009498854 1chr1_65091381_65092579 69.97134659 29.89654973 110.0461435 3.680897778 1.880057685 0.196998227 1chr8_101599861_101602209 157.8349791 67.48078367 248.1891746 3.677923715 1.878891557 0.061804142 1chr13_34571078_34572790 119.8672582 51.2512281 188.4832883 3.677634571 1.878778133 0.096772195 1chr22_22137417_22140401 245.6986117 105.0650176 386.3322057 3.677077438 1.878559559 0.026771229 1chr5_17436130_17437694 87.86363254 37.58423394 138.1430311 3.675557984 1.877963281 0.149922999 1chr7_3973153_3974946 137.7595441 58.93891232 216.5801759 3.67465512 1.877608854 0.077887255 1chr12_3450203_3452109 149.6877348 64.06403513 235.3114344 3.67306608 1.876984851 0.067943173 1chr10_12707105_12708858 155.6518301 66.62659653 244.6770636 3.672363236 1.876708764 0.063599507 1chr3_87146439_87147868 105.7559185 45.27191816 166.2399188 3.672031705 1.876578515 0.116817322 1chr1_89449620_89452318 223.4400276 95.66895913 351.2110961 3.671108156 1.876215619 0.032604827 1chr12_3229198_3230306 279.3000345 119.5861989 439.0138702 3.671108156 1.876215619 0.020666426 1chr6_44304586_44305585 55.86000691 23.91723978 87.80277403 3.671108156 1.876215619 0.251080896 1chr7_2974734_2975828 55.86000691 23.91723978 87.80277403 3.671108156 1.876215619 0.251080896 1chr10_14295402_14298012 197.4004973 84.56452637 310.2364682 3.668636029 1.87524378 0.041537826 1chr1_88898638_88899825 67.78819754 29.04236259 106.5340325 3.668228856 1.875083649 0.205271081 1chr1_74105902_74107428 67.78819754 29.04236259 106.5340325 3.668228856 1.875083649 0.205271081 1chr1_51431954_51434135 141.5404904 60.64728659 222.4336942 3.667661106 1.874860339 0.074907441 1chr2_205450242_205451560 73.75229286 31.604924 115.8996617 3.66713939 1.874655105 0.186613285 1chr2_168862558_168863901 79.71638818 34.1674854 125.265291 3.666213345 1.874290742 0.170189323 1chr15_48323007_48324606 91.64457881 39.29260821 143.9965494 3.66472362 1.8737044 0.142755534 1chr5_139702178_139704297 195.2173483 83.71033924 306.7243573 3.664115569 1.873465008 0.042603457 1chr5_17123650_17125300 145.3214367 62.35566086 228.2872125 3.661050325 1.872257605 0.072080747 1chr10_52833229_52835061 131.210097 56.37635091 206.0438431 3.65479212 1.86978935 0.085463899 1chr13_90027682_90029254 101.3896204 43.56354389 159.2156969 3.65479212 1.86978935 0.125257129 1chr2_99295695_99296892 59.64095318 25.62561405 93.6562923 3.65479212 1.86978935 0.236573891 1chr2_200645341_200647020 95.42552508 41.00098248 149.8500677 3.65479212 1.86978935 0.136050219 1chr5_145807795_145808886 65.60504849 28.18817546 103.0219215 3.65479212 1.86978935 0.214004069 1chr7_140443620_140444493 47.71276254 20.50049124 74.92503384 3.65479212 1.86978935 0.292810208 1chr8_36974203_36975398 65.60504849 28.18817546 103.0219215 3.65479212 1.86978935 0.214004069 1chr8_98821049_98826218 504.7649529 216.9635323 792.5663736 3.652993502 1.869079187 0.005766079 1chr3_8923244_8924952 176.7397105 76.02265502 277.4567659 3.649658985 1.867761668 0.051853013 1chr8_120691873_120694568 293.8384678 126.419696 461.2572396 3.648618484 1.867350304 0.019088961 1chr8_128236725_128238444 129.0269479 55.52216378 202.5317321 3.647763673 1.867012265 0.088195671 1chr13_114749476_114752140 351.2962719 151.1911229 551.4014209 3.647048916 1.86672955 0.013027315 1chr21_27283833_27285406 111.134662 47.83447956 174.4348444 3.646634102 1.866565449 0.11053188 1chr5_67046968_67048588 105.1705667 45.27191816 165.0692152 3.646172327 1.866382748 0.119636368 1chr2_200994412_200996228 99.20647135 42.70935675 155.7035859 3.645655139 1.866178096 0.129769159 1chr8_68167142_68168599 99.20647135 42.70935675 155.7035859 3.645655139 1.866178096 0.129769159 1chr1_66640315_66643934 279.7271281 120.440386 439.0138702 3.645071928 1.865947283 0.0212608 1chr21_43160531_43164767 452.1005405 194.7546668 709.4464142 3.642769777 1.86503582 0.007502804 1chr14_39778575_39779970 69.38599476 29.89654973 108.8754398 3.641739291 1.864627645 0.202386263 1chr5_84900237_84901511 69.38599476 29.89654973 108.8754398 3.641739291 1.864627645 0.202386263 1chr1_48711831_48713658 160.4452217 69.18915794 251.7012856 3.637871786 1.863094697 0.06212052 1chr2_134522644_134527143 320.8904435 138.3783159 503.4025711 3.637871786 1.863094697 0.016045091 1chr14_22865814_22868557 211.9389306 91.39802345 332.4798377 3.637713652 1.863031984 0.037409438 1chr3_13875503_13877033 102.9874176 44.41773102 161.5571042 3.637221004 1.862836589 0.12387831 1chr5_43936356_43937381 51.49370881 22.20886551 80.77855211 3.637221004 1.862836589 0.274811966 1chr5_169182903_169184516 102.9874176 44.41773102 161.5571042 3.637221004 1.862836589 0.12387831 1chr12_7252614_7254957 130.6247452 56.37635091 204.8731394 3.634026255 1.861568843 0.087377486 1chr1_58628345_58629871 79.13103635 34.1674854 124.0945873 3.631949669 1.86074421 0.174664964 1chr7_26538746_26539898 79.13103635 34.1674854 124.0945873 3.631949669 1.86074421 0.174664964 1chr3_58162181_58165603 371.7988005 160.5871814 583.0104196 3.63049164 1.860164931 0.011780845 1chr1_3235516_3240410 585.3355283 252.839392 917.8316645 3.630097578 1.860008329 0.00431539 0.915344059chr1_110726811_110728551 146.3338821 63.20984799 229.4579161 3.630097578 1.860008329 0.073013146 1chr9_22117321_22118742 67.20284571 29.04236259 105.3633288 3.627918648 1.859142106 0.210926363 1chrX_132227847_132229349 67.20284571 29.04236259 105.3633288 3.627918648 1.859142106 0.210926363 1chr7_27594868_27596223 94.84017325 41.00098248 148.679364 3.626239056 1.858474037 0.139434201 1chr12_110124593_110125643 61.2387504 26.47980119 95.99769961 3.62531799 1.858107545 0.232986836 1chr13_100204487_100205591 61.2387504 26.47980119 95.99769961 3.62531799 1.858107545 0.232986836 1chr2_237301881_237302932 61.2387504 26.47980119 95.99769961 3.62531799 1.858107545 0.232986836 1chr20_23753788_23755972 183.7162512 79.43940356 287.9930988 3.62531799 1.858107545 0.049437437 1chr10_97967673_97970343 177.7521559 76.87684216 278.6274696 3.624335519 1.857716517 0.052512128 1chr8_10661467_10664099 266.6282338 115.3152632 417.9412044 3.624335519 1.857716517 0.024067795 1chr5_175279879_175283796 493.6909494 213.5467838 773.8351151 3.623726387 1.857474027 0.006362756 1chr21_37416006_37419822 288.301466 124.7113217 451.8916103 3.623501091 1.857384329 0.020498766 1chr12_65723951_65726051 110.5493102 47.83447956 173.2641408 3.622160047 1.856850294 0.113147554 1chr2_198341489_198343487 104.5852148 45.27191816 163.8985115 3.620312949 1.856114413 0.122518607 1chr4_178376124_178378557 181.5331021 78.58521643 284.4809879 3.620031869 1.856002398 0.050764506 1chr12_59234494_59235666 76.9478873 33.31329827 120.5824763 3.619649888 1.855850159 0.181660057 1chr3_5067428_5068966 98.62111952 42.70935675 154.5328823 3.618244199 1.85528978 0.132954005 1chr7_8470021_8470889 49.31055976 21.35467838 77.26644115 3.618244199 1.85528978 0.28781171 1chr7_66565244_66567004 98.62111952 42.70935675 154.5328823 3.618244199 1.85528978 0.132954005 1chr1_93550560_93552514 163.6408162 70.89753221 256.3841002 3.616262685 1.854499479 0.061158264 1chr16_2166140_2169957 364.6640015 158.02462 571.303383 3.615280854 1.854107728 0.012559248 1chr1_109212614_109214715 157.6767209 68.3349708 247.0184709 3.614817831 1.853922944 0.065246893 1chr1_85412784_85414352 108.3661611 46.98029243 169.7520298 3.613260391 1.853301227 0.117075212 1chr10_6169978_6171643 108.3661611 46.98029243 169.7520298 3.613260391 1.853301227 0.117075212 1chr12_100754312_100755501 65.01969667 28.18817546 101.8512179 3.613260391 1.853301227 0.219943697 1chr10_12429865_12432260 254.1146913 110.1901404 398.0392423 3.612294537 1.852915531 0.026909812 1chr4_24301945_24304540 167.4217625 72.60590648 262.2376184 3.611794565 1.852715836 0.0590148 1chr2_25310829_25313066 145.7485302 63.20984799 228.2872125 3.611576672 1.852628799 0.074579698 1chr1_111046660_111050066 354.3336081 153.7536843 554.9135319 3.609107218 1.851642003 0.013481943 1chr7_11394652_11395684 59.05560135 25.62561405 92.48558865 3.609107218 1.851642003 0.243313039 1chr4_148860545_148862101 96.43797048 41.85516962 151.0207713 3.608174873 1.851269262 0.137810459 1chr1_49288263_49289679 74.76473825 32.45911113 117.0703654 3.60670275 1.850680527 0.189021852 1chr1_64388406_64390079 90.47387516 39.29260821 141.6551421 3.605134618 1.850053132 0.15003914 1chr7_17278978_17280350 90.47387516 39.29260821 141.6551421 3.605134618 1.850053132 0.15003914 1chr1_109386048_109387148 53.09150603 23.06305265 83.11995942 3.604031118 1.849611468 0.270281412 1chr7_151408178_151410375 228.075161 99.08570767 357.0646144 3.603593523 1.849436288 0.03364879 1chr7_55246533_55250604 1515.797294 658.5782811 2373.016306 3.603241064 1.849295174 0.000697318 0.36512849chr1_58684815_58688333 312.5849409 135.8157545 489.3541273 3.603073363 1.849228027 0.017758259 1chr1_58898024_58899241 84.50977984 36.73004681 132.2895129 3.601670141 1.848666058 0.163769408 1chr2_127891227_127893691 169.0195597 73.46009361 264.5790257 3.601670141 1.848666058 0.058565426 1chr13_115022562_115027541 373.2383394 162.2955557 584.1811232 3.599489344 1.847792247 0.012216459 1chr3_59111659_59112955 78.54568452 34.1674854 122.9238836 3.597685993 1.847069273 0.179247773 1chr11_121465285_121467802 223.7088629 97.3773334 350.0403925 3.594680407 1.84586351 0.035264548 1chr18_46196483_46198036 103.999863 45.27191816 162.7278079 3.594453571 1.845772469 0.125465275 1chr3_71992917_71994491 103.999863 45.27191816 162.7278079 3.594453571 1.845772469 0.125465275 1chr5_72103825_72105322 88.29072611 38.43842108 138.1430311 3.593878918 1.845541803 0.155729567 1chr10_49957165_49958949 170.6173569 74.31428075 266.9204331 3.591778463 1.844698369 0.058120456 1chr12_107436058_107437206 56.8724523 24.77142692 88.97347769 3.591778463 1.844698369 0.254253054 1chr3_172529070_172530265 56.8724523 24.77142692 88.97347769 3.591778463 1.844698369 0.254253054 1chr4_95573265_95575785 154.90822 67.48078367 242.3356563 3.591180232 1.84445806 0.068556464 1chr22_29931847_29936361 523.1949095 228.0679651 818.321854 3.588061365 1.843204564 0.005948274 1chr5_1839962_1843854 656.4298973 286.1526902 1026.707104 3.587969428 1.843167598 0.003636199 0.858657824chr7_122358667_122359798 66.61749389 29.04236259 104.1926252 3.587608441 1.84302244 0.216721792 1chr12_59343617_59345012 92.07167238 40.14679535 143.9965494 3.586750777 1.842677504 0.148223433 1chr8_136801384_136806631 867.6252178 378.4049008 1356.845535 3.585697573 1.842253813 0.002038609 0.636342509chr8_8387090_8388884 193.8883863 84.56452637 303.2122463 3.585572572 1.842203518 0.046542802 1chr2_134546957_134547978 50.90835698 22.20886551 79.60784846 3.584507656 1.841774974 0.282933648 1chr8_8708051_8709241 254.5417849 111.0443276 398.0392423 3.584507656 1.841774974 0.027693346 1chr14_40202460_40204735 315.1951835 137.5241287 492.8662382 3.583852832 1.841511395 0.017896558 1chr1_54545977_54547932 111.5617556 48.6886667 174.4348444 3.582658065 1.841030357 0.114583864 1chr10_95492233_95494283 111.5617556 48.6886667 174.4348444 3.582658065 1.841030357 0.114583864 1chr8_12610266_12613786 309.2310882 134.9615673 483.500609 3.582505883 1.840969074 0.018658619 1chr3_31670879_31672213 86.10757706 37.58423394 134.6309202 3.582111595 1.840810283 0.161699685 1chrX_67908879_67910160 86.10757706 37.58423394 134.6309202 3.582111595 1.840810283 0.161699685 1chr11_47372671_47374765 121.3067971 52.95960237 189.6539919 3.581106795 1.840405543 0.101565603 1chr8_27504959_27506067 60.65339857 26.47980119 94.82699595 3.581106795 1.840405543 0.239552201 1chr20_3255860_3258935 217.1594158 94.81477199 339.5040596 3.580708496 1.840245074 0.037877783 1chr2_168912387_168914188 105.5976602 46.12610529 165.0692152 3.578650617 1.839415701 0.124071669 1chr8_8662779_8665128 246.3945406 107.627579 385.1615021 3.578650617 1.839415701 0.029736279 1chr1_60968358_60972366 326.5380223 142.6492516 510.426793 3.57819468 1.839231883 0.016741888 1chr20_24362998_24366095 256.1395821 111.8985147 400.3806496 3.578069384 1.839181364 0.027555274 1chr6_44695053_44696664 185.741142 81.14777783 290.3345061 3.577849128 1.839092553 0.050670626 1chr3_28718422_28719886 115.3427018 50.39704097 180.2883627 3.577360083 1.838895342 0.109623139 1chr15_101671158_101675219 320.573927 140.0866901 501.0611638 3.576793507 1.838666832 0.017435414 1chr12_53670055_53674867 494.3868783 216.1093452 772.6644115 3.575340117 1.83808049 0.00687858 1chr5_158630895_158632321 89.88852333 39.29260821 140.4844385 3.575340117 1.83808049 0.153807832 1chr12_65330676_65333206 164.0679098 71.75171934 256.3841002 3.573211939 1.837221487 0.063187021 1chr4_4645264_4648735 247.9923378 108.4817662 387.5029094 3.572055684 1.83675457 0.02958115 1chr12_10025025_10026085 64.43434484 28.18817546 100.6805142 3.571728663 1.836622486 0.226031168 1chr15_76667955_76669121 64.43434484 28.18817546 100.6805142 3.571728663 1.836622486 0.226031168 1chr3_63890805_63892156 64.43434484 28.18817546 100.6805142 3.571728663 1.836622486 0.226031168 1chr3_71602683_71603852 64.43434484 28.18817546 100.6805142 3.571728663 1.836622486 0.226031168 1chr10_48426477_48429285 296.7175457 129.8364445 463.5986469 3.570635722 1.836180957 0.020639793 1chr2_1848930_1851206 251.773284 110.1901404 393.3564277 3.569796954 1.835842018 0.028785647 1chr2_171595034_171596602 83.92442801 36.73004681 131.1188092 3.569796954 1.835842018 0.167966776 1chr5_169168733_169174806 813.7901016 356.1960353 1271.384168 3.569338347 1.835656665 0.002404238 0.689399359chr15_63501930_63506540 364.9328368 159.7329943 570.1326794 3.56928562 1.835635353 0.013392186 1chr15_51542879_51544568 93.6694696 41.00098248 146.3379567 3.569132929 1.835573634 0.146436496 1chr3_72420931_72423364 187.3389392 82.00196497 292.6759134 3.569132929 1.835573634 0.050312487 1chr13_100703378_100705181 113.1595528 49.54285383 176.7762517 3.568148341 1.835175595 0.113364044 1chr17_60727278_60729192 113.1595528 49.54285383 176.7762517 3.568148341 1.835175595 0.113364044 1chr1_78055536_78057447 122.9045944 53.81378951 191.9953992 3.56777326 1.835023932 0.100545654 1chr3_66659625_66661923 210.6099686 92.25221059 328.9677267 3.565960367 1.834290669 0.040741731 1chr1_118697248_118698372 68.21529111 29.89654973 106.5340325 3.563422317 1.833263474 0.213563805 1chr11_126042636_126044047 97.45041587 42.70935675 152.191475 3.563422317 1.833263474 0.139541825 1chr2_169166248_169168174 116.940499 51.2512281 182.62977 3.563422317 1.833263474 0.108480123 1chr4_103394134_103395718 97.45041587 42.70935675 152.191475 3.563422317 1.833263474 0.139541825 1chr4_140752410_140753779 68.21529111 29.89654973 106.5340325 3.563422317 1.833263474 0.213563805 1chr7_68846036_68847455 87.70537428 38.43842108 136.9723275 3.563422317 1.833263474 0.15966447 1chrX_24052667_24053681 48.72520794 21.35467838 76.09573749 3.563422317 1.833263474 0.296393978 1chr8_125520681_125525370 582.5193462 255.4019534 909.636739 3.561588809 1.832520965 0.004937831 0.965381333chr3_25867104_25869445 247.4069859 108.4817662 386.3322057 3.561263975 1.832389378 0.030086885 1chr7_2827443_2831402 455.8338055 199.8797896 711.7878215 3.561079501 1.832314644 0.008400767 1chr6_167606776_167610348 212.2077659 93.10639772 331.309134 3.558392786 1.831225769 0.040485608 1chr13_111006278_111008502 215.9887121 94.81477199 337.1626523 3.556013954 1.830260986 0.039266939 1chr5_60575680_60577544 134.2474332 58.93891232 209.555954 3.555477117 1.830043171 0.088750631 1chr10_3971544_3972612 52.5061542 23.06305265 81.94925576 3.553270116 1.829147365 0.278173276 1chr5_171040506_171043446 315.0369252 138.3783159 491.6955346 3.553270116 1.829147365 0.018653612 1chr13_21880054_21881495 95.26726682 41.85516962 148.679364 3.552234178 1.828726693 0.144678013 1chr14_39936901_39938400 95.26726682 41.85516962 148.679364 3.552234178 1.828726693 0.144678013 1chr8_123159921_123161336 95.26726682 41.85516962 148.679364 3.552234178 1.828726693 0.144678013 1chr2_54850997_54853199 138.0283794 60.64728659 215.4094723 3.551840229 1.828566687 0.085238899 1chr2_150060076_150062411 223.5506047 98.23152053 348.8696888 3.551504516 1.82843032 0.036977232 1chr1_51927651_51929244 85.52222524 37.58423394 133.4602165 3.550962798 1.828210246 0.165809993 1chr1_114427280_114428606 85.52222524 37.58423394 133.4602165 3.550962798 1.828210246 0.165809993 1chr5_17283354_17286186 184.5704383 81.14777783 287.9930988 3.548995506 1.827410747 0.052656432 1chr10_48647478_48649600 108.7932547 47.83447956 169.7520298 3.548737884 1.827306018 0.12134492 1chr4_154076891_154080588 217.5865094 95.66895913 339.5040596 3.548737884 1.827306018 0.039024726 1chr5_151431085_151433055 141.8093257 62.35566086 221.2629906 3.548402623 1.827169716 0.081913704 1chr3_155989452_155991621 132.0642841 58.08472518 206.0438431 3.547298234 1.826720628 0.091572816 1chr18_45692746_45695916 221.3674556 97.3773334 345.3575779 3.546591037 1.826432981 0.037867222 1chr4_11670481_11672601 155.3353136 68.3349708 242.3356563 3.546290479 1.826310713 0.070843952 1chr3_25711571_25713011 112.5742009 49.54285383 175.6055481 3.54451822 1.825589546 0.116002168 1chr6_149328273_149329346 56.28710047 24.77142692 87.80277403 3.54451822 1.825589546 0.261531386 1chr1_93406762_93411833 454.0777501 199.8797896 708.2757105 3.543508385 1.825178463 0.008715422 1chr3_14815034_14816735 159.1162598 70.04334507 248.1891746 3.543365531 1.8251203 0.068252188 1chr4_184231351_184232704 79.55812992 35.02167254 124.0945873 3.543365531 1.8251203 0.181467792 1chr8_36331129_36333936 182.3872893 80.2935907 284.4809879 3.543009914 1.824975502 0.054074915 1chr8_71964238_71965543 102.8291594 45.27191816 160.3864006 3.542734814 1.824863479 0.13155693 1chr7_73516531_73520538 527.6717847 232.3389007 823.0046686 3.542259458 1.824669888 0.006322192 1chr8_56547018_56549176 172.6422477 76.02265502 269.2618404 3.54186315 1.82450847 0.05954667 1chr5_175267940_175275830 1233.364561 543.2630179 1923.466103 3.540579866 1.82398566 0.001157413 0.49576642chr1_241718372_241719833 116.3551472 51.2512281 181.4590663 3.540579866 1.82398566 0.110975343 1chr12_10004136_10005847 116.3551472 51.2512281 181.4590663 3.540579866 1.82398566 0.110975343 1chr12_109825026_109826121 69.81308833 30.75073686 108.8754398 3.540579866 1.82398566 0.210468672 1chr10_22841354_22844547 259.7622701 114.4610761 405.0634642 3.538875206 1.823290888 0.02805846 1chr5_59648397_59649810 106.6101056 46.98029243 166.2399188 3.53850328 1.823139256 0.125603028 1chr1_77907134_77908631 120.1360935 52.95960237 187.3125846 3.5368956 1.822483635 0.106240946 1chr20_19964351_19968221 434.0023151 191.3379183 676.6667119 3.536500857 1.822322611 0.00971174 1chr1_118225652_118228575 267.3241627 117.8778246 416.7705007 3.535614116 1.821960825 0.026614429 1chr21_42579090_42580663 123.9170398 54.66797664 193.1661029 3.5334416 1.821074065 0.101777611 1chr11_63986062_63987100 298.1570847 131.5448188 464.7693505 3.533163486 1.820960507 0.021440109 1chr1_116511135_116514095 174.2400449 76.87684216 271.6032477 3.532965716 1.820879749 0.059085982 1chr6_170666115_170669153 251.6150258 111.0443276 392.185724 3.531794308 1.820401323 0.030114505 1chr1_50731149_50732897 114.1719982 50.39704097 177.9469554 3.530900861 1.820036315 0.11475413 1chr18_63471053_63472344 63.84899301 28.18817546 99.50981057 3.530196934 1.819748667 0.232269423 1chr5_32794709_32796386 104.4269566 46.12610529 162.7278079 3.527889616 1.818805421 0.130052669 1chr8_10669762_10670571 145.0049202 64.06403513 225.9458052 3.526874396 1.818390197 0.080435545 1chr8_11118507_11120572 172.0568959 76.02265502 268.0911367 3.526463745 1.818222208 0.060731114 1chr12_83255472_83257754 135.2598786 59.79309945 210.7266577 3.52426383 1.81732193 0.089827727 1chr18_76755089_76756874 175.8378421 77.73102929 273.944655 3.52426383 1.81732193 0.058630115 1chr2_211441666_211443065 67.62993928 29.89654973 105.3633288 3.52426383 1.81732193 0.219358665 1chr6_76875519_76877077 148.7858664 65.7724094 231.7993234 3.52426383 1.81732193 0.077367983 1chr8_18247008_18248108 67.62993928 29.89654973 105.3633288 3.52426383 1.81732193 0.219358665 1chr1_71443880_71446331 166.0928005 73.46009361 258.7255075 3.521987174 1.816389655 0.064722474 1chr2_203452951_203454902 152.5668127 67.48078367 237.6528417 3.521785445 1.816307019 0.074457591 1chr8_102823118_102824798 125.514837 55.52216378 195.5075102 3.521251638 1.81608833 0.100748385 1chr8_28362291_28364120 98.46286126 43.56354389 153.3621786 3.520424762 1.81574951 0.141245033 1chr19_19116989_19119697 169.8737468 75.16846788 264.5790257 3.519814002 1.815499194 0.062434451 1chr5_74946386_74948427 133.0767295 58.93891232 207.2145467 3.515751115 1.813832944 0.092678695 1chr8_36433110_36434779 133.0767295 58.93891232 207.2145467 3.515751115 1.813832944 0.092678695 1chrX_67263529_67265805 133.0767295 58.93891232 207.2145467 3.515751115 1.813832944 0.092678695 1chr15_57895426_57898052 252.6274712 111.8985147 393.3564277 3.515296237 1.813646272 0.030458448 1chr2_152764436_152765624 75.19183182 33.31329827 117.0703654 3.514223192 1.813205821 0.196399892 1chr7_158830659_158832617 150.3836636 66.62659653 234.1407308 3.514223192 1.813205821 0.076683112 1chr14_79054672_79056777 154.1646099 68.3349708 239.994249 3.512026803 1.812303855 0.07381073 1chr6_79898175_79899843 109.8057001 48.6886667 170.9227334 3.51052401 1.811686395 0.122813949 1chr12_51795840_51802050 697.229015 309.2157429 1085.242287 3.509660527 1.811331492 0.003730399 0.869554825chr1_85916313_85919379 254.2252684 112.7527018 395.697835 3.50943107 1.811237167 0.030295268 1chr2_190075172_190076068 48.13985611 21.35467838 74.92503384 3.508600435 1.810895661 0.305200169 1chr8_102257173_102259000 161.7265025 71.75171934 251.7012856 3.507947794 1.810627277 0.068490787 1chr12_105469380_105470517 65.44679023 29.04236259 101.8512179 3.506988027 1.810232505 0.228744316 1chr6_38780148_38782455 190.3762754 84.56452637 296.1880244 3.502509115 1.808388805 0.052125292 1chr5_73814737_73815840 73.00868277 32.45911113 113.5582544 3.498501667 1.806737179 0.204462457 1chr1_5779896_5781848 128.7104314 57.23053805 200.1903248 3.497963353 1.806515175 0.098731763 1chr1_81330091_81331218 55.70174865 24.77142692 86.63207038 3.497257977 1.806224221 0.268994048 1chr12_3186960_3192500 445.6139892 198.1714153 693.056563 3.497257977 1.806224221 0.009776387 1chr6_170246863_170247842 55.70174865 24.77142692 86.63207038 3.497257977 1.806224221 0.268994048 1chr7_33804091_33805101 55.70174865 24.77142692 86.63207038 3.497257977 1.806224221 0.268994048 1chr5_67679239_67680699 94.09656317 41.85516962 146.3379567 3.496293482 1.805826291 0.151859855 1chr1_112486495_112488342 170.8861922 76.02265502 265.7497294 3.495664935 1.805566907 0.063167807 1chr12_107562026_107564287 213.061953 94.81477199 331.309134 3.494277601 1.804994227 0.042958568 1chr5_59798801_59802511 332.0273428 147.7743744 516.2803113 3.493706629 1.804758468 0.01814456 1chr8_119252296_119253706 97.87750944 43.56354389 152.191475 3.493551291 1.804694322 0.144660778 1chr1_80072721_80074036 59.48269492 26.47980119 92.48558865 3.492684405 1.804336288 0.253180736 1chr17_71973673_71975618 118.9653898 52.95960237 184.9711773 3.492684405 1.804336288 0.111116989 1chr7_56395699_56396772 59.48269492 26.47980119 92.48558865 3.492684405 1.804336288 0.253180736 1chr17_2377526_2380054 182.229031 81.14777783 283.3102842 3.491288262 1.803759479 0.056854111 1chr1_48332017_48333623 101.6584557 45.27191816 158.0449933 3.491016058 1.803646993 0.137921573 1chr1_111072300_111075166 245.4926722 109.3359533 381.6493911 3.490612005 1.803480005 0.033113857 1chr12_107850457_107852893 245.4926722 109.3359533 381.6493911 3.490612005 1.803480005 0.033113857 1chr11_60739479_60742901 431.5026495 192.1921054 670.8131936 3.490326474 1.803361988 0.010578871 1chr7_55073535_55075206 143.8342165 64.06403513 223.6043979 3.490326474 1.803361988 0.083882527 1chr12_7260215_7261296 63.26364119 28.18817546 98.33910692 3.488665205 1.802675154 0.238661432 1chr5_32034153_32035313 84.35152158 37.58423394 131.1188092 3.488665205 1.802675154 0.174318704 1chr21_46849750_46855091 682.3740652 304.0906201 1060.65751 3.487965232 1.80238566 0.004070155 0.900319516chr2_138979013_138982687 235.7476306 105.0650176 366.4302436 3.487652236 1.802256192 0.035872103 1chr7_22496547_22501153 605.5844361 269.9231347 941.2457376 3.487088051 1.802022793 0.005218733 0.9889824chr3_57107197_57109782 243.3095232 108.4817662 378.1372802 3.485722012 1.801457518 0.033869966 1chr1_87821619_87824388 222.2216428 99.08570767 345.3575779 3.485442916 1.801342 0.040141546 1chr21_46066038_46069023 335.2229373 149.4827486 520.9631259 3.485105343 1.801202264 0.018005826 1chr10_24671401_24673027 113.0012945 50.39704097 175.6055481 3.48444164 1.800927492 0.120108287 1chr2_202891627_202892901 70.82553372 31.604924 110.0461435 3.48193033 1.799887337 0.212958665 1chr8_125202631_125205469 244.9073204 109.3359533 380.4786875 3.479904606 1.799047758 0.033675874 1chr12_54247894_54249566 99.47530666 44.41773102 154.5328823 3.47908096 1.798706252 0.142920776 1chr14_30123443_30124753 74.60647999 33.31329827 115.8996617 3.47908096 1.798706252 0.201549025 1chr20_31714924_31716174 74.60647999 33.31329827 115.8996617 3.47908096 1.798706252 0.201549025 1chr6_82530171_82533587 273.5570933 122.1487603 424.9654263 3.47908096 1.798706252 0.027244701 1chrX_82228409_82229336 49.73765333 22.20886551 77.26644115 3.47908096 1.798706252 0.299813938 1chr21_18839696_18841064 78.38742626 35.02167254 121.75318 3.476509577 1.797639564 0.190974537 1chr7_27757865_27759511 107.0371992 47.83447956 166.2399188 3.475315721 1.797144048 0.130091716 1chr1_29006158_29007687 82.16837253 36.73004681 127.6066983 3.474177393 1.796671421 0.181155596 1chr9_33465986_33467212 82.16837253 36.73004681 127.6066983 3.474177393 1.796671421 0.181155596 1chr21_43927807_43929321 110.8181455 49.54285383 172.0934371 3.473627855 1.7964432 0.124263544 1chr1_58086910_58088111 57.29954587 25.62561405 88.97347769 3.472052514 1.795788768 0.264569275 1chr12_9113039_9114208 57.29954587 25.62561405 88.97347769 3.472052514 1.795788768 0.264569275 1chr3_47569582_47570697 57.29954587 25.62561405 88.97347769 3.472052514 1.795788768 0.264569275 1chr5_136899094_136900308 57.29954587 25.62561405 88.97347769 3.472052514 1.795788768 0.264569275 1chr3_50320906_50324653 290.2786756 129.8364445 450.7209067 3.471451397 1.795538973 0.02448241 1chr1_11789170_11790363 61.08049214 27.33398832 94.82699595 3.469197207 1.794601853 0.249149168 1chr18_47377951_47379042 61.08049214 27.33398832 94.82699595 3.469197207 1.794601853 0.249149168 1chr19_13379712_13380843 64.86143841 29.04236259 100.6805142 3.466677819 1.793553764 0.234977089 1chr21_42782895_42784066 64.86143841 29.04236259 100.6805142 3.466677819 1.793553764 0.234977089 1chr8_8727438_8730936 1049.125853 469.8029243 1628.448782 3.466238072 1.793370747 0.001844697 0.615533758chr14_87746542_87748070 101.0731039 45.27191816 156.8742896 3.46515668 1.792920586 0.141209542 1chr6_53363478_53365515 169.7154886 76.02265502 263.4083221 3.464866125 1.792799611 0.065697551 1chr1_63945110_63946416 68.64238468 30.75073686 106.5340325 3.464438364 1.792621489 0.221918396 1chr7_68859436_68860887 68.64238468 30.75073686 106.5340325 3.464438364 1.792621489 0.221918396 1chr7_26572322_26576122 310.7812042 139.232503 482.3299053 3.464204801 1.792524223 0.021583206 1chr8_8819737_8822625 278.350485 124.7113217 431.9896482 3.463916846 1.792404298 0.02683632 1chr8_8748246_8749822 419.4162006 187.9211697 650.9112315 3.463746168 1.79233321 0.011707668 1chr12_3401549_3405885 560.4819162 251.1310177 869.8328147 3.463661409 1.792297906 0.006400796 1chr22_38695471_38697393 423.1971469 189.629544 656.7647498 3.463409424 1.792192945 0.011497581 1chr1_47950868_47952914 152.4085544 68.3349708 236.4821381 3.460631288 1.791035238 0.078482703 1chr11_75903087_75904730 76.20427722 34.1674854 118.241069 3.460631288 1.791035238 0.198693773 1chr8_61805002_61806249 76.20427722 34.1674854 118.241069 3.460631288 1.791035238 0.198693773 1chr1_47087368_47089062 119.9778352 53.81378951 186.1418809 3.458999685 1.790354882 0.112412965 1chr12_8931737_8934666 171.3132858 76.87684216 265.7497294 3.456824213 1.789447241 0.065158036 1chr10_119003544_119004990 87.54711602 39.29260821 135.8016238 3.456162113 1.78917089 0.169767821 1chrX_149331329_149333887 218.8677901 98.23152053 339.5040596 3.456162113 1.78917089 0.042593894 1chr7_28738059_28742272 369.0931954 165.7123042 572.4740867 3.454626314 1.788529663 0.015449744 1chr2_138520483_138522078 95.10900856 42.70935675 147.5086604 3.453778553 1.788175584 0.153630224 1chr2_232794997_232796463 98.88995483 44.41773102 153.3621786 3.452724286 1.787735134 0.146345822 1chr17_17425395_17428052 260.458199 117.0236375 403.8927605 3.451377595 1.787172319 0.030975249 1chr8_54780263_54781881 106.4518474 47.83447956 165.0692152 3.450841667 1.786948281 0.133134873 1chr3_168935815_168936980 55.11639682 24.77142692 85.46136672 3.449997734 1.786595414 0.276644624 1chr2_2098703_2100752 169.1301367 76.02265502 262.2376184 3.44946672 1.786373342 0.066998331 1chr1_58041686_58044469 235.5893724 105.9192047 365.25954 3.44847321 1.785957759 0.037532151 1chr8_8628134_8633348 678.1183441 304.9448072 1051.291881 3.447482483 1.785543221 0.004465187 0.924729978chr1_64134672_64136210 125.3565787 56.37635091 194.3368065 3.447133477 1.785397162 0.106508828 1chr15_39711697_39712805 62.67828936 28.18817546 97.16840326 3.447133477 1.785397162 0.245210186 1chr5_168572067_168576136 446.3099181 200.7339767 691.8858594 3.446780015 1.785249224 0.010575716 1chr1_18909635_18910773 66.45923563 29.89654973 103.0219215 3.445946856 1.784900452 0.231374153 1chr6_42166734_42169997 343.639017 154.6078714 532.6701625 3.44529782 1.784628698 0.018104294 1chr20_24830123_24832203 218.2824382 98.23152053 338.3333559 3.444244313 1.784187482 0.043351395 1chr2_160298361_160299813 77.80207444 35.02167254 120.5824763 3.443081601 1.783700373 0.195895484 1chr3_52217584_52218939 77.80207444 35.02167254 120.5824763 3.443081601 1.783700373 0.195895484 1chr1_54717768_54721182 400.353211 180.2334855 620.4729365 3.442606321 1.783501211 0.013318521 1chr2_169459028_169461437 178.2898265 80.2935907 276.2860623 3.4409479 1.782806048 0.061864773 1chr10_44868769_44872920 274.9966323 123.8571346 426.13613 3.440545685 1.782637401 0.028284475 1chr6_107388088_107389444 92.92585951 41.85516962 143.9965494 3.440352786 1.782556512 0.159368208 1chr2_169528118_169529652 104.2686983 46.98029243 161.5571042 3.438827131 1.781916594 0.137871527 1chr15_93070207_93071802 108.0496446 48.6886667 167.4106225 3.438389955 1.781733173 0.131590609 1chr10_79083322_79088547 447.3223634 201.5881639 693.056563 3.437982418 1.781562167 0.010678397 1chr8_56888144_56889759 119.3924834 53.81378951 184.9711773 3.43724497 1.781252675 0.114938232 1chr21_33944525_33946245 134.5162685 60.64728659 208.3852504 3.436019352 1.780738162 0.096904578 1chr1_3153875_3155809 142.078161 64.06403513 220.0922869 3.435504593 1.780522012 0.089315465 1chr12_53026965_53029013 160.9828924 72.60590648 249.3598782 3.434429654 1.780070535 0.073566982 1chr14_99666252_99671420 494.2915159 222.9428422 765.6401896 3.434244319 1.779992679 0.008745525 1chr2_219884664_219890634 675.7769368 304.9448072 1046.609066 3.432126213 1.779102608 0.004635033 0.941730535chr1_53832860_53835633 263.0684416 118.7320118 407.4048715 3.431297638 1.778754274 0.031137333 1chr15_71522043_71525446 395.4015611 178.5251112 612.2780109 3.429646433 1.778059855 0.013936507 1chr1_26880521_26883590 241.9805612 109.3359533 374.6251692 3.426367612 1.776679945 0.036627659 1chr10_44451352_44452802 90.74271047 41.00098248 140.4844385 3.426367612 1.776679945 0.165381844 1chr12_89393257_89394259 60.49514031 27.33398832 93.6562923 3.426367612 1.776679945 0.256034542 1chr2_236092147_236093513 71.83797912 32.45911113 111.2168471 3.426367612 1.776679945 0.215380281 1chr2_122156203_122157469 60.49514031 27.33398832 93.6562923 3.426367612 1.776679945 0.256034542 1chr3_52344675_52346127 86.9617642 39.29260821 134.6309202 3.426367612 1.776679945 0.173987755 1chr3_52808875_52810162 83.18081793 37.58423394 128.7774019 3.426367612 1.776679945 0.183224034 1chr5_139344130_139345467 79.39987166 35.87585967 122.9238836 3.426367612 1.776679945 0.193152935 1chr6_42346637_42348892 147.4569045 66.62659653 228.2872125 3.426367612 1.776679945 0.08501547 1chr7_68293196_68294292 60.49514031 27.33398832 93.6562923 3.426367612 1.776679945 0.256034542 1chr7_111838764_111840080 86.9617642 39.29260821 134.6309202 3.426367612 1.776679945 0.173987755 1chrX_94575643_94577395 105.8664955 47.83447956 163.8985115 3.426367612 1.776679945 0.136243812 1chr9_92232312_92235515 319.1972839 144.3576258 494.0369419 3.422312739 1.774971603 0.021655583 1chr22_46824888_46828580 540.0899647 244.2975206 835.8824088 3.42157549 1.774660778 0.007453948 1chr22_48672093_48676353 438.0044154 198.1714153 677.8374155 3.420460082 1.774190393 0.011472354 1chr1_113780961_113783286 194.426057 87.98127491 300.870839 3.419714471 1.773875872 0.054518248 1chr8_141031912_141034143 175.5213256 79.43940356 271.6032477 3.41899908 1.773574034 0.064811182 1chr6_39011092_39013212 164.1784868 74.31428075 254.0426929 3.418490905 1.773359587 0.072292511 1chr1_93324641_93326032 118.8071316 53.81378951 183.8004736 3.415490255 1.772092676 0.117516297 1chr12_97975056_97976836 107.4642928 48.6886667 166.2399188 3.41434527 1.771608956 0.134643002 1chr14_40090682_40092299 103.6833465 46.98029243 160.3864006 3.413908094 1.77142422 0.141107224 1chr5_6611364_6616905 606.9762939 275.0482575 938.9043303 3.41359854 1.771293399 0.005965147 1chr12_95347214_95348897 92.34050769 41.85516962 142.8258458 3.412382438 1.770779344 0.163248731 1chr10_12462455_12465307 250.5548992 113.606889 387.5029094 3.410910315 1.770156822 0.034961194 1chr1_86107313_86108783 80.99766888 36.73004681 125.265291 3.410431019 1.769954082 0.19046491 1chr15_91998769_92000709 158.2143915 71.75171934 244.6770636 3.410051576 1.76979356 0.077246431 1chr6_90377366_90378638 77.21672261 35.02167254 119.4117727 3.409653624 1.769625188 0.200931011 1chr7_2954513_2955780 77.21672261 35.02167254 119.4117727 3.409653624 1.769625188 0.200931011 1chr10_47125494_47128889 305.0859442 138.3783159 471.7935725 3.409447278 1.769537876 0.024075174 1chr4_124417595_124419170 73.43577634 33.31329827 113.5582544 3.408796496 1.769262474 0.212214929 1chr12_132976073_132977833 139.3096601 63.20984799 215.4094723 3.407846706 1.768860441 0.094046105 1chr14_38331049_38332298 69.65483007 31.604924 107.7047361 3.407846706 1.768860441 0.22440445 1chr2_220478546_220479755 69.65483007 31.604924 107.7047361 3.407846706 1.768860441 0.22440445 1chr2_203796884_203798504 131.7477676 59.79309945 203.7024358 3.406788369 1.768412329 0.102162907 1chr5_141570778_141574293 325.5884727 147.7743744 503.4025711 3.406562019 1.768316472 0.021280241 1chr9_117227265_117230561 325.5884727 147.7743744 503.4025711 3.406562019 1.768316472 0.021280241 1chr13_93473208_93474288 62.09293753 28.18817546 95.99769961 3.405601748 1.767909736 0.251918704 1chr21_44503228_44504329 62.09293753 28.18817546 95.99769961 3.405601748 1.767909736 0.251918704 1chr10_71169945_71172588 299.1218489 135.8157545 462.4279432 3.40481813 1.767577738 0.025168827 1chr2_168639654_168640698 58.31199126 26.47980119 90.14418134 3.404262015 1.767342081 0.267494782 1chr10_125626904_125628530 105.2811437 47.83447956 162.7278079 3.401893558 1.766338001 0.139419761 1chr10_30316119_30318343 152.2502962 69.18915794 235.3114344 3.400987111 1.765953539 0.082658415 1chr8_123748345_123750614 199.2194486 90.54383632 307.8950609 3.400508234 1.765750385 0.053305656 1chr5_133899091_133901607 234.8457623 106.7733919 362.9181327 3.398956671 1.765091971 0.039921459 1chr7_68180418_68181978 93.93830491 42.70935675 145.1672531 3.398956671 1.765091971 0.161154488 1chr14_99643950_99646976 227.2838697 103.3566433 351.2110961 3.398050525 1.764707304 0.04239351 1chr11_45879069_45882020 266.6911296 121.2945732 412.0876861 3.397412393 1.76443635 0.031622876 1chr2_174478562_174480032 86.37641237 39.29260821 133.4602165 3.396573111 1.764079909 0.178303191 1chr19_18648962_18650878 125.7836723 57.23053805 194.3368065 3.395683723 1.763702091 0.110074326 1chr12_110211088_110213324 165.1909322 75.16846788 255.2133965 3.395218816 1.763504557 0.073087481 1chr6_38985415_38986902 118.2217797 53.81378951 182.62977 3.39373554 1.762874146 0.120148125 1chr20_25559421_25566167 622.9542661 283.5901288 962.3184034 3.393342382 1.762707003 0.005884724 1chr12_70632217_70633350 75.03357356 34.1674854 115.8996617 3.392103936 1.762180376 0.209116484 1chr13_39680137_39681398 75.03357356 34.1674854 115.8996617 3.392103936 1.762180376 0.209116484 1chr7_151324596_151326860 256.9460881 117.0236375 396.8685386 3.391353637 1.761861231 0.034148921 1chr12_65083895_65089159 660.178377 300.6738715 1019.682882 3.391325216 1.76184914 0.005263435 0.9889824chr12_59325848_59327940 146.2862009 66.62659653 225.9458052 3.39122538 1.761806669 0.08858205 1chr12_123483658_123486743 356.2631365 162.2955557 550.2307173 3.390300585 1.761413189 0.018227627 1chr12_124737871_124741038 372.9847188 169.9832399 575.9861976 3.388488171 1.760641735 0.016683775 1chr8_125341379_125342562 67.47168102 30.75073686 104.1926252 3.388296861 1.76056028 0.233924598 1chr1_201164377_201165756 99.3170484 45.27191816 153.3621786 3.387578545 1.760254397 0.151512178 1chr3_66834928_66837438 230.4794642 105.0650176 355.8939107 3.387368306 1.760164859 0.041839626 1chr12_67813314_67815160 127.3814695 58.08472518 196.6782138 3.386057405 1.759606432 0.108903974 1chr2_111265710_111266835 63.69073475 29.04236259 98.33910692 3.386057405 1.759606432 0.247900039 1chr22_36991546_36992757 63.69073475 29.04236259 98.33910692 3.386057405 1.759606432 0.247900039 1chr7_47330998_47334536 350.2990411 159.7329943 540.865088 3.386057405 1.759606432 0.018963036 1chrX_115088769_115090611 123.6005232 56.37635091 190.8246956 3.384835884 1.759085885 0.113713146 1chr9_91433170_91434489 91.75515586 41.85516962 141.6551421 3.384412091 1.758905244 0.167215606 1chr7_127714138_127718376 511.1236753 233.1930879 789.0542626 3.383694902 1.758599491 0.008917686 1chr19_17291298_17293034 119.819577 54.66797664 184.9711773 3.383538017 1.758532599 0.11881588 1chr3_39539543_39540635 59.90978848 27.33398832 92.48558865 3.383538017 1.758532599 0.263088492 1chr7_149186379_149188677 267.703575 122.1487603 413.2583898 3.383238509 1.758404887 0.03195127 1chr7_55196718_55198210 351.8968384 160.5871814 543.2064953 3.382626749 1.758143994 0.01888689 1chr1_61896096_61897866 116.0386307 52.95960237 179.117659 3.382156417 1.757943383 0.124235413 1chr10_77299537_77301159 116.0386307 52.95960237 179.117659 3.382156417 1.757943383 0.124235413 1chr8_8809458_8811964 260.1416825 118.7320118 401.5513532 3.381997384 1.757875544 0.033753465 1chr1_59335132_59337076 172.1674729 78.58521643 265.7497294 3.381675861 1.757738382 0.069241409 1chr2_211426849_211428706 112.2576844 51.2512281 173.2641408 3.380682711 1.75731462 0.129997311 1chr1_54350168_54351659 108.4767382 49.54285383 167.4106225 3.379107369 1.756642192 0.136129772 1chr10_64830526_64831572 52.34789595 23.91723978 80.77855211 3.377419504 1.755921385 0.297730853 1chr1_114076412_114079611 254.1775872 116.1694504 392.185724 3.375979853 1.755306295 0.035486546 1chr8_75327922_75329293 100.9148456 46.12610529 155.7035859 3.375606611 1.755146784 0.149634416 1chr12_65043683_65045540 452.2263322 206.7132867 697.7393776 3.375396854 1.755057134 0.011576079 1chr7_135117956_135119832 149.4817953 68.3349708 230.6286198 3.374972098 1.754875575 0.086896663 1chr1_111005547_111010067 509.9529716 233.1930879 786.7128553 3.373654264 1.754312132 0.009118497 1chr1_116270027_116271265 72.85042451 33.31329827 112.3875508 3.373654264 1.754312132 0.217736401 1chr13_80706642_80707989 97.13389935 44.41773102 149.8500677 3.373654264 1.754312132 0.157079501 1chr2_25242382_25243380 48.56694968 22.20886551 74.92503384 3.373654264 1.754312132 0.317570969 1chr11_125037100_125038765 117.6364279 53.81378951 181.4590663 3.371980825 1.753596332 0.122834698 1chr12_59202936_59204558 89.57200681 41.00098248 138.1430311 3.369261485 1.752432399 0.173569585 1chr1_72904435_72906664 199.6465422 91.39802345 307.8950609 3.368727783 1.752203853 0.054862026 1chr8_8295645_8302535 1066.716837 488.5950413 1644.838634 3.366466081 1.751234929 0.002261304 0.666805939chr21_44264225_44267210 404.6718279 185.3586083 623.9850475 3.366366705 1.751192341 0.014670633 1chr3_15593418_15596065 208.806232 95.66895913 321.9435048 3.365182476 1.750684737 0.050996184 1chr2_206539824_206541039 61.50758571 28.18817546 94.82699595 3.364070019 1.750207734 0.258790023 1chr3_182579500_182580585 61.50758571 28.18817546 94.82699595 3.364070019 1.750207734 0.258790023 1chr6_139663832_139666295 184.5227571 84.56452637 284.4809879 3.364070019 1.750207734 0.062882713 1chr9_80233936_80235584 102.5126428 46.98029243 158.0449933 3.364070019 1.750207734 0.147789802 1chrX_149015353_149016692 61.50758571 28.18817546 94.82699595 3.364070019 1.750207734 0.258790023 1chr5_169128729_169130718 180.7418108 82.8561521 278.6274696 3.362785533 1.749656772 0.065135411 1chr1_55329684_55336147 776.9129366 356.1960353 1197.629838 3.362277283 1.749438707 0.004070487 0.900319516chr12_67046905_67048960 176.9608646 81.14777783 272.7739513 3.361446963 1.749082387 0.067499454 1chr2_241532251_241538129 1139.994355 522.7625267 1757.226184 3.36142339 1.749072269 0.002042215 0.636342509chrX_133030737_133032162 78.229168 35.87585967 120.5824763 3.361103467 1.748934955 0.203113754 1chr6_64958584_64962813 305.3547795 140.0866901 470.6228688 3.359511659 1.748251537 0.025689342 1chr2_206530125_206531477 74.44822174 34.1674854 114.7289581 3.35784026 1.7475336 0.214510643 1chr2_158692843_158697994 493.073131 226.3595908 759.7866713 3.356547291 1.746977969 0.010050332 1chr13_73614587_73616154 91.16980403 41.85516962 140.4844385 3.356441743 1.746932602 0.171270447 1chr15_42474435_42475378 53.94569317 24.77142692 83.11995942 3.355477248 1.746517975 0.292523954 1chr8_70016925_70018010 53.94569317 24.77142692 83.11995942 3.355477248 1.746517975 0.292523954 1chr3_66316860_66318262 87.38885776 40.14679535 134.6309202 3.353466174 1.74565305 0.180234014 1chr7_2338357_2341041 208.2208801 95.66895913 320.7728011 3.352945449 1.745429011 0.051909006 1chr2_166291290_166293336 120.8320224 55.52216378 186.1418809 3.352568925 1.745266993 0.12010032 1chr1_112946295_112949370 237.8830985 109.3359533 366.4302436 3.351415821 1.744770697 0.04118449 1chr1_114995439_114997336 133.7726584 61.50147372 206.0438431 3.35022611 1.744258468 0.104396302 1chr11_134274985_134281973 711.4661463 327.1536727 1095.77862 3.349430898 1.743915988 0.004946945 0.965381333chr10_74387893_74390866 230.3212059 105.9192047 354.7232071 3.348998021 1.743729524 0.043738075 1chr20_18595790_18597683 129.9917121 59.79309945 200.1903248 3.348050638 1.743321348 0.108909985 1chr1_54044764_54049159 339.8103895 156.3162457 523.3045332 3.347729667 1.743183033 0.021261799 1chr5_146418694_146421064 142.9323482 65.7724094 220.0922869 3.346270707 1.742554161 0.095142982 1chr4_4613195_4615107 189.3161488 87.12708778 291.5052098 3.345747198 1.742328441 0.061373445 1chr22_42297975_42300139 181.7542562 83.71033924 279.7981732 3.342456569 1.740908814 0.065820838 1chr2_231531006_231533068 135.3704556 62.35566086 208.3852504 3.341881836 1.740660723 0.103311382 1chr4_71481574_71483148 88.98665499 41.00098248 136.9723275 3.340708422 1.740154069 0.177800711 1chr7_68703274_68704509 72.26507269 33.31329827 111.2168471 3.338512032 1.73920524 0.223386725 1chr10_73815254_73818141 187.1329997 86.27290064 287.9930988 3.33816409 1.739054873 0.063026487 1chr19_13079785_13081298 127.8085631 58.93891232 196.6782138 3.336984109 1.738544816 0.112461898 1chr11_121416198_121418770 251.8361799 116.1694504 387.5029094 3.335669646 1.737976416 0.037871582 1chr14_35361907_35363042 55.54349039 25.62561405 85.46136672 3.334997809 1.737685814 0.287455566 1chr2_101449189_101450258 55.54349039 25.62561405 85.46136672 3.334997809 1.737685814 0.287455566 1chr6_122997141_123002380 397.9641225 183.650234 612.2780109 3.33393537 1.737226137 0.015951564 1chr11_75416814_75418085 68.48412642 31.604924 105.3633288 3.333763082 1.737151581 0.236399306 1chr9_125285811_125287091 68.48412642 31.604924 105.3633288 3.333763082 1.737151581 0.236399306 1chr5_78596699_78598056 81.42476245 37.58423394 125.265291 3.332921223 1.736787218 0.197359242 1chr3_72748284_72752560 321.9181035 148.6285615 495.2076455 3.331847126 1.736322208 0.024121264 1chr4_187822927_187824796 120.2466705 55.52216378 184.9711773 3.331483586 1.736164786 0.122749301 1chr12_2876603_2879970 253.4339771 117.0236375 389.8443167 3.331329679 1.736098135 0.037637253 1chr18_52382613_52384732 133.1873066 61.50147372 204.8731394 3.331190734 1.736037961 0.106617363 1chr12_3299511_3301485 184.9498507 85.41871351 284.4809879 3.330429319 1.735708164 0.064735688 1chr2_152923690_152926765 197.8904867 91.39802345 304.38295 3.330301231 1.735652677 0.057919635 1chr14_33225105_33226393 77.64381618 35.87585967 119.4117727 3.328471395 1.73485977 0.208266275 1chr17_61346959_61348012 51.76254412 23.91723978 79.60784846 3.328471395 1.73485977 0.306206451 1chr8_70752363_70753826 90.58445221 41.85516962 139.3137348 3.328471395 1.73485977 0.175414887 1chr11_75545038_75546725 138.5660501 64.06403513 213.068065 3.325860829 1.7337278 0.101189968 1chr8_124064728_124066470 138.5660501 64.06403513 213.068065 3.325860829 1.7337278 0.101189968 1chr2_128371916_128378368 740.8118481 342.5290412 1139.094655 3.325541832 1.733589419 0.004811614 0.956928541chr3_63546636_63548843 112.684778 52.10541524 173.2641408 3.325261683 1.733467879 0.134359692 1chr2_190756879_190758550 99.74414197 46.12610529 153.3621786 3.324845609 1.73328735 0.156742832 1chr2_206755822_206759105 299.2324259 138.3783159 460.0865359 3.324845609 1.73328735 0.027955761 1chr2_99496731_99502901 729.4690093 337.4039183 1121.5341 3.324010301 1.732924853 0.004969903 0.965381333chr19_18860135_18862870 278.7298973 128.9822574 428.4775373 3.321988202 1.732046949 0.032012902 1chr6_136695464_136697145 108.9038317 50.39704097 167.4106225 3.321834363 1.731980138 0.140729681 1chr8_129287633_129290737 313.7708592 145.211813 482.3299053 3.321561074 1.731861441 0.025683956 1chr10_70353277_70357718 309.9899129 143.5034387 476.4763871 3.320313377 1.731319412 0.026322305 1chr1_116171827_116173806 118.0635215 54.66797664 181.4590663 3.319293624 1.730876256 0.126902062 1chr12_110116074_110121193 516.4547375 239.1723978 793.7370772 3.318681773 1.730610297 0.009800957 1chr19_5772929_5778261 499.7331552 231.4847136 767.9815969 3.317634175 1.730154814 0.010474462 1chr17_74673520_74675392 162.2641731 75.16846788 249.3598782 3.317346825 1.730029852 0.080599265 1chr7_90157206_90159102 149.323537 69.18915794 229.4579161 3.316385442 1.729611692 0.091440554 1chr1_107315229_107316714 114.2825752 52.95960237 175.6055481 3.315839625 1.729374231 0.132782918 1chr2_191296880_191298156 57.14128761 26.47980119 87.80277403 3.315839625 1.729374231 0.282520778 1chr7_75907715_75909311 114.2825752 52.95960237 175.6055481 3.315839625 1.729374231 0.132782918 1chrX_67538456_67539506 57.14128761 26.47980119 87.80277403 3.315839625 1.729374231 0.282520778 1chr8_125306855_125308978 193.5241886 89.68964918 297.3587281 3.315418566 1.72919102 0.061017018 1chr2_148719934_148721715 136.382901 63.20984799 209.555954 3.315242176 1.729114262 0.104394053 1chr3_142366476_142369873 281.9254918 130.6906317 433.1603519 3.314394814 1.728745468 0.031654667 1chr14_40141146_40142734 123.442265 57.23053805 189.6539919 3.313860019 1.728512663 0.120012807 1chr1_58752810_58759965 657.6310302 304.9448072 1010.317253 3.313115125 1.728188336 0.006191328 1chr2_151727160_151732325 322.3451971 149.4827486 495.2076455 3.312808 1.728054592 0.024705691 1chr3_58283498_58285233 132.6019547 61.50147372 203.7024358 3.312155359 1.727770345 0.108882559 1chr6_41557718_41560805 627.9688119 291.2778131 964.6598107 3.311820425 1.727624449 0.006783156 1chr11_87903797_87907706 296.463925 137.5241287 455.4037213 3.311445966 1.727461317 0.028955414 1chr1_113210367_113212151 141.7616445 65.7724094 217.7508796 3.310672083 1.727124121 0.099131294 1chr3_28897382_28898802 75.46066713 35.02167254 115.8996617 3.309369694 1.726556466 0.216746975 1chr6_5744339_5745712 75.46066713 35.02167254 115.8996617 3.309369694 1.726556466 0.216746975 1chr4_155480418_155482224 128.8210085 59.79309945 197.8489175 3.308892151 1.72634827 0.113637003 1chr4_82462158_82463233 53.36034134 24.77142692 81.94925576 3.308217005 1.726053872 0.300759971 1chr20_2264993_2266518 115.8803724 53.81378951 177.9469554 3.30671668 1.72539944 0.131232878 1chr7_55115686_55116842 231.7607449 107.627579 355.8939107 3.30671668 1.72539944 0.045387677 1chr4_120635870_120637902 147.140388 68.3349708 225.9458052 3.306444746 1.725280793 0.094233133 1chr1_85593234_85594858 125.0400622 58.08472518 191.9953992 3.305436991 1.724841014 0.118677693 1chr5_77493519_77494773 62.5200311 29.04236259 95.99769961 3.305436991 1.724841014 0.261452308 1chr17_9963907_9965446 112.0994262 52.10541524 172.0934371 3.302793698 1.723686858 0.137381681 1chr5_107573998_107576214 152.5191315 70.89753221 234.1407308 3.302523 1.723568609 0.08966778 1chr10_70624181_70625545 80.83941062 37.58423394 124.0945873 3.301772426 1.723240686 0.202286071 1chr2_45427280_45428580 80.83941062 37.58423394 124.0945873 3.301772426 1.723240686 0.202286071 1chr8_37691082_37692699 121.2591159 56.37635091 186.1418809 3.301772426 1.723240686 0.124026891 1chrX_67877704_67879970 130.4188057 60.64728659 200.1903248 3.300894995 1.722857246 0.112405137 1chr5_133261057_133263508 179.9982008 83.71033924 276.2860623 3.300501047 1.722685056 0.069621215 1chr1_60583646_60584626 49.57939507 23.06305265 76.09573749 3.299465108 1.722232161 0.32075337 1chr9_137852191_137855432 374.5348348 174.2541756 574.815494 3.298718623 1.721905723 0.018943944 1chr21_47807184_47809323 225.7966496 105.0650176 346.5282815 3.298227035 1.721690711 0.047940005 1chr12_105317341_105318626 58.73908483 27.33398832 90.14418134 3.297878827 1.721538391 0.277714875 1chr3_23042166_23045900 398.2329578 185.3586083 611.1073073 3.29689197 1.721106615 0.016881116 1chr12_3700153_3701287 67.89877459 31.604924 104.1926252 3.29672127 1.721031916 0.242609117 1chr12_70608364_70609673 67.89877459 31.604924 104.1926252 3.29672127 1.721031916 0.242609117 1chr7_9243640_9244868 67.89877459 31.604924 104.1926252 3.29672127 1.721031916 0.242609117 1chr11_68011230_68012795 144.9572389 67.48078367 222.4336942 3.296252387 1.720826711 0.097133216 1chr6_64965964_64970677 394.4520115 183.650234 605.253789 3.295687545 1.720579471 0.017227024 1chr2_149054944_149056264 86.21815411 40.14679535 132.2895129 3.295145023 1.720341961 0.189166004 1chr5_31944222_31945687 372.3516857 173.3999884 571.303383 3.294714078 1.720153271 0.019274116 1chr4_139144150_139145636 95.37784387 44.41773102 146.3379567 3.294584243 1.720096417 0.168531321 1chr5_53946767_53948114 95.37784387 44.41773102 146.3379567 3.294584243 1.720096417 0.168531321 1chr9_111425982_111427661 95.37784387 44.41773102 146.3379567 3.294584243 1.720096417 0.168531321 1chrX_118196157_118197784 95.37784387 44.41773102 146.3379567 3.294584243 1.720096417 0.168531321 1chr8_11719489_11725150 790.5018202 368.1546552 1212.848985 3.294400785 1.720016079 0.004553596 0.933005102chr1_114022481_114023829 104.5375336 48.6886667 160.3864006 3.294121845 1.719893919 0.1509102 1chr10_120615556_120617597 132.0166029 61.50147372 202.5317321 3.293119983 1.719455077 0.111192666 1chr12_65732161_65734124 150.3359824 70.04334507 230.6286198 3.292655705 1.719251665 0.092379088 1chr10_70959555_70963650 306.0507084 142.6492516 469.4521652 3.290954281 1.718505984 0.028054694 1chr12_110202840_110203742 155.7147259 72.60590648 238.8235454 3.289312908 1.717786256 0.087945017 1chr14_31864646_31866028 109.9162771 51.2512281 168.5813261 3.289312908 1.717786256 0.142182529 1chr3_59711277_59713374 155.7147259 72.60590648 238.8235454 3.289312908 1.717786256 0.087945017 1chr4_84475709_84477364 100.7565874 46.98029243 154.5328823 3.289312908 1.717786256 0.158361304 1chr5_168375389_168376568 73.27751808 34.1674854 112.3875508 3.289312908 1.717786256 0.225676073 1chr1_86742455_86744837 234.3709875 109.3359533 359.4060217 3.287171428 1.716846696 0.04552222 1chr12_3211732_3217559 561.9366698 262.2354505 861.6378892 3.28574145 1.716218961 0.008832043 1chr10_125764945_125766858 133.6144001 62.35566086 204.8731394 3.285557984 1.716138403 0.10999923 1chr12_51532058_51533848 133.6144001 62.35566086 204.8731394 3.285557984 1.716138403 0.10999923 1chr12_57152501_57154503 133.6144001 62.35566086 204.8731394 3.285557984 1.716138403 0.10999923 1chr20_36508762_36511123 267.2288003 124.7113217 409.7462788 3.285557984 1.716138403 0.036188569 1chr3_194339308_194343213 479.499462 223.7970294 735.2018946 3.285128031 1.715949598 0.012014991 1chr8_11547840_11551406 479.499462 223.7970294 735.2018946 3.285128031 1.715949598 0.012014991 1chr12_105055468_105057274 106.1353309 49.54285383 162.7278079 3.284586883 1.715711928 0.149036306 1chr15_86042511_86043852 87.81595133 41.00098248 134.6309202 3.283602295 1.715279401 0.186545999 1chr3_71412372_71413585 78.65626157 36.73004681 120.5824763 3.28293827 1.714987623 0.210395971 1chr8_9587003_9588242 78.65626157 36.73004681 120.5824763 3.28293827 1.714987623 0.210395971 1chrX_149150304_149151637 78.65626157 36.73004681 120.5824763 3.28293827 1.714987623 0.210395971 1chr14_39578211_39580179 138.9931436 64.91822226 213.068065 3.282099502 1.714618977 0.104362968 1chr9_123721456_123722943 69.49657181 32.45911113 106.5340325 3.282099502 1.714618977 0.238801695 1chr1_93718150_93719266 60.33688205 28.18817546 92.48558865 3.281006562 1.714138479 0.273033331 1chr1_213554665_213555768 60.33688205 28.18817546 92.48558865 3.281006562 1.714138479 0.273033331 1chr21_20698515_20699628 60.33688205 28.18817546 92.48558865 3.281006562 1.714138479 0.273033331 1chr21_28156670_28157821 60.33688205 28.18817546 92.48558865 3.281006562 1.714138479 0.273033331 1chr7_28065502_28066638 60.33688205 28.18817546 92.48558865 3.281006562 1.714138479 0.273033331 1chr7_131473969_131475090 60.33688205 28.18817546 92.48558865 3.281006562 1.714138479 0.273033331 1chr3_46505797_46509229 265.0456512 123.8571346 406.2341679 3.279860859 1.713634613 0.036985967 1chr1_68239733_68240783 51.17719229 23.91723978 78.4371448 3.279523286 1.713486119 0.31489233 1chr3_179486974_179488489 102.3543846 47.83447956 156.8742896 3.279523286 1.713486119 0.156348673 1chr10_12581454_12582085 93.19469482 43.56354389 142.8258458 3.278563519 1.713063846 0.174846499 1chr7_16629287_16631076 135.2121974 63.20984799 207.2145467 3.278200364 1.712904035 0.108824472 1chr12_65586524_65587844 261.2647049 122.1487603 400.3806496 3.277811814 1.712733029 0.038041765 1chr1_59314223_59315659 84.03500506 39.29260821 128.7774019 3.277395107 1.712549608 0.196556218 1chr8_10694109_10696845 252.1050152 117.8778246 386.3322057 3.277395107 1.712549608 0.040542221 1chr15_75099045_75102685 401.8556458 187.9211697 615.7901219 3.276853389 1.712311126 0.01712182 1chr2_232855413_232857593 200.9278229 93.96058486 307.8950609 3.276853389 1.712311126 0.059689844 1chr20_20050896_20052123 74.8753153 35.02167254 114.7289581 3.275941717 1.71190969 0.222262826 1chr14_34711952_34713464 107.7331281 50.39704097 165.0692152 3.275375141 1.711660153 0.147196288 1chr6_137544314_137546139 140.5909408 65.7724094 215.4094723 3.275073458 1.711527266 0.103276065 1chr8_8263098_8270856 1051.450009 492.0117898 1610.888228 3.274084607 1.711091604 0.002884759 0.740026776chr12_92489736_92491009 65.71562554 30.75073686 100.6805142 3.274084607 1.711091604 0.253024198 1chr21_46077232_46078443 65.71562554 30.75073686 100.6805142 3.274084607 1.711091604 0.253024198 1chr9_74919879_74921230 65.71562554 30.75073686 100.6805142 3.274084607 1.711091604 0.253024198 1chr1_112158119_112159541 89.41374855 41.85516962 136.9723275 3.272530699 1.710406726 0.183979189 1chr12_7143759_7145191 89.41374855 41.85516962 136.9723275 3.272530699 1.710406726 0.183979189 1chr1_167624187_167626417 169.6678073 79.43940356 259.8962111 3.27162843 1.710008906 0.078478533 1chr2_11313368_11314398 56.55593578 26.47980119 86.63207038 3.27162843 1.710008906 0.290309106 1chr6_168429837_168430989 56.55593578 26.47980119 86.63207038 3.27162843 1.710008906 0.290309106 1chr8_116166487_116167629 56.55593578 26.47980119 86.63207038 3.27162843 1.710008906 0.290309106 1chr22_29812938_29814370 80.25405879 37.58423394 122.9238836 3.27062363 1.709565749 0.207323772 1chr12_15863614_15866392 184.2062406 86.27290064 282.1395806 3.270315226 1.709429704 0.069121792 1chr19_2220034_2222325 142.1887381 66.62659653 217.7508796 3.268227569 1.708508443 0.102205734 1chr6_39949040_39950020 47.39624602 22.20886551 72.58362653 3.268227569 1.708508443 0.336237925 1chr8_24086320_24087255 47.39624602 22.20886551 72.58362653 3.268227569 1.708508443 0.336237925 1chr8_24151268_24152224 71.09436903 33.31329827 108.8754398 3.268227569 1.708508443 0.235083571 1chr10_47097250_47099008 180.4252943 84.56452637 276.2860623 3.267162653 1.70803828 0.071640523 1chr6_41690096_41691564 233.2002838 109.3359533 357.0646144 3.26575663 1.707417283 0.047063643 1chr1_103715625_103716655 61.93467927 29.04236259 94.82699595 3.265126783 1.707139012 0.268471808 1chr7_27468810_27470223 123.8693585 58.08472518 189.6539919 3.265126783 1.707139012 0.123865285 1chr6_39049919_39053182 224.0405941 105.0650176 343.0161705 3.264799058 1.7069942 0.050441489 1chr8_129391133_129392444 100.1712355 46.98029243 153.3621786 3.264393871 1.706815138 0.162036533 1chr10_123611040_123612067 52.77498951 24.77142692 80.77855211 3.260956762 1.705295312 0.309198423 1chr12_96824275_96825650 105.549979 49.54285383 161.5571042 3.260956762 1.705295312 0.152434473 1chrX_118819416_118820930 105.549979 49.54285383 161.5571042 3.260956762 1.705295312 0.152434473 1chr1_116721056_116722570 120.0884123 56.37635091 183.8004736 3.260240698 1.70497848 0.129503727 1chr8_10785295_10786905 120.0884123 56.37635091 183.8004736 3.260240698 1.70497848 0.129503727 1chr12_105296702_105298026 67.31342276 31.604924 103.0219215 3.259679458 1.704730103 0.248964017 1chr13_106880006_106881397 67.31342276 31.604924 103.0219215 3.259679458 1.704730103 0.248964017 1chr18_59402356_59403545 67.31342276 31.604924 103.0219215 3.259679458 1.704730103 0.248964017 1chr2_29494141_29495300 67.31342276 31.604924 103.0219215 3.259679458 1.704730103 0.248964017 1chr4_187642709_187643774 67.31342276 31.604924 103.0219215 3.259679458 1.704730103 0.248964017 1chr6_48355507_48357854 134.6268455 63.20984799 206.0438431 3.259679458 1.704730103 0.111105016 1chr1_61712545_61714652 216.4787015 101.6482691 331.309134 3.259368182 1.70459233 0.05376118 1chr18_48408945_48410355 81.85185601 38.43842108 125.265291 3.258856307 1.704365741 0.204317905 1chr7_140028858_140030851 163.703712 76.87684216 250.5305819 3.258856307 1.704365741 0.083846182 1chr1_109906767_109909285 198.159322 93.10639772 303.2122463 3.25662096 1.703375813 0.062354087 1chr11_66368945_66371218 256.313055 120.440386 392.185724 3.256264256 1.703217783 0.040405401 1chr1_243574133_243575707 101.7690328 47.83447956 155.7035859 3.255049232 1.702679364 0.159953312 1chr15_29738077_29739310 72.69216625 34.1674854 111.2168471 3.255049232 1.702679364 0.231452007 1chr18_55344476_55345838 72.69216625 34.1674854 111.2168471 3.255049232 1.702679364 0.231452007 1chr7_94176429_94177633 58.153733 27.33398832 88.97347769 3.255049232 1.702679364 0.285293733 1chr6_42593948_42595980 165.3015093 77.73102929 252.8719892 3.253166612 1.701844712 0.083060511 1chr16_4886084_4887999 150.763076 70.89753221 230.6286198 3.252985155 1.701764239 0.095183763 1chr9_16242977_16245037 150.763076 70.89753221 230.6286198 3.252985155 1.701764239 0.095183763 1chr5_28194565_28196311 121.6862095 57.23053805 186.1418809 3.252492241 1.701545615 0.128007897 1chr6_157636249_157639747 306.9048955 144.3576258 469.4521652 3.252008077 1.701330841 0.029430172 1chr3_46620403_46622151 92.609343 43.56354389 141.6551421 3.251690048 1.701189746 0.178999791 1chr7_50660424_50662653 185.218686 87.12708778 283.3102842 3.251690048 1.701189746 0.069801272 1chr6_131894821_131896050 78.07090975 36.73004681 119.4117727 3.251065083 1.700912438 0.215664163 1chr16_68101690_68102813 63.53247649 29.89654973 97.16840326 3.250154421 1.700508265 0.264026148 1chr2_30071098_30072283 63.53247649 29.89654973 97.16840326 3.250154421 1.700508265 0.264026148 1chr2_74062142_74064380 239.5914727 112.7527018 366.4302436 3.249857766 1.700376578 0.045770985 1chr1_108468505_108470047 112.5265197 52.95960237 172.0934371 3.249522832 1.700227885 0.141872103 1chr12_89531108_89532666 97.98808649 46.12610529 149.8500677 3.248704106 1.699864348 0.167987799 1chr7_51714752_51716485 244.9702162 115.3152632 374.6251692 3.248704106 1.699864348 0.044115872 1chr5_159352867_159354505 83.44965324 39.29260821 127.6066983 3.247600606 1.699374219 0.201376536 1chr1_65612419_65614903 152.3608732 71.75171934 232.9700271 3.246891214 1.699059048 0.094235084 1chr17_71415865_71418359 186.8164832 87.98127491 285.6516915 3.246732805 1.698988661 0.069203938 1chr1_92823742_92824888 68.91121999 32.45911113 105.3633288 3.246032475 1.698677433 0.245001993 1chr3_64170490_64175595 398.9288867 187.9211697 609.9366036 3.245704593 1.698531699 0.018238397 1chr8_99414648_99416573 123.2840067 58.08472518 188.4832883 3.24497168 1.698205887 0.126537375 1chr10_3903750_3904694 54.37278673 25.62561405 83.11995942 3.243628006 1.697608374 0.303664927 1chr1_87288731_87294586 603.4793976 284.444316 922.5144792 3.243216431 1.697425303 0.008360121 1chr1_60926170_60929434 257.3255004 121.2945732 393.3564277 3.242984557 1.697322154 0.04078728 1chr4_71318224_71320330 168.4971037 79.43940356 257.5548038 3.2421543 1.696952753 0.081521753 1chr9_132911605_132913582 114.124317 53.81378951 174.4348444 3.241452535 1.696640447 0.140160286 1chr6_170707475_170709527 134.0414937 63.20984799 204.8731394 3.241158552 1.696509597 0.113430119 1chr13_31446247_31447547 79.66870697 37.58423394 121.75318 3.239474833 1.69575995 0.212474368 1chr2_208160858_208162264 99.58588371 46.98029243 152.191475 3.239474833 1.69575995 0.165789738 1chr21_38704130_38705241 59.75153022 28.18817546 91.31488499 3.239474833 1.69575995 0.280411497 1chr3_59197768_59198985 79.66870697 37.58423394 121.75318 3.239474833 1.69575995 0.212474368 1chr4_159811267_159812375 59.75153022 28.18817546 91.31488499 3.239474833 1.69575995 0.280411497 1chr6_144093792_144097103 219.0889442 103.3566433 334.821245 3.239474833 1.69575995 0.053870909 1chr7_107366313_107368158 124.8818039 58.93891232 190.8246956 3.237669106 1.694955548 0.125091636 1chr7_128323469_128325946 190.0120777 89.68964918 290.3345061 3.237101592 1.694702643 0.068032167 1chr2_220347371_220348775 85.04745046 40.14679535 129.9481056 3.236823872 1.694578865 0.19849789 1chr5_133694305_133695806 85.04745046 40.14679535 129.9481056 3.236823872 1.694578865 0.19849789 1chr9_15888614_15890122 85.04745046 40.14679535 129.9481056 3.236823872 1.694578865 0.19849789 1chr8_18702858_18705046 150.1777242 70.89753221 229.4579161 3.23647254 1.694422263 0.097092252 1chrX_135646159_135648248 150.1777242 70.89753221 229.4579161 3.23647254 1.694422263 0.097092252 1chr18_55588513_55590773 135.6392909 64.06403513 207.2145467 3.234491026 1.69353871 0.112200064 1chr19_18310774_18312305 135.6392909 64.06403513 207.2145467 3.234491026 1.69353871 0.112200064 1chr8_128017511_128018914 70.50901721 33.31329827 107.7047361 3.233085337 1.692911588 0.241134965 1chr5_142007025_142008103 50.59184046 23.91723978 77.26644115 3.230575177 1.691791048 0.323792311 1chr14_22009938_22013474 502.1374584 237.4640235 766.8108932 3.229166599 1.691161874 0.012136236 1chr12_65925767_65928497 294.391353 139.232503 449.550203 3.228773406 1.690986197 0.032741886 1chr3_131831433_131834082 187.8289286 88.83546205 286.8223952 3.228692558 1.690950071 0.069871758 1chr1_84435490_84437053 81.26650419 38.43842108 124.0945873 3.228399706 1.690819209 0.209354798 1chr5_173734148_173735919 167.9117519 79.43940356 256.3841002 3.227417235 1.690380099 0.083084901 1chr6_91463041_91464635 111.9411679 52.95960237 170.9227334 3.227417235 1.690380099 0.145027248 1chrX_21690653_21691723 55.97058396 26.47980119 85.46136672 3.227417235 1.690380099 0.298285472 1chr1_53855502_53857932 198.5864156 93.96058486 303.2122463 3.227015315 1.690200425 0.064084799 1chr15_61478868_61480729 86.64524768 41.00098248 132.2895129 3.226496168 1.689968312 0.195680246 1chr22_43280293_43285728 644.1679382 304.9448072 983.3910692 3.224816576 1.689217104 0.007675721 1chr7_155609820_155612417 337.421301 159.7329943 515.1096077 3.224816576 1.689217104 0.025710885 1chr5_159577215_159578889 97.40273466 46.12610529 148.679364 3.223323606 1.688549035 0.171897062 1chr12_6532560_6533649 66.72807094 31.604924 101.8512179 3.222637646 1.688241981 0.255466654 1chr21_42859494_42861197 138.8348854 65.7724094 211.8973613 3.221675521 1.687811197 0.109798511 1chr3_66499743_66502768 632.8250994 299.8196844 965.8305143 3.221371259 1.687674939 0.007986584 1chr3_66699767_66701069 252.3738505 119.5861989 385.1615021 3.220785556 1.687412607 0.043345875 1chr6_55338762_55340064 72.10681443 34.1674854 110.0461435 3.220785556 1.687412607 0.237359894 1chr7_45154715_45157418 304.5634882 144.3576258 464.7693505 3.219569094 1.686867611 0.03119318 1chr11_68694083_68696263 160.3498593 76.02265502 244.6770636 3.218475645 1.686377552 0.089696302 1chr4_157478945_157480354 82.86430141 39.29260821 126.4359946 3.217806105 1.686077397 0.206303439 1chr11_60818365_60820872 212.539497 100.7940819 324.2849121 3.217301114 1.685850967 0.058031827 1chr10_62788207_62790056 93.62178839 44.41773102 142.8258458 3.215514221 1.68504947 0.180741441 1chr6_76753091_76754023 46.8108942 22.20886551 71.41292288 3.215514221 1.68504947 0.345923025 1chr22_37819564_37823094 343.8124898 163.1497428 524.4752369 3.214686262 1.684677944 0.025212023 1chr5_169384075_169385431 99.00053188 46.98029243 151.0207713 3.214555796 1.684619392 0.16962231 1chr3_42559771_42562096 203.3798073 96.52314626 310.2364682 3.214114751 1.684421437 0.062528921 1chr8_37113984_37116201 203.3798073 96.52314626 310.2364682 3.214114751 1.684421437 0.062528921 1chr9_96959505_96961672 203.3798073 96.52314626 310.2364682 3.214114751 1.684421437 0.062528921 1chr6_12559672_12561391 104.3792754 49.54285383 159.2156969 3.213696519 1.684233697 0.159445318 1chrX_69729008_69730504 104.3792754 49.54285383 159.2156969 3.213696519 1.684233697 0.159445318 1chr9_141025881_141030789 422.895845 200.7339767 645.0577132 3.213495412 1.684143412 0.017216398 1chr10_80670555_80674880 543.4113508 257.9645148 828.8581869 3.213070556 1.683952661 0.010773538 1chr1_68097725_68100234 219.5160377 104.2108305 334.821245 3.212921761 1.68388585 0.055311635 1chr2_196937455_196939141 109.7580189 52.10541524 167.4106225 3.212921761 1.68388585 0.15010799 1chr19_47859367_47861912 230.2735247 109.3359533 351.2110961 3.212219637 1.683570541 0.051140165 1chr3_71318583_71319671 57.56838118 27.33398832 87.80277403 3.212219637 1.683570541 0.293054013 1chr16_20806488_20808575 120.5155058 57.23053805 183.8004736 3.211580389 1.683283409 0.133609502 1chr7_68543629_68546518 188.841374 89.68964918 287.9930988 3.210995934 1.683020838 0.070536649 1chr2_201587177_201588219 68.32586816 32.45911113 104.1926252 3.209965447 1.682557768 0.251345349 1chr5_173446500_173447759 68.32586816 32.45911113 104.1926252 3.209965447 1.682557768 0.251345349 1chr8_128030056_128031867 204.9776045 97.3773334 312.5778756 3.209965447 1.682557768 0.062023086 1chr17_43944479_43949159 420.7126959 199.8797896 641.5456023 3.209657182 1.682419214 0.017493119 1chr7_4212376_4216396 636.4477874 302.3822458 970.513329 3.20955791 1.682374592 0.008090626 1chr14_40721918_40723507 152.7879668 72.60590648 232.9700271 3.208692493 1.681985535 0.097039495 1chr13_28691661_28692882 79.08335514 37.58423394 120.5824763 3.208326037 1.681820759 0.217739902 1chr2_235236807_235238392 84.46209863 40.14679535 128.7774019 3.207663297 1.681522712 0.203318346 1chr5_118762633_118764125 84.46209863 40.14679535 128.7774019 3.207663297 1.681522712 0.203318346 1chr7_106751164_106752250 89.84084212 42.70935675 136.9723275 3.207080085 1.68126038 0.19022135 1chr4_187648230_187652759 393.2336266 187.0669826 599.4002707 3.20420131 1.679964791 0.020006195 1chr12_89431177_89433223 149.0070205 70.89753221 227.1165088 3.20344731 1.679625261 0.101017517 1chr14_33441155_33443103 175.900738 83.71033924 268.0911367 3.202604829 1.679245794 0.079315949 1chr19_18498323_18500328 186.658225 88.83546205 284.4809879 3.202335884 1.679124636 0.072452768 1chr7_30836430_30841027 570.7321618 271.6315089 869.8328147 3.202253001 1.679087296 0.010035485 1chr8_11427436_11432625 1854.654059 883.2294976 2826.07862 3.199710412 1.677941341 0.001523133 0.56153351chr1_3187055_3190288 328.1033529 156.3162457 499.8904602 3.197943105 1.677144272 0.028120735 1chr1_57245756_57246768 53.78743491 25.62561405 81.94925576 3.197943105 1.677144272 0.312065673 1chr1_85967777_85968809 53.78743491 25.62561405 81.94925576 3.197943105 1.677144272 0.312065673 1chr12_96513389_96515610 145.2260742 69.18915794 221.2629906 3.197943105 1.677144272 0.105219099 1chr16_20836480_20838033 96.81738283 46.12610529 147.5086604 3.197943105 1.677144272 0.175889196 1chr2_149914699_149918316 322.7246094 153.7536843 491.6955346 3.197943105 1.677144272 0.028969868 1chr2_201519308_201520478 69.92366538 33.31329827 106.5340325 3.197943105 1.677144272 0.247324954 1chr8_57699611_57700593 48.40869142 23.06305265 73.75433019 3.197943105 1.677144272 0.339315776 1chrX_134052642_134053741 53.78743491 25.62561405 81.94925576 3.197943105 1.677144272 0.312065673 1chr1_167511736_167515205 351.2161241 167.4206785 535.0115698 3.195612242 1.676092361 0.024943841 1chr2_47513584_47516733 227.5050238 108.4817662 346.5282815 3.194345868 1.675520529 0.053260179 1chr5_54487067_54490146 222.1262803 105.9192047 338.3333559 3.194258839 1.675481223 0.055400154 1chr13_110131414_110133940 195.2325629 93.10639772 297.3587281 3.193751829 1.675252212 0.068157548 1chr5_42452920_42455772 342.0564344 163.1497428 520.9631259 3.193159345 1.674984548 0.026264244 1chr12_107882764_107889634 907.8369463 433.0728775 1382.601015 3.192536608 1.674703163 0.004470551 0.924729978chr20_5899053_5901186 141.445128 67.48078367 215.4094723 3.192160206 1.674533058 0.109660279 1chr14_23118808_23123040 517.3718205 246.860082 787.883559 3.191619935 1.674288863 0.012278808 1chr3_15428444_15430232 103.7939235 49.54285383 158.0449933 3.190066398 1.673586452 0.163060531 1chr1_247680912_247682304 82.27894958 39.29260821 125.265291 3.188011604 1.672656881 0.211338841 1chr12_46503320_46504575 82.27894958 39.29260821 125.265291 3.188011604 1.672656881 0.211338841 1chr4_115563891_115565518 82.27894958 39.29260821 125.265291 3.188011604 1.672656881 0.211338841 1chr2_160413537_160414899 76.90020609 36.73004681 117.0703654 3.187318709 1.672343286 0.226557776 1chr3_16488855_16492500 307.6008244 146.9201872 468.2814615 3.187318709 1.672343286 0.032034403 1chr7_55113804_55115665 532.9226991 254.5477662 811.2976321 3.187211752 1.672294872 0.011718099 1chr12_66323367_66324620 71.5214626 34.1674854 108.8754398 3.186521879 1.671982567 0.243402116 1chr2_138894885_138896148 66.14271911 31.604924 100.6805142 3.185595834 1.67156324 0.262119693 1chr12_133206743_133209406 243.0559025 116.1694504 369.9423546 3.184506369 1.671069757 0.048273982 1chr2_205641443_205642692 60.76397562 29.04236259 92.48558865 3.184506369 1.671069757 0.28301244 1chrX_4704575_4707257 182.2919269 87.12708778 277.4567659 3.184506369 1.671069757 0.076483352 1chr1_81013597_81015255 116.1492077 55.52216378 176.7762517 3.183886212 1.670788777 0.142865075 1chr1_113206174_113207718 110.7704643 52.95960237 168.5813261 3.18320604 1.670480542 0.151532325 1chr2_6054824_6056742 166.1556964 79.43940356 252.8719892 3.18320604 1.670480542 0.087945983 1chr3_58173147_58175338 166.1556964 79.43940356 252.8719892 3.18320604 1.670480542 0.087945983 1chr6_82694564_82695606 55.38523213 26.47980119 84.29066307 3.18320604 1.670480542 0.306453256 1chr8_116175817_116176903 55.38523213 26.47980119 84.29066307 3.18320604 1.670480542 0.306453256 1chr9_137874500_137875620 55.38523213 26.47980119 84.29066307 3.18320604 1.670480542 0.306453256 1chr1_47064203_47069721 537.7160908 257.1103277 818.321854 3.182765397 1.670280819 0.011621202 1chr21_44161296_44163279 105.3917208 50.39704097 160.3864006 3.182456698 1.670140884 0.160957169 1chr3_53100051_53102745 210.7834415 100.7940819 320.7728011 3.182456698 1.670140884 0.061101068 1chr9_109899506_109901402 105.3917208 50.39704097 160.3864006 3.182456698 1.670140884 0.160957169 1chr2_230954178_230955784 100.0129773 47.83447956 152.191475 3.181627069 1.669764741 0.171228843 1chr3_14759944_14761476 100.0129773 47.83447956 152.191475 3.181627069 1.669764741 0.171228843 1chr8_68489557_68491100 100.0129773 47.83447956 152.191475 3.181627069 1.669764741 0.171228843 1chr20_14769801_14771942 133.8832354 64.06403513 203.7024358 3.179669144 1.668876656 0.119332879 1chr8_21880653_21882577 133.8832354 64.06403513 203.7024358 3.179669144 1.668876656 0.119332879 1chr15_77847361_77851794 524.7754548 251.1310177 798.4198919 3.179296206 1.668707435 0.012232747 1chr1_164779985_164781288 83.8767468 40.14679535 127.6066983 3.178502721 1.668347324 0.208244275 1chr2_25695865_25697320 83.8767468 40.14679535 127.6066983 3.178502721 1.668347324 0.208244275 1chr8_69129441_69131665 162.3747501 77.73102929 247.0184709 3.177861829 1.668056399 0.091396799 1chr14_24747859_24749676 584.9540786 280.1733803 889.7347769 3.175657787 1.667055454 0.010092053 1chr9_117172935_117174227 73.11925982 35.02167254 111.2168471 3.175657787 1.667055454 0.239573621 1chr1_48161812_48166005 349.4600686 167.4206785 531.4994588 3.174634482 1.666590494 0.025969477 1chr1_84497005_84498148 67.74051633 32.45911113 103.0219215 3.17389842 1.666255956 0.257834334 1chr18_74765842_74769431 406.443098 194.7546668 618.1315292 3.17389842 1.666255956 0.019760402 1chr3_16577298_16579224 101.6107745 48.6886667 154.5328823 3.17389842 1.666255956 0.168967562 1chr3_178276139_178277201 67.74051633 32.45911113 103.0219215 3.17389842 1.666255956 0.257834334 1chr5_160400763_160402035 67.74051633 32.45911113 103.0219215 3.17389842 1.666255956 0.257834334 1chr7_18585874_18589529 260.2045783 124.7113217 395.697835 3.172910282 1.665806728 0.043651009 1chrX_118626298_118629838 226.3343202 108.4817662 344.1868742 3.17276245 1.665739509 0.055059893 1chr2_234168382_234171684 254.8258349 122.1487603 387.5029094 3.172385118 1.66556792 0.045250072 1chr2_138591103_138593321 124.7235457 59.79309945 189.6539919 3.171837447 1.665318836 0.131728697 1chr7_44693808_44695570 124.7235457 59.79309945 189.6539919 3.171837447 1.665318836 0.131728697 1chr8_73044966_73048581 311.8088642 149.4827486 474.1349798 3.171837447 1.665318836 0.031953252 1chr3_53354576_53355975 90.85328752 43.56354389 138.1430311 3.171069633 1.664969558 0.192003974 1chr19_21879855_21881059 56.98302935 27.33398832 86.63207038 3.169390041 1.664205216 0.300998965 1chr2_44373638_44375166 85.47454402 41.00098248 129.9481056 3.169390041 1.664205216 0.205217599 1chr7_131102658_131103857 56.98302935 27.33398832 86.63207038 3.169390041 1.664205216 0.300998965 1chr2_170834258_170835820 137.0788299 65.7724094 208.3852504 3.168277584 1.663698741 0.116704204 1chr14_101256232_101257936 108.5873152 52.10541524 165.0692152 3.167985792 1.663565865 0.156870139 1chr5_175214335_175215776 80.09580053 38.43842108 121.75318 3.167486504 1.663338472 0.219765532 1chr1_167586238_167588653 183.3043723 87.98127491 278.6274696 3.166895113 1.663069087 0.077187883 1chr2_203581924_203584014 126.3213429 60.64728659 191.9953992 3.165770639 1.662556736 0.130195115 1chr4_185418997_185420337 97.82982823 46.98029243 148.679364 3.164717722 1.662076824 0.177531194 1chr10_54133343_54135310 190.280913 91.39802345 289.1638025 3.163786169 1.661652096 0.073153277 1chr10_12534278_12536002 115.5638559 55.52216378 175.6055481 3.162800873 1.661202728 0.145975483 1chr14_32497457_32498762 69.33831355 33.31329827 105.3633288 3.162800873 1.661202728 0.25365602 1chr16_30592427_30596384 254.240483 122.1487603 386.3322057 3.162800873 1.661202728 0.045967188 1chr3_71419785_71421121 69.33831355 33.31329827 105.3633288 3.162800873 1.661202728 0.25365602 1chr2_143534663_143537273 214.9914814 103.3566433 326.6263194 3.160186988 1.660009925 0.060675368 1chr2_234607190_234609253 127.9191401 61.50147372 194.3368065 3.159872354 1.65986628 0.128688052 1chr4_108520846_108521990 63.95957006 30.75073686 97.16840326 3.159872354 1.65986628 0.27349773 1chr12_8874602_8878999 285.9275921 137.5241287 434.3310555 3.158217103 1.659110349 0.037863564 1chr3_38791376_38792963 81.69359776 39.29260821 124.0945873 3.158217103 1.659110349 0.216484672 1chr1_167563202_167564717 99.42762545 47.83447956 151.0207713 3.157153014 1.658624184 0.175145775 1chr17_9898087_9899071 58.58082657 28.18817546 88.97347769 3.156411376 1.658285245 0.295696418 1chr3_65897682_65900817 175.7424797 84.56452637 266.9204331 3.156411376 1.658285245 0.083066604 1chr12_120853196_120854456 76.31485426 36.73004681 115.8996617 3.155445522 1.657843716 0.232187475 1chr3_69822622_69824188 94.04888196 45.27191816 142.8258458 3.154844141 1.657568733 0.186698415 1chr8_101611615_101613271 94.04888196 45.27191816 142.8258458 3.154844141 1.657568733 0.186698415 1chr5_15371917_15373770 205.8317916 99.08570767 312.5778756 3.154621215 1.657466787 0.065398047 1chr2_27073999_27076517 147.250965 70.89753221 223.6043979 3.153909465 1.657141247 0.107185266 1chr8_99338124_99340502 147.250965 70.89753221 223.6043979 3.153909465 1.657141247 0.107185266 1chr10_49677279_49679142 384.7698658 185.3586083 584.1811232 3.151626615 1.656096623 0.022617303 1chr20_36694924_36697046 171.9615334 82.8561521 261.0669148 3.150845268 1.655738908 0.086233805 1chr1_94509691_94512170 272.9869561 131.5448188 414.4290934 3.150478272 1.65557086 0.041404502 1chr3_40916830_40918584 118.7594504 57.23053805 180.2883627 3.150212611 1.655449201 0.142438351 1chr14_87555958_87561427 416.4569749 200.7339767 632.179973 3.149342146 1.655050501 0.019675472 1chr1_84596510_84598845 214.4061295 103.3566433 325.4556157 3.148860153 1.654829686 0.061712138 1chr14_77095634_77096966 65.55736728 31.604924 99.50981057 3.148554022 1.654689421 0.268925817 1chr2_202447270_202448441 65.55736728 31.604924 99.50981057 3.148554022 1.654689421 0.268925817 1chr8_8421517_8423295 375.610176 181.0876726 570.1326794 3.148379297 1.654609358 0.023739176 1chr1_50952610_50955637 173.5593307 83.71033924 263.4083221 3.146664134 1.653823198 0.08541632 1chr8_140762162_140763473 329.3846336 158.8788071 499.8904602 3.146363377 1.653685299 0.030077592 1chr5_76133744_76136588 203.6486426 98.23152053 309.0657646 3.146299303 1.653655919 0.067096706 1chr12_65711994_65715724 414.2738258 199.8797896 628.6678621 3.145229757 1.653165409 0.019997972 1chr6_40655295_40657454 138.0912753 66.62659653 209.555954 3.145229757 1.653165409 0.117774275 1chr7_28909936_28914052 318.6271467 153.7536843 483.500609 3.144644053 1.652896726 0.031961654 1chr14_24742510_24744134 408.8950823 197.3172282 620.4729365 3.144545168 1.652851358 0.020498584 1chr1_241519542_241521234 150.4465595 72.60590648 228.2872125 3.144196162 1.652691228 0.104943639 1chr14_36366488_36367818 60.17862379 29.04236259 91.31488499 3.144196162 1.652691228 0.29053958 1chr18_55137947_55140659 180.5358714 87.12708778 273.944655 3.144196162 1.652691228 0.08077924 1chr2_151699014_151700151 60.17862379 29.04236259 91.31488499 3.144196162 1.652691228 0.29053958 1chr2_10974813_10976172 90.26793569 43.56354389 136.9723275 3.144196162 1.652691228 0.196525532 1chr9_36911395_36913057 90.26793569 43.56354389 136.9723275 3.144196162 1.652691228 0.196525532 1chr3_11769396_11771007 102.6232199 49.54285383 155.7035859 3.142806155 1.652053291 0.170516984 1chr2_138994574_138996303 157.4231002 76.02265502 238.8235454 3.14147862 1.651443761 0.09875508 1chr22_43287728_43292290 484.6245848 234.047275 735.2018946 3.141253811 1.651340516 0.015153738 1chr19_4692186_4698905 880.5790311 425.3851933 1335.772869 3.140148952 1.650832995 0.005304017 0.9889824chr1_227766462_227767834 97.2444764 46.98029243 147.5086604 3.139798685 1.65067206 0.181610363 1chr11_119514350_119516189 109.5997606 52.95960237 166.2399188 3.138994845 1.65030266 0.158304695 1chr1_47166601_47171833 463.1096108 223.7970294 702.4221923 3.138657355 1.650147539 0.016528191 1chr1_3072927_3074765 134.310329 64.91822226 203.7024358 3.137831392 1.649767833 0.122913535 1chr14_35736606_35738993 201.4654935 97.3773334 305.5536536 3.137831392 1.649767833 0.068850537 1chr2_7309376_7310555 67.1551645 32.45911113 101.8512179 3.137831392 1.649767833 0.264471537 1chr2_202974227_202975365 67.1551645 32.45911113 101.8512179 3.137831392 1.649767833 0.264471537 1chr5_119307037_119308322 67.1551645 32.45911113 101.8512179 3.137831392 1.649767833 0.264471537 1chr8_28089868_28091056 79.51044871 38.43842108 120.5824763 3.137029903 1.649399281 0.225143378 1chr12_2872268_2875491 330.397079 159.7329943 501.0611638 3.136867033 1.649324377 0.030330141 1chr15_63451746_63453466 91.86573291 44.41773102 139.3137348 3.136444199 1.649129896 0.193751379 1chr1_116226833_116228863 220.7973184 106.7733919 334.821245 3.135811639 1.648838903 0.059771013 1chr10_99107080_99111296 362.0841881 175.1083627 549.0600136 3.135544215 1.648715863 0.025852209 1chr10_79232698_79236804 411.505325 199.0256025 623.9850475 3.135199892 1.648557429 0.020576322 1chr7_4262862_4266947 381.4160131 184.5044212 578.327605 3.134491853 1.64823158 0.023602997 1chr1_217706482_217709327 203.0632907 98.23152053 307.8950609 3.134381503 1.648180789 0.068267666 1chr20_25549053_25553755 573.7218168 277.6108189 869.8328147 3.133281398 1.647674343 0.011340989 1chr1_74267270_74270400 296.5268209 143.5034387 449.550203 3.13267896 1.647396928 0.036810399 1chr1_87490482_87491345 49.42113681 23.91723978 74.92503384 3.13267896 1.647396928 0.342249884 1chr1_52761827_52763143 86.48698942 41.85516962 131.1188092 3.13267896 1.647396928 0.207075706 1chr1_84365736_84368512 222.3951156 107.627579 337.1626523 3.13267896 1.647396928 0.059300523 1chr10_74609105_74610988 111.1975578 53.81378951 168.5813261 3.13267896 1.647396928 0.156293408 1chr2_202958712_202959900 61.77642101 29.89654973 93.6562923 3.13267896 1.647396928 0.285522743 1chr21_35870890_35872438 111.1975578 53.81378951 168.5813261 3.13267896 1.647396928 0.156293408 1chr5_174430255_174431368 74.13170522 35.87585967 112.3875508 3.13267896 1.647396928 0.241727338 1chr8_112264800_112265889 61.77642101 29.89654973 93.6562923 3.13267896 1.647396928 0.285522743 1chr6_42270916_42273597 352.9244984 170.837427 535.0115698 3.131699998 1.646946016 0.027211158 1chr7_75042872_75046338 471.0985969 228.0679651 714.1292288 3.13121235 1.646721351 0.016234675 1chr8_127766222_127767786 130.5293827 63.20984799 197.8489175 3.130033116 1.646177921 0.128360037 1chr20_30345793_30349464 305.1011588 147.7743744 462.4279432 3.129283715 1.645832466 0.03521342 1chr1_55219264_55220533 81.10824593 39.29260821 122.9238836 3.128422603 1.645435412 0.221742879 1chr14_24525070_24528939 636.5106832 308.3615558 964.6598107 3.128340069 1.645397351 0.009517607 1chr2_3579754_3581565 125.1506392 60.64728659 189.6539919 3.12716368 1.644854734 0.135738464 1chr20_20020180_20022336 156.8377484 76.02265502 237.6528417 3.126079215 1.644354337 0.100667065 1chr10_11908101_11909653 132.12718 64.06403513 200.1903248 3.124847262 1.643785675 0.12688587 1chr11_73184038_73187469 352.3391466 170.837427 533.8408661 3.124847262 1.643785675 0.027574231 1chr9_5649984_5651411 88.08478664 42.70935675 133.4602165 3.124847262 1.643785675 0.204076801 1chr10_12593821_12596029 146.0802614 70.89753221 221.2629906 3.120884235 1.641954843 0.111490948 1chr6_40501930_40504232 248.1181294 120.440386 375.7958729 3.12018157 1.641629985 0.050441026 1chr7_9929400_9930749 82.70604315 40.14679535 125.265291 3.12018157 1.641629985 0.218420045 1chrX_71411453_71413050 82.70604315 40.14679535 125.265291 3.12018157 1.641629985 0.218420045 1chr1_82014371_82018514 369.4878224 179.3792984 559.5963465 3.119626131 1.641373141 0.025559488 1chr7_100439404_100441968 235.7628452 114.4610761 357.0646144 3.119528722 1.641328092 0.054842927 1chr18_13836187_13837361 102.0378681 49.54285383 154.5328823 3.119176033 1.641164975 0.174360845 1chr2_129910149_129911156 51.01893403 24.77142692 77.26644115 3.119176033 1.641164975 0.335765061 1chr8_98881558_98884403 376.4643631 182.7960469 570.1326794 3.118955191 1.641062826 0.024753463 1chr7_92840089_92843488 223.407561 108.4817662 338.3333559 3.118803905 1.640992846 0.059817779 1chr3_64227352_64229255 121.369693 58.93891232 183.8004736 3.118491102 1.640848143 0.14198204 1chr4_3281723_3283369 121.369693 58.93891232 183.8004736 3.118491102 1.640848143 0.14198204 1chr8_127755680_127757532 191.7204519 93.10639772 290.3345061 3.118308873 1.640763836 0.07580108 1chr12_94315900_94317227 70.35075895 34.1674854 106.5340325 3.117994527 1.640618396 0.255899166 1chr7_4649980_4651065 70.35075895 34.1674854 106.5340325 3.117994527 1.640618396 0.255899166 1chr7_107455978_107457232 70.35075895 34.1674854 106.5340325 3.117994527 1.640618396 0.255899166 1chr13_78640415_78642214 89.68258386 43.56354389 135.8016238 3.11732269 1.640307504 0.201143242 1chr7_17791723_17793315 89.68258386 43.56354389 135.8016238 3.11732269 1.640307504 0.201143242 1chr2_215004021_215007815 198.6969926 96.52314626 300.870839 3.117084872 1.640197437 0.071902616 1chr1_47146610_47149036 256.6924674 124.7113217 388.673613 3.116586431 1.639966723 0.047919618 1chr1_241879876_241882536 244.3371832 118.7320118 369.9423546 3.115776016 1.639591526 0.051998066 1chr4_5823492_5826502 263.6690081 128.1280703 399.2099459 3.115710282 1.639561089 0.045893854 1chr1_17510045_17511962 96.65912457 46.98029243 146.3379567 3.114879648 1.639176422 0.185774613 1chr19_19001283_19002765 96.65912457 46.98029243 146.3379567 3.114879648 1.639176422 0.185774613 1chr2_193239343_193240407 57.99547474 28.18817546 87.80277403 3.114879648 1.639176422 0.303609442 1chr8_102985521_102987288 96.65912457 46.98029243 146.3379567 3.114879648 1.639176422 0.185774613 1chr1_93342515_93344803 161.63114 78.58521643 244.6770636 3.113525352 1.638549026 0.097629956 1chr7_97958098_97960303 161.63114 78.58521643 244.6770636 3.113525352 1.638549026 0.097629956 1chr7_11214481_11218695 414.5426611 201.5881639 627.4971584 3.112767865 1.638197992 0.021069135 1chr18_54821662_54823021 84.30384037 41.00098248 127.6066983 3.112283914 1.637973675 0.215172478 1chr7_69336065_69338967 194.9160464 94.81477199 295.0173207 3.11151221 1.637615908 0.074464758 1chr5_31705537_31708139 286.1964275 139.232503 433.1603519 3.111057709 1.637405157 0.040259002 1chr1_77933639_77935702 110.612206 53.81378951 167.4106225 3.110924245 1.637343264 0.159718072 1chr10_12130140_12132270 156.2523965 76.02265502 236.4821381 3.11067981 1.637229902 0.102613731 1chr11_117538659_117542458 358.1449836 174.2541756 542.0357917 3.110604323 1.637194892 0.027378233 1chr6_55860283_55863147 247.5327776 120.440386 374.6251692 3.110461379 1.637128593 0.051243158 1chr3_53307558_53311096 293.1729682 142.6492516 443.6966848 3.110403174 1.637101596 0.038687245 1chrX_13397552_13399686 117.5887467 57.23053805 177.9469554 3.109300759 1.636590173 0.148620035 1chr4_6385472_6388445 189.5373029 92.25221059 286.8223952 3.109111352 1.636502287 0.077851363 1chr1_75155429_75157177 143.8971123 70.04334507 217.7508796 3.108801834 1.636358657 0.114954643 1chr10_21405582_21406937 71.94855617 35.02167254 108.8754398 3.108801834 1.636358657 0.251773214 1chr14_21801777_21803673 215.8456685 105.0650176 326.6263194 3.108801834 1.636358657 0.063866738 1chr15_61901217_61902498 71.94855617 35.02167254 108.8754398 3.108801834 1.636358657 0.251773214 1chr8_99001733_99002944 71.94855617 35.02167254 108.8754398 3.108801834 1.636358657 0.251773214 1chr9_110415171_110416323 71.94855617 35.02167254 108.8754398 3.108801834 1.636358657 0.251773214 1chr12_26031513_26033424 150.873653 73.46009361 228.2872125 3.107635741 1.63581741 0.107960204 1chr18_63597584_63599436 150.873653 73.46009361 228.2872125 3.107635741 1.63581741 0.107960204 1chr18_47426061_47427048 52.61673125 25.62561405 79.60784846 3.106573302 1.635324096 0.329476932 1chr19_17894621_17895621 52.61673125 25.62561405 79.60784846 3.106573302 1.635324096 0.329476932 1chr10_71245360_71247496 540.120394 263.0896376 817.1511503 3.105979991 1.635048536 0.013303449 1chr2_206079862_206084178 303.3451033 147.7743744 458.9158323 3.105517004 1.634833467 0.036755842 1chr12_110093016_110094241 59.59327197 29.04236259 90.14418134 3.103885955 1.63407555 0.298241966 1chr8_11142228_11145253 297.9663598 145.211813 450.7209067 3.103885955 1.63407555 0.037975293 1chr7_55192336_55193729 245.3496286 119.5861989 371.1130582 3.103310095 1.633807864 0.052441651 1chr3_66037478_66040614 192.7328973 93.96058486 291.5052098 3.102420129 1.633394069 0.076461465 1chr10_44845572_44848572 226.0178037 110.1901404 341.8454669 3.102323544 1.633349154 0.059857603 1chr14_28786916_28788080 66.56981268 32.45911113 100.6805142 3.101764365 1.633089092 0.271259562 1chr2_169834771_169835861 66.56981268 32.45911113 100.6805142 3.101764365 1.633089092 0.271259562 1chr8_9408227_9410180 133.1396254 64.91822226 201.3610284 3.101764365 1.633089092 0.128008469 1chr6_41565984_41567939 775.7249809 378.4049008 1173.045061 3.099973226 1.632255755 0.007176289 1chr6_90679361_90682393 187.3541538 91.39802345 283.3102842 3.099741915 1.632148101 0.079970827 1chr14_32717938_32719730 154.0692475 75.16846788 232.9700271 3.099305249 1.631944852 0.105699323 1chr1_57219988_57221944 201.3072353 98.23152053 304.38295 3.098628102 1.631629613 0.071898394 1chr5_174896530_174897809 120.7843412 58.93891232 182.62977 3.098628102 1.631629613 0.144993952 1chr1_48216712_48219566 181.9754103 88.83546205 275.1153586 3.096909188 1.630829079 0.083710015 1chr3_123138435_123142639 363.9508207 177.6709241 550.2307173 3.096909188 1.630829079 0.027183418 1chr7_140349404_140352045 229.2133981 111.8985147 346.5282815 3.09680859 1.630782215 0.05892308 1chr2_112538814_112539748 47.23798776 23.06305265 71.41292288 3.096421101 1.630601686 0.358800498 1chr2_192310230_192312919 195.9284918 95.66895913 296.1880244 3.095967878 1.630390503 0.075109289 1chr1_87399077_87400728 101.4525162 49.54285383 153.3621786 3.095545912 1.630193857 0.178283564 1chr18_74791868_74793346 101.4525162 49.54285383 153.3621786 3.095545912 1.630193857 0.178283564 1chr12_3441497_3443544 209.8815732 102.5024562 317.2606902 3.095152076 1.630010296 0.067721382 1chr1_60614638_60615760 54.21452847 26.47980119 81.94925576 3.09478365 1.629838557 0.323376654 1chr1_111704194_111705251 54.21452847 26.47980119 81.94925576 3.09478365 1.629838557 0.323376654 1chr14_78675693_78678300 162.6435854 79.43940356 245.8477673 3.09478365 1.629838557 0.098483309 1chr1_71132072_71134747 223.8346546 109.3359533 338.3333559 3.09443825 1.629677533 0.061335402 1chr12_98966535_98969524 223.8346546 109.3359533 338.3333559 3.09443825 1.629677533 0.061335402 1chr7_541786_546641 631.2425168 308.3615558 954.1234778 3.094171306 1.629553073 0.010345801 1chr22_41805548_41808032 298.9788052 146.0660001 451.8916103 3.09374947 1.629356373 0.038288555 1chr1_84976730_84977880 61.19106919 29.89654973 92.48558865 3.093520473 1.629249582 0.293023151 1chr11_130008349_130009378 61.19106919 29.89654973 92.48558865 3.093520473 1.629249582 0.293023151 1chr4_40837284_40838350 61.19106919 29.89654973 92.48558865 3.093520473 1.629249582 0.293023151 1chr6_89922314_89923304 61.19106919 29.89654973 92.48558865 3.093520473 1.629249582 0.293023151 1chr14_24538662_24540954 251.7408175 123.0029474 380.4786875 3.093248539 1.629122757 0.050931498 1chr21_43887009_43890131 265.6938989 129.8364445 401.5513532 3.092747608 1.628889103 0.04666988 1chr1_109189282_109190399 68.1676099 33.31329827 103.0219215 3.092516409 1.62878125 0.266751393 1chr1_198551439_198552575 68.1676099 33.31329827 103.0219215 3.092516409 1.62878125 0.266751393 1chr3_32070127_32071476 68.1676099 33.31329827 103.0219215 3.092516409 1.62878125 0.266751393 1chr12_51118182_51120413 150.2883012 73.46009361 227.1165088 3.091699148 1.628399938 0.110072334 1chr8_11217683_11221101 636.0359084 310.9241172 961.1476997 3.091261329 1.628195622 0.010270778 1chr2_174116978_174118388 82.12069132 40.14679535 124.0945873 3.091020995 1.628083453 0.223673674 1chr6_1024156_1025396 82.12069132 40.14679535 124.0945873 3.091020995 1.628083453 0.223673674 1chr4_84249338_84252291 260.3151554 127.2738831 393.3564277 3.090629578 1.627900753 0.048390184 1chr3_31602732_31606323 445.4861602 217.8177194 673.1546009 3.090449219 1.627816559 0.019252613 1chr12_90968512_90970657 185.1710048 90.54383632 279.7981732 3.090195696 1.627698204 0.082162236 1chr1_54860624_54864714 466.4157823 228.0679651 704.7635996 3.09014727 1.627675596 0.017761195 1chr12_69164695_69166072 96.07377275 46.98029243 145.1672531 3.08996061 1.627588447 0.190025409 1chr3_58612508_58615281 206.1006269 100.7940819 311.4071719 3.089538254 1.627391237 0.070061928 1chr12_1447667_1449520 117.0033949 57.23053805 176.7762517 3.088844833 1.627067399 0.151802638 1chr4_56303104_56305106 123.9799356 60.64728659 187.3125846 3.088556721 1.626932826 0.141499392 1chr2_235197458_235199652 144.9095577 70.89753221 218.9215833 3.087859005 1.626606879 0.115957702 1chr8_112750578_112753811 289.8191155 141.7950644 437.8431665 3.087859005 1.626606879 0.040669393 1chr11_129016764_129019884 324.701819 158.8788071 490.5248309 3.087415117 1.626399473 0.033579548 1chr18_12551256_12553986 375.1354012 183.650234 566.6205684 3.08532451 1.625422238 0.026254979 1chr1_117025857_117028579 216.2727621 105.9192047 326.6263194 3.083730851 1.624676852 0.065498523 1chr12_54859514_54860639 55.8123257 27.33398832 84.29066307 3.083730851 1.624676852 0.317455946 1chr12_96524810_96525962 62.78886641 30.75073686 94.82699595 3.083730851 1.624676852 0.287947073 1chr14_40788804_40790104 90.69502926 44.41773102 136.9723275 3.083730851 1.624676852 0.202890104 1chr14_105497933_105499902 139.5308142 68.3349708 210.7266577 3.083730851 1.624676852 0.122291676 1chr15_94355937_94357149 76.74194783 37.58423394 115.8996617 3.083730851 1.624676852 0.239992882 1chr2_202599953_202600850 48.83578498 23.91723978 73.75433019 3.083730851 1.624676852 0.351815136 1chr21_30450425_30452352 125.5777328 61.50147372 189.6539919 3.083730851 1.624676852 0.139799488 1chr22_23131257_23133006 83.71848854 41.00098248 126.4359946 3.083730851 1.624676852 0.220311067 1chr3_51653529_51656335 153.4838957 75.16846788 231.7993234 3.083730851 1.624676852 0.107747831 1chr6_80045927_80047048 69.76540712 34.1674854 105.3633288 3.083730851 1.624676852 0.262358838 1chr6_140310707_140312567 104.6481107 51.2512281 158.0449933 3.083730851 1.624676852 0.173540113 1chr7_18609796_18610989 69.76540712 34.1674854 105.3633288 3.083730851 1.624676852 0.262358838 1chr7_43949828_43951526 125.5777328 61.50147372 189.6539919 3.083730851 1.624676852 0.139799488 1chr8_9127041_9128261 69.76540712 34.1674854 105.3633288 3.083730851 1.624676852 0.262358838 1chrX_153185895_153189275 322.51867 158.02462 487.01272 3.08187876 1.623810108 0.034241017 1chr1_116403818_116406764 252.7532629 123.8571346 381.6493911 3.081367839 1.623570915 0.051356836 1chr22_27894289_27899111 449.6942 220.3802808 679.0081192 3.081074752 1.623433685 0.019243989 1chr8_74112583_74114537 169.0347743 82.8561521 255.2133965 3.080198513 1.623023333 0.094599972 1chr1_54325957_54328566 155.0816929 76.02265502 234.1407308 3.079881 1.622874609 0.106613343 1chr19_4566532_4568364 148.1051522 72.60590648 223.6043979 3.07969983 1.622789742 0.11344745 1chr12_97673377_97675346 127.17553 62.35566086 191.9953992 3.079037197 1.622479296 0.138130176 1chr3_69245841_69247844 127.17553 62.35566086 191.9953992 3.079037197 1.622479296 0.138130176 1chr2_239013921_239016940 360.596968 176.816737 544.377199 3.078765101 1.622351799 0.028406086 1chr3_66551674_66553274 120.1989893 58.93891232 181.4590663 3.078765101 1.622351799 0.148064649 1chrX_24150213_24152163 120.1989893 58.93891232 181.4590663 3.078765101 1.622351799 0.148064649 1chr1_49180601_49182168 99.26936719 48.6886667 149.8500677 3.07771968 1.621861836 0.184812236 1chr7_29923930_29925248 92.29282648 45.27191816 139.3137348 3.077266007 1.621649159 0.199986042 1chr20_48256980_48260926 518.8742553 254.5477662 783.2007444 3.076832124 1.621445731 0.015077715 1chr1_46728746_46730319 78.33974505 38.43842108 118.241069 3.076116701 1.621110237 0.236254165 1chr5_133152430_133154458 149.7029494 73.46009361 225.9458052 3.075762554 1.620944133 0.112223023 1chr2_191859866_191861665 142.7264087 70.04334507 215.4094723 3.075373857 1.620761802 0.119576627 1chr9_96856427_96857571 71.36320434 35.02167254 107.7047361 3.075373857 1.620761802 0.258077727 1chr10_12496812_12498477 128.7733273 63.20984799 194.3368065 3.074470398 1.620337916 0.136490738 1chr14_34302562_34307688 708.2532999 347.654164 1068.852436 3.074470398 1.620337916 0.008842127 1chr22_34849922_34851052 64.38666363 31.604924 97.16840326 3.074470398 1.620337916 0.283008131 1chrX_35578164_35579333 64.38666363 31.604924 97.16840326 3.074470398 1.620337916 0.283008131 1chr2_239233405_239235992 186.1834502 91.39802345 280.9688769 3.074124213 1.62017546 0.082862903 1chr2_159818575_159819710 57.41012292 28.18817546 86.63207038 3.073347919 1.619811097 0.311707039 1chr4_166150684_166151634 57.41012292 28.18817546 86.63207038 3.073347919 1.619811097 0.311707039 1chr17_800846_802229 100.8671644 49.54285383 152.191475 3.07191579 1.619138668 0.182286487 1chr2_166042433_166043451 50.43358221 24.77142692 76.09573749 3.07191579 1.619138668 0.34504996 1chr3_41709328_41710392 50.43358221 24.77142692 76.09573749 3.07191579 1.619138668 0.34504996 1chr22_45092840_45095677 288.6484118 141.7950644 435.5017592 3.07134639 1.61887123 0.041870976 1chr13_51782401_51783996 86.91408299 42.70935675 131.1188092 3.070025381 1.618250583 0.213813461 1chr16_69792738_69794116 86.91408299 42.70935675 131.1188092 3.070025381 1.618250583 0.213813461 1chr1_172390611_172392998 210.3086668 103.3566433 317.2606902 3.069572307 1.618037655 0.069457566 1chr13_40637106_40639730 210.3086668 103.3566433 317.2606902 3.069572307 1.618037655 0.069457566 1chr6_80372944_80374455 166.8516253 82.00196497 251.7012856 3.069454319 1.617982199 0.097336632 1chr1_64401931_64403532 123.3945838 60.64728659 186.1418809 3.069253241 1.617887686 0.144463727 1chr1_75014560_75016222 79.93754227 39.29260821 120.5824763 3.068833601 1.617690422 0.23260429 1chr7_72175554_72176763 79.93754227 39.29260821 120.5824763 3.068833601 1.617690422 0.23260429 1chr3_155293148_155296129 189.3790446 93.10639772 285.6516915 3.068013568 1.617304863 0.081341381 1chr6_166903917_166905219 72.96100156 35.87585967 110.0461435 3.067414815 1.617023279 0.253904096 1chr3_71471203_71475020 343.8753857 169.1290527 518.6217186 3.066425964 1.616558119 0.031402152 1chr1_54894424_54895574 65.98446085 32.45911113 99.50981057 3.065697337 1.616215273 0.278201027 1chr4_6012838_6014012 65.98446085 32.45911113 99.50981057 3.065697337 1.616215273 0.278201027 1chr2_103340071_103341424 95.48842092 46.98029243 143.9965494 3.065041573 1.615906642 0.194364232 1chr2_224783083_224784877 95.48842092 46.98029243 143.9965494 3.065041573 1.615906642 0.194364232 1chrX_69833473_69834855 95.48842092 46.98029243 143.9965494 3.065041573 1.615906642 0.194364232 1chr9_15935559_15937250 124.992381 61.50147372 188.4832883 3.064695475 1.615743727 0.142712607 1chr4_936977_943341 838.4661661 412.5723862 1264.359946 3.064577243 1.615688069 0.006827231 1chrX_7087088_7089824 213.5042612 105.0650176 321.9435048 3.064231198 1.615525154 0.068290924 1chr10_119333587_119334723 59.00792014 29.04236259 88.97347769 3.063575747 1.615216523 0.306122633 1chr18_33795181_33796472 88.51188021 43.56354389 133.4602165 3.063575747 1.615216523 0.2106738 1chr7_122425041_122426363 88.51188021 43.56354389 133.4602165 3.063575747 1.615216523 0.2106738 1chr4_41308855_41310737 140.5432596 69.18915794 211.8973613 3.062580434 1.614747735 0.123336482 1chr11_116661609_116663581 111.0392996 54.66797664 167.4106225 3.062316053 1.614623187 0.16460771 1chr10_50087899_50089131 81.5353395 40.14679535 122.9238836 3.061860419 1.614408516 0.22904035 1chr8_128559786_128560945 81.5353395 40.14679535 122.9238836 3.061860419 1.614408516 0.22904035 1chr1_242000842_242001890 52.03137943 25.62561405 78.4371448 3.0608884 1.613950445 0.338494519 1chr5_150499230_150501633 178.6215576 87.98127491 269.2618404 3.060444858 1.613741374 0.089197706 1chr15_51652026_51653201 74.55879878 36.73004681 112.3875508 3.059825961 1.613449596 0.24983417 1chr8_9524617_9527328 246.2038157 121.2945732 371.1130582 3.059601502 1.613343761 0.055024955 1chr3_13695876_13697619 164.6684762 81.14777783 248.1891746 3.058483932 1.612816697 0.100171868 1chr2_120159810_120161337 90.10967743 44.41773102 135.8016238 3.057374177 1.612293127 0.207603946 1chr2_204986600_204988088 90.10967743 44.41773102 135.8016238 3.057374177 1.612293127 0.207603946 1chr20_17839249_17841766 180.2193549 88.83546205 271.6032477 3.057374177 1.612293127 0.088343245 1chrX_117774023_117775886 112.6370968 55.52216378 169.7520298 3.057374177 1.612293127 0.162483151 1chr15_90513007_90515909 204.3445714 100.7940819 307.8950609 3.054693837 1.61102779 0.073734411 1chr15_90843831_90845421 121.2114347 59.79309945 182.62977 3.054361986 1.610871052 0.149322757 1chr20_36779813_36781105 60.60571736 29.89654973 91.31488499 3.054361986 1.610871052 0.300695866 1chr5_31698344_31700062 197.3680307 97.3773334 297.3587281 3.053674995 1.610546523 0.077720067 1chr8_10500247_10505182 683.8115668 337.4039183 1030.219215 3.053370632 1.610402721 0.009809783 1chr13_52342705_52346492 349.6812227 172.5458013 526.8166442 3.053198862 1.610321559 0.031134604 1chr6_38987122_38989124 174.8406114 86.27290064 263.4083221 3.053198862 1.610321559 0.092610354 1chr19_4186116_4187355 76.15659601 37.58423394 114.7289581 3.052582055 1.610030076 0.245864351 1chr17_54651052_54654372 335.7281413 165.7123042 505.7439784 3.051939811 1.60972651 0.033421006 1chr21_43957996_43959782 297.6498433 146.9201872 448.3794994 3.051857664 1.609687678 0.040872997 1chr14_24178739_24180467 145.3366513 71.75171934 218.9215833 3.051098779 1.609328888 0.119252521 1chr14_32902387_32904607 198.9658279 98.23152053 299.7001354 3.0509569 1.6092618 0.077025608 1chr17_74009483_74012344 375.4042365 185.3586083 565.4498648 3.050572455 1.609079997 0.027696249 1chr2_131486311_131487360 53.62917665 26.47980119 80.77855211 3.050572455 1.609079997 0.332139073 1chr2_203049833_203051680 160.8875299 79.43940356 242.3356563 3.050572455 1.609079997 0.104186309 1chr5_27979293_27980284 53.62917665 26.47980119 80.77855211 3.050572455 1.609079997 0.332139073 1chr8_123769782_123771363 107.2583533 52.95960237 161.5571042 3.050572455 1.609079997 0.17268861 1chrX_81473788_81475286 107.2583533 52.95960237 161.5571042 3.050572455 1.609079997 0.17268861 1chr1_59238437_59241067 230.0675852 113.606889 346.5282815 3.05024004 1.608922781 0.061884138 1chr8_120132288_120134125 138.3601106 68.3349708 208.3852504 3.049467175 1.608557187 0.1272438 1chr20_18407451_18412546 499.8112657 246.860082 752.7624494 3.049348615 1.608501095 0.016928452 1chr5_32831071_32834353 338.9237358 167.4206785 510.426793 3.048767916 1.608226331 0.033046338 1chr12_64535170_64537476 300.8454378 148.6285615 453.062314 3.048285669 1.607998111 0.040357738 1chr4_176827691_176829341 100.2818126 49.54285383 151.0207713 3.048285669 1.607998111 0.186370972 1chr1_54288211_54289364 62.20351458 30.75073686 93.6562923 3.0456601 1.606754944 0.295420273 1chr13_27977601_27979060 77.75439323 38.43842108 117.0703654 3.0456601 1.606754944 0.241991208 1chr14_36170354_36171376 62.20351458 30.75073686 93.6562923 3.0456601 1.606754944 0.295420273 1chr15_57946112_57947794 124.4070292 61.50147372 187.3125846 3.0456601 1.606754944 0.145681628 1chrX_154020385_154022326 124.4070292 61.50147372 187.3125846 3.0456601 1.606754944 0.145681628 1chr10_48571138_48575394 459.5498186 227.2137779 691.8858594 3.045087607 1.606483735 0.019727024 1chr20_19805476_19809496 335.1427895 165.7123042 504.5732748 3.044875136 1.606383067 0.033872561 1chr4_147045018_147047773 148.5322457 73.46009361 223.6043979 3.043889367 1.605915924 0.116642561 1chr10_13678797_13680551 101.8796098 50.39704097 153.3621786 3.043079032 1.605531803 0.183817163 1chr2_5887341_5891097 580.1759015 287.0068774 873.3449257 3.04294076 1.605466248 0.013251313 1chr22_50890119_50892857 188.208341 93.10639772 283.3102842 3.042865916 1.605430763 0.084253237 1chr8_56962298_56963573 86.32873116 42.70935675 129.9481056 3.04261444 1.605311527 0.218836898 1chr13_114051712_114052994 70.77785251 35.02167254 106.5340325 3.04194588 1.604994486 0.264521862 1chr2_213945390_213947608 141.555705 70.04334507 213.068065 3.04194588 1.604994486 0.124371097 1chr4_8726181_8727660 141.555705 70.04334507 213.068065 3.04194588 1.604994486 0.124371097 1chr4_140645950_140647313 70.77785251 35.02167254 106.5340325 3.04194588 1.604994486 0.264521862 1chrX_4853394_4854895 126.0048264 62.35566086 189.6539919 3.041487963 1.604777294 0.143911176 1chr18_42450210_42451770 110.4539477 54.66797664 166.2399188 3.040901256 1.60449897 0.168174005 1chr8_145478297_145480959 364.0613977 180.2334855 547.88931 3.039886337 1.604017381 0.029675589 1chr1_82023063_82025142 174.2552595 86.27290064 262.2376184 3.03962909 1.60389529 0.094304417 1chr14_39806602_39809853 293.2835452 145.211813 441.3552775 3.039389623 1.603781628 0.042605295 1chr1_167471792_167474050 198.3804761 98.23152053 298.5294317 3.0390391 1.603615236 0.078360596 1chr3_87496126_87497631 119.0282857 58.93891232 179.117659 3.0390391 1.603615236 0.154386325 1chr7_21146350_21149112 238.0565713 117.8778246 358.235318 3.0390391 1.603615236 0.059485939 1chr10_74513113_74514973 103.477407 51.2512281 155.7035859 3.03804595 1.60314369 0.181316092 1chr2_149906055_149907243 63.8013118 31.604924 95.99769961 3.037428586 1.602850489 0.290289757 1chr5_158719922_158721269 63.8013118 31.604924 95.99769961 3.037428586 1.602850489 0.290289757 1chr1_150468679_150471108 224.1034899 111.0443276 337.1626523 3.036288838 1.602309039 0.065496825 1chr14_40813859_40815683 112.051745 55.52216378 168.5813261 3.036288838 1.602309039 0.165983416 1chr1_116432706_116434466 120.6260829 59.79309945 181.4590663 3.034782742 1.601593239 0.152457443 1chr15_39718876_39720562 96.50086631 47.83447956 145.1672531 3.034782742 1.601593239 0.195981694 1chrX_94584372_94586735 144.7512995 71.75171934 217.7508796 3.034782742 1.601593239 0.121595474 1chr14_36399137_36404036 684.0804021 339.1122926 1029.048512 3.034536152 1.601476008 0.010198778 1chr8_145658722_145661622 813.280823 403.1763277 1223.385318 3.034367928 1.601396028 0.007678838 1chr16_23445753_23447704 129.2004208 64.06403513 194.3368065 3.033477459 1.600972591 0.140466497 1chr8_9082017_9084055 129.2004208 64.06403513 194.3368065 3.033477459 1.600972591 0.140466497 1chr2_201179823_201185788 549.5012378 272.4856961 826.5167796 3.033248319 1.60086361 0.014818139 1chr1_81480307_81481883 80.94998767 40.14679535 121.75318 3.032699844 1.600602717 0.234522006 1chr7_82574029_82575406 80.94998767 40.14679535 121.75318 3.032699844 1.600602717 0.234522006 1chr8_21153627_21155183 80.94998767 40.14679535 121.75318 3.032699844 1.600602717 0.234522006 1chr1_53951034_53951959 56.82477109 28.18817546 85.46136672 3.03181619 1.60018229 0.319992367 1chr2_152661601_152662550 56.82477109 28.18817546 85.46136672 3.03181619 1.60018229 0.319992367 1chr2_206664862_206667458 203.1738678 100.7940819 305.5536536 3.031464226 1.600014798 0.076283564 1chr12_27431921_27433332 89.5243256 44.41773102 134.6309202 3.031017503 1.599802183 0.212416296 1chr12_111539755_111542871 220.3225436 109.3359533 331.309134 3.030193857 1.599410093 0.067688342 1chr3_66643211_66646111 270.170774 134.1073802 406.2341679 3.029170857 1.598922954 0.049331484 1chr6_41585282_41587096 615.9127922 305.7989944 926.0265901 3.028219867 1.598469958 0.012325189 1chr12_55189015_55190194 73.97344696 36.73004681 111.2168471 3.027952774 1.598342704 0.255976257 1chr18_53199789_53201015 73.97344696 36.73004681 111.2168471 3.027952774 1.598342704 0.255976257 1chr2_232396511_232397652 73.97344696 36.73004681 111.2168471 3.027952774 1.598342704 0.255976257 1chr1_55046467_55048343 230.4946788 114.4610761 346.5282815 3.027477054 1.598116026 0.063397227 1chr8_8425928_8427217 271.7685712 134.9615673 408.5755752 3.027347587 1.598054328 0.048985576 1chr2_48002193_48005404 306.065923 152.04531 460.0865359 3.025983082 1.597403922 0.040477696 1chr12_104892982_104895979 223.5181381 111.0443276 335.9919486 3.025746168 1.597290964 0.066571692 1chr2_205591798_205593247 91.12212282 45.27191816 136.9723275 3.02554725 1.597196115 0.209313465 1chr15_63518650_63523092 331.7889368 164.8581171 498.7197565 3.025145291 1.597004433 0.035468923 1chr1_65057623_65059889 166.693367 82.8561521 250.5305819 3.023681109 1.596305995 0.101836589 1chr4_89709453_89711744 166.693367 82.8561521 250.5305819 3.023681109 1.596305995 0.101836589 1chr5_32627422_32629409 166.693367 82.8561521 250.5305819 3.023681109 1.596305995 0.101836589 1chr12_59470536_59472252 116.8451366 58.08472518 175.6055481 3.02326554 1.5961077 0.159664751 1chr21_47056778_47062842 752.5168473 374.1339652 1130.89973 3.022713346 1.59584417 0.008937361 1chr21_34608254_34609398 66.99690625 33.31329827 100.6805142 3.022231945 1.595614386 0.280441259 1chrX_135622852_135624756 142.5681504 70.89753221 214.2387686 3.021808545 1.595412257 0.125395662 1chr1_15225223_15227237 151.1424884 75.16846788 227.1165088 3.021433258 1.595233074 0.116318661 1chr4_25966638_25968198 75.57124418 37.58423394 113.5582544 3.021433258 1.595233074 0.251863369 1chr8_11661693_11665546 783.0332528 389.5093336 1176.557172 3.020613553 1.594841622 0.008417945 1chr1_55801568_55803334 92.71992004 46.12610529 139.3137348 3.020279599 1.594682112 0.206279371 1chr5_72673806_72675145 92.71992004 46.12610529 139.3137348 3.020279599 1.594682112 0.206279371 1chr8_10507949_10515274 1823.296644 907.1467374 2739.44655 3.019849421 1.594476614 0.00258065 0.70682191chr4_6523815_6525082 101.294258 50.39704097 152.191475 3.019849421 1.594476614 0.187902565 1chr10_44883816_44886973 321.0314498 159.7329943 482.3299053 3.019600976 1.594357918 0.037764621 1chr2_134895709_134898921 329.6057877 164.0039299 495.2076455 3.019486458 1.594303203 0.036160748 1chr1_232407829_232410782 222.9327863 111.0443276 334.821245 3.015203499 1.592255374 0.067663368 1chr11_125049157_125051674 205.7841104 102.5024562 309.0657646 3.015203499 1.592255374 0.076218857 1chr12_92600305_92602381 120.0407311 59.79309945 180.2883627 3.015203499 1.592255374 0.155652506 1chr12_38015961_38016917 51.4460276 25.62561405 77.26644115 3.015203499 1.592255374 0.347724799 1chr17_40329168_40330238 60.02036553 29.89654973 90.14418134 3.015203499 1.592255374 0.308543824 1chr19_16848387_16849494 77.1690414 38.43842108 115.8996617 3.015203499 1.592255374 0.24785211 1chr2_137197408_137198843 77.1690414 38.43842108 115.8996617 3.015203499 1.592255374 0.24785211 1chr2_152918672_152920758 188.6354345 93.96058486 283.3102842 3.015203499 1.592255374 0.086440289 1chr2_232774595_232776359 102.8920552 51.2512281 154.5328823 3.015203499 1.592255374 0.185321799 1chr20_24819999_24821321 94.31771727 46.98029243 141.6551421 3.015203499 1.592255374 0.203311943 1chr3_58631830_58634465 248.6558001 123.8571346 373.4544655 3.015203499 1.592255374 0.057245222 1chr5_38022924_38024417 111.4663931 55.52216378 167.4106225 3.015203499 1.592255374 0.169552515 1chr5_153520909_153522150 68.59470347 34.1674854 103.0219215 3.015203499 1.592255374 0.275712694 1chr7_67135289_67137130 145.7637449 72.60590648 218.9215833 3.015203499 1.592255374 0.122590758 1chr12_109949144_109952601 438.8890318 218.6719066 659.1061571 3.014132759 1.591742963 0.022525138 1chr14_40439064_40440682 173.0845559 86.27290064 259.8962111 3.012489544 1.590956234 0.097780361 1chr11_67405741_67406827 320.446098 159.7329943 481.1592017 3.012271847 1.590851974 0.038283918 1chr2_139362221_139364244 130.2128662 64.91822226 195.5075102 3.011596796 1.590528629 0.141614476 1chr6_43112676_43119704 921.6621987 459.5526787 1383.771719 3.011127522 1.590303808 0.006651411 1chr20_2613586_2615035 113.0641904 56.37635091 169.7520298 3.011050326 1.590266821 0.167338971 1chr11_96008204_96013494 400.8107338 199.8797896 601.741678 3.010517868 1.59001168 0.026429364 1chr1_66604623_66607011 191.831029 95.66895913 287.9930988 3.010308688 1.589911434 0.084829414 1chr14_79032696_79034201 95.91551449 47.83447956 143.9965494 3.010308688 1.589911434 0.200409189 1chr15_91522432_91523781 95.91551449 47.83447956 143.9965494 3.010308688 1.589911434 0.200409189 1chr22_24978028_24982756 559.9422083 279.3191932 840.5652234 3.009335713 1.589445059 0.015027392 1chrX_149974890_149976252 78.76683862 39.29260821 118.241069 3.009244599 1.589401377 0.243938967 1chr4_76359938_76361537 148.9593393 74.31428075 223.6043979 3.008902133 1.589237183 0.119879973 1chr8_128505856_128507845 148.9593393 74.31428075 223.6043979 3.008902133 1.589237183 0.119879973 1chr17_60770851_60773080 140.3850014 70.04334507 210.7266577 3.008517903 1.589052942 0.129343648 1chr12_93237584_93238785 61.61816275 30.75073686 92.48558865 3.007589349 1.588607597 0.303062933 1chr8_70473387_70475685 176.2801503 87.98127491 264.5790257 3.00721973 1.588430286 0.095844138 1chr3_37851717_37856329 343.9859627 171.6916141 516.2803113 3.007021128 1.588335005 0.034324593 1chr8_118344398_118346243 167.7058124 83.71033924 251.7012856 3.006812394 1.588234856 0.102660145 1chr19_16970028_16973434 220.7496372 110.1901404 331.309134 3.006703982 1.588182838 0.069353209 1chr1_29397660_29398740 53.04382482 26.47980119 79.60784846 3.00636126 1.588018381 0.341106516 1chr19_39302920_39305222 212.1752993 105.9192047 318.4313938 3.00636126 1.588018381 0.073588008 1chr7_32625840_32626869 53.04382482 26.47980119 79.60784846 3.00636126 1.588018381 0.341106516 1chr12_76049622_76051238 97.51331171 48.6886667 146.3379567 3.005585625 1.587646121 0.197569196 1chr16_70621374_70627063 620.9750192 310.06993 931.8801084 3.005386908 1.587550733 0.012730988 1chr2_234614181_234616110 141.9827986 70.89753221 213.068065 3.00529593 1.587507059 0.127864221 1chr1_241851579_241854115 186.4522855 93.10639772 279.7981732 3.005144438 1.587434334 0.088806323 1chr3_55325410_55327323 124.8341227 62.35566086 187.3125846 3.003938729 1.586855386 0.149914525 1chr6_42687858_42689371 124.8341227 62.35566086 187.3125846 3.003938729 1.586855386 0.149914525 1chr6_53809845_53811426 205.1987586 102.5024562 307.8950609 3.003782273 1.586780244 0.077512466 1chr1_115906878_115908121 80.36463584 40.14679535 120.5824763 3.003539269 1.586663526 0.240120588 1chr2_203619032_203625009 509.5086261 254.5477662 764.4694859 3.003245706 1.586522512 0.017831025 1chr1_168310129_168312705 304.3098675 152.04531 456.574425 3.002883975 1.586348733 0.042214654 1chr2_14983479_14985759 206.7965558 103.3566433 310.2364682 3.001611297 1.585737163 0.076825038 1chr1_47095254_47098408 305.9076647 152.8994972 458.9158323 3.001421461 1.585645917 0.041944826 1chr11_59658220_59660091 162.3270689 81.14777783 243.50636 3.000776688 1.58533596 0.107885544 1chr3_59486756_59487883 63.21595998 31.604924 94.82699595 3.000386774 1.585148488 0.297735341 1chr8_102311139_102314567 252.8638399 126.419696 379.3079838 3.000386774 1.585148488 0.056821891 1chr1_197755328_197757594 543.220626 271.6315089 814.809743 2.999687872 1.584812391 0.016111594 1chr7_83138157_83139551 90.536771 45.27191816 135.8016238 2.999687872 1.584812391 0.21412267 1chr1_50939653_50942598 281.7824481 140.9408773 422.624019 2.998590807 1.584284663 0.048046246 1chr1_7377241_7378692 81.96243306 41.00098248 122.9238836 2.998071661 1.584034867 0.236393778 1chr1_51537270_51538355 54.64162204 27.33398832 81.94925576 2.998071661 1.584034867 0.334690873 1chr10_44556131_44558091 109.2832441 54.66797664 163.8985115 2.998071661 1.584034867 0.175519187 1chr12_100052027_100053092 54.64162204 27.33398832 81.94925576 2.998071661 1.584034867 0.334690873 1chr20_36269412_36270727 81.96243306 41.00098248 122.9238836 2.998071661 1.584034867 0.236393778 1chr1_20506265_20508932 209.9921502 105.0650176 314.9192829 2.997375245 1.583699708 0.075478201 1chr2_208330575_208335688 404.4334218 202.442351 606.4244926 2.995541642 1.582816889 0.026683442 1chr2_176305669_176309683 303.7245157 152.04531 455.4037213 2.995184272 1.582644764 0.042809508 1chr11_74850669_74852596 138.2018523 69.18915794 207.2145467 2.994899098 1.582507398 0.133446255 1chr12_42921748_42922514 46.06728411 23.06305265 69.07151557 2.994899098 1.582507398 0.379239423 1chr21_34813369_34815464 184.2691364 92.25221059 276.2860623 2.994899098 1.582507398 0.0912527 1chr4_124983282_124984965 92.13456822 46.12610529 138.1430311 2.994899098 1.582507398 0.21098842 1chr5_60616906_60619819 230.3364205 115.3152632 345.3575779 2.994899098 1.582507398 0.065977352 1chr12_104594880_104595984 64.8137572 32.45911113 97.16840326 2.993563282 1.581863768 0.292554818 1chr22_29756758_29759437 148.3739875 74.31428075 222.4336942 2.993148719 1.581663963 0.122196046 1chr1_54602026_54604339 231.9342178 116.1694504 347.6989852 2.993032885 1.58160813 0.065439714 1chr7_22231630_22233887 334.2409211 167.4206785 501.0611638 2.992827221 1.581508993 0.03676489 1chr10_21530902_21533582 196.0390688 98.23152053 293.8466171 2.991367898 1.580805353 0.08392294 1chr1_114529753_114530927 56.23941926 28.18817546 84.29066307 2.990284462 1.580282733 0.328468591 1chr1_117732177_117733889 149.9717847 75.16846788 224.7751015 2.990284462 1.580282733 0.120838616 1chr16_2448013_2449397 93.73236544 46.98029243 140.4844385 2.990284462 1.580282733 0.207923859 1chr22_19904139_19905971 131.2253116 65.7724094 196.6782138 2.990284462 1.580282733 0.142749305 1chr7_1654345_1655729 93.73236544 46.98029243 140.4844385 2.990284462 1.580282733 0.207923859 1chr8_119468935_119470676 131.2253116 65.7724094 196.6782138 2.990284462 1.580282733 0.142749305 1chr5_168415970_168419685 366.3551238 183.650234 549.0600136 2.989704949 1.580003113 0.031760557 1chr6_145950109_145952750 197.636866 99.08570767 296.1880244 2.989210365 1.57976443 0.083149339 1chr10_126044589_126046785 178.8903929 89.68964918 268.0911367 2.989097841 1.579710121 0.095651041 1chr22_35512192_35513633 103.9045006 52.10541524 155.7035859 2.988241918 1.579296948 0.186801061 1chr1_51359204_51360914 85.15802751 42.70935675 127.6066983 2.987792558 1.579079985 0.22920279 1chr2_74134383_74135858 85.15802751 42.70935675 127.6066983 2.987792558 1.579079985 0.22920279 1chr3_66787777_66792756 728.9293014 365.5920938 1092.266509 2.987664469 1.579018135 0.010157752 1chr2_232638851_232639983 66.41155442 33.31329827 99.50981057 2.987089713 1.578740568 0.28751549 1chrX_118005797_118006894 66.41155442 33.31329827 99.50981057 2.987089713 1.578740568 0.28751549 1chr20_32458167_32459725 114.0766357 57.23053805 170.9227334 2.986565202 1.578487219 0.168674816 1chr12_65778434_65779345 47.66508133 23.91723978 71.41292288 2.985834634 1.578134266 0.371637711 1chr5_115638807_115640315 95.33016266 47.83447956 142.8258458 2.985834634 1.578134266 0.204926872 1chr9_80182746_80184753 95.33016266 47.83447956 142.8258458 2.985834634 1.578134266 0.204926872 1chr6_137598178_137599892 124.2487709 62.35566086 186.1418809 2.985164112 1.577810247 0.153001846 1chr7_131818960_131820751 124.2487709 62.35566086 186.1418809 2.985164112 1.577810247 0.153001846 1chr10_48450668_48454287 430.5835292 216.1093452 645.0577132 2.98486728 1.577666784 0.024461236 1chr12_112371732_112372867 76.58368957 38.43842108 114.7289581 2.984746898 1.577608598 0.253838959 1chr3_88131303_88132727 76.58368957 38.43842108 114.7289581 2.984746898 1.577608598 0.253838959 1chr8_144209047_144213487 536.085827 269.0689475 803.1027065 2.984746898 1.577608598 0.0169492 1chr6_116804022_116805789 105.5022978 52.95960237 158.0449933 2.984255662 1.577371137 0.184248625 1chr4_828308_830314 239.9232039 120.440386 359.4060217 2.984098885 1.577295343 0.062851733 1chr14_39540015_39541127 57.83721648 29.04236259 86.63207038 2.982955333 1.576742375 0.322430997 1chr6_140567087_140568425 86.75582473 43.56354389 129.9481056 2.982955333 1.576742375 0.225732909 1chr12_64861510_64864098 241.5210011 121.2945732 361.747429 2.982387584 1.576467759 0.06235342 1chr1_54924883_54926543 183.6837846 92.25221059 275.1153586 2.982208848 1.576381295 0.09286934 1chr17_16364799_16368176 300.9560148 151.1911229 450.7209067 2.981133403 1.575860936 0.044313435 1chrX_7992992_7994140 68.00935164 34.1674854 101.8512179 2.980939823 1.575767251 0.282611806 1chr1_64237374_64239712 214.2001901 107.627579 320.7728011 2.980395955 1.57550401 0.074744949 1chr12_3266461_3268521 146.1908384 73.46009361 218.9215833 2.980142993 1.575381556 0.12597209 1chr2_138467038_138468954 146.1908384 73.46009361 218.9215833 2.980142993 1.575381556 0.12597209 1chr6_51459123_51461103 117.2722302 58.93891232 175.6055481 2.979450098 1.575046084 0.164334547 1chr6_56268592_56270900 166.5351087 83.71033924 249.3598782 2.978842047 1.574751627 0.106479932 1chr12_74071192_74072729 127.4443653 64.06403513 190.8246956 2.978655578 1.574661314 0.149246872 1chr3_193101318_193103389 137.6165005 69.18915794 206.0438431 2.977978764 1.574333466 0.136090966 1chr5_71470782_71472623 98.5257571 49.54285383 147.5086604 2.977395304 1.574050779 0.199127569 1chr3_58341061_58343597 207.2236494 104.2108305 310.2364682 2.977007925 1.573863062 0.078730703 1chr3_61916073_61918860 217.3957845 109.3359533 325.4556157 2.976656863 1.573692923 0.073456886 1chr10_8419620_8421484 118.8700274 59.79309945 177.9469554 2.976045012 1.573396347 0.162227715 1chr2_239247259_239249473 237.7400548 119.5861989 355.8939107 2.976045012 1.573396347 0.064358051 1chr3_138297511_138300316 358.2078795 180.2334855 536.1822734 2.9749315 1.57285645 0.033729241 1chr12_95672326_95673918 79.77928402 40.14679535 119.4117727 2.974378693 1.572588341 0.245838057 1chr8_69827007_69828647 79.77928402 40.14679535 119.4117727 2.974378693 1.572588341 0.245838057 1chr12_65203907_65205562 349.6335415 175.9625498 523.3045332 2.973954025 1.572382344 0.035145609 1chr14_40645906_40647426 89.95141917 45.27191816 134.6309202 2.973828494 1.572321447 0.219031032 1chr5_168389339_168393327 410.6663525 206.7132867 614.6194182 2.973294209 1.572062226 0.026992895 1chr5_178644210_178645655 110.2956895 55.52216378 165.0692152 2.973032821 1.57193539 0.176901758 1chr5_18791782_18793400 120.4678246 60.64728659 180.2883627 2.972735844 1.571791272 0.160161623 1chr12_110162567_110168043 632.8555286 318.6118014 947.0992559 2.972580588 1.571715923 0.013192794 1chr1_87178852_87180950 140.8120949 70.89753221 210.7266577 2.9722707 1.571565515 0.132937117 1chr18_55194160_55196169 161.1563653 81.14777783 241.1649527 2.971923066 1.571396769 0.111947584 1chr6_43228406_43233769 876.9867723 441.6147488 1312.358796 2.971727732 1.571301943 0.007873297 1chr3_66689699_66693895 374.7712035 188.7753568 560.7670501 2.970552192 1.570731137 0.031534819 1chr12_11962827_11967094 466.3204199 234.9014621 697.7393776 2.970349232 1.570632563 0.021964553 1chr1_64296465_64298052 142.4098922 71.75171934 213.068065 2.969518597 1.570229068 0.131401647 1chr1_110996515_110997657 61.03281093 30.75073686 91.31488499 2.969518597 1.570229068 0.310877897 1chr10_4034550_4035754 71.20494608 35.87585967 106.5340325 2.969518597 1.570229068 0.273190667 1chr11_100322551_100323552 50.86067577 25.62561405 76.09573749 2.969518597 1.570229068 0.357171311 1chr2_233089738_233090971 61.03281093 30.75073686 91.31488499 2.969518597 1.570229068 0.310877897 1chr20_777008_778461 81.37708124 41.00098248 121.75318 2.969518597 1.570229068 0.241983287 1chr3_47995539_47997561 132.237757 66.62659653 197.8489175 2.969518597 1.570229068 0.143871205 1chr3_169013535_169015083 111.8934867 56.37635091 167.4106225 2.969518597 1.570229068 0.17455157 1chr7_12999162_13000219 61.03281093 30.75073686 91.31488499 2.969518597 1.570229068 0.310877897 1chr9_98219921_98222403 233.9591086 117.8778246 350.0403925 2.969518597 1.570229068 0.066450333 1chrX_149533625_149535215 101.7213515 51.2512281 152.191475 2.969518597 1.570229068 0.193575195 1chrY_2350035_2351932 122.0656219 61.50147372 182.62977 2.969518597 1.570229068 0.158135221 1chr9_129995906_130001751 672.9587174 339.1122926 1006.805142 2.968943221 1.569949503 0.012028664 1chr3_31830442_31833987 337.2782573 169.9832399 504.5732748 2.968370735 1.569671289 0.037580685 1chr12_65049522_65051156 316.933987 159.7329943 474.1349798 2.968297076 1.569635488 0.04154821 1chr7_483791_486045 255.9011761 128.9822574 382.8200948 2.968005852 1.569493937 0.058135324 1chr3_51579284_51585224 471.1138116 237.4640235 704.7635996 2.967875256 1.569430454 0.021690083 1chr20_19672590_19674502 144.0076894 72.60590648 215.4094723 2.96683125 1.56892287 0.129892962 1chr2_206047326_206049150 133.8355542 67.48078367 200.1903248 2.966627148 1.568823617 0.142144475 1chr8_83465701_83467396 133.8355542 67.48078367 200.1903248 2.966627148 1.568823617 0.142144475 1chr3_66652370_66654642 247.3268382 124.7113217 369.9423546 2.966389494 1.56870804 0.061357254 1chr5_169371828_169374333 237.154703 119.5861989 354.7232071 2.96625539 1.568642817 0.065374884 1chr20_2781513_2782978 103.3191488 52.10541524 154.5328823 2.965773933 1.568408632 0.190887139 1chr3_59562717_59564141 103.3191488 52.10541524 154.5328823 2.965773933 1.568408632 0.190887139 1chr4_124280296_124281741 93.14701361 46.98029243 139.3137348 2.965365424 1.5682099 0.212629856 1chr11_60013840_60015158 82.97487846 41.85516962 124.0945873 2.964856873 1.567962461 0.238221241 1chr2_8551933_8554412 145.6054866 73.46009361 217.7508796 2.964206399 1.567645907 0.128410446 1chr12_85833631_85834715 62.63060815 31.604924 93.6562923 2.963344962 1.56722658 0.305347628 1chr6_41493988_41497952 1455.469514 734.6009361 2176.338092 2.96261274 1.566870056 0.003967206 0.893973995chr6_47359046_47360658 104.916946 52.95960237 156.8742896 2.962150065 1.566644731 0.188255589 1chr8_56918635_56920644 147.2032838 74.31428075 220.0922869 2.96164189 1.566397206 0.126953501 1chr14_99713681_99715026 241.9480947 122.1487603 361.747429 2.961531726 1.566343542 0.063812156 1chr22_19889014_19894313 664.8114731 335.6955441 993.927402 2.960800105 1.565987092 0.012479664 1chr12_2033122_2036479 348.4628379 175.9625498 520.9631259 2.960647743 1.565912849 0.036067355 1chr10_123298959_123301319 169.1453514 85.41871351 252.8719892 2.960381617 1.565783163 0.106192889 1chr12_90271423_90272852 84.57267568 42.70935675 126.4359946 2.960381617 1.565783163 0.234548812 1chr2_169534415_169535629 84.57267568 42.70935675 126.4359946 2.960381617 1.565783163 0.234548812 1chr7_55909139_55910717 126.8590135 64.06403513 189.6539919 2.960381617 1.565783163 0.152284159 1chr7_18464902_18467662 243.5458919 123.0029474 364.0888363 2.96000091 1.565597619 0.063304259 1chr2_129338292_129340093 116.6868784 58.93891232 174.4348444 2.959587097 1.565395914 0.167778883 1chr8_99489682_99491200 116.6868784 58.93891232 174.4348444 2.959587097 1.565395914 0.167778883 1chr8_128687752_128689328 116.6868784 58.93891232 174.4348444 2.959587097 1.565395914 0.167778883 1chr1_243397617_243400459 191.0874189 96.52314626 285.6516915 2.959411318 1.565310226 0.090037124 1chr1_93460266_93462634 255.3158243 128.9822574 381.6493911 2.958929382 1.565075265 0.059021483 1chr7_140861084_140862639 138.6289459 70.04334507 207.2145467 2.958375938 1.564805396 0.137148936 1chr1_87238424_87241938 299.1999593 151.1911229 447.2087957 2.957903792 1.564575128 0.046215295 1chr7_27117842_27122170 556.1135808 281.0275674 831.1995942 2.957715507 1.564483291 0.016801006 1chr18_1542604_1544799 160.5710134 81.14777783 239.994249 2.957496255 1.564376342 0.114031761 1chr22_27349168_27350802 96.34260805 48.6886667 143.9965494 2.957496255 1.564376342 0.206511015 1chr5_16677843_16679414 96.34260805 48.6886667 143.9965494 2.957496255 1.564376342 0.206511015 1chr7_139242520_139244121 96.34260805 48.6886667 143.9965494 2.957496255 1.564376342 0.206511015 1chr3_16545515_16547411 182.513081 92.25221059 272.7739513 2.956828347 1.564050493 0.096183846 1chr10_44276919_44278185 86.1704729 43.56354389 128.7774019 2.956081862 1.563686222 0.23096303 1chr12_107624605_107626018 86.1704729 43.56354389 128.7774019 2.956081862 1.563686222 0.23096303 1chr4_186444757_186446823 140.2267431 70.89753221 209.555954 2.955758085 1.563528196 0.135542883 1chr8_21841632_21843567 140.2267431 70.89753221 209.555954 2.955758085 1.563528196 0.135542883 1chr18_2766089_2768434 270.2813511 136.6699416 403.8927605 2.955242066 1.563276307 0.054351627 1chr19_2003025_2008986 368.2217564 186.2127954 550.2307173 2.954849134 1.563084472 0.033277489 1chr15_61992482_61995698 260.1092159 131.5448188 388.673613 2.954685837 1.563004741 0.057686744 1chr4_99603581_99605313 130.054608 65.7724094 194.3368065 2.954685837 1.563004741 0.148564133 1chr8_12522392_12523799 130.054608 65.7724094 194.3368065 2.954685837 1.563004741 0.148564133 1chr6_40377014_40378123 206.0529457 104.2108305 307.8950609 2.954539941 1.562933502 0.08139736 1chr6_68121743_68123165 75.99833775 38.43842108 113.5582544 2.954290297 1.562811596 0.259953853 1chr7_11430528_11431614 75.99833775 38.43842108 113.5582544 2.954290297 1.562811596 0.259953853 1chr7_2507872_2509348 119.8824728 60.64728659 179.117659 2.953432364 1.562392574 0.163483169 1chr3_71611218_71614718 305.5911482 154.6078714 456.574425 2.953112417 1.562236277 0.045003477 1chr7_56084777_56088743 295.4190131 149.4827486 441.3552775 2.95254992 1.561961452 0.04750513 1chr1_54800732_54804433 383.1872832 193.9004797 572.4740867 2.952411916 1.561894018 0.031308436 1chr3_123108523_123113013 427.0714182 216.1093452 638.0334913 2.952364188 1.561870695 0.026215137 1chr11_66468803_66470417 131.6524052 66.62659653 196.6782138 2.951947481 1.561667054 0.146755649 1chr12_59336510_59338148 131.6524052 66.62659653 196.6782138 2.951947481 1.561667054 0.146755649 1chr12_94826988_94828340 65.82620259 33.31329827 98.33910692 2.951947481 1.561667054 0.294746001 1chr14_58910826_58912405 87.76827012 44.41773102 131.1188092 2.951947481 1.561667054 0.227461061 1chr2_1927124_1928258 65.82620259 33.31329827 98.33910692 2.951947481 1.561667054 0.294746001 1chr2_18972958_18974654 131.6524052 66.62659653 196.6782138 2.951947481 1.561667054 0.146755649 1chr2_204049974_204051410 87.76827012 44.41773102 131.1188092 2.951947481 1.561667054 0.227461061 1chr5_10662153_10665228 307.1889454 155.4620586 458.9158323 2.951947481 1.561667054 0.04470855 1chr6_42826457_42827528 65.82620259 33.31329827 98.33910692 2.951947481 1.561667054 0.294746001 1chr7_111180085_111181903 109.7103377 55.52216378 163.8985115 2.951947481 1.561667054 0.180684202 1chr7_44372583_44374735 197.4786078 99.9398948 295.0173207 2.951947481 1.561667054 0.086686918 1chr21_16526255_16528332 143.4223376 72.60590648 214.2387686 2.950707167 1.561060753 0.13241533 1chr2_234307667_234312328 431.8648099 218.6719066 645.0577132 2.949888366 1.560660359 0.025848454 1chrX_71765015_71766881 133.2502024 67.48078367 199.0196211 2.949278451 1.560362038 0.144980394 1chr4_77521150_77523537 656.0788769 332.2787955 979.8789582 2.948966264 1.560209318 0.013068246 1chr7_2067987_2073365 1079.369349 546.6797664 1612.058931 2.948817626 1.5601366 0.006129918 1chr4_152448146_152452879 367.6364045 186.2127954 549.0600136 2.94856222 1.560011638 0.033697519 1chr7_5003709_5005030 89.36606734 45.27191816 133.4602165 2.947969115 1.55972141 0.22404019 1chr8_120365628_120366860 89.36606734 45.27191816 133.4602165 2.947969115 1.55972141 0.22404019 1chr2_208201973_208203743 123.0780672 62.35566086 183.8004736 2.947614877 1.55954804 0.159352398 1chr19_17912343_17915318 291.6380668 147.7743744 435.5017592 2.947072258 1.559282434 0.04884421 1chr2_1868272_1870064 168.5599995 85.41871351 251.7012856 2.946676147 1.55908851 0.108128339 1chr21_44860015_44861920 134.8479996 68.3349708 201.3610284 2.946676147 1.55908851 0.143237557 1chr3_41694347_41695730 67.42399981 34.1674854 100.6805142 2.946676147 1.55908851 0.289662343 1chr11_60542222_60545565 283.0637288 143.5034387 422.624019 2.945044543 1.558289455 0.051306871 1chr22_20995002_20996747 124.6758645 63.20984799 186.1418809 2.944824056 1.55818144 0.157346218 1chr2_134347066_134350807 352.0855259 178.5251112 525.6459405 2.944380972 1.557964353 0.036371038 1chr20_24715293_24718473 284.6615261 144.3576258 424.9654263 2.943837736 1.557698152 0.050945497 1chr5_31866445_31868887 239.1795938 121.2945732 357.0646144 2.943780625 1.557670163 0.066359102 1chr1_66428566_66429635 57.25186466 29.04236259 85.46136672 2.942645126 1.557113568 0.330864802 1chr1_81983468_81984515 57.25186466 29.04236259 85.46136672 2.942645126 1.557113568 0.330864802 1chr21_15351471_15352477 57.25186466 29.04236259 85.46136672 2.942645126 1.557113568 0.330864802 1chr8_36582983_36584346 114.5037293 58.08472518 170.9227334 2.942645126 1.557113568 0.17356694 1chr20_38960030_38961433 69.02179703 35.02167254 103.0219215 2.94166195 1.556631464 0.284715831 1chr1_172188732_172189983 80.79172941 41.00098248 120.5824763 2.940965534 1.556289877 0.247690276 1chr3_58257200_58258358 80.79172941 41.00098248 120.5824763 2.940965534 1.556289877 0.247690276 1chr7_20029185_20031334 161.5834588 82.00196497 241.1649527 2.940965534 1.556289877 0.114901013 1chr18_46392153_46393690 104.3315942 52.95960237 155.7035859 2.940044467 1.555837976 0.192341461 1chr2_232247819_232249181 104.3315942 52.95960237 155.7035859 2.940044467 1.555837976 0.192341461 1chr11_64529378_64533735 1102.165603 559.4925735 1644.838634 2.939875722 1.555755169 0.00608807 1chr7_55274954_55278792 513.0836329 260.5270762 765.6401896 2.938812352 1.555233244 0.019877102 1chr6_42292950_42294822 198.4910532 100.7940819 296.1880244 2.938545783 1.555102376 0.087339996 1chr4_6094861_6097966 257.340715 130.6906317 383.9907984 2.938166214 1.554916012 0.059898576 1chr6_138297420_138301248 410.3498359 208.421661 612.2780109 2.937688953 1.554681649 0.028690933 1chr10_51503771_51505471 153.0091209 77.73102929 228.2872125 2.936886525 1.554287524 0.123663679 1chr12_105493936_105496989 211.8587828 107.627579 316.0899865 2.936886525 1.554287524 0.079817287 1chr17_75111917_75113731 105.9293914 53.81378951 158.0449933 2.936886525 1.554287524 0.189686002 1chr19_19736005_19738785 282.478377 143.5034387 421.4533154 2.936886525 1.554287524 0.052048412 1chr2_223910560_223913903 211.8587828 107.627579 316.0899865 2.936886525 1.554287524 0.079817287 1chr7_21919941_21921077 70.61959426 35.87585967 105.3633288 2.936886525 1.554287524 0.279901139 1chr1_81918298_81920092 119.297121 60.64728659 177.9469554 2.934128885 1.552932244 0.16686766 1chr14_36537736_36540217 226.8243096 115.3152632 338.3333559 2.933985896 1.552861936 0.072593888 1chr8_123628768_123630230 107.5271886 54.66797664 160.3864006 2.933827268 1.552783933 0.187086065 1chr15_90027687_90030480 203.2844448 103.3566433 303.2122463 2.933650286 1.552696901 0.084929953 1chr3_48776887_48778363 95.75725623 48.6886667 142.8258458 2.93345157 1.552599174 0.211117426 1chr6_43790640_43796539 959.1703595 487.7408541 1430.599865 2.933114692 1.552433485 0.007548369 1chr4_48916208_48917674 83.98732385 42.70935675 125.265291 2.932970676 1.552362647 0.240005948 1chr2_79745844_79747419 72.21739148 36.73004681 107.7047361 2.932333212 1.552049052 0.275213227 1chr3_66436969_66438417 120.8949182 61.50147372 180.2883627 2.931447846 1.55161339 0.16472174 1chrX_100085768_100086994 60.4474591 30.75073686 90.14418134 2.931447846 1.55161339 0.318868014 1chr2_191064169_191066370 109.1249858 55.52216378 162.7278079 2.930862142 1.55132511 0.184540079 1chr1_19373356_19374245 48.67752672 24.77142692 72.58362653 2.930135062 1.550967166 0.37424422 1chr1_47448354_47450902 292.0651603 148.6285615 435.5017592 2.930135062 1.550967166 0.049896801 1chr15_67032319_67035153 292.0651603 148.6285615 435.5017592 2.930135062 1.550967166 0.049896801 1chr2_119035977_119037540 97.35505345 49.54285383 145.1672531 2.930135062 1.550967166 0.20806525 1chr8_56608826_56611017 231.6177012 117.8778246 345.3575779 2.929792596 1.550798538 0.070772506 1chr8_11266813_11269826 268.5252956 136.6699416 400.3806496 2.929544309 1.55067627 0.056801394 1chr8_37317085_37318975 219.8477689 111.8985147 327.7970231 2.929413531 1.550611866 0.076358506 1chr19_19861826_19863114 85.58512107 43.56354389 127.6066983 2.92920839 1.550510833 0.236301266 1chr2_232768404_232771360 244.9854308 124.7113217 365.25954 2.92884026 1.55032951 0.065242753 1chr1_84401570_84403065 73.8151887 37.58423394 110.0461435 2.927986869 1.549909083 0.270647325 1chr1_100471196_100472655 110.722783 56.37635091 165.0692152 2.927986869 1.549909083 0.182046531 1chr4_1630500_1631662 73.8151887 37.58423394 110.0461435 2.927986869 1.549909083 0.270647325 1chr17_63549767_63551840 172.7680394 87.98127491 257.5548038 2.927382038 1.549611037 0.106691975 1chr1_61643233_61645834 321.996214 164.0039299 479.988498 2.926689002 1.549269449 0.04306351 1chr4_1993223_1996061 321.996214 164.0039299 479.988498 2.926689002 1.549269449 0.04306351 1chr1_91491100_91492787 124.0905126 63.20984799 184.9711773 2.92630315 1.549079233 0.160553871 1chr16_84958793_84959901 62.04525632 31.604924 92.48558865 2.92630315 1.549079233 0.313129371 1chr21_33753762_33756080 186.135769 94.81477199 277.4567659 2.92630315 1.549079233 0.096677809 1chr5_149020041_149021185 62.04525632 31.604924 92.48558865 2.92630315 1.549079233 0.313129371 1chr7_151856822_151857701 62.04525632 31.604924 92.48558865 2.92630315 1.549079233 0.313129371 1chr5_32056476_32058362 149.2281746 76.02265502 222.4336942 2.92588695 1.548874028 0.128888861 1chr2_204984432_204985816 87.18291829 44.41773102 129.9481056 2.925590807 1.548727999 0.232684173 1chr2_202422652_202424713 238.0088901 121.2945732 354.7232071 2.924477145 1.548178714 0.068452912 1chr6_41700243_41704385 476.0177803 242.5891464 709.4464142 2.924477145 1.548178714 0.023062183 1chr12_66212058_66213690 125.6883099 64.06403513 187.3125846 2.923833696 1.547861255 0.158529791 1chr2_3181748_3183640 226.2389578 115.3152632 337.1626523 2.923833696 1.547861255 0.073756295 1chr5_170852803_170854048 100.5506479 51.2512281 149.8500677 2.923833696 1.547861255 0.202160101 1chr8_8373542_8375416 125.6883099 64.06403513 187.3125846 2.923833696 1.547861255 0.158529791 1chr18_10465390_10467084 139.0560395 70.89753221 207.2145467 2.922732855 1.547317969 0.140896687 1chr7_55224610_55229432 594.7295494 303.2364329 886.2226659 2.922546797 1.547226126 0.016151503 1chr2_109938310_109939843 88.78071552 45.27191816 132.2895129 2.922109737 1.547010358 0.229151793 1chr2_158657071_158658491 88.78071552 45.27191816 132.2895129 2.922109737 1.547010358 0.229151793 1chr5_73335373_73337628 177.561431 90.54383632 264.5790257 2.922109737 1.547010358 0.103445679 1chr8_145954709_145956397 152.4237691 77.73102929 227.1165088 2.921825568 1.546870052 0.12599754 1chr8_125022443_125023542 63.64305354 32.45911113 94.82699595 2.921429227 1.54667434 0.307554091 1chr12_96496043_96497761 140.6538367 71.75171934 209.555954 2.920570489 1.546250205 0.139231812 1chr11_119620219_119622468 179.1592283 91.39802345 266.9204331 2.920418002 1.546174878 0.102397119 1chr2_134215200_134216591 77.01078314 39.29260821 114.7289581 2.919861096 1.545899739 0.261863677 1chr8_54628507_54629987 115.5161747 58.93891232 172.0934371 2.919861096 1.545899739 0.174866186 1chr12_95063231_95066904 321.4108622 164.0039299 478.8177944 2.919550736 1.545746383 0.043644415 1chr21_33387624_33389248 128.8839043 65.7724094 191.9953992 2.919087213 1.545517314 0.154596277 1chr8_28538201_28539850 128.8839043 65.7724094 191.9953992 2.919087213 1.545517314 0.154596277 1chr17_17464742_17465988 271.1355382 138.3783159 403.8927605 2.918757596 1.545354399 0.056785377 1chr8_18768061_18770730 180.7570255 92.25221059 269.2618404 2.918757596 1.545354399 0.101364759 1chr1_26614625_26618590 555.638806 283.5901288 827.6874832 2.918604701 1.545278824 0.018156876 1chr8_11644344_11648647 374.8817805 191.3379183 558.4256428 2.918530984 1.545242384 0.034258857 1chr5_136839582_136840557 51.87312117 26.47980119 77.26644115 2.91793887 1.544949659 0.359670111 1chr5_1944091_1946130 168.9870931 86.27290064 251.7012856 2.917501135 1.544733217 0.110916308 1chr6_42331330_42336174 808.0278711 412.5723862 1203.483356 2.917023524 1.544497021 0.010118063 1chr2_205208072_205209402 91.97630996 46.98029243 136.9723275 2.91552735 1.543756857 0.222330286 1chr8_134274418_134279397 593.5588458 303.2364329 883.8812586 2.914825405 1.54340947 0.016456295 1chr2_128393246_128396454 409.6062258 209.2758481 609.9366036 2.914510247 1.543253474 0.029920619 1chr22_37366554_37368516 172.1826875 87.98127491 256.3841002 2.914075756 1.543038383 0.108608155 1chr1_54730812_54733465 253.9868623 129.8364445 378.1372802 2.91241247 1.542214692 0.06314895 1chr1_65969530_65970547 53.47091839 27.33398832 79.60784846 2.91241247 1.542214692 0.352730021 1chr1_77877587_77879017 80.20637758 41.00098248 119.4117727 2.91241247 1.542214692 0.253516673 1chr16_27527033_27532092 561.4446431 287.0068774 835.8824088 2.91241247 1.542214692 0.018074273 1chr2_139039287_139040716 106.9418368 54.66797664 159.2156969 2.91241247 1.542214692 0.191092897 1chr3_72936222_72937241 66.83864799 34.1674854 99.50981057 2.91241247 1.542214692 0.296866797 1chr8_58998687_59000524 213.8836736 109.3359533 318.4313938 2.91241247 1.542214692 0.080996289 1chr8_57682268_57684851 227.2514032 116.1694504 338.3333559 2.91241247 1.542214692 0.07429332 1chr17_4621902_4626892 616.5133587 315.1950528 917.8316645 2.911948193 1.541984689 0.015582352 1chr1_90341328_90344383 175.378282 89.68964918 261.0669148 2.910780867 1.541406233 0.106372662 1chr8_136815699_136817941 148.6428228 76.02265502 221.2629906 2.910487545 1.541260844 0.131339676 1chr12_105391302_105395916 554.4681024 283.5901288 825.3460759 2.910348394 1.541191866 0.018514773 1chr1_85003039_85008193 352.3543612 180.2334855 524.4752369 2.909976664 1.541007584 0.038320157 1chr4_140827753_140829426 108.539634 55.52216378 161.5571042 2.909776803 1.540908494 0.188470567 1chr1_111139059_111140482 95.1719044 48.6886667 141.6551421 2.909406885 1.540725074 0.215816129 1chr6_41640717_41644278 340.5844288 174.2541756 506.9146821 2.909053287 1.540549723 0.040485053 1chr1_26465012_26466504 81.8041748 41.85516962 121.75318 2.908916177 1.540481724 0.249497329 1chr1_54746835_54749284 232.0447948 118.7320118 345.3575779 2.908714951 1.540381922 0.072414903 1chr8_143104548_143108224 562.4570885 287.8610645 837.0531124 2.907837202 1.539946501 0.018185087 1chrX_131809672_131812492 178.5738764 91.39802345 265.7497294 2.907609151 1.539833351 0.10420684 1chrX_4464641_4466223 110.1374312 56.37635091 163.8985115 2.907221004 1.539640748 0.185902744 1chr17_54571515_54574151 373.7110769 191.3379183 556.0842355 2.906293957 1.539180632 0.035114322 1chr1_71484755_71487247 166.8039441 85.41871351 248.1891746 2.905559735 1.538816115 0.11413443 1chr15_93689055_93690388 83.40197203 42.70935675 124.0945873 2.905559735 1.538816115 0.245576013 1chr19_5147380_5150726 278.5391725 142.6492516 414.4290934 2.90523146 1.538653108 0.055523565 1chr15_43178726_43180994 153.4362145 78.58521643 228.2872125 2.904963845 1.538520208 0.12692666 1chr1_3704606_3707851 265.1714429 135.8157545 394.5271313 2.904870152 1.538473676 0.059810933 1chr1_68064278_68065696 111.7352284 57.23053805 166.2399188 2.904741498 1.53840978 0.183387896 1chr11_16827397_16828989 98.36749884 50.39704097 146.3379567 2.903701366 1.537893086 0.209590416 1chr11_74321783_74324883 268.3670373 137.5241287 399.2099459 2.902835667 1.537462901 0.058908299 1chr1_103216390_103217468 56.66651283 29.04236259 84.29066307 2.902334919 1.537214011 0.339489104 1chr10_49671291_49672610 254.9993077 130.6906317 379.3079838 2.902334919 1.537214011 0.063599552 1chr18_48507474_48509676 169.9995385 87.12708778 252.8719892 2.902334919 1.537214011 0.111729855 1chr2_212096745_212098254 84.99976925 43.56354389 126.4359946 2.902334919 1.537214011 0.241749376 1chr6_148742936_148743983 56.66651283 29.04236259 84.29066307 2.902334919 1.537214011 0.339489104 1chr7_11050075_11054184 311.6658206 159.7329943 463.5986469 2.902334919 1.537214011 0.046955038 1chr2_45439317_45442034 284.9303614 146.0660001 423.7947227 2.90139199 1.536745223 0.053932959 1chr13_50984629_50987011 199.9305921 102.5024562 297.3587281 2.900991245 1.536545942 0.09012834 1chr19_716941_719739 243.2293754 124.7113217 361.747429 2.900678335 1.536390319 0.068309922 1chr4_120243164_120244444 71.63203965 36.73004681 106.5340325 2.900460025 1.536281736 0.281903286 1chr6_135517600_135518761 71.63203965 36.73004681 106.5340325 2.900460025 1.536281736 0.281903286 1chr7_36217255_36219030 143.2640793 73.46009361 213.068065 2.900460025 1.536281736 0.138607899 1chr7_68531806_68533045 71.63203965 36.73004681 106.5340325 2.900460025 1.536281736 0.281903286 1chr5_145327855_145329396 114.9308229 58.93891232 170.9227334 2.899998095 1.536051953 0.178511266 1chr20_3270654_3274747 273.160429 140.0866901 406.2341679 2.899876979 1.535991698 0.05759428 1chr11_114301943_114304502 201.5283894 103.3566433 299.7001354 2.899669781 1.535888613 0.089279477 1chr11_59686397_59688135 86.59756647 44.41773102 128.7774019 2.899234133 1.535671846 0.238014223 1chr20_19829607_19831734 173.1951329 88.83546205 257.5548038 2.899234133 1.535671846 0.109401855 1chr5_25362103_25363789 129.8963497 66.62659653 193.1661029 2.899234133 1.535671846 0.155731413 1chr7_499439_500810 389.6890491 199.8797896 579.4983086 2.899234133 1.535671846 0.033235887 1chr1_59685668_59686634 58.26431005 29.89654973 86.63207038 2.897728038 1.534922199 0.333168634 1chr11_94650281_94651338 58.26431005 29.89654973 86.63207038 2.897728038 1.534922199 0.333168634 1chr7_28419260_28420351 58.26431005 29.89654973 86.63207038 2.897728038 1.534922199 0.333168634 1chr5_71461150_71465704 364.5513871 187.0669826 542.0357917 2.897549232 1.534833174 0.037034013 1chr14_35563585_35565588 146.4596737 75.16846788 217.7508796 2.896838072 1.534479043 0.13539686 1chr20_31078378_31079625 73.22983687 37.58423394 108.8754398 2.896838072 1.534479043 0.277178208 1chr12_92210573_92213629 249.6205642 128.1280703 371.1130582 2.896422755 1.53427219 0.066125545 1chrX_154032134_154033931 176.3907274 90.54383632 262.2376184 2.896250359 1.534186318 0.107147241 1chr1_87081431_87083404 118.1264173 60.64728659 175.6055481 2.895521926 1.533823421 0.173829483 1chr2_173541354_173543325 118.1264173 60.64728659 175.6055481 2.895521926 1.533823421 0.173829483 1chr1_59319200_59320940 133.0919441 68.3349708 197.8489175 2.895280632 1.533703192 0.151912282 1chr2_159572409_159574542 163.0229978 83.71033924 242.3356563 2.894931003 1.533528964 0.118751937 1chr13_95886802_95888849 177.9885246 91.39802345 264.5790257 2.8948003 1.533463826 0.106046501 1chr8_10612759_10620687 1410.540467 724.3506905 2096.730244 2.894634148 1.533381018 0.004961985 0.965381333chr8_11211536_11213456 207.9195782 106.7733919 309.0657646 2.894595359 1.533361685 0.086004449 1chr1_196786237_196787413 89.79316091 46.12610529 133.4602165 2.893377095 1.532754362 0.230806307 1chr10_45324482_45326109 134.6897414 69.18915794 200.1903248 2.893377095 1.532754362 0.150055895 1chr10_89318017_89319146 59.86210727 30.75073686 88.97347769 2.893377095 1.532754362 0.327036139 1chr2_130578502_130579658 59.86210727 30.75073686 88.97347769 2.893377095 1.532754362 0.327036139 1chr20_2235678_2239381 284.3450095 146.0660001 422.624019 2.893377095 1.532754362 0.054703356 1chr3_50938272_50939368 59.86210727 30.75073686 88.97347769 2.893377095 1.532754362 0.327036139 1chr3_160678569_160679751 59.86210727 30.75073686 88.97347769 2.893377095 1.532754362 0.327036139 1chr4_169851628_169852782 59.86210727 30.75073686 88.97347769 2.893377095 1.532754362 0.327036139 1chr5_169260237_169262170 256.0117531 131.5448188 380.4786875 2.892388244 1.532261218 0.064048989 1chr1_107421647_107423690 211.1151727 108.4817662 313.7485792 2.892178016 1.532156354 0.084436065 1chr3_30912511_30914384 121.3220118 62.35566086 180.2883627 2.891291026 1.531713832 0.169332137 1chr1_47719353_47721262 106.356485 54.66797664 158.0449933 2.890997673 1.531567448 0.195177356 1chr2_214063958_214065529 106.356485 54.66797664 158.0449933 2.890997673 1.531567448 0.195177356 1chr5_74730727_74732194 91.39095813 46.98029243 135.8016238 2.890608313 1.531373132 0.227327848 1chr7_22450965_22452400 91.39095813 46.98029243 135.8016238 2.890608313 1.531373132 0.227327848 1chr2_196952251_196953613 76.42543131 39.29260821 113.5582544 2.890066595 1.531102737 0.268092808 1chr3_194899536_194901982 152.8508626 78.58521643 227.1165088 2.890066595 1.531102737 0.129305224 1chr7_107224321_107226106 152.8508626 78.58521643 227.1165088 2.890066595 1.531102737 0.129305224 1chr8_117838687_117840210 76.42543131 39.29260821 113.5582544 2.890066595 1.531102737 0.268092808 1chr2_164419959_164421090 61.45990449 31.604924 91.31488499 2.889261338 1.530700704 0.321083332 1chr7_27052026_27053392 107.9542822 55.52216378 160.3864006 2.888691464 1.53041612 0.192476852 1chr3_72327428_72329699 200.9430375 103.3566433 298.5294317 2.888342946 1.53024205 0.09077545 1chr1_7494792_7496428 92.98875535 47.83447956 138.1430311 2.887938416 1.530039978 0.223929466 1chr10_71672327_71673246 46.49437768 23.91723978 69.07151557 2.887938416 1.530039978 0.392408476 1chr5_75842673_75845372 171.0119839 87.98127491 254.0426929 2.887463192 1.529802556 0.112537446 1chr19_2794945_2796452 78.02322853 40.14679535 115.8996617 2.886896967 1.529519619 0.263723573 1chr1_47855649_47857262 109.5520794 56.37635091 162.7278079 2.88645514 1.529298804 0.189832985 1chr14_34047327_34048673 298.7251845 153.7536843 443.6966848 2.885762945 1.528952792 0.051313575 1chr2_169556053_169558049 126.1154034 64.91822226 187.3125846 2.8853622 1.528752432 0.1629109 1chr1_197927168_197928718 142.6787275 73.46009361 211.8973613 2.884523432 1.528332983 0.141271716 1chr2_25285027_25286710 111.1498766 57.23053805 165.0692152 2.884285572 1.528214012 0.187244145 1chr2_228154188_228155633 111.1498766 57.23053805 165.0692152 2.884285572 1.528214012 0.187244145 1chr1_165784539_165785751 79.62102576 41.00098248 118.241069 2.883859407 1.528000832 0.259464413 1chr2_159759756_159761836 127.7132007 65.7724094 189.6539919 2.883488588 1.527815313 0.160852611 1chr9_117549466_117552458 240.4608745 123.8571346 357.0646144 2.882874819 1.527508193 0.071018929 1chr21_44180858_44182748 160.8398487 82.8561521 238.8235454 2.8823876 1.52726435 0.122259952 1chr2_220355008_220356053 64.65549894 33.31329827 95.99769961 2.881663018 1.526901636 0.309686172 1chr2_232150536_232152442 129.3109979 66.62659653 191.9953992 2.881663018 1.526901636 0.158833623 1chr22_28210993_28212048 64.65549894 33.31329827 95.99769961 2.881663018 1.526901636 0.309686172 1chr8_70589395_70590496 64.65549894 33.31329827 95.99769961 2.881663018 1.526901636 0.309686172 1chr5_1199602_1205658 1213.488953 625.2649829 1801.712923 2.881518992 1.526829528 0.006213486 1chr17_1777955_1779478 97.78214702 50.39704097 145.1672531 2.880471755 1.526305111 0.214189828 1chr3_52670121_52672156 147.4721191 76.02265502 218.9215833 2.879688735 1.525912879 0.136371332 1chr7_7303600_7306709 311.5075623 160.5871814 462.4279432 2.879606823 1.525871842 0.048574513 1chr1_230838258_230840467 182.1965644 93.96058486 270.432544 2.878148794 1.525141178 0.104638687 1chr2_134850343_134851165 49.68997212 25.62561405 73.75433019 2.878148794 1.525141178 0.376727174 1chr2_170141815_170142925 66.25329616 34.1674854 98.33910692 2.878148794 1.525141178 0.304227667 1chr2_198489279_198490626 82.8166202 42.70935675 122.9238836 2.878148794 1.525141178 0.251260833 1chr22_23721662_23723120 149.0699164 76.87684216 221.2629906 2.878148794 1.525141178 0.134795227 1chr4_96111438_96112865 99.37994424 51.2512281 147.5086604 2.878148794 1.525141178 0.211087322 1chr7_26281849_26284946 231.8865366 119.5861989 344.1868742 2.878148794 1.525141178 0.075241937 1chr7_135279027_135280629 115.9432683 59.79309945 172.0934371 2.878148794 1.525141178 0.179792543 1chr8_19136004_19138083 149.0699164 76.87684216 221.2629906 2.878148794 1.525141178 0.134795227 1chrX_118188997_118190807 115.9432683 59.79309945 172.0934371 2.878148794 1.525141178 0.179792543 1chr2_232233942_232236386 233.4843338 120.440386 346.5282815 2.877176775 1.524653864 0.074599882 1chr2_220401416_220404662 485.1297888 250.2768306 719.9827471 2.876745504 1.524437596 0.024414313 1chr12_97788169_97790619 150.6677136 77.73102929 223.6043979 2.876642699 1.524386038 0.133246686 1chr4_77940015_77946067 1004.984023 518.491591 1491.476455 2.876568262 1.524348706 0.008109812 1chrX_68251257_68254457 235.082131 121.2945732 348.8696888 2.876218446 1.524173251 0.073966331 1chr12_104805284_104806249 168.8288348 87.12708778 250.5305819 2.875461447 1.523793495 0.115783815 1chr8_56560373_56562131 152.2655108 78.58521643 225.9458052 2.875169344 1.523646932 0.131725071 1chr10_48370117_48371723 102.5755387 52.95960237 152.191475 2.873727675 1.522923353 0.205085325 1chr7_36157733_36160871 496.3143694 256.2561405 736.3725982 2.873580304 1.522849367 0.023689754 1chr10_74559571_74562205 172.0244293 88.83546205 255.2133965 2.87287746 1.522496457 0.113339142 1chr1_85378339_85380582 190.1855505 98.23152053 282.1395806 2.872189894 1.522151136 0.09950484 1chr4_148560988_148563369 190.1855505 98.23152053 282.1395806 2.872189894 1.522151136 0.09950484 1chr7_25991118_25993637 259.6344411 134.1073802 385.1615021 2.872038075 1.522074875 0.064432896 1chr1_93350410_93353770 277.7955624 143.5034387 412.0876861 2.87162238 1.521866046 0.0583631 1chr4_142109614_142110886 69.4488906 35.87585967 103.0219215 2.87162238 1.521866046 0.29375849 1chr10_7336935_7340754 279.3933596 144.3576258 414.4290934 2.870850023 1.521477964 0.057929834 1chr11_112225773_112227322 122.3344572 63.20984799 181.4590663 2.870740432 1.52142289 0.170539503 1chr2_205559156_205560853 87.61001186 45.27191816 129.9481056 2.870390981 1.521247262 0.239688995 1chr10_32173757_32179920 474.3723018 245.1517078 703.5928959 2.870030571 1.521066104 0.025607539 1chr1_51668004_51670137 158.6566997 82.00196497 235.3114344 2.869582875 1.520841041 0.125895733 1chr3_55539267_55540749 105.7711331 54.66797664 156.8742896 2.869582875 1.520841041 0.19934067 1chr5_135236392_135238515 158.6566997 82.00196497 235.3114344 2.869582875 1.520841041 0.125895733 1chr6_12233234_12235362 158.6566997 82.00196497 235.3114344 2.869582875 1.520841041 0.125895733 1chr10_72134010_72139155 636.224596 328.862047 943.5871449 2.869249138 1.520673243 0.016202008 1chr7_34127726_34128530 123.9322544 64.06403513 183.8004736 2.869011814 1.520553909 0.168339481 1chr1_14260938_14262411 71.04668782 36.73004681 105.3633288 2.868586838 1.520340192 0.288735987 1chr2_217980800_217982113 71.04668782 36.73004681 105.3633288 2.868586838 1.520340192 0.288735987 1chr6_170223889_170225075 71.04668782 36.73004681 105.3633288 2.868586838 1.520340192 0.288735987 1chr2_152949135_152953978 444.4412482 229.7763393 659.1061571 2.868468351 1.5202806 0.028454152 1chr21_37616221_37619699 444.4412482 229.7763393 659.1061571 2.868468351 1.5202806 0.028454152 1chr1_76875524_76876891 89.20780908 46.12610529 132.2895129 2.867996594 1.520043311 0.236014247 1chr9_117438872_117440357 89.20780908 46.12610529 132.2895129 2.867996594 1.520043311 0.236014247 1chr5_168509654_168511313 125.5300516 64.91822226 186.1418809 2.867328686 1.519707292 0.166182366 1chr17_74692662_74694821 180.0134154 93.10639772 266.9204331 2.866832351 1.51945754 0.107561736 1chr8_8701259_8703139 396.3490733 205.0049124 587.6932342 2.866727569 1.519404809 0.034152493 1chr13_93014641_93015699 54.48336378 28.18817546 80.77855211 2.865689276 1.518882188 0.355074339 1chr2_190964404_190966029 90.8056063 46.98029243 134.6309202 2.865689276 1.518882188 0.232425746 1chr20_24851276_24853609 163.4500913 84.56452637 242.3356563 2.865689276 1.518882188 0.121777794 1chr3_12918608_12920668 145.2889701 75.16846788 215.4094723 2.865689276 1.518882188 0.140599791 1chr6_16598391_16599636 72.64448504 37.58423394 107.7047361 2.865689276 1.518882188 0.283847531 1chr8_58985577_58987235 311.9346559 161.4413685 462.4279432 2.864370808 1.518218269 0.049565652 1chr1_75924963_75928353 275.6124134 142.6492516 408.5755752 2.864197118 1.518130784 0.059649575 1chr7_5474593_5478311 275.6124134 142.6492516 408.5755752 2.864197118 1.518130784 0.059649575 1chr2_150168919_150172209 239.2901708 123.8571346 354.7232071 2.863970721 1.518016744 0.073227659 1chr8_8236038_8242938 1178.289733 609.8896144 1746.689851 2.863944245 1.518003407 0.006758695 1chr15_79480484_79481830 92.40340353 47.83447956 136.9723275 2.863464362 1.517761648 0.228920682 1chr3_52426981_52428721 92.40340353 47.83447956 136.9723275 2.863464362 1.517761648 0.228920682 1chr7_22457462_22460231 240.8879681 124.7113217 357.0646144 2.863129101 1.517592724 0.072613832 1chr12_10234296_10235483 74.24228227 38.43842108 110.0461435 2.862920494 1.517487606 0.279087987 1chr8_86343403_86344607 74.24228227 38.43842108 110.0461435 2.862920494 1.517487606 0.279087987 1chr10_73658827_73660567 130.3234433 67.48078367 193.1661029 2.862534967 1.517293317 0.159957425 1chr2_203211620_203214417 316.7280475 164.0039299 469.4521652 2.862444609 1.517247776 0.048572349 1chr18_47176161_47179042 372.8092085 193.0462925 552.5721246 2.862381439 1.517215937 0.03783433 1chr12_28375642_28376715 56.081161 29.04236259 83.11995942 2.862024711 1.517036129 0.348306963 1chr5_24702946_24705472 168.243483 87.12708778 249.3598782 2.862024711 1.517036129 0.117861864 1chr6_38161621_38163600 112.162322 58.08472518 166.2399188 2.862024711 1.517036129 0.188564776 1chr6_40457441_40459110 112.162322 58.08472518 166.2399188 2.862024711 1.517036129 0.188564776 1chr8_102261011_102264104 262.2446838 135.8157545 388.673613 2.861771188 1.516908327 0.06436549 1chr12_59478511_59481100 150.0823618 77.73102929 222.4336942 2.861581742 1.516812819 0.135747679 1chr10_43452455_43457723 732.2506875 379.259088 1085.242287 2.861479979 1.516761513 0.013298942 1chr12_31627769_31629123 94.00120075 48.6886667 139.3137348 2.861317515 1.5166796 0.225496367 1chr7_69342368_69344023 169.8412802 87.98127491 251.7012856 2.860850628 1.516444173 0.116599178 1chr7_65702912_65704430 75.84007949 39.29260821 112.3875508 2.860272094 1.516152395 0.274452493 1chrX_75395705_75398717 171.4390775 88.83546205 254.0426929 2.859699123 1.515863365 0.115357037 1chr20_61976192_61979066 362.6370734 187.9211697 537.3529771 2.859459516 1.51574248 0.039672649 1chr2_204916046_204917728 95.59899797 49.54285383 141.6551421 2.859244697 1.515634093 0.222150227 1chr2_238988768_238990148 210.9569144 109.3359533 312.5778756 2.858875476 1.515447782 0.087833424 1chr9_85295783_85298751 210.9569144 109.3359533 312.5778756 2.858875476 1.515447782 0.087833424 1chr13_100166764_100169324 173.0368747 89.68964918 256.3841002 2.858569551 1.515293392 0.114135002 1chr19_8204133_8205248 57.67895823 29.89654973 85.46136672 2.858569551 1.515293392 0.341747066 1chr8_58842872_58844409 135.1168349 70.04334507 200.1903248 2.858092008 1.515052361 0.154080414 1chr7_105659612_105664549 657.4230534 340.8206669 974.0254399 2.85788256 1.514946632 0.015779721 1chr12_32628869_32630096 77.43787671 40.14679535 114.7289581 2.857736392 1.514872843 0.269936437 1chr1_49368626_49369969 97.19679519 50.39704097 143.9965494 2.857242144 1.514623306 0.2188798 1chrX_18981263_18982916 97.19679519 50.39704097 143.9965494 2.857242144 1.514623306 0.2188798 1chr22_24012960_24015138 214.1525089 111.0443276 317.2606902 2.857063455 1.514533079 0.086226341 1chr7_8293163_8296618 451.2595306 234.047275 668.4717863 2.85614001 1.514066703 0.028398782 1chr10_35987878_35991244 255.268143 132.3990059 378.1372802 2.856043197 1.5140178 0.067433724 1chr12_73965995_73967419 98.79459241 51.2512281 146.3379567 2.855306344 1.51364554 0.215682726 1chr2_42380475_42381582 59.27675545 30.75073686 87.80277403 2.855306344 1.51364554 0.335385133 1chr3_38461411_38464366 256.8659403 133.2531931 380.4786875 2.855306344 1.51364554 0.066894108 1chr4_115520293_115522035 118.5535109 61.50147372 175.6055481 2.855306344 1.51364554 0.178653055 1chrX_153378781_153381166 138.3124294 71.75171934 204.8731394 2.855306344 1.51364554 0.15034293 1chr18_55794859_55797134 238.704819 123.8571346 353.5525034 2.854518673 1.5132475 0.074356381 1chr4_82555949_82557852 139.9102266 72.60590648 207.2145467 2.85396267 1.512966464 0.148525676 1chr14_32186439_32188675 120.1513081 62.35566086 177.9469554 2.853741792 1.512854805 0.176299118 1chr19_6410422_6412301 100.3923896 52.10541524 148.679364 2.853434012 1.5126992 0.212556744 1chr6_109745183_109746479 483.8008269 251.1310177 716.4706361 2.852975481 1.512467348 0.025620026 1chr2_174861746_174864157 141.5080238 73.46009361 209.555954 2.852650245 1.512302873 0.146741627 1chr21_32654079_32658189 385.0062345 199.8797896 570.1326794 2.852377824 1.512165093 0.03658856 1chr1_19979565_19982284 284.6138449 147.7743744 421.4533154 2.852005411 1.511976719 0.057851586 1chr3_195575107_195576893 162.8647395 84.56452637 241.1649527 2.851845366 1.511895758 0.123987561 1chr14_24875648_24878579 305.9705606 158.8788071 453.062314 2.851622077 1.511782795 0.051983257 1chr12_1061829_1063286 123.3469026 64.06403513 182.62977 2.850737853 1.511335379 0.17173073 1chr7_67147576_67148971 123.3469026 64.06403513 182.62977 2.850737853 1.511335379 0.17173073 1chr9_100657436_100659111 144.7036183 75.16846788 214.2387686 2.850114877 1.51102007 0.143269938 1chr12_100612659_100616548 249.8893996 129.8364445 369.9423546 2.849295172 1.510605085 0.070135666 1chr12_107789148_107792912 312.3617494 162.2955557 462.4279432 2.849295172 1.510605085 0.050569632 1chr13_34061516_34062651 62.47234989 32.45911113 92.48558865 2.849295172 1.510605085 0.323220165 1chr17_62102905_62103967 62.47234989 32.45911113 92.48558865 2.849295172 1.510605085 0.323220165 1chr6_106205520_106207695 124.9446998 64.91822226 184.9711773 2.849295172 1.510605085 0.169513805 1chrX_17757235_17759987 187.4170497 97.3773334 277.4567659 2.849295172 1.510605085 0.103974254 1chr1_113733325_113735258 146.3014155 76.02265502 216.5801759 2.848889925 1.51039988 0.141580756 1chr12_62930284_62932062 105.1857813 54.66797664 155.7035859 2.848168078 1.510034286 0.20358408 1chr3_65793338_65794807 105.1857813 54.66797664 155.7035859 2.848168078 1.510034286 0.20358408 1chr1_235551710_235554479 190.6126441 99.08570767 282.1395806 2.847429636 1.509660192 0.101904611 1chr13_107253877_107256314 190.6126441 99.08570767 282.1395806 2.847429636 1.509660192 0.101904611 1chr3_72004079_72006593 192.2104413 99.9398948 284.4809879 2.846520786 1.509199634 0.100893409 1chr5_35002981_35004001 64.07014711 33.31329827 94.82699595 2.846520786 1.509199634 0.317401001 1chr7_78873166_78874612 106.7835785 55.52216378 158.0449933 2.846520786 1.509199634 0.200721619 1chr7_111912647_111913920 106.7835785 55.52216378 158.0449933 2.846520786 1.509199634 0.200721619 1chr1_47075560_47082231 1312.346441 682.4955209 1942.197362 2.845729096 1.508798328 0.006187748 1chr6_106557418_106558956 129.7380914 67.48078367 191.9953992 2.84518627 1.508523107 0.163118141 1chr11_66734699_66736170 108.3813757 56.37635091 160.3864006 2.844923411 1.508389814 0.197920233 1chr7_27913316_27916475 325.1441272 169.1290527 481.1592017 2.844923411 1.508389814 0.04792145 1chr8_145626799_145628006 262.6717773 136.6699416 388.673613 2.843885118 1.507863187 0.06575488 1chr17_80391911_80395905 638.5183221 332.2787955 944.7578486 2.843268548 1.507550369 0.017013086 1chr2_28865726_28868033 177.2449145 92.25221059 262.2376184 2.842616093 1.50721927 0.11252114 1chr7_69360733_69364304 265.8673718 138.3783159 393.3564277 2.842616093 1.50721927 0.064726762 1chr10_7392146_7394779 200.1994274 104.2108305 296.1880244 2.842200019 1.507008088 0.096061286 1chr12_52334189_52335864 111.5769702 58.08472518 165.0692152 2.841869608 1.506840362 0.192493656 1chr2_233140356_233142315 201.7972247 105.0650176 298.5294317 2.84137802 1.506590782 0.095137606 1chr3_183217393_183219327 134.5314831 70.04334507 199.0196211 2.84137802 1.506590782 0.157082511 1chr2_237117605_237119675 136.1292803 70.89753221 201.3610284 2.84016978 1.505977174 0.15514594 1chr8_10535724_10539867 1521.974394 792.6856613 2251.263126 2.840045223 1.505913903 0.005271488 0.9889824chr1_68623924_68626518 183.6361034 95.66895913 271.6032477 2.838990307 1.505377924 0.107938268 1chr2_123249389_123250627 68.86353877 35.87585967 101.8512179 2.838990307 1.505377924 0.300910063 1chr7_107439684_107440475 45.90902585 23.91723978 67.90081192 2.838990307 1.505377924 0.403158921 1chr10_11836532_11837946 93.41584892 48.6886667 138.1430311 2.83727283 1.504504886 0.230480903 1chr4_186729958_186731747 93.41584892 48.6886667 138.1430311 2.83727283 1.504504886 0.230480903 1chr5_153862242_153864093 93.41584892 48.6886667 138.1430311 2.83727283 1.504504886 0.230480903 1chr8_101870400_101874663 608.0019166 316.9034271 899.1004061 2.837143209 1.504438975 0.018564395 1chr1_163986078_163987463 117.9681591 61.50147372 174.4348444 2.836270968 1.503995369 0.182299674 1chr22_45057624_45061002 353.9044772 184.5044212 523.3045332 2.836270968 1.503995369 0.042722404 1chr5_230281_232217 117.9681591 61.50147372 174.4348444 2.836270968 1.503995369 0.182299674 1chr3_49414711_49416497 95.01364614 49.54285383 140.4844385 2.835614576 1.503661451 0.22703195 1chr6_126588908_126589886 47.50682307 24.77142692 70.24221923 2.835614576 1.503661451 0.394860042 1chr2_220492470_220494708 167.0727794 87.12708778 247.0184709 2.83515124 1.503425697 0.122122606 1chr1_249237636_249239197 144.1182664 75.16846788 213.068065 2.834540479 1.503114872 0.145986791 1chr18_60245692_60247052 72.05913322 37.58423394 106.5340325 2.834540479 1.503114872 0.290657504 1chrX_149241330_149242744 72.05913322 37.58423394 106.5340325 2.834540479 1.503114872 0.290657504 1chr8_121277994_121279389 96.61144336 50.39704097 142.8258458 2.834012534 1.502846139 0.223661758 1chr6_108497871_108499742 121.1637535 63.20984799 179.117659 2.83369862 1.502686328 0.177508313 1chr1_54836209_54839185 679.475698 354.487661 1004.463735 2.833564734 1.502618162 0.015812643 1chrX_68508270_68510944 194.8206839 101.6482691 287.9930988 2.833231706 1.502448592 0.100578083 1chr12_66801246_66802552 98.20924058 51.2512281 145.1672531 2.832463893 1.502057565 0.220367842 1chr14_40804111_40806806 245.5231015 128.1280703 362.9181327 2.832463893 1.502057565 0.073479627 1chr3_155419874_155422683 173.4639682 90.54383632 256.3841002 2.831601914 1.501618455 0.116950282 1chr16_46764807_46767274 248.7186959 129.8364445 367.6009473 2.831261659 1.501445086 0.072263514 1chr2_205153257_205154718 75.25472766 39.29260821 111.2168471 2.830477593 1.501045503 0.28094481 1chr20_22714976_22716548 150.5094553 78.58521643 222.4336942 2.830477593 1.501045503 0.139238537 1chr14_23605867_23608752 1038.60066 542.4088308 1534.79249 2.829586104 1.500591039 0.008717017 1chr12_39771862_39773378 101.404835 52.95960237 149.8500677 2.82951648 1.50055554 0.213999429 1chr7_67635459_67636984 101.404835 52.95960237 149.8500677 2.82951648 1.50055554 0.213999429 1chr5_60017601_60018937 76.85252488 40.14679535 113.5582544 2.828575816 1.500075841 0.276278167 1chr6_84127754_84128911 76.85252488 40.14679535 113.5582544 2.828575816 1.500075841 0.276278167 1chr7_50609892_50611027 256.707682 134.1073802 379.3079838 2.82839008 1.499981104 0.069357399 1chr12_329913_331579 129.1527396 67.48078367 190.8246956 2.827837574 1.499699257 0.166335941 1chr7_28763562_28766934 387.4582188 202.442351 572.4740867 2.827837574 1.499699257 0.037733888 1chr8_63849332_63852349 310.605694 162.2955557 458.9158323 2.827654956 1.499606086 0.052643639 1chr3_194553692_194555767 207.6030617 108.4817662 306.7243573 2.827427762 1.499490165 0.093071105 1chr1_88056874_88058168 78.4503221 41.00098248 115.8996617 2.82675328 1.49914597 0.271731729 1chr2_135124987_135127528 209.2008589 109.3359533 309.0657646 2.82675328 1.49914597 0.092193701 1chr3_23292028_23292977 52.30021473 27.33398832 77.26644115 2.82675328 1.49914597 0.371599669 1chr3_53039509_53041010 130.7505368 68.3349708 193.1661029 2.82675328 1.49914597 0.164230078 1chr4_4664543_4666100 78.4503221 41.00098248 115.8996617 2.82675328 1.49914597 0.271731729 1chr4_15615908_15616939 52.30021473 27.33398832 77.26644115 2.82675328 1.49914597 0.371599669 1chr9_80440784_80442026 78.4503221 41.00098248 115.8996617 2.82675328 1.49914597 0.271731729 1chr8_10632293_10636435 555.5434436 290.4236259 820.6632613 2.825745525 1.498631548 0.021800223 1chr22_43558305_43560628 212.3964534 111.0443276 313.7485792 2.825435446 1.498473228 0.090476721 1chr5_80782158_80784027 106.1982267 55.52216378 156.8742896 2.825435446 1.498473228 0.204962528 1chr12_65550026_65551559 80.04811932 41.85516962 118.241069 2.825005133 1.498253489 0.267301097 1chr6_167773756_167775085 80.04811932 41.85516962 118.241069 2.825005133 1.498253489 0.267301097 1chr7_2543876_2545478 401.8383938 210.1300352 593.5467525 2.824664032 1.498079282 0.035883632 1chr1_59470394_59471840 107.7960239 56.37635091 159.2156969 2.824157547 1.497820572 0.202079605 1chr4_144076570_144077557 53.89801196 28.18817546 79.60784846 2.824157547 1.497820572 0.364345845 1chr10_114220363_114221677 81.64591654 42.70935675 120.5824763 2.823326913 1.497396188 0.262982092 1chr10_118927155_118928278 81.64591654 42.70935675 120.5824763 2.823326913 1.497396188 0.262982092 1chr14_34534218_34535001 137.1417257 71.75171934 202.5317321 2.822674271 1.497062655 0.156199382 1chr1_75409100_75411406 164.8896303 86.27290064 243.50636 2.822512726 1.496980086 0.125686956 1chr12_48537932_48539285 83.24371377 43.56354389 122.9238836 2.821714504 1.496572026 0.258770742 1chr13_100280350_100281709 83.24371377 43.56354389 122.9238836 2.821714504 1.496572026 0.258770742 1chr7_151660635_151661728 55.49580918 29.04236259 81.94925576 2.821714504 1.496572026 0.357321439 1chr8_59194243_59199141 417.8163661 218.6719066 616.9608255 2.821399581 1.496411002 0.033998917 1chr1_105614436_105616208 168.0852248 87.98127491 248.1891746 2.820931782 1.496171778 0.122948673 1chr6_42315761_42318746 308.4225449 161.4413685 455.4037213 2.820861378 1.496135771 0.053727558 1chr7_21082585_21085195 197.4309266 103.3566433 291.5052098 2.820381935 1.495890545 0.100258998 1chr21_47048444_47049715 84.84151099 44.41773102 125.265291 2.820164112 1.495779119 0.254663266 1chr4_1866304_1868977 256.1223302 134.1073802 378.1372802 2.819660481 1.495521456 0.070384469 1chr1_86428854_86429895 57.0936064 29.89654973 84.29066307 2.819411064 1.495393835 0.350515508 1chr10_30707153_30708261 57.0936064 29.89654973 84.29066307 2.819411064 1.495393835 0.350515508 1chr17_40631354_40632462 57.0936064 29.89654973 84.29066307 2.819411064 1.495393835 0.350515508 1chr18_56691677_56695520 342.5616384 179.3792984 505.7439784 2.819411064 1.495393835 0.046072551 1chr4_147319719_147320835 57.0936064 29.89654973 84.29066307 2.819411064 1.495393835 0.350515508 1chr1_57332158_57334565 174.4764136 91.39802345 257.5548038 2.817947195 1.494644578 0.117738124 1chr10_43490671_43493346 174.4764136 91.39802345 257.5548038 2.817947195 1.494644578 0.117738124 1chr11_118584737_118587290 233.1678173 122.1487603 344.1868742 2.81776805 1.494552859 0.080430092 1chr20_20642921_20647652 498.8769308 261.3812633 736.3725982 2.817235592 1.494280215 0.02612069 1chr5_138534040_138538517 410.8398253 215.255158 606.4244926 2.817235592 1.494280215 0.035133715 1chr3_130490818_130492656 118.9806045 62.35566086 175.6055481 2.816192558 1.493745982 0.183526319 1chr1_1387297_1388931 89.63490265 46.98029243 132.2895129 2.815851201 1.493571099 0.24292893 1chr6_41511297_41513525 687.7335194 360.466971 1015.000068 2.815792152 1.493540845 0.016139571 1chr6_129002798_129004454 91.23269987 47.83447956 134.6309202 2.814516253 1.492886979 0.239202599 1chr1_16324062_16325108 61.88699806 32.45911113 91.31488499 2.813228145 1.492226556 0.33130994 1chr3_53888496_53890117 92.83049709 48.6886667 136.9723275 2.813228145 1.492226556 0.235563736 1chr7_69465821_69468004 123.7739961 64.91822226 182.62977 2.813228145 1.492226556 0.176360291 1chr5_67943627_67950182 532.4306724 279.3191932 785.5421517 2.812345771 1.491773981 0.023877294 1chr1_14653019_14654250 63.48479528 33.31329827 93.6562923 2.811378554 1.491277726 0.325282449 1chr12_96551000_96551924 63.48479528 33.31329827 93.6562923 2.811378554 1.491277726 0.325282449 1chr5_150628081_150629256 63.48479528 33.31329827 93.6562923 2.811378554 1.491277726 0.325282449 1chr2_232182643_232184423 159.5108868 83.71033924 235.3114344 2.811019959 1.491093698 0.132331058 1chr12_105085128_105086597 96.02609154 50.39704097 141.6551421 2.810782923 1.490972038 0.228537138 1chr8_101528847_101530639 161.108684 84.56452637 237.6528417 2.810313638 1.490731148 0.130842394 1chr3_62110620_62112211 97.62388876 51.2512281 143.9965494 2.809621442 1.49037576 0.225144068 1chr8_74378307_74379616 97.62388876 51.2512281 143.9965494 2.809621442 1.49037576 0.225144068 1chr1_68148010_68150714 196.8455747 103.3566433 290.3345061 2.8090551 1.490084924 0.101927411 1chr5_17105187_17106926 164.3042785 86.27290064 242.3356563 2.808942953 1.490027325 0.127940567 1chr7_18191988_18197711 624.6758177 328.0078599 921.3437755 2.808907615 1.490009175 0.018895491 1chr10_73494992_73498271 398.4845411 209.2758481 587.6932342 2.808222925 1.489657465 0.037367194 1chr7_38375076_38376957 232.5824654 122.1487603 343.0161705 2.808183805 1.489637368 0.081673039 1chr12_14341320_14342409 66.68038973 35.02167254 98.33910692 2.807950043 1.489517269 0.313741289 1chr13_57258306_57259604 66.68038973 35.02167254 98.33910692 2.807950043 1.489517269 0.313741289 1chr18_44060555_44063553 234.1802627 123.0029474 345.3575779 2.807717905 1.489397993 0.080957856 1chr3_16591441_16593954 235.7780599 123.8571346 347.6989852 2.80725843 1.489161881 0.080252351 1chr15_77873241_77877204 473.153917 248.5684563 697.7393776 2.807031061 1.489045028 0.028858101 1chr1_168360349_168363179 375.5300282 197.3172282 553.7428282 2.806358235 1.488699182 0.040987509 1chr12_104794216_104796170 170.6954674 89.68964918 251.7012856 2.806358235 1.488699182 0.122423471 1chr3_10046004_10049692 409.6691217 215.255158 604.0830853 2.806358235 1.488699182 0.035949787 1chr10_120741683_120744007 172.2932646 90.54383632 254.0426929 2.805742535 1.488382628 0.121100856 1chr15_82459799_82461880 172.2932646 90.54383632 254.0426929 2.805742535 1.488382628 0.121100856 1chr13_101297083_101298865 138.1541711 72.60590648 203.7024358 2.805590421 1.48830441 0.157240944 1chr12_66040970_66042797 104.0150776 54.66797664 153.3621786 2.805338483 1.488174852 0.212316199 1chr1_60157162_60158331 69.87598417 36.73004681 103.0219215 2.804840464 1.487918714 0.302838439 1chr1_80545452_80547643 139.7519683 73.46009361 206.0438431 2.804840464 1.487918714 0.155313015 1chr8_111472076_111473179 69.87598417 36.73004681 103.0219215 2.804840464 1.487918714 0.302838439 1chrX_109472615_109473771 71.47378139 37.58423394 105.3633288 2.803391683 1.487173328 0.297610345 1chr7_69147170_69151480 610.7227363 321.1743628 900.2711097 2.803060313 1.487002787 0.019782866 1chr13_52269233_52270510 73.07157861 38.43842108 107.7047361 2.802007292 1.48646071 0.292523304 1chr1_93226430_93228341 110.4062665 58.08472518 162.7278079 2.801559401 1.486230082 0.200574589 1chr10_11895244_11897175 147.7409544 77.73102929 217.7508796 2.801337916 1.486116021 0.146191815 1chr12_133395913_133398275 222.4103303 117.0236375 327.7970231 2.801118048 1.486002784 0.087534611 1chr1_47151667_47157430 743.4981639 391.2177079 1095.77862 2.800943306 1.485912782 0.014880882 1chr11_16517693_16519010 74.66937583 39.29260821 110.0461435 2.800683092 1.485778746 0.287571845 1chr2_122790812_122792148 74.66937583 39.29260821 110.0461435 2.800683092 1.485778746 0.287571845 1chr5_34057406_34058976 112.0040637 58.93891232 165.0692152 2.800683092 1.485778746 0.197794125 1chr15_91251449_91253583 150.9365489 79.43940356 222.4336942 2.80004235 1.485448648 0.142769924 1chr18_2460175_2461960 113.601861 59.79309945 167.4106225 2.79983182 1.48534017 0.195072069 1chr2_212223124_212226061 189.869034 99.9398948 279.7981732 2.799664476 1.485253939 0.107885668 1chr21_18947793_18953202 532.2724142 280.1733803 784.371448 2.799593049 1.485217131 0.024488383 1chr13_78049595_78050983 76.26717305 40.14679535 112.3875508 2.799415241 1.485125499 0.282750789 1chr11_76001814_76004466 420.2683504 221.234468 619.3022328 2.799302652 1.485067475 0.035013089 1chr10_33498077_33499509 115.1996582 60.64728659 169.7520298 2.799004528 1.484913821 0.192406756 1chr4_157441775_157443482 115.1996582 60.64728659 169.7520298 2.799004528 1.484913821 0.192406756 1chr5_169759893_169761530 115.1996582 60.64728659 169.7520298 2.799004528 1.484913821 0.192406756 1chr1_22309651_22311568 154.1321433 81.14777783 227.1165088 2.798801334 1.484809084 0.139468387 1chr22_37043037_37045130 193.0646285 101.6482691 284.4809879 2.7986801 1.484746591 0.105720827 1chr2_28842616_28845210 274.1251932 144.3576258 403.8927605 2.797862311 1.484324966 0.065827488 1chr10_44776482_44779019 236.7905053 124.7113217 348.8696888 2.797417941 1.484095812 0.08077302 1chr1_89046055_89049985 445.8331059 234.9014621 656.7647498 2.795915972 1.483321003 0.032221126 1chr1_59268196_59269274 81.06056472 42.70935675 119.4117727 2.795915972 1.483321003 0.269022234 1chr1_120193257_120195243 163.7189267 86.27290064 241.1649527 2.795373181 1.483040895 0.130231634 1chr12_64829533_64832022 163.7189267 86.27290064 241.1649527 2.795373181 1.483040895 0.130231634 1chr10_46507930_46509182 82.65836194 43.56354389 121.75318 2.794841033 1.482766227 0.264676188 1chr15_79470790_79472737 165.3167239 87.12708778 243.50636 2.794841033 1.482766227 0.128783008 1chr4_187740717_187742252 82.65836194 43.56354389 121.75318 2.794841033 1.482766227 0.264676188 1chr9_33354065_33355437 82.65836194 43.56354389 121.75318 2.794841033 1.482766227 0.264676188 1chr22_27570874_27573106 124.7864415 65.7724094 183.8004736 2.794492027 1.482586059 0.177520635 1chr12_107778097_107781177 291.7009626 153.7536843 429.6482409 2.794393142 1.482535007 0.060533987 1chr2_183532605_183534455 127.982036 67.48078367 188.4832883 2.79314018 1.48188798 0.172946255 1chr19_729507_730816 85.85395638 45.27191816 126.4359946 2.792812846 1.481718898 0.256305046 1chr14_27066344_27068429 129.5798332 68.3349708 190.8246956 2.792489604 1.48155191 0.170725998 1chr22_43831357_43833373 217.0315868 114.4610761 319.6020975 2.792233905 1.481419801 0.091414552 1chr11_65994944_65997221 260.7574636 137.5241287 383.9907984 2.792170377 1.481386977 0.07117521 1chr7_1693138_1695237 220.2271812 116.1694504 324.2849121 2.791481849 1.481031175 0.089734228 1chr7_27293353_27295383 221.8249785 117.0236375 326.6263194 2.791114055 1.480841079 0.088911882 1chr14_24994924_24996183 89.04955082 46.98029243 131.1188092 2.790932164 1.480747059 0.24833743 1chr3_75502067_75503449 89.04955082 46.98029243 131.1188092 2.790932164 1.480747059 0.24833743 1chr5_18169450_18171160 178.0991016 93.96058486 262.2376184 2.790932164 1.480747059 0.11802875 1chr5_145870247_145871442 89.04955082 46.98029243 131.1188092 2.790932164 1.480747059 0.24833743 1chr8_143263047_143266885 859.5520092 453.5733687 1265.53065 2.79013438 1.480334607 0.012423942 1chr18_2556604_2558025 90.64734805 47.83447956 133.4602165 2.790042199 1.480286943 0.244496426 1chr3_62859678_62860516 45.32367402 23.91723978 66.73010826 2.790042199 1.480286943 0.414157768 1chr8_6291217_6292504 90.64734805 47.83447956 133.4602165 2.790042199 1.480286943 0.244496426 1chrX_95792120_95794679 137.5688193 72.60590648 202.5317321 2.789466338 1.479989142 0.160248787 1chr2_205622345_205623643 92.24514527 48.6886667 135.8016238 2.78918346 1.479842831 0.240746393 1chr11_60770988_60773260 323.656907 170.837427 476.4763871 2.789063236 1.479780645 0.052489594 1chr12_62685135_62687040 139.1666165 73.46009361 204.8731394 2.78890387 1.479698208 0.158270823 1chr3_58448248_58450200 142.362211 75.16846788 209.555954 2.787817285 1.479136009 0.154424596 1chr3_62117972_62121228 237.8029507 125.5655089 350.0403925 2.787711336 1.47908118 0.081291722 1chr6_51608317_51609614 95.44073971 50.39704097 140.4844385 2.787553312 1.478999397 0.233507388 1chr7_99855233_99856784 95.44073971 50.39704097 140.4844385 2.787553312 1.478999397 0.233507388 1chr9_71444407_71449866 483.5948874 255.4019534 711.7878215 2.786931784 1.47867769 0.028981947 1chr22_37019554_37020846 97.03853693 51.2512281 142.8258458 2.786778991 1.478598592 0.230012813 1chr7_7746274_7748367 147.1556026 77.73102929 216.5801759 2.786276959 1.478338671 0.148916171 1chr1_84568409_84569787 98.63633415 52.10541524 145.1672531 2.786030059 1.478210823 0.226597989 1chr22_31854993_31856825 98.63633415 52.10541524 145.1672531 2.786030059 1.478210823 0.226597989 1chr14_40991880_40992870 50.11706569 26.47980119 73.75433019 2.785305285 1.477835464 0.38913832 1chr7_19455025_19457532 151.9489943 80.2935907 223.6043979 2.784834953 1.477591827 0.143700983 1chr12_66459566_66461092 101.8319286 53.81378951 149.8500677 2.78460352 1.477471927 0.219997656 1chr6_57076503_57078060 101.8319286 53.81378951 149.8500677 2.78460352 1.477471927 0.219997656 1chr14_22873001_22875843 357.2106487 188.7753568 525.6459405 2.784505082 1.477420926 0.04574297 1chr7_134643656_134646820 256.9765173 135.8157545 378.1372802 2.784193054 1.47725925 0.073410871 1chr10_44761317_44762309 51.71486291 27.33398832 76.09573749 2.783923685 1.477119663 0.381354123 1chr2_223899441_223902581 208.4572489 110.1901404 306.7243573 2.783591673 1.476947597 0.097381692 1chr12_49973767_49975231 105.027523 55.52216378 154.5328823 2.783264768 1.476778157 0.213687535 1chr3_71476955_71478902 158.3401832 83.71033924 232.9700271 2.783049612 1.476666627 0.137168354 1chr15_90035964_90040298 476.6183467 251.9852048 701.2514886 2.78290739 1.476592899 0.02984287 1chr1_24501553_24502679 53.31266013 28.18817546 78.4371448 2.782625818 1.476446922 0.373824086 1chr5_18113291_18115241 106.6253203 56.37635091 156.8742896 2.782625818 1.476446922 0.210635828 1chr8_102294067_102296263 159.9379804 84.56452637 235.3114344 2.782625818 1.476446922 0.135605747 1chr1_64430589_64434085 321.473758 169.9832399 472.9642761 2.782417116 1.476338713 0.053543693 1chr1_72132415_72133442 54.91045735 29.04236259 80.77855211 2.781404297 1.475813466 0.36653559 1chr8_36956975_36959475 331.0605413 175.1083627 487.01272 2.781207662 1.475711469 0.05139932 1chr6_90397933_90400949 221.2396266 117.0236375 325.4556157 2.781110062 1.47566084 0.090309598 1chr7_56285723_56287528 166.3291693 87.98127491 244.6770636 2.781012936 1.475610455 0.129618517 1chr1_219110171_219111283 56.50825457 29.89654973 83.11995942 2.780252577 1.475215953 0.359476918 1chr7_67641919_67642950 56.50825457 29.89654973 83.11995942 2.780252577 1.475215953 0.359476918 1chr7_94181685_94182730 56.50825457 29.89654973 83.11995942 2.780252577 1.475215953 0.359476918 1chr3_53116622_53119984 340.6473247 180.2334855 501.0611638 2.780066992 1.475119648 0.049389665 1chr8_9497700_9500536 171.1225609 90.54383632 251.7012856 2.779883157 1.475024245 0.125388201 1chr5_149508680_149510152 114.6143064 60.64728659 168.5813261 2.779701049 1.474929732 0.196331608 1chr7_44440333_44442731 229.2286127 121.2945732 337.1626523 2.779701049 1.474929732 0.086283798 1chr1_15220352_15222630 232.4242072 123.0029474 341.8454669 2.779164841 1.474651408 0.084750741 1chr1_88252210_88253254 58.10605179 30.75073686 85.46136672 2.779164841 1.474651408 0.352637167 1chr1_236187029_236188152 58.10605179 30.75073686 85.46136672 2.779164841 1.474651408 0.352637167 1chr17_74401390_74403340 174.3181554 92.25221059 256.3841002 2.779164841 1.474651408 0.122683749 1chr6_91212213_91213316 58.10605179 30.75073686 85.46136672 2.779164841 1.474651408 0.352637167 1chr8_68243090_68246118 234.0220044 123.8571346 344.1868742 2.778902284 1.474515105 0.083999972 1chr10_80288166_80289911 117.8099008 62.35566086 173.2641408 2.778643324 1.474380657 0.191021754 1chr11_125761408_125763635 179.111547 94.81477199 263.4083221 2.778135902 1.474117175 0.118790784 1chr12_66543623_66544782 59.70384901 31.604924 87.80277403 2.778135902 1.474117175 0.346006173 1chr7_22351341_22353376 179.111547 94.81477199 263.4083221 2.778135902 1.474117175 0.118790784 1chr9_128964892_128965958 59.70384901 31.604924 87.80277403 2.778135902 1.474117175 0.346006173 1chr2_240230031_240236120 601.8318818 318.6118014 885.0519622 2.777837978 1.473962454 0.021299568 1chr14_102353332_102355566 180.7093443 95.66895913 265.7497294 2.777805171 1.473945416 0.117534921 1chr22_40099642_40101224 121.0054952 64.06403513 177.9469554 2.777642011 1.473860673 0.18592766 1chr1_68824109_68825366 61.30164624 32.45911113 90.14418134 2.777161117 1.473610878 0.339574447 1chr10_21514321_21517541 306.5082312 162.2955557 450.7209067 2.777161117 1.473610878 0.05780608 1chr11_61975153_61977250 245.2065849 129.8364445 360.5767254 2.777161117 1.473610878 0.079021707 1chr12_76276609_76277755 61.30164624 32.45911113 90.14418134 2.777161117 1.473610878 0.339574447 1chr13_23895904_23896973 61.30164624 32.45911113 90.14418134 2.777161117 1.473610878 0.339574447 1chr2_161070695_161071626 61.30164624 32.45911113 90.14418134 2.777161117 1.473610878 0.339574447 1chr4_55374922_55376121 61.30164624 32.45911113 90.14418134 2.777161117 1.473610878 0.339574447 1chr8_27668199_27669374 61.30164624 32.45911113 90.14418134 2.777161117 1.473610878 0.339574447 1chr21_33571947_33572964 62.89944346 33.31329827 92.48558865 2.776236322 1.47313038 0.333333112 1chr7_28487858_28489036 62.89944346 33.31329827 92.48558865 2.776236322 1.47313038 0.333333112 1chrX_153538449_153539643 62.89944346 33.31329827 92.48558865 2.776236322 1.47313038 0.333333112 1chr1_235275190_235278416 316.0950145 167.4206785 464.7693505 2.776057025 1.473037203 0.055392784 1chr8_131168582_131170740 254.7933683 134.9615673 374.6251692 2.775791483 1.472899197 0.075110123 1chr2_204677211_204680398 196.6873165 104.2108305 289.1638025 2.774796066 1.472381744 0.106018061 1chr12_110182713_110183952 67.69283512 35.87585967 99.50981057 2.773726162 1.471825364 0.315670794 1chr4_56860746_56862072 67.69283512 35.87585967 99.50981057 2.773726162 1.471825364 0.315670794 1chr6_48047040_48049199 135.3856702 71.75171934 199.0196211 2.773726162 1.471825364 0.165377389 1chr7_17861832_17863428 135.3856702 71.75171934 199.0196211 2.773726162 1.471825364 0.165377389 1chr12_6250612_6251795 69.29063234 36.73004681 101.8512179 2.772967277 1.471430592 0.310112769 1chr18_47570019_47571314 69.29063234 36.73004681 101.8512179 2.772967277 1.471430592 0.310112769 1chr2_148406218_148407420 69.29063234 36.73004681 101.8512179 2.772967277 1.471430592 0.310112769 1chr6_138316222_138317403 69.29063234 36.73004681 101.8512179 2.772967277 1.471430592 0.310112769 1chrX_129727936_129729310 69.29063234 36.73004681 101.8512179 2.772967277 1.471430592 0.310112769 1chr1_85694085_85696902 280.3581238 148.6285615 412.0876861 2.772600918 1.471239973 0.066040358 1chr2_208170749_208171987 70.88842956 37.58423394 104.1926252 2.772242886 1.471053663 0.304708276 1chr1_54618244_54620836 285.1515155 151.1911229 419.111908 2.772066904 1.470962078 0.064531501 1chr14_19108915_19110350 72.48622678 38.43842108 106.5340325 2.771550691 1.470693394 0.299451182 1chr17_77851608_77852841 74.08402401 39.29260821 108.8754398 2.770888591 1.470348706 0.294335695 1chr7_65447113_65450574 370.42012 196.4630411 544.377199 2.770888591 1.470348706 0.044327648 1chr8_70374157_70375305 74.08402401 39.29260821 108.8754398 2.770888591 1.470348706 0.294335695 1chr14_61030538_61032678 151.3636425 80.2935907 222.4336942 2.770254665 1.470018607 0.146341504 1chr8_125237902_125239602 151.3636425 80.2935907 222.4336942 2.770254665 1.470018607 0.146341504 1chr1_48508087_48510342 228.6432609 121.2945732 335.9919486 2.770049309 1.469911658 0.087617106 1chr8_36812453_36813789 77.27961845 41.00098248 113.5582544 2.769647153 1.469702192 0.284507924 1chr10_121608209_121610799 156.1570341 82.8561521 229.4579161 2.769352792 1.469548852 0.141276967 1chr1_232902799_232904271 78.87741567 41.85516962 115.8996617 2.769064438 1.469398626 0.279785543 1chr12_109816013_109817243 78.87741567 41.85516962 115.8996617 2.769064438 1.469398626 0.279785543 1chr3_32235431_32236951 159.3526286 84.56452637 234.1407308 2.768781909 1.46925142 0.138047149 1chr4_105309401_105310918 80.47521289 42.70935675 118.241069 2.768505031 1.469107143 0.275184535 1chr12_13127930_13129288 82.07301011 43.56354389 120.5824763 2.767967561 1.468827036 0.270700477 1chr3_41465078_41466627 83.67080733 44.41773102 122.9238836 2.767450764 1.46855765 0.266329166 1chr4_91069337_91070740 85.26860455 45.27191816 125.265291 2.766953468 1.468298382 0.262066605 1chr10_54433125_54435659 257.4036109 136.6699416 378.1372802 2.766791847 1.46821411 0.074952507 1chr7_65791266_65794041 172.1350063 91.39802345 252.8719892 2.766711792 1.468172366 0.126188723 1chr13_99578014_99579458 86.86640178 46.12610529 127.6066983 2.766474591 1.468048673 0.257908989 1chr18_43929456_43931589 173.7328036 92.25221059 255.2133965 2.766474591 1.468048673 0.124816188 1chr10_46974113_46977458 961.9216084 510.8039068 1413.03931 2.766304821 1.467960137 0.0113018 1chr1_78339926_78341264 88.464199 46.98029243 129.9481056 2.766013127 1.467808003 0.253852695 1chr5_21203434_21205373 178.5261952 94.81477199 262.2376184 2.765788631 1.467690906 0.120832335 1chr11_118732900_118734437 90.06199622 47.83447956 132.2895129 2.765568144 1.467575891 0.249894267 1chr7_18051766_18053265 90.06199622 47.83447956 132.2895129 2.765568144 1.467575891 0.249894267 1chr1_49605519_49606893 91.65979344 48.6886667 134.6309202 2.765138775 1.467351887 0.246030411 1chr7_33934395_33935781 91.65979344 48.6886667 134.6309202 2.765138775 1.467351887 0.246030411 1chr9_33877570_33879654 184.9173841 98.23152053 271.6032477 2.764929691 1.467242795 0.11581511 1chr1_89330327_89331780 93.25759066 49.54285383 136.9723275 2.764724211 1.467135575 0.242257978 1chr12_110030740_110034644 466.2879533 247.7142692 684.8616374 2.764724211 1.467135575 0.03188567 1chr21_33799782_33801320 93.25759066 49.54285383 136.9723275 2.764724211 1.467135575 0.242257978 1chr1_84415727_84416974 94.85538788 50.39704097 139.3137348 2.764323701 1.466926564 0.238573965 1chr2_206492920_206495287 189.7107758 100.7940819 278.6274696 2.764323701 1.466926564 0.112257881 1chr3_123943014_123944830 96.4531851 51.2512281 141.6551421 2.763936541 1.466724492 0.234975496 1chr3_66118555_66120280 101.2465768 53.81378951 148.679364 2.762848805 1.466156614 0.224666457 1chr6_51299087_51300564 101.2465768 53.81378951 148.679364 2.762848805 1.466156614 0.224666457 1chr1_12237010_12238504 102.844374 54.66797664 151.0207713 2.762508887 1.465979106 0.221383829 1chr8_9004519_9006974 523.8086533 278.465006 769.1523005 2.76211475 1.465773257 0.026978218 1chr13_52599074_52600709 106.0399684 56.37635091 155.7035859 2.761859954 1.465640167 0.215035101 1chr1_120159983_120162089 213.6777341 113.606889 313.7485792 2.76170382 1.465558605 0.096749462 1chr3_65949304_65951016 107.6377657 57.23053805 158.0449933 2.761550016 1.465478257 0.21196465 1chr18_43343427_43344922 109.2355629 58.08472518 160.3864006 2.761249193 1.465321092 0.208960718 1chr3_9810733_9812751 226.4601119 120.440386 332.4798377 2.760534473 1.464947618 0.089785732 1chr3_14342142_14346989 460.9092098 245.1517078 676.6667119 2.760195791 1.464770606 0.032708243 1chr2_120890604_120892450 115.6267518 61.50147372 169.7520298 2.760129465 1.464735939 0.197571966 1chr1_168253426_168258724 594.111731 316.04924 872.174222 2.759614996 1.464467005 0.022536971 1chr18_55128492_55129953 118.8223462 63.20984799 174.4348444 2.759614996 1.464467005 0.19222903 1chr5_171781516_171783252 358.0648358 190.4837311 525.6459405 2.759531942 1.464423585 0.047444604 1chr15_101686052_101687936 125.2135351 66.62659653 183.8004736 2.758665206 1.46397038 0.182182298 1chr4_58056336_58058262 125.2135351 66.62659653 183.8004736 2.758665206 1.46397038 0.182182298 1chr11_68007090_68008720 128.4091295 68.3349708 188.4832883 2.758225928 1.463740634 0.17745569 1chr12_83198304_83200128 128.4091295 68.3349708 188.4832883 2.758225928 1.463740634 0.17745569 1chr20_18612916_18617909 391.6185775 208.421661 574.815494 2.757945078 1.463593727 0.041740011 1chr2_6099054_6101035 131.604724 70.04334507 193.1661029 2.757808078 1.46352206 0.172913021 1chr13_60252897_60256965 271.1984341 144.3576258 398.0392423 2.757313581 1.46326335 0.070649478 1chr8_36960211_36966502 975.5581733 519.3457781 1431.770569 2.756873414 1.463033026 0.011356966 1chr8_17599327_17601651 141.1915073 75.16846788 207.2145467 2.756668488 1.462925782 0.160296302 1chr5_134649783_134651766 147.5826962 78.58521643 216.5801759 2.75599134 1.462571355 0.152645743 1chr12_82259118_82260705 150.7782906 80.2935907 221.2629906 2.755674378 1.462405423 0.14902667 1chr1_213832823_213835212 152.3760878 81.14777783 223.6043979 2.755520901 1.46232507 0.147265801 1chr22_42752818_42754747 177.9408434 94.81477199 261.0669148 2.753441361 1.461235884 0.122906514 1chr7_50544914_50549304 549.8005024 292.9861873 806.6148174 2.753081382 1.461047257 0.025575591 1chr8_144158817_144164009 604.1256079 322.0285499 886.2226659 2.7520003 1.460480627 0.022344512 1chr10_115938139_115940966 201.9078017 107.627579 296.1880244 2.751971447 1.460465502 0.105233347 1chr10_78933472_78936617 206.7011934 110.1901404 303.2122463 2.751718486 1.460332883 0.102166735 1chr10_54829578_54832466 208.2989906 111.0443276 305.5536536 2.751636759 1.460290034 0.101174915 1chr1_68026343_68028662 214.6901795 114.4610761 314.9192829 2.751322053 1.460125023 0.09735178 1chrX_106725009_106730002 448.5539256 239.1723978 657.9354534 2.750883712 1.459895154 0.034625294 1chr1_236045845_236048273 235.4615434 125.5655089 345.3575779 2.750417539 1.45965065 0.086348773 1chr22_24087362_24090054 237.0593406 126.419696 347.6989852 2.750354543 1.459617606 0.085582818 1chr7_22294682_22296796 249.8417183 133.2531931 366.4302436 2.749879648 1.459368479 0.079816559 1chr10_47002307_47005670 253.0373128 134.9615673 371.1130582 2.749768438 1.459310132 0.078469048 1chr12_691081_693888 253.0373128 134.9615673 371.1130582 2.749768438 1.459310132 0.078469048 1chr5_171863082_171867864 759.1119384 404.884702 1113.339175 2.749768438 1.459310132 0.016219456 1chr12_50224707_50228188 299.3734322 159.7329943 439.0138702 2.748423218 1.458604176 0.062337288 1chr7_69281721_69283111 312.15581 166.5664913 457.7451286 2.748122536 1.458446334 0.058796299 1chr11_66005323_66011137 648.2785783 345.9457897 950.6113668 2.747862223 1.45830967 0.020361337 1chr14_24560997_24563485 873.5679865 466.3861757 1280.749797 2.746114409 1.457391732 0.013497131 1chr16_24964061_24968417 446.3707766 238.3182107 654.4233424 2.746006445 1.457335011 0.035182415 1chr14_89133395_89137846 540.6408126 288.7152516 792.5663736 2.745148963 1.456884438 0.02662634 1chr1_54022752_54029016 983.2306429 525.3250881 1441.136198 2.743322622 1.455924297 0.011627714 1chr1_17682489_17683628 62.31409163 33.31329827 91.31488499 2.74109409 1.45475185 0.341555586 1chr1_19412751_19414976 178.9532888 95.66895913 262.2376184 2.74109409 1.45475185 0.123668905 1chr1_47140944_47142180 83.08545551 44.41773102 121.75318 2.74109409 1.45475185 0.272337363 1chr1_53094668_53095686 54.32510552 29.04236259 79.60784846 2.74109409 1.45475185 0.375952466 1chr1_56183755_56185454 129.4215749 69.18915794 189.6539919 2.74109409 1.45475185 0.178556517 1chr1_64366294_64367336 55.92290274 29.89654973 81.94925576 2.74109409 1.45475185 0.368634247 1chr1_64471765_64473454 142.2039527 76.02265502 208.3852504 2.74109409 1.45475185 0.161292275 1chr1_67965991_67967056 57.52069997 30.75073686 84.29066307 2.74109409 1.45475185 0.361545925 1chr1_86076882_86079481 236.4739887 126.419696 346.5282815 2.74109409 1.45475185 0.08687714 1chr1_101048949_101050003 59.11849719 31.604924 86.63207038 2.74109409 1.45475185 0.354676666 1chr1_116466690_116467864 63.91188885 34.1674854 93.6562923 2.74109409 1.45475185 0.335285484 1chr10_21674197_21675476 75.0964694 40.14679535 110.0461435 2.74109409 1.45475185 0.296096852 1chr11_134261935_134264160 143.8017499 76.87684216 210.7266577 2.74109409 1.45475185 0.159313809 1chr12_72032892_72034727 115.0413999 61.50147372 168.5813261 2.74109409 1.45475185 0.201566713 1chr12_93593235_93595049 156.5841277 83.71033924 229.4579161 2.74109409 1.45475185 0.144731524 1chr13_71438337_71439565 65.50968607 35.02167254 95.99769961 2.74109409 1.45475185 0.329197776 1chr13_78634536_78635642 59.11849719 31.604924 86.63207038 2.74109409 1.45475185 0.354676666 1chr14_39712353_39713977 124.6281833 66.62659653 182.62977 2.74109409 1.45475185 0.185761854 1chr14_87253801_87254921 67.10748329 35.87585967 98.33910692 2.74109409 1.45475185 0.323284684 1chr15_100047100_100048229 65.50968607 35.02167254 95.99769961 2.74109409 1.45475185 0.329197776 1chr16_25159746_25160911 57.52069997 30.75073686 84.29066307 2.74109409 1.45475185 0.361545925 1chr16_56424669_56426099 86.28104995 46.12610529 126.4359946 2.74109409 1.45475185 0.263656731 1chr17_66545031_66546535 79.88986106 42.70935675 117.0703654 2.74109409 1.45475185 0.281470867 1chr17_75973722_75974894 63.91188885 34.1674854 93.6562923 2.74109409 1.45475185 0.335285484 1chr18_34073223_34076438 202.9202471 108.4817662 297.3587281 2.74109409 1.45475185 0.105883206 1chr19_33755353_33756373 57.52069997 30.75073686 84.29066307 2.74109409 1.45475185 0.361545925 1chr2_69412440_69413657 70.30307774 37.58423394 103.0219215 2.74109409 1.45475185 0.311953527 1chr2_154353846_154355439 89.47664439 47.83447956 131.1188092 2.74109409 1.45475185 0.25539771 1chr2_163617791_163620469 156.5841277 83.71033924 229.4579161 2.74109409 1.45475185 0.144731524 1chr2_171363587_171364912 97.4656305 52.10541524 142.8258458 2.74109409 1.45475185 0.236414679 1chr2_171967589_171968888 75.0964694 40.14679535 110.0461435 2.74109409 1.45475185 0.296096852 1chr2_233326740_233330160 274.8211221 146.9201872 402.7220569 2.74109409 1.45475185 0.070939505 1chr2_242790293_242793663 433.003047 231.4847136 634.5213803 2.74109409 1.45475185 0.037105803 1chr20_24841756_24843925 151.790736 81.14777783 222.4336942 2.74109409 1.45475185 0.14995294 1chr22_36800553_36803416 223.691611 119.5861989 327.7970231 2.74109409 1.45475185 0.093447477 1chr3_9206405_9207119 154.9863305 82.8561521 227.1165088 2.74109409 1.45475185 0.1464409 1chr3_9548234_9549285 65.50968607 35.02167254 95.99769961 2.74109409 1.45475185 0.329197776 1chr3_29563170_29564326 65.50968607 35.02167254 95.99769961 2.74109409 1.45475185 0.329197776 1chr3_33276892_33278135 81.48765829 43.56354389 119.4117727 2.74109409 1.45475185 0.276845429 1chr3_37562047_37563504 89.47664439 47.83447956 131.1188092 2.74109409 1.45475185 0.25539771 1chr3_53868801_53870493 135.8127638 72.60590648 199.0196211 2.74109409 1.45475185 0.16959132 1chr3_66705522_66707140 278.0167165 148.6285615 407.4048715 2.74109409 1.45475185 0.069820728 1chr4_3320888_3323739 263.6365415 140.9408773 386.3322057 2.74109409 1.45475185 0.075084573 1chr4_48842473_48844215 110.2480083 58.93891232 161.5571042 2.74109409 1.45475185 0.210249594 1chr4_146399075_146400311 78.29206384 41.85516962 114.7289581 2.74109409 1.45475185 0.286218102 1chr5_29084867_29085885 52.7273083 28.18817546 77.26644115 2.74109409 1.45475185 0.383512216 1chr6_73447036_73450171 188.5400721 100.7940819 276.2860623 2.74109409 1.45475185 0.116043834 1chr6_34407637_34408541 57.52069997 30.75073686 84.29066307 2.74109409 1.45475185 0.361545925 1chr6_41006354_41007817 97.4656305 52.10541524 142.8258458 2.74109409 1.45475185 0.236414679 1chr6_76603004_76604690 121.4325888 64.91822226 177.9469554 2.74109409 1.45475185 0.190813963 1chr6_109084968_109086676 127.8237777 68.3349708 187.3125846 2.74109409 1.45475185 0.180910376 1chr6_109533635_109534871 87.87884717 46.98029243 128.7774019 2.74109409 1.45475185 0.259476356 1chr6_128366935_128368505 103.8568194 55.52216378 152.191475 2.74109409 1.45475185 0.222745089 1chr7_17771480_17772689 76.69426662 41.00098248 112.3875508 2.74109409 1.45475185 0.291091794 1chr7_26419102_26420543 97.4656305 52.10541524 142.8258458 2.74109409 1.45475185 0.236414679 1chr8_116801339_116802878 129.4215749 69.18915794 189.6539919 2.74109409 1.45475185 0.178556517 1chr8_120796334_120797417 70.30307774 37.58423394 103.0219215 2.74109409 1.45475185 0.311953527 1chr8_128188940_128190094 76.69426662 41.00098248 112.3875508 2.74109409 1.45475185 0.291091794 1chr9_71563920_71566016 135.8127638 72.60590648 199.0196211 2.74109409 1.45475185 0.16959132 1chr9_5870422_5871970 84.68325273 45.27191816 124.0945873 2.74109409 1.45475185 0.267942464 1chr9_79206697_79210359 246.0607721 131.5448188 360.5767254 2.74109409 1.45475185 0.082405908 1chr9_127682329_127683419 65.50968607 35.02167254 95.99769961 2.74109409 1.45475185 0.329197776 1chrX_76833914_76835374 94.27003606 50.39704097 138.1430311 2.74109409 1.45475185 0.243738332 1chrX_132092497_132096802 391.4603192 209.2758481 573.6447903 2.74109409 1.45475185 0.042969048 1chr5_16826392_16831528 521.8943395 279.3191932 764.4694859 2.736902814 1.452544207 0.028486639 1chr2_170147418_170152317 435.6132896 233.1930879 638.0334913 2.736073771 1.452107129 0.037112992 1chr1_168211418_168214346 403.6573452 216.1093452 591.2053451 2.735676908 1.451897853 0.041481475 1chr12_104813985_104818403 362.1146174 193.9004797 530.3287552 2.735056438 1.451570603 0.048562015 1chr7_408548_411731 307.7895119 164.8581171 450.7209067 2.73399281 1.451009449 0.061246589 1chr11_60949694_60955513 542.0803516 290.4236259 793.7370772 2.733032048 1.450502377 0.027167551 1chr1_57261352_57264191 266.2467841 142.6492516 389.8443167 2.732887221 1.450425925 0.074916744 1chr12_66995904_66998457 242.2798258 129.8364445 354.7232071 2.732077333 1.44999832 0.085107545 1chr2_160628417_160631127 226.3018536 121.2945732 331.309134 2.73144235 1.449662974 0.093147427 1chr14_105922881_105925657 224.7040564 120.440386 328.9677267 2.731373898 1.449626818 0.094015337 1chr14_21896924_21898104 210.3238814 112.7527018 307.8950609 2.730711158 1.44927672 0.102419277 1chr1_115082040_115084958 205.5304897 110.1901404 300.870839 2.730469694 1.449149144 0.105478461 1chr1_114057342_114059901 189.5525175 101.6482691 277.4567659 2.729576888 1.448677336 0.116753397 1chr21_32680554_32684279 379.105035 203.2965381 554.9135319 2.729576888 1.448677336 0.045881269 1chr3_13609462_13611247 183.1613286 98.23152053 268.0911367 2.729176289 1.448465588 0.121785017 1chr15_58584458_58587106 173.5745453 93.10639772 254.0426929 2.728520264 1.448118758 0.129975555 1chr7_72418520_72421982 520.7236359 279.3191932 762.1280786 2.728520264 1.448118758 0.029049797 1chr7_1025434_1030039 1133.534159 608.1812402 1658.887077 2.72761961 1.447642462 0.010124609 1chr1_59387312_59389063 157.5965731 84.56452637 230.6286198 2.72725018 1.44744705 0.145617149 1chr7_1730442_1734111 598.4303479 321.1743628 875.686333 2.726513802 1.447057459 0.023880215 1chr2_203203591_203204584 146.4119925 78.58521643 214.2387686 2.726196839 1.446889733 0.158333611 1chr19_6220417_6224854 986.5368144 529.5960237 1443.477605 2.725620172 1.44658453 0.01210148 1chr1_59478515_59480233 140.0208036 75.16846788 204.8731394 2.725519692 1.446531344 0.16637199 1chr1_52584215_52585944 136.8252092 73.46009361 200.1903248 2.725157496 1.446339611 0.17062551 1chr20_20769818_20777101 679.3326544 364.7379067 993.927402 2.72504553 1.446280334 0.020018359 1chr10_30993781_30995461 135.227412 72.60590648 197.8489175 2.724970007 1.446240351 0.17281444 1chr6_41730531_41737886 1614.739958 866.9999421 2362.479973 2.724890578 1.446198297 0.006848391 1chr3_61775283_61777205 132.0318175 70.89753221 193.1661029 2.724581475 1.446034633 0.17732216 1chr14_35266402_35268183 130.4340203 70.04334507 190.8246956 2.724380101 1.445928 0.179643274 1chr20_19887525_19889075 130.4340203 70.04334507 190.8246956 2.724380101 1.445928 0.179643274 1chr1_54866803_54871407 640.985521 344.2374154 937.7336267 2.724089784 1.445774254 0.021769772 1chr2_86540407_86542332 125.6406287 67.48078367 183.8004736 2.723745393 1.445591851 0.186890447 1chr5_132213186_132215018 125.6406287 67.48078367 183.8004736 2.723745393 1.445591851 0.186890447 1chr11_71053924_71055410 122.4450342 65.7724094 179.117659 2.723294778 1.445353152 0.191971663 1chr2_232544517_232548174 241.694474 129.8364445 353.5525034 2.723060576 1.445229076 0.086376824 1chr3_59422967_59424913 119.2494398 64.06403513 174.4348444 2.722820129 1.44510168 0.197265968 1chr7_45163346_45164840 114.4560481 61.50147372 167.4106225 2.722058714 1.444698186 0.205634246 1chr12_16645316_16646892 112.8582509 60.64728659 165.0692152 2.72179061 1.444556083 0.208544208 1chr7_70684673_70686162 111.2604537 59.79309945 162.7278079 2.721514846 1.444409906 0.211517428 1chr1_227737075_227738608 106.467062 57.23053805 155.7035859 2.720638164 1.443945095 0.22083604 1chr8_81567572_81570129 313.0099971 168.2748656 457.7451286 2.720222815 1.443724828 0.061067411 1chr13_65330007_65331903 100.0758731 53.81378951 146.3379567 2.719339375 1.443256212 0.234271075 1chr1_91442733_91444192 98.47807589 52.95960237 143.9965494 2.718988492 1.443070045 0.237825768 1chr8_102771761_102774093 574.8904831 309.2157429 840.5652234 2.71837784 1.442745997 0.025714615 1chr22_47591051_47592543 95.28248145 51.2512281 139.3137348 2.718251639 1.442679018 0.245188392 1chr5_36003480_36004995 95.28248145 51.2512281 139.3137348 2.718251639 1.442679018 0.245188392 1chr11_63028371_63029780 93.68468423 50.39704097 136.9723275 2.717864479 1.442473521 0.249001965 1chr7_22317684_22320364 187.3693685 100.7940819 273.944655 2.717864479 1.442473521 0.119948645 1chr12_8922149_8924463 185.7715712 99.9398948 271.6032477 2.717665935 1.442368126 0.121224119 1chr22_23791666_23793571 184.173774 99.08570767 269.2618404 2.717463968 1.442260906 0.122520366 1chrX_17453183_17454439 92.08688701 49.54285383 134.6309202 2.717463968 1.442260906 0.252907688 1chr20_24702237_24704114 182.5759768 98.23152053 266.9204331 2.717258489 1.442151814 0.123837834 1chr21_16511930_16514836 268.2716749 144.3576258 392.185724 2.716764852 1.4418897 0.075807016 1chr1_236876882_236878508 88.89129256 47.83447956 129.9481056 2.716620035 1.441812795 0.261008349 1chr6_107721149_107722599 88.89129256 47.83447956 129.9481056 2.716620035 1.441812795 0.261008349 1chr1_46829428_46831415 176.1847879 94.81477199 257.5548038 2.716399548 1.441695698 0.129329378 1chr1_86128531_86129910 85.69569812 46.12610529 125.265291 2.715713589 1.441331335 0.26951745 1chr13_49982376_49983801 85.69569812 46.12610529 125.265291 2.715713589 1.441331335 0.26951745 1chr6_10932359_10933752 85.69569812 46.12610529 125.265291 2.715713589 1.441331335 0.26951745 1chr14_99722522_99723410 169.793599 91.39802345 248.1891746 2.715476388 1.441205319 0.135199903 1chr4_82728309_82730302 168.1958018 90.54383632 245.8477673 2.715234712 1.441076914 0.136730824 1chr8_102802530_102803771 84.0979009 45.27191816 122.9238836 2.715234712 1.441076914 0.273934354 1chr1_64506712_64508889 166.5980046 89.68964918 243.50636 2.714988432 1.440946051 0.138288227 1chr12_31133204_31134994 166.5980046 89.68964918 243.50636 2.714988432 1.440946051 0.138288227 1chr3_66858446_66860905 249.0981083 134.1073802 364.0888363 2.714905293 1.440901872 0.08389381 1chr16_15010668_15014656 326.8048203 175.9625498 477.6470907 2.714481526 1.440676665 0.058007782 1chr22_28368433_28370496 161.8046129 87.12708778 236.4821381 2.714220618 1.440537991 0.143125538 1chrX_72009511_72010837 80.90230646 43.56354389 118.241069 2.714220618 1.440537991 0.283112873 1chr14_32611303_32613624 158.6090185 85.41871351 231.7993234 2.713683149 1.440252281 0.146494547 1chr5_69499066_69501329 158.6090185 85.41871351 231.7993234 2.713683149 1.440252281 0.146494547 1chr1_15159644_15161079 77.70671202 41.85516962 113.5582544 2.713123742 1.439954849 0.292780129 1chr2_208999404_209000547 77.70671202 41.85516962 113.5582544 2.713123742 1.439954849 0.292780129 1chr5_71131801_71133037 77.70671202 41.85516962 113.5582544 2.713123742 1.439954849 0.292780129 1chr8_90933641_90935814 233.120136 125.5655089 340.6747632 2.713123742 1.439954849 0.091702321 1chr8_99297019_99299856 310.8268481 167.4206785 454.2330177 2.713123742 1.439954849 0.062322669 1chr1_113218089_113219951 152.2178296 82.00196497 222.4336942 2.712541026 1.439644958 0.153603887 1chr2_228486108_228487498 76.10891479 41.00098248 111.2168471 2.712541026 1.439644958 0.297808828 1chr1_85139595_85141881 150.6200324 81.14777783 220.0922869 2.712240468 1.439485094 0.155463019 1chr3_71432602_71435199 225.1311499 121.2945732 328.9677267 2.712138871 1.439431051 0.09603448 1chr1_29044851_29046058 74.51111757 40.14679535 108.8754398 2.711933514 1.43932181 0.302974381 1chr4_113191341_113192542 74.51111757 40.14679535 108.8754398 2.711933514 1.43932181 0.302974381 1chr6_68853924_68855658 147.4244379 79.43940356 215.4094723 2.71161996 1.439154995 0.159285148 1chr4_185708804_185711426 218.7399611 117.8778246 319.6020975 2.711299589 1.438984535 0.099731017 1chr10_47073926_47075863 508.7954452 274.1940704 743.3968201 2.711206771 1.438935145 0.031059937 1chr4_178447215_178450579 435.8821249 234.9014621 636.8627876 2.711191245 1.438926883 0.038767474 1chr5_60692662_60694759 144.2288435 77.73102929 210.7266577 2.710972177 1.438810307 0.163251593 1chr1_84305087_84306301 71.31552313 38.43842108 104.1926252 2.710637489 1.438632185 0.313738197 1chr1_109275339_109276451 71.31552313 38.43842108 104.1926252 2.710637489 1.438632185 0.313738197 1chr11_129774780_129776094 71.31552313 38.43842108 104.1926252 2.710637489 1.438632185 0.313738197 1chr6_164604825_164606073 71.31552313 38.43842108 104.1926252 2.710637489 1.438632185 0.313738197 1chr8_101834329_101840297 1136.254978 612.4521758 1660.057781 2.710510055 1.438564359 0.010557646 1chr22_18263356_18265422 210.7509749 113.606889 307.8950609 2.710179495 1.438388404 0.104667113 1chr2_211792728_211793982 69.71772591 37.58423394 101.8512179 2.709945293 1.438263728 0.319348332 1chr1_48115207_48118580 408.7195721 220.3802808 597.0588634 2.709220903 1.437878032 0.042649187 1chr15_81393941_81395263 68.11992869 36.73004681 99.50981057 2.709220903 1.437878032 0.32511912 1chr7_12894822_12896024 68.11992869 36.73004681 99.50981057 2.709220903 1.437878032 0.32511912 1chr10_49440883_49442177 66.52213147 35.87585967 97.16840326 2.708462017 1.437473859 0.331057416 1chr5_171289101_171291974 266.0885259 143.5034387 388.673613 2.708462017 1.437473859 0.077519285 1chr5_177388053_177389233 66.52213147 35.87585967 97.16840326 2.708462017 1.437473859 0.331057416 1chr8_145173243_145174525 66.52213147 35.87585967 97.16840326 2.708462017 1.437473859 0.331057416 1chr22_41656199_41663686 991.4407831 534.7211465 1448.16042 2.708253506 1.437362788 0.012561014 1chr19_7504827_7506399 131.4464657 70.89753221 191.9953992 2.70806886 1.437264424 0.180716229 1chr21_46648527_46649891 64.92433425 35.02167254 94.82699595 2.707666113 1.437049849 0.337170497 1chr4_170019541_170022783 258.0995398 139.232503 376.9665765 2.707461034 1.436940574 0.080839748 1chr17_9143405_9146252 386.350411 208.421661 564.2791611 2.707392113 1.436903849 0.046348644 1chr5_69517246_69519168 128.2508713 69.18915794 187.3125846 2.707253422 1.436829942 0.185525822 1chr7_122327998_122331455 319.8282796 172.5458013 467.1107578 2.707169658 1.436785304 0.060447692 1chr14_33786296_33787440 63.32653702 34.1674854 92.48558865 2.706830414 1.436604504 0.343466103 1chr19_32962744_32963812 63.32653702 34.1674854 92.48558865 2.706830414 1.436604504 0.343466103 1chr2_211405546_211406731 63.32653702 34.1674854 92.48558865 2.706830414 1.436604504 0.343466103 1chr5_28748049_28749306 63.32653702 34.1674854 92.48558865 2.706830414 1.436604504 0.343466103 1chr5_133727152_133731026 251.7083509 135.8157545 367.6009473 2.706614919 1.436489644 0.083657823 1chr12_56296127_56297105 61.7287398 33.31329827 90.14418134 2.705951858 1.436136172 0.34995247 1chr2_169011060_169014664 308.643699 166.5664913 450.7209067 2.705951858 1.436136172 0.063609571 1chr2_186624425_186625748 61.7287398 33.31329827 90.14418134 2.705951858 1.436136172 0.34995247 1chr7_30803903_30805512 121.8596824 65.7724094 177.9469554 2.705495465 1.435892823 0.195747289 1chr8_59392112_59394620 243.7193648 131.5448188 355.8939107 2.705495465 1.435892823 0.087399716 1chr8_103727148_103730701 545.9718749 294.6945616 797.2491882 2.705340689 1.435810287 0.028413044 1chr12_89674793_89675898 60.13094258 32.45911113 87.80277403 2.705027062 1.435643028 0.35663838 1chr17_62271589_62272624 60.13094258 32.45911113 87.80277403 2.705027062 1.435643028 0.35663838 1chr4_189006162_189007236 60.13094258 32.45911113 87.80277403 2.705027062 1.435643028 0.35663838 1chr8_118008618_118009754 60.13094258 32.45911113 87.80277403 2.705027062 1.435643028 0.35663838 1chrX_106754261_106755308 60.13094258 32.45911113 87.80277403 2.705027062 1.435643028 0.35663838 1chr14_99695720_99696853 178.7950305 96.52314626 261.0669148 2.704707885 1.435472788 0.128684172 1chr12_16102745_16103778 56.93534814 30.75073686 83.11995942 2.703023339 1.434573969 0.370646983 1chr16_24917314_24918400 56.93534814 30.75073686 83.11995942 2.703023339 1.434573969 0.370646983 1chr3_66742070_66745269 227.7413926 123.0029474 332.4798377 2.703023339 1.434573969 0.095703244 1chr3_176350597_176352364 113.8706963 61.50147372 166.2399188 2.703023339 1.434573969 0.209775638 1chr7_69581000_69582132 56.93534814 30.75073686 83.11995942 2.703023339 1.434573969 0.370646983 1chr8_136832333_136833395 56.93534814 30.75073686 83.11995942 2.703023339 1.434573969 0.370646983 1chr1_154739195_154742064 283.0789435 152.8994972 413.2583898 2.702810653 1.434460446 0.071896757 1chr1_118294916_118297964 281.4811463 152.04531 410.9169825 2.702595577 1.43434564 0.072470187 1chr3_49035107_49037225 393.7540453 212.6925966 574.815494 2.702564655 1.434329133 0.045488858 1chr1_98385577_98387514 112.2728991 60.64728659 163.8985115 2.702487131 1.434287748 0.212764724 1chr7_94492717_94494391 112.2728991 60.64728659 163.8985115 2.702487131 1.434287748 0.212764724 1chr12_67041462_67045507 335.2209 181.0876726 489.3541273 2.702305023 1.434190528 0.057055018 1chr12_121856147_121857918 167.61045 90.54383632 244.6770636 2.702305023 1.434190528 0.139110408 1chr13_38619058_38620214 55.33755092 29.89654973 80.77855211 2.701935603 1.43399329 0.377990443 1chr8_110951868_110952884 55.33755092 29.89654973 80.77855211 2.701935603 1.43399329 0.377990443 1chr8_11852008_11854458 166.0126528 89.68964918 242.3356563 2.701935603 1.43399329 0.140704042 1chr2_152778696_152780479 109.0773046 58.93891232 159.2156969 2.701368089 1.433690235 0.218941133 1chr1_93636199_93638952 162.8170583 87.98127491 237.6528417 2.701175244 1.43358724 0.143975552 1chr14_74435239_74439586 377.7760731 204.1507253 551.4014209 2.700952545 1.433468292 0.048362694 1chr4_8504222_8507052 268.6987685 145.211813 392.185724 2.700783883 1.4333782 0.07732621 1chr1_110718517_110719678 213.3612176 115.3152632 311.4071719 2.700485288 1.433218689 0.104261013 1chrX_67707218_67711271 316.0473333 170.837427 461.2572396 2.699977678 1.43294748 0.062122826 1chr12_102295770_102298405 210.1656231 113.606889 306.7243573 2.69987463 1.432892416 0.106321928 1chr1_46754208_46755966 104.283913 56.37635091 152.191475 2.699562361 1.432725544 0.228732284 1chr1_59057283_59059129 156.4258694 84.56452637 228.2872125 2.699562361 1.432725544 0.150874499 1chr7_3037732_3039251 156.4258694 84.56452637 228.2872125 2.699562361 1.432725544 0.150874499 1chrX_149516166_149517703 104.283913 56.37635091 152.191475 2.699562361 1.432725544 0.228732284 1chrX_152853886_152854898 52.14195648 28.18817546 76.09573749 2.699562361 1.432725544 0.39341338 1chr21_32841301_32844345 308.0583472 166.5664913 449.550203 2.698923412 1.432384037 0.064447624 1chr5_20366074_20368622 308.0583472 166.5664913 449.550203 2.698923412 1.432384037 0.064447624 1chr11_126009309_126011969 353.8091148 191.3379183 516.2803113 2.698264495 1.432031774 0.053258747 1chr6_54372526_54373474 50.54415925 27.33398832 73.75433019 2.698264495 1.432031774 0.401518596 1chr12_31090926_31096231 802.3153592 433.9270646 1170.703654 2.697927254 1.431851448 0.016952232 1chr18_42264957_42267097 200.5788398 108.4817662 292.6759134 2.697927254 1.431851448 0.112892769 1chr7_2675350_2681687 2330.837163 1260.780211 3400.894114 2.697452009 1.431597293 0.005295666 0.9889824chr8_119502696_119505294 244.7318102 132.3990059 357.0646144 2.696882895 1.431292878 0.087908151 1chr22_24938007_24943290 1318.35618 713.2462578 1923.466103 2.696777 1.431236228 0.009217625 1chr14_39917592_39919007 96.29492684 52.10541524 140.4844385 2.696158121 1.430905108 0.246609056 1chr15_39332337_39334009 96.29492684 52.10541524 140.4844385 2.696158121 1.430905108 0.246609056 1chr3_60788983_60790346 96.29492684 52.10541524 140.4844385 2.696158121 1.430905108 0.246609056 1chr5_90414746_90416304 96.29492684 52.10541524 140.4844385 2.696158121 1.430905108 0.246609056 1chr5_118721783_118723309 96.29492684 52.10541524 140.4844385 2.696158121 1.430905108 0.246609056 1chr3_9300412_9303383 287.2869833 155.4620586 419.111908 2.69591122 1.430772988 0.071176161 1chr1_84854743_84856509 94.69712962 51.2512281 138.1430311 2.695409188 1.430504304 0.250441486 1chr2_230696700_230699187 142.0456944 76.87684216 207.2145467 2.695409188 1.430504304 0.168356274 1chr5_18244093_18245389 94.69712962 51.2512281 138.1430311 2.695409188 1.430504304 0.250441486 1chrX_23788917_23790175 94.69712962 51.2512281 138.1430311 2.695409188 1.430504304 0.250441486 1chr2_192507035_192509608 186.1986648 100.7940819 271.6032477 2.694634868 1.430089796 0.123975573 1chr3_160812693_160814246 137.2523028 74.31428075 200.1903248 2.693833847 1.429660869 0.174870998 1chr5_16726282_16729156 594.7599787 322.0285499 867.4914074 2.693833847 1.429660869 0.025778428 1chr1_118399478_118401271 135.6545055 73.46009361 197.8489175 2.693284309 1.429366532 0.177127702 1chr13_100849316_100851267 134.0567083 72.60590648 195.5075102 2.692721841 1.429065207 0.179428853 1chr3_39062603_39064527 134.0567083 72.60590648 195.5075102 2.692721841 1.429065207 0.179428853 1chr7_132530638_132532219 88.30594074 47.83447956 128.7774019 2.692145981 1.428756642 0.266727788 1chr6_34948794_34951274 217.5692574 117.8778246 317.2606902 2.691436588 1.428376435 0.102856382 1chr5_16718908_16723251 476.0958907 257.9645148 694.2272667 2.691173502 1.428235406 0.035467736 1chr6_82598821_82600844 129.2633167 70.04334507 188.4832883 2.690952125 1.428116724 0.186611084 1chr19_39202353_39205721 773.9821028 419.4058833 1128.558322 2.690850003 1.428061972 0.018080223 1chr14_37200332_37201468 85.1103463 46.12610529 124.0945873 2.690333088 1.427784803 0.275492838 1chr7_96258950_96260231 85.1103463 46.12610529 124.0945873 2.690333088 1.427784803 0.275492838 1chr8_136601571_136602943 85.1103463 46.12610529 124.0945873 2.690333088 1.427784803 0.275492838 1chrX_106762429_106764446 127.6655194 69.18915794 186.1418809 2.690333088 1.427784803 0.189102333 1chr1_174154998_174156956 168.6228954 91.39802345 245.8477673 2.689858686 1.427530382 0.139925645 1chr1_28184127_28186290 209.5802713 113.606889 305.5536536 2.689569765 1.427375412 0.108001238 1chr7_27934974_27939890 628.7408139 340.8206669 916.6609609 2.689569765 1.427375412 0.024072815 1chr1_108377458_108378990 83.51254907 45.27191816 121.75318 2.689375333 1.427271114 0.280044012 1chr14_40588067_40589250 83.51254907 45.27191816 121.75318 2.689375333 1.427271114 0.280044012 1chr4_31366756_31368111 83.51254907 45.27191816 121.75318 2.689375333 1.427271114 0.280044012 1chr5_174771230_174772403 83.51254907 45.27191816 121.75318 2.689375333 1.427271114 0.280044012 1chr15_49465837_49468551 165.4273009 89.68964918 241.1649527 2.688882774 1.42700686 0.143158787 1chr9_71328726_71330928 206.3846769 111.8985147 300.870839 2.688783134 1.426953398 0.110169958 1chr3_16439325_16442489 329.2568046 178.5251112 479.988498 2.688632959 1.426872818 0.059748116 1chr1_241877176_241878094 122.8721278 66.62659653 179.117659 2.688380742 1.426737474 0.196886732 1chr18_73608019_73609350 81.91475185 44.41773102 119.4117727 2.688380742 1.426737474 0.284713143 1chr3_40624483_40626171 122.8721278 66.62659653 179.117659 2.688380742 1.426737474 0.196886732 1chr5_79460657_79462799 163.8295037 88.83546205 238.8235454 2.688380742 1.426737474 0.144818006 1chr1_68297904_68299903 119.6765333 64.91822226 174.4348444 2.686993549 1.425992857 0.202350022 1chr1_48304249_48308383 314.8766296 170.837427 458.9158323 2.686272208 1.425605505 0.063747807 1chr3_87347180_87348750 78.71915741 42.70935675 114.7289581 2.686272208 1.425605505 0.294423153 1chr6_3494055_3495799 118.0787361 64.06403513 172.0934371 2.686272208 1.425605505 0.205168512 1chr7_72838872_72840129 78.71915741 42.70935675 114.7289581 2.686272208 1.425605505 0.294423153 1chr1_82135550_82137378 155.8405176 84.56452637 227.1165088 2.685718452 1.425308073 0.153568165 1chr3_13708483_13709702 77.12136019 41.85516962 112.3875508 2.685153394 1.425004507 0.299473562 1chr7_1021612_1023412 500.4899426 271.6315089 729.3483763 2.685065437 1.424957248 0.033436999 1chr7_73390968_73395320 306.8876435 166.5664913 447.2087957 2.684866519 1.424850365 0.066155533 1chr1_47895778_47902473 612.1774898 332.2787955 892.0761842 2.684721975 1.424772693 0.025238328 1chr20_36255209_36257137 190.4067046 103.3566433 277.4567659 2.684459914 1.424631862 0.122079052 1chr1_164214352_164215572 75.52356297 41.00098248 110.0461435 2.683987963 1.424378201 0.304661023 1chr2_241614495_241617823 302.0942519 164.0039299 440.1845738 2.683987963 1.424378201 0.067669539 1chr3_89542306_89544078 151.0471259 82.00196497 220.0922869 2.683987963 1.424378201 0.159208479 1chr6_138246738_138250193 264.3324704 143.5034387 385.1615021 2.683987963 1.424378201 0.080864636 1chr8_57309036_57311399 151.0471259 82.00196497 220.0922869 2.683987963 1.424378201 0.159208479 1chr8_9465087_9467457 264.3324704 143.5034387 385.1615021 2.683987963 1.424378201 0.080864636 1chr1_116150630_116152086 111.6875472 60.64728659 162.7278079 2.683183651 1.423945804 0.217061903 1chr1_246037725_246040764 295.703063 160.5871814 430.8189446 2.682772939 1.423724955 0.069774335 1chr1_80823591_80824709 73.92576575 40.14679535 107.7047361 2.682772939 1.423724955 0.309990977 1chr10_7260087_7261514 73.92576575 40.14679535 107.7047361 2.682772939 1.423724955 0.309990977 1chr15_65984786_65985977 73.92576575 40.14679535 107.7047361 2.682772939 1.423724955 0.309990977 1chr2_135092353_135093746 73.92576575 40.14679535 107.7047361 2.682772939 1.423724955 0.309990977 1chr9_85888993_85890326 73.92576575 40.14679535 107.7047361 2.682772939 1.423724955 0.309990977 1chr1_93397004_93401981 440.359 239.1723978 641.5456023 2.682356359 1.423500916 0.040245109 1chr19_17867510_17869011 110.08975 59.79309945 160.3864006 2.682356359 1.423500916 0.220199468 1chr3_25777958_25781501 256.3434843 139.232503 373.4544655 2.682236241 1.42343631 0.084379684 1chr11_1660919_1662215 72.32796853 39.29260821 105.3633288 2.681505088 1.423042991 0.315469168 1chr2_18363947_18365293 72.32796853 39.29260821 105.3633288 2.681505088 1.423042991 0.315469168 1chr1_120180739_120183787 179.2221241 97.3773334 261.0669148 2.680982377 1.422761736 0.131622226 1chr2_128386840_128390505 358.4442482 194.7546668 522.1338296 2.680982377 1.422761736 0.05378654 1chr6_43977410_43980710 393.0104352 213.5467838 572.4740867 2.68079002 1.422658221 0.047335592 1chr1_179755513_179757338 106.8941556 58.08472518 155.7035859 2.680628779 1.422571445 0.226685833 1chr2_210635976_210637254 70.7301713 38.43842108 103.0219215 2.680180888 1.422330373 0.321101675 1chr8_37249575_37251646 176.0265296 95.66895913 256.3841002 2.679908954 1.422183988 0.134557493 1chr5_169645497_169647663 139.8625454 76.02265502 203.7024358 2.67949647 1.421961915 0.173655905 1chr7_131376457_131377668 69.13237408 37.58423394 100.6805142 2.678796497 1.421584987 0.326894928 1chr11_67286969_67292275 653.5619593 355.3418482 951.7820705 2.678496989 1.421423674 0.023370887 1chr20_23182013_23189113 1021.007639 555.2216378 1486.79364 2.677838072 1.421068724 0.013079277 1chr2_170092908_170095261 169.6353408 92.25221059 247.0184709 2.677642838 1.420963537 0.14073402 1chr1_57018117_57019724 301.5089 164.0039299 439.0138702 2.676849697 1.420536135 0.068566559 1chr13_100418580_100419821 100.5029667 54.66797664 146.3379567 2.676849697 1.420536135 0.240566817 1chr2_159319341_159321788 201.0059334 109.3359533 292.6759134 2.676849697 1.420536135 0.115371369 1chr1_54847035_54852001 533.885426 290.4236259 777.3472261 2.676597758 1.420400346 0.03104937 1chr8_101230488_101233602 400.4140695 217.8177194 583.0104196 2.676597758 1.420400346 0.046451269 1chr18_60164280_60165314 65.93677964 35.87585967 95.99769961 2.675829945 1.419986432 0.338991362 1chr7_15795225_15796338 65.93677964 35.87585967 95.99769961 2.675829945 1.419986432 0.338991362 1chr8_81937434_81939596 197.8103389 107.627579 287.9930988 2.675829945 1.419986432 0.117763378 1chr19_33478665_33481222 229.1809315 124.7113217 333.6505413 2.67538293 1.419745401 0.098280104 1chr6_42526862_42527686 163.2441519 88.83546205 237.6528417 2.675202405 1.419648049 0.147350669 1chr7_21292230_21294310 130.2757621 70.89753221 189.6539919 2.67504363 1.419562422 0.187682195 1chr7_66985914_66987510 97.30737224 52.95960237 141.6551421 2.674777297 1.419418777 0.248001184 1chr1_56879001_56885066 907.1369427 493.7201641 1320.553721 2.674700807 1.41937752 0.015276309 1chr7_94684629_94686815 161.6463547 87.98127491 235.3114344 2.67456268 1.419303014 0.149078493 1chr18_46175021_46176150 64.33898242 35.02167254 93.6562923 2.674238136 1.419127941 0.345309463 1chr9_92237567_92239680 193.0169473 105.0650176 280.9688769 2.674238136 1.419127941 0.121495491 1chr6_164165978_164167831 127.0801676 69.18915794 184.9711773 2.673412754 1.418682596 0.19274126 1chr6_42206407_42209092 348.272113 189.629544 506.9146821 2.673184101 1.418559199 0.056673896 1chr18_9645716_9648257 315.3037232 171.6916141 458.9158323 2.67290767 1.418410003 0.064932052 1chr6_40550315_40555334 693.3486316 377.5507137 1009.14655 2.672876816 1.41839335 0.021833079 1chr12_83110060_83111550 94.1117778 51.2512281 136.9723275 2.672566738 1.418225974 0.255794276 1chr17_74117511_74119797 188.2235556 102.5024562 273.944655 2.672566738 1.418225974 0.125410466 1chr3_58625930_58627122 62.7411852 34.1674854 91.31488499 2.672566738 1.418225974 0.351818225 1chr8_129813309_129814507 62.7411852 34.1674854 91.31488499 2.672566738 1.418225974 0.351818225 1chr5_1545305_1547084 404.6221093 220.3802808 588.8639378 2.672035518 1.417939185 0.046142247 1chr3_53108801_53114365 588.0522733 320.3201756 855.7843709 2.67165304 1.417732661 0.027421107 1chr1_14290746_14292075 92.51398058 50.39704097 134.6309202 2.671405257 1.417598852 0.259832962 1chr3_8796488_8797836 92.51398058 50.39704097 134.6309202 2.671405257 1.417598852 0.259832962 1chr5_65619758_65621202 92.51398058 50.39704097 134.6309202 2.671405257 1.417598852 0.259832962 1chr2_239685013_239686046 61.14338798 33.31329827 88.97347769 2.670809626 1.417277145 0.35852636 1chr3_24006977_24008625 122.286776 66.62659653 177.9469554 2.670809626 1.417277145 0.200726892 1chr7_15111252_15112402 61.14338798 33.31329827 88.97347769 2.670809626 1.417277145 0.35852636 1chr9_103043569_103044714 61.14338798 33.31329827 88.97347769 2.670809626 1.417277145 0.35852636 1chr8_145309203_145311849 363.6647334 198.1714153 529.1580515 2.670203725 1.416949818 0.053659679 1chr21_41084971_41088075 241.3779575 131.5448188 351.2110961 2.669896841 1.416784 0.092678702 1chr1_116448229_116450832 271.1507528 147.7743744 394.5271313 2.669793955 1.416728404 0.0797523 1chr1_39848348_39853457 387.0463399 210.9842224 563.1084575 2.668960035 1.416277703 0.049336507 1chr10_8515525_8516569 59.54559075 32.45911113 86.63207038 2.668960035 1.416277703 0.365443162 1chr2_175124164_175125281 59.54559075 32.45911113 86.63207038 2.668960035 1.416277703 0.365443162 1chr2_189437956_189438993 59.54559075 32.45911113 86.63207038 2.668960035 1.416277703 0.365443162 1chr4_92751537_92752624 59.54559075 32.45911113 86.63207038 2.668960035 1.416277703 0.365443162 1chr5_73711294_73712293 59.54559075 32.45911113 86.63207038 2.668960035 1.416277703 0.365443162 1chr5_68444782_68445853 59.54559075 32.45911113 86.63207038 2.668960035 1.416277703 0.365443162 1chr8_122626849_122627974 59.54559075 32.45911113 86.63207038 2.668960035 1.416277703 0.365443162 1chr2_203116816_203118320 117.4933843 64.06403513 170.9227334 2.667998247 1.415757719 0.209225906 1chr12_3553125_3556785 556.0963288 303.2364329 808.9562247 2.667740868 1.415618536 0.029862989 1chr1_101685158_101686503 87.72058891 47.83447956 127.6066983 2.667671927 1.415581253 0.272557637 1chr2_186442963_186444483 87.72058891 47.83447956 127.6066983 2.667671927 1.415581253 0.272557637 1chr16_16411243_16413298 145.6683824 79.43940356 211.8973613 2.667408765 1.415438926 0.16818426 1chr12_40156215_40158475 173.8433806 94.81477199 252.8719892 2.667010466 1.415223486 0.138378062 1chr4_71796305_71797956 115.8955871 63.20984799 168.5813261 2.667010466 1.415223486 0.212180798 1chr8_11799142_11800728 115.8955871 63.20984799 168.5813261 2.667010466 1.415223486 0.212180798 1chrX_21817931_21819647 115.8955871 63.20984799 168.5813261 2.667010466 1.415223486 0.212180798 1chr18_47368420_47369916 144.0705852 78.58521643 209.555954 2.666607837 1.415005672 0.170296314 1chr1_28498991_28500302 86.12279169 46.98029243 125.265291 2.666336978 1.414859123 0.277014138 1chr2_192096598_192098150 86.12279169 46.98029243 125.265291 2.666336978 1.414859123 0.277014138 1chr7_131341551_131343211 142.472788 77.73102929 207.2145467 2.665789307 1.41456276 0.172448768 1chr3_85008042_85009577 112.6999926 61.50147372 163.8985115 2.664952587 1.414109866 0.218284328 1chr5_19000813_19002654 140.8749908 76.87684216 204.8731394 2.664952587 1.414109866 0.174642666 1chr7_102884890_102885877 56.34999631 30.75073686 81.94925576 2.664952587 1.414109866 0.37994319 1chr8_122418149_122419822 140.8749908 76.87684216 204.8731394 2.664952587 1.414109866 0.174642666 1chr6_41569648_41571448 392.852177 214.4009709 571.303383 2.664649235 1.413945634 0.048686811 1chr7_2967552_2969820 306.7293853 167.4206785 446.0380921 2.664175633 1.413689194 0.068279314 1chr1_7506364_7509740 446.0065788 243.4433335 648.5698242 2.664151098 1.413675907 0.040863415 1chr1_120408926_120414540 446.0065788 243.4433335 648.5698242 2.664151098 1.413675907 0.040863415 1chr16_28818280_28820065 139.2771936 76.02265502 202.5317321 2.664097065 1.413646647 0.176879087 1chr2_233075787_233077670 139.2771936 76.02265502 202.5317321 2.664097065 1.413646647 0.176879087 1chr1_105214018_105215606 111.1021954 60.64728659 161.5571042 2.663880172 1.413529188 0.221436858 1chr2_75181408_75182788 82.92719725 45.27191816 120.5824763 2.663515955 1.413331923 0.286273182 1chr13_30316676_30318546 137.6793963 75.16846788 200.1903248 2.663222099 1.413172747 0.179159146 1chr1_62164637_62168005 301.9359936 164.8581171 439.0138702 2.66298001 1.413041599 0.06985639 1chr19_18973206_18976759 356.6881927 194.7546668 518.6217186 2.662948864 1.413024725 0.055762867 1chr1_62351821_62354299 219.0087964 119.5861989 318.4313938 2.662777116 1.412931675 0.105640756 1chr12_14890541_14891537 54.75219909 29.89654973 79.60784846 2.662777116 1.412931675 0.387548442 1chr15_60591544_60592554 54.75219909 29.89654973 79.60784846 2.662777116 1.412931675 0.387548442 1chr3_9279335_9282733 219.0087964 119.5861989 318.4313938 2.662777116 1.412931675 0.105640756 1chr3_140900434_140901470 54.75219909 29.89654973 79.60784846 2.662777116 1.412931675 0.387548442 1chr4_129465586_129466620 54.75219909 29.89654973 79.60784846 2.662777116 1.412931675 0.387548442 1chr5_43615573_43616610 54.75219909 29.89654973 79.60784846 2.662777116 1.412931675 0.387548442 1chr10_32108083_32110459 136.0815991 74.31428075 197.8489175 2.662327018 1.412687791 0.181483995 1chr6_134435941_134437450 136.0815991 74.31428075 197.8489175 2.662327018 1.412687791 0.181483995 1chr1_60404944_60406234 81.32940003 44.41773102 118.241069 2.662024068 1.412523615 0.291084303 1chr21_48117315_48118790 107.906601 58.93891232 156.8742896 2.661642087 1.412316584 0.227953765 1chr7_122365317_122368824 242.3904029 132.3990059 352.3817998 2.661513939 1.412247122 0.093197222 1chr18_71673807_71675578 134.4838019 73.46009361 195.5075102 2.661411122 1.412191388 0.183854829 1chr8_101847091_101848696 134.4838019 73.46009361 195.5075102 2.661411122 1.412191388 0.183854829 1chr11_118051492_118052561 53.15440187 29.04236259 77.26644115 2.660473675 1.411683129 0.395406558 1chr17_43474333_43476212 159.4632056 87.12708778 231.7993234 2.660473675 1.411683129 0.153491546 1chr3_58394416_58395444 53.15440187 29.04236259 77.26644115 2.660473675 1.411683129 0.395406558 1chr3_87210333_87211286 53.15440187 29.04236259 77.26644115 2.660473675 1.411683129 0.395406558 1chr9_71527072_71528552 106.3088037 58.08472518 154.5328823 2.660473675 1.411683129 0.231322526 1chr8_126342642_126345765 290.7514131 158.8788071 422.624019 2.660040232 1.411448066 0.073765615 1chr1_109228481_109230484 157.8654084 86.27290064 229.4579161 2.659675453 1.411250212 0.155323644 1chr14_32741298_32744283 578.3072317 316.04924 840.5652234 2.659602103 1.411210424 0.028766882 1chr21_46867221_46871526 628.2660391 343.3832283 913.1488499 2.659270386 1.411030473 0.025704915 1chr9_116402580_116404000 78.13380558 42.70935675 113.5582544 2.658861267 1.410808503 0.301092885 1chr5_6418571_6420938 259.3808205 141.7950644 376.9665765 2.658531015 1.410629297 0.085804792 1chr8_37261595_37263883 181.2470149 99.08570767 263.4083221 2.658388665 1.410552047 0.133121926 1chr21_46121974_46125280 284.3602242 155.4620586 413.2583898 2.658258829 1.410481584 0.076154812 1chr10_524329_525955 103.1132093 56.37635091 149.8500677 2.658030633 1.410357731 0.238292305 1chr7_121684833_121685861 51.55660465 28.18817546 74.92503384 2.658030633 1.410357731 0.403530709 1chr10_31106136_31108323 281.1646297 153.7536843 408.5755752 2.657338437 1.40998198 0.077394843 1chr1_242475545_242476812 76.53600836 41.85516962 111.2168471 2.657183046 1.409897615 0.306300345 1chr12_104851719_104853765 153.0720167 83.71033924 222.4336942 2.657183046 1.409897615 0.161022922 1chr17_77829501_77832098 229.6080251 125.5655089 333.6505413 2.657183046 1.409897615 0.100322777 1chr7_72218681_72219923 76.53600836 41.85516962 111.2168471 2.657183046 1.409897615 0.306300345 1chr10_32062402_32064147 101.5154121 55.52216378 147.5086604 2.656752733 1.409663961 0.24189828 1chr18_47139571_47141129 101.5154121 55.52216378 147.5086604 2.656752733 1.409663961 0.24189828 1chr4_103700931_103702514 101.5154121 55.52216378 147.5086604 2.656752733 1.409663961 0.24189828 1chr1_10474394_10476148 126.4948158 69.18915794 183.8004736 2.65649242 1.409522596 0.196443494 1chr17_54415684_54417519 126.4948158 69.18915794 183.8004736 2.65649242 1.409522596 0.196443494 1chr5_141683630_141688410 479.4020622 262.2354505 696.568674 2.65627196 1.409402863 0.037513064 1chr1_111279848_111281799 99.91761485 54.66797664 145.1672531 2.6554349 1.408948161 0.245588461 1chr12_39796390_39798424 124.8970186 68.3349708 181.4590663 2.6554349 1.408948161 0.199117726 1chr13_100870231_100871623 74.93821114 41.00098248 108.8754398 2.6554349 1.408948161 0.311650376 1chr14_33813715_33816066 274.7734409 150.3369358 399.2099459 2.6554349 1.408948161 0.079970901 1chr5_77547676_77549397 124.8970186 68.3349708 181.4590663 2.6554349 1.408948161 0.199117726 1chr7_7785196_7786980 149.8764223 82.00196497 217.7508796 2.6554349 1.408948161 0.165000033 1chr7_44932710_44934293 124.8970186 68.3349708 181.4590663 2.6554349 1.408948161 0.199117726 1chr11_64554774_64556555 123.2992213 67.48078367 179.117659 2.654350606 1.408358945 0.201847557 1chr2_3328638_3330328 98.31981763 53.81378951 142.8258458 2.65407523 1.408209265 0.249365603 1chr7_38669860_38671528 171.6602316 93.96058486 249.3598782 2.65387746 1.408101757 0.142330521 1chr2_213734862_213736167 73.34041392 40.14679535 106.5340325 2.653612364 1.407957639 0.317148693 1chr2_220157797_220160492 220.0212418 120.440386 319.6020975 2.653612364 1.407957639 0.106229836 1chr4_102575685_102576606 48.36101021 26.47980119 70.24221923 2.6526717 1.407446136 0.420635075 1chr8_125055810_125059693 506.9917086 277.6108189 736.3725982 2.652535665 1.407372149 0.034972271 1chr1_86981071_86985111 457.0329011 250.2768306 663.7889717 2.652219025 1.407199921 0.040379345 1chr12_66297034_66298287 71.7426167 39.29260821 104.1926252 2.651710587 1.406923325 0.322801338 1chr13_82514728_82516088 71.7426167 39.29260821 104.1926252 2.651710587 1.406923325 0.322801338 1chr11_62740118_62743838 333.7336798 182.7960469 484.6713127 2.651432134 1.406771822 0.062166526 1chr20_5949995_5952586 166.8668399 91.39802345 242.3356563 2.651432134 1.406771822 0.147300973 1chr8_8225448_8229604 452.2395095 247.7142692 656.7647498 2.651299628 1.406699721 0.041040631 1chr1_81499686_81501229 95.12422319 52.10541524 138.1430311 2.651222152 1.406657562 0.257192409 1chr7_37546013_37548279 190.2484464 104.2108305 276.2860623 2.651222152 1.406657562 0.126825441 1chr2_9520120_9523689 544.1681382 298.1113101 790.2249663 2.650771505 1.406412316 0.031839386 1chr18_56003936_56006353 187.0528519 102.5024562 271.6032477 2.649724287 1.40584225 0.129571693 1chr2_2007648_2009556 163.6712454 89.68964918 237.6528417 2.649724287 1.40584225 0.150761985 1chr2_233755134_233756542 93.52642597 51.2512281 135.8016238 2.649724287 1.40584225 0.261248222 1chr7_66063002_66064792 163.6712454 89.68964918 237.6528417 2.649724287 1.40584225 0.150761985 1chr3_178746073_178748570 160.475651 87.98127491 232.9700271 2.647950116 1.404875944 0.15434748 1chr6_42729424_42731703 160.475651 87.98127491 232.9700271 2.647950116 1.404875944 0.15434748 1chr1_50654890_50656143 68.54702226 37.58423394 99.50981057 2.6476477 1.404711168 0.334595554 1chr11_48095854_48096914 68.54702226 37.58423394 99.50981057 2.6476477 1.404711168 0.334595554 1chr2_242062775_242064206 137.0940445 75.16846788 199.0196211 2.6476477 1.404711168 0.182492816 1chr22_27800908_27803207 137.0940445 75.16846788 199.0196211 2.6476477 1.404711168 0.182492816 1chr6_39123857_39125099 68.54702226 37.58423394 99.50981057 2.6476477 1.404711168 0.334595554 1chr5_133216401_133221782 750.8216504 411.7181991 1089.925102 2.647259956 1.404499872 0.02080873 1chr2_26933548_26939808 727.4400439 398.9053921 1055.974696 2.647180802 1.404456734 0.021694508 1chr12_57371907_57373209 90.33083153 49.54285383 131.1188092 2.646573604 1.404125777 0.269661123 1chr15_82450435_82451878 90.33083153 49.54285383 131.1188092 2.646573604 1.404125777 0.269661123 1chr7_72777921_72779774 112.1146408 61.50147372 162.7278079 2.645917212 1.403767922 0.222653809 1chr12_30670859_30672608 133.8984501 73.46009361 194.3368065 2.645474529 1.403526527 0.187304742 1chr3_64750615_64752176 88.73303431 48.6886667 128.7774019 2.64491535 1.40322155 0.274025263 1chr7_132569320_132570769 88.73303431 48.6886667 128.7774019 2.64491535 1.40322155 0.274025263 1chr2_203314076_203315970 110.5168436 60.64728659 160.3864006 2.644576692 1.403036814 0.22589071 1chr5_413276_416386 750.2362985 411.7181991 1088.754398 2.644416497 1.40294942 0.020966375 1chr3_32218246_32220155 154.0844621 84.56452637 223.6043979 2.644186723 1.402824058 0.161916414 1chr21_42558261_42560744 175.8682714 96.52314626 255.2133965 2.644064211 1.402757213 0.139928802 1chr7_68143754_68146232 197.6520807 108.4817662 286.8223952 2.643968709 1.402705103 0.122264572 1chr1_235182766_235189811 832.578144 456.9901173 1208.166171 2.643746823 1.402584025 0.018360253 1chr10_32116588_32120709 328.3549363 180.2334855 476.4763871 2.643661835 1.402537646 0.064313672 1chr1_7704870_7707673 261.4057113 143.5034387 379.3079838 2.643197872 1.402284431 0.08674639 1chr1_9541977_9543474 108.9190464 59.79309945 158.0449933 2.643197872 1.402284431 0.229199835 1chr5_59784993_59787344 196.0542834 107.627579 284.4809879 2.643197872 1.402284431 0.123548519 1chr5_67600676_67603267 174.2704742 95.66895913 252.8719892 2.643197872 1.402284431 0.141510206 1chr9_71612608_71615008 152.4866649 83.71033924 221.2629906 2.643197872 1.402284431 0.163896334 1chrX_83846360_83847792 87.13523708 47.83447956 126.4359946 2.643197872 1.402284431 0.278499513 1chr2_215538742_215541866 259.807914 142.6492516 376.9665765 2.642611667 1.401964436 0.087498129 1chr8_118525642_118527972 172.6726769 94.81477199 250.5305819 2.642315924 1.401802971 0.14311881 1chr2_127921996_127924777 323.5615446 177.6709241 469.4521652 2.642256563 1.401770559 0.065731461 1chr12_110046550_110047969 107.3212491 58.93891232 155.7035859 2.641779087 1.401509829 0.232583417 1chr2_174940318_174941491 85.53743986 46.98029243 124.0945873 2.641417941 1.401312591 0.283087843 1chr20_5693552_5696656 320.3659502 175.9625498 464.7693505 2.641296975 1.40124652 0.066704125 1chr3_66513198_66514926 298.5821409 164.0039299 433.1603519 2.641158368 1.40117081 0.073224534 1chr1_27283279_27284940 127.5072612 70.04334507 184.9711773 2.64081016 1.400980594 0.197523316 1chr1_57636301_57637510 63.75363059 35.02167254 92.48558865 2.64081016 1.400980594 0.353617115 1chr18_2524453_2525533 63.75363059 35.02167254 92.48558865 2.64081016 1.400980594 0.353617115 1chr7_1789036_1790861 191.2608918 105.0650176 277.4567659 2.64081016 1.400980594 0.12752558 1chr1_110704501_110712150 1337.228445 734.6009361 1939.855954 2.64069355 1.400916888 0.010589584 1chr1_197806498_197808810 167.8792853 92.25221059 243.50636 2.639572086 1.400304066 0.148114146 1chr10_77831491_77832948 83.93964264 46.12610529 121.75318 2.639572086 1.400304066 0.287794417 1chr16_21219435_21221715 167.8792853 92.25221059 243.50636 2.639572086 1.400304066 0.148114146 1chr3_87475976_87478096 125.909464 69.18915794 182.62977 2.639572086 1.400304066 0.200209933 1chr12_109536878_109540640 313.9747613 172.5458013 455.4037213 2.639320794 1.400166713 0.068718454 1chr2_208375746_208379401 313.9747613 172.5458013 455.4037213 2.639320794 1.400166713 0.068718454 1chr1_55639167_55640609 104.1256547 57.23053805 151.0207713 2.63881446 1.399889915 0.239583352 1chr1_24862504_24863963 104.1256547 57.23053805 151.0207713 2.63881446 1.399889915 0.239583352 1chr17_61200275_61205781 478.6584521 263.0896376 694.2272667 2.638748044 1.399853604 0.038861323 1chr5_70499360_70501770 166.2814881 91.39802345 241.1649527 2.638623283 1.399785391 0.14983797 1chr2_219747414_219749372 124.3116667 68.3349708 180.2883627 2.638303061 1.399610296 0.202952309 1chr5_29960067_29961495 144.4976788 79.43940356 209.555954 2.637934635 1.399408817 0.174367072 1chr7_97949742_97953310 371.337203 204.1507253 538.5236807 2.637872973 1.399375093 0.055017648 1chr9_35381602_35382867 102.5278575 56.37635091 148.679364 2.637264768 1.399042418 0.243204654 1chr18_59381097_59382543 122.7138695 67.48078367 177.9469554 2.637001909 1.398898616 0.205752026 1chr4_124285497_124287683 122.7138695 67.48078367 177.9469554 2.637001909 1.398898616 0.205752026 1chr7_50540243_50544461 653.9413717 359.6127839 948.2699595 2.636919493 1.398853525 0.02555811 1chr12_109989865_109991535 142.8998816 78.58521643 207.2145467 2.636813336 1.398795444 0.17658232 1chr12_3642005_3645040 364.9460141 200.7339767 529.1580515 2.636116018 1.398413866 0.056486971 1chr1_49255140_49257299 121.1160723 66.62659653 175.6055481 2.635667394 1.398168322 0.208610723 1chr14_57097189_57098845 121.1160723 66.62659653 175.6055481 2.635667394 1.398168322 0.208610723 1chr16_5261665_5263632 121.1160723 66.62659653 175.6055481 2.635667394 1.398168322 0.208610723 1chr22_39232918_39233869 161.4880964 88.83546205 234.1407308 2.635667394 1.398168322 0.155195395 1chr4_119848777_119850189 100.9300602 55.52216378 146.3379567 2.635667394 1.398168322 0.246910322 1chr5_43202456_43203946 80.7440482 44.41773102 117.0703654 2.635667394 1.398168322 0.297580037 1chr7_122052064_122053586 121.1160723 66.62659653 175.6055481 2.635667394 1.398168322 0.208610723 1chr7_140421816_140422837 60.55803615 33.31329827 87.80277403 2.635667394 1.398168322 0.367279847 1chr8_121154131_121155057 60.55803615 33.31329827 87.80277403 2.635667394 1.398168322 0.367279847 1chr19_3682817_3684013 240.6343474 132.3990059 348.8696888 2.634987222 1.397795966 0.097359493 1chr8_62748230_62752826 537.0333392 295.5487487 778.5179297 2.634143887 1.397334153 0.033516464 1chr7_127849367_127851386 297.9967891 164.0039299 431.9896482 2.634020102 1.397266356 0.074191644 1chr2_173536125_173539334 257.624765 141.7950644 373.4544655 2.633762092 1.397125033 0.089512302 1chr2_27520807_27523081 257.624765 141.7950644 373.4544655 2.633762092 1.397125033 0.089512302 1chr10_31363335_31364551 79.14625098 43.56354389 114.7289581 2.633600204 1.397036353 0.302668532 1chr2_14903941_14905091 79.14625098 43.56354389 114.7289581 2.633600204 1.397036353 0.302668532 1chr5_73390895_73392253 79.14625098 43.56354389 114.7289581 2.633600204 1.397036353 0.302668532 1chr7_129353793_129355598 158.292502 87.12708778 229.4579161 2.633600204 1.397036353 0.158929466 1chr7_18459128_18462057 254.4291705 140.0866901 368.7716509 2.632453166 1.396407864 0.09109622 1chr6_34882063_34883564 97.73446581 53.81378951 141.6551421 2.632320515 1.396335164 0.254585878 1chr1_22834715_22837143 173.6851223 95.66895913 251.7012856 2.630960845 1.395589778 0.143900565 1chr20_23846701_23848528 173.6851223 95.66895913 251.7012856 2.630960845 1.395589778 0.143900565 1chr22_46816929_46818508 134.9108955 74.31428075 195.5075102 2.63082019 1.395512647 0.188323645 1chr1_116423863_116424904 57.36244171 31.604924 83.11995942 2.629968654 1.395045604 0.381815478 1chr2_38237421_38243568 573.6244171 316.04924 831.1995942 2.629968654 1.395045604 0.030918042 1chr8_116728524_116729588 57.36244171 31.604924 83.11995942 2.629968654 1.395045604 0.381815478 1chr9_80118336_80120778 133.3130982 73.46009361 193.1661029 2.629537935 1.39480931 0.1908133 1chr5_74873030_74874390 75.95065654 41.85516962 110.0461435 2.629212698 1.394630858 0.313262429 1chr21_46997479_46999721 246.4401844 135.8157545 357.0646144 2.629036784 1.394534328 0.095249717 1chr1_25995960_25998337 207.6659576 114.4610761 300.870839 2.628586497 1.394287209 0.117410313 1chr11_73005455_73007290 168.8917307 93.10639772 244.6770636 2.627929655 1.393926658 0.148920232 1chr12_113172330_113174706 224.6563752 123.8571346 325.4556157 2.627669507 1.393783833 0.106942005 1chr2_7233156_7238407 542.2538245 298.9654973 785.5421517 2.627534478 1.393709695 0.033521451 1chr1_80785078_80786115 74.35285931 41.00098248 107.7047361 2.626881836 1.393351306 0.318778892 1chr11_101437202_101438418 74.35285931 41.00098248 107.7047361 2.626881836 1.393351306 0.318778892 1chr18_45372438_45373485 55.76464449 30.75073686 80.77855211 2.626881836 1.393351306 0.389437384 1chr3_41151994_41153295 74.35285931 41.00098248 107.7047361 2.626881836 1.393351306 0.318778892 1chr5_17089549_17090715 74.35285931 41.00098248 107.7047361 2.626881836 1.393351306 0.318778892 1chr1_54559099_54561597 295.81364 163.1497428 428.4775373 2.626283866 1.393022861 0.075765657 1chr8_143548272_143552621 552.8530532 304.9448072 800.7612992 2.625922069 1.392824101 0.032763576 1chr15_93671775_93673811 147.1079214 81.14777783 213.068065 2.625679602 1.392690882 0.173126557 1chr2_223964010_223967333 311.2062604 171.6916141 450.7209067 2.625177176 1.392414795 0.071034111 1chr5_15271290_15272457 72.75506209 40.14679535 105.3633288 2.624451788 1.392016095 0.324449591 1chr6_135182765_135184022 72.75506209 40.14679535 105.3633288 2.624451788 1.392016095 0.324449591 1chr7_100761003_100762778 145.5101242 80.2935907 210.7266577 2.624451788 1.392016095 0.175310147 1chr7_111505059_111506337 72.75506209 40.14679535 105.3633288 2.624451788 1.392016095 0.324449591 1chr18_21115363_21123281 1306.395523 720.933942 1891.857104 2.624175385 1.391864144 0.011363669 1chr22_46762853_46764638 126.9219094 70.04334507 183.8004736 2.624096171 1.391820595 0.201287036 1chr1_55514745_55517020 270.8342363 149.4827486 392.185724 2.623618629 1.391558024 0.085190995 1chr11_75459568_75460587 54.16684726 29.89654973 78.4371448 2.623618629 1.391558024 0.397311174 1chr12_94217562_94219266 108.3336945 59.79309945 156.8742896 2.623618629 1.391558024 0.233822265 1chr1_117514128_117516261 143.912327 79.43940356 208.3852504 2.62319757 1.39132647 0.177535594 1chr6_157314814_157316634 160.9027446 88.83546205 232.9700271 2.622489057 1.390936753 0.157894423 1chr2_170695455_170697888 196.481377 108.4817662 284.4809879 2.622385291 1.390879667 0.126212314 1chr3_9152537_9154453 106.7358973 58.93891232 154.5328823 2.621916086 1.390621513 0.237296867 1chr6_149428590_149430377 106.7358973 58.93891232 154.5328823 2.621916086 1.390621513 0.237296867 1chrX_149004910_149007023 123.7263149 68.3349708 179.117659 2.621171223 1.390211598 0.206853406 1chr5_60647387_60649524 140.7167325 77.73102929 203.7024358 2.620606438 1.389900706 0.182116418 1chr3_59662929_59665901 245.8548326 135.8157545 355.8939107 2.620416992 1.389796409 0.096627406 1chr1_114455199_114456208 52.56905004 29.04236259 76.09573749 2.620163468 1.389656822 0.405449826 1chr5_67775798_67778906 227.2666178 125.5655089 328.9677267 2.619889249 1.389505826 0.106487019 1chr22_41611623_41614978 401.9641855 222.0886551 581.8397159 2.61985339 1.389486079 0.050982391 1chr1_205416434_205423551 765.1541442 422.8226319 1107.485656 2.619267686 1.389163509 0.021661496 1chr14_79045083_79046279 69.55946765 38.43842108 100.6805142 2.619267686 1.389163509 0.33627945 1chr7_122503397_122508880 468.3280587 258.8187019 677.8374155 2.618966135 1.388997405 0.041553859 1chr8_120902472_120906471 485.3184763 268.2147604 702.4221923 2.618879704 1.388949792 0.039608319 1chr12_105367906_105369225 86.54988526 47.83447956 125.265291 2.618723818 1.388863915 0.28455504 1chr1_67821114_67822712 103.5403029 57.23053805 149.8500677 2.618358534 1.38866266 0.244486619 1chr7_46549504_46553856 431.1516291 238.3182107 623.9850475 2.618285215 1.388622261 0.046519225 1chr6_79946123_79950906 427.9560346 236.6098364 619.3022328 2.617398508 1.388133597 0.047063039 1chr11_100796150_100800524 444.9464522 246.0058949 643.8870096 2.617364148 1.388114657 0.044660087 1chr12_82191243_82192760 84.95208804 46.98029243 122.9238836 2.616498904 1.387637655 0.289278207 1chr2_137389875_137392456 186.8945937 103.3566433 270.432544 2.616498904 1.387637655 0.134578569 1chr2_148619346_148621216 135.9233409 75.16846788 196.6782138 2.616498904 1.387637655 0.189329828 1chr20_32879370_32880965 135.9233409 75.16846788 196.6782138 2.616498904 1.387637655 0.189329828 1chr6_136945814_136946859 50.97125282 28.18817546 73.75433019 2.616498904 1.387637655 0.413867319 1chr8_128377093_128379692 355.2009725 196.4630411 513.938904 2.615957186 1.387338929 0.060541325 1chr7_22208300_22210454 151.3159612 83.71033924 218.9215833 2.615227524 1.386936466 0.169784405 1chr7_529160_533498 520.3117569 287.8610645 752.7624494 2.615020029 1.386821997 0.036334995 1chr20_5616436_5617983 100.3447084 55.52216378 145.1672531 2.614582055 1.386580348 0.252014455 1chr3_126151907_126154098 233.0724548 128.9822574 337.1626523 2.614023503 1.386272113 0.104043905 1chr1_235152736_235155837 382.7906189 211.8384095 553.7428282 2.613986904 1.386251913 0.054985085 1chr8_102145306_102147850 216.0820372 119.5861989 312.5778756 2.613829007 1.386164765 0.114003395 1chr15_38498674_38502606 282.4459104 156.3162457 408.5755752 2.613775512 1.386135238 0.081889455 1chrX_153687532_153691073 282.4459104 156.3162457 408.5755752 2.613775512 1.386135238 0.081889455 1chr14_94357797_94359738 132.7277464 73.46009361 191.9953992 2.613601341 1.3860391 0.194381319 1chr5_134773315_134774913 132.7277464 73.46009361 191.9953992 2.613601341 1.3860391 0.194381319 1chr7_34345586_34349981 447.5566948 247.7142692 647.3991205 2.613491434 1.385978431 0.044626062 1chr13_53144208_53145147 49.3734556 27.33398832 71.41292288 2.612605304 1.385489188 0.422578741 1chr5_71251352_71253922 180.5034048 99.9398948 261.0669148 2.612239239 1.385287031 0.140631197 1chr15_44220230_44223462 262.2598984 145.211813 379.3079838 2.612101427 1.385210917 0.090154309 1chr7_69474560_69477672 195.8960252 108.4817662 283.3102842 2.611593582 1.384930401 0.128230322 1chr6_164553386_164555201 114.1395316 63.20984799 165.0692152 2.611447748 1.384849837 0.225028345 1chr7_158698944_158700305 114.1395316 63.20984799 165.0692152 2.611447748 1.384849837 0.225028345 1chr9_133505580_133507256 114.1395316 63.20984799 165.0692152 2.611447748 1.384849837 0.225028345 1chrX_152984176_152988118 260.6621012 144.3576258 376.9665765 2.611338157 1.384789292 0.090942314 1chr1_156153290_156155108 178.9056076 99.08570767 258.7255075 2.611128422 1.384673415 0.142208646 1chr7_56280049_56281947 178.9056076 99.08570767 258.7255075 2.611128422 1.384673415 0.142208646 1chr1_53580576_53582490 161.91519 89.68964918 234.1407308 2.6105658 1.384362523 0.158735162 1chr5_56710776_56713108 129.532152 71.75171934 187.3125846 2.6105658 1.384362523 0.19963932 1chr7_51407386_51414286 712.4268358 394.6344564 1030.219215 2.6105658 1.384362523 0.024218746 1chr1_17271580_17276965 596.689507 330.5704213 862.8085928 2.610059876 1.384082903 0.030573044 1chr3_155971853_155975233 257.4665067 142.6492516 372.2837619 2.609784193 1.383930513 0.092550386 1chrX_121496412_121498965 272.8591271 151.1911229 394.5271313 2.609459628 1.383751082 0.086086179 1chr2_233375888_233377036 80.15869637 44.41773102 115.8996617 2.60931072 1.383668752 0.304202147 1chr3_12748503_12749837 80.15869637 44.41773102 115.8996617 2.60931072 1.383668752 0.304202147 1chr1_85146107_85148295 127.9343548 70.89753221 184.9711773 2.60899317 1.383493167 0.202349342 1chr2_133377040_133380740 319.0369883 176.816737 461.2572396 2.608674085 1.383316712 0.070525237 1chr1_59679543_59682048 286.6539503 158.8788071 414.4290934 2.608460505 1.383198589 0.080978379 1chr14_23334156_23336681 238.8782919 132.3990059 345.3575779 2.608460505 1.383198589 0.101696306 1chr4_89691938_89694175 143.3269751 79.43940356 207.2145467 2.608460505 1.383198589 0.18075647 1chr1_244509964_244512374 206.4952539 114.4610761 298.5294317 2.608130571 1.383016097 0.121107512 1chr12_27378647_27380324 110.9439371 61.50147372 160.3864006 2.60784646 1.382858932 0.231628554 1chr1_59323222_59325014 174.1122159 96.52314626 251.7012856 2.607678006 1.382765738 0.14710471 1chr2_166272796_166275518 237.2804947 131.5448188 343.0161705 2.607599248 1.382722164 0.102647272 1chr2_31478104_31480841 220.2900771 122.1487603 318.4313938 2.606914659 1.382343355 0.112365075 1chr7_72586119_72587362 78.56089915 43.56354389 113.5582544 2.606726732 1.382239351 0.309444346 1chr5_115776681_115778516 93.95351954 52.10541524 135.8016238 2.606286184 1.381995508 0.268176145 1chr6_11136084_11137781 93.95351954 52.10541524 135.8016238 2.606286184 1.381995508 0.268176145 1chr7_18161942_18167527 765.4229795 424.5310061 1106.314953 2.605969733 1.381820328 0.022330909 1chr15_64747646_64749591 140.1313807 77.73102929 202.5317321 2.605545481 1.381585438 0.185446929 1chr14_80552481_80554855 155.5240011 86.27290064 224.7751015 2.605396363 1.381502868 0.166551098 1chr1_75562851_75565686 169.3188242 93.96058486 244.6770636 2.604039385 1.380751269 0.15225971 1chr1_164402405_164404131 107.7483427 59.79309945 155.7035859 2.604039385 1.380751269 0.23852774 1chr10_7053452_7054500 61.57048154 34.1674854 88.97347769 2.604039385 1.380751269 0.369047037 1chr11_115501678_115503073 92.35572232 51.2512281 133.4602165 2.604039385 1.380751269 0.27246545 1chr12_48515617_48517098 123.1409631 68.3349708 177.9469554 2.604039385 1.380751269 0.210821947 1chr12_64788857_64790320 107.7483427 59.79309945 155.7035859 2.604039385 1.380751269 0.23852774 1chr12_112617382_112618787 123.1409631 68.3349708 177.9469554 2.604039385 1.380751269 0.210821947 1chr13_53206524_53207589 61.57048154 34.1674854 88.97347769 2.604039385 1.380751269 0.369047037 1chr14_37201560_37203394 123.1409631 68.3349708 177.9469554 2.604039385 1.380751269 0.210821947 1chr4_1394139_1395870 92.35572232 51.2512281 133.4602165 2.604039385 1.380751269 0.27246545 1chr7_20992075_20993256 76.96310193 42.70935675 111.2168471 2.604039385 1.380751269 0.314829005 1chr3_14595456_14599820 475.5734347 263.9438247 687.2030448 2.603595843 1.380505516 0.041904711 1chr4_9880585_9883178 183.1136474 101.6482691 264.5790257 2.602887665 1.38011305 0.139813544 1chr5_159617509_159619936 167.721027 93.10639772 242.3356563 2.602782003 1.380054483 0.15403873 1chrX_152727770_152729768 152.3284066 84.56452637 220.0922869 2.602654994 1.379984082 0.170678548 1chr19_19531365_19532668 196.9084706 109.3359533 284.4809879 2.601897906 1.379564354 0.128905021 1chr13_91599328_91600747 90.75792509 50.39704097 131.1188092 2.601716424 1.379463723 0.276861231 1chr2_37956570_37958269 90.75792509 50.39704097 131.1188092 2.601716424 1.379463723 0.276861231 1chr3_55022069_55023576 90.75792509 50.39704097 131.1188092 2.601716424 1.379463723 0.276861231 1chr2_46108276_46114645 619.9128553 344.2374154 895.5882951 2.601658783 1.37943176 0.029595928 1chr2_71785478_71788881 226.0959141 125.5655089 326.6263194 2.601242351 1.379200817 0.109700695 1chr21_30603940_30605788 135.337989 75.16846788 195.5075102 2.600924506 1.379024524 0.19283474 1chr11_67838014_67840319 419.8087903 233.1930879 606.4244926 2.600525162 1.378802997 0.04977263 1chr13_74241508_74242512 59.97268432 33.31329827 86.63207038 2.600525162 1.378802997 0.376215519 1chr1_22332766_22334803 193.7128761 107.627579 279.7981732 2.599688442 1.378338735 0.131673745 1chr14_23739036_23741121 238.2929401 132.3990059 344.1868742 2.599618266 1.37829979 0.103181816 1chr7_68922261_68923851 192.1150789 106.7733919 277.4567659 2.598557197 1.377710815 0.13309195 1chr8_38424259_38425560 73.76750749 41.00098248 106.5340325 2.598328773 1.37758399 0.326048574 1chr9_80283871_80285988 147.535015 82.00196497 213.068065 2.598328773 1.37758399 0.177165019 1chr1_48236780_48239240 308.8648531 171.6916141 446.0380921 2.597902608 1.377347347 0.074762839 1chr1_220520414_220522016 102.954951 57.23053805 148.679364 2.597902608 1.377347347 0.249478948 1chr12_125563200_125567420 454.8020709 252.839392 656.7647498 2.597557068 1.377155445 0.044983841 1chr2_242231073_242233653 161.3298381 89.68964918 232.9700271 2.597512971 1.377130953 0.161477713 1chr21_30275802_30279804 381.0345634 211.8384095 550.2307173 2.597407706 1.377072487 0.056903057 1chr1_85288871_85291165 219.7047252 122.1487603 317.2606902 2.597330414 1.377029555 0.114067677 1chr17_9045298_9050236 439.4094505 244.2975206 634.5213803 2.597330414 1.377029555 0.047112078 1chr1_114780205_114781332 58.3748871 32.45911113 84.29066307 2.59682598 1.376749338 0.383611709 1chr16_28431749_28433482 145.9372178 81.14777783 210.7266577 2.59682598 1.376749338 0.17940995 1chr21_48024425_48025951 116.7497742 64.91822226 168.5813261 2.59682598 1.376749338 0.222980937 1chr4_188087705_188089803 175.1246613 97.3773334 252.8719892 2.59682598 1.376749338 0.14787482 1chr18_46306460_46309946 393.2315893 218.6719066 567.7912721 2.596544206 1.376592788 0.054664497 1chr2_236399143_236402508 364.0441458 202.442351 525.6459405 2.596521617 1.376580236 0.060514175 1chr11_60794675_60796863 247.2943716 137.5241287 357.0646144 2.596377942 1.376500405 0.099045965 1chr6_38655829_38658237 247.2943716 137.5241287 357.0646144 2.596377942 1.376500405 0.099045965 1chr10_119168048_119174913 721.6971027 401.4679535 1041.926252 2.595291213 1.37589643 0.024660627 1chr21_45312531_45313853 72.16971027 40.14679535 104.1926252 2.595291213 1.37589643 0.33189573 1chr7_28505606_28507006 115.151977 64.06403513 166.2399188 2.594902405 1.375680279 0.226186864 1chr3_69211011_69212492 85.96453343 47.83447956 124.0945873 2.594249764 1.375317383 0.290725844 1chr6_56258138_56260967 214.9113336 119.5861989 310.2364682 2.594249764 1.375317383 0.117518598 1chr7_7189819_7192534 171.9290669 95.66895913 248.1891746 2.594249764 1.375317383 0.151296857 1chr15_100740903_100743849 271.6884235 151.1911229 392.185724 2.593973221 1.375163586 0.088462587 1chr5_174777898_174780285 199.5187132 111.0443276 287.9930988 2.593496716 1.374898543 0.128217532 1chr3_122861146_122863848 256.2958031 142.6492516 369.9423546 2.593370456 1.374828306 0.095178606 1chr12_105037005_105039085 227.1083595 126.419696 327.7970231 2.592926842 1.374581502 0.110268757 1chr12_132259594_132260759 56.77708988 31.604924 81.94925576 2.592926842 1.374581502 0.391246702 1chr6_36274197_36275925 113.5541798 63.20984799 163.8985115 2.592926842 1.374581502 0.229463254 1chr2_75467278_75469362 184.1260928 102.5024562 265.7497294 2.59261816 1.374409742 0.140541442 1chr1_113088273_113089128 127.3490029 70.89753221 183.8004736 2.592480555 1.374333168 0.206174354 1chrX_24886750_24888267 127.3490029 70.89753221 183.8004736 2.592480555 1.374333168 0.206174354 1chr22_39722447_39724630 141.1438261 78.58521643 203.7024358 2.592121585 1.37413339 0.186408223 1chr7_22149707_22151948 141.1438261 78.58521643 203.7024358 2.592121585 1.37413339 0.186408223 1chr11_70260222_70264922 719.5139536 400.6137663 1038.414141 2.592058057 1.374098032 0.024938546 1chr6_53492960_53495201 154.9386493 86.27290064 223.6043979 2.59182659 1.373969196 0.169471688 1chr8_8538645_8541043 182.5282956 101.6482691 263.4083221 2.591370463 1.373715278 0.142106756 1chr1_71356163_71358681 210.1179419 117.0236375 303.2122463 2.591034194 1.373528055 0.121130351 1chr12_52473480_52477738 532.1922664 296.4029359 767.9815969 2.591005364 1.373512002 0.036984679 1chr1_7528988_7532446 361.8609967 201.5881639 522.1338296 2.590101619 1.373008701 0.061645399 1chr7_65603253_65609014 499.8092284 278.465006 721.1534507 2.589745336 1.372810237 0.040283281 1chr1_93433398_93434483 68.97411582 38.43842108 99.50981057 2.588811085 1.37228969 0.344098101 1chr1_116901464_116902511 55.17929266 30.75073686 79.60784846 2.588811085 1.37228969 0.399132392 1chr1_233369074_233370119 55.17929266 30.75073686 79.60784846 2.588811085 1.37228969 0.399132392 1chr12_16368295_16369178 55.17929266 30.75073686 79.60784846 2.588811085 1.37228969 0.399132392 1chr15_71694534_71695444 55.17929266 30.75073686 79.60784846 2.588811085 1.37228969 0.399132392 1chr16_14765407_14767106 137.9482316 76.87684216 199.0196211 2.588811085 1.37228969 0.191304961 1chr2_239757567_239758562 55.17929266 30.75073686 79.60784846 2.588811085 1.37228969 0.399132392 1chr20_969519_970781 68.97411582 38.43842108 99.50981057 2.588811085 1.37228969 0.344098101 1chr4_82407041_82408671 110.3585853 61.50147372 159.2156969 2.588811085 1.37228969 0.236236041 1chr5_53432338_53433412 55.17929266 30.75073686 79.60784846 2.588811085 1.37228969 0.399132392 1chr6_149184731_149187034 165.537878 92.25221059 238.8235454 2.588811085 1.37228969 0.158509033 1chr7_73685485_73686674 82.76893899 46.12610529 119.4117727 2.588811085 1.37228969 0.300575119 1chr8_119518073_119523231 551.7929266 307.5073686 796.0784846 2.588811085 1.37228969 0.035420773 1chr8_102745558_102746861 110.3585853 61.50147372 159.2156969 2.588811085 1.37228969 0.236236041 1chr6_38135081_38139189 481.2210135 268.2147604 694.2272667 2.588326107 1.372019396 0.042469842 1chr22_27972037_27974339 191.5297271 106.7733919 276.2860623 2.587592821 1.371610615 0.135229034 1chr10_14574413_14576814 177.7349039 99.08570767 256.3841002 2.5874983 1.371557915 0.146963793 1chr17_54305845_54310494 355.4698078 198.1714153 512.7682003 2.5874983 1.371557915 0.063365897 1chr12_715674_717592 136.3504344 76.02265502 196.6782138 2.58710004 1.371335842 0.193826405 1chr21_35071469_35072881 122.5556113 68.3349708 176.7762517 2.586907547 1.371228495 0.214858869 1chr3_186743023_186744637 122.5556113 68.3349708 176.7762517 2.586907547 1.371228495 0.214858869 1chr12_68027048_68028670 108.7607881 60.64728659 156.8742896 2.586666254 1.371093921 0.239736969 1chr11_119179494_119180959 94.96596493 52.95960237 136.9723275 2.586354907 1.37092026 0.269518297 1chr2_152931629_152933014 94.96596493 52.95960237 136.9723275 2.586354907 1.37092026 0.269518297 1chr7_149933146_149934781 94.96596493 52.95960237 136.9723275 2.586354907 1.37092026 0.269518297 1chr2_211413177_211414538 81.17114177 45.27191816 117.0703654 2.585937821 1.370687586 0.305695286 1chr3_38394510_38397574 229.7186021 128.1280703 331.309134 2.585765425 1.370591403 0.109772019 1chr2_193388951_193390074 67.3763186 37.58423394 97.16840326 2.585350107 1.370359663 0.350467848 1chr16_15694766_15695534 120.957814 67.48078367 174.4348444 2.584955819 1.370139623 0.217876993 1chr17_74895053_74898632 456.2416098 254.5477662 657.9354534 2.58472295 1.37000965 0.045879168 1chr15_65242971_65243885 53.58149544 29.89654973 77.26644115 2.584460142 1.369862953 0.407281552 1chr7_129926434_129928076 107.1629909 59.79309945 154.5328823 2.584460142 1.369862953 0.243317428 1chr1_167617495_167620139 200.5311586 111.8985147 289.1638025 2.584161222 1.369696081 0.128877121 1chr16_28904278_28906245 119.3600168 66.62659653 172.0934371 2.582954046 1.369021976 0.220959749 1chr19_4206588_4209740 278.5067059 155.4620586 401.5513532 2.582954046 1.369021976 0.087122302 1chr2_200839403_200840973 119.3600168 66.62659653 172.0934371 2.582954046 1.369021976 0.220959749 1chr5_71454818_71456171 79.57334455 44.41773102 114.7289581 2.582954046 1.369021976 0.310952436 1chr6_36308107_36309266 79.57334455 44.41773102 114.7289581 2.582954046 1.369021976 0.310952436 1chr14_23572082_23575342 344.2852273 192.1921054 496.3783492 2.582719765 1.368891114 0.066563999 1chr5_60274231_60275811 105.5651937 58.93891232 152.191475 2.582190085 1.368595207 0.246979925 1chr12_63886372_63888033 171.343715 95.66895913 247.0184709 2.582012736 1.368496117 0.153839021 1chr11_1080441_1083104 380.8763051 212.6925966 549.0600136 2.581472145 1.368194031 0.05849913 1chr2_30703512_30705017 91.77037049 51.2512281 132.2895129 2.581196935 1.368040217 0.278231682 1chr5_134605033_134606456 91.77037049 51.2512281 132.2895129 2.581196935 1.368040217 0.278231682 1chr10_22583223_22584730 117.7622196 65.7724094 169.7520298 2.580900279 1.3678744 0.224109032 1chr20_5956038_5958557 235.5244392 131.5448188 339.5040596 2.580900279 1.3678744 0.107220828 1chr21_27305970_27309074 287.5081374 160.5871814 414.4290934 2.580710925 1.367768549 0.083999149 1chr12_65575616_65576666 169.7459178 94.81477199 244.6770636 2.580579571 1.367695116 0.155633546 1chr2_241896606_241898491 195.7377669 109.3359533 282.1395806 2.580483108 1.367641187 0.133034566 1chr1_98105766_98106663 51.98369822 29.04236259 74.92503384 2.579853261 1.367289009 0.415707921 1chr11_1649658_1650676 51.98369822 29.04236259 74.92503384 2.579853261 1.367289009 0.415707921 1chr15_29431419_29433346 155.9510946 87.12708778 224.7751015 2.579853261 1.367289009 0.170338659 1chr2_205044835_205046082 77.97554732 43.56354389 112.3875508 2.579853261 1.367289009 0.316351875 1chr2_58670052_58671012 51.98369822 29.04236259 74.92503384 2.579853261 1.367289009 0.415707921 1chr2_220137519_220140045 272.115517 152.04531 392.185724 2.579400337 1.367035705 0.090125346 1chr9_16261245_16263981 246.1236679 137.5241287 354.7232071 2.579352513 1.367008956 0.101904342 1chr1_118187893_118189922 232.3288448 129.8364445 334.821245 2.578792466 1.366695674 0.109278187 1chr14_23848626_23850023 116.1644224 64.91822226 167.4106225 2.578792466 1.366695674 0.227326814 1chr4_187672184_187673844 116.1644224 64.91822226 167.4106225 2.578792466 1.366695674 0.227326814 1chr6_114224654_114226126 116.1644224 64.91822226 167.4106225 2.578792466 1.366695674 0.227326814 1chr13_111317573_111319089 90.17257327 50.39704097 129.9481056 2.578486813 1.366524667 0.282751823 1chr21_44474058_44475347 90.17257327 50.39704097 129.9481056 2.578486813 1.366524667 0.282751823 1chr7_69516249_69519097 180.3451465 100.7940819 259.8962111 2.578486813 1.366524667 0.146060479 1chr11_86356376_86357510 64.18072416 35.87585967 92.48558865 2.577933727 1.366215176 0.363784203 1chr2_42690724_42692365 102.3695992 57.23053805 147.5086604 2.577446681 1.365942583 0.254561589 1chr2_136360786_136363831 204.7391984 114.4610761 295.0173207 2.577446681 1.365942583 0.126857082 1chr10_45322688_45323977 114.5666252 64.06403513 165.0692152 2.576628444 1.365484512 0.23061514 1chr12_20327314_20329462 114.5666252 64.06403513 165.0692152 2.576628444 1.365484512 0.23061514 1chr13_72415516_72416984 76.3777501 42.70935675 110.0461435 2.576628444 1.365484512 0.321899197 1chr7_73118406_73120648 687.3997509 384.3842108 990.4152911 2.576628444 1.365484512 0.0273623 1chr8_110329111_110331573 190.9443753 106.7733919 275.1153586 2.576628444 1.365484512 0.137397859 1chr1_236643433_236645544 279.5191513 156.3162457 402.7220569 2.576328871 1.365316767 0.087558226 1chr12_89823049_89826825 253.5273022 141.7950644 365.25954 2.57596794 1.365114638 0.098760008 1chr2_135116594_135118122 126.7636511 70.89753221 182.62977 2.57596794 1.365114638 0.210064415 1chr11_2545067_2548984 468.8657293 262.2354505 675.4960082 2.575914153 1.365084514 0.044999674 1chr2_169868353_169869592 88.57477605 49.54285383 127.6066983 2.57568324 1.36495518 0.287386083 1chrX_136023312_136024869 88.57477605 49.54285383 127.6066983 2.57568324 1.36495518 0.287386083 1chr1_92024019_92026625 227.5354531 127.2738831 327.7970231 2.575524648 1.364866347 0.112478558 1chr17_60826221_60829167 189.346578 105.9192047 272.7739513 2.575302109 1.364741685 0.13890036 1chr2_149954345_149958749 390.890182 218.6719066 563.1084575 2.575129409 1.364644934 0.057171701 1chr14_50182639_50183634 50.38590099 28.18817546 72.58362653 2.574967175 1.364554041 0.424426309 1chr7_155475743_155478152 163.3547289 91.39802345 235.3114344 2.574579028 1.364336555 0.16313629 1chr5_78268286_78271559 276.3235569 154.6078714 398.0392423 2.574508261 1.3642969 0.089047093 1chr13_30562814_30563941 62.58292694 35.02167254 90.14418134 2.573954206 1.363986387 0.370748236 1chr4_2714543_2717308 262.5287337 146.9201872 378.1372802 2.573759858 1.36387745 0.094936769 1chr1_54589438_54592042 149.5599058 83.71033924 215.4094723 2.573272003 1.363603962 0.178979308 1chr2_192340526_192341893 74.77995288 41.85516962 107.7047361 2.573272003 1.363603962 0.327600303 1chr2_46384208_46386679 236.5368846 132.3990059 340.6747632 2.573091549 1.363502788 0.107761492 1chr2_233337831_233339025 86.97697882 48.6886667 125.265291 2.572781295 1.363328823 0.29213856 1chr8_24013075_24014741 86.97697882 48.6886667 125.265291 2.572781295 1.363328823 0.29213856 1chrX_121227588_121228936 99.17400477 55.52216378 142.8258458 2.572411377 1.363121375 0.262504233 1chr2_142192406_142193805 111.3710307 62.35566086 160.3864006 2.572122536 1.362959375 0.237412025 1chr4_188852741_188854526 111.3710307 62.35566086 160.3864006 2.572122536 1.362959375 0.237412025 1chr8_131133322_131136073 222.7420614 124.7113217 320.7728011 2.572122536 1.362959375 0.115823337 1chr4_87921130_87925416 346.3101181 193.9004797 498.7197565 2.572039829 1.362912983 0.067214459 1chr5_56789783_56792024 160.1591345 89.68964918 230.6286198 2.571407313 1.362558152 0.167093467 1chr18_44037572_44039933 221.1442642 123.8571346 318.4313938 2.570957215 1.362305602 0.116971833 1chr3_64141097_64145427 440.6907312 246.860082 634.5213803 2.570368506 1.361975209 0.049308597 1chr12_89938088_89939238 85.3791816 47.83447956 122.9238836 2.569775709 1.361642446 0.297013559 1chr13_49945590_49947009 73.18215566 41.00098248 105.3633288 2.569775709 1.361642446 0.33346143 1chr2_174289700_174291095 73.18215566 41.00098248 105.3633288 2.569775709 1.361642446 0.33346143 1chr21_30311540_30312976 85.3791816 47.83447956 122.9238836 2.569775709 1.361642446 0.297013559 1chr21_46035881_46037067 73.18215566 41.00098248 105.3633288 2.569775709 1.361642446 0.33346143 1chr6_25582914_25584946 109.7732335 61.50147372 158.0449933 2.569775709 1.361642446 0.240925099 1chr7_35604660_35605954 73.18215566 41.00098248 105.3633288 2.569775709 1.361642446 0.33346143 1chr7_51577828_51578927 60.98512972 34.1674854 87.80277403 2.569775709 1.361642446 0.377928745 1chr8_10579379_10580928 97.57620755 54.66797664 140.4844385 2.569775709 1.361642446 0.266618422 1chrY_2129603_2130985 85.3791816 47.83447956 122.9238836 2.569775709 1.361642446 0.297013559 1chr8_130956641_130958344 547.2683702 306.6531815 787.883559 2.569298499 1.361374511 0.037254191 1chr18_59487294_59489495 230.1456957 128.9822574 331.309134 2.56864115 1.361005354 0.111955486 1chr8_119639798_119641929 217.9486698 122.1487603 313.7485792 2.568577679 1.360969704 0.119321186 1chr2_29264736_29266717 205.7516438 115.3152632 296.1880244 2.568506684 1.360929828 0.127496477 1chr14_35548246_35550014 181.3575919 101.6482691 261.0669148 2.568336059 1.360833987 0.146797701 1chr8_36947239_36951985 544.0727758 304.9448072 783.2007444 2.568336059 1.360833987 0.03761178 1chr9_138922909_138925160 181.3575919 101.6482691 261.0669148 2.568336059 1.360833987 0.146797701 1chr3_187945879_187948095 169.160566 94.81477199 243.50636 2.5682323 1.360775702 0.158254182 1chr6_55590127_55592174 169.160566 94.81477199 243.50636 2.5682323 1.360775702 0.158254182 1chr11_118064593_118067804 326.124106 182.7960469 469.4521652 2.568174603 1.360743291 0.073021169 1chr3_58480466_58484435 301.7300541 169.1290527 434.3310555 2.568045221 1.360670607 0.080564676 1chr17_53117520_53119827 277.3360023 155.4620586 399.2099459 2.56789309 1.360585139 0.089488375 1chr1_116693423_116695631 132.5694882 74.31428075 190.8246956 2.567806532 1.360536509 0.202708473 1chr14_89148540_89150199 132.5694882 74.31428075 190.8246956 2.567806532 1.360536509 0.202708473 1chr6_73839192_73840611 132.5694882 74.31428075 190.8246956 2.567806532 1.360536509 0.202708473 1chr1_52562642_52564453 120.3724622 67.48078367 173.2641408 2.567607122 1.360424468 0.222059028 1chr12_88931743_88933432 108.1754363 60.64728659 155.7035859 2.567362774 1.360287166 0.24451776 1chr7_52395513_52396778 108.1754363 60.64728659 155.7035859 2.567362774 1.360287166 0.24451776 1chr20_24759080_24762364 263.5411791 147.7743744 379.3079838 2.56680487 1.359973626 0.09540853 1chr8_19916010_19917377 83.78138438 46.98029243 120.5824763 2.56666083 1.359892664 0.302015598 1chr3_182477809_182479683 155.3657428 87.12708778 223.6043979 2.566416525 1.359755337 0.173305581 1chr10_114901586_114902800 71.58435844 40.14679535 103.0219215 2.566130637 1.359594617 0.33948917 1chr5_31581749_31582899 71.58435844 40.14679535 103.0219215 2.566130637 1.359594617 0.33948917 1chr13_63203447_63205864 130.9716909 73.46009361 188.4832883 2.565791561 1.359403974 0.205450399 1chr8_23422935_23425583 249.7463559 140.0866901 359.4060217 2.565597212 1.359294691 0.101988396 1chr2_11376313_11377328 59.3873325 33.31329827 85.46136672 2.56538293 1.35917419 0.385335954 1chr7_131790784_131791926 59.3873325 33.31329827 85.46136672 2.56538293 1.35917419 0.385335954 1chrX_55040591_55041731 59.3873325 33.31329827 85.46136672 2.56538293 1.35917419 0.385335954 1chrX_149394060_149395133 59.3873325 33.31329827 85.46136672 2.56538293 1.35917419 0.385335954 1chr19_17206823_17209745 213.1552781 119.5861989 306.7243573 2.564880898 1.358891835 0.122981427 1chr13_98890813_98892638 153.7679456 86.27290064 221.2629906 2.564687044 1.358782792 0.175453642 1chr5_1563018_1569716 1453.898072 815.748714 2092.047429 2.564573371 1.358718847 0.012037341 1chr8_124761461_124762769 94.3806131 52.95960237 135.8016238 2.56424931 1.358536535 0.275148729 1chr1_116827468_116834366 730.6508529 410.0098248 1051.291881 2.564065097 1.35843289 0.026077481 1chr21_37588748_37591042 129.3738937 72.60590648 186.1418809 2.563729178 1.35824387 0.208248641 1chr2_152772723_152773951 82.18358716 46.12610529 118.241069 2.563430584 1.358075831 0.307149428 1chr5_17814643_17816069 82.18358716 46.12610529 118.241069 2.563430584 1.358075831 0.307149428 1chr12_47067502_47070391 174.966403 98.23152053 251.7012856 2.562327084 1.357454649 0.153612846 1chr2_219766733_219767831 69.98656122 39.29260821 100.6805142 2.562327084 1.357454649 0.345690505 1chr9_71263134_71266583 325.5387542 182.7960469 468.2814615 2.561770177 1.357141054 0.073927205 1chr8_10531554_10533664 616.0842278 345.9457897 886.2226659 2.561738551 1.357123243 0.032495736 1chr3_12858660_12860602 127.7760965 71.75171934 183.8004736 2.561617691 1.357055177 0.21110477 1chr4_94692832_94694299 92.78281588 52.10541524 133.4602165 2.561350215 1.356904527 0.279571773 1chr6_40866800_40868323 92.78281588 52.10541524 133.4602165 2.561350215 1.356904527 0.279571773 1chr12_94821268_94822257 57.78953527 32.45911113 83.11995942 2.560758952 1.356571457 0.392980794 1chr7_102022863_102024991 196.1648605 110.1901404 282.1395806 2.560479363 1.356413931 0.135819829 1chr4_8268311_8269917 138.3753252 77.73102929 199.0196211 2.560362611 1.356348146 0.195772786 1chr11_111436514_111437794 80.58578994 45.27191816 115.8996617 2.560078442 1.356188016 0.312420046 1chr7_151355592_151357049 80.58578994 45.27191816 115.8996617 2.560078442 1.356188016 0.312420046 1chr19_4051123_4055344 574.6997583 322.8827371 826.5167796 2.559804798 1.3560338 0.035665196 1chr1_110489011_110492159 229.5603439 128.9822574 330.1384304 2.55956468 1.355898464 0.113603929 1chr19_16795650_16797280 148.9745539 83.71033924 214.2387686 2.559286829 1.355741845 0.18214364 1chr20_61327287_61331328 469.7199165 263.9438247 675.4960082 2.559241569 1.355716331 0.046333856 1chr1_113147589_113149703 159.5737827 89.68964918 229.4579161 2.558354484 1.355216177 0.169967826 1chr13_34419786_34421344 113.9812733 64.06403513 163.8985115 2.558354484 1.355216177 0.23512073 1chr15_90914753_90916406 113.9812733 64.06403513 163.8985115 2.558354484 1.355216177 0.23512073 1chr17_19300581_19302533 182.3700373 102.5024562 262.2376184 2.558354484 1.355216177 0.147529064 1chr2_16273818_16275313 91.18501866 51.2512281 131.1188092 2.558354484 1.355216177 0.284104967 1chr2_153422388_153423506 68.388764 38.43842108 98.33910692 2.558354484 1.355216177 0.352072829 1chr2_160661863_160663102 91.18501866 51.2512281 131.1188092 2.558354484 1.355216177 0.284104967 1chr20_60687725_60688901 68.388764 38.43842108 98.33910692 2.558354484 1.355216177 0.352072829 1chr3_41488373_41489735 91.18501866 51.2512281 131.1188092 2.558354484 1.355216177 0.284104967 1chr6_47756562_47758083 91.18501866 51.2512281 131.1188092 2.558354484 1.355216177 0.284104967 1chr7_18447794_18449097 68.388764 38.43842108 98.33910692 2.558354484 1.355216177 0.352072829 1chrX_13160732_13161886 68.388764 38.43842108 98.33910692 2.558354484 1.355216177 0.352072829 1chr1_11038985_11041643 238.5617754 134.1073802 343.0161705 2.557772511 1.354887956 0.10883501 1chr3_31773737_31777646 340.3460228 191.3379183 489.3541273 2.557538682 1.35475606 0.070350151 1chr12_52837626_52839675 226.3647494 127.2738831 325.4556157 2.557128044 1.354524403 0.115836571 1chrX_134084458_134085978 101.7842474 57.23053805 146.3379567 2.556990755 1.354446945 0.259735803 1chr1_3492962_3495755 180.7722401 101.6482691 259.8962111 2.556818857 1.354349953 0.149196334 1chr7_75020702_75023551 180.7722401 101.6482691 259.8962111 2.556818857 1.354349953 0.149196334 1chr2_38092628_38093938 78.98799272 44.41773102 113.5582544 2.556597372 1.354224974 0.317832714 1chrX_71881890_71883014 78.98799272 44.41773102 113.5582544 2.556597372 1.354224974 0.317832714 1chr20_18706969_18711364 619.7069158 348.5083511 890.9054805 2.556338973 1.354079152 0.032630266 1chr6_38687515_38691070 382.7429376 215.255158 550.2307173 2.556179012 1.353988874 0.060697418 1chr10_80604297_80606152 112.3834761 63.20984799 161.5571042 2.55588503 1.353822942 0.23856808 1chr13_95913916_95914924 56.19173805 31.604924 80.77855211 2.55588503 1.353822942 0.400874968 1chr3_158998092_158998985 56.19173805 31.604924 80.77855211 2.55588503 1.353822942 0.400874968 1chr5_72818063_72819686 112.3834761 63.20984799 161.5571042 2.55588503 1.353822942 0.23856808 1chr8_57759352_57760438 56.19173805 31.604924 80.77855211 2.55588503 1.353822942 0.400874968 1chr12_107931461_107933989 145.7789595 82.00196497 209.555954 2.555499177 1.353605127 0.186819033 1chr7_98007122_98008537 235.3661809 132.3990059 338.3333559 2.555407071 1.353553128 0.110919735 1chr8_70304570_70307002 235.3661809 132.3990059 338.3333559 2.555407071 1.353553128 0.110919735 1chr8_37486950_37488652 122.9827048 69.18915794 176.7762517 2.554970417 1.353306587 0.220037062 1chr14_25678810_25679965 66.79096678 37.58423394 95.99769961 2.554201311 1.352872236 0.358643992 1chr18_48724569_48726126 100.1864502 56.37635091 143.9965494 2.554201311 1.352872236 0.263766027 1chr2_32269477_32270663 66.79096678 37.58423394 95.99769961 2.554201311 1.352872236 0.358643992 1chr5_90125283_90126436 66.79096678 37.58423394 95.99769961 2.554201311 1.352872236 0.358643992 1chr19_1901052_1904731 344.5540626 193.9004797 495.2076455 2.553926872 1.352717216 0.06966807 1chr12_99973324_99974509 77.3901955 43.56354389 111.2168471 2.55297979 1.352182117 0.323392976 1chr2_74928701_74930048 77.3901955 43.56354389 111.2168471 2.55297979 1.352182117 0.323392976 1chr3_48196071_48197302 77.3901955 43.56354389 111.2168471 2.55297979 1.352182117 0.323392976 1chr7_13824258_13825613 77.3901955 43.56354389 111.2168471 2.55297979 1.352182117 0.323392976 1chr7_70638437_70639673 77.3901955 43.56354389 111.2168471 2.55297979 1.352182117 0.323392976 1chr7_135420884_135422139 77.3901955 43.56354389 111.2168471 2.55297979 1.352182117 0.323392976 1chr8_103067983_103069257 77.3901955 43.56354389 111.2168471 2.55297979 1.352182117 0.323392976 1chr9_95206498_95207915 154.780391 87.12708778 222.4336942 2.55297979 1.352182117 0.176319576 1chr3_14885984_14892606 1092.464168 615.0147372 1569.9136 2.552643871 1.351992276 0.016728753 1chr12_10879396_10880161 43.99471211 24.77142692 63.2179973 2.552053118 1.351658357 0.462560784 1chr2_2067063_2068423 87.98942422 49.54285383 126.4359946 2.552053118 1.351658357 0.293517529 1chr7_36186522_36187718 87.98942422 49.54285383 126.4359946 2.552053118 1.351658357 0.293517529 1chr1_110443608_110445078 142.5833651 80.2935907 204.8731394 2.55155035 1.351374111 0.19167724 1chr14_33946353_33949194 285.1667301 160.5871814 409.7462788 2.55155035 1.351374111 0.088544091 1chr8_8431441_8436555 612.7303751 345.0916026 880.3691476 2.551117272 1.351129219 0.033475069 1chr6_138997982_138999564 109.1878817 61.50147372 156.8742896 2.550740334 1.350916039 0.245696855 1chrX_23972155_23973113 54.59394083 30.75073686 78.4371448 2.550740334 1.350916039 0.409031032 1chr12_27443913_27447718 359.3613312 202.442351 516.2803113 2.550258425 1.350643447 0.06644496 1chr8_95970124_95975803 761.1195772 428.8019418 1093.437213 2.549981952 1.350487036 0.025621 1chr10_74240348_74241448 65.19316955 36.73004681 93.6562923 2.549854967 1.350415191 0.365412329 1chr12_109981843_109984189 325.9658478 183.650234 468.2814615 2.549854967 1.350415191 0.075200699 1chr11_125940589_125944536 412.3574748 232.3389007 592.3760488 2.549620606 1.350282584 0.05579545 1chr6_159178370_159182225 347.1643052 195.6088539 498.7197565 2.549576599 1.350257683 0.069495268 1chrX_6986175_6988659 281.9711357 158.8788071 405.0634642 2.549512245 1.350221267 0.090040422 1chr16_25100087_25101385 75.79239828 42.70935675 108.8754398 2.549217504 1.350054472 0.32910668 1chr18_20508562_20510539 151.5847966 85.41871351 217.7508796 2.549217504 1.350054472 0.180754311 1chr15_43224291_43228526 313.7688218 176.816737 450.7209067 2.549085084 1.349979529 0.07896669 1chr1_120284418_120286922 562.3444741 316.9034271 807.7855211 2.548995852 1.349929025 0.037501968 1chr5_16833505_16834530 162.1840253 91.39802345 232.9700271 2.548961327 1.349909485 0.168752104 1chr1_51294648_51295906 86.391627 48.6886667 124.0945873 2.54873661 1.349782291 0.298405243 1chr1_117525086_117526465 86.391627 48.6886667 124.0945873 2.54873661 1.349782291 0.298405243 1chr2_231235026_231236327 86.391627 48.6886667 124.0945873 2.54873661 1.349782291 0.298405243 1chr5_171839517_171843183 442.5573637 249.4226434 635.692084 2.548654265 1.349735679 0.051040126 1chr12_65130427_65132438 183.3824827 103.3566433 263.4083221 2.548537893 1.349669804 0.148254637 1chr8_102052368_102055880 398.5626516 224.6512165 572.4740867 2.548279487 1.349523516 0.058421301 1chr2_174094235_174096757 204.5809402 115.3152632 293.8466171 2.548202284 1.349479807 0.13147248 1chr1_98895026_98896555 107.5900844 60.64728659 154.5328823 2.548059295 1.34939885 0.249383154 1chr21_35273722_35275195 107.5900844 60.64728659 154.5328823 2.548059295 1.34939885 0.249383154 1chr1_115865359_115867040 128.7885419 72.60590648 184.9711773 2.547605095 1.349141662 0.212130643 1chr22_50935810_50937720 128.7885419 72.60590648 184.9711773 2.547605095 1.349141662 0.212130643 1chr19_13304377_13307816 278.7755412 157.1704329 400.3806496 2.547429833 1.349042409 0.091575834 1chr20_24599275_24600987 149.9869993 84.56452637 215.4094723 2.547279356 1.348957186 0.183034786 1chr10_126039196_126041163 171.1854568 96.52314626 245.8477673 2.547034331 1.348818406 0.15982178 1chr12_65028764_65031911 684.7418271 386.092585 983.3910692 2.547034331 1.348818406 0.029380042 1chrX_69261644_69264129 181.7846855 102.5024562 261.0669148 2.546933258 1.348761155 0.149928543 1chr3_194838261_194840212 202.9831429 114.4610761 291.5052098 2.546762792 1.348664592 0.132859494 1chr1_163566112_163567407 84.79382978 47.83447956 121.75318 2.545301655 1.347836647 0.303419821 1chr13_105042214_105043256 63.59537233 35.87585967 91.31488499 2.545301655 1.347836647 0.372386707 1chr14_76662077_76663713 74.19460105 41.85516962 106.5340325 2.545301655 1.347836647 0.334980002 1chr18_55637535_55639032 105.9922872 59.79309945 152.191475 2.545301655 1.347836647 0.253154145 1chr2_202524789_202525958 74.19460105 41.85516962 106.5340325 2.545301655 1.347836647 0.334980002 1chr2_232781997_232783133 63.59537233 35.87585967 91.31488499 2.545301655 1.347836647 0.372386707 1chr3_9069041_9071180 222.5838032 125.5655089 319.6020975 2.545301655 1.347836647 0.11989454 1chr3_140697692_140700216 222.5838032 125.5655089 319.6020975 2.545301655 1.347836647 0.11989454 1chr3_169640757_169642010 74.19460105 41.85516962 106.5340325 2.545301655 1.347836647 0.334980002 1chr4_144228308_144229309 52.99614361 29.89654973 76.09573749 2.545301655 1.347836647 0.417462477 1chr4_34092456_34093338 52.99614361 29.89654973 76.09573749 2.545301655 1.347836647 0.417462477 1chr4_188105064_188106703 105.9922872 59.79309945 152.191475 2.545301655 1.347836647 0.253154145 1chr5_148366385_148367720 84.79382978 47.83447956 121.75318 2.545301655 1.347836647 0.303419821 1chr7_21792360_21793609 74.19460105 41.85516962 106.5340325 2.545301655 1.347836647 0.334980002 1chr7_104801530_104802558 63.59537233 35.87585967 91.31488499 2.545301655 1.347836647 0.372386707 1chr7_129444176_129446416 222.5838032 125.5655089 319.6020975 2.545301655 1.347836647 0.11989454 1chr8_66579425_66581454 148.3892021 83.71033924 213.068065 2.545301655 1.347836647 0.185358759 1chr9_132624629_132626258 116.5915159 65.7724094 167.4106225 2.545301655 1.347836647 0.232863209 1chrX_67484238_67485485 74.19460105 41.85516962 106.5340325 2.545301655 1.347836647 0.334980002 1chr7_2709689_2711654 199.7875485 112.7527018 286.8223952 2.543818379 1.34699567 0.135700479 1chr1_111962795_111967237 535.7672732 302.3822458 769.1523005 2.543642397 1.346895861 0.040330962 1chr3_83735730_83738115 157.3906336 88.83546205 225.9458052 2.543419035 1.34676917 0.175012166 1chr6_38662821_38667805 429.7749859 242.5891464 616.9608255 2.543233425 1.346663883 0.053525597 1chr1_243374921_243377678 282.9835811 159.7329943 406.2341679 2.543207618 1.346649243 0.090472391 1chr16_28704142_28705868 136.1921762 76.87684216 195.5075102 2.543126183 1.346603047 0.201981514 1chr16_30691006_30692587 136.1921762 76.87684216 195.5075102 2.543126183 1.346603047 0.201981514 1chr6_3052122_3053659 114.9937187 64.91822226 165.0692152 2.542725439 1.346375689 0.23624647 1chr2_42740388_42741705 104.39449 58.93891232 149.8500677 2.542464083 1.346227394 0.257012529 1chr3_24278675_24281133 198.1897513 111.8985147 284.4809879 2.542312457 1.346141353 0.137155397 1chr4_149080380_149081881 93.79526128 52.95960237 134.6309202 2.542143712 1.346045591 0.280882448 1chr6_148716499_148717771 93.79526128 52.95960237 134.6309202 2.542143712 1.346045591 0.280882448 1chr17_72399317_72401503 176.9912938 99.9398948 254.0426929 2.541954776 1.345938363 0.155124262 1chr10_7173911_7175490 83.19603256 46.98029243 119.4117727 2.541741792 1.345817479 0.308565978 1chr4_79104115_79107784 322.1849015 181.9418598 462.4279432 2.541624802 1.345751073 0.077296992 1chrX_106693604_106698115 300.9864441 169.9832399 431.9896482 2.541366129 1.345604236 0.084123348 1chr8_118072196_118073395 72.59680383 41.00098248 104.1926252 2.541222646 1.345522781 0.341019465 1chr2_27127867_27129560 113.3959215 64.06403513 162.7278079 2.540080523 1.344874233 0.239704691 1chr4_74388769_74390386 113.3959215 64.06403513 162.7278079 2.540080523 1.344874233 0.239704691 1chr2_128157710_128161890 513.9834639 290.4236259 737.5433019 2.539543054 1.344568933 0.042874868 1chr5_71827342_71828334 51.39834639 29.04236259 73.75433019 2.539543054 1.344568933 0.42618383 1chr1_10437419_10439799 143.5958104 81.14777783 206.0438431 2.539118736 1.344327861 0.192602986 1chr16_3145426_3147049 132.9965817 75.16846788 190.8246956 2.538626913 1.344048386 0.207449016 1chr1_74808403_74809577 81.59823533 46.12610529 117.0703654 2.538050083 1.343720538 0.313848669 1chr12_8897366_8898759 81.59823533 46.12610529 117.0703654 2.538050083 1.343720538 0.313848669 1chr8_28046105_28048997 285.5938237 161.4413685 409.7462788 2.538050083 1.343720538 0.090143448 1chrX_22884515_22886041 111.7981243 63.20984799 160.3864006 2.537364124 1.343330568 0.243240159 1chr18_9220224_9223086 253.7961375 143.5034387 364.0888363 2.537143637 1.343205198 0.103988132 1chr4_11601642_11608438 1034.78521 585.1181875 1484.452233 2.537012632 1.343130703 0.018498934 1chr1_71052485_71055263 212.9970198 120.440386 305.5536536 2.536970062 1.343106495 0.127366516 1chr7_79649092_79650294 70.99900661 40.14679535 101.8512179 2.536970062 1.343106495 0.347231973 1chr9_71407220_71410902 243.1969088 137.5241287 348.8696888 2.53678894 1.343003493 0.109392927 1chr2_228166028_228169146 232.5976801 131.5448188 333.6505413 2.536401999 1.342783419 0.1152702 1chr12_8886038_8889848 342.7980071 193.9004797 491.6955346 2.535813916 1.342448881 0.072207127 1chr1_17309944_17312054 150.9994447 85.41871351 216.5801759 2.535512033 1.342277121 0.18391616 1chr1_56298613_56300399 150.9994447 85.41871351 216.5801759 2.535512033 1.342277121 0.18391616 1chr1_90418265_90421604 211.3992226 119.5861989 303.2122463 2.535512033 1.342277121 0.12867992 1chr1_107694550_107696915 150.9994447 85.41871351 216.5801759 2.535512033 1.342277121 0.18391616 1chr12_104922474_104923659 60.39977789 34.1674854 86.63207038 2.535512033 1.342277121 0.386991989 1chr2_215642945_215644076 60.39977789 34.1674854 86.63207038 2.535512033 1.342277121 0.386991989 1chr20_39991720_39992776 60.39977789 34.1674854 86.63207038 2.535512033 1.342277121 0.386991989 1chr7_27614349_27616367 150.9994447 85.41871351 216.5801759 2.535512033 1.342277121 0.18391616 1chrX_2400091_2401498 90.59966684 51.2512281 129.9481056 2.535512033 1.342277121 0.290086793 1chr13_45752477_45754191 110.2003271 62.35566086 158.0449933 2.534573302 1.341742889 0.246855251 1chr2_5840459_5844137 300.4010922 169.9832399 430.8189446 2.534478957 1.341689186 0.085204387 1chr16_21391117_21392424 80.00043811 45.27191816 114.7289581 2.534219064 1.34154124 0.319273108 1chr6_41437307_41441837 449.8027397 254.5477662 645.0577132 2.534132288 1.341491839 0.051355696 1chr1_108751034_108752694 129.8009873 73.46009361 186.1418809 2.533918374 1.341370051 0.213142328 1chr2_102671654_102674906 229.4020856 129.8364445 328.9677267 2.533708682 1.341250657 0.11753084 1chr12_3356435_3360486 378.8037331 214.4009709 543.2064953 2.533600912 1.341189292 0.063925469 1chr5_72602825_72604192 99.60109834 56.37635091 142.8258458 2.533435447 1.341095069 0.269140795 1chr6_89946779_89948163 99.60109834 56.37635091 142.8258458 2.533435447 1.341095069 0.269140795 1chr22_35736407_35738947 318.4039552 180.2334855 456.574425 2.533238614 1.340982975 0.079470485 1chr3_42577102_42578884 169.0023077 95.66895913 242.3356563 2.533064628 1.340883886 0.164404892 1chr8_19615155_19616746 119.2017586 67.48078367 170.9227334 2.532909729 1.340795661 0.230639481 1chr4_171034565_171035915 69.40120939 39.29260821 99.50981057 2.532532583 1.340580831 0.353624837 1chr2_220363887_220367812 436.0079166 246.860082 625.1557511 2.532429488 1.3405221 0.05360021 1chr2_45406632_45407937 89.00186961 50.39704097 127.6066983 2.532027591 1.340293126 0.294863869 1chr13_111142147_111146627 503.8113287 285.2985031 722.3241544 2.531818942 1.340174237 0.044655853 1chr1_56098407_56101515 256.4063801 145.211813 367.6009473 2.531481012 1.339981663 0.10353842 1chr1_46736607_46739991 256.4063801 145.211813 367.6009473 2.531481012 1.339981663 0.10353842 1chr2_25872961_25875024 128.2031901 72.60590648 183.8004736 2.531481012 1.339981663 0.216077608 1chr14_105037642_105039338 147.8038503 83.71033924 211.8973613 2.531316481 1.339887893 0.188625335 1chr7_68901281_68903504 167.4045105 94.81477199 239.994249 2.531190488 1.339816084 0.166360807 1chr3_72786871_72790331 324.2097923 183.650234 464.7693505 2.530731055 1.339554198 0.078021333 1chr1_120659505_120661071 137.2046216 77.73102929 196.6782138 2.530240698 1.339274633 0.202942869 1chr14_77156841_77157879 196.0066022 111.0443276 280.9688769 2.530240698 1.339274633 0.140809009 1chr14_95837002_95838244 78.40264089 44.41773102 112.3875508 2.530240698 1.339274633 0.32484479 1chr3_39236511_39238842 176.405942 99.9398948 252.8719892 2.530240698 1.339274633 0.157669293 1chr5_105207886_105209036 58.80198067 33.31329827 84.29066307 2.530240698 1.339274633 0.394643724 1chr6_78197317_78198371 58.80198067 33.31329827 84.29066307 2.530240698 1.339274633 0.394643724 1chr6_164518580_164520634 137.2046216 77.73102929 196.6782138 2.530240698 1.339274633 0.202942869 1chr7_11517834_11519143 78.40264089 44.41773102 112.3875508 2.530240698 1.339274633 0.32484479 1chr8_9483917_9486051 215.6072625 122.1487603 309.0657646 2.530240698 1.339274633 0.126677178 1chr15_61801841_61805758 322.6119951 182.7960469 462.4279432 2.52974805 1.338993707 0.07862036 1chr20_23416093_23418259 312.0127663 176.816737 447.2087957 2.529222083 1.338693721 0.081988111 1chr14_36416818_36423863 866.6370896 491.1576027 1242.116577 2.528957243 1.338542646 0.023084555 1chr12_117464975_117467148 233.6101255 132.3990059 334.821245 2.528880354 1.338498782 0.115819895 1chr5_173286124_173288098 340.6148581 193.0462925 488.1834236 2.528841229 1.338476461 0.073610968 1chr3_125604021_125605627 107.0047326 60.64728659 153.3621786 2.528755815 1.338427732 0.254334309 1chr1_62196420_62198511 174.8081448 99.08570767 250.5305819 2.528422997 1.338237842 0.159499474 1chr20_60555695_60556945 87.40407239 49.54285383 125.265291 2.528422997 1.338237842 0.299763137 1chr8_111647124_111648482 87.40407239 49.54285383 125.265291 2.528422997 1.338237842 0.299763137 1chrX_2958570_2959891 87.40407239 49.54285383 125.265291 2.528422997 1.338237842 0.299763137 1chr12_32570960_32574459 504.8237741 286.1526902 723.494858 2.528352459 1.338197593 0.044847525 1chr5_141071473_141077552 795.638083 451.0108073 1140.265359 2.528243981 1.338135693 0.025566885 1chr8_19142758_19146400 358.6177211 203.2965381 513.938904 2.528025852 1.338011217 0.069124643 1chr3_51661158_51662392 67.80341217 38.43842108 97.16840326 2.527897883 1.337938185 0.360205807 1chr3_60975009_60976168 67.80341217 38.43842108 97.16840326 2.527897883 1.337938185 0.360205807 1chr8_123658934_123660705 164.2089161 93.10639772 235.3114344 2.527339046 1.337619217 0.170378801 1chr4_4535112_4539483 337.4192636 191.3379183 483.500609 2.526946114 1.3373949 0.074705617 1chr1_2022580_2025309 201.8124393 114.4610761 289.1638025 2.526306866 1.337029892 0.137006293 1chr8_21524311_21526318 201.8124393 114.4610761 289.1638025 2.526306866 1.337029892 0.137006293 1chr1_116033665_116035905 153.6096873 87.12708778 220.0922869 2.526106318 1.33691536 0.182491267 1chr2_180859450_180860929 76.80484367 43.56354389 110.0461435 2.526106318 1.33691536 0.330569504 1chr12_1355450_1357577 182.2117791 103.3566433 261.0669148 2.525884223 1.336788513 0.15309085 1chr12_49333513_49338264 468.2326963 265.652199 670.8131936 2.525155809 1.336372409 0.049635932 1chr1_116371698_116373199 85.80627517 48.6886667 122.9238836 2.524691925 1.336107354 0.304789046 1chr1_118384551_118386194 143.0104586 81.14777783 204.8731394 2.524691925 1.336107354 0.196035816 1chr14_78736470_78739012 143.0104586 81.14777783 204.8731394 2.524691925 1.336107354 0.196035816 1chr17_54483031_54487193 629.2460179 357.0502225 901.4418134 2.524691925 1.336107354 0.034277213 1chr6_26852126_26855972 257.4188255 146.0660001 368.7716509 2.524691925 1.336107354 0.104026987 1chr21_27257165_27261646 352.2265322 199.8797896 504.5732748 2.52438366 1.33593119 0.071120147 1chr8_59600800_59603107 180.6139818 102.5024562 258.7255075 2.524090808 1.335763814 0.154835609 1chr2_213160864_213162204 123.4097984 70.04334507 176.7762517 2.523812241 1.335604585 0.225258279 1chrX_153264683_153266444 123.4097984 70.04334507 176.7762517 2.523812241 1.335604585 0.225258279 1chr15_58715865_58719708 313.0252117 177.6709241 448.3794994 2.52365153 1.335512714 0.08236811 1chr1_64165643_64170922 492.0413964 279.3191932 704.7635996 2.523147771 1.335224702 0.0468005 1chr7_6498505_6502676 624.4526263 354.487661 894.4175915 2.523127572 1.335213152 0.034720941 1chr10_44393720_44395379 198.6168448 112.7527018 284.4809879 2.523052514 1.335170235 0.139962514 1chr15_76431959_76432989 66.20561495 37.58423394 94.82699595 2.523052514 1.335170235 0.36698311 1chr8_101313860_101316885 595.8505345 338.2581055 853.4429636 2.523052514 1.335170235 0.036808619 1chr11_61219799_61221918 141.4126614 80.2935907 202.5317321 2.522389774 1.334791226 0.198603299 1chr22_30146010_30147773 141.4126614 80.2935907 202.5317321 2.522389774 1.334791226 0.198603299 1chr7_2922719_2924820 320.428846 181.9418598 458.9158323 2.522321322 1.334752074 0.080208363 1chr15_93701115_937023
[truncated: 10,072,752 more chars]
