# Supplementary material for: Transcriptome-wide identification of mRNAs and lincRNAs associated with trastuzumab-resistance in HER2-positive breast cancer
Source: Oncotarget. 2016 Jul 16;7(33):53230–44. doi: 10.18632/oncotarget.10637 (PMC5288181; doi:10.18632/oncotarget.10637)
Supplement: Supplementary file 10 [file oncotarget-07-53230-s010.docx]

id baseMean BT-474-J BT-474-R-J foldChange log2FoldChange pval padj

chr18_14744860_14753329 1525.960431 2994.958129 56.96273292 0.019019542 -5.716373659 1.09E-12 4.67E-08

chr19_52190533_52196315 1152.199069 2221.452404 82.94573389 0.037338515 -4.743191639 1.26E-08 0.000270389

chr1_152711191_152719633 1056.641168 2037.332025 75.95031055 0.037279299 -4.745481442 3.22E-08 0.000345865

chr15_24512506_24515542 1647.949993 3101.027478 194.8725073 0.062841271 -3.992143835 2.66E-08 0.000345865

chr15_24350458_24356052 1112.666687 2132.394177 92.93919581 0.043584435 -4.520043176 5.86E-08 0.000418818

chrX_138281319_138287985 689.9347453 1354.885836 24.98365479 0.018439675 -5.761042943 5.80E-08 0.000418818

chr15_24313333_24314858 1084.64706 2074.356232 94.93788819 0.045767398 -4.449535902 1.08E-07 0.000665001

chr19_57701876_57704384 586.37161 1154.754989 17.98823145 0.015577531 -6.004389617 1.77E-07 0.0009511

chr12_126465081_126469314 577.865395 1136.743212 18.98757764 0.016703489 -5.903706716 2.85E-07 0.001127101

chr19_29878086_29883231 521.3323548 1029.673209 12.99150049 0.01261711 -6.308474658 2.89E-07 0.001127101

chr19_52306326_52313685 1000.096355 1912.250245 87.94246485 0.045988994 -4.442567554 2.48E-07 0.001127101

chr19_32061475_32066054 558.8529645 1098.718351 18.98757764 0.01728157 -5.854621935 4.80E-07 0.001716984

chr8_8956262_8964055 4927.672081 1369.895649 8485.448512 6.194229842 2.630924916 7.52E-07 0.002481062

chr12_131701624_131708412 753.4560146 1450.948643 55.96338672 0.038570205 -4.696369372 1.23E-06 0.003776061

chr12_131717138_131722824 587.8647431 1145.749101 29.98038574 0.026166624 -5.256128397 1.82E-06 0.005032485

chr15_20783558_20788759 1041.597999 1956.279032 126.9169663 0.06487672 -3.946155315 1.88E-06 0.005032485

chr19_29891339_29897590 587.363762 1143.747792 30.97973194 0.027086157 -5.206300487 2.15E-06 0.005424834

chr20_58871355_58876014 525.3284315 1027.671901 22.9849624 0.022366051 -5.48254562 2.72E-06 0.006474214

chr8_11319873_11325324 2964.068652 776.5076873 5151.629617 6.634357523 2.729956761 3.08E-06 0.006952636

chr11_47349344_47357720 1470.211564 265.1733726 2675.249755 10.08868171 3.334665765 3.53E-06 0.007210049

chr19_5137387_5145598 1251.810601 198.1295388 2305.491664 11.63628441 3.540558559 3.52E-06 0.007210049

chr19_57348334_57352301 441.2793619 868.5678771 13.99084668 0.016107949 -5.95608342 4.20E-06 0.008197409

chr4_158742587_158747781 696.5822288 58.0379457 1335.126512 23.00437232 4.523836188 4.79E-06 0.008945165

chr15_24248267_24250697 1210.664091 2226.455676 194.8725073 0.087525887 -3.514146417 5.13E-06 0.00917561

chr15_23451149_23456136 1118.120579 2067.351652 168.8895064 0.081693652 -3.613632212 5.50E-06 0.009436573

chr20_60576150_60582119 677.09367 56.03663723 1298.150703 23.16610644 4.533943684 5.88E-06 0.009709639

chr7_41097531_41101475 478.7986636 935.6117109 21.98561621 0.023498654 -5.411278046 7.88E-06 0.012525427

chr15_22385599_22388186 692.4108741 1320.863592 63.95815626 0.04842147 -4.368209324 8.92E-06 0.013669957

chr8_12610662_12614016 698.4134914 1330.870134 65.95684864 0.049559192 -4.334703521 9.41E-06 0.013920217

chr10_118029852_118034658 537.8294152 1041.68106 33.97777051 0.032618209 -4.938178622 1.10E-05 0.01567215

chr1_31974352_31978814 1976.473935 3466.266274 486.6815953 0.140405138 -2.832332362 1.19E-05 0.016490351

chr12_38513693_38522102 1029.06693 1896.239778 161.894083 0.085376377 -3.55001924 1.34E-05 0.017420913

chr6_159880381_159884763 403.754828 793.5188093 13.99084668 0.017631399 -5.825709243 1.31E-05 0.017420913

chr2_203612708_203618375 593.6423768 47.0307491 1140.254004 24.24486163 4.599607115 1.40E-05 0.017610546

chr16_60585050_60590310 435.2721663 851.556755 18.98757764 0.022297489 -5.486974918 1.50E-05 0.018354631

chr8_8951638_8955436 1927.053388 480.3140334 3373.792742 7.024139434 2.812321484 1.54E-05 0.018354631

chr1_37499231_37506566 2707.752051 4623.022571 792.4815299 0.171420649 -2.54438719 1.73E-05 0.020092948

chr5_3308737_3313383 518.8163307 1002.655545 34.9771167 0.034884479 -4.841270886 1.97E-05 0.022214708

chr16_78065943_78072062 509.3107695 984.6437685 33.97777051 0.034507678 -4.856938774 2.18E-05 0.024007164

chr6_17010526_17017558 775.4429389 1452.949951 97.93592677 0.067404887 -3.891003003 2.29E-05 0.024541218

chr19_53539685_53541931 351.7234238 693.4533857 9.993461915 0.014411152 -6.116670557 2.38E-05 0.024726284

chr7_41226060_41231182 603.8588603 1152.75368 54.96404053 0.047680646 -4.390452397 2.42E-05 0.024726284

chr8_8627177_8634269 871.0132667 127.083088 1614.943445 12.70777623 3.667639686 2.50E-05 0.024990613

chr5_3738775_3743400 433.7692229 845.5528296 21.98561621 0.02600147 -5.265263022 2.68E-05 0.026174162

chr8_136801006_136807745 1042.938745 185.1210337 1900.756456 10.26764176 3.360032961 2.95E-05 0.028159915

chr5_27239651_27246964 573.3408682 1094.715734 51.96600196 0.047469859 -4.396844414 3.50E-05 0.032668474

chr5_29112526_29116791 722.5881205 93.06084397 1352.115397 14.5293696 3.860900203 4.20E-05 0.038382959

chr1_241426592_241430857 387.7417441 757.4952568 17.98823145 0.023746989 -5.396111598 4.84E-05 0.03925785

chr10_106592479_106597376 388.7423984 759.4965653 17.98823145 0.023684414 -5.399918184 4.70E-05 0.03925785

chr12_115129662_115131094 720.9092451 1346.880602 94.93788819 0.070487234 -3.826494196 4.85E-05 0.03925785

chr22_21309992_21313516 841.4651898 1553.015375 129.9150049 0.083653393 -3.579432139 4.53E-05 0.03925785

chr6_120459825_120465335 505.8045555 971.6352634 39.97384766 0.041140795 -4.6032865 4.77E-05 0.03925785

chrX_150992722_150999341 454.2780566 879.5750737 28.98103955 0.03294891 -4.92362547 4.45E-05 0.03925785

chr15_25199736_25202492 336.2126291 661.4324502 10.99280811 0.016619699 -5.910961952 5.16E-05 0.041042727

chr17_903245_911773 1268.856386 285.1864573 2252.526316 7.898433666 2.981566582 5.70E-05 0.044461499

chrX_16634114_16638407 1414.701414 2487.626431 341.7763975 0.137390564 -2.863645176 5.81E-05 0.044514929

chr4_139829282_139835884 2782.714133 4640.033693 925.3945733 0.199437037 -2.325994738 6.73E-05 0.050347195

chr8_11426889_11433646 3412.056322 1205.788355 5618.324289 4.659461395 2.220163198 6.80E-05 0.050347195

chr1_2411471_2413279 686.1113305 92.06018973 1280.162471 13.90571185 3.797605695 7.47E-05 0.053209117

chr15_24121776_24124215 259.6671582 515.3369317 3.997384766 0.007756837 -7.010315732 7.39E-05 0.053209117

chr20_33861277_33868158 1259.632709 2227.45633 291.8090879 0.131005526 -2.932300428 7.56E-05 0.053209117

chr4_158739531_158742172 369.2701902 18.01177625 720.5286041 40.00319535 5.322043338 8.28E-05 0.05728641

chr10_110356605_110360753 320.2021614 629.4115146 10.99280811 0.017465216 -5.839371698 8.66E-05 0.058981404

chr11_35639090_35642473 1254.126495 2212.446516 295.8064727 0.133701073 -2.902917054 8.94E-05 0.059692414

chr22_49446873_49449183 450.7724967 867.5672228 33.97777051 0.039164424 -4.674312452 9.04E-05 0.059692414

chr1_60967188_60973502 696.6090437 99.06476939 1294.153318 13.06370899 3.707492653 9.34E-05 0.060739377

chr1_177744666_177752664 813.442293 1489.974158 136.9104282 0.091887787 -3.443983065 9.60E-05 0.061511117

chr15_23508297_23514993 570.8313843 1077.704612 63.95815626 0.059346648 -4.074689644 9.94E-05 0.061829217

chr5_10015921_10021933 451.7724969 868.5678771 34.9771167 0.040269871 -4.634155325 9.88E-05 0.061829217

chr2_76538704_76545917 677.8811129 1260.824338 94.93788819 0.075298267 -3.73123952 0.000101991 0.062527788

chr4_77939775_77946502 1104.437123 244.1596336 1964.714612 8.04684453 3.008423159 0.000106387 0.064304103

chr15_20545062_20549807 626.3565761 1171.766111 80.94704151 0.069081228 -3.855562467 0.000110177 0.065312992

chr6_83275996_83283528 971.507075 1747.142296 195.8718535 0.112109846 -3.157015109 0.0001111 0.065312992

chr14_27065670_27068299 397.2433812 769.5031077 24.98365479 0.032467257 -4.944870708 0.000113499 0.065821742

chr10_131264070_131266388 357.2217899 696.4553484 17.98823145 0.025828262 -5.274905604 0.00011667 0.066397682

chr19_58569717_58572224 310.195619 609.3984299 10.99280811 0.018038786 -5.792753909 0.000119663 0.066397682

chr2_184860199_184866707 891.0276595 169.1105659 1612.944753 9.537811811 3.253658317 0.000120681 0.066397682

chrX_151141134_151145330 294.6867864 580.379457 8.994115723 0.015496957 -6.011871198 0.000119932 0.066397682

chr5_65491552_65497380 910.5201424 177.1157998 1643.924485 9.281636572 3.214379209 0.0001255 0.068175192

chr5_128794910_128798230 564.3258238 1062.694799 65.95684864 0.062065655 -4.010061041 0.000130917 0.070229029

chr11_101166757_101173294 691.3834049 1277.83546 104.9313501 0.08211648 -3.606184402 0.000140125 0.073350698

chrX_30165126_30169710 523.8058674 991.6483481 55.96338672 0.05643471 -4.147273435 0.000140155 0.073350698

chr12_126455700_126461101 521.804559 987.6457312 55.96338672 0.056663422 -4.141438462 0.000146135 0.075558835

chr8_54998885_55006274 1102.553539 1949.274452 255.832625 0.131245051 -2.929665072 0.000153648 0.078497564

chr12_5610888_5616666 429.2577766 823.5384364 34.9771167 0.042471748 -4.557352713 0.0001703 0.085981711

chr10_106231059_106234786 1221.59673 2134.395486 308.7979732 0.144677018 -2.789092332 0.00017636 0.087203627

chr3_139632648_139637519 613.845782 1142.747138 84.94442628 0.074333528 -3.749843109 0.000176785 0.087203627

chr15_51657595_51663010 491.2120083 51.03336605 931.3906505 18.25062156 4.189873693 0.000187424 0.091400921

chr11_70649718_70656932 1119.55485 1968.286883 270.8228179 0.137593163 -2.86151931 0.000193613 0.093358295

chr10_87038676_87041032 314.1962739 614.4017011 13.99084668 0.022771497 -5.456627033 0.000201632 0.095482176

chr2_128371150_128378785 755.5933598 134.0876677 1377.099052 10.2701395 3.360383873 0.000202608 0.095482176

chr5_169486655_169491819 443.2361968 40.02616945 846.4462242 21.14732026 4.402402955 0.000204692 0.095482176

chr15_24728941_24735321 557.317974 1043.682368 70.9535796 0.067983883 -3.878663433 0.000211644 0.097663305

chr15_22525588_22527620 763.9086003 1388.90808 138.9091206 0.100013185 -3.321737889 0.000219804 0.100349943

chr19_52301210_52304389 380.7312783 734.4802094 26.98234717 0.036736657 -4.76663586 0.000228051 0.101946125

chr5_3593989_3597877 322.7005268 629.4115146 15.98953906 0.025403951 -5.298803316 0.000226269 0.101946125

chr14_23833340_23836485 1413.838105 402.263003 2425.413207 6.02942152 2.592019592 0.000232949 0.10306206

chr8_103608451_103617243 2225.950116 3679.405627 772.494606 0.209950923 -2.251875967 0.00023903 0.104673111

chr9_112316117_112322174 553.8150302 1035.677135 71.95292579 0.069474282 -3.84737716 0.000241662 0.10475676

chr10_106377824_106382955 347.7136126 674.4409552 20.98627002 0.031116542 -5.006174468 0.000246394 0.105739978

chr4_44448972_44451644 381.7312785 735.4808636 27.98169336 0.03804544 -4.716132627 0.000251295 0.106775405

chr14_89237049_89245646 1691.240388 530.3467452 2852.134031 5.377866568 2.42703396 0.000255291 0.107409786

chr20_56684987_56689464 1281.108842 2212.446516 349.771167 0.158092485 -2.661159308 0.000258715 0.107793639

chr21_22638497_22645633 538.8071787 1008.65947 68.95488721 0.068362901 -3.870642576 0.000265436 0.10953056

chr19_54113862_54117901 322.6998728 628.4108604 16.98888526 0.027034678 -5.209045017 0.000268902 0.109904217

chr6_43488956_43494758 550.1884761 74.04841348 1026.328539 13.86023671 3.792879991 0.000295963 0.118703355

chr7_21903614_21910389 607.6626545 92.06018973 1123.265119 12.20142086 3.608977255 0.00029548 0.118703355

chr3_188815682_188818429 1284.605899 2211.445862 357.7659366 0.161779197 -2.627901987 0.000299267 0.118916877

chr6_96462353_96465334 592.830081 1097.717697 87.94246485 0.080113917 -3.641803301 0.000306315 0.120601086

chr15_22544107_22546916 1585.713877 2677.750736 493.6770186 0.184362574 -2.439382279 0.00031046 0.121121554

chr15_24651531_24655343 231.6481856 458.2996402 4.996730957 0.01090276 -6.519162803 0.000324884 0.125357401

chr5_9544192_9547128 527.7999821 986.6450769 68.95488721 0.069888239 -3.838806489 0.000328846 0.125357401

chr8_8725834_8732433 804.5809438 164.1072947 1445.054593 8.805547585 3.138412723 0.00033008 0.125357401

chr15_64423103_64428323 556.1871693 78.05103043 1034.323308 13.25188537 3.728125724 0.000337705 0.127128214

chr12_29934121_29937870 579.822884 1073.701995 85.94377247 0.080044345 -3.643056716 0.00034585 0.12849505

chr19_52096008_52098855 420.2486183 800.523389 39.97384766 0.049934641 -4.323815204 0.000347324 0.12849505

chr7_41103627_41108060 390.7338965 748.4893687 32.97842432 0.044059977 -4.50438745 0.000352159 0.129170129

chr1_31949297_31951319 969.4802597 1704.114164 234.846355 0.137811398 -2.859232883 0.00039331 0.138955974

chr1_40494078_40499522 2040.362111 3359.196271 721.5279503 0.214791841 -2.218988904 0.000391619 0.138955974

chr15_24299109_24302448 314.6939849 611.3997383 17.98823145 0.029421392 -5.086990678 0.000399285 0.138955974

chr15_51601758_51607829 419.7522153 41.02682369 798.477607 19.46233062 4.282612579 0.00038704 0.138955974

chr17_80295768_80298743 564.8150325 1046.684331 82.94573389 0.079246179 -3.657514814 0.000383175 0.138955974

chr19_58544358_58546463 251.6593082 495.3238469 7.994769532 0.01614049 -5.953171825 0.000398455 0.138955974

chr7_152939983_152946991 595.8281196 1097.717697 93.938542 0.08557623 -3.546646068 0.000401504 0.138955974

chr7_24756535_24763175 533.3003103 992.6490024 73.95161817 0.074499262 -3.746630054 0.000405322 0.13915501

chr12_131912027_131916322 371.223101 712.4658162 29.98038574 0.042079753 -4.570729945 0.000414209 0.139721354

chr17_55937522_55940080 913.4573569 1613.054629 213.860085 0.132580807 -2.915056152 0.000416739 0.139721354

chr8_28515305_28519050 684.1342209 125.0817795 1243.186662 9.93899085 3.313099376 0.00041092 0.139721354

chr7_105657621_105666125 1284.905442 376.2459928 2193.56489 5.830134891 2.543529263 0.000444434 0.147851911

chr5_65517444_65524788 965.5253864 240.1570167 1690.893756 7.040784314 2.815736148 0.000479226 0.158200014

chr22_37795962_37799328 1052.493358 278.1818777 1826.804838 6.566944092 2.715222173 0.0004921 0.161209674

chr19_52048927_52055658 370.7214658 709.4638535 31.97907813 0.045074993 -4.471528927 0.000519883 0.169021016

chr8_9002917_9010824 3185.277333 1316.860975 5053.69369 3.837682023 1.94023518 0.000524281 0.169169308

chr5_4204117_4208306 364.7181944 698.4566569 30.97973194 0.044354552 -4.494774025 0.000543607 0.174096276

chr8_11244680_11251940 817.0858517 184.1203795 1450.051324 7.875561239 2.977382738 0.00055885 0.17765232

chr1_31942482_31946834 1689.725672 2799.830553 579.6207911 0.207019954 -2.272158267 0.000574966 0.179216666

chr10_91961979_91966439 284.1759928 553.3617926 14.99019287 0.027089317 -5.206132184 0.0006043 0.179216666

chr12_119615436_119619586 463.2649781 868.5678771 57.96207911 0.06673293 -3.905457347 0.000571733 0.179216666

chr12_66216264_66220819 1409.371132 448.2930978 2370.449166 5.287721755 2.402646263 0.000594585 0.179216666

chr14_25838737_25842021 422.2453486 797.5214263 46.969271 0.058894055 -4.085734172 0.000605532 0.179216666

chr17_14639328_14644245 349.2100158 670.4383383 27.98169336 0.041736416 -4.582549473 0.000580878 0.179216666

chr19_48773452_48776004 420.2446942 794.5194636 45.96992481 0.057858777 -4.111320351 0.000584152 0.179216666

chr19_12305074_12307240 254.6599629 499.3264639 9.993461915 0.020013884 -5.64285502 0.000584973 0.179216666

chr2_76603537_76608117 364.7175404 697.4560027 31.97907813 0.045851033 -4.446901955 0.00060333 0.179216666

chrX_33939716_33943120 503.2826452 935.6117109 70.9535796 0.075836566 -3.720962545 0.000599323 0.179216666

chr11_10760907_10768299 975.4717587 1697.109585 253.8339326 0.149568381 -2.741122877 0.000619794 0.180941865

chr8_136591004_136597565 568.688807 93.06084397 1044.31677 11.22187083 3.488241306 0.00061598 0.180941865

chr8_144116278_144122460 775.8955224 1380.902846 170.8881987 0.123751066 -3.014487146 0.000624064 0.1809574

chr1_215646960_215652567 484.2734849 902.5901211 65.95684864 0.073075084 -3.774476613 0.000636538 0.182760914

chr18_45528804_45531102 698.1355319 141.0922473 1255.178817 8.896157234 3.153182287 0.000639298 0.182760914

chr3_194336826_194343999 857.5731069 205.1341184 1510.012095 7.361096764 2.879920736 0.000643059 0.182760914

chr3_129625389_129630509 510.7849359 946.6189075 74.95096436 0.079177548 -3.658764792 0.000658852 0.186017425

chr15_24560029_24565134 364.2165593 695.4546942 32.97842432 0.047419946 -4.398362158 0.000675804 0.188821211

chr19_1025601_1032487 1759.257407 624.4082434 2894.106571 4.634958941 2.212556559 0.000677583 0.188821211

chr15_25786155_25790774 268.6671601 524.3428198 12.99150049 0.02477673 -5.334870393 0.0006834 0.18921358

chr17_78249900_78255880 752.1165768 166.1086032 1338.12455 8.055720923 3.010013704 0.000734744 0.200116245

chr3_175051735_175058845 748.8817821 1332.871443 164.8921216 0.123711947 -3.014943262 0.000736767 0.200116245

chr5_135028708_135034448 544.2983506 1000.654236 87.94246485 0.087884967 -3.508239776 0.000727786 0.200116245

chr3_180010987_180017072 472.7666152 880.5757279 64.95750245 0.073767083 -3.76087901 0.000750234 0.201226834

chr5_34419264_34425661 1008.979287 1742.139025 275.8195489 0.158322352 -2.65906314 0.000747752 0.201226834

chr19_52204993_52208821 567.8078389 1038.679097 96.93658058 0.093326785 -3.421564996 0.000758266 0.20211782

chr10_108362748_108367859 310.6887519 599.3918875 21.98561621 0.036679869 -4.768867684 0.000787206 0.203642748

chr12_131939112_131943010 381.2237571 723.4730128 38.97450147 0.053871396 -4.214336728 0.000787713 0.203642748

chr5_9556992_9563526 467.2636709 870.5691855 63.95815626 0.073467057 -3.7667587 0.000785597 0.203642748

chr7_31444992_31450688 522.7882086 963.6300295 81.9463877 0.085039263 -3.555727093 0.000780556 0.203642748

chr7_44111671_44114350 1042.009379 292.191037 1791.827721 6.132384278 2.616448104 0.000777943 0.203642748

chr15_24503070_24506812 345.2060908 660.4317959 29.98038574 0.045395128 -4.461318728 0.000798992 0.20427318

chr4_190700227_190703609 224.1419708 441.2885182 6.99542334 0.015852267 -5.979167033 0.000799671 0.20427318

chr12_116826706_116829730 971.4619475 1678.097154 264.8267407 0.157813712 -2.663705534 0.000861825 0.217501143

chr19_53634941_53636910 307.186462 592.3873079 21.98561621 0.037113584 -4.751908857 0.00086666 0.217501143

chr22_27634727_27641835 2548.015587 1043.682368 4052.348807 3.882741463 1.957075647 0.000862003 0.217501143

chr16_34749353_34753674 323.6946408 621.4062807 25.98300098 0.041813226 -4.57989685 0.0008994 0.223374614

chr7_148085487_148090312 646.3376137 1162.760223 129.9150049 0.111729832 -3.16191365 0.000900473 0.223374614

chr19_37287702_37289552 258.6606178 504.3297351 12.99150049 0.025759934 -5.278727315 0.00093421 0.225233845

chr3_8750189_8756375 2079.668201 808.5286229 3350.80778 4.144327962 2.051138175 0.000921143 0.225233845

chr5_175267490_175275593 1053.510363 305.1995421 1801.821183 5.903747991 2.561631139 0.000933019 0.225233845

chr6_106957819_106965349 1184.535244 2003.309781 365.7607061 0.182578206 -2.45341353 0.000927035 0.225233845

chr6_164570030_164576217 823.5940283 203.13281 1444.055247 7.10892173 2.829630751 0.000930832 0.225233845

chr19_52067006_52071763 258.1602907 503.3290808 12.99150049 0.025811146 -5.275861981 0.000948922 0.22750278

chr12_125562435_125567569 590.1858685 110.071966 1070.299771 9.723636364 3.281495942 0.000956459 0.228035838

chr1_156659074_156665086 2127.84349 3418.234871 837.4521085 0.244995485 -2.029172935 0.000967644 0.229427797

chr14_32738823_32745296 1042.016573 303.1982336 1780.834913 5.873500291 2.554220529 0.001005503 0.237094217

chr2_38619466_38627128 759.6201755 179.1171083 1340.123243 7.481827144 2.903390635 0.001021678 0.238289727

chr6_31475138_31479609 370.2165605 701.4586196 38.97450147 0.055562082 -4.169755525 0.001018524 0.238289727

chr21_45899602_45904747 884.5737667 233.152437 1535.995096 6.587943561 2.719828195 0.001029772 0.238879356

chr17_17463826_17466261 814.5999126 203.13281 1426.067015 7.020367687 2.811546593 0.001043232 0.240700589

chrX_115007573_115012820 427.2571221 56.03663723 798.477607 14.24920635 3.832809661 0.001056332 0.242419833

chr1_58683441_58690248 454.7450286 65.04252536 844.4475318 12.98300654 3.698552609 0.00107784 0.242843727

chr19_54482651_54485255 199.62725 394.2577691 4.996730957 0.012673767 -6.302010834 0.001086473 0.242843727

chr21_18947884_18955185 593.1865232 114.0745829 1072.298463 9.399977067 3.232657237 0.001083061 0.242843727

chr7_155106969_155109944 505.2767595 928.6071312 81.9463877 0.088246563 -3.5023161 0.001074597 0.242843727

chr8_70801192_70808531 763.6195224 182.119071 1345.119974 7.385936939 2.884780945 0.001065515 0.242843727

chr13_24288533_24294628 824.9014191 1438.940792 210.8620464 0.146539766 -2.770635881 0.001146865 0.253699595

chr17_5785283_5789608 283.1727224 547.3578672 18.98757764 0.034689513 -4.849356619 0.001142616 0.253699595

chr6_54710475_54714576 867.4167977 1504.983971 229.849624 0.152725629 -2.71098591 0.001155974 0.254403171

chr2_138977214_138983583 462.7424142 69.0451423 856.4396861 12.40405418 3.632739828 0.001170385 0.256260677

chr1_19661014_19668610 737.6319432 175.1144913 1300.149395 7.424567694 2.892307025 0.001208239 0.261876653

chr18_14794287_14801900 1859.746637 3001.962709 717.5305655 0.239020479 -2.064793861 0.001206128 0.261876653

chrX_37153459_37158535 422.7391355 788.5155382 56.96273292 0.07224047 -3.791048915 0.001233634 0.266037282

chr7_43929268_43935474 995.5391272 291.1903827 1699.887872 5.83771983 2.545404973 0.001249649 0.268143482

chr1_111017798_111023851 464.2427415 71.04645077 857.4390323 12.0687103 3.593199608 0.001275865 0.272406594

chr19_53560554_53562413 363.711654 687.4494603 39.97384766 0.058148053 -4.104125303 0.001288578 0.273759128

chr11_133903016_133906020 1168.981283 376.2459928 1961.716574 5.213920178 2.382368496 0.001323436 0.279152496

chr17_11181291_11184480 223.1400085 437.2859012 8.994115723 0.020568044 -5.603451578 0.001346489 0.279152496

chr3_151523273_151529683 615.3186404 1102.720968 127.9163125 0.116000617 -3.107795618 0.001340438 0.279152496

chr3_168958148_168963138 416.264314 56.03663723 776.4919908 13.85686275 3.792528757 0.001327438 0.279152496

chr4_8199034_8204237 1924.237822 759.4965653 3088.979078 4.067140286 2.024014754 0.001340362 0.279152496

chr5_134995051_135000511 394.7253951 739.4834806 49.96730957 0.067570556 -3.887461474 0.001382568 0.285254393

chr11_121383223_121387575 352.2943854 38.02486098 666.5639097 17.52968696 4.131728328 0.001429059 0.292038498

chr8_10509093_10513761 1436.895664 513.3356232 2360.455704 4.598269821 2.201091124 0.00142785 0.292038498

chr15_24627850_24631773 309.1845004 591.3866536 26.98234717 0.04562556 -4.454013928 0.001464772 0.297917983

chr16_830849_836597 1038.529326 319.2087014 1757.849951 5.506898601 2.461240044 0.001485737 0.299914318

chr8_139287399_139290913 269.1642171 520.3402028 17.98823145 0.034570136 -4.854329921 0.001488565 0.299914318

chr1_37216012_37223735 965.9446147 1646.076219 285.8130108 0.173632914 -2.525887641 0.001517695 0.301405428

chr10_106165074_106169033 323.6913707 616.4030095 30.97973194 0.050258891 -4.31447734 0.001514462 0.301405428

chr16_78492509_78498749 1052.974718 1779.163232 326.7862046 0.1836741 -2.444779893 0.001507068 0.301405428

chr8_123710123_123712936 953.559393 280.1831861 1626.9356 5.806685341 2.537714858 0.001524059 0.301405428

chr7_37959425_37961684 593.8078445 1064.696107 122.9195816 0.115450391 -3.11465504 0.00153929 0.303021178

chrX_16582463_16587236 1336.06765 2204.441282 467.6940176 0.212159889 -2.23677617 0.001553285 0.304380051

chr10_54698984_54703051 362.2904634 42.02747792 682.5534488 16.24064737 4.021537236 0.001570418 0.304952458

chr12_3550287_3557409 862.5920746 239.1563625 1486.027787 6.213624306 2.635435015 0.001564387 0.304952458

chr1_86966878_86974495 1448.896974 527.3447825 2370.449166 4.495065173 2.168342033 0.001614691 0.312137162

chr14_66281485_66286071 621.3173337 1106.723585 135.911082 0.122804903 -3.025559939 0.001654096 0.318156523

chr19_51593506_51598978 1631.157968 2637.724567 624.5913697 0.236791732 -2.078309385 0.001689146 0.318156523

chr2_163346208_163350115 291.6750133 559.3657181 23.9843086 0.042877688 -4.543629082 0.00167568 0.318156523

chr2_42000065_42004721 265.1616002 512.334969 17.98823145 0.035110294 -4.831962108 0.001670603 0.318156523

chr2_224791968_224798892 561.206137 112.0732745 1010.339 9.014985994 3.17232525 0.001669127 0.318156523

chr6_40961182_40968892 1716.814625 669.4376841 2764.191566 4.129124535 2.045835931 0.001690311 0.318156523

chr1_46442492_46448321 1625.655024 2627.718024 623.5920235 0.237313143 -2.075136092 0.001732841 0.322160429

chr19_52132225_52137143 286.1720691 549.3591757 22.9849624 0.04183959 -4.578987492 0.001734104 0.322160429

chr4_28313576_28319493 522.2774171 946.6189075 97.93592677 0.103458663 -3.272873639 0.001731489 0.322160429

chr2_41682197_41686770 268.1629089 517.3382401 18.98757764 0.036702444 -4.767980066 0.001751248 0.322891873

chr9_114771916_114777692 727.8562708 1272.832189 182.880353 0.143679862 -2.799070227 0.001753089 0.322891873

chr8_86850934_86857170 951.9348014 1617.057246 286.812357 0.177366854 -2.495191666 0.001805935 0.33120389

chr1_54024795_54028230 1263.961683 441.2885182 2086.634848 4.728504735 2.241384041 0.001837772 0.335608515

chr1_179058328_179066092 895.0858683 262.1714099 1528.000327 5.828249264 2.54306258 0.001904219 0.336857107

chr12_3236971_3244484 1054.031618 338.2211319 1769.842105 5.232795761 2.387581951 0.001889338 0.336857107

chr12_1713430_1719950 2664.035233 1189.777887 4138.292579 3.478205995 1.798343378 0.001877047 0.336857107

chr14_23113033_23118334 2165.68457 919.6012431 3411.767898 3.71005142 1.891439182 0.001909733 0.336857107

chr18_32846424_32848437 405.2270324 752.4919857 57.96207911 0.077026839 -3.698494966 0.001885642 0.336857107

chr6_166246666_166249730 269.6632362 519.3395486 19.98692383 0.038485272 -4.699549743 0.001907028 0.336857107

chr7_2811877_2819720 1246.968874 435.2845928 2058.653154 4.729441815 2.241669922 0.001921397 0.336857107

chr7_41743637_41745984 370.7116555 694.45404 46.969271 0.067634816 -3.88609011 0.001882547 0.336857107

chr7_73516643_73521648 431.2617011 67.04383383 795.4795684 11.86506682 3.568648321 0.001923104 0.336857107

chr8_101835830_101839637 1042.036194 333.2178607 1750.854528 5.254383796 2.393521583 0.001922926 0.336857107

chr10_52832898_52835474 320.6881 608.3977756 32.97842432 0.054205366 -4.205420504 0.001962923 0.342434392

chr1_35289688_35294272 1555.626886 2514.644096 596.6096763 0.237254122 -2.075494944 0.001980047 0.343662094

chr12_34260490_34264990 432.2381565 796.5207721 67.95554102 0.085315466 -3.551048889 0.001986139 0.343662094

chr3_18464149_18468075 977.9406931 1652.080144 303.8012422 0.183890136 -2.443084001 0.001995424 0.343662094

chrX_150891199_150897010 330.6926803 625.4088977 35.97646289 0.057524706 -4.119674488 0.002001993 0.343662094

chr11_134276542_134283362 536.2192121 107.0700033 965.368421 9.016236027 3.172525283 0.002037436 0.347100362

chr14_77049169_77053856 300.3218432 28.01831861 572.6253677 20.43753501 4.353149297 0.002046287 0.347100362

chr16_34682706_34687517 363.7077298 681.4455349 45.96992481 0.067459426 -3.889836141 0.002044272 0.347100362

chr1_172289430_172297366 1178.993712 405.2649657 1952.722458 4.818384572 2.268549545 0.002073826 0.347478534

chr10_6337147_6342218 400.2237612 742.4854433 57.96207911 0.078064937 -3.679181491 0.002089001 0.347478534

chr19_1108608_1114812 644.1780318 152.0994439 1136.25662 7.470485036 2.901201916 0.002085399 0.347478534

chr8_70712380_70719485 893.5888111 265.1733726 1522.00425 5.739657171 2.520964567 0.002079177 0.347478534

chrX_4626610_4629720 846.6051516 243.1589794 1450.051324 5.963387934 2.576132191 0.002086664 0.347478534

chr17_54246609_54253276 2472.101248 1098.718351 3845.484145 3.499972618 1.807343635 0.002135104 0.352415396

chr18_74202160_74207948 715.3474388 1246.815178 183.8796992 0.147479516 -2.761413507 0.002130849 0.352415396

chr6_159870833_159874469 189.1197265 372.2433759 5.996077149 0.016107949 -5.95608342 0.002155968 0.354495603

chr5_170887505_170893502 586.2009102 129.0843965 1043.317424 8.08244414 3.014791632 0.002189566 0.358645977

chr1_3424703_3429381 326.3100763 36.0235525 616.5966002 17.11648511 4.097314568 0.002231042 0.362671096

chrX_150919823_150924714 272.6638909 523.3421656 21.98561621 0.042010023 -4.573122627 0.00223074 0.362671096

chr10_24156631_24160964 311.1825388 590.3859994 31.97907813 0.05416639 -4.206458254 0.00226509 0.366816411

chr14_106655667_106661373 466.2499362 848.5547923 83.94508009 0.098927118 -3.337490141 0.002409086 0.379854638

chr15_63501927_63507239 441.7600683 75.04906772 808.4710689 10.77256645 3.429290092 0.002425263 0.379854638

chr15_24761286_24764647 274.1642182 525.343474 22.9849624 0.043752256 -4.514498766 0.002398164 0.379854638

chr17_4621822_4626924 535.7221551 111.0726202 960.37169 8.646340458 3.112089645 0.002420311 0.379854638

chr19_52481844_52485328 954.4272806 1608.051358 300.8032036 0.187060694 -2.418421646 0.002393725 0.379854638

chr21_41709193_41714538 316.1845019 598.3912333 33.97777051 0.056781865 -4.138425942 0.0023906 0.379854638

chr5_38845105_38846871 330.6913722 623.4075892 37.97515528 0.060915452 -4.037047949 0.002359855 0.379854638

chr6_41733823_41737617 1362.941429 509.3330063 2216.549853 4.351867689 2.121634695 0.002420525 0.379854638

chr7_149427942_149433918 2262.171184 995.6509651 3528.691402 3.544104838 1.825421281 0.00240841 0.379854638

chr3_120376940_120384000 795.8726359 1365.893032 225.8522393 0.165351337 -2.596393383 0.002455681 0.383220107

chr1_64155726_64162226 475.246668 88.05757279 862.4357633 9.793998812 3.291898021 0.002492481 0.386509404

chr11_69256505_69260575 1378.061119 2236.462218 519.6600196 0.232358059 -2.105578413 0.002509331 0.386509404

chr18_5236933_5238965 422.7312873 776.5076873 68.95488721 0.088801294 -3.493275495 0.00252179 0.386509404

chr3_31410890_31413322 732.1499277 197.1288845 1267.170971 6.428134435 2.684400101 0.002507639 0.386509404

chr8_61859192_61864826 693.8359888 1207.789663 179.8823145 0.148935133 -2.747243974 0.002516989 0.386509404

chr9_133048501_133052863 1320.45679 490.3205758 2150.593004 4.386095772 2.132937314 0.002544605 0.388618238

chr5_166212946_166217560 245.6494966 474.310108 16.98888526 0.035818097 -4.803167517 0.002569156 0.390976324

chr1_86880518_86884554 443.7600687 77.05037619 810.4697613 10.5186996 3.394884454 0.002587381 0.390977013

chr5_17111967_17116889 1561.883264 619.4049722 2504.361556 4.043173155 2.015487991 0.002581395 0.390977013

chr6_93650183_93654607 283.1681443 540.3532876 25.98300098 0.048085209 -4.378262988 0.00259675 0.391015814

chr19_8923834_8926842 240.1465523 464.3035656 15.98953906 0.034437683 -4.859868105 0.002633633 0.395183047

chr12_8970221_8977050 2017.749941 871.5698398 3163.930042 3.630150905 1.860029522 0.00269208 0.402545724

chr11_87905094_87909649 398.7796798 62.04056265 735.5187969 11.85545014 3.567478536 0.002742399 0.408175521

chr15_20980246_20981924 442.7384858 807.5279687 77.94900294 0.09652793 -3.372909754 0.002757474 0.408175521

chr2_110476633_110481535 389.783602 59.03859994 720.5286041 12.20436468 3.60932529 0.002758264 0.408175521

chr19_42200068_42207331 1024.554176 343.224403 1705.883949 4.970170926 2.313295467 0.002792495 0.411821075

chr7_155607769_155613963 554.717581 123.0804711 986.354691 8.013900845 3.00250466 0.002847094 0.418435024

chr15_24343917_24347657 250.6514598 482.3153419 18.98757764 0.039367559 -4.666848932 0.002877834 0.42046482

chrX_16678132_16683142 1984.235218 3109.032712 859.4377247 0.276432513 -1.855000786 0.0028805 0.42046482

chr2_236351352_236355301 476.7515735 861.5632974 91.93984962 0.106712821 -3.228194581 0.002895787 0.42126342

chr19_58608610_58610451 307.6789409 581.3801113 33.97777051 0.058443297 -4.096818622 0.002935277 0.425565532

chr16_4200031_4206124 594.2963992 1047.684985 140.907813 0.134494447 -2.894381484 0.002946982 0.425823965

chr18_922337_925670 799.6306485 235.1537455 1364.107551 5.800917814 2.53628118 0.002967118 0.427294925

chr13_70401296_70404241 330.8104043 41.02682369 620.5939849 15.12654232 3.919010344 0.002978394 0.42748416

chr19_42710269_42713337 955.5790141 312.2041217 1598.953906 5.121501592 2.356566861 0.003005314 0.429910169

chr19_43768134_43772034 284.1674905 540.3532876 27.98169336 0.051784072 -4.271347785 0.003079173 0.437558588

chr20_17984407_17989236 1225.494049 452.2957148 1998.692383 4.418994737 2.143718213 0.003070165 0.437558588

chr15_48026935_48032098 350.8019063 48.03140334 653.5724092 13.60718954 3.766297215 0.00312712 0.44144847

chr8_11330628_11333157 834.6201916 254.166176 1415.074207 5.567515825 2.477033754 0.00312189 0.44144847

chr4_154124166_154129587 405.7783732 67.04383383 744.5129127 11.10486782 3.473120315 0.003156402 0.444121288

chr8_29450056_29456345 1536.097778 2450.602225 621.5933311 0.253649215 -1.979093398 0.003193615 0.447888818

chr9_80136620_80142070 501.2401334 104.0680406 898.4122262 8.632931121 3.109850478 0.00320468 0.447976731

chr3_194703821_194710382 529.7293481 116.0758914 943.3828048 8.127293216 3.022774945 0.003239483 0.451371477

chr7_17313234_17318502 342.8058287 46.03009487 639.5815626 13.89485649 3.796479029 0.003252294 0.451689899

chr10_46511072_46516946 351.8019065 49.03205758 654.5717554 13.34987328 3.738754143 0.003269684 0.452640236

chr1_197750912_197754458 1081.541762 381.249264 1781.834259 4.673672654 2.224556691 0.00329588 0.45471704

chr18_74397092_74402052 430.2309619 783.512267 76.94965675 0.09821117 -3.347969066 0.003305877 0.45471704

chr20_49775659_49779578 1042.054507 361.2361793 1722.872834 4.769380624 2.253801923 0.003319385 0.455116259

chr10_131328568_131331903 183.6154742 360.235525 6.99542334 0.019419027 -5.686385284 0.003371462 0.458376066

chr12_119606314_119611682 311.6795958 586.3833824 36.97580909 0.063057396 -3.987190598 0.003403212 0.458376066

chr2_9131975_9137825 686.6731357 187.1223422 1186.223929 6.339296075 2.66432265 0.003407246 0.458376066

chr21_30011954_30016707 420.7267087 767.5017992 73.95161817 0.096353674 -3.375516511 0.00339934 0.458376066

chr6_50182049_50184757 655.1839204 172.1125286 1138.255312 6.613436693 2.725400167 0.003357396 0.458376066

chr9_38619477_38622576 516.7646625 921.6025516 111.9267734 0.121447986 -3.041589534 0.003398257 0.458376066

chr7_73470580_73478748 1583.891771 654.4278705 2513.355672 3.840538866 1.94130875 0.00347779 0.466404283

chr2_206078523_206084383 397.2832766 66.04317959 728.5233736 11.03101604 3.463493775 0.00352769 0.471622436

chr2_70841789_70845685 417.724746 761.4978738 73.95161817 0.097113361 -3.364186387 0.003595811 0.47698728

chr6_125591775_125598203 799.6358806 243.1589794 1356.112782 5.57706232 2.479505392 0.00359974 0.47698728

chr9_130149795_130153608 1018.064966 353.2309454 1682.898986 4.764302246 2.25226494 0.003601162 0.47698728

chr2_242711868_242717939 2456.146372 1151.753026 3760.539719 3.265057381 1.707108346 0.00367086 0.484722979

chr8_128060125_128068438 1160.521504 429.2806674 1891.762341 4.406819324 2.139737749 0.003692754 0.486118235

chr9_95735405_95739223 1044.942016 1721.125286 368.7587447 0.214254446 -2.222602953 0.003707855 0.48661349

chrX_153028084_153033260 1144.02706 421.2754335 1866.778686 4.43125456 2.147715207 0.003738111 0.489088569

chr1_107161348_107166584 582.7123548 143.0935558 1022.331154 7.144494721 2.836831983 0.003756033 0.489939619

chr1_48991046_48993201 615.7005895 158.1033693 1073.29781 6.788582775 2.76311042 0.003782448 0.491890161

chr5_171022177_171025640 222.864011 15.00981354 430.7182085 28.69577342 4.842766354 0.003844566 0.498457903

chr4_23036375_23039135 222.6357572 430.2813216 14.99019287 0.034838121 -4.843189364 0.003859394 0.498873201

chr2_19298343_19305440 1803.83688 790.5168466 2817.156914 3.563689915 1.83337181 0.004064123 0.523759335

chr1_60925620_60929748 254.3486483 23.01504743 485.6822491 21.1028133 4.399363438 0.004100242 0.526832029

chr3_194721965_194725152 1142.031637 426.2787046 1857.78457 4.358145386 2.123714325 0.004132388 0.528135197

chr8_103134458_103142433 1806.337208 793.5188093 2819.155606 3.552726883 1.828926785 0.004134998 0.528135197

chr3_83982120_83985560 210.6292144 408.2669284 12.99150049 0.031821094 -4.973872733 0.004166209 0.530542557

chr11_70258904_70265246 975.5835965 339.2217861 1611.945407 4.75189234 2.24850215 0.004187408 0.531664593

chr15_28909980_28914200 437.2695507 85.05561008 789.4834913 9.281968474 3.214430797 0.004257457 0.535853323

chr20_15275861_15280446 373.7044619 686.4488061 60.96011768 0.088805046 -3.493214538 0.004254522 0.535853323

chr7_157914568_157918214 256.6521151 489.3199215 23.9843086 0.049015598 -4.350615264 0.004257858 0.535853323

chr12_89762910_89767852 1373.964323 555.3631011 2192.565544 3.947985633 1.98111674 0.00432618 0.542859655

chr10_70959334_70965242 407.7816438 74.04841348 741.5148741 10.0139198 3.323934901 0.004351026 0.544385692

chr2_76014777_76018657 393.7123144 718.4697416 68.95488721 0.095974657 -3.381202687 0.004400983 0.549035433

chr6_53327158_53331756 1552.585682 2448.600916 656.5704478 0.268141061 -1.898935935 0.00443246 0.549765946

chrX_16619111_16626452 2620.882837 3971.596664 1270.169009 0.319813193 -1.644698642 0.004422039 0.549765946

chr1_110705759_110710029 1047.563991 381.249264 1713.878718 4.495428268 2.168458564 0.004519966 0.550919452

chr11_101311376_101314492 875.8811553 1458.953876 292.8084341 0.200697526 -2.316905259 0.004631794 0.550919452

chr12_107882542_107889950 865.1218332 287.1877658 1443.055901 5.024781945 2.329060991 0.004621876 0.550919452

chr14_67943745_67951326 1323.017288 2114.382401 531.6521739 0.251445611 -1.991681726 0.004598094 0.550919452

chr15_64146706_64153771 1289.507797 2066.350998 512.6645962 0.24810141 -2.01099816 0.004562465 0.550919452

chr17_12632349_12636936 389.2100244 710.4645077 67.95554102 0.095649452 -3.386099483 0.004558509 0.550919452

chr19_50716205_50720310 592.2865884 1030.673863 153.8993135 0.149319119 -2.743529191 0.004615668 0.550919452

chr19_54123000_54128988 286.1655288 539.3526333 32.97842432 0.061144458 -4.031634453 0.004543484 0.550919452

chr2_167487719_167489952 663.1885002 187.1223422 1139.254658 6.088287722 2.60603654 0.004623996 0.550919452

chr22_24995087_25000956 1715.868909 751.4913314 2680.246486 3.566570063 1.834537315 0.004579251 0.550919452

chr3_188691523_188698276 392.7116602 716.4684332 68.95488721 0.096242743 -3.37717843 0.004485979 0.550919452

chr5_169481710_169486607 539.2316392 129.0843965 949.3788819 7.354714496 2.878669338 0.004473824 0.550919452

chr7_26001928_26009200 777.6496104 242.1583252 1313.140896 5.422654351 2.438999214 0.004535188 0.550919452

chrX_16650330_16653164 674.8157101 1157.756951 191.8744688 0.16572949 -2.593097758 0.00452528 0.550919452

chrX_149530174_149534663 1223.012034 477.3120707 1968.711997 4.12458037 2.044247349 0.004634322 0.550919452

chr17_6578423_6581634 902.3893362 1497.979392 306.7992808 0.204808746 -2.28765077 0.004663239 0.552825644

chr10_106399728_106401854 174.109259 341.2230946 6.99542334 0.020501025 -5.608160117 0.004719969 0.55727924

chr12_110114150_110121271 405.7836054 75.04906772 736.5181431 9.813821351 3.294815009 0.004739763 0.55727924

chr7_50569237_50572101 1050.064973 385.251881 1714.878065 4.451316527 2.154232092 0.004734879 0.55727924

chr2_190403932_190409224 529.7633573 932.6097482 126.9169663 0.136087969 -2.877388567 0.004793587 0.562067683

chr10_8582266_8589048 948.0976521 333.2178607 1562.977443 4.690557224 2.229759321 0.004905 0.572243633

chr8_54056710_54062545 673.1865403 194.1269218 1152.246159 5.935529951 2.569376846 0.004907041 0.572243633

chr1_40253974_40255746 200.122999 388.2538437 11.9921543 0.030887407 -5.016837451 0.004963412 0.577248851

chr10_54439469_54445692 710.6744489 213.1393523 1208.209546 5.668636657 2.503001799 0.005005 0.580512365

chr3_116687824_116694392 401.7136242 728.476284 74.95096436 0.102887309 -3.280863059 0.005086705 0.588398718

chr1_71051451_71056807 470.2597474 103.0673863 837.4521085 8.125287138 3.022418797 0.005136877 0.592605053

chr12_62170468_62177167 689.6822927 204.1334642 1175.231121 5.757170319 2.525359893 0.005168826 0.593102057

chr6_17280396_17283558 467.238818 832.5443246 101.9333115 0.122435897 -3.029901486 0.00516513 0.593102057

chr4_124466572_124469308 737.1669333 228.1491659 1246.184701 5.462148836 2.449468626 0.005241901 0.598287666

chr5_154445899_154450533 245.1452454 467.3055283 22.9849624 0.049186156 -4.345603893 0.005228923 0.598287666

chr20_33529365_33534913 1663.891788 734.4802094 2593.303367 3.530800876 1.819995461 0.005275818 0.599535668

chr20_50487635_50494469 1586.413681 690.451423 2482.37594 3.595294118 1.846109797 0.005290779 0.599535668

chr3_16471608_16477386 329.3166172 49.03205758 609.6011768 12.43270642 3.636068479 0.005294746 0.599535668

chr6_166044687_166048841 211.6285606 408.2669284 14.99019287 0.036716647 -4.767421856 0.005363675 0.60574236

chr19_41413246_41416788 743.8340374 1254.820412 232.8476626 0.18556254 -2.430022598 0.005413085 0.609717985

chr2_150022941_150029663 1804.855193 819.5358195 2790.174567 3.404579154 1.767476475 0.005501261 0.616110785

chr5_42497972_42502070 1274.004851 517.3382401 2030.671461 3.92522977 1.972777107 0.00551291 0.616110785

chr8_124177328_124181769 1709.881334 764.4998365 2655.262831 3.473202614 1.796266575 0.005490397 0.616110785

chr3_41142258_41146352 335.8143295 52.03402028 619.5946387 11.9074912 3.573797578 0.005535785 0.61706032

chr5_174106809_174111644 1192.029032 472.3087995 1911.749264 4.047668107 2.017090999 0.005551727 0.617234139

chr5_135169310_135172134 348.6907221 640.4187112 56.96273292 0.088946078 -3.49092519 0.005636456 0.620065175

chr6_37810300_37816636 608.7117064 168.1099117 1049.313501 6.241830065 2.64196908 0.00560377 0.620065175

chr7_142254711_142257290 234.6396839 448.2930978 20.98627002 0.046813725 -4.416924609 0.005649435 0.620065175

chr8_87210032_87214727 329.1825426 608.3977756 49.96730957 0.082129343 -3.605958433 0.005622713 0.620065175

chr9_98490453_98495676 404.2865482 78.05103043 730.522066 9.35954416 3.226438268 0.005608802 0.620065175

chr17_38735222_38740227 491.2460174 867.5672228 114.924812 0.132467905 -2.916285242 0.005707817 0.624783999

chr17_80506214_80508703 779.1558239 253.1655218 1305.146126 5.155307551 2.366058497 0.005721545 0.624783999

chr15_48308815_48310960 591.718243 161.105332 1022.331154 6.345731336 2.665786442 0.005774163 0.628929424

chr8_88672211_88677952 975.4043944 1594.042198 356.7665904 0.223812513 -2.159637397 0.005790975 0.629163813

chr11_60597957_60600004 599.2159555 165.107949 1033.323962 6.258474946 2.645811147 0.005850587 0.629268008

chr20_3201428_3205861 1930.324808 898.5875042 2962.062112 3.296353553 1.720870988 0.005844659 0.629268008

chr3_116637238_116641745 421.7195147 757.4952568 85.94377247 0.113457836 -3.139771845 0.00582726 0.629268008

chr9_95764867_95767995 1188.967553 1904.245012 473.6900948 0.248754804 -2.007203707 0.005812369 0.629268008

chr10_9101964_9108070 563.2708857 977.6391888 148.9025825 0.15230832 -2.714933341 0.005973114 0.638486689

chr16_54208410_54213314 944.3939234 1547.011449 341.7763975 0.22092687 -2.178359199 0.005963861 0.638486689

chr6_53343003_53350554 526.2577973 920.6018973 131.9136973 0.143290708 -2.802983041 0.005980931 0.638486689

chr5_171863411_171865888 705.6809881 218.1426235 1193.219353 5.469904659 2.451515687 0.006012794 0.640295468

chr5_139256117_139263985 872.1283749 304.1988878 1440.057862 4.733935329 2.243039997 0.00605752 0.64346161

chr11_100993897_101000873 2763.892677 4129.700033 1398.085322 0.338544037 -1.562584585 0.006089405 0.645251415

chr6_164433316_164438034 440.7738026 95.06215244 786.4854527 8.273381493 3.048477108 0.006126142 0.647545287

chr11_64630133_64633340 781.1577864 258.168793 1304.14678 5.051527588 2.336719726 0.006241722 0.656343409

chr16_15235438_15242297 1727.884608 787.5148839 2668.254331 3.388195431 1.760517092 0.006283989 0.656343409

chr5_15481205_15485887 326.8195598 51.03336605 602.6057535 11.80807382 3.56170174 0.006301141 0.656343409

chr5_168388634_168394052 377.798642 70.04579654 685.5514874 9.787189542 3.29089464 0.006288686 0.656343409

chr9_91386913_91395041 1470.455514 638.4174027 2302.493625 3.606564632 1.850625278 0.006274486 0.656343409

chrX_55844304_55847016 219.1315053 420.2747792 17.98823145 0.04280112 -4.546207626 0.006249231 0.656343409

chr10_34231522_34236725 345.6881053 633.4141315 57.96207911 0.091507398 -3.449967804 0.006329403 0.65769092

chr1_224918761_224923449 1078.931558 1739.137063 418.7260542 0.240766564 -2.054293043 0.006365826 0.65898072

chr10_70975675_70978686 951.106155 349.2283285 1552.983982 4.446901511 2.152800453 0.006393633 0.65898072

chr12_30035797_30040138 314.6750182 582.3807655 46.969271 0.08065045 -3.632173601 0.006379044 0.65898072

chr2_115918219_115920195 182.1125308 354.2315996 9.993461915 0.028211661 -5.147564565 0.006403238 0.65898072

chr16_71618061_71620653 630.7921558 1077.704612 183.8796992 0.170621613 -2.551127688 0.006454649 0.6611009

chr7_55246629_55251068 1426.469239 615.4023553 2237.536123 3.635891386 1.862309103 0.006447387 0.6611009

chr1_85237809_85244739 811.6496177 276.1805692 1347.118666 4.877673582 2.286193215 0.006534075 0.663730527

chr14_24731063_24734254 1307.00355 548.3585215 2065.648578 3.766967225 1.913403481 0.006542188 0.663730527

chr14_64128619_64132170 349.6894142 639.418057 59.96077149 0.093773973 -3.414668635 0.006505327 0.663730527

chrX_56257077_56260828 360.1936676 656.429179 63.95815626 0.097433445 -3.359439114 0.006513596 0.663730527

chr4_26235492_26241411 450.7711886 101.0660779 800.4762994 7.92032615 2.98555984 0.006559634 0.663930893

chr10_129704370_129706426 221.1321598 423.2767419 18.98757764 0.044858542 -4.478473449 0.006613412 0.667799013

chr1_96522632_96528876 568.2702327 981.6418058 154.8986597 0.1577955 -2.66387203 0.006663602 0.668284057

chr7_150276286_150279468 276.6580055 518.3388944 34.9771167 0.067479244 -3.88941238 0.006664932 0.668284057

chr9_32738485_32745246 655.2002709 197.1288845 1113.271657 5.64743041 2.497594588 0.006664165 0.668284057

chr8_99176080_99180859 1568.43199 700.4579654 2436.406015 3.478304388 1.798384189 0.006691711 0.669405083

chr12_125593094_125599343 893.6254364 321.2100098 1466.040863 4.56411948 2.190336559 0.006742246 0.672891856

chr1_172326319_172331297 1470.959765 645.4219824 2296.497548 3.558133455 1.831120623 0.006804521 0.677531318

chr15_83218760_83224834 445.2257329 790.5168466 99.93461915 0.126416811 -2.983739763 0.006884611 0.683305346

chr18_61636579_61638928 499.2453651 874.5718025 123.9189277 0.141690971 -2.819180269 0.006894354 0.683305346

chr2_180897449_180900275 367.3041989 68.04448806 666.5639097 9.796001538 3.292193 0.006963869 0.688604733

chr14_77152876_77159371 502.7535411 126.0824338 879.4246485 6.974997406 2.80219268 0.007026779 0.693228127

chr14_23025098_23031991 2172.773519 1062.694799 3282.852239 3.089176914 1.627222495 0.007046298 0.693559378

chr5_72671946_72677741 226.3656468 21.01373896 431.7175547 20.54453782 4.36068297 0.007104404 0.697678438

chr16_34793295_34796568 674.8039377 1139.745175 209.8627002 0.184131247 -2.441193624 0.007164765 0.701999732

chr20_58846320_58850206 210.1262712 403.2636572 16.98888526 0.042128481 -4.569060297 0.00719754 0.7036046

chr2_143449384_143456227 1067.076749 420.2747792 1713.878718 4.077995643 2.027860234 0.007240769 0.704620367

chr7_8752818_8757499 634.7090959 190.1243049 1079.293887 5.676780186 2.505072879 0.007227103 0.704620367

chr11_27739205_27741556 225.1334687 429.2806674 20.98627002 0.048887061 -4.354403524 0.00732517 0.706426254

chr17_28993869_28996109 243.1419748 460.3009487 25.98300098 0.056447855 -4.146937442 0.007324627 0.706426254

chr19_4692045_4698944 1071.575769 423.2767419 1719.874796 4.063239543 2.022630419 0.007297113 0.706426254

chr3_77087512_77089762 183.112531 355.2322539 10.99280811 0.030945411 -5.014130705 0.007290387 0.706426254

chr10_43473161_43479351 486.2604048 120.0785083 852.4423013 7.099041394 2.827624226 0.007391122 0.707131114

chr10_73722982_73726225 552.7627081 954.6241414 150.9012749 0.158074019 -2.661327826 0.00736146 0.707131114

chr12_15052253_15059873 1168.951198 1859.215571 478.6868257 0.257467092 -1.95754005 0.007373129 0.707131114

chr3_77873628_77875856 217.1295428 415.271508 18.98757764 0.045723285 -4.450927122 0.007398389 0.707131114

chr1_19969086_19975739 1095.569888 438.2865555 1752.85322 3.999331483 1.999758863 0.007478164 0.713167579

chr1_65412758_65417392 873.6358965 317.2073929 1430.0644 4.508294674 2.172581817 0.007651984 0.719240791

chr11_72245169_72250488 435.2793606 98.06411515 772.494606 7.877444311 2.97772765 0.007560911 0.719240791

chr14_36399141_36404023 338.8169462 59.03859994 618.5952925 10.47781101 3.38926544 0.007697042 0.719240791

chr15_71940503_71944964 443.2767462 102.0667321 784.4867603 7.686018198 2.942236392 0.007709443 0.719240791

chr15_71388484_71390383 556.2630359 958.6267583 153.8993135 0.160541433 -2.638982415 0.007653725 0.719240791

chr3_64169885_64176126 596.2231491 173.1131829 1019.333115 5.888246628 2.5578381 0.007616775 0.719240791

chr6_106984689_106992035 674.8019756 1136.743212 212.8607388 0.187254902 -2.416924609 0.007701219 0.719240791

chr6_41574597_41580799 784.6626923 269.1759896 1300.149395 4.830109094 2.272055775 0.007580862 0.719240791

chr8_73044503_73049470 372.3035459 72.04710501 672.5599869 9.335003631 3.222650584 0.007666802 0.719240791

chr9_95378988_95383138 386.7018486 695.4546942 77.94900294 0.11208351 -3.157354058 0.007686704 0.719240791

chr5_71218189_71223998 960.6104082 365.2387962 1555.98202 4.260177276 2.090913466 0.007727119 0.719326075

chr5_28504626_28507915 381.1995583 686.4488061 75.95031055 0.110642352 -3.176024362 0.00777324 0.722053267

chr1_47076536_47080687 1113.066948 451.2950605 1774.838836 3.932768141 1.975545134 0.007843398 0.726996588

chr10_48701559_48704577 243.8581293 27.01766438 460.6985943 17.05175502 4.091848329 0.0078757 0.728417427

chr1_222623672_222630068 450.2747856 106.069349 794.4802222 7.490196078 2.905003486 0.00797351 0.735877846

chrX_51238819_51240393 159.0994454 311.2034675 6.99542334 0.022478616 -5.475302958 0.008015935 0.738205651

chr15_36659872_36665635 354.1884342 642.4200197 65.95684864 0.102669354 -3.283922477 0.008083805 0.7428619

chr15_71361554_71366841 636.788233 1077.704612 195.8718535 0.181749109 -2.4599798 0.008143139 0.745123242

chr17_1430856_1436936 849.1453745 307.2008505 1391.089899 4.528274893 2.178961541 0.008132863 0.745123242

chr12_38530737_38533551 340.6828721 620.4056265 60.96011768 0.098258486 -3.347274177 0.008193422 0.748129145

chr13_110130116_110135807 436.2806688 101.0660779 771.4952598 7.633572769 2.932358445 0.008332201 0.750721986

chr14_67903724_67910459 2096.192731 3156.063461 1036.322001 0.32835905 -1.606653876 0.008334977 0.750721986

chr14_77014702_77019188 399.2943954 85.05561008 713.5331807 8.389019608 3.068502218 0.008325396 0.750721986

chr15_21986871_21988631 372.6953053 671.4389925 73.95161817 0.110138998 -3.1826027 0.008305197 0.750721986

chr17_61508677_61514634 1877.863057 904.5914296 2851.134684 3.151847996 1.65619796 0.008328154 0.750721986

chr4_184239523_184246887 678.3009953 1138.744521 217.8574697 0.191313737 -2.385987627 0.008306756 0.750721986

chr8_33100644_33104835 282.1583337 524.3428198 39.97384766 0.076236092 -3.713382016 0.008344271 0.750721986

chr10_47053141_47059998 1313.014669 571.3735689 2054.65577 3.595993727 1.846390504 0.008447101 0.755223661

chr22_22137328_22140589 278.8430943 39.02551521 518.6606734 13.29029663 3.7323014 0.008443496 0.755223661

chr22_43280222_43285555 477.7659622 120.0785083 835.4534161 6.957559913 2.798581427 0.008426342 0.755223661

chr1_60946278_60951981 386.2996248 80.0523389 692.5469107 8.651176471 3.112896338 0.008487127 0.757224615

chr8_101899883_101902564 866.6417812 319.2087014 1414.074861 4.429938329 2.147286614 0.008521361 0.758701643

chr1_174543718_174546719 218.6292162 416.2721623 20.98627002 0.050414781 -4.310009405 0.00874482 0.765885631

chr12_65711503_65717735 373.3048542 75.04906772 671.5606407 8.948287582 3.161611623 0.008628671 0.765885631

chr13_36871053_36873221 268.3479972 36.0235525 500.6724419 13.89847495 3.796854682 0.008741402 0.765885631

chr15_63669947_63675379 771.3284841 1273.832843 268.8241255 0.211035637 -2.244441451 0.008705515 0.765885631

chr17_38644641_38649027 1213.543752 516.3375859 1910.749918 3.700582662 1.887752443 0.008742026 0.765885631

chr20_46956356_46963900 666.7960877 1119.73209 213.860085 0.190992191 -2.388414444 0.00869044 0.765885631

chr20_46616835_46623309 2995.916926 4398.876023 1592.957829 0.362128376 -1.465426867 0.008686217 0.765885631

chr6_12233011_12237055 345.8156397 64.04187112 627.5894083 9.799673203 3.292733639 0.008651095 0.765885631

chr5_56642989_56647008 249.3564954 30.01962709 468.6933638 15.6128976 3.964666407 0.008795302 0.768738044

chr13_34216009_34218743 1025.402443 1641.072947 409.7319385 0.249673203 -2.00188711 0.008858439 0.769493421

chr2_152848174_152852522 255.3538806 32.02093556 478.6868257 14.94918301 3.901994736 0.008875667 0.769493421

chr3_46008353_46010623 809.6600816 290.1897285 1329.130435 4.580211855 2.195414331 0.008840217 0.769493421

chr3_194872721_194879357 915.6287112 348.2276742 1483.029748 4.258793479 2.090444771 0.008844319 0.769493421

chr15_65655499_65659739 976.388044 1570.026497 382.7495913 0.243785434 -2.036316165 0.008943301 0.770686262

chr3_3840051_3843154 532.7515855 917.5999346 147.9032363 0.161184881 -2.633211668 0.008941332 0.770686262

chr8_77592757_77595153 261.1485189 488.3192673 33.97777051 0.069581056 -3.845161606 0.0089378 0.770686262

chr2_96360898_96365856 368.8071423 74.04841348 663.5658712 8.961243597 3.163698956 0.008962108 0.770759234

chr11_61111182_61115986 2263.769614 1147.750409 3379.78882 2.944707136 1.558124159 0.009008361 0.770925193

chr22_36789988_36794359 1026.097669 411.2688911 1640.926446 3.989911423 1.996356719 0.009018918 0.770925193

chr8_129087849_129090986 671.2964156 1124.735362 217.8574697 0.193696648 -2.368129105 0.009035894 0.770925193

chr8_144249742_144254170 401.794723 88.05757279 715.5318731 8.125727867 3.022497049 0.009010554 0.770925193

chr10_133109073_133111248 149.5938842 293.1916912 5.996077149 0.020451047 -5.611681463 0.00909406 0.773298411

chr11_1049455_1057120 1442.483631 653.4272163 2231.540046 3.41513177 1.771941245 0.009099748 0.773298411

chr11_22213809_22215776 209.6246361 400.2616945 18.98757764 0.047437908 -4.397815786 0.009125327 0.773939504

chr3_183627733_183633567 1547.456184 716.4684332 2378.443936 3.319677219 1.731042971 0.009175152 0.776630494

chr3_48883975_48886005 227.1328151 430.2813216 23.9843086 0.055740994 -4.165117458 0.009271021 0.783200511

chr16_51199883_51202308 180.1099143 348.2276742 11.9921543 0.034437683 -4.859868105 0.009302477 0.784313932

chr18_11497599_11502802 336.6796011 611.3997383 61.95946387 0.101340351 -3.302719369 0.009322348 0.784448156

chr1_177879924_177885537 466.2276995 814.5325483 117.9228506 0.144773651 -2.788129044 0.009368017 0.785211828

chr8_142228683_142234392 1828.883975 887.5803076 2770.187643 3.121055773 1.642034138 0.009367633 0.785211828

chr10_6621704_6623295 170.1046799 330.215898 9.993461915 0.030263418 -5.046281229 0.009478476 0.790290529

chr15_102150206_102158311 1008.104859 404.2643114 1611.945407 3.987355206 1.995432131 0.009480413 0.790290529

chr8_50707069_50711804 258.6468833 483.3159961 33.97777051 0.070301357 -3.830303647 0.009483855 0.790290529

chr12_89775253_89780563 1392.499971 628.4108604 2156.589081 3.43181383 1.778971291 0.009598096 0.796716197

chr2_115511258_115515299 192.115803 369.2414132 14.99019287 0.040597269 -4.62247352 0.009582385 0.796716197

chr8_128046693_128053045 1865.876789 913.5973177 2818.15626 3.08468097 1.625121288 0.009685273 0.80240052

chr6_169310848_169317489 577.2643484 981.6418058 172.8868911 0.176120139 -2.505368208 0.009737472 0.805170706

chr10_27770143_27775357 606.7260944 188.1229964 1025.329192 5.450312891 2.446339054 0.009823641 0.808323861

chr10_129803742_129808461 210.6246363 401.2623487 19.98692383 0.049810115 -4.327417441 0.009843773 0.808323861

chr17_39494323_39501930 1273.966263 1987.299313 560.6332134 0.28210809 -1.825680056 0.009850947 0.808323861

chr7_22893384_22894982 260.1472106 485.3173046 34.9771167 0.072070615 -3.79444503 0.009833408 0.808323861

chr14_64579141_64582468 241.3610719 29.01897285 453.7031709 15.63470814 3.966680383 0.009896625 0.810522256

chr22_25678089_25679644 168.6036986 327.2139353 9.993461915 0.030541065 -5.03310584 0.009953435 0.813622247

chr10_49659702_49665995 2030.836929 1017.665358 3044.008499 2.991168437 1.580709153 0.00998118 0.814339072

chr11_42225780_42228281 320.1720763 583.3814197 56.96273292 0.09764235 -3.356349169 0.010031807 0.816916517

chr12_13527404_13532098 290.8398268 46.03009487 535.6495586 11.63694231 3.540640125 0.010076819 0.818354573

chr12_109746992_109748922 600.7287093 186.1216879 1015.335731 5.455225244 2.447638766 0.010087605 0.818354573

chr15_24437618_24441047 168.1033715 326.213281 9.993461915 0.030634749 -5.028687169 0.010116914 0.819183726

chrY_2496858_2501153 471.7280276 820.5364737 122.9195816 0.149803922 -2.738852704 0.010148291 0.820176821

chr18_37101540_37105747 337.1786202 610.3990841 63.95815626 0.104780885 -3.254552542 0.010182284 0.821377278

chr8_135478100_135482542 389.6985791 693.4533857 85.94377247 0.123935904 -3.012333897 0.01020882 0.821972791

chr10_52946109_52951314 209.1236549 398.260386 19.98692383 0.050185568 -4.316583635 0.010258386 0.824416895

chr14_78662771_78670231 664.7091023 220.143932 1109.274273 5.03885918 2.333097138 0.010302572 0.82642037

chr7_100313858_100319891 455.2218109 794.5194636 115.9241582 0.145904743 -2.776901312 0.010394242 0.832218093

chr19_58663724_58667242 741.8137623 1221.798822 261.8287022 0.21429772 -2.222311593 0.010429734 0.83350469

chr13_23488666_23491129 242.1387045 454.2970233 29.98038574 0.065992917 -3.921545001 0.010468256 0.835028232

chr1_167531910_167537309 1577.956518 747.4887145 2408.424322 3.222020982 1.687965889 0.010510767 0.836863752

chr2_44230497_44234229 257.3545351 35.02289827 479.6861719 13.69635854 3.775720469 0.010534135 0.837171127

chr5_56676838_56681729 394.7999536 89.05822703 700.5416802 7.866108541 2.975650093 0.010576969 0.839021492

chr3_19187589_19189579 188.6135132 362.2368335 14.99019287 0.041382299 -4.594842401 0.010628767 0.841574821

chr12_66284670_66290722 268.3499593 39.02551521 497.6744034 12.75253896 3.672712604 0.010716006 0.845757755

chr13_52781288_52787802 833.8399429 1353.885182 313.7947041 0.231773498 -2.109212485 0.01073932 0.845757755

chr3_189032470_189036737 1119.420122 1762.15211 476.6881333 0.270514748 -1.886220847 0.010740719 0.845757755

chr8_42152255_42158898 1130.42274 1777.161924 483.6835567 0.272166284 -1.877439739 0.010827632 0.849484155

chrX_16450806_16457778 482.230319 834.545633 129.9150049 0.155671541 -2.68342287 0.010815555 0.849484155

chr17_6267279_6270502 217.6272539 412.2695453 22.9849624 0.055752269 -4.164825681 0.010870359 0.851279999

chr21_33735919_33738529 584.236227 181.1184168 987.3540372 5.451428159 2.446634235 0.010996876 0.859619172

chr11_100730728_100734707 1134.076763 487.3186131 1780.834913 3.654354391 1.86961655 0.011054441 0.860296109

chr12_67054525_67059974 542.2499525 160.1046778 924.3952271 5.77369281 2.529494351 0.011064219 0.860296109

chr3_96531792_96534398 529.7456986 905.5920838 153.8993135 0.169943307 -2.556874551 0.011072029 0.860296109

chr6_106052040_106058551 1681.433322 815.5332025 2547.333442 3.123518986 1.643172299 0.011085722 0.860296109

chr18_47269251_47274171 366.3113929 78.05103043 654.5717554 8.386458857 3.068061768 0.011160084 0.862945944

chr2_241532155_241537973 598.7319789 189.1236507 1008.340307 5.331645745 2.414580925 0.011151136 0.862945944

chr6_42243003_42250149 1079.092448 456.2983317 1701.886564 3.729767229 1.899085596 0.011222196 0.866188019

chr10_120527462_120530710 219.6279083 415.271508 23.9843086 0.057755729 -4.113892135 0.011281392 0.868015544

chr6_38777855_38783700 282.8444032 45.02944063 520.6593658 11.56264343 3.531399356 0.011286326 0.868015544

chr10_62605260_62611806 670.7895483 1113.728165 227.8509317 0.204583972 -2.289234973 0.011386912 0.871891018

chr14_64524867_64530493 702.7006081 245.1602879 1160.240928 4.732581032 2.242627208 0.011434888 0.871891018

chr2_38236815_38243126 374.3087786 82.05364737 666.5639097 8.12351347 3.022103837 0.011423875 0.871891018

chr3_14057395_14061746 672.709758 229.1498201 1116.269696 4.871353141 2.284322573 0.011456073 0.871891018

chr7_153214319_153217303 190.1138405 364.238142 15.98953906 0.043898585 -4.50968175 0.01143768 0.871891018

chrX_2546599_2551250 490.7319558 845.5528296 135.911082 0.160736358 -2.637231799 0.011458617 0.871891018

chr8_8297513_8301819 1323.028406 602.3938502 2043.662962 3.392569431 1.762378342 0.011517377 0.874811031

chr1_177552842_177558145 1433.999653 2198.437357 669.5619483 0.304562669 -1.715188979 0.011580686 0.876516989

chr14_32433324_32439648 725.6940728 258.168793 1193.219353 4.621857425 2.208472756 0.011565656 0.876516989

chr13_51083842_51090249 747.1878642 270.1766438 1224.199085 4.53110627 2.179863327 0.011628611 0.877337485

chr9_71446048_71450154 337.8228322 67.04383383 608.6018306 9.077670471 3.182322118 0.011632414 0.877337485

chr1_177317679_177324213 553.2519169 938.6136736 167.8901602 0.178870354 -2.483013799 0.011747814 0.877812166

chr11_76867874_76872066 320.1701143 580.379457 59.96077149 0.103313049 -3.274905604 0.011675317 0.877812166

chr17_43944570_43949830 307.3349251 55.03598299 559.6338672 10.16850862 3.346036194 0.011761435 0.877812166

chr2_208250624_208256814 339.8221786 68.04448806 611.5998692 8.988235294 3.168037892 0.011726153 0.877812166

chr6_78048522_78053700 581.2388424 182.119071 980.3586139 5.383063995 2.428427576 0.011723234 0.877812166

chrX_24669286_24673214 264.3525745 39.02551521 489.6796338 12.5476789 3.649348611 0.011730872 0.877812166

chr10_5989980_5995728 430.2891699 108.0706575 752.5076822 6.963108206 2.799731443 0.011797007 0.878938485

chr16_82788318_82793164 380.8071449 86.05626432 675.5580255 7.850189998 2.972727572 0.011968443 0.890165911

chr21_33718624_33726383 2250.79708 1176.769382 3324.824779 2.825383487 1.498446697 0.011993412 0.890479683

chr19_51545828_51550827 1307.464635 2018.319595 596.6096763 0.295597227 -1.758295357 0.012015586 0.890585279

chr19_44763339_44765182 176.1066433 339.2217861 12.99150049 0.038297954 -4.706588855 0.012057424 0.892145469

chr17_67322315_67324296 515.2388283 880.5757279 149.9019287 0.170231729 -2.554428133 0.012116416 0.894967295

chr15_75962260_75968430 1567.968288 755.4939484 2380.442628 3.150842748 1.655737754 0.012159742 0.896624311

chr5_172958879_172962778 297.6606261 543.3552503 51.96600196 0.095639091 -3.386255779 0.012190268 0.897333378

chr3_180022251_180026493 322.6704418 583.3814197 61.95946387 0.106207469 -3.235042872 0.012252569 0.90037503

chr1_209555689_209558696 166.6017361 322.2106641 10.99280811 0.034116835 -4.873372369 0.012342654 0.905444417

chr14_99529540_99534652 822.1682596 315.2060844 1329.130435 4.216702977 2.076115402 0.012389546 0.907333364

chr1_176865454_176869860 250.1406683 465.3042199 34.9771167 0.075170427 -3.733690999 0.01242933 0.908696232

chr1_228915306_228922166 918.6417923 371.2427216 1466.040863 3.949009038 1.98149067 0.012632861 0.911481278

chr1_38882569_38888527 503.7339207 861.5632974 145.904544 0.169348607 -2.561931978 0.012724343 0.911481278

chr11_7924504_7928916 246.1387054 458.2996402 33.97777051 0.074138768 -3.753628056 0.012743534 0.911481278

chr12_120893877_120898388 306.3362329 56.03663723 556.6358287 9.933426704 3.312291486 0.012633902 0.911481278

chr12_1922627_1927102 923.1408122 374.2446844 1472.03694 3.933354304 1.975760146 0.012717041 0.911481278

chr15_22891296_22894667 1242.945328 1924.258096 561.6325596 0.291869662 -1.776603837 0.012569399 0.911481278

chr22_26325163_26328197 186.6115507 357.2335623 15.98953906 0.044759342 -4.481667374 0.012661709 0.911481278

chr3_170975458_170982093 582.2591173 978.6398431 185.8783916 0.189935442 -2.396418953 0.012699939 0.911481278

chr5_43771365_43774144 923.1401582 373.2440301 1473.036286 3.946576951 1.980601879 0.012514694 0.911481278

chr7_153118732_153121674 269.1485206 496.3245012 41.97254004 0.08456673 -3.563765997 0.012671811 0.911481278

chr8_8745078_8752529 1962.872231 1003.656199 2922.088264 2.911443447 1.541734596 0.012650104 0.911481278

chrX_55514381_55516822 293.6586632 536.3506706 50.96665577 0.095024876 -3.395550958 0.012549821 0.911481278

chrX_132006731_132013054 868.155843 342.2237488 1394.087937 4.073615411 2.026309782 0.012604595 0.911481278

chr7_158299710_158302281 292.658009 534.3493622 50.96665577 0.095380774 -3.390157699 0.012812981 0.914923621

chr17_38761973_38764626 618.7699165 1031.674518 205.8653154 0.199544829 -2.3252152 0.012939616 0.922431283

chr6_21431436_21437496 965.8694021 1531.000981 400.7378228 0.2617489 -1.933744621 0.013073408 0.930423372

chr12_109562025_109566007 238.8646685 32.02093556 445.7084014 13.91928105 3.79901279 0.013194658 0.937229082

chr3_66500259_66502043 425.2930929 109.0713118 741.5148741 6.798440967 2.765203942 0.013212713 0.937229082

chr11_114313669_114320223 499.2682559 145.0948643 853.4416475 5.881956277 2.55629606 0.013246788 0.937748793

chr6_84417947_84419517 181.1086064 347.22702 14.99019287 0.043171159 -4.533788367 0.013263743 0.937748793

chr15_101670759_101675257 381.30878 89.05822703 673.5593331 7.563134317 2.918984241 0.013325581 0.940571239

chr4_124424053_124432077 1118.590821 493.3225385 1743.859104 3.534926885 1.821680375 0.013576182 0.956686141

chr18_49140515_49144865 202.1184213 383.2505725 20.98627002 0.054758614 -4.190770269 0.013676446 0.960596871

chr3_87365663_87374361 1612.464701 794.5194636 2430.409938 3.058968407 1.613045207 0.013667893 0.960596871

chr11_60633477_60635916 922.6444092 379.2479555 1466.040863 3.865652646 1.950712008 0.013830894 0.966544466

chr15_24681995_24684122 194.6148225 370.2420674 18.98757764 0.051284225 -4.285341057 0.013851214 0.966544466

chr8_110628709_110635944 953.6359136 397.2597318 1510.012095 3.801070117 1.926405638 0.013824133 0.966544466

chrX_138276338_138277781 183.609588 351.2296369 15.98953906 0.045524459 -4.45721433 0.01381252 0.966544466

chr19_16111768_16118282 536.74243 907.5933923 165.8914678 0.182781705 -2.451806419 0.013901918 0.967318665

chr7_30777711_30785778 1831.411772 932.6097482 2730.213795 2.927498668 1.549668514 0.013907389 0.967318665

chr21_14796956_14801432 332.672406 596.3899248 68.95488721 0.115620476 -3.112531173 0.013967631 0.96993673

chr16_75084569_75090194 1567.521591 2365.546614 769.4965675 0.325293344 -1.620186794 0.014048948 0.972948647

chrX_30920928_30924660 313.1648806 565.3696435 60.96011768 0.107823472 -3.213256829 0.014056348 0.972948647

chr1_66603491_66608068 269.8522487 44.02878639 495.675711 11.25799168 3.492877582 0.014102305 0.974557835

chr12_7305908_7310797 498.2296684 849.5554466 146.9038901 0.172918543 -2.531835508 0.014142094 0.974601955

chr3_139194074_139200414 456.2839432 126.0824338 786.4854527 6.237866999 2.641052792 0.014148363 0.974601955

chr1_154304619_154308636 1143.412279 1774.159961 512.6645962 0.288961879 -1.791048915 0.014403318 0.977364286

chr10_3109079_3112133 726.2983895 1182.773307 269.8234717 0.228127799 -2.132085833 0.014469606 0.977364286

chr10_79979622_79984139 659.2787545 1085.709846 232.8476626 0.214465829 -2.221180292 0.014492618 0.977364286

chr11_8102271_8103801 142.0883234 277.1812234 6.99542334 0.025237724 -5.308274354 0.014560837 0.977364286

chr14_21962459_21965430 1099.098338 485.3173046 1712.879372 3.529400984 1.819423347 0.014251623 0.977364286

chr14_23321572_23324896 385.3087809 93.06084397 677.5567178 7.280792747 2.864095542 0.014457325 0.977364286

chr15_23084817_23088230 1006.876605 1583.035002 430.7182085 0.272083819 -1.877876935 0.014284515 0.977364286

chr15_22832097_22835087 1107.902788 1724.127249 491.6783262 0.285175196 -1.81007959 0.014461881 0.977364286

chr15_47338176_47341470 142.0883234 277.1812234 6.99542334 0.025237724 -5.308274354 0.014560837 0.977364286

chr17_35085211_35088803 995.8733327 1567.024534 424.7221314 0.271037321 -1.883436576 0.014388069 0.977364286

chr19_17216982_17220139 977.1310134 413.2701996 1540.991827 3.728775578 1.898701969 0.014300605 0.977364286

chr2_70201582_70207753 947.860242 1498.980046 396.740438 0.264673595 -1.917713821 0.014439885 0.977364286

chr2_203670455_203673982 371.8136832 87.05691855 656.5704478 7.541852603 2.914918955 0.014529718 0.977364286

chr5_111979955_111986930 503.2689108 150.0981354 856.4396861 5.705864924 2.512445594 0.014398078 0.977364286

chr7_35766832_35774565 959.6365689 404.2643114 1515.008826 3.747570051 1.905955446 0.01457563 0.977364286

chr7_68260998_68268732 926.145391 384.2512267 1468.039555 3.820520153 1.93376907 0.014486967 0.977364286

chr8_21197993_21201789 273.8509415 46.03009487 501.6717881 10.89877806 3.446094488 0.014508482 0.977364286

chr1_61957906_61964534 534.7404675 902.5901211 166.890814 0.184902106 -2.43516644 0.014651667 0.980293072

chr8_124182254_124187471 1335.038873 630.4121688 2039.665577 3.235447661 1.693965339 0.014664992 0.980293072

chr18_1651985_1655826 303.3395024 58.0379457 548.6410591 9.453144016 3.240794234 0.014814965 0.985977364

chr19_51572931_51574295 377.1877851 664.4344129 89.94115723 0.135364989 -2.885073445 0.014775306 0.985977364

chr8_8890638_8892370 396.8051862 99.06476939 694.5456031 7.011025286 2.809625438 0.014818954 0.985977364

chr2_38738897_38743956 473.2793687 136.0889761 810.4697613 5.955440215 2.574208153 0.014842909 0.986042501

chr1_93396247_93402588 483.7764279 142.0929015 825.4599542 5.809297616 2.538363742 0.015159267 0.98893331

chr10_54166582_54174391 818.6770881 325.2126268 1312.141549 4.034718954 2.012468183 0.015277537 0.98893331

chr10_72089731_72095800 575.7476706 190.1243049 961.3710362 5.056539388 2.338150366 0.015278173 0.98893331

chr10_108331281_108333483 141.0876692 275.179915 6.99542334 0.025421272 -5.297819996 0.015079082 0.98893331

chr12_49739330_49743809 1013.6235 438.2865555 1588.960444 3.625391709 1.858136881 0.015037583 0.98893331

chr12_3434212_3439283 374.3133567 89.05822703 659.5684864 7.406036572 2.888701674 0.015013287 0.98893331

chr17_6468981_6473515 1115.097034 499.3264639 1730.867604 3.466404705 1.7934401 0.015113157 0.98893331

chr17_66021829_66027714 487.2754476 144.09421 830.4566851 5.76328976 2.526892556 0.015258964 0.98893331

chr19_12074805_12077353 471.2191982 806.5273144 135.911082 0.168513923 -2.569060297 0.015212605 0.98893331

chr21_30332072_30336105 304.3395026 59.03859994 549.6404053 9.309848233 3.21875765 0.015244351 0.98893331

chr21_46197092_46199994 755.1944061 288.18842 1222.200392 4.240976761 2.084396578 0.015060617 0.98893331

chr5_173571703_173576207 393.6933478 689.4507688 97.93592677 0.142049195 -2.815537438 0.014986293 0.98893331

chr5_134468902_134472693 1335.539854 632.4134773 2038.666231 3.223628692 1.688685579 0.014948852 0.98893331

chr7_55274797_55278914 393.3068205 98.06411515 688.5495259 7.021421902 2.811763219 0.015151064 0.98893331

chr7_69117526_69122780 1689.452291 852.5574093 2526.347172 2.963257541 1.567184018 0.015051483 0.98893331

chr8_35572954_35576796 155.5951935 301.1969251 9.993461915 0.033179163 -4.913578691 0.015183669 0.98893331

chr8_74095425_74101317 1209.572528 556.3637554 1862.781301 3.348135609 1.743357962 0.015097142 0.98893331

chr19_45218855_45223163 550.244068 923.6038601 176.8842759 0.191515306 -2.384468397 0.015370326 0.993399894

chr1_234878374_234884668 1089.051901 1168.764148 1009.339653 0.863595667 -0.211572091 0.770705462 1

chr1_1043230_1048619 335.8954283 176.1151456 495.675711 2.81449792 1.492877582 0.208745788 1

chr1_2096973_2098826 121.4931588 111.0726202 131.9136973 1.187634694 0.248091143 0.903269794 1

chr1_15726236_15728666 139.0503895 216.141315 61.95946387 0.286661825 -1.802578301 0.335236744 1

chr1_19921753_19924945 733.0217395 766.501145 699.542334 0.91264356 -0.13187658 0.875941451 1

chr1_24116670_24120175 613.4991502 612.4003926 614.5979078 1.003588363 0.005167648 0.99748217 1

chr1_47183291_47188960 1515.647806 977.6391888 2053.656424 2.100628173 1.070820817 0.103276383 1

chr1_88077708_88082768 353.4016452 203.13281 503.6704805 2.479513185 1.310056897 0.256665025 1

chr1_111148623_111150755 242.4251663 128.0837422 356.7665904 2.785416667 1.477893154 0.287113707 1

chr1_113246110_113250972 1979.321548 1707.116127 2251.526969 1.318906742 0.399342557 0.509040808 1

chr1_155185728_155189914 268.4389063 175.1144913 361.7633213 2.065867414 1.046747666 0.423949104 1

chr1_164626411_164634059 3227.208669 2782.819431 3671.597908 1.319380577 0.399860772 0.459041608 1

chr1_222297331_222298839 46.47875422 14.00915931 78.94834913 5.635480859 2.494538717 0.503450051 1

chr1_521231_526745 1207.624195 1397.913968 1017.334423 0.727751812 -0.458481569 0.512532811 1

chr1_1308968_1312065 879.5750737 994.6503108 764.4998365 0.76861167 -0.379673214 0.627177307 1

chr1_3273138_3279251 990.5358561 1045.683677 935.3880352 0.894522938 -0.160809617 0.830656427 1

chr1_3406300_3413487 1356.318145 1078.705267 1633.931023 1.514714977 0.599046348 0.374935673 1

chr1_6684582_6686685 533.0210427 565.3696435 500.6724419 0.885566545 -0.175327374 0.855177624 1

chr1_9170896_9175607 493.5069728 504.3297351 482.6842105 0.95708061 -0.063287654 0.950719937 1

chr1_9958989_9962575 481.4611886 422.2760877 540.6462896 1.280314717 0.356498486 0.72060081 1

chr1_10091881_10096209 692.5292521 737.4821721 647.5763321 0.878090829 -0.187557916 0.827430618 1

chr1_10612887_10618879 1316.019902 1346.880602 1285.159202 0.954174557 -0.067674877 0.921762409 1

chr1_11138396_11143680 986.8909894 820.5364737 1153.245505 1.405477443 0.4910603 0.512614895 1

chr1_11401993_11403499 270.9013006 120.0785083 421.7240928 3.512069717 1.812321484 0.172064633 1

chr1_11795404_11799290 697.4730072 656.429179 738.5168355 1.125051809 0.16999144 0.843099807 1

chr1_14154100_14161951 1738.122674 1926.259405 1549.985943 0.804661064 -0.313546869 0.616733773 1

chr1_14810990_14814971 306.5245914 344.2250573 268.8241255 0.780954552 -0.356689502 0.771286577 1

chr1_15334377_15336438 141.9954522 135.0883219 148.9025825 1.102260954 0.140465814 0.941759294 1

chr1_15466039_15467401 65.99478194 58.0379457 73.95161817 1.274194275 0.349585261 0.906380321 1

chr1_18217651_18221220 266.9752043 229.1498201 304.8005884 1.330136713 0.411574535 0.753571996 1

chr1_18608427_18613362 472.9798265 442.2891724 503.6704805 1.138780942 0.187490255 0.852939395 1

chr1_19005271_19008171 273.9941724 265.1733726 282.8149722 1.066528549 0.092922584 0.945210168 1

chr1_20574478_20580151 800.0511848 878.5744194 721.5279503 0.82124853 -0.284109212 0.725981633 1

chr1_22281185_22289002 1334.424092 1218.79686 1450.051324 1.189739957 0.250646277 0.711733138 1

chr1_24901625_24903723 172.9791083 141.0922473 204.8659693 1.452000185 0.538041638 0.742279571 1

chr1_25557898_25560443 699.5246754 737.4821721 661.5671788 0.897061927 -0.156720512 0.855180783 1

chr1_25820452_25821866 70.99412898 62.04056265 79.94769532 1.288635884 0.365844675 0.896785847 1

chr1_26082040_26085897 216.4781366 183.1197252 249.8365479 1.364334441 0.448197337 0.758302017 1

chr1_26605304_26608295 568.0033916 573.3748774 562.6319058 0.981263617 -0.027287326 0.978560109 1

chr1_27915467_27918651 233.472908 192.1256134 274.8202027 1.43041939 0.516438198 0.712130503 1

chr1_28572521_28578554 1823.388225 1653.080798 1993.695652 1.206048521 0.27028795 0.662001358 1

chr1_28830929_28837577 2307.308538 2779.817468 1834.799608 0.660043197 -0.59936765 0.303569561 1

chr1_30931412_30936687 573.7502862 956.6254499 190.8751226 0.199529631 -2.325325089 0.01594537 1

chr1_31326157_31327557 70.01636553 95.06215244 44.97057862 0.473065015 -1.079889622 0.696315239 1

chr1_32374516_32380393 863.157804 1104.722277 621.5933311 0.562669319 -0.829640796 0.293374146 1

chr1_33158608_33162925 755.7719079 1171.766111 339.7777051 0.289970585 -1.786021534 0.035337418 1

chr1_33684100_33689307 1584.193275 1880.22931 1288.157241 0.685106457 -0.545599913 0.396809077 1

chr1_33812063_33817725 1733.774733 2154.408571 1313.140896 0.609513401 -0.714270153 0.255826119 1

chr1_38271756_38275393 1796.30385 2261.478574 1331.129127 0.588610099 -0.7646158 0.219590398 1

chr1_38401577_38407690 1519.781228 1950.275106 1089.287349 0.558530099 -0.840293065 0.198904186 1

chr1_39189288_39192507 141.0700106 248.1622506 33.97777051 0.136917563 -2.868620579 0.136785801 1

chr1_39582349_39584638 147.0000314 147.0961727 146.9038901 0.99869281 -0.00188711 1 1

chr1_39713123_39716134 227.5540055 310.2028132 144.9051978 0.467130508 -1.098102425 0.439925633 1

chr1_41156215_41160382 1457.135694 1665.088649 1249.182739 0.75021996 -0.414614448 0.529665504 1

chr1_41417129_41418914 141.0163807 166.1086032 115.9241582 0.697881723 -0.518945546 0.7768837 1

chr1_43122600_43125793 1231.24356 1604.048741 858.4383785 0.535169759 -0.901931499 0.196926775 1

chr1_43777854_43779804 94.51996789 125.0817795 63.95815626 0.511330719 -0.967671394 0.673730592 1

chr1_46267828_46271521 765.626717 959.6274126 571.6260215 0.595674961 -0.747402778 0.364970713 1

chr1_53737703_53741281 298.972595 257.1681387 340.7770513 1.325113807 0.40611627 0.743358565 1

chr1_54394234_54395467 59.98824043 42.02747792 77.94900294 1.854715219 0.891197687 0.771065476 1

chr1_55180664_55182652 382.4585514 319.2087014 445.7084014 1.396291516 0.481600177 0.662493017 1

chr1_55442185_55447377 582.3781494 396.2590776 768.4972213 1.939380735 0.955596058 0.3001595 1

chr1_56878778_56885786 840.216007 406.2656199 1274.166394 3.136288998 1.649058505 0.042307593 1

chr1_59111119_59113623 193.4964443 188.1229964 198.8698921 1.057126964 0.080148659 0.961744444 1

chr1_67894240_67897831 1495.961733 1437.940137 1553.983328 1.080700988 0.111967409 0.864786329 1

chr1_68025980_68028868 275.9071879 134.0876677 417.726708 3.115325334 1.639382832 0.210357987 1

chr1_68678418_68684666 518.9575995 454.2970233 583.6181758 1.284662117 0.361388962 0.707999099 1

chr1_73792226_73793904 56.96992716 11.0071966 102.9326577 9.351396316 3.225181799 0.335306978 1

chr1_90308304_90310137 213.6174427 393.2571148 33.97777051 0.086400905 -3.532809771 0.02720147 1

chr1_91485793_91488880 1166.928307 1057.691528 1276.165087 1.206556972 0.270896039 0.70215425 1

chr1_101446586_101450500 307.9510142 233.152437 382.7495913 1.641628096 0.715127328 0.558069015 1

chr1_105775007_105776654 69.48202925 42.02747792 96.93658058 2.306504824 1.20570831 0.664736896 1

chr1_108134397_108139587 602.4317835 498.3258097 706.5377574 1.417822926 0.503677363 0.57812019 1

chr1_109966803_109970565 727.9412937 638.4174027 817.4651846 1.280455672 0.35665731 0.671127244 1

chr1_115735698_115738893 180.9352905 82.05364737 279.8169336 3.410170572 1.769843903 0.275683315 1

chr1_116651713_116656734 568.8156874 287.1877658 850.443609 2.961280772 1.566221285 0.097112675 1

chr1_116781879_116787117 589.7941091 275.179915 904.4083033 3.286607249 1.716599064 0.06613064 1

chr1_116914597_116919676 1074.380871 892.5835787 1256.178163 1.407350743 0.492981925 0.498387202 1

chr1_118225638_118230288 242.3924652 78.05103043 406.7338999 5.21112787 2.381595656 0.095577688 1

chr1_118619757_118621028 87.97843608 55.03598299 120.9208892 2.197124183 1.135616414 0.636347322 1

chr1_119930093_119931308 117.0111434 134.0876677 99.93461915 0.745293142 -0.42412011 0.835855473 1

chr1_144045555_144050880 416.9987811 415.271508 418.7260542 1.008318765 0.011951798 0.993551497 1

chr1_144964683_144968512 279.0412631 342.2237488 215.8587774 0.630753354 -0.664852122 0.603497362 1

chr1_145095553_145098265 484.4500709 408.2669284 560.6332134 1.373202614 0.457544509 0.644987598 1

chr1_148240104_148242943 435.55405 518.3388944 352.7692056 0.680576375 -0.555171024 0.593589986 1

chr1_148765427_148768488 248.0353702 302.1975793 193.8731612 0.641544388 -0.640379007 0.636880936 1

chr1_149809365_149816969 2538.462283 3246.122342 1830.802223 0.56399668 -0.826241426 0.148073704 1

chr1_150206685_150209490 436.54751 509.3330063 363.7620137 0.714192894 -0.485614315 0.640358282 1

chr1_150333001_150339968 3466.665882 3721.433105 3211.898659 0.863081122 -0.212431928 0.689883401 1

chr1_150469425_150470761 49.47679279 14.00915931 84.94442628 6.063492063 2.600148905 0.468465112 1

chr1_150599455_150603584 824.0354934 878.5744194 769.4965675 0.87584677 -0.191249603 0.811902745 1

chr1_151253105_151257095 1245.634668 1451.949297 1039.320039 0.715810147 -0.482351101 0.486481713 1

chr1_151519125_151523509 806.5096558 821.537128 791.4821837 0.963416207 -0.053768901 0.948349455 1

chr1_153484891_153489822 604.4533667 533.3487079 675.5580255 1.266634784 0.341000604 0.706392515 1

chr1_153746863_153750186 1319.594134 1463.957148 1175.231121 0.802776996 -0.316928819 0.641328601 1

chr1_159905679_159909053 865.2853388 537.3513249 1193.219353 2.220557199 1.150921734 0.146848565 1

chr1_161085498_161090840 1493.894368 1332.871443 1654.917293 1.241618089 0.312221481 0.633455246 1

chr1_162659667_162662429 167.0190024 196.1282303 137.9097744 0.703161265 -0.508072497 0.760740299 1

chr1_167638447_167642231 736.2427997 343.224403 1129.261196 3.290154157 1.718155182 0.044592611 1

chr1_167901546_167908131 2304.43738 2209.444554 2399.430206 1.08598797 0.119008122 0.838305632 1

chr1_178519352_178522805 312.006607 322.2106641 301.8025498 0.936662201 -0.094399248 0.940326907 1

chr1_179699070_179702596 194.9039004 48.03140334 341.7763975 7.115686275 2.831002905 0.081191839 1

chr1_181010354_181015055 600.9340723 500.3271181 701.5410264 1.402164706 0.487655826 0.590649406 1

chr1_182053431_182059085 446.5121949 465.3042199 427.72017 0.919226931 -0.121507029 0.907673969 1

chr1_182582833_182588760 974.2506989 593.3879621 1355.113436 2.283688787 1.191366059 0.117377126 1

chr1_184941536_184944310 727.9177489 602.3938502 853.4416475 1.416750266 0.502585473 0.549489506 1

chr1_186120845_186124372 165.0739398 278.1818777 51.96600196 0.186805849 -2.420388464 0.164491007 1

chr1_190445387_190449299 700.8987765 546.357213 855.4403399 1.565716201 0.646822736 0.448207361 1

chr1_197787263_197788800 58.98169999 31.02028132 86.94311866 2.802783049 1.486860076 0.631745121 1

chr1_201457384_201460382 393.9608429 334.2185149 453.7031709 1.357504599 0.440957085 0.685512493 1

chr1_202111631_202115179 1304.648415 1532.001636 1077.295194 0.703194546 -0.508004214 0.45700673 1

chr1_205781134_205783601 482.9857148 461.3016029 504.6698267 1.094012732 0.129629528 0.897789569 1

chr1_207223004_207227624 1128.016592 1153.754334 1102.278849 0.955384362 -0.065846832 0.927804371 1

chr1_208403432_208408599 644.5665211 746.4880602 542.644982 0.726930558 -0.460110542 0.601540113 1

chr1_210501440_210503693 803.1329383 1006.658162 599.6077149 0.595641835 -0.747483009 0.356208789 1

chr1_211284438_211289628 229.117773 409.2675826 48.96796338 0.119647794 -3.063134298 0.042689251 1

chr1_221642555_221645171 128.5670648 231.1511286 25.98300098 0.112406983 -3.153196433 0.124626177 1

chr1_223476326_223478058 81.53893176 141.0922473 21.98561621 0.15582441 -2.682006843 0.305588668 1

chr1_223870778_223872385 139.0503895 216.141315 61.95946387 0.286661825 -1.802578301 0.335236744 1

chr1_229113161_229115794 213.9261412 101.0660779 326.7862046 3.233391574 1.693048233 0.25471763 1

chr1_231472154_231475734 942.59994 1095.716389 789.4834913 0.720518101 -0.472893421 0.535015177 1

chr1_235665604_235669886 2002.945491 1920.255479 2085.635502 1.086123968 0.119188779 0.843709269 1

chr1_235798456_235800649 186.9876136 168.1099117 205.8653154 1.224587613 0.292295995 0.8537495 1

chr1_246544744_246546760 126.5010081 128.0837422 124.9182739 0.975285948 -0.036102825 0.989623769 1

chr1_247332428_247335884 1140.000898 1141.746484 1138.255312 0.996942253 -0.004418154 0.996457491 1

chr1_249166186_249168848 684.4409574 594.3886163 774.4932984 1.303008296 0.38184627 0.657209557 1

chr1_439603_445251 1131.46002 1070.700033 1192.220006 1.113495816 0.155096137 0.828964425 1

chr1_456405_464620 1870.139707 2084.362774 1655.916639 0.794447425 -0.331976346 0.58868809 1

chr1_538261_543193 1285.482943 1259.823683 1311.142203 1.040734684 0.057602327 0.934088031 1

chr1_865464_869880 741.5593476 832.5443246 650.5743707 0.78142911 -0.355813095 0.669624252 1

chr1_900836_903786 716.0819059 841.5502127 590.6135992 0.701816232 -0.51083478 0.545536737 1

chr1_931248_939004 2972.825358 3471.269546 2474.38117 0.712817353 -0.488395636 0.374146593 1

chr1_947934_950806 246.9627736 190.1243049 303.8012422 1.597908497 0.676184796 0.618916473 1

chr1_1012029_1017271 1353.637307 1564.022571 1143.252043 0.730969018 -0.452117836 0.502930309 1

chr1_1076850_1083374 1899.39609 1741.138371 2057.653808 1.181786492 0.240969414 0.693566694 1

chr1_1259131_1262606 1054.494012 1045.683677 1063.304348 1.016850862 0.024108099 0.975114886 1

chr1_1341367_1343996 979.0721518 1089.712463 868.4318404 0.796936687 -0.327462981 0.663310314 1

chr1_1405582_1409038 801.5495501 877.5737652 725.525335 0.82674 -0.274494404 0.734760768 1

chr1_1589186_1591929 687.0145354 709.4638535 664.5652173 0.936714695 -0.094318396 0.913931009 1

chr1_1621795_1625767 1335.550972 1413.924436 1257.177509 0.889140521 -0.169516653 0.802930334 1

chr1_1654343_1657159 625.0243325 662.4331044 587.6155606 0.887056454 -0.172902172 0.847320958 1

chr1_1669155_1672121 154.9287447 46.03009487 263.8273946 5.731628303 2.518945054 0.163456737 1

chr1_1849191_1852615 393.4716342 350.2289827 436.7142857 1.246939309 0.318391248 0.770617514 1

chr1_1931101_1936289 671.8824197 492.3218842 851.4429552 1.729443647 0.790308005 0.362833795 1

chr1_1980369_1983631 962.5672431 1065.696762 859.4377247 0.806456166 -0.310331975 0.681790031 1

chr1_2143081_2146667 387.8529279 163.1066405 612.5992154 3.755820201 1.909128 0.089866448 1

chr1_2159754_2164266 1150.571862 1260.824338 1040.319385 0.825110489 -0.277340775 0.69676313 1

chr1_2177513_2183731 584.9046378 439.2872097 730.522066 1.6629714 0.733763357 0.424101112 1

chr1_2242065_2248495 729.4789003 697.4560027 761.5017979 1.091827721 0.126745232 0.881032044 1

chr1_2342692_2349727 1864.478489 1832.197907 1896.759071 1.035237004 0.049961092 0.93597615 1

chr1_2422062_2428349 485.3003 180.1177625 790.4828375 4.388700073 2.133793679 0.037889589 1

chr1_2472955_2481393 3072.395687 2149.405299 3995.386074 1.858833266 0.894397369 0.103019074 1

chr1_2522839_2526897 700.9288615 592.3873079 809.4704151 1.36645469 0.450437623 0.597104693 1

chr1_2781006_2786040 504.5390222 564.3689892 444.7090552 0.78797571 -0.343776936 0.724910978 1

chr1_3815677_3818684 1116.583627 1244.81387 988.3533834 0.79397684 -0.332831169 0.64325477 1

chr1_6061704_6063589 82.97385686 43.02813216 122.9195816 2.856725946 1.514362641 0.545008258 1

chr1_6257851_6261084 961.6064843 1124.735362 798.477607 0.709924871 -0.494261737 0.51375812 1

chr1_6451526_6455481 577.0236682 613.4010468 540.6462896 0.881391208 -0.182145589 0.844391944 1

chr1_7286790_7291475 260.4048955 115.0752372 405.7345537 3.525819835 1.817958757 0.179088969 1

chr1_7437900_7441110 292.3715472 96.06280668 488.6802876 5.087091503 2.346841045 0.071712803 1

chr1_7632995_7636079 173.4604688 113.0739287 233.8470088 2.068089537 1.048298648 0.521267547 1

chr1_7830469_7832542 499.0236515 535.3500164 462.6972867 0.864289292 -0.210413808 0.83103117 1

chr1_8175296_8179103 380.4677073 331.2165522 429.7188623 1.297395494 0.375618333 0.734484059 1

chr1_8240263_8247344 1104.375645 914.5979719 1294.153318 1.414996925 0.500798918 0.48754369 1

chr1_8372034_8375534 291.9614751 233.152437 350.7705132 1.504468569 0.589253966 0.637980932 1

chr1_8584846_8586687 202.5095266 217.1419693 187.877084 0.865226951 -0.20884949 0.891690852 1

chr1_8961560_8967050 915.0812945 1039.679751 790.4828375 0.760313776 -0.395333164 0.607863205 1

chr1_9239708_9244465 1170.771343 821.537128 1520.005557 1.850197034 0.887678916 0.211486589 1

chr1_9351980_9356498 585.4559787 518.3388944 652.573063 1.258969894 0.332243784 0.717228706 1

chr1_9404104_9407342 285.5167386 311.2034675 259.8300098 0.834920034 -0.260290067 0.838390365 1

chr1_9596566_9601877 1239.395295 1079.705921 1399.084668 1.295801608 0.373844853 0.590149706 1

chr1_9648027_9650533 413.0452159 482.3153419 343.7750899 0.712760014 -0.488511691 0.646594085 1

chr1_9894002_9897439 338.9490588 261.1707557 416.7273619 1.595612651 0.674110467 0.56339665 1

chr1_9910466_9914073 347.3571704 129.0843965 565.6299444 4.381861478 2.131543878 0.073887947 1

chr1_10009926_10012011 146.5225951 181.1184168 111.9267734 0.617975662 -0.694378075 0.697692146 1

chr1_10518225_10520875 165.4611211 106.069349 224.8528931 2.119866815 1.083973627 0.517967778 1

chr1_10534138_10537206 439.501075 441.2885182 437.7136319 0.991898982 -0.011734896 0.99343171 1

chr1_10727706_10732504 450.4729544 409.2675826 491.6783262 1.201361523 0.264670363 0.797017327 1

chr1_10780330_10788587 1648.27308 2066.350998 1230.195162 0.595346658 -0.748198129 0.240860405 1

chr1_10925492_10931382 700.9824913 674.4409552 727.5240274 1.078706775 0.109302749 0.899194265 1

chr1_11351765_11354141 102.4571835 37.02420674 167.8901602 4.534605193 2.180976948 0.331796571 1

chr1_11432985_11436297 262.0890031 398.260386 125.9176201 0.316169081 -1.661231807 0.215982395 1

chr1_11713666_11716015 346.0066142 356.2329081 335.7803203 0.942586473 -0.085303118 0.943413151 1

chr1_11779289_11780291 58.50622572 68.04448806 48.96796338 0.71964629 -0.474640107 0.881193949 1

chr1_12039486_12042857 630.0289117 674.4409552 585.6168682 0.868299684 -0.203735036 0.819601369 1

chr1_12220269_12225486 567.3925347 403.2636572 731.5214122 1.814002822 0.8591767 0.356065501 1

chr1_12672804_12681260 2207.606097 2370.549886 2044.662308 0.862526589 -0.213359163 0.716824647 1

chr1_13151349_13159079 1033.7772 693.4533857 1374.101013 1.981533354 0.986617252 0.183748997 1

chr1_13203512_13209359 954.7941872 640.4187112 1269.169663 1.981781046 0.986797577 0.196012334 1

chr1_13432950_13435509 143.0255375 182.119071 103.9320039 0.570681606 -0.809242032 0.654983004 1

chr1_14696158_14698659 163.4912057 150.0981354 176.8842759 1.178457516 0.23689975 0.890395725 1

chr1_14924476_14926445 263.4748765 225.1472032 301.8025498 1.340467683 0.422736439 0.748647823 1

chr1_15234123_15241592 1320.685044 839.5489042 1801.821183 2.146177756 1.101769571 0.108258771 1

chr1_15302494_15305337 186.5121393 205.1341184 167.8901602 0.818440937 -0.289049786 0.855592972 1

chr1_15415087_15418020 163.0490865 238.1557082 87.94246485 0.369264569 -1.437273254 0.398041644 1

chr1_15675199_15681497 1429.559495 1520.994439 1338.12455 0.879769522 -0.184802473 0.78096532 1

chr1_15940190_15947130 2216.673463 2482.62316 1950.723766 0.785751054 -0.347855792 0.553716691 1

chr1_16218947_16220966 190.5232585 226.1478574 154.8986597 0.684944184 -0.545941667 0.725351874 1

chr1_16301283_16303757 501.0112255 518.3388944 483.6835567 0.933141545 -0.09983216 0.920428129 1

chr1_16399237_16400869 241.9745449 203.13281 280.8162798 1.382426994 0.467203293 0.734117815 1

chr1_16443980_16450292 618.1499033 847.5541381 388.7456685 0.458667654 -1.124478924 0.212967783 1

chr1_16476901_16484780 1473.016666 1498.980046 1447.053285 0.965358605 -0.050863131 0.939276146 1

chr1_16562097_16565602 652.0217222 685.4481518 618.5952925 0.902468394 -0.148051688 0.867043017 1

chr1_16675288_16680411 1035.949862 959.6274126 1112.272311 1.159066838 0.212963763 0.773095651 1

chr1_16824400_16826833 590.0001262 590.3859994 589.614253 0.99869281 -0.00188711 1 1

chr1_16891772_16896783 792.4233219 675.4416095 909.4050343 1.346385863 0.429091934 0.597511289 1

chr1_16936937_16942450 1290.449589 1213.793589 1367.10559 1.126308132 0.17160157 0.802686258 1

chr1_17628743_17635635 703.2395225 305.1995421 1101.279503 3.608391728 1.851355967 0.03424318 1

chr1_17854954_17859496 402.9503805 327.2139353 478.6868257 1.462916991 0.548847911 0.610406956 1

chr1_17885397_17892947 1408.386174 1234.807328 1581.965021 1.281143208 0.357431751 0.59178682 1

chr1_17919660_17927103 1855.060567 1183.773961 2526.347172 2.134146597 1.09365928 0.078168305 1

chr1_18005498_18013235 1361.126517 1555.016683 1167.236352 0.750626257 -0.413833339 0.53902152 1

chr1_18622781_18629160 583.5723943 694.45404 472.6907486 0.680665273 -0.554982589 0.545346757 1

chr1_18789321_18794735 838.0080275 850.5561008 825.4599542 0.970494425 -0.043208169 0.958175235 1

chr1_18805112_18810813 903.5757328 1019.666667 787.4847989 0.772296305 -0.372773626 0.630096974 1

chr1_18923436_18927353 405.0648348 504.3297351 305.7999346 0.606349206 -0.72177919 0.502110662 1

chr1_19247233_19251416 426.9477695 347.22702 506.6685191 1.459185173 0.545162975 0.603491012 1

chr1_19280423_19284463 889.4782805 856.5600262 922.3965348 1.076861523 0.106832742 0.89188928 1

chr1_19577010_19580150 955.576398 1072.701341 838.4514547 0.781626183 -0.3554493 0.639388512 1

chr1_19610471_19614992 325.5206713 357.2335623 293.8077803 0.822452903 -0.281995029 0.813520026 1

chr1_19692019_19699266 1059.943981 974.6372261 1145.250735 1.175053348 0.232726257 0.750668653 1

chr1_19904824_19907034 103.9575108 39.02551521 168.8895064 4.327668845 2.113590108 0.341850944 1

chr1_20363342_20365982 144.4460741 62.04056265 226.8515855 3.656504322 1.870465067 0.308262609 1

chr1_20462577_20467781 388.4186573 264.1727184 512.6645962 1.940641711 0.956533787 0.384048273 1

chr1_20477976_20481081 274.975206 237.155054 312.7953579 1.318948733 0.399388488 0.757299809 1

chr1_20512512_20514090 100.5062347 110.071966 90.94050343 0.826191325 -0.275452183 0.903967761 1

chr1_20692831_20694188 99.51866092 128.0837422 70.9535796 0.553962418 -0.85213999 0.70223603 1

chr1_20790434_20793905 295.9856748 274.1792607 317.7920889 1.159066838 0.212963763 0.865648382 1

chr1_20937758_20943489 714.5371051 771.5044161 657.569794 0.852321491 -0.230530386 0.785763043 1

chr1_20986511_20989416 762.5338452 814.5325483 710.5351422 0.87232259 -0.197066344 0.811835797 1

chr1_21051273_21055725 296.4245238 181.1184168 411.7306309 2.273267613 1.184767531 0.34358803 1

chr1_21347219_21352191 783.1296639 981.6418058 584.617522 0.595550759 -0.747703621 0.360649623 1

chr1_21609385_21613688 690.4743138 651.4259078 729.5227198 1.119885947 0.163351811 0.849828678 1

chr1_21997871_22004543 576.3938447 414.2708538 738.5168355 1.78269079 0.834056487 0.367024841 1

chr1_22101348_22102493 67.49510927 60.03925417 74.95096436 1.248366013 0.320040985 0.913427569 1

chr1_22259229_22266091 1048.441035 958.6267583 1138.255312 1.187381118 0.247783076 0.736018268 1

chr1_22378126_22381053 804.0119444 822.5377822 785.4861065 0.954954439 -0.066496191 0.935808473 1

chr1_22737804_22743907 1096.567926 1200.785083 992.3507682 0.826418301 -0.275055893 0.703775496 1

chr1_23150559_23157412 1518.507846 1531.000981 1506.014711 0.983679781 -0.023739346 0.97198392 1

chr1_23344762_23347827 931.0891462 1067.69807 794.4802222 0.744105702 -0.42642052 0.577544572 1

chr1_23493524_23495741 317.0098781 332.2172064 301.8025498 0.908449484 -0.138521802 0.91040127 1

chr1_23542963_23544402 156.981721 129.0843965 184.8790454 1.432233875 0.518267096 0.763900677 1

chr1_23936820_23940508 192.4598188 131.0857049 253.8339326 1.936396747 0.953374576 0.537448818 1

chr1_24017562_24019924 495.0478495 568.3716062 421.7240928 0.74198656 -0.43053504 0.661710583 1

chr1_24279986_24288110 2381.933145 2280.491004 2483.375286 1.088965175 0.122957818 0.831653788 1

chr1_24343122_24350089 1417.454849 1348.88191 1486.027787 1.101673746 0.139697041 0.834215949 1

chr1_24540154_24545700 784.915145 655.4285247 914.4017652 1.395120491 0.480389727 0.555890189 1

chr1_24608689_24614605 737.5560766 823.5384364 651.5737169 0.791187986 -0.337907576 0.686011091 1

chr1_24655264_24658026 122.4526096 50.03271181 194.8725073 3.894901961 1.961587014 0.330650285 1

chr1_24739326_24746474 795.5299282 841.5502127 749.5096436 0.890629736 -0.167102314 0.837666232 1

chr1_24933732_24938588 672.3173446 393.2571148 951.3775743 2.419225332 1.274545152 0.14560728 1

chr1_24965617_24971410 1263.492749 1252.819104 1274.166394 1.017039404 0.024375575 0.973008415 1

chr1_25049695_25054585 913.0930664 1055.690219 770.4959136 0.729850386 -0.454327343 0.555701285 1

chr1_25802437_25806692 535.9994606 535.3500164 536.6489048 1.002426242 0.003496087 0.9993195 1

chr1_25868162_25873140 935.4887547 918.6005889 952.3769205 1.036769334 0.052094951 0.946975195 1

chr1_25983166_25991129 1455.664798 943.6169448 1967.712651 2.085287533 1.060246326 0.110935331 1

chr1_26230535_26233909 614.0720738 724.473667 503.6704805 0.695222619 -0.524453073 0.559484597 1

chr1_26361032_26363744 397.9909288 384.2512267 411.7306309 1.071514161 0.099650917 0.928681284 1

chr1_26436478_26445080 2613.90965 2476.619235 2751.200065 1.110869215 0.151688975 0.788480936 1

chr1_26623582_26626306 547.8326875 292.191037 803.474338 2.74982541 1.459340023 0.126946256 1

chr1_27030246_27034322 340.5304849 387.2531894 293.8077803 0.758696864 -0.398404521 0.732529184 1

chr1_27111635_27116186 1542.089277 1679.097808 1405.080745 0.836806968 -0.257033231 0.691960394 1

chr1_27424315_27427667 318.9693292 272.1779523 365.7607061 1.343829296 0.426349887 0.722792524 1

chr1_27851618_27856697 998.9707827 954.6241414 1043.317424 1.092909113 0.128173431 0.864540091 1

chr1_27900717_27903786 561.0341291 613.4010468 508.6672115 0.829257162 -0.270108527 0.772713367 1

chr1_28260900_28262911 360.0157735 384.2512267 335.7803203 0.873856209 -0.194532187 0.865263146 1

chr1_28622363_28624703 256.972586 215.1406608 298.8045113 1.388879769 0.473921715 0.722498825 1

chr1_28639156_28642447 301.4689984 254.166176 348.7718208 1.372219649 0.45651143 0.711500355 1

chr1_28654183_28656993 653.4932725 643.4206739 663.5658712 1.031309527 0.044477394 0.961255871 1

chr1_28850494_28855435 1182.290639 862.5639516 1502.017326 1.741340248 0.800198125 0.257856437 1

chr1_29046724_29049234 91.45064088 16.01046778 166.890814 10.42385621 3.381817183 0.17921971 1

chr1_29059371_29066160 1544.363313 1335.873405 1752.85322 1.312140217 0.391921896 0.545444744 1

chr1_29207980_29215177 1315.387462 1143.747792 1487.027133 1.300135522 0.378662013 0.578150803 1

chr1_29506347_29509778 1176.08462 1305.853778 1046.315462 0.801250094 -0.319675474 0.650813106 1

chr1_29556406_29558360 404.0334415 455.2976775 352.7692056 0.774810027 -0.368085471 0.73266068 1

chr1_30457262_30458287 32.01504936 55.03598299 8.994115723 0.16342246 -2.613321822 0.592179092 1

chr1_30817619_30819744 125.0301118 171.1118744 78.94834913 0.461384398 -1.115958876 0.56830946 1

chr1_30998285_31001314 143.5350209 197.1288845 89.94115723 0.456255599 -1.132085833 0.532104371 1

chr1_31291368_31299305 1700.139016 1913.2509 1487.027133 0.777225367 -0.363595106 0.56409093 1

chr1_31358168_31363291 1366.588912 1502.982663 1230.195162 0.818502563 -0.288941161 0.667668996 1

chr1_31373517_31376646 344.0059598 353.2309454 334.7809742 0.947767965 -0.077394197 0.949073093 1

chr1_31534880_31539724 1393.785779 1831.197252 956.3743053 0.522267224 -0.937139928 0.163463841 1

chr1_31634464_31637686 267.4997302 267.1746811 267.8247793 1.002433233 0.003506149 1 1

chr1_31699251_31704759 1060.654903 1297.848544 823.4612618 0.634481786 -0.656349347 0.37006944 1

chr1_31768111_31771302 924.1983664 1227.802748 620.5939849 0.505450885 -0.984357185 0.202334378 1

chr1_32012137_32016263 663.7686181 1074.70265 252.8345864 0.235260038 -2.087671813 0.020600185 1

chr1_32027779_32030778 184.0451669 253.1655218 114.924812 0.453951277 -1.139390633 0.472589648 1

chr1_32307557_32310457 567.1865176 852.5574093 281.815626 0.330553254 -1.597045377 0.091371073 1

chr1_32536763_32539820 962.6568442 1202.786392 722.5272965 0.600711233 -0.735256453 0.33212351 1

chr1_32796833_32803398 1972.348362 2505.638208 1439.058516 0.574328134 -0.80005286 0.187977703 1

chr1_32814695_32819181 1237.69876 1542.008178 933.3893429 0.605307648 -0.724259516 0.298078658 1

chr1_32866280_32867331 64.0150562 87.05691855 40.97319385 0.470648336 -1.087278601 0.710679804 1

chr1_33205126_33213099 3400.151806 4397.875368 2402.428244 0.546270197 -0.872313379 0.104538824 1

chr1_33437756_33440647 358.5461853 429.2806674 287.8117032 0.670451118 -0.576795946 0.611738228 1

chr1_35323898_35326670 765.682309 1044.683023 486.6815953 0.465865324 -1.102015144 0.184076288 1

chr1_35728452_35735816 1938.903619 2556.671574 1321.135665 0.516740468 -0.952488224 0.119583924 1

chr1_36191693_36194520 239.9928571 229.1498201 250.8358941 1.094637098 0.130452656 0.927122971 1

chr1_36272781_36276286 1041.184657 1323.865555 758.5037593 0.572946216 -0.80352838 0.276397075 1

chr1_36712471_36719208 1233.492089 1221.798822 1245.185355 1.019141066 0.027353758 0.969816026 1

chr1_36731151_36737925 1607.82899 2111.380438 1104.277542 0.523012112 -0.935083739 0.14672943 1

chr1_36764039_36767204 218.4977577 215.1406608 221.8548545 1.03120839 0.044335907 0.978876407 1

chr1_36861708_36864473 853.134257 1058.692182 647.5763321 0.611675748 -0.709161019 0.370444463 1

chr1_36992339_36996648 373.1138797 547.3578672 198.8698921 0.363327001 -1.460659512 0.196400194 1

chr1_37322065_37324498 168.5239079 205.1341184 131.9136973 0.643060736 -0.63697309 0.700701326 1

chr1_37912440_37917579 567.8438101 329.2152437 806.4723765 2.449681149 1.29259398 0.168271039 1

chr1_37927767_37934555 857.9988755 856.5600262 859.4377247 1.003359599 0.004838754 0.996787723 1

chr1_37978075_37981577 1320.150053 1550.013412 1090.286695 0.703404684 -0.507573152 0.455702273 1

chr1_38059881_38062295 596.131586 797.5214263 394.7417456 0.494960678 -1.01461418 0.267182889 1

chr1_38154483_38159232 1977.400685 2590.693818 1364.107551 0.526541401 -0.925381124 0.128307481 1

chr1_38450847_38456931 1622.359405 1407.92051 1836.7983 1.3046179 0.383627329 0.548226922 1

chr1_38616699_38619315 113.5435168 180.1177625 46.969271 0.260769789 -1.939151354 0.356429304 1

chr1_38681676_38684712 223.5592368 314.2054302 132.9130435 0.423013197 -1.241225423 0.388106542 1

chr1_38992906_38994036 53.50687868 64.04187112 42.97188623 0.670996732 -0.575622355 0.863587527 1

chr1_39255468_39256405 48.5036075 54.03532876 42.97188623 0.795255386 -0.330509857 0.929477159 1

chr1_39337757_39340304 905.6999975 1211.79228 599.6077149 0.494810641 -1.015051569 0.192091636 1

chr1_39648288_39651541 341.4938598 332.2172064 350.7705132 1.055846917 0.078400679 0.948569006 1

chr1_40038791_40043317 1138.080034 1260.824338 1015.335731 0.805295155 -0.312410441 0.661753415 1

chr1_40156540_40157714 81.00328744 86.05626432 75.95031055 0.882565739 -0.180224351 0.947258686 1

chr1_40303061_40307297 398.5651605 498.3258097 298.8045113 0.599616768 -0.737887367 0.495819019 1

chr1_40314920_40322255 1041.15588 1279.836768 802.4749918 0.627013547 -0.673431481 0.360898941 1

chr1_40549117_40553441 323.0504288 400.2616945 245.8391631 0.614196078 -0.703228794 0.555705956 1

chr1_40745110_40750541 757.0943411 901.5894669 612.5992154 0.679465808 -0.557527142 0.50032378 1

chr1_40844914_40852530 1732.540593 1795.1737 1669.907486 0.930220561 -0.104355266 0.868407403 1

chr1_40942249_40943780 484.6953293 783.512267 185.8783916 0.237237373 -2.075596796 0.043122093 1

chr1_41090485_41092152 88.99217077 77.05037619 100.9339653 1.309973686 0.389537833 0.872433192 1

chr1_41172948_41178818 1049.21082 1371.896958 726.5246812 0.529576713 -0.917088411 0.213707581 1

chr1_41186799_41190984 414.5710501 523.3421656 305.7999346 0.584321224 -0.775166403 0.466602126 1

chr1_41351559_41357497 518.9759122 482.3153419 555.6364825 1.152019093 0.204164627 0.833251693 1

chr1_41794087_41799546 362.4722816 320.2093556 404.7352076 1.263970588 0.337962893 0.765612768 1

chr1_41842484_41847744 643.8961482 485.3173046 802.4749918 1.653505828 0.725528131 0.411119142 1

chr1_42252939_42259300 905.4325023 802.5246975 1008.340307 1.256460157 0.329364923 0.670311695 1

chr1_42265620_42272236 1127.289319 805.5266602 1449.051978 1.798887671 0.847105102 0.238722803 1

chr1_42286072_42291833 614.9746247 576.3768401 653.5724092 1.133932462 0.181334715 0.840939057 1

chr1_42364881_42370262 578.7064678 894.5848872 262.8280484 0.293798892 -1.767099141 0.060924421 1

chr1_42381118_42386375 1618.275035 2039.333333 1197.216737 0.5870628 -0.768413253 0.230975473 1

chr1_43859668_43863639 291.9405464 201.1315015 382.7495913 1.902981823 0.928261781 0.459492729 1

chr1_43956637_43961937 991.6431159 1210.791626 772.494606 0.638007886 -0.648353838 0.387021052 1

chr1_43970409_43977654 982.0178686 1009.660124 954.3756129 0.945244434 -0.081240646 0.914982417 1

chr1_44104581_44107395 198.5271844 240.1570167 156.8973521 0.653311547 -0.614156956 0.686125064 1

chr1_44348659_44352474 684.5567193 771.5044161 597.6090225 0.774602206 -0.368472485 0.668495386 1

chr1_44512851_44518907 1086.191207 1378.901538 793.480876 0.575444188 -0.797252085 0.273160166 1

chr1_44677762_44680369 790.0930401 932.6097482 647.5763321 0.694370109 -0.526223251 0.517841444 1

chr1_44789412_44797138 1209.569912 1316.860975 1102.278849 0.837050281 -0.256613808 0.713970201 1

chr1_45137001_45143794 1139.214763 1467.959765 810.4697613 0.55210625 -0.856982161 0.231715369 1

chr1_45263748_45270608 1467.677881 1740.137717 1195.218045 0.68685256 -0.541927653 0.410645887 1

chr1_45791658_45797743 658.6770539 929.6077855 387.7463223 0.417107439 -1.261509054 0.152973336 1

chr1_45824790_45827954 380.5671187 483.3159961 277.8182412 0.57481698 -0.798825416 0.470511058 1

chr1_45954143_45959788 936.2278 1284.840039 587.6155606 0.457345306 -1.128644252 0.14304172 1

chr1_46149923_46155996 2962.570287 3835.507688 2089.632886 0.544812592 -0.876168046 0.112832772 1

chr1_46985130_46990541 382.886282 209.1367354 556.6358287 2.661588016 1.412287276 0.20574325 1

chr1_47068366_47071959 564.3944961 403.2636572 725.525335 1.799133946 0.8473026 0.363730789 1

chr1_47151992_47156126 253.8960648 95.06215244 412.7299771 4.341685587 2.118255254 0.125733902 1

chr1_47169423_47170616 45.9784271 13.00850507 78.94834913 6.068979387 2.60145392 0.490083411 1

chr1_47232311_47236263 414.0798793 536.3506706 291.8090879 0.544063994 -0.878151741 0.410310371 1

chr1_47479302_47481552 205.4761721 169.1105659 241.8417783 1.430080829 0.516096691 0.729832858 1

chr1_47494263_47499136 1014.92435 899.5881584 1130.260543 1.256419987 0.329318799 0.65746526 1

chr1_47644136_47646044 233.9712731 190.1243049 277.8182412 1.46124527 0.547198355 0.695404393 1

chr1_47901537_47906894 1674.919259 1552.01472 1797.823799 1.158380636 0.212109391 0.737796804 1

chr1_48018540_48026211 2190.853968 1203.787046 3177.920889 2.639936107 1.400503013 0.019356809 1

chr1_48216709_48218435 72.97385472 33.0215898 112.9261196 3.41976629 1.773897734 0.514748355 1

chr1_48462061_48463891 428.4827601 402.263003 454.7025171 1.130361266 0.176783934 0.867392756 1

chr1_49381230_49382281 55.51080324 72.04710501 38.97450147 0.540958606 -0.886409892 0.783537948 1

chr1_51887741_51889772 77.97516383 40.02616945 115.9241582 2.89620915 1.534165791 0.55515024 1

chr1_52133796_52135869 82.96535457 30.01962709 135.911082 4.527407407 2.178685136 0.392052203 1

chr1_52606855_52609239 965.4455956 882.5770364 1048.314155 1.187787708 0.248277007 0.742834063 1

chr1_52868932_52871839 462.9588956 400.2616945 525.6560967 1.313281046 0.39317569 0.698062735 1

chr1_53017507_53020175 922.4272738 811.5305856 1033.323962 1.273302547 0.348575257 0.650067679 1

chr1_53279453_53281245 73.4761439 37.02420674 109.9280811 2.969086734 1.570019238 0.560831489 1

chr1_53573298_53576228 200.4696308 154.1007524 246.8385093 1.601799508 0.679693581 0.652968636 1

chr1_53640270_53643747 277.9313871 173.1131829 382.7495913 2.210978881 1.144685245 0.374634726 1

chr1_53950954_53952800 83.46306559 27.01766438 139.9084668 5.178407165 2.372508405 0.35265369 1

chr1_54016951_54019685 401.8509689 174.1138371 629.5881006 3.615956728 1.854377413 0.093571032 1

chr1_54406160_54413487 1071.315468 789.5161924 1353.114743 1.713853061 0.777243424 0.287516185 1

chr1_54869288_54876325 1737.908155 1598.044815 1877.771494 1.175043075 0.232713644 0.710436437 1

chr1_54950610_54956148 1220.403139 1072.701341 1368.104936 1.275382889 0.350930431 0.614931383 1

chr1_55229045_55231417 430.965429 378.2473013 483.6835567 1.278749525 0.354733704 0.734726277 1

chr1_56655101_56658759 395.3748393 204.1334642 586.6162144 2.873689607 1.522904242 0.167446951 1

chr1_56981115_56984429 608.8444729 371.2427216 846.4462242 2.28003453 1.189055673 0.191160068 1

chr1_59275420_59283701 1895.298639 1588.038273 2202.559006 1.386968465 0.471934986 0.440624094 1

chr1_59358790_59363870 329.926166 217.1419693 442.7103628 2.03880606 1.027724547 0.386158634 1

chr1_59404019_59410963 956.2637755 595.3892706 1317.13828 2.212230461 1.145501687 0.134350651 1

chr1_59521918_59523983 137.5010104 139.0909388 135.911082 0.977138289 -0.033365341 0.989941748 1

chr1_60355895_60360648 784.8647853 578.3781486 991.351422 1.714019495 0.777383518 0.341669951 1

chr1_61388582_61391784 152.4676585 103.0673863 201.8679307 1.958601434 0.969823846 0.579457916 1

chr1_61585538_61588984 226.4160066 98.06411515 354.767898 3.617713752 1.855078261 0.201246216 1

chr1_61632043_61638194 583.3814197 402.263003 764.4998365 1.900497512 0.926377137 0.314615909 1

chr1_61718370_61721467 175.9666825 125.0817795 226.8515855 1.813626144 0.858877093 0.595807566 1

chr1_62205828_62212517 2102.448455 2024.32352 2180.57339 1.077186215 0.107267672 0.857469121 1

chr1_62732542_62734879 100.4754957 63.04121688 137.9097744 2.187612823 1.129357424 0.610736896 1

chr1_62750594_62753473 225.5088775 239.1563625 211.8613926 0.885869773 -0.174833463 0.904461674 1

chr1_62848928_62853732 360.3525951 135.0883219 585.6168682 4.33506657 2.116054148 0.071000375 1

chr1_63223669_63228789 556.8294193 296.1936539 817.4651846 2.759901078 1.464616558 0.123077716 1

chr1_63782133_63783740 240.459175 178.1164541 302.801896 1.700022031 0.765553443 0.578534067 1

chr1_64388290_64389815 61.97385237 22.0143932 101.9333115 4.63030303 2.211106614 0.469230272 1

chr1_64514941_64520384 304.3584693 88.05757279 520.6593658 5.912715389 2.563820834 0.046770324 1

chr1_64601603_64603228 60.96927399 14.00915931 107.9293887 7.704201681 2.945645471 0.352465141 1

chr1_65190231_65192042 93.45194935 20.01308472 166.890814 8.339084967 3.059889088 0.211911906 1

chr1_65321380_65324094 212.3819944 32.02093556 392.7430533 12.26519608 3.616498393 0.024751766 1

chr1_66778134_66784121 459.3813932 278.1818777 640.5809087 2.302741336 1.203352364 0.240168151 1

chr1_66910846_66912651 150.4499995 74.04841348 226.8515855 3.063557675 1.615208012 0.365047149 1

chr1_67007861_67011495 267.9523137 195.1275761 340.7770513 1.746432043 0.804410506 0.538107886 1

chr1_67517965_67520777 1179.953163 1108.724894 1251.181432 1.128486822 0.174389571 0.805232164 1

chr1_68662262_68665847 331.97064 287.1877658 376.7535142 1.311871741 0.391626677 0.739977819 1

chr1_68695563_68699075 782.3827703 603.3945045 961.3710362 1.593271117 0.671991782 0.411123232 1

chr1_70875769_70877904 475.4742678 436.285247 514.6632886 1.179648618 0.238357188 0.812665549 1

chr1_71214559_71221346 2077.407246 1936.265947 2218.548545 1.1457871 0.196339001 0.742474137 1

chr1_74267507_74270692 190.3976862 34.02224403 346.7731284 10.19254133 3.349441902 0.046351916 1

chr1_75463701_75466242 110.4382185 16.01046778 204.8659693 12.79575163 3.67759299 0.108270717 1

chr1_75543880_75547674 970.7242102 549.3591757 1392.089245 2.534023834 1.341430094 0.079301948 1

chr1_76054430_76057086 180.4742047 141.0922473 219.8561621 1.558244101 0.639921252 0.688749412 1

chr1_76250585_76255147 917.9930021 907.5933923 928.3926119 1.022916892 0.032688936 0.967564872 1

chr1_77151760_77154780 389.4140794 258.168793 520.6593658 2.016740133 1.012025197 0.356925829 1

chr1_78297154_78300726 331.5134784 352.2302912 310.7966656 0.882367796 -0.180547958 0.879904339 1

chr1_79838037_79840685 168.4637378 113.0739287 223.8535469 1.979709642 0.98528885 0.552525329 1

chr1_81097807_81101459 448.0092521 462.3022571 433.7162471 0.938165973 -0.092084919 0.930458739 1

chr1_81639035_81641914 257.5376614 315.2060844 199.8692383 0.634090673 -0.657238938 0.621570295 1

chr1_82014593_82018521 231.9078325 91.0595355 372.7561294 4.093543058 2.033350071 0.158662977 1

chr1_82164543_82166966 119.4499928 43.02813216 195.8718535 4.552181183 2.18655798 0.288358735 1

chr1_84424721_84427148 72.96339036 17.01112202 128.9156587 7.578316032 2.921877305 0.301454128 1

chr1_84732120_84739806 969.7327123 561.3670265 1378.098398 2.454897301 1.295662671 0.08985948 1

chr1_85097533_85101947 1199.17488 702.4592738 1695.890487 2.414218945 1.27155652 0.073398784 1

chr1_85664304_85672809 2981.746877 2595.697089 3367.796665 1.297453651 0.375683002 0.493643327 1

chr1_85687231_85692500 1262.201055 805.5266602 1718.875449 2.133852961 1.093460767 0.116081141 1

chr1_85704617_85707518 319.4206046 198.1295388 440.7116704 2.22436126 1.153391116 0.339159109 1

chr1_86045063_86049232 1128.761523 764.4998365 1493.02321 1.952941176 0.965648495 0.179899949 1

chr1_86064134_86066833 268.9111105 133.0870134 404.7352076 3.041132242 1.604608553 0.225503431 1

chr1_86440535_86443404 326.8463747 92.06018973 561.6325596 6.100710429 2.608977255 0.036764748 1

chr1_86981065_86984186 156.4362663 59.03859994 253.8339326 4.299457184 2.104154528 0.234944343 1

chr1_87456326_87460737 236.4022742 87.05691855 385.7476299 4.430981895 2.147626432 0.1343417 1

chr1_87782618_87786396 262.5357004 317.2073929 207.8640078 0.655293705 -0.609786422 0.643844607 1

chr1_87850278_87854374 273.5173901 300.1962709 246.8385093 0.822257081 -0.282338568 0.828315191 1

chr1_87899511_87905054 549.9608763 490.3205758 609.6011768 1.243270642 0.314140384 0.738791262 1

chr1_87912547_87920046 973.726827 556.3637554 1391.089899 2.500324446 1.322115314 0.083240675 1

chr1_88652436_88656258 278.9496999 202.1321557 355.7672442 1.760072478 0.815634839 0.524408581 1

chr1_89046580_89049908 256.3767716 68.04448806 444.7090552 6.535563245 2.708311575 0.054475865 1

chr1_89355890_89359565 619.4330952 517.3382401 721.5279503 1.394692861 0.479947447 0.591960192 1

chr1_90285505_90293356 1798.240411 1401.916585 2194.564237 1.565402864 0.646533989 0.298404906 1

chr1_90371240_90374904 265.9588535 203.13281 328.784897 1.618571107 0.694720747 0.596053051 1

chr1_92042349_92045696 237.9686578 190.1243049 285.8130108 1.503295494 0.588128619 0.671155089 1

chr1_93387827_93389069 50.47679301 15.00981354 85.94377247 5.72583878 2.51748705 0.475602877 1

chr1_93911884_93915726 1453.940141 1362.89107 1544.989212 1.133611663 0.180926506 0.784373798 1

chr1_94532687_94537623 1245.110796 1414.92509 1075.296502 0.759967089 -0.395991152 0.567741761 1

chr1_94565239_94572284 501.6456272 724.473667 278.8175874 0.384855379 -1.377611685 0.164189048 1

chr1_94580958_94586271 1293.853122 1834.199215 753.5070284 0.410809809 -1.283457467 0.064113218 1

chr1_95698563_95700929 565.4566285 499.3264639 631.586793 1.264877467 0.338997633 0.715679798 1

chr1_96418566_96420582 95.54416694 163.1066405 27.98169336 0.171554593 -2.543260342 0.283277717 1

chr1_98385810_98387411 86.017677 113.0739287 58.9614253 0.521441379 -0.939423023 0.69942782 1

chr1_100597431_100599985 482.4801555 452.2957148 512.6645962 1.13347215 0.180748944 0.857003746 1

chr1_102560730_102566068 341.8751549 151.0987897 532.6515201 3.525187205 1.817699874 0.126290278 1

chr1_103300722_103305267 810.7307162 1163.760877 457.7005557 0.393294331 -1.346318703 0.09909548 1

chr1_105740754_105743650 201.4709391 157.1027151 245.8391631 1.564830773 0.646006647 0.668346854 1

chr1_107296232_107300192 249.3806943 67.04383383 431.7175547 6.439332748 2.686911202 0.05958165 1

chr1_108084717_108089815 554.3840296 377.2466471 731.5214122 1.939106465 0.955392015 0.310129397 1

chr1_108166778_108171643 413.662613 662.4331044 164.8921216 0.248918903 -2.006252302 0.067456987 1

chr1_108182809_108187724 393.028861 437.2859012 348.7718208 0.797583045 -0.326293353 0.765155011 1

chr1_108296559_108299909 230.5441958 298.1949624 162.8934292 0.546264859 -0.872327476 0.535704356 1

chr1_108624662_108626478 93.00786816 105.0686948 80.94704151 0.770420168 -0.376282624 0.87346487 1

chr1_109608865_109611174 192.9987332 191.1249591 194.8725073 1.019607843 0.028014376 0.989200372 1

chr1_109641537_109644722 911.4122289 777.5083416 1045.316116 1.344443603 0.427009238 0.5799558 1

chr1_109655175_109662436 1471.8597 1257.822375 1685.897025 1.340329969 0.422588214 0.520520175 1

chr1_109690589_109698805 2807.257631 2437.59372 3176.921543 1.303302317 0.382171774 0.491832325 1

chr1_109720606_109726007 1226.273644 880.5757279 1571.971559 1.785163399 0.836056132 0.231788151 1

chr1_109755563_109760005 1435.238371 1035.677135 1834.799608 1.771594203 0.825048181 0.214930444 1

chr1_109805252_109808815 225.4781385 192.1256134 258.8306636 1.347194989 0.429958677 0.763182129 1

chr1_109813386_109822202 3018.321116 2745.795224 3290.847009 1.198504164 0.261234921 0.633152831 1

chr1_110051222_110053235 479.9471268 399.2610403 560.6332134 1.404177109 0.489724915 0.623282014 1

chr1_110197757_110199809 494.4592294 432.2826301 556.6358287 1.287666425 0.364758906 0.711191124 1

chr1_110247846_110249054 65.499687 65.04252536 65.95684864 1.014057315 0.020139197 1 1

chr1_110443291_110445295 78.46764268 29.01897285 127.9163125 4.408023439 2.140131895 0.415783942 1

chr1_110526121_110529237 738.4350827 639.418057 837.4521085 1.30970982 0.389247203 0.641150415 1

chr1_110639833_110642257 199.4984076 197.1288845 201.8679307 1.024040344 0.034272554 0.985539956 1

chr1_110657347_110660975 370.0314722 418.2734708 321.7894737 0.769327954 -0.378329364 0.735913671 1

chr1_110769809_110775712 461.4252131 347.22702 575.6234063 1.657772504 0.729246039 0.472523542 1

chr1_110981744_110985510 227.4382435 133.0870134 321.7894737 2.417887857 1.273747333 0.371972565 1

chr1_111311972_111315717 823.9249637 709.4638535 938.3860738 1.322669322 0.403452421 0.614421031 1

chr1_112049631_112052476 310.4094843 172.1125286 448.70644 2.607052744 1.38241977 0.260672721 1

chr1_112262815_112266591 244.4349771 145.0948643 343.7750899 2.369312599 1.244468555 0.36562238 1

chr1_112836075_112837161 38.51341569 59.03859994 17.98823145 0.304685942 -1.714605157 0.681102729 1

chr1_113147162_113148649 50.9823523 24.01570167 77.94900294 3.245751634 1.698552609 0.620476857 1

chr1_113179590_113181606 123.9928323 113.0739287 134.9117359 1.193128579 0.254749525 0.899338613 1

chr1_113228368_113233457 1189.813858 905.5920838 1474.035632 1.627703752 0.702838148 0.318705601 1

chr1_113687040_113692650 753.4213515 633.4141315 873.4285714 1.378921827 0.463540671 0.57584201 1

chr1_113928265_113935160 1702.807428 1408.921165 1996.693691 1.417179144 0.50302214 0.424959026 1

chr1_116225974_116229304 418.8208875 145.0948643 692.5469107 4.77306288 2.254915343 0.040524579 1

chr1_116993159_116998364 693.9347462 594.3886163 793.480876 1.334953016 0.416788967 0.626176245 1

chr1_117079158_117080381 47.97908155 16.01046778 79.94769532 4.993464052 2.320040985 0.522617508 1

chr1_117112170_117114684 575.9517255 502.3284266 649.5750245 1.293128141 0.370865244 0.687889303 1

chr1_117159167_117165086 632.5076565 644.4213281 620.5939849 0.96302521 -0.054354529 0.952895867 1

chr1_117208071_117211952 923.4390464 830.5430161 1016.335077 1.223699504 0.291249328 0.704675703 1

chr1_117553636_117557629 405.9287984 297.1943082 514.6632886 1.731740059 0.792222392 0.461171204 1

chr1_117749248_117757560 1671.855819 1451.949297 1891.762341 1.302912123 0.381739782 0.546736759 1

chr1_117881798_117883182 54.98431523 31.02028132 78.94834913 2.545055872 1.347697328 0.677864119 1

chr1_118170619_118177911 1358.81324 1073.701995 1643.924485 1.531080777 0.614550398 0.362495055 1

chr1_118470812_118473857 741.4887132 724.473667 758.5037593 1.046972159 0.066223079 0.938148754 1

chr1_118685288_118687436 147.9470558 67.04383383 228.8502779 3.413442591 1.771227488 0.326974598 1

chr1_119160156_119162162 95.97909182 64.04187112 127.9163125 1.997385621 0.99811289 0.661220698 1

chr1_120225595_120232773 905.4037254 758.4959111 1052.31154 1.387366134 0.472348573 0.541391676 1

chr1_120599639_120607243 1662.874783 1471.962382 1853.787185 1.25939848 0.332734831 0.599935121 1

chr1_121224813_121227024 157.4486929 79.05168466 235.8457012 2.98343675 1.576975192 0.364342805 1

chr1_143686588_143687829 132.519322 162.1059863 102.9326577 0.634971355 -0.655236585 0.729014558 1

chr1_144506150_144508592 96.97974605 66.04317959 127.9163125 1.936858784 0.953718771 0.673475189 1

chr1_144930237_144934592 1818.063829 1916.252862 1719.874796 0.897519753 -0.155984404 0.801303107 1

chr1_144981707_144985454 543.5933144 686.4488061 400.7378228 0.58378399 -0.776493449 0.412137879 1

chr1_145014444_145016231 77.48660912 57.03729147 97.93592677 1.717050797 0.77993272 0.764145355 1

chr1_145029548_145031320 70.0183276 98.06411515 41.97254004 0.428011204 -1.224279531 0.658377719 1

chr1_145309028_145312298 411.4958369 405.2649657 417.726708 1.030749617 0.043693925 0.969669819 1

chr1_145437058_145440913 592.5396951 653.4272163 531.6521739 0.813636409 -0.297543856 0.744607712 1

chr1_145506041_145508859 745.1335799 949.6208702 540.6462896 0.569328567 -0.812666603 0.330026485 1

chr1_145669673_145671250 82.54154807 146.0955185 18.98757764 0.129966873 -2.943784155 0.262211225 1

chr1_146372502_146375547 914.1277298 1109.725548 718.5299117 0.647484338 -0.627082799 0.416290692 1

chr1_147011896_147014454 293.4748829 255.1668302 331.7829356 1.300258875 0.378798885 0.76231191 1

chr1_147929575_147933683 1553.440489 1462.956493 1643.924485 1.123700187 0.168257163 0.79523094 1

chr1_148175868_148182380 871.3690549 671.4389925 1071.299117 1.595527113 0.674033125 0.390758581 1

chr1_148192924_148193663 95.49576928 89.05822703 101.9333115 1.144569288 0.194804801 0.935718067 1

chr1_148749348_148751915 207.9817319 180.1177625 235.8457012 1.30939724 0.388902843 0.794073556 1

chr1_149222111_149225547 701.5652253 801.5240432 601.6064073 0.750578117 -0.413925865 0.627067608 1

chr1_149830482_149835319 1039.189889 1329.86948 748.5102974 0.562844932 -0.82919059 0.261836294 1

chr1_150239911_150242338 619.0943116 763.4991823 474.689441 0.621728814 -0.685642653 0.444383995 1

chr1_150486344_150490097 1054.483548 1029.673209 1079.293887 1.048190705 0.067901221 0.927283378 1

chr1_150896575_150900218 705.5462595 776.5076873 634.5848316 0.817229297 -0.29118717 0.732245288 1

chr1_150941893_150948254 1908.324149 2404.57213 1412.076169 0.587246334 -0.767962292 0.210082319 1

chr1_150976485_150982388 1086.036858 1142.747138 1029.326577 0.900747456 -0.150805423 0.83596012 1

chr1_151027665_151033067 1382.619655 1566.02388 1199.21543 0.765770845 -0.385015361 0.565738732 1

chr1_151041789_151044718 1038.63397 1243.813216 833.4547237 0.670080293 -0.577594117 0.433247628 1

chr1_151370868_151373332 614.0550692 698.4566569 529.6534815 0.758319756 -0.399121786 0.657019851 1

chr1_151483187_151489602 2169.233296 2526.651947 1811.814645 0.717081214 -0.479791572 0.416827942 1

chr1_151533977_151535784 153.5265207 194.1269218 112.9261196 0.581712823 -0.781620989 0.653641178 1

chr1_151566202_151570407 303.9948328 296.1936539 311.7960117 1.052676206 0.074061744 0.95446621 1

chr1_151583022_151586381 893.0767116 1010.660779 775.4926446 0.767312496 -0.382113845 0.623023231 1

chr1_151830456_151835709 452.478187 419.274125 485.6822491 1.15838832 0.21211896 0.836806936 1

chr1_151894303_151899164 259.5814813 384.2512267 134.9117359 0.351102941 -1.510034013 0.261039138 1

chr1_151943567_151946644 195.971919 153.1000981 238.8437398 1.560049554 0.641591856 0.674926623 1

chr1_151959678_151967384 1804.171086 2066.350998 1541.991173 0.746238744 -0.422290829 0.495870894 1

chr1_152005940_152011468 1676.679234 1951.275761 1402.082707 0.718546673 -0.476846225 0.451365115 1

chr1_152731493_152734841 139.5788395 260.1701014 18.98757764 0.072981398 -3.776327409 0.061703195 1

chr1_153332180_153339734 1449.902207 1300.850507 1598.953906 1.229160382 0.297673172 0.652337245 1

chr1_153401736_153406508 281.9202696 160.1046778 403.7358614 2.521699346 1.334396278 0.299210318 1

chr1_153515236_153520519 1068.509712 1083.708538 1053.310886 0.971950344 -0.041045486 0.956379241 1

chr1_153629969_153633013 742.5436513 809.5292771 675.5580255 0.834507219 -0.261003566 0.754507684 1

chr1_153775707_153780793 571.9111753 436.285247 707.5371036 1.621730527 0.697534116 0.451391971 1

chr1_153892072_153897554 1724.740722 2093.368662 1356.112782 0.647813644 -0.62634924 0.319256494 1

chr1_154598689_154601821 1097.597357 1246.815178 948.3795357 0.760641635 -0.394711186 0.584957117 1

chr1_154907333_154913167 1783.150152 2013.316323 1552.983982 0.771356177 -0.374530911 0.5470988 1

chr1_154942537_154950552 2040.524308 2078.358849 2002.689768 0.963591908 -0.053505817 0.92963812 1

chr1_155530879_155535245 1674.726323 2021.321557 1328.131088 0.657060765 -0.605901298 0.3392325 1

chr1_155909153_155913636 611.9667757 561.3670265 662.566525 1.180273321 0.23912099 0.791101917 1

chr1_156022252_156025817 1385.698138 1689.104351 1082.291925 0.640749001 -0.642168771 0.338519136 1

chr1_156071527_156077987 2135.863112 2691.759896 1579.966329 0.586964064 -0.768655915 0.196447666 1

chr1_156119889_156126030 1084.442351 996.6516193 1172.233083 1.176171352 0.234098256 0.74732043 1

chr1_156249619_156256237 2267.96386 2213.447171 2322.480549 1.049259535 0.069371574 0.906038973 1

chr1_156335569_156340837 1291.170322 1552.01472 1030.325923 0.663863499 -0.591041463 0.388697616 1

chr1_156677336_156683667 1207.301762 1669.091266 745.5122589 0.446657576 -1.162758863 0.100013663 1

chr1_156694767_156699525 839.163685 1089.712463 588.6149068 0.540156167 -0.888551524 0.265820437 1

chr1_159841357_159843657 129.4650375 76.04972195 182.880353 2.404747162 1.265885215 0.51018359 1

chr1_159891449_159896727 2384.878208 2199.438011 2570.318405 1.168625072 0.224812147 0.696889724 1

chr1_160283802_160286736 182.0791756 303.1982336 60.96011768 0.201056968 -2.314323755 0.160088355 1

chr1_160985464_160993045 2145.504056 2152.407262 2138.60085 0.993585595 -0.009283838 0.988361668 1

chr1_161097008_161105076 2275.853332 2052.341839 2499.364825 1.217811175 0.284290456 0.626156794 1

chr1_161134452_161137724 988.0917744 1128.737978 847.4455704 0.75079034 -0.413518007 0.581154041 1

chr1_161167539_161173911 1738.120712 1923.257442 1552.983982 0.807475873 -0.308508942 0.622410153 1

chr1_161283100_161285548 1078.566614 1180.771999 976.3612291 0.826883793 -0.274243502 0.706206281 1

chr1_161431017_161436110 649.4409499 559.3657181 739.5161817 1.322062039 0.402789878 0.646638795 1

chr1_161971369_161975298 496.1060577 658.4304875 333.781628 0.506935256 -0.980126591 0.320661464 1

chr1_162283007_162284319 60.50295604 65.04252536 55.96338672 0.860412267 -0.216900001 0.947657789 1

chr1_162295539_162301106 466.9687068 419.274125 514.6632886 1.227510256 0.295735079 0.770024738 1

chr1_163774309_163781301 1351.455489 1283.839385 1419.071592 1.10533421 0.14448265 0.83110263 1

chr1_164478749_164481394 149.5212877 182.119071 116.9235044 0.642016807 -0.63931703 0.717742886 1

chr1_164589925_164593851 687.9432472 601.393196 774.4932984 1.287831827 0.36494421 0.670885572 1

chr1_164675155_164677763 317.9974519 314.2054302 321.7894737 1.024137213 0.03440902 0.979948047 1

chr1_164722712_164725899 251.9385758 158.1033693 345.7737823 2.187010838 1.12896037 0.403792561 1

chr1_164739179_164745996 1425.854458 1203.787046 1647.92187 1.368948 0.453067647 0.495121769 1

chr1_165129958_165134924 689.4049872 544.3559045 834.4540699 1.532920031 0.616282437 0.472710014 1

chr1_165296343_165299029 125.0340359 177.1157998 72.95227198 0.411890255 -1.279668101 0.513935173 1

chr1_165508283_165512022 275.5965272 423.2767419 127.9163125 0.302204917 -1.726400963 0.18847915 1

chr1_165855111_165860296 1424.828297 1162.760223 1686.896371 1.450768902 0.536817726 0.419253808 1

chr1_167689687_167693756 1222.990451 1208.790317 1237.190585 1.023494784 0.03350375 0.962845663 1

chr1_167721690_167723285 78.9679698 30.01962709 127.9163125 4.261089325 2.091222295 0.424038462 1

chr1_168065403_168069746 1284.80276 983.6431142 1585.962406 1.612335188 0.689151697 0.316105594 1

chr1_168083386_168086799 187.9679931 139.0909388 236.8450474 1.702807166 0.767915067 0.623271472 1

chr1_168145771_168150254 1115.420775 994.6503108 1236.191239 1.242840047 0.313640634 0.662659042 1

chr1_168720085_168722604 120.4748459 82.05364737 158.8960444 1.936489718 0.953443841 0.632663454 1

chr1_168885767_168892101 506.4520377 433.2832843 579.6207911 1.337740947 0.419798766 0.666601852 1

chr1_169537529_169543397 430.5965604 578.3781486 282.8149722 0.488979352 -1.032154549 0.325879758 1

chr1_169816588_169821538 1174.440408 1083.708538 1265.172278 1.167446997 0.223357052 0.752191631 1

chr1_170426099_170427728 97.53828116 156.1020609 38.97450147 0.249673203 -2.00188711 0.384002252 1

chr1_172144971_172148748 243.9189534 120.0785083 367.7593985 3.062657952 1.614784251 0.245034856 1

chr1_172390831_172392593 68.47287272 27.01766438 109.9280811 4.068748487 2.024585102 0.477787843 1

chr1_173373040_173376985 296.9333532 195.1275761 398.7391304 2.043479135 1.031027513 0.408190743 1

chr1_173650539_173656336 504.2963799 193.1262676 815.4664923 4.222452504 2.078081195 0.039464072 1

chr1_173833558_173840567 2730.727203 3079.013085 2382.441321 0.773767845 -0.370027317 0.508255922 1

chr1_174127206_174130135 844.4743467 805.5266602 883.4220333 1.096701173 0.133170477 0.867665341 1

chr1_174160886_174162149 67.47287251 26.01701014 108.9287349 4.186827552 2.065857497 0.473485868 1

chr1_174931650_174939781 1659.099112 1811.184168 1507.014057 0.832060087 -0.265240378 0.676150127 1

chr1_175159168_175164288 1482.608558 1649.078181 1316.138934 0.798105844 -0.325348006 0.620033466 1

chr1_176174675_176177986 1543.135713 1751.144913 1335.126512 0.762430626 -0.391322024 0.546153664 1

chr1_177224006_177228167 499.621428 685.4481518 313.7947041 0.457794953 -1.127226539 0.253206302 1

chr1_177912560_177917213 352.6756804 621.4062807 83.94508009 0.135088883 -2.888019145 0.017958565 1

chr1_178993997_178996437 741.0976078 890.5822703 591.6129454 0.664299038 -0.59009527 0.479333822 1

chr1_180122755_180128763 1345.332531 1854.2123 836.4527623 0.451109489 -1.148450463 0.092524872 1

chr1_180234279_180241980 1112.702658 1422.930324 802.4749918 0.563959442 -0.826336682 0.252362163 1

chr1_180600733_180602340 313.5651423 413.2701996 213.860085 0.517482473 -0.950418095 0.43341815 1

chr1_181056904_181060209 893.1015645 1048.68564 737.5174893 0.703277952 -0.507833105 0.513594537 1

chr1_181069889_181077347 1172.552246 1252.819104 1092.285387 0.871862014 -0.197828271 0.779981579 1

chr1_181599313_181601425 87.53239282 137.0896304 37.97515528 0.277009685 -1.851991679 0.448104613 1

chr1_181615893_181617247 56.03336712 107.0700033 4.996730957 0.046667888 -4.421426001 0.217147729 1

chr1_182612397_182617894 523.5272539 565.3696435 481.6848643 0.851982185 -0.231104831 0.810663307 1

chr1_182991094_182993938 884.0296201 929.6077855 838.4514547 0.901941085 -0.148894895 0.84947131 1

chr1_183271249_183275681 309.8960767 151.0987897 468.6933638 3.101900186 1.633152264 0.187096097 1

chr1_184385498_184390668 892.3173916 613.4010468 1171.233736 1.909409419 0.933126481 0.23233193 1

chr1_184449483_184455686 775.9589626 713.4664704 838.4514547 1.175179899 0.232881624 0.776712118 1

chr1_184580066_184582435 130.472886 89.05822703 171.8875449 1.930058016 0.948644214 0.618838767 1

chr1_185284677_185288023 1123.662111 1371.896958 875.4272638 0.638114443 -0.648112906 0.366689054 1

chr1_185562869_185565592 108.0373024 165.107949 50.96665577 0.308686869 -1.695783982 0.430057039 1

chr1_185613244_185620230 539.1610048 785.5135755 292.8084341 0.372760501 -1.423679099 0.138760189 1

chr1_187022687_187024243 69.02290554 104.0680406 33.97777051 0.326495726 -1.614863986 0.565494053 1

chr1_189710290_189711174 26.0157021 50.03271181 1.998692383 0.039947712 -4.645743299 0.457679152 1

chr1_191577378_191578369 41.01308922 61.03990841 20.98627002 0.343812279 -1.540307024 0.698711155 1

chr1_193887641_193888757 51.02290169 86.05626432 15.98953906 0.185803314 -2.428151864 0.487003075 1

chr1_195706798_195709902 214.1118836 385.251881 42.97188623 0.111542314 -3.16433699 0.043924907 1

chr1_197114950_197116712 248.5317732 297.1943082 199.8692383 0.672520411 -0.572350041 0.672856681 1

chr1_197852907_197854321 61.47679536 26.01701014 96.93658058 3.725892408 1.897586015 0.533121176 1

chr1_197884573_197886042 125.4990458 124.0811253 126.9169663 1.022854733 0.032601267 0.991171883 1

chr1_198655863_198657726 74.4669878 24.01570167 124.9182739 5.201525054 2.378934674 0.384699502 1

chr1_198900854_198907394 1188.858985 973.6365719 1404.081399 1.442100101 0.528171311 0.453068199 1

chr1_200589212_200590644 138.0294605 183.1197252 92.93919581 0.507532412 -0.978428137 0.596553976 1

chr1_200637540_200640393 825.1401372 1039.679751 610.600523 0.587296735 -0.767838479 0.338487968 1

chr1_201241648_201247557 870.1244503 1060.69349 679.5554102 0.64067086 -0.642344723 0.413523545 1

chr1_201272776_201279221 1521.310332 1996.305201 1046.315462 0.524126001 -0.932014414 0.154672685 1

chr1_202043336_202047821 585.5985556 736.4815179 434.7155933 0.590260017 -0.760577475 0.407268305 1

chr1_202209288_202211592 146.5278273 189.1236507 103.9320039 0.54954525 -0.863689816 0.628913034 1

chr1_202276456_202279699 193.4964443 188.1229964 198.8698921 1.057126964 0.080148659 0.961744444 1

chr1_202521620_202526882 281.4153643 152.0994439 410.7312847 2.700412797 1.433179961 0.266367724 1

chr1_202554649_202561507 497.8928468 334.2185149 661.5671788 1.979445032 0.985096005 0.317510018 1

chr1_203239550_203244543 430.5520869 510.3336605 350.7705132 0.68733564 -0.540913326 0.604965657 1

chr1_203272701_203279945 2301.787935 2742.793262 1860.782609 0.678426127 -0.559736365 0.336757059 1

chr1_203341734_203344170 199.9660336 148.096827 251.8352403 1.700476948 0.765939448 0.612846369 1

chr1_203455820_203458154 293.3924761 129.0843965 457.7005557 3.545746567 1.826089423 0.152971416 1

chr1_203504310_203508599 287.8973801 131.0857049 444.7090552 3.392506112 1.762351415 0.170847931 1

chr1_203567397_203571970 267.4402142 176.1151456 358.7652827 2.037106358 1.026521306 0.433654569 1

chr1_203603779_203609350 543.8307246 285.1864573 802.4749918 2.813860796 1.492550959 0.119978849 1

chr1_203617406_203623142 1126.813191 841.5502127 1412.076169 1.677946422 0.746696651 0.298422637 1

chr1_203879247_203887134 1176.338381 1694.107622 658.5691402 0.388741029 -1.363118712 0.057044593 1

chr1_203897024_203904053 1642.42285 1524.997056 1759.848643 1.154001338 0.206644897 0.745725985 1

chr1_204029002_204031480 149.0072261 160.1046778 137.9097744 0.861372549 -0.215290748 0.905854339 1

chr1_204980216_204985768 658.8883031 488.3192673 829.4573389 1.698596378 0.764343079 0.38236769 1

chr1_205284930_205291663 1687.695587 1987.299313 1388.09186 0.698481528 -0.517706133 0.412796401 1

chr1_205416390_205423227 459.7901572 139.0909388 780.4893756 5.611360324 2.488350556 0.019609655 1

chr1_205717139_205720632 1169.098353 1319.862938 1018.333769 0.77154509 -0.374177623 0.596910706 1

chr1_206093693_206098316 340.884311 164.1072947 517.6613272 3.154407779 1.657369174 0.161860335 1

chr1_206847593_206849316 96.96731963 47.0307491 146.9038901 3.123571131 1.643196384 0.47217988 1

chr1_206896359_206898026 103.9830177 78.05103043 129.9150049 1.664488017 0.735078485 0.735227629 1

chr1_206912527_206913628 56.50295518 61.03990841 51.96600196 0.851344691 -0.232184729 0.946379666 1

chr1_207174210_207177387 321.0033388 326.213281 315.7933965 0.968058062 -0.046834516 0.971457248 1

chr1_207203753_207209416 725.4219995 606.3964672 844.4475318 1.392566708 0.477746438 0.569893808 1

chr1_207470010_207473184 229.555314 314.2054302 144.9051978 0.461179801 -1.116598768 0.430372753 1

chr1_207519094_207520787 112.994138 104.0680406 121.9202354 1.171543489 0.22841051 0.915148238 1

chr1_207961638_207965070 286.0294922 331.2165522 240.8424322 0.727144916 -0.45968518 0.716564923 1

chr1_208303818_208307123 331.0301558 377.2466471 284.8136646 0.754979976 -0.405489714 0.731401244 1

chr1_208338606_208341407 163.5154045 187.1223422 139.9084668 0.747684457 -0.419498552 0.804267626 1

chr1_211877091_211879932 215.4650559 162.1059863 268.8241255 1.658323247 0.72972525 0.616362376 1

chr1_211906770_211911646 969.8968719 812.5312398 1127.262504 1.387346663 0.472328326 0.531332583 1

chr1_212318531_212323748 905.5750792 1020.667321 790.4828375 0.774476483 -0.368706662 0.633566986 1

chr1_212778884_212783854 1447.456817 1381.9035 1513.010134 1.094873943 0.130764776 0.843747222 1

chr1_213187791_213190393 920.5894709 1057.691528 783.4874141 0.740752283 -0.432936927 0.573316501 1

chr1_214611026_214614664 269.5599007 361.2361793 177.8836221 0.49243025 -1.022008705 0.433877793 1

chr1_214744254_214749662 680.0361167 735.4808636 624.5913697 0.84922858 -0.23577517 0.785148092 1

chr1_214775713_214778014 243.5376584 301.1969251 185.8783916 0.617132434 -0.696347975 0.610996824 1

chr1_214806794_214812943 457.1734137 722.4723586 191.8744688 0.26558036 -1.912779636 0.067626911 1

chr1_215711696_215715664 267.5932554 410.2682369 124.9182739 0.304479515 -1.715582924 0.197486096 1

chr1_216382559_216384601 92.95816246 29.01897285 156.8973521 5.40671625 2.434752644 0.310208807 1

chr1_216937408_216941231 322.9797944 292.191037 353.7685518 1.210744024 0.275893882 0.818215365 1

chr1_217199316_217205414 834.9406625 744.4867518 925.3945733 1.242996697 0.313822463 0.693886358 1

chr1_217249144_217253393 280.5023491 284.1858031 276.818895 0.974077143 -0.037892063 0.979282775 1

chr1_217282553_217286059 292.4585321 229.1498201 355.7672442 1.552553015 0.634642533 0.61201174 1

chr1_217544892_217547742 264.0837713 392.2564606 135.911082 0.346485261 -1.529134112 0.251305536 1

chr1_218561364_218567666 761.9203722 640.4187112 883.4220333 1.379444444 0.464087355 0.57374085 1

chr1_218790564_218795118 363.5710392 472.3087995 254.8332788 0.539548023 -0.890176722 0.431438008 1

chr1_219689722_219693339 253.0876931 387.2531894 118.9221968 0.307091588 -1.703259102 0.212910187 1

chr1_220444659_220446878 551.0465534 622.4069349 479.6861719 0.770695416 -0.375767284 0.689467172 1

chr1_220507744_220513255 532.4101858 395.2584233 669.5619483 1.693985273 0.760421333 0.425854694 1

chr1_220540446_220546924 780.8863671 607.3971214 954.3756129 1.571254751 0.651917107 0.425491575 1

chr1_220692045_220698207 849.831444 592.3873079 1107.27558 1.869175057 0.902401691 0.256318777 1

chr1_220904640_220907083 178.5448387 247.1615964 109.9280811 0.44476198 -1.168894628 0.468513039 1

chr1_220920546_220923301 678.1067507 841.5502127 514.6632886 0.611565752 -0.709420478 0.41188927 1

chr1_221051984_221054067 366.576272 483.3159961 249.8365479 0.516921745 -0.951982204 0.398759093 1

chr1_222018723_222020713 243.9444603 159.1040236 328.784897 2.066477576 1.047173709 0.445384552 1

chr1_222755616_222757015 71.47025727 26.01701014 116.9235044 4.494117647 2.168037892 0.43682543 1

chr1_222772481_222775320 228.5134563 249.1629048 207.8640078 0.834249416 -0.261449323 0.854851415 1

chr1_223492759_223494186 70.50819036 83.05430161 57.96207911 0.697881723 -0.518945546 0.852377847 1

chr1_223672888_223674697 127.0117996 145.0948643 108.9287349 0.750741492 -0.413611875 0.832221693 1

chr1_223723042_223724447 98.99936715 98.06411515 99.93461915 1.019074296 0.027259236 0.995358384 1

chr1_223918360_223922693 500.001415 502.3284266 497.6744034 0.990735099 -0.013428731 0.991338886 1

chr1_223935207_223937306 116.999371 116.0758914 117.9228506 1.015911652 0.022774945 0.995798291 1

chr1_224032001_224035890 1237.619624 1420.929015 1054.310232 0.74198656 -0.43053504 0.535273961 1

chr1_224178989_224182531 1066.52737 1108.724894 1024.329846 0.923880984 -0.114221081 0.876499379 1

chr1_224329490_224332360 216.0020083 219.1432777 212.8607388 0.971331364 -0.041964549 0.980359524 1

chr1_224750812_224756921 1125.677808 1397.913968 853.4416475 0.610510852 -0.711911155 0.321458665 1

chr1_224803150_224805326 396.5684301 501.3277724 291.8090879 0.582072456 -0.780729344 0.472311569 1

chr1_225718253_225722514 355.5023651 359.2348708 351.7698594 0.979219692 -0.030295525 0.981456851 1

chr1_226294460_226299816 1411.600694 1566.02388 1257.177509 0.802783103 -0.316917844 0.634245989 1

chr1_227914962_227919043 625.5429722 691.4520772 559.6338672 0.809360309 -0.305145993 0.7325906 1

chr1_228078429_228085299 940.0924182 1081.707229 798.477607 0.738164251 -0.437986224 0.565925269 1

chr1_228113828_228116876 198.984346 175.1144913 222.8542007 1.272619981 0.347801679 0.819928768 1

chr1_228274897_228279002 498.8385632 252.1648675 745.5122589 2.956447764 1.563864787 0.116928506 1

chr1_228359911_228364887 777.9210297 657.4298332 898.4122262 1.366552263 0.450540636 0.581979299 1

chr1_228604499_228606574 137.459807 76.04972195 198.8698921 2.61499828 1.386809998 0.456766865 1

chr1_228965422_228967660 193.9712645 150.0981354 237.8443936 1.584592593 0.664111963 0.665858286 1

chr1_229192879_229196909 265.9052236 121.0791626 410.7312847 3.392254092 1.762244237 0.187530348 1

chr1_229224901_229232093 914.8576188 697.4560027 1132.259235 1.623413134 0.69903019 0.365059741 1

chr1_229273856_229278958 336.9052388 192.1256134 481.6848643 2.507135076 1.326039726 0.261598279 1

chr1_229405895_229410600 726.1165713 904.5914296 547.6417129 0.605402279 -0.724033989 0.389729555 1

chr1_229979072_229983654 358.5945829 503.3290808 213.860085 0.424891176 -1.234834713 0.280973023 1

chr1_231273853_231277638 750.4671324 700.4579654 800.4762994 1.142789916 0.192560211 0.817220692 1

chr1_231911791_231916135 579.0544077 662.4331044 495.675711 0.748265308 -0.418378206 0.649557501 1

chr1_232468554_232472634 554.6077053 719.4703959 389.7450147 0.541710982 -0.884404756 0.346858043 1

chr1_232733275_232738683 1097.751706 718.4697416 1477.033671 2.055804978 1.039703411 0.153148391 1

chr1_232745833_232753039 851.7725824 504.3297351 1199.21543 2.377840025 1.249651657 0.118114124 1

chr1_232799821_232801188 67.52846441 111.0726202 23.9843086 0.215933581 -2.211340475 0.444620734 1

chr1_233242590_233245074 267.960162 207.1354269 328.784897 1.58729437 0.666569707 0.609695819 1

chr1_233748482_233752497 1688.715861 2019.320249 1358.111474 0.672558736 -0.572267828 0.365436909 1

chr1_233848262_233852489 325.0569695 412.2695453 237.8443936 0.576914779 -0.793569873 0.505120902 1

chr1_234633261_234639770 833.4678042 784.5129212 882.4226871 1.124803255 0.169672674 0.832256169 1

chr1_234666584_234669047 428.9124528 295.1929997 562.6319058 1.905979838 0.930532858 0.375837302 1

chr1_234699743_234701798 184.4722435 142.0929015 226.8515855 1.596501887 0.674914258 0.669043872 1

chr1_234973999_234979761 433.4010083 282.1844946 584.617522 2.071756362 1.050854353 0.315960407 1

chr1_235289328_235293668 1369.467265 1319.862938 1419.071592 1.075165876 0.104559256 0.877149523 1

chr1_235322845_235325899 904.9517959 831.5436703 978.3599215 1.176558678 0.234573273 0.762110947 1

chr1_236272208_236274583 194.9745348 156.1020609 233.8470088 1.498039216 0.583075391 0.703973464 1

chr1_236304295_236307273 1094.367795 892.5835787 1296.15201 1.452135174 0.538175755 0.457132766 1

chr1_236551566_236554961 625.185876 909.5947008 340.7770513 0.374647138 -1.416395665 0.117455268 1

chr1_236730112_236733393 265.4264793 153.1000981 377.7528604 2.467358708 1.302967472 0.324539404 1

chr1_237055618_237060532 556.9196744 434.2839385 679.5554102 1.564772145 0.645952594 0.490182491 1

chr1_238647899_238649449 66.02748306 108.0706575 23.9843086 0.221931736 -2.171812111 0.45865948 1

chr1_239549091_239550763 70.52911907 115.0752372 25.98300098 0.225791418 -2.146937442 0.444771646 1

chr1_240041196_240044705 443.9863605 423.2767419 464.695979 1.097853799 0.134685943 0.897710605 1

chr1_240598800_240601027 71.53762158 129.0843965 13.99084668 0.108385266 -3.205759443 0.268334432 1

chr1_241270065_241273030 157.0876726 291.1903827 22.9849624 0.078934483 -3.663200496 0.052178342 1

chr1_241319028_241321198 94.55136097 173.1131829 15.98953906 0.092364653 -3.436515337 0.165032885 1

chr1_243416194_243420650 2196.961229 2138.398103 2255.524354 1.054772893 0.076932401 0.896595942 1

chr1_244415611_244419207 361.0589392 451.2950605 270.8228179 0.600101445 -0.736721691 0.515667223 1

chr1_244461995_244469681 1245.615701 1422.930324 1068.301079 0.750775397 -0.413546721 0.550655466 1

chr1_244529165_244535943 931.2670402 1339.876022 522.6580582 0.390079417 -1.358160219 0.080136148 1

chr1_244612921_244620119 2485.568877 2591.694472 2379.443282 0.918103313 -0.123271587 0.829523947 1

chr1_245316420_245320748 538.1368057 747.4887145 328.784897 0.439852657 -1.184907769 0.215725752 1

chr1_246610890_246614985 275.0079071 287.1877658 262.8280484 0.915178429 -0.127875047 0.923280263 1

chr1_246758919_246762399 260.0262165 300.1962709 219.8561621 0.732374728 -0.449346087 0.734947158 1

chr1_246769544_246777322 1632.993155 1623.061171 1642.925139 1.012238582 0.01754937 0.979071325 1

chr1_247266128_247269058 1180.072849 1291.844619 1068.301079 0.826957873 -0.274114257 0.697706325 1

chr1_247493237_247497458 2431.596334 2579.686621 2283.506048 0.885187382 -0.175945208 0.759525072 1

chr1_249118633_249121880 1466.042825 1532.001636 1400.084014 0.913891984 -0.129904437 0.844164055 1

chr1_249131161_249136277 1747.452303 1675.095191 1819.809415 1.086391641 0.119544285 0.848957173 1

chr1_249149605_249154557 2615.583947 2744.79457 2486.373324 0.905850424 -0.142655246 0.800815392 1

chr1_249199263_249202118 962.5136133 983.6431142 941.3841124 0.957038278 -0.063351466 0.934490752 1

chr1_9822_10779 160.5049395 168.1099117 152.8999673 0.90952381 -0.13681669 0.939004759 1

chr1_10941_16071 568.3212465 295.1929997 841.4494932 2.850506259 1.511218169 0.109025502 1

chr1_27966_30401 1141.539158 1201.785738 1081.292579 0.899738236 -0.152422761 0.831388965 1

chr1_91408_92581 70.00066899 71.04645077 68.95488721 0.970560619 -0.043109772 0.993306248 1

chr1_235991_238709 221.9941613 213.1393523 230.8489702 1.083089386 0.115152312 0.938752275 1

chr1_411691_412682 48.00262636 52.03402028 43.97123243 0.845047763 -0.242895209 0.950371781 1

chr1_712849_716146 883.0001888 883.5776906 882.4226871 0.99869281 -0.00188711 0.999739619 1

chr1_761619_763887 769.0570644 856.5600262 681.5541026 0.795687496 -0.329726167 0.68850171 1

chr1_770560_774184 176.0510514 254.166176 97.93592677 0.385322423 -1.375861952 0.398850105 1

chr1_778668_783279 453.645617 676.4422637 230.8489702 0.341269289 -1.551017504 0.135423545 1

chr1_804921_805879 63.00328359 68.04448806 57.96207911 0.851826221 -0.231368956 0.942024532 1

chr1_838845_842092 696.5940012 840.5495584 552.6384439 0.657472767 -0.604996957 0.47902681 1

chr1_848182_850329 199.0602126 291.1903827 106.9300425 0.367216944 -1.445295466 0.345565258 1

chr1_854109_862254 1746.283565 2180.425581 1312.141549 0.601782313 -0.73268639 0.242935949 1

chr1_872780_878484 957.5410812 1020.667321 894.4148414 0.876303986 -0.190496674 0.802120089 1

chr1_892491_897387 1019.567255 1122.734053 916.4004576 0.816222199 -0.292966147 0.692857383 1

chr1_910243_912453 476.5180875 504.3297351 448.70644 0.889708476 -0.168595398 0.867410961 1

chr1_954161_962321 1942.151495 2174.421655 1709.881334 0.786361435 -0.346735524 0.568311462 1

chr1_993780_996209 448.9922478 437.2859012 460.6985943 1.053540928 0.075246361 0.943550259 1

chr1_1002032_1006678 1311.658227 1554.016029 1069.300425 0.688088414 -0.539334143 0.429038669 1

chr1_1006703_1012008 1706.773419 2125.389598 1288.157241 0.606080524 -0.722418612 0.252582988 1

chr1_1049585_1052890 615.5213874 648.4239451 582.6188296 0.898515291 -0.154385039 0.864573546 1

chr1_1056217_1062354 1235.977374 1201.785738 1270.169009 1.056901384 0.07984077 0.90942911 1

chr1_1136344_1137736 157.5167113 183.1197252 131.9136973 0.720368585 -0.473192828 0.783769502 1

chr1_1140241_1143091 487.1367948 696.4553484 277.8182412 0.398903163 -1.325889533 0.185561942 1

chr1_1165064_1169152 1200.080047 1322.8649 1077.295194 0.814365242 -0.296252108 0.672866149 1

chr1_1171351_1175903 317.0589298 407.2662742 226.8515855 0.557010486 -0.844223607 0.483694722 1

chr1_1206322_1210920 1366.082699 1492.97612 1239.189277 0.830012792 -0.268794525 0.689670358 1

chr1_1216183_1218480 213.4715957 170.1112202 256.8319712 1.509788543 0.594346504 0.684744395 1

chr1_1223423_1227959 317.9575566 253.1655218 382.7495913 1.511855124 0.596319898 0.619874064 1

chr1_1263801_1269757 974.9511568 900.5888126 1049.313501 1.165141612 0.220505312 0.770099833 1

chr1_1278189_1285807 2034.057335 2122.387635 1945.727035 0.916763273 -0.125378846 0.834996674 1

chr1_1298294_1301818 437.541624 501.3277724 373.7554756 0.74553116 -0.423659443 0.683396415 1

chr1_1332698_1336709 1321.551623 1400.915931 1242.187316 0.886696545 -0.17348764 0.79904904 1

chr1_1356036_1357255 56.49249083 45.02944063 67.95554102 1.509135802 0.593722635 0.853808481 1

chr1_1365615_1372469 2343.014236 2365.546614 2320.481857 0.980949537 -0.027749173 0.962540971 1

chr1_1396655_1398476 100.9764768 65.04252536 136.9104282 2.104937154 1.07377716 0.627231904 1

chr1_1439409_1441361 109.9509718 35.02289827 184.8790454 5.278804855 2.400211334 0.269645309 1

chr1_1446076_1450256 1257.524795 1295.847236 1219.202354 0.940853459 -0.08795806 0.8995564 1

chr1_1493707_1495581 75.47549031 38.02486098 112.9261196 2.969797042 1.570364339 0.554167852 1

chr1_1502015_1504475 173.9418292 85.05561008 262.8280484 3.090073049 1.627640944 0.324210162 1

chr1_1508496_1511885 1250.050627 1327.868172 1172.233083 0.882793268 -0.179852467 0.79543301 1

chr1_1534170_1536369 309.5363644 365.2387962 253.8339326 0.69498075 -0.524955077 0.66645913 1

chr1_1548718_1554422 1409.986567 1389.908734 1430.0644 1.028890865 0.041089963 0.951850089 1

chr1_1609569_1611051 371.3205503 97.06346092 645.5776397 6.651088202 2.733590403 0.020978354 1

chr1_1675227_1679075 1352.55817 1441.942754 1263.173586 0.876022007 -0.190960982 0.777607863 1

chr1_1688591_1696985 2792.147098 2253.47334 3330.820856 1.478083098 0.56372738 0.311796468 1

chr1_1707647_1715648 2708.890704 2542.662414 2875.118993 1.130751364 0.177281736 0.751826716 1

chr1_1759802_1761286 52.47417734 13.00850507 91.93984962 7.067672197 2.821235128 0.417008766 1

chr1_1788437_1792670 463.0256059 502.3284266 423.7227852 0.843517434 -0.245510209 0.809300491 1

chr1_1820539_1824510 1079.533259 1130.739287 1028.327231 0.909429117 -0.1369669 0.851261715 1

chr1_1836131_1842156 1690.776686 1349.882565 2031.670807 1.505072264 0.589832757 0.350817154 1

chr1_1874688_1876058 71.99870735 70.04579654 73.95161817 1.055760971 0.078283239 0.982372443 1

chr1_1886251_1888852 155.4552327 87.05691855 223.8535469 2.571347006 1.362524317 0.434102151 1

chr1_1950315_1951537 117.49512 110.071966 124.9182739 1.134878194 0.182537462 0.931460334 1

chr1_1962177_1965603 490.9772142 456.2983317 525.6560967 1.152000917 0.204141866 0.83721179 1

chr1_1975841_1977149 177.9895737 162.1059863 193.8731612 1.195965464 0.25817573 0.874504868 1

chr1_2015565_2017768 136.9195845 14.00915931 259.8300098 18.54715219 4.213125781 0.043591489 1

chr1_2023271_2025310 99.96339613 44.02878639 155.8980059 3.540819964 1.824083491 0.418661338 1

chr1_2034372_2038780 473.3434629 234.1530913 712.5338345 3.043025529 1.605506437 0.115977538 1

chr1_2064593_2070250 1042.800092 737.4821721 1348.118012 1.828000816 0.870266714 0.238663215 1

chr1_2120106_2122710 554.5566915 641.4193654 467.6940176 0.729154813 -0.455702937 0.626868346 1

chr1_2124558_2127950 655.0243389 692.4527315 617.5959463 0.89189618 -0.165052309 0.851446194 1

chr1_2138143_2141597 493.4062533 350.2289827 636.583524 1.817620915 0.862051341 0.382702616 1

chr1_2157672_2159586 573.5253025 612.4003926 534.6502125 0.873040284 -0.195879871 0.83309792 1

chr1_2206566_2208565 133.5160521 158.1033693 108.9287349 0.688971622 -0.537483533 0.775842947 1

chr1_2257521_2258938 63.97973899 33.0215898 94.93788819 2.875024757 1.523574379 0.604837703 1

chr1_2283614_2285060 54.48595017 33.0215898 75.95031055 2.300019806 1.201646285 0.712547146 1

chr1_2312594_2313931 52.48791181 34.02224403 70.9535796 2.085505575 1.060397169 0.751275341 1

chr1_2321503_2324814 823.0394174 883.5776906 762.5011441 0.862970118 -0.21261749 0.791271114 1

chr1_2514580_2522441 1405.035618 1459.954531 1350.116705 0.924766269 -0.112839318 0.866340278 1

chr1_2573207_2575336 271.1125498 443.2898267 98.93527296 0.223184172 -2.163693378 0.1069323 1

chr1_2583284_2587821 244.9647352 191.1249591 298.8045113 1.563398693 0.644685737 0.636716649 1

chr1_2979523_2981044 153.0046109 160.1046778 145.904544 0.91130719 -0.133990646 0.942048243 1

chr1_2984454_2987934 872.9138558 741.4847891 1004.342922 1.354502395 0.437762944 0.576501405 1

chr1_3000754_3003250 124.4715767 81.05299314 167.8901602 2.071362866 1.05058031 0.591867754 1

chr1_3016678_3019154 147.9594823 86.05626432 209.8627002 2.438668491 1.286093653 0.471594585 1

chr1_3022591_3024466 129.9705968 85.05561008 174.8855835 2.056132257 1.039933066 0.586573872 1

chr1_3048147_3053484 848.4409924 758.4959111 938.3860738 1.237166951 0.3070402 0.698483469 1

chr1_3072869_3074891 109.4480286 30.01962709 188.8764302 6.291764706 2.653464719 0.227940268 1

chr1_3081320_3082836 84.4833405 59.03859994 109.9280811 1.861969647 0.896829555 0.715488171 1

chr1_3102987_3104273 75.48922478 59.03859994 91.93984962 1.557283704 0.639031797 0.809590633 1

chr1_3129854_3131271 53.51211086 72.04710501 34.9771167 0.485475672 -1.042529094 0.752219734 1

chr1_3186537_3190144 200.9025937 52.03402028 349.771167 6.72197084 2.748884284 0.084425538 1

chr1_3235558_3242306 860.6993339 401.2623487 1320.136319 3.289958111 1.718069215 0.033179044 1

chr1_3295799_3298104 134.4800811 104.0680406 164.8921216 1.584464555 0.663995387 0.723325405 1

chr1_3309663_3311061 79.02683181 120.0785083 37.97515528 0.316252723 -1.660850192 0.520610788 1

chr1_3369172_3374027 1122.938108 1028.672555 1217.203661 1.183276112 0.242786759 0.735217136 1

chr1_3395875_3401003 1030.974714 992.6490024 1069.300425 1.07721906 0.107311661 0.885382871 1

chr1_3417995_3420287 169.4558897 102.0667321 236.8450474 2.320492118 1.214430797 0.46411915 1

chr1_3444888_3450477 1500.889791 1332.871443 1668.90814 1.25211486 0.324366911 0.619799388 1

chr1_3467034_3472916 1062.690875 590.3859994 1534.99575 2.599986707 1.378504247 0.063002308 1

chr1_3502134_3506003 234.4598278 173.1131829 295.8064727 1.70874608 0.772938028 0.579710001 1

chr1_3515282_3517591 176.9647206 123.0804711 230.8489702 1.875593815 0.907347427 0.574204465 1

chr1_3527019_3528371 136.9876029 118.0771999 155.8980059 1.320305749 0.40087206 0.830319203 1

chr1_3528977_3531153 195.9791132 164.1072947 227.8509317 1.38842659 0.4734509 0.757467521 1

chr1_3534127_3536452 111.9653608 59.03859994 164.8921216 2.79295447 1.481792055 0.479409313 1

chr1_3539936_3542762 536.0184273 564.3689892 507.6678653 0.899531822 -0.152753775 0.873629925 1

chr1_3565537_3567051 348.0105388 364.238142 331.7829356 0.91089564 -0.134642318 0.908877094 1

chr1_3568211_3569757 181.9692999 135.0883219 228.8502779 1.694078916 0.760501082 0.632469022 1

chr1_3584875_3586383 103.9954441 97.06346092 110.9274273 1.142834041 0.192615915 0.93284341 1

chr1_3589000_3594314 581.4180446 456.2983317 706.5377574 1.548411879 0.630789281 0.492683579 1

chr1_3611974_3613115 61.00262914 65.04252536 56.96273292 0.875776772 -0.191364908 0.954275197 1

chr1_3687928_3695070 893.8667706 690.451423 1097.282118 1.589224211 0.668322678 0.390347232 1

chr1_3705053_3706784 66.96731321 17.01112202 116.9235044 6.873356401 2.781014769 0.348448425 1

chr1_3711574_3714285 709.5698051 816.5338568 602.6057535 0.738004614 -0.43829826 0.605295848 1

chr1_3772495_3775205 723.0446282 791.5175009 654.5717554 0.826983301 -0.274069898 0.74514724 1

chr1_4655817_4657279 56.98169956 29.01897285 84.94442628 2.927203065 1.549522831 0.625693589 1

chr1_4663497_4664496 42.50949241 57.03729147 27.98169336 0.490585942 -1.027422202 0.791097116 1

chr1_5490011_5491155 41.02093748 73.04775925 8.994115723 0.123126511 -3.021786667 0.465486374 1

chr1_5631064_5632503 102.5219317 136.0889761 68.95488721 0.506689735 -0.980825494 0.654283035 1

chr1_5656185_5660279 476.5370542 533.3487079 419.7254004 0.78696244 -0.345633315 0.729992365 1

chr1_5719513_5721405 87.98236022 61.03990841 114.924812 1.882781528 0.912865604 0.70367784 1

chr1_5726966_5728738 126.4885817 109.0713118 143.9058516 1.319373988 0.399853567 0.838243962 1

chr1_5747144_5749619 202.0209719 234.1530913 169.8888526 0.725546059 -0.462860893 0.759092276 1

chr1_5785507_5791745 637.7673045 282.1844946 993.3501143 3.520215084 1.81566358 0.045504422 1

chr1_5825296_5827358 194.0000415 194.1269218 193.8731612 0.99869281 -0.00188711 1 1

chr1_6050966_6053528 449.0079443 461.3016029 436.7142857 0.946700126 -0.07902058 0.940597198 1

chr1_6084503_6087714 595.9968574 591.3866536 600.6070611 1.015591166 0.022319751 0.982417127 1

chr1_6107301_6110184 162.450002 86.05626432 238.8437398 2.775436997 1.472714944 0.387823892 1

chr1_6116142_6121840 609.2787438 271.177298 947.3801895 3.49358223 1.804707098 0.050904885 1

chr1_6214785_6220747 1047.003494 1052.688257 1041.318732 0.989199533 -0.015666537 0.984399977 1

chr1_6230637_6232402 85.5003453 86.05626432 84.94442628 0.987080103 -0.018760928 0.99936625 1

chr1_6238735_6240861 184.515409 208.1360811 160.8947368 0.773026647 -0.37140995 0.815159952 1

chr1_6267662_6270983 770.6620353 1018.666013 522.6580582 0.513080884 -0.962741819 0.24350209 1

chr1_6279517_6282058 152.043852 219.1432777 84.94442628 0.387620497 -1.367283233 0.43803471 1

chr1_6294370_6297088 620.9981707 618.404318 623.5920235 1.008388857 0.012052081 0.991258933 1

chr1_6302025_6302973 49.49510542 42.02747792 56.96273292 1.355368814 0.438685482 0.90288501 1

chr1_6319681_6322026 296.4552629 228.1491659 364.7613599 1.598784543 0.67697553 0.586067133 1

chr1_6328706_6331588 326.0510835 404.2643114 247.8378555 0.613058953 -0.705902282 0.552479927 1

chr1_6385554_6386681 44.01047377 60.03925417 27.98169336 0.466056645 -1.101422783 0.770800598 1

chr1_6403692_6405532 123.9817139 96.06280668 151.9006211 1.581263617 0.661077903 0.736436122 1

chr1_6432967_6435208 173.9614499 115.0752372 232.8476626 2.023438477 1.016808984 0.533078505 1

chr1_6444585_6448425 661.9033463 514.3362774 809.4704151 1.573815518 0.654266439 0.453308093 1

chr1_6477974_6481159 480.4873493 461.3016029 499.6730957 1.083180922 0.115274235 0.909531329 1

chr1_6505068_6507519 188.0209689 220.143932 155.8980059 0.708163993 -0.497844604 0.750588357 1

chr1_6530403_6533716 489.5939569 633.4141315 345.7737823 0.545888961 -0.873320571 0.378172506 1

chr1_6549172_6552125 668.4592666 606.3964672 730.522066 1.204693803 0.268666503 0.757472026 1

chr1_6556611_6558391 175.4800899 145.0948643 205.8653154 1.418832545 0.504704328 0.75607444 1

chr1_6570253_6574851 444.3944705 283.1851489 605.603792 2.138543615 1.096628631 0.290416066 1

chr1_6578849_6581366 138.9594803 77.05037619 200.8685845 2.606977336 1.382378041 0.455479109 1

chr1_6612913_6615719 625.0452612 694.45404 555.6364825 0.800105479 -0.321737889 0.718663667 1

chr1_6639124_6641687 435.5220029 469.3068368 401.737169 0.856022409 -0.224279531 0.830362854 1

chr1_6659289_6663924 1633.594202 1778.162578 1489.025825 0.837395772 -0.25601846 0.688147761 1

chr1_6670811_6676275 922.0551351 1006.658162 837.4521085 0.831913097 -0.265495266 0.729956187 1

chr1_6760443_6762872 765.5423482 830.5430161 700.5416802 0.843474289 -0.245584002 0.765990726 1

chr1_6843440_6846806 766.0426753 831.5436703 700.5416802 0.842459278 -0.247321142 0.764316056 1

chr1_6860887_6861876 48.01374474 69.0451423 26.98234717 0.390792839 -1.355524064 0.703175547 1

chr1_6928467_6930295 87.00982894 102.0667321 71.95292579 0.704959631 -0.50438745 0.836160471 1

chr1_6959643_6960708 49.00785875 61.03990841 36.97580909 0.605764492 -0.723171081 0.8378621 1

chr1_6986308_6989175 153.9516353 80.0523389 227.8509317 2.84627451 1.50907481 0.390311277 1

chr1_7018827_7022394 554.3303998 295.1929997 813.4677999 2.755715077 1.46242673 0.124320367 1

chr1_7177235_7178437 65.50164906 68.04448806 62.95881006 0.925259516 -0.112070027 0.974305217 1

chr1_7384891_7388124 281.3944356 120.0785083 442.7103628 3.686840959 1.882385183 0.149448565 1

chr1_7443646_7450949 1142.668001 635.41544 1649.920562 2.596601307 1.376624514 0.05707025 1

chr1_7461917_7463252 53.98039088 24.01570167 83.94508009 3.495424837 1.805467813 0.585255801 1

chr1_7506428_7509617 233.4382448 139.0909388 327.7855508 2.356627639 1.236723822 0.37948052 1

chr1_7528950_7534340 475.2983358 167.1092575 783.4874141 4.688474032 2.229118442 0.03219115 1

chr1_7607990_7612197 383.8960926 225.1472032 542.644982 2.410178649 1.269140087 0.253352522 1

chr1_7653178_7655501 114.9503189 39.02551521 190.8751226 4.89103402 2.2901395 0.278194429 1

chr1_7676808_7678597 65.96665897 15.00981354 116.9235044 7.789803922 2.961587014 0.326043345 1

chr1_7704620_7708216 287.3780863 101.0660779 473.6900948 4.686934576 2.228644657 0.088438346 1

chr1_7739460_7741885 841.0439994 908.5940465 773.4939522 0.851308629 -0.232245841 0.770559932 1

chr1_7763762_7765907 251.0052859 259.1694472 242.8411245 0.936997502 -0.093882894 0.947196992 1

chr1_7842651_7845471 416.5821689 542.354596 290.8097417 0.536198538 -0.899160808 0.398064587 1

chr1_8020492_8023036 674.5554091 759.4965653 589.614253 0.776322475 -0.365272041 0.673023903 1

chr1_8098288_8102184 321.9366287 225.1472032 418.7260542 1.859787945 0.895138133 0.454730232 1

chr1_8106656_8108838 305.3931327 142.0929015 468.6933638 3.298499494 1.721809883 0.168284734 1

chr1_8117232_8124293 1075.439079 982.64246 1168.235698 1.188871584 0.249592891 0.73189256 1

chr1_8128971_8132982 794.4677958 745.487406 843.4481856 1.131405009 0.178115464 0.827110399 1

chr1_8150379_8155349 349.3872559 177.1157998 521.658712 2.945297441 1.558413337 0.182189466 1

chr1_8179582_8182481 245.5481232 319.2087014 171.8875449 0.538480136 -0.893034969 0.512985286 1

chr1_8212714_8217521 537.8967795 380.2486098 695.5449493 1.829184727 0.871200778 0.360176122 1

chr1_8270943_8273598 554.2859263 227.1485116 881.4233409 3.880383519 1.956199249 0.04281235 1

chr1_8295708_8300920 357.9170157 231.1511286 484.6829029 2.096822567 1.068204786 0.349889439 1

chr1_8316384_8322126 774.9707347 730.4775925 819.463877 1.121819321 0.165840336 0.840493553 1

chr1_8438214_8440010 166.5167132 192.1256134 140.907813 0.733415033 -0.447298258 0.789332675 1

chr1_8455127_8456521 82.48922628 66.04317959 98.93527296 1.498039216 0.583075391 0.816475686 1

chr1_8471245_8475011 287.4951563 280.1831861 294.8071265 1.052194211 0.073401018 0.956248749 1

chr1_8482104_8486000 872.0825933 998.6529278 745.5122589 0.746517872 -0.421751295 0.590656741 1

chr1_8520602_8526700 900.9753398 863.5646059 938.3860738 1.086642583 0.119877489 0.878005346 1

chr1_8548768_8551801 191.0163914 216.141315 165.8914678 0.767513919 -0.38173518 0.806544491 1

chr1_8720491_8723169 114.4584941 51.03336605 177.8836221 3.485633731 1.801420979 0.387602129 1

chr1_8739181_8742742 319.9915661 307.2008505 332.7822818 1.083272658 0.115396412 0.925460752 1

chr1_8769502_8775113 716.463855 661.4324502 771.4952598 1.16640068 0.222063466 0.79329476 1

chr1_8793042_8794496 80.51015456 96.06280668 64.95750245 0.676198257 -0.564481797 0.825027514 1

chr1_8802127_8803667 114.5003515 115.0752372 113.9254658 0.990008525 -0.014487146 0.999249887 1

chr1_8847507_8852145 450.5651716 550.3598299 350.7705132 0.637347594 -0.649847698 0.526159525 1

chr1_8875347_8878988 891.0577445 979.6404973 802.4749918 0.81915253 -0.287795982 0.711641631 1

chr1_8907342_8909943 164.4840116 140.0915931 188.8764302 1.348235294 0.431072298 0.798252284 1

chr1_8936411_8941724 1927.153453 2162.413805 1691.893102 0.782409499 -0.354004209 0.561042936 1

chr1_8948777_8953165 452.9523533 380.2486098 525.6560967 1.382401101 0.467176271 0.647800033 1

chr1_8972611_8975751 476.5416323 540.3532876 412.7299771 0.763815057 -0.388704735 0.697720447 1

chr1_9060457_9062900 116.4669968 66.04317959 166.890814 2.526995445 1.337423064 0.512668119 1

chr1_9183829_9190435 2076.211693 2400.569513 1751.853874 0.729765943 -0.454494271 0.446816042 1

chr1_9209652_9215089 431.870596 234.1530913 629.5881006 2.688788336 1.426956189 0.177440225 1

chr1_9233501_9237286 252.4506753 177.1157998 327.7855508 1.850684982 0.888059345 0.509523506 1

chr1_9284730_9286355 90.01440774 112.0732745 67.95554102 0.606349206 -0.72177919 0.760800034 1

chr1_9293066_9296263 590.024979 628.4108604 551.6390977 0.877831897 -0.187983402 0.83791027 1

chr1_9304151_9305674 88.99086272 75.04906772 102.9326577 1.371538126 0.455794727 0.850132721 1

chr1_9329409_9330816 73.49314848 63.04121688 83.94508009 1.331590414 0.41315039 0.880093044 1

chr1_9358689_9364587 633.5632486 730.4775925 536.6489048 0.734654848 -0.444861485 0.61611442 1

chr1_9375599_9377249 119.4905422 105.0686948 133.9123897 1.274522253 0.349956563 0.863343941 1

chr1_9390786_9393713 211.025552 250.1635591 171.8875449 0.687100654 -0.54140664 0.713279005 1

chr1_9397408_9403222 524.4906289 510.3336605 538.6475972 1.055481225 0.077900916 0.937076633 1

chr1_9433562_9434828 51.98562263 30.01962709 73.95161817 2.463442266 1.30067566 0.699368317 1

chr1_9470703_9471719 55.49249061 44.02878639 66.95619483 1.52073678 0.604770462 0.852825525 1

chr1_9487953_9490117 254.5193481 284.1858031 224.8528931 0.791217896 -0.337853038 0.801801813 1

chr1_9524814_9529838 394.3395219 149.0974812 639.5815626 4.28968724 2.100872465 0.061728672 1

chr1_9711458_9712792 113.5081996 126.0824338 100.9339653 0.800539475 -0.32095555 0.878711842 1

chr1_9748602_9750243 136.9849868 114.0745829 159.8953906 1.40167412 0.487150971 0.793750307 1

chr1_9759927_9761723 79.46960496 33.0215898 125.9176201 3.813190731 1.930998695 0.456604452 1

chr1_9778859_9780191 64.50491896 72.04710501 56.96273292 0.790631808 -0.338922097 0.910827282 1

chr1_9882349_9888406 1431.465316 1378.901538 1484.029094 1.076240075 0.105999933 0.873712289 1

chr1_9899475_9907247 1793.968337 1746.141642 1841.795031 1.054779857 0.076941925 0.902075048 1

chr1_9934774_9936007 82.00001754 82.05364737 81.9463877 0.99869281 -0.00188711 1 1

chr1_9937554_9939189 101.5173533 128.0837422 74.95096436 0.585171569 -0.773068419 0.725756409 1

chr1_9949778_9955815 1021.885111 846.5534839 1197.216737 1.414224571 0.50001123 0.499752173 1

chr1_9968720_9972039 693.518788 722.4723586 664.5652173 0.919848641 -0.120531606 0.889188975 1

chr1_10002207_10004651 526.5285626 570.3729147 482.6842105 0.84626075 -0.24082584 0.80229875 1

chr1_10269368_10272580 953.0440233 1020.667321 885.4207257 0.86749199 -0.205077658 0.787572591 1

chr1_10433548_10435834 129.9411658 40.02616945 219.8561621 5.492810458 2.457544509 0.215485382 1

chr1_10446705_10449516 203.9732287 163.1066405 244.8398169 1.501102691 0.586022675 0.695809796 1

chr1_10457999_10461390 757.9877357 739.4834806 776.4919908 1.050046433 0.070453125 0.933492031 1

chr1_10474166_10477108 162.9254763 49.03205758 276.818895 5.645671602 2.497145212 0.155565844 1

chr1_10489605_10491863 411.0157844 435.2845928 386.7469761 0.888492224 -0.170568944 0.874515443 1

chr1_10531414_10533531 362.0040016 368.2407589 355.7672442 0.966126741 -0.049715635 0.967418931 1

chr1_10541858_10543747 85.01767678 112.0732745 57.96207911 0.517180205 -0.951261036 0.697886262 1

chr1_10554574_10556113 102.0078701 114.0745829 89.94115723 0.788441692 -0.342924027 0.87827672 1

chr1_10575173_10577957 318.5383284 377.2466471 259.8300098 0.688753662 -0.53794001 0.654307219 1

chr1_10596150_10597774 71.51538482 95.06215244 47.96861719 0.504602683 -0.986780217 0.717737637 1

chr1_10690850_10697045 1061.688258 1349.882565 773.4939522 0.573008329 -0.803371986 0.273269275 1

chr1_10804096_10807726 223.5428863 289.1890743 157.8966983 0.545998146 -0.873032044 0.541696621 1

chr1_10851004_10858310 2385.653878 2621.714099 2149.593658 0.819919174 -0.286446397 0.619554705 1

chr1_10862933_10865496 365.0406275 427.2793589 302.801896 0.708674289 -0.496805386 0.659401371 1

chr1_10874524_10876529 158.9954558 152.0994439 165.8914678 1.090677675 0.125224808 0.944841341 1

chr1_10895420_10896484 83.99543981 77.05037619 90.94050343 1.180273321 0.23912099 0.926422238 1

chr1_10965804_10971915 666.4710386 622.4069349 710.5351422 1.141592586 0.19104787 0.826896856 1

chr1_11016780_11022162 1393.775968 1051.687602 1735.864335 1.650551296 0.722947975 0.280840238 1

chr1_11038557_11041641 147.4336483 46.03009487 248.8372017 5.405967604 2.434552866 0.188279892 1

chr1_11071318_11074003 859.0538136 941.6156363 776.4919908 0.824637953 -0.278167234 0.724514799 1

chr1_11117710_11124553 1392.588264 1527.999019 1257.177509 0.822760678 -0.281455249 0.673996017 1

chr1_11130752_11133163 350.4251894 236.1543998 464.695979 1.967763377 0.976556747 0.396692148 1

chr1_11158413_11160938 647.0079866 659.4311417 634.5848316 0.9623216 -0.055408983 0.951460567 1

chr1_11193239_11194975 111.4774601 77.05037619 145.904544 1.893625329 0.921150909 0.658693687 1

chr1_11321496_11323885 522.0059978 531.3473994 512.6645962 0.964838817 -0.051640145 0.959130968 1

chr1_11332834_11334805 578.5769714 696.4553484 460.6985943 0.661490497 -0.596207665 0.517467692 1

chr1_11340883_11343701 109.4630712 53.03467452 165.8914678 3.127981255 1.645231867 0.439931279 1

chr1_11379573_11386040 657.3258437 391.2558064 923.3958809 2.360082243 1.238837135 0.160621046 1

chr1_11411451_11416391 537.8091405 246.1609421 829.4573389 3.369573304 1.752565911 0.071268199 1

chr1_11447323_11448613 62.50361049 68.04448806 56.96273292 0.837139562 -0.256459937 0.935447214 1

chr1_11480320_11482580 107.4656868 55.03598299 159.8953906 2.905288176 1.538681272 0.473726597 1

chr1_11538273_11540591 754.4540528 684.4474976 824.460608 1.204563697 0.268510685 0.746129062 1

chr1_11582345_11583367 55.9947798 48.03140334 63.95815626 1.331590414 0.41315039 0.900398421 1

chr1_11622895_11624636 94.97712953 60.03925417 129.9150049 2.163834423 1.113590108 0.627296588 1

chr1_11723614_11725502 347.0020363 350.2289827 343.7750899 0.981572362 -0.026833467 0.984150094 1

chr1_11739986_11742136 341.4951679 334.2185149 348.7718208 1.043544284 0.061491824 0.960280586 1

chr1_11749788_11752412 682.5076672 694.45404 670.5612945 0.965594922 -0.050510006 0.954880388 1

chr1_11767230_11769757 164.465045 111.0726202 217.8574697 1.961396691 0.971881349 0.562924555 1

chr1_11847017_11849042 168.002652 172.1125286 163.8927754 0.952241982 -0.07059986 0.969661966 1

chr1_11862897_11867409 1115.526072 1155.755643 1075.296502 0.930383952 -0.104101883 0.885718087 1

chr1_11967011_11971334 869.1362225 1077.704612 660.5678326 0.612939599 -0.706183183 0.369166262 1

chr1_11984375_11987640 529.523331 565.3696435 493.6770186 0.87319336 -0.195626935 0.83883557 1

chr1_11993659_11995649 404.5089158 418.2734708 390.7443609 0.934183945 -0.098221444 0.929187125 1

chr1_12028989_12030057 51.99281688 41.02682369 62.95881006 1.534576758 0.617840809 0.856173813 1

chr1_12060871_12062624 90.9718965 48.03140334 133.9123897 2.788017429 1.47923958 0.531738086 1

chr1_12078591_12080989 468.5095835 483.3159961 453.7031709 0.938729888 -0.091218001 0.929679638 1

chr1_12185171_12187402 103.9817096 76.04972195 131.9136973 1.734571723 0.794579496 0.714553023 1

chr1_12191033_12194965 284.9510093 210.1373896 359.7646289 1.712044818 0.775720469 0.54062032 1

chr1_12201112_12205710 508.9000434 356.2329081 661.5671788 1.857119777 0.893066866 0.359676115 1

chr1_12289623_12292962 968.0074013 979.6404973 956.3743053 0.976250275 -0.034677045 0.964751434 1

chr1_12415368_12418140 156.4500008 80.0523389 232.8476626 2.90869281 1.54037094 0.376785081 1

chr1_12439197_12442919 361.4585469 298.1949624 424.7221314 1.424310216 0.510263401 0.652242374 1

chr1_12495929_12497149 77.49968956 77.05037619 77.94900294 1.011662847 0.016728569 1 1

chr1_12512897_12515721 227.9902384 213.1393523 242.8411245 1.13935377 0.188215774 0.896319348 1

chr1_12534650_12542763 1748.052696 1829.195944 1666.909447 0.911279873 -0.134033891 0.830776118 1

chr1_12556050_12557373 61.51865279 90.05888126 32.97842432 0.366187364 -1.449346087 0.630894328 1

chr1_12607101_12611325 436.5671306 539.3526333 333.781628 0.618856027 -0.69232428 0.50553853 1

chr1_12714895_12716116 54.02813451 97.06346092 10.99280811 0.113253824 -3.142368333 0.362810335 1

chr1_12801551_12807805 676.8157105 395.2584233 958.3729976 2.424674444 1.277791052 0.143557642 1

chr1_13653740_13656237 150.5258661 190.1243049 110.9274273 0.583446852 -0.777326852 0.658880956 1

chr1_13882762_13883770 46.00851213 59.03859994 32.97842432 0.558590894 -0.84013604 0.819424088 1

chr1_14025529_14027939 789.0021308 792.5181551 785.4861065 0.991126956 -0.012858227 0.989122325 1

chr1_14074667_14077277 651.0648874 750.4906772 551.6390977 0.735037908 -0.444109438 0.61281915 1

chr1_14083154_14089734 805.0263331 845.5528296 764.4998365 0.904142012 -0.145378703 0.858106919 1

chr1_14127199_14129062 93.50819528 106.069349 80.94704151 0.763152053 -0.389957561 0.86828923 1

chr1_14218688_14220731 347.4520037 274.1792607 420.7247466 1.534487858 0.617757231 0.592133282 1

chr1_14225858_14227547 141.0360014 196.1282303 85.94377247 0.438201947 -1.190332199 0.515630296 1

chr1_14227832_14229260 148.5409082 211.1380438 85.94377247 0.40705015 -1.296721544 0.467072701 1

chr1_14439975_14441813 65.47679621 30.01962709 100.9339653 3.362265795 1.749433778 0.548250267 1

chr1_14507942_14510886 118.4519547 45.02944063 191.8744688 4.261089325 2.091222295 0.310667559 1

chr1_14559403_14560382 42.51276253 62.04056265 22.9849624 0.370482817 -1.432521464 0.711648899 1

chr1_14605942_14609711 230.9509977 156.1020609 305.7999346 1.958974359 0.970098514 0.491058112 1

chr1_14617014_14619613 205.9738832 166.1086032 245.8391631 1.47999055 0.565587964 0.704547387 1

chr1_14686794_14688781 200.4761711 164.1072947 236.8450474 1.443232903 0.529304135 0.726533018 1

chr1_14785102_14787869 213.4886003 196.1282303 230.8489702 1.177030812 0.235152088 0.874212263 1

chr1_14790827_14793645 224.4833704 199.130193 249.8365479 1.254639209 0.327272555 0.819706357 1

chr1_14824381_14826825 120.9640546 66.04317959 175.8849297 2.663180828 1.41315039 0.48032876 1

chr1_15159528_15161257 65.96927506 19.01243049 112.9261196 5.939594083 2.570364339 0.38696571 1

chr1_15196883_15198303 85.97908968 54.03532876 117.9228506 2.182328734 1.125868438 0.64386825 1

chr1_15204444_15209945 471.7967 161.105332 782.4880679 4.856996712 2.28006451 0.029266988 1

chr1_15249429_15252575 520.4566188 454.2970233 586.6162144 1.291261409 0.368781096 0.701930106 1

chr1_15262894_15264982 121.9627468 65.04252536 178.8829683 2.750246355 1.459560855 0.46438259 1

chr1_15270665_15274738 233.9248375 119.0778541 348.7718208 2.928939419 1.550378353 0.273490774 1

chr1_15280984_15284662 407.4271637 296.1936539 518.6606734 1.75108638 0.808250253 0.451481034 1

chr1_15294266_15296339 229.994817 222.1452404 237.8443936 1.070670671 0.098514787 0.94706388 1

chr1_15477463_15481614 1074.694147 1371.896958 777.491337 0.566727211 -0.81927362 0.262005747 1

chr1_15554416_15558493 284.1387135 496.3245012 71.95292579 0.144971537 -2.786158418 0.03824134 1

chr1_15573160_15574545 179.5376447 237.155054 121.9202354 0.514095033 -0.959893021 0.549386884 1

chr1_15598456_15599938 108.5252031 147.0961727 69.9542334 0.475568005 -1.072276437 0.612979826 1

chr1_15719701_15721819 139.0412331 202.1321557 75.95031055 0.37574581 -1.412171079 0.445947848 1

chr1_15735297_15738988 979.5803272 1102.720968 856.4396861 0.77666038 -0.364644224 0.627702538 1

chr1_15747414_15754278 1073.47832 1040.680406 1106.276234 1.063031674 0.088184584 0.90460834 1

chr1_15849821_15854633 871.1107161 1040.680406 701.5410264 0.674117647 -0.568927702 0.468478723 1

chr1_15884004_15887343 386.0085848 399.2610403 372.7561294 0.933615083 -0.099100226 0.930132806 1

chr1_15910925_15912024 35.98104105 7.004579654 64.95750245 9.273576097 3.213125781 0.482727385 1

chr1_15925359_15933172 1317.021864 1350.883219 1283.16051 0.949867829 -0.074201314 0.914100868 1

chr1_16009454_16012124 900.073443 1012.662087 787.4847989 0.777638275 -0.362828865 0.639742816 1

chr1_16058833_16063798 326.4271463 215.1406608 437.7136319 2.034546284 1.0247071 0.389901762 1

chr1_16082756_16088031 662.9249292 548.3585215 777.491337 1.417852202 0.503707152 0.563170403 1

chr1_16093375_16095538 163.501016 165.107949 161.894083 0.980534759 -0.028359321 0.990575396 1

chr1_16110707_16116221 460.3866256 287.1877658 633.5854854 2.206171574 1.141544994 0.264124792 1

chr1_16157794_16163845 2209.012899 2229.457638 2188.568159 0.98165945 -0.026705472 0.964544022 1

chr1_16172722_16177820 1993.697614 2296.501472 1690.893756 0.73629117 -0.441651695 0.464444334 1

chr1_16211453_16215621 686.0491984 761.4978738 610.600523 0.801841402 -0.318611183 0.711094133 1

chr1_16264858_16266409 106.9915206 94.06149821 119.921543 1.274926992 0.350414634 0.871874606 1

chr1_16275307_16279311 358.9294424 251.1642133 466.6946714 1.858125667 0.893848076 0.432287315 1

chr1_16329695_16331547 136.0288061 180.1177625 91.93984962 0.510442992 -0.97017825 0.602577502 1

chr1_16338298_16343141 889.4723943 847.5541381 931.3906505 1.09891582 0.136080876 0.862113978 1

chr1_16414013_16416273 182.4807454 153.1000981 211.8613926 1.383809646 0.468645502 0.768535157 1

chr1_16435854_16440125 250.5239254 287.1877658 213.860085 0.7446699 -0.42532705 0.753224982 1

chr1_16469355_16473578 980.4926884 969.6339549 991.351422 1.022397593 0.031956345 0.967473244 1

chr1_16532188_16534123 110.5552885 195.1275761 25.98300098 0.133159041 -2.908777705 0.18827264 1

chr1_16535832_16540231 549.9307913 444.2904809 655.5711016 1.475546134 0.561249029 0.550756477 1

chr1_16543973_16545079 59.01178502 77.05037619 40.97319385 0.531771496 -0.911121646 0.768501775 1

chr1_16572556_16578031 787.2807439 452.2957148 1122.265773 2.481265545 1.31107614 0.11188062 1

chr1_16583363_16585092 58.97188966 16.01046778 101.9333115 6.366666667 2.670538232 0.404405893 1

chr1_16660371_16662424 127.5153968 151.0987897 103.9320039 0.687841406 -0.539852131 0.780537298 1

chr1_16692299_16695244 651.5462479 722.4723586 580.6201373 0.803657234 -0.315347783 0.719546874 1

chr1_16703069_16707001 180.4388875 87.05691855 273.8208565 3.145308392 1.653201478 0.307697241 1

chr1_16717572_16719115 64.98300932 39.02551521 90.94050343 2.330283224 1.220505312 0.674312023 1

chr1_16766336_16768463 485.5187435 514.3362774 456.7012095 0.88794283 -0.171461304 0.8640198 1

chr1_16838846_16842423 1023.087204 1156.756297 889.4181104 0.768889794 -0.379151266 0.608613927 1

chr1_16933519_16936731 505.5305202 552.3611384 458.6999019 0.830434783 -0.268061223 0.783997369 1

chr1_16945723_16953200 1032.135603 475.3107622 1588.960444 3.342992776 1.741140237 0.021378161 1

chr1_16968732_16972703 1350.530701 1397.913968 1303.147434 0.932208608 -0.101275261 0.881543113 1

chr1_16982919_16984300 74.96731492 25.01635591 124.9182739 4.993464052 2.320040985 0.393699411 1

chr1_16991831_16995026 877.5757273 993.6496566 761.5017979 0.766368501 -0.38388983 0.623642911 1

chr1_17012038_17013143 91.01309991 111.0726202 70.9535796 0.638803509 -0.646555856 0.783999788 1

chr1_17019897_17020796 39.51734004 66.04317959 12.99150049 0.19671222 -2.345841511 0.572311169 1

chr1_17027376_17032018 458.4919228 446.2917894 470.6920562 1.054673349 0.07679624 0.941760103 1

chr1_17065455_17068578 849.070162 956.6254499 741.5148741 0.775136052 -0.367478541 0.642663758 1

chr1_17076146_17077560 64.97254496 23.01504743 106.9300425 4.64609264 2.216017921 0.454797383 1

chr1_17092211_17093901 173.4715871 130.0850507 216.8581236 1.667048768 0.73729631 0.651229772 1

chr1_17101073_17103158 125.0111451 142.0929015 107.9293887 0.75956918 -0.396746727 0.84059403 1

chr1_17208342_17211182 216.9634212 161.105332 272.8215103 1.693435635 0.759953153 0.600576088 1

chr1_17220366_17224659 948.562008 1043.682368 853.4416475 0.81772163 -0.290318292 0.702843083 1

chr1_17229176_17233714 1171.554861 1255.821066 1087.288656 0.865799026 -0.207895917 0.769100322 1

chr1_17239440_17242230 937.0302854 983.6431142 890.4174566 0.905224104 -0.143653094 0.851634205 1

chr1_17274314_17275790 397.8378876 150.0981354 645.5776397 4.301037037 2.104684555 0.060271088 1

chr1_17286691_17288299 176.0137721 197.1288845 154.8986597 0.785773531 -0.347814524 0.831226144 1

chr1_17337231_17339477 412.5089175 426.2787046 398.7391304 0.935395379 -0.096351794 0.929925323 1

chr1_17379148_17381853 493.5076268 505.3303893 481.6848643 0.953207791 -0.069137351 0.945951841 1

chr1_17401038_17403008 130.0183404 158.1033693 101.9333115 0.644725738 -0.633242516 0.740554849 1

chr1_17421896_17429863 1802.911439 1668.090612 1937.732265 1.161646886 0.21617159 0.727560058 1

chr1_17432957_17439754 1341.372426 1146.749755 1535.995096 1.339433551 0.421623011 0.533296465 1

chr1_17443732_17447064 732.4318113 628.4108604 836.4527623 1.331060322 0.412575954 0.622361522 1

chr1_17488421_17491199 156.4519628 83.05430161 229.849624 2.767462005 1.46856351 0.398641312 1

chr1_17494333_17496922 121.4519553 48.03140334 194.8725073 4.057189542 2.020480703 0.319438406 1

chr1_17673859_17676345 160.9764896 125.0817795 196.8711997 1.573939869 0.654380425 0.700030323 1

chr1_17701198_17707739 946.7621384 583.3814197 1310.142857 2.245774056 1.167212787 0.128653111 1

chr1_17753301_17756134 580.350026 351.2296369 809.4704151 2.304675716 1.204563768 0.194261873 1

chr1_17763431_17770845 3458.446783 3378.208702 3538.684864 1.04750333 0.06695483 0.900350208 1

chr1_17802123_17804901 149.9836815 125.0817795 174.8855835 1.398169935 0.483539718 0.784850022 1

chr1_17844425_17849555 742.4121928 608.3977756 876.4266099 1.440548676 0.526618409 0.52744304 1

chr1_17862170_17868616 1193.931583 1089.712463 1298.150703 1.191278201 0.252510367 0.719520126 1

chr1_17875993_17881779 565.5115663 583.3814197 547.6417129 0.938736981 -0.0912071 0.923447505 1

chr1_17895036_17897370 230.0092055 244.1596336 215.8587774 0.884088717 -0.177736945 0.901798932 1

chr1_17911564_17917008 771.3624933 561.3670265 981.35796 1.748157469 0.805835144 0.32765992 1

chr1_18229838_18232077 121.9490123 44.02878639 199.8692383 4.539512775 2.182537462 0.283588257 1

chr1_18391117_18391973 50.00720494 61.03990841 38.97450147 0.638508518 -0.647222228 0.853187886 1

chr1_18433952_18436165 150.0301171 196.1282303 103.9320039 0.529918634 -0.916157236 0.603657196 1

chr1_18459491_18460480 34.51341484 55.03598299 13.99084668 0.254212715 -1.975891901 0.663372631 1

chr1_18460838_18461728 43.51080067 60.03925417 26.98234717 0.449411765 -1.153890203 0.762011229 1

chr1_18515935_18518187 92.98301531 67.04383383 118.9221968 1.773797678 0.826841463 0.721753707 1

chr1_18527252_18529631 113.5232421 149.0974812 77.94900294 0.522805632 -0.935653411 0.650263715 1

chr1_18585615_18588871 285.470303 240.1570167 330.7835894 1.377363834 0.461909702 0.715504924 1

chr1_18670196_18674710 264.9366165 168.1099117 361.7633213 2.151945223 1.105641355 0.401813533 1

chr1_18691371_18694137 286.0680795 390.2551521 181.8810069 0.466056645 -1.101422783 0.38599256 1

chr1_18734090_18736494 139.5402522 201.1315015 77.94900294 0.387552434 -1.367536582 0.459168011 1

chr1_18755336_18758803 133.5631417 230.1504743 36.97580909 0.160659278 -2.637923795 0.180077694 1

chr1_18766290_18768237 114.0032945 119.0778541 108.9287349 0.914769045 -0.128520548 0.954097435 1

chr1_18812408_18816886 322.5402914 384.2512267 260.829356 0.67879902 -0.558943614 0.639731661 1

chr1_18861060_18863747 385.9484148 307.2008505 464.695979 1.512678035 0.597104951 0.586757159 1

chr1_18880012_18883094 206.4428172 119.0778541 293.8077803 2.467358708 1.302967472 0.384825624 1

chr1_18932751_18934914 206.9594949 145.0948643 268.8241255 1.852747352 0.889666163 0.549952672 1

chr1_18969543_18970861 89.50623235 99.06476939 79.94769532 0.807024493 -0.309315635 0.899245495 1

chr1_18973520_18977913 263.9542749 194.1269218 333.781628 1.719398962 0.781904341 0.552409185 1

chr1_19027823_19032851 448.9033007 301.1969251 596.6096763 1.980796039 0.986080335 0.338729681 1

chr1_19121719_19126505 919.8236107 650.4252536 1189.221968 1.828376068 0.870562841 0.259439121 1

chr1_19146102_19147730 69.49249361 58.0379457 80.94704151 1.394726166 0.479981898 0.865035986 1

chr1_19226454_19234617 1818.524261 1856.213608 1780.834913 0.959391153 -0.05980896 0.923736276 1

chr1_19239225_19242161 168.9516385 95.06215244 242.8411245 2.554551084 1.353069786 0.416651862 1

chr1_19256660_19260581 678.9288568 570.3729147 787.4847989 1.380649008 0.465346601 0.589732128 1

chr1_19261141_19267284 803.3710024 606.3964672 1000.345538 1.649655946 0.722165166 0.372544813 1

chr1_19321296_19327109 869.0839007 997.6522735 740.5155279 0.742258147 -0.430007072 0.583884226 1

chr1_19354722_19357146 141.989566 126.0824338 157.8966983 1.25232908 0.324613715 0.860195214 1

chr1_19361008_19363721 153.0137672 174.1138371 131.9136973 0.757629029 -0.400436486 0.819771564 1

chr1_19363747_19365644 132.98564 111.0726202 154.8986597 1.394571042 0.479821429 0.800156381 1

chr1_19392115_19397580 912.4187694 788.5155382 1036.322001 1.3142696 0.39426125 0.609216868 1

chr1_19535397_19538460 772.0348283 825.5397449 718.5299117 0.870375916 -0.200289458 0.807886272 1

chr1_19596524_19597391 45.50295283 50.03271181 40.97319385 0.818928105 -0.288191295 0.942333801 1

chr1_19636965_19640332 899.0041164 905.5920838 892.416149 0.985450475 -0.021144726 0.979781534 1

chr1_19669726_19672664 233.005282 241.1576709 224.8528931 0.932389553 -0.100995255 0.945245294 1

chr1_19710532_19714814 351.9804546 322.2106641 381.7502452 1.184784638 0.24462484 0.832138018 1

chr1_19745554_19747664 90.97320454 50.03271181 131.9136973 2.63654902 1.39865082 0.553748836 1

chr1_19751986_19758054 522.4631595 466.3048741 578.6214449 1.240865101 0.311346284 0.74644971 1

chr1_19784849_19785958 87.48595723 66.04317959 108.9287349 1.649356308 0.721903096 0.764779182 1

chr1_19809963_19814113 1453.060481 1546.010795 1360.110167 0.879754638 -0.184826881 0.779861813 1

chr1_19899832_19901779 81.47352952 41.02682369 121.9202354 2.97172007 1.571298223 0.535011586 1

chr1_19979772_19982465 119.9588223 57.03729147 182.880353 3.206329549 1.680922715 0.406249153 1

chr1_19990647_19992224 153.9941467 145.0948643 162.8934292 1.12266847 0.166931955 0.926491148 1

chr1_20096009_20097241 74.48857054 57.03729147 91.93984962 1.611925238 0.688784832 0.796473963 1

chr1_20111867_20114911 253.4552537 185.1210337 321.7894737 1.738265324 0.797648308 0.552475849 1

chr1_20124793_20127877 763.0204379 794.5194636 731.5214122 0.920709241 -0.119182468 0.886183221 1

chr1_20207970_20210135 486.5200517 517.3382401 455.7018633 0.880858649 -0.183017566 0.85466867 1

chr1_20246389_20249971 241.9307254 136.0889761 347.7724746 2.555478662 1.353593545 0.328672708 1

chr1_20303946_20309612 946.8445452 709.4638535 1184.225237 1.669183329 0.739142417 0.332448799 1

chr1_20356676_20360701 268.4402144 177.1157998 359.7646289 2.031239614 1.022360437 0.434656522 1

chr1_20432528_20433984 69.9803943 40.02616945 99.93461915 2.496732026 1.320040985 0.633995177 1

chr1_20506141_20507732 64.97581507 28.01831861 101.9333115 3.638095238 1.86318331 0.5256836 1

chr1_20537125_20540817 709.8856979 535.3500164 884.4213795 1.652043247 0.724251454 0.393758158 1

chr1_20681321_20687083 1358.688322 882.5770364 1834.799608 2.078911565 1.055828388 0.120122935 1

chr1_20702696_20703988 56.47810233 23.01504743 89.94115723 3.907928389 1.966404031 0.541789637 1

chr1_20739238_20741097 102.4584915 39.02551521 165.8914678 4.250846321 2.087750103 0.35143815 1

chr1_20762914_20765561 147.5284815 191.1249591 103.9320039 0.54379085 -0.878876219 0.621614548 1

chr1_20784513_20786723 145.0039551 151.0987897 138.9091206 0.919326494 -0.121350776 0.94962762 1

chr1_20800947_20805295 798.4305174 692.4527315 904.4083033 1.306093921 0.385258645 0.634535903 1

chr1_20809743_20814718 1611.712575 1172.766765 2050.658385 1.748564545 0.806171052 0.209637906 1

chr1_20814885_20821883 519.3990646 365.2387962 673.5593331 1.844161518 0.882965018 0.360905107 1

chr1_20833558_20835467 355.5200237 386.2525352 324.7875122 0.840868299 -0.250048239 0.827612937 1

chr1_20859229_20861124 81.01702191 107.0700033 54.96404053 0.513346772 -0.961994382 0.703145107 1

chr1_20879259_20880485 76.5245422 114.0745829 38.97450147 0.341658067 -1.549374905 0.55594291 1

chr1_20959037_20961334 433.509576 448.2930978 418.7260542 0.934045285 -0.098435598 0.926646505 1

chr1_21013209_21014505 49.48529508 27.01766438 71.95292579 2.663180828 1.41315039 0.685215554 1

chr1_21022482_21029230 739.3415577 497.3251554 981.35796 1.973272314 0.980590063 0.242479215 1

chr1_21043590_21045277 88.48726549 69.0451423 107.9293887 1.563171355 0.644475936 0.788291087 1

chr1_21057942_21060342 206.5029873 211.1380438 201.8679307 0.956094539 -0.064774815 0.968560264 1

chr1_21111829_21115036 1067.076749 1184.774616 949.3788819 0.801316022 -0.319556772 0.661525338 1

chr1_21500517_21504377 785.9622348 728.476284 843.4481856 1.157825181 0.211417439 0.795976736 1

chr1_21563379_21566670 352.9536399 282.1844946 423.7227852 1.50158068 0.586481993 0.608394143 1

chr1_21587251_21590589 214.4781361 181.1184168 247.8378555 1.36837468 0.452463314 0.757180714 1

chr1_21600027_21607675 952.5011847 954.6241414 950.3782281 0.995552267 -0.006431035 0.994806104 1

chr1_21615363_21617746 216.5860502 348.2276742 84.94442628 0.243933589 -2.035439669 0.172738974 1

chr1_21632805_21638197 342.8967378 185.1210337 500.6724419 2.704568098 1.435398223 0.221700576 1

chr1_21694525_21696009 78.4944576 70.04579654 86.94311866 1.241232493 0.311773369 0.906734285 1

chr1_21707219_21708593 72.49707241 68.04448806 76.94965675 1.130872741 0.17743659 0.952165328 1

chr1_21730540_21732611 179.5160619 204.1334642 154.8986597 0.758810714 -0.398188046 0.804706892 1

chr1_21754235_21755762 193.0131217 213.1393523 172.8868911 0.811144865 -0.301968502 0.846272767 1

chr1_21762124_21767801 755.4115415 620.4056265 890.4174566 1.435218216 0.521270107 0.528884873 1

chr1_21829483_21830017 45.50883903 59.03859994 31.97907813 0.541663897 -0.884530159 0.811277369 1

chr1_21864550_21871433 521.3702881 323.2113183 719.5292579 2.226188308 1.154575632 0.23363403 1

chr1_21939701_21943660 512.5664929 614.4017011 410.7312847 0.6685061 -0.580987371 0.54887125 1

chr1_21975934_21980661 1001.390665 834.545633 1168.235698 1.399846397 0.485268532 0.515437718 1

chr1_21991907_21997133 662.8680293 461.3016029 864.4344556 1.873902996 0.906046273 0.30019763 1

chr1_22107518_22112479 1296.010742 1312.858358 1279.163125 0.974334449 -0.037511019 0.957384766 1

chr1_22112615_22116469 547.9497575 471.3081453 624.5913697 1.325229313 0.40624202 0.666410674 1

chr1_22137302_22139840 283.9536252 213.1393523 354.767898 1.664488017 0.735078485 0.562603387 1

chr1_22181499_22183779 166.0327366 216.141315 115.9241582 0.536335028 -0.898793617 0.590545153 1

chr1_22195372_22197993 312.9686739 265.1733726 360.7639751 1.360483413 0.444119368 0.714161828 1

chr1_22221652_22224609 360.5023662 364.238142 356.7665904 0.979487179 -0.029901486 0.981578535 1

chr1_22230161_22231840 120.9928316 110.071966 131.9136973 1.198431373 0.261147296 0.898212189 1

chr1_22309832_22311781 86.46045015 26.01701014 146.9038901 5.646455505 2.497345517 0.319792949 1

chr1_22331820_22335046 147.4441126 62.04056265 232.8476626 3.753152013 1.908102725 0.293793787 1

chr1_22350996_22353558 509.0190755 538.3519791 479.6861719 0.891027043 -0.166458876 0.865252503 1

chr1_22366142_22372641 707.4520807 634.4147858 780.4893756 1.230250923 0.298952598 0.725073299 1

chr1_22448578_22450344 102.9862876 82.05364737 123.9189277 1.510218396 0.594757196 0.786136689 1

chr1_22458210_22459450 78.99805483 76.04972195 81.9463877 1.07753698 0.107737382 0.97135566 1

chr1_22467273_22471890 544.5017515 547.3578672 541.6456358 0.989563991 -0.015135091 0.989369538 1

chr1_22488967_22491294 184.0222761 218.1426235 149.9019287 0.687173952 -0.541252744 0.732398653 1

chr1_22498103_22501065 209.008547 222.1452404 195.8718535 0.881728788 -0.181593132 0.904617422 1

chr1_22521906_22523186 69.99936095 69.0451423 70.9535796 1.027640428 0.039335553 0.99442929 1

chr1_22561744_22567096 802.3997792 649.4245993 955.3749591 1.471109903 0.55690503 0.491267834 1

chr1_22598668_22604302 724.8870092 552.3611384 897.41288 1.624685043 0.700160068 0.405746212 1

chr1_22622361_22625709 227.1216967 413.2701996 40.97319385 0.099143838 -3.334333076 0.030032966 1

chr1_22776587_22781657 961.0361768 1016.664704 905.4076495 0.89056662 -0.167204556 0.825849368 1

chr1_22794910_22801436 874.6430909 1093.71508 655.5711016 0.59939843 -0.738412791 0.346776763 1

chr1_22812462_22813776 58.00262849 62.04056265 53.96469434 0.869829222 -0.201195918 0.953454258 1

chr1_22882355_22884343 122.0078744 134.0876677 109.9280811 0.819822456 -0.286616586 0.887334059 1

chr1_22889190_22890751 93.02160263 126.0824338 59.96077149 0.475568005 -1.072276437 0.64415545 1

chr1_23036070_23038827 306.454611 237.155054 375.754168 1.584424037 0.663958493 0.587371254 1

chr1_23042751_23044595 100.4898841 85.05561008 115.9241582 1.362921953 0.446702949 0.84188048 1

chr1_23075293_23079020 428.9922435 417.2728165 440.7116704 1.056171533 0.078844163 0.942017712 1

chr1_23503549_23504954 172.4964398 167.1092575 177.8836221 1.064474972 0.090142029 0.959445596 1

chr1_23521930_23524608 145.9503255 70.04579654 221.8548545 3.167282913 1.66324574 0.359466835 1

chr1_23669344_23672604 1170.601951 1326.867517 1014.336384 0.764459429 -0.387488158 0.583760194 1

chr1_23692458_23698242 1623.571963 1734.133791 1513.010134 0.872487545 -0.196793559 0.75828649 1

chr1_23703158_23705224 148.0137661 169.1105659 126.9169663 0.750496964 -0.414081859 0.816982396 1

chr1_23726988_23731760 399.9909292 386.2525352 413.7293233 1.071136849 0.099142811 0.928885708 1

chr1_23748300_23750563 109.5140849 131.0857049 87.94246485 0.670877613 -0.575878492 0.78536378 1

chr1_23759490_23762172 163.9424811 76.04972195 251.8352403 3.311455108 1.727465301 0.3118508 1

chr1_23775447_23782370 1489.16971 1749.143605 1229.195816 0.702741509 -0.508933979 0.437608017 1

chr1_23809358_23811605 297.0242623 334.2185149 259.8300098 0.777425541 -0.363223589 0.770602926 1

chr1_23822847_23824491 95.98694008 76.04972195 115.9241582 1.524320605 0.608166372 0.790204597 1

chr1_23832015_23835799 411.4232404 294.1923455 528.6541353 1.796967676 0.845564458 0.429042952 1

chr1_23865547_23866836 80.49903618 79.05168466 81.9463877 1.036617854 0.051884147 0.988999614 1

chr1_23901618_23903055 80.48595574 59.03859994 101.9333115 1.726553672 0.787895183 0.756220469 1

chr1_23910647_23915021 296.0438828 363.2374878 228.8502779 0.630029349 -0.666509059 0.592107682 1

chr1_23945357_23947701 218.4715968 175.1144913 261.8287022 1.495185808 0.58032478 0.688336042 1

chr1_23960744_23962300 96.99021041 82.05364737 111.9267734 1.364068229 0.447915808 0.844800725 1

chr1_23977328_23978547 56.49249083 45.02944063 67.95554102 1.509135802 0.593722635 0.853808481 1

chr1_24051960_24056638 634.4978466 631.4128231 637.5828702 1.009771812 0.014029309 0.989346681 1

chr1_24068910_24072165 1142.058452 1231.805365 1052.31154 0.854283939 -0.227212435 0.750315655 1

chr1_24103654_24106053 494.0426171 559.3657181 428.7195162 0.766438668 -0.383757745 0.696907204 1

chr1_24124254_24129047 1190.874028 998.6529278 1383.095129 1.384960771 0.469845113 0.504116201 1

chr1_24150923_24153561 539.5043665 546.357213 532.6515201 0.97491441 -0.036652528 0.971186716 1

chr1_24156637_24158698 109.4826918 83.05430161 135.911082 1.636412316 0.7105363 0.736224973 1

chr1_24164453_24165474 55.00262785 59.03859994 50.96665577 0.863276836 -0.212104817 0.952588809 1

chr1_24191473_24195608 535.4402715 444.2904809 626.5900621 1.410316199 0.496018657 0.602070647 1

chr1_24246044_24247840 114.0366496 170.1112202 57.96207911 0.340730488 -1.553297051 0.454438827 1

chr1_24305530_24308516 856.5508699 934.6110567 778.4906832 0.832956852 -0.263686331 0.738653578 1

chr1_24419448_24423768 349.4945156 341.2230946 357.7659366 1.048481015 0.068300739 0.955020739 1

chr1_24446011_24447905 81.96535435 29.01897285 134.9117359 4.649087221 2.216947492 0.38784929 1

chr1_24511967_24515119 447.3552298 226.1478574 668.5626021 2.956307479 1.563796329 0.134739365 1

chr1_24526873_24534108 1134.854396 912.5966635 1357.112128 1.487088637 0.572490641 0.423389874 1

chr1_24600432_24601784 65.48268242 39.02551521 91.93984962 2.355890732 1.236272628 0.668851783 1

chr1_24619418_24622892 617.40955 479.3133792 755.5057208 1.576224979 0.656473469 0.464389265 1

chr1_24637219_24638637 85.49118898 72.04710501 98.93527296 1.373202614 0.457544509 0.853252852 1

chr1_24643195_24650875 1402.428685 1293.845927 1511.011442 1.167844957 0.223848755 0.737560823 1

chr1_24698823_24704680 497.1433372 716.4684332 277.8182412 0.387760616 -1.366761814 0.169061056 1

chr1_24710252_24717711 712.3834094 534.3493622 890.4174566 1.666358229 0.73669858 0.385091957 1

chr1_24752471_24755020 90.45260273 18.01177625 162.8934292 9.043718228 3.176916043 0.205832121 1

chr1_24827716_24830813 673.4834665 648.4239451 698.5429879 1.077293634 0.107411533 0.902570878 1

chr1_24841944_24843524 95.49773135 92.06018973 98.93527296 1.074680307 0.103907554 0.968195398 1

chr1_24866343_24869530 282.9601652 222.1452404 343.7750899 1.547523995 0.629961779 0.620261034 1

chr1_24881618_24883673 191.4251554 77.05037619 305.7999346 3.968831169 1.988714192 0.210284125 1

chr1_24891647_24898159 882.9033935 735.4808636 1030.325923 1.400887466 0.486341068 0.53326611 1

chr1_24956258_24961934 530.8575367 313.2047759 748.5102974 2.389843179 1.256915952 0.192340064 1

chr1_25030621_25034081 514.5115554 532.3480537 496.6750572 0.932989336 -0.100067503 0.919274341 1

chr1_25068552_25074021 1169.543089 1235.807982 1103.278195 0.892758593 -0.163657979 0.817609167 1

chr1_25135028_25136458 63.00720772 74.04841348 51.96600196 0.701784137 -0.510900757 0.865135728 1

chr1_25158999_25161628 160.5324084 210.1373896 110.9274273 0.527880486 -0.921716761 0.5879626 1

chr1_25174245_25175698 191.997425 188.1229964 195.8718535 1.041190377 0.058233883 0.973407558 1

chr1_25187755_25190289 197.5141037 219.1432777 175.8849297 0.802602441 -0.317242551 0.836448235 1

chr1_25223206_25224598 76.48333879 51.03336605 101.9333115 1.997385621 0.99811289 0.702855896 1

chr1_25255749_25257795 218.975194 181.1184168 256.8319712 1.418033438 0.503891553 0.727552048 1

chr1_25358961_25361434 122.9575149 58.0379457 187.877084 3.237142213 1.694720747 0.396028323 1

chr1_25406341_25408536 127.4689612 80.0523389 174.8855835 2.184640523 1.127395907 0.560163536 1

chr1_25565139_25567822 545.0373958 602.3938502 487.6809415 0.809571581 -0.304769449 0.747291501 1

chr1_25572395_25575115 877.0577415 965.631338 788.4841451 0.8165478 -0.292390752 0.708857303 1

chr1_25592899_25595103 279.0000597 279.1825319 278.8175874 0.99869281 -0.00188711 1 1

chr1_25663166_25665916 729.0577099 817.534511 640.5809087 0.783552132 -0.351898831 0.675091266 1

chr1_25755737_25758786 922.5620024 1017.665358 827.4586466 0.813095032 -0.298504116 0.697751407 1

chr1_25826416_25828964 343.9745667 305.1995421 382.7495913 1.254096218 0.32664804 0.778684448 1

chr1_25881338_25884998 283.4585302 220.143932 346.7731284 1.575210933 0.65554503 0.605829538 1

chr1_25927339_25929475 115.4826931 89.05822703 141.9071592 1.59341999 0.672126579 0.742502747 1

chr1_25941905_25945751 767.4200464 645.4219824 889.4181104 1.378041242 0.462619066 0.573897356 1

chr1_26007440_26012581 246.9627736 190.1243049 303.8012422 1.597908497 0.676184796 0.618916473 1

chr1_26014672_26017547 315.4173336 189.1236507 441.7110166 2.335567313 1.223773026 0.314048762 1

chr1_26020661_26022490 161.9627554 105.0686948 218.8568159 2.082987862 1.058654432 0.532361801 1

chr1_26067244_26071914 568.3971131 411.2688911 725.525335 1.764114308 0.818944045 0.378437836 1

chr1_26122007_26128008 747.4377007 652.426562 842.4488394 1.291254661 0.368773557 0.657329386 1

chr1_26133974_26138164 578.4343945 478.3127249 678.556064 1.418645227 0.504513847 0.583833006 1

chr1_26144885_26148263 1151.597369 1300.850507 1002.34423 0.770529915 -0.376077127 0.596897448 1

chr1_26184786_26186979 349.506288 359.2348708 339.7777051 0.945837202 -0.080336207 0.946596756 1

chr1_26200508_26203510 523.4749321 485.3173046 561.6325596 1.157248164 0.210698274 0.827295001 1

chr1_26215494_26220860 743.5214147 776.5076873 710.5351422 0.915039418 -0.128094202 0.878844014 1

chr1_26248990_26250055 47.02420888 84.05495584 9.993461915 0.118892001 -3.072276437 0.415961551 1

chr1_26317360_26318750 56.47483222 18.01177625 94.93788819 5.270878722 2.398043497 0.462470308 1

chr1_26323306_26325923 506.0066484 516.3375859 495.675711 0.959983787 -0.058918055 0.953735922 1

chr1_26338251_26341283 260.5029988 265.1733726 255.832625 0.964774941 -0.051735659 0.971800742 1

chr1_26371833_26374113 188.5134478 209.1367354 167.8901602 0.802776996 -0.316928819 0.84058936 1

chr1_26401874_26408662 854.0668929 956.6254499 751.508336 0.785582629 -0.348165066 0.65957321 1

chr1_26419714_26425547 480.009913 495.3238469 464.695979 0.938165973 -0.092084919 0.928188989 1

chr1_26450712_26455770 888.9328258 786.5142297 991.351422 1.260436728 0.333923699 0.668118398 1

chr1_26487479_26489099 257.5448556 326.213281 188.8764302 0.578996752 -0.78837284 0.553954076 1

chr1_26490158_26491602 162.4827032 136.0889761 188.8764302 1.387889273 0.472892473 0.780290555 1

chr1_26495606_26497815 544.0295474 589.3853452 498.6737496 0.846091193 -0.241114928 0.799327848 1

chr1_26503286_26506162 189.017699 216.141315 161.894083 0.749019608 -0.416924609 0.789975071 1

chr1_26559711_26562256 516.0158069 540.3532876 491.6783262 0.909920116 -0.136188201 0.889330311 1

chr1_26614589_26618881 457.3133745 172.1125286 742.5142203 4.314120687 2.109066536 0.045305405 1

chr1_26632156_26634105 303.007259 314.2054302 291.8090879 0.928720703 -0.1066833 0.933196449 1

chr1_26662224_26664649 367.0092348 381.249264 352.7692056 0.925298063 -0.112009924 0.922625654 1

chr1_26678243_26685111 933.9799251 903.5907753 964.3690748 1.067263081 0.093915845 0.903340435 1

chr1_26757747_26759858 333.0406206 395.2584233 270.8228179 0.685179118 -0.545446911 0.642851123 1

chr1_26797493_26800871 828.5475938 901.5894669 755.5057208 0.837970882 -0.255027981 0.75000962 1

chr1_26812408_26814975 188.959491 127.083088 250.8358941 1.973794452 0.980971758 0.529803663 1

chr1_26826057_26829197 1236.108178 1401.916585 1070.299771 0.763454675 -0.389385585 0.575119198 1

chr1_26855364_26859704 964.960965 905.5920838 1024.329846 1.131116167 0.177747103 0.814703554 1

chr1_26881212_26883670 94.46241392 37.02420674 151.9006211 4.102738032 2.036587038 0.385273217 1

chr1_26945813_26948954 1176.102279 1332.871443 1019.333115 0.764764765 -0.38691204 0.583734612 1

chr1_26962871_26964211 143.0013386 145.0948643 140.907813 0.971142664 -0.042244847 0.985556764 1

chr1_27003646_27004663 53.50230052 57.03729147 49.96730957 0.876046325 -0.190920934 0.958913845 1

chr1_27017398_27025905 3433.76594 3841.511613 3026.020268 0.787716028 -0.344252464 0.518645143 1

chr1_27144292_27145704 48.01309071 68.04448806 27.98169336 0.411226451 -1.281995029 0.718466248 1

chr1_27150048_27154661 805.9785896 773.5057246 838.4514547 1.08396283 0.116315287 0.886547808 1

chr1_27174042_27176378 109.4748436 71.04645077 147.9032363 2.081782196 1.057819137 0.615958023 1

chr1_27187957_27193146 1250.804715 952.6228329 1548.986597 1.626022958 0.701347627 0.311847167 1

chr1_27215389_27217902 847.5148968 870.5691855 824.460608 0.947036286 -0.078508391 0.92233652 1

chr1_27225377_27227942 413.5148039 436.285247 390.7443609 0.895616718 -0.159046637 0.88277296 1

chr1_27239058_27242472 734.2885809 411.2688911 1057.308271 2.570844266 1.362242219 0.108165524 1

chr1_27247276_27250283 532.4971708 528.3454367 536.6489048 1.015715983 0.022497049 0.983308958 1

chr1_27271653_27277192 1189.434525 1089.712463 1289.156587 1.183024541 0.242480002 0.730580544 1

chr1_27284818_27288514 1344.040837 1406.919856 1281.161817 0.910614639 -0.135087442 0.842285556 1

chr1_27319780_27321793 337.524598 375.2453386 299.8038574 0.798954248 -0.323815204 0.782484659 1

chr1_27357560_27358869 92.00394381 98.06411515 85.94377247 0.876403895 -0.190332199 0.938857781 1

chr1_27437878_27439811 129.9869474 110.071966 149.9019287 1.361853832 0.445571867 0.816781227 1

chr1_27448350_27449643 90.51277279 110.071966 70.9535796 0.644610814 -0.633499704 0.789025721 1

chr1_27456254_27458493 170.98434 147.0961727 194.8725073 1.324796585 0.405770859 0.806075448 1

chr1_27477904_27483355 1135.998281 1133.74125 1138.255312 1.003981563 0.005732776 0.994991191 1

chr1_27518436_27519782 68.52127038 101.0660779 35.97646289 0.355969715 -1.490173591 0.596752273 1

chr1_27559886_27562692 394.5095677 409.2675826 379.7515528 0.927880851 -0.107988534 0.922835273 1

chr1_27624776_27627683 283.0314536 331.2165522 234.846355 0.709041723 -0.49605757 0.696573233 1

chr1_27647744_27650187 590.5331544 641.4193654 539.6469434 0.841332477 -0.249252059 0.785514117 1

chr1_27666987_27670898 967.1565182 1206.789009 727.5240274 0.602859342 -0.730106661 0.334692287 1

chr1_27674929_27676781 227.508878 241.1576709 213.860085 0.886806064 -0.173309459 0.9048749 1

chr1_27690848_27694692 849.9432818 763.4991823 936.3873814 1.226441892 0.294478881 0.71011102 1

chr1_27705740_27707067 73.48987837 58.0379457 88.94181104 1.532476899 0.615865326 0.81960766 1

chr1_27718215_27720054 174.9758386 138.0902846 211.8613926 1.534223738 0.617508888 0.703841013 1

chr1_27724709_27726277 99.00198324 102.0667321 95.93723438 0.939946175 -0.089349951 0.972649581 1

chr1_27744451_27745781 69.48399132 45.02944063 93.938542 2.086158315 1.060848646 0.702786807 1

chr1_27762396_27763392 62.50230245 66.04317959 58.9614253 0.892770846 -0.16363818 0.961074159 1

chr1_27814342_27818105 1120.509069 1134.741904 1106.276234 0.97491441 -0.036652528 0.960506055 1

chr1_27829941_27832515 221.0177059 248.1622506 193.8731612 0.781235505 -0.356170578 0.805366052 1

chr1_27880007_27885316 513.9752571 476.3114165 551.6390977 1.158147965 0.211819584 0.827732378 1

chr1_27924112_27931425 1205.928969 1097.717697 1314.140242 1.197156833 0.259612164 0.711041909 1

chr1_27985011_27987029 158.4637357 103.0673863 213.860085 2.074953995 1.05307935 0.539274677 1

chr1_27998147_28000056 142.9915283 130.0850507 155.8980059 1.198431373 0.261147296 0.887702825 1

chr1_28009080_28014542 1063.301732 760.4972195 1366.106244 1.796332989 0.84505481 0.249190232 1

chr1_28051414_28053753 412.9706573 368.2407589 457.7005557 1.242938335 0.313754722 0.768988522 1

chr1_28099244_28100977 350.4925537 339.2217861 361.7633213 1.06645073 0.092817314 0.937780579 1

chr1_28156190_28158848 657.5004676 658.4304875 656.5704478 0.99717504 -0.004081323 0.998265355 1

chr1_28189219_28192114 156.4643893 102.0667321 210.8620464 2.065923363 1.046786737 0.544433288 1

chr1_28239999_28242077 383.032783 433.2832843 332.7822818 0.76804782 -0.380731957 0.730181932 1

chr1_28285693_28287496 220.0379803 278.1818777 161.894083 0.581972069 -0.780978179 0.587899407 1

chr1_28413583_28416336 523.4952068 516.3375859 530.6528277 1.027724578 0.039453686 0.969271389 1

chr1_28478725_28480983 123.985638 102.0667321 145.904544 1.429501474 0.515512107 0.793630671 1

chr1_28519656_28521525 128.9967575 124.0811253 133.9123897 1.079232553 0.110005771 0.958060086 1

chr1_28558290_28560599 466.4834222 441.2885182 491.6783262 1.114187897 0.15599255 0.878575025 1

chr1_28561147_28564106 800.4802235 770.5037619 830.4566851 1.077810033 0.108102922 0.894911743 1

chr1_28581854_28589299 1344.358692 1128.737978 1559.979405 1.382056274 0.46681636 0.49011191 1

chr1_28630625_28635193 380.4415464 291.1903827 469.69271 1.613009007 0.689754494 0.532916526 1

chr1_28665720_28667980 112.4624178 55.03598299 169.8888526 3.086868687 1.626144113 0.437978984 1

chr1_28694539_28697659 1058.993686 1049.686294 1068.301079 1.017733665 0.025360066 0.973700746 1

chr1_28742657_28745909 333.9202807 212.1386981 455.7018633 2.148131706 1.10308245 0.350195841 1

chr1_28763460_28765346 110.0026396 114.0745829 105.9306963 0.928609104 -0.106856669 0.963592957 1

chr1_28843727_28849461 1069.439732 977.6391888 1161.240275 1.187800456 0.248292492 0.733736778 1

chr1_28878092_28880911 580.4834466 555.3631011 605.603792 1.090464582 0.124942913 0.893284507 1

chr1_28905897_28910307 987.0204858 1018.666013 955.3749591 0.93786869 -0.092542148 0.902815778 1

chr1_28967735_28971464 1034.514937 1057.691528 1011.338346 0.956175141 -0.064653196 0.931303436 1

chr1_28973560_28976182 431.0262531 471.3081453 390.7443609 0.829063458 -0.270445562 0.796619414 1

chr1_28993593_28996827 960.48876 943.6169448 977.3605753 1.035759882 0.050689585 0.94792085 1

chr1_29101382_29102699 86.53435467 139.0909388 33.97777051 0.244284572 -2.033365341 0.410528289 1

chr1_29138253_29139629 92.52454562 130.0850507 54.96404053 0.422523881 -1.242895209 0.594175562 1

chr1_29240017_29242230 369.4912497 356.2329081 382.7495913 1.074436366 0.103580041 0.928405666 1

chr1_29252507_29255814 300.9085012 161.105332 440.7116704 2.735549872 1.451830858 0.245211495 1

chr1_29450607_29451723 71.00459334 78.05103043 63.95815626 0.819440255 -0.287289328 0.920104635 1

chr1_29527908_29530407 197.4735543 157.1027151 237.8443936 1.513941967 0.598309905 0.694651574 1

chr1_29543979_29545949 84.4715681 41.02682369 127.9163125 3.117870238 1.640560886 0.508531476 1

chr1_29562557_29566996 967.6247982 1158.757606 776.4919908 0.670107352 -0.577535859 0.444655829 1

chr1_29573856_29576742 129.4728858 88.05757279 170.8881987 1.940641711 0.956533787 0.617462052 1

chr1_29585350_29586968 121.5049312 129.0843965 113.9254658 0.882565739 -0.180224351 0.930948849 1

chr1_29596437_29600517 559.0674839 662.4331044 455.7018633 0.687921332 -0.539684502 0.563460781 1

chr1_30265948_30266862 53.99674144 49.03205758 58.9614253 1.20250767 0.266046096 0.939726558 1

chr1_30416805_30418308 97.51408233 119.0778541 75.95031055 0.637820618 -0.648777359 0.774238414 1

chr1_30532441_30536524 194.9110947 59.03859994 330.7835894 5.602835937 2.486157248 0.120432167 1

chr1_30556323_30557233 39.50818373 52.03402028 26.98234717 0.518552036 -0.947439326 0.81711793 1

chr1_30560620_30563404 139.9758311 103.0673863 176.8842759 1.716200267 0.779217913 0.670723456 1

chr1_30666386_30669409 175.5696909 282.1844946 68.95488721 0.244361007 -2.032914005 0.221472066 1

chr1_30775668_30777218 58.52911651 103.0673863 13.99084668 0.135744654 -2.881032715 0.37447015 1

chr1_30844865_30847100 118.5343615 171.1118744 65.95684864 0.385460383 -1.375345505 0.496786771 1

chr1_30851706_30853849 339.6181236 520.3402028 158.8960444 0.305369532 -1.711371967 0.149956183 1

chr1_30961723_30964108 182.0085412 195.1275761 168.8895064 0.865533769 -0.208337987 0.898083725 1

chr1_31091321_31092275 33.01243348 52.03402028 13.99084668 0.268878834 -1.894971906 0.685811717 1

chr1_31124129_31125225 48.0157068 72.04710501 23.9843086 0.332897603 -1.58684961 0.656358492 1

chr1_31173059_31175077 111.0229145 146.0955185 75.95031055 0.51986749 -0.943784155 0.651646851 1

chr1_31179233_31181371 116.0301098 162.1059863 69.9542334 0.43153393 -1.212454095 0.552895222 1

chr1_31191399_31193233 611.1302811 810.5299314 411.7306309 0.507977084 -0.97716468 0.280057998 1

chr1_31207788_31209384 93.50688723 104.0680406 82.94573389 0.797033685 -0.327287396 0.890120834 1

chr1_31349085_31355028 764.571779 874.5718025 654.5717554 0.748448273 -0.418025483 0.611891611 1

chr1_31377171_31383245 757.6201751 941.6156363 573.6247139 0.609192001 -0.715031096 0.38784873 1

chr1_31497624_31499403 90.51277279 110.071966 70.9535796 0.644610814 -0.633499704 0.789025721 1

chr1_31542542_31545623 252.9568886 187.1223422 318.7914351 1.703652441 0.768631044 0.567329003 1

chr1_31573436_31574704 69.49445567 61.03990841 77.94900294 1.277017036 0.352777772 0.90208065 1

chr1_31583387_31585509 162.9915326 150.0981354 175.8849297 1.171799564 0.228725819 0.894478027 1

chr1_31623954_31629591 1324.769413 1737.135754 912.4030728 0.52523418 -0.928967292 0.173869936 1

chr1_31641485_31648355 1146.588865 1282.838731 1010.339 0.78758068 -0.344500374 0.628585295 1

chr1_31687620_31689782 147.484662 124.0811253 170.8881987 1.377229602 0.461769095 0.796336963 1

chr1_31710829_31713861 842.6430841 1061.694145 623.5920235 0.587355621 -0.767693832 0.33480497 1

chr1_31859274_31864905 695.0060348 704.4605823 685.5514874 0.973158051 -0.039253962 0.965084155 1

chr1_31884093_31892244 2787.88091 3371.204122 2204.557698 0.653937768 -0.612774747 0.271966445 1

chr1_31903845_31905441 359.6187819 541.3539418 177.8836221 0.328590241 -1.605638462 0.164214449 1

chr1_31925886_31928605 107.0556148 192.1256134 21.98561621 0.114433551 -3.127417992 0.168789062 1

chr1_31987933_31990677 114.5546354 198.1295388 30.97973194 0.156360996 -2.677047419 0.21235558 1

chr1_31996224_31997890 68.5336968 120.0785083 16.98888526 0.141481481 -2.821314864 0.335253711 1

chr1_32083195_32084287 52.48071757 23.01504743 81.9463877 3.560556976 1.832102939 0.587027055 1

chr1_32091887_32094505 163.9915328 151.0987897 176.8842759 1.170653162 0.227313701 0.894788708 1

chr1_32108930_32111789 1259.201708 1568.025188 950.3782281 0.606098828 -0.722375043 0.29664147 1

chr1_32115040_32118014 148.5409082 211.1380438 85.94377247 0.40705015 -1.296721544 0.467072701 1

chr1_32153781_32155977 220.5134546 241.1576709 199.8692383 0.828790714 -0.270920256 0.852277747 1

chr1_32201157_32203193 153.0046109 160.1046778 145.904544 0.91130719 -0.133990646 0.942048243 1

chr1_32278602_32282062 900.4096105 762.498528 1038.320693 1.361734685 0.445445642 0.565430246 1

chr1_32294191_32298752 685.0544304 768.5024534 601.6064073 0.782829521 -0.353229934 0.681392781 1

chr1_32319144_32323459 604.1649428 856.5600262 351.7698594 0.410677417 -1.283922477 0.160293246 1

chr1_32342933_32348324 637.6175334 817.534511 457.7005557 0.559854721 -0.83687559 0.345629051 1

chr1_32361384_32366788 884.2264808 1230.804711 537.648251 0.436826611 -1.194867347 0.129341682 1

chr1_32419761_32422773 255.5265426 296.1936539 214.8594312 0.725401872 -0.463147626 0.729332575 1

chr1_32428646_32430754 161.015731 185.1210337 136.9104282 0.739572514 -0.435236487 0.798627653 1

chr1_32442206_32444268 142.9856421 121.0791626 164.8921216 1.361853832 0.445571867 0.806772461 1

chr1_32477675_32482828 1778.804174 2244.467452 1313.140896 0.585056778 -0.773351454 0.215662736 1

chr1_32526835_32529863 382.4382767 288.18842 476.6881333 1.654084967 0.726033345 0.510687452 1

chr1_32572434_32575507 1158.72425 1501.982009 815.4664923 0.542926938 -0.881170029 0.216378584 1

chr1_32598181_32599905 73.01571215 97.06346092 48.96796338 0.504494306 -0.987090108 0.714071773 1

chr1_32643940_32647657 1256.708575 1576.030422 937.3867276 0.594777052 -0.74957911 0.279293515 1

chr1_32650650_32652890 145.9490175 68.04448806 223.8535469 3.289811611 1.718004971 0.344570646 1

chr1_32664895_32667196 258.0615333 352.2302912 163.8927754 0.465300059 -1.103766724 0.408639038 1

chr1_32670226_32672583 715.147308 940.6149821 489.6796338 0.520595188 -0.941766117 0.26747963 1

chr1_32686547_32690069 1077.212134 1401.916585 752.5076822 0.536770654 -0.897622295 0.219275709 1

chr1_32704388_32708646 1809.302545 2272.485771 1346.11932 0.592355445 -0.755464965 0.22418155 1

chr1_32738251_32741852 230.4787936 198.1295388 262.8280484 1.326546511 0.40767526 0.772782607 1

chr1_32754731_32759427 1125.10096 1279.836768 970.3651519 0.758194464 -0.399360173 0.577360588 1

chr1_32774189_32776065 93.01833252 121.0791626 64.95750245 0.536487873 -0.898382534 0.698648705 1

chr1_32786697_32788662 133.5382889 192.1256134 74.95096436 0.390114379 -1.35803092 0.473011189 1

chr1_32827083_32828433 54.97712098 20.01308472 89.94115723 4.494117647 2.168037892 0.511001556 1

chr1_32858560_32861765 1011.692826 1306.854433 716.5312193 0.548286941 -0.866996982 0.245355729 1

chr1_32965217_32967214 576.5913594 716.4684332 436.7142857 0.609537372 -0.714213417 0.439169994 1

chr1_33004249_33006738 608.5652054 708.4631993 508.6672115 0.71798678 -0.477970814 0.596149339 1

chr1_33077108_33078764 177.5101753 193.1262676 161.894083 0.838281012 -0.254494144 0.87651685 1

chr1_33101564_33104197 216.4715963 173.1131829 259.8300098 1.500925611 0.585852476 0.686912769 1

chr1_33114606_33118949 1759.294032 2209.444554 1309.143511 0.59252155 -0.75506047 0.228021689 1

chr1_33166725_33170353 542.6299395 741.4847891 343.7750899 0.46363067 -1.108952087 0.244248324 1

chr1_33189448_33192515 567.0655235 667.4363756 466.6946714 0.699234696 -0.516151321 0.578332476 1

chr1_33236577_33240410 347.5762679 464.3035656 230.8489702 0.49719405 -1.008119063 0.38363217 1

chr1_33280793_33285463 2134.296075 2587.691855 1680.900294 0.649575138 -0.622431679 0.294772915 1

chr1_33335729_33337140 89.52192889 123.0804711 55.96338672 0.45468941 -1.137046693 0.63236121 1

chr1_33365093_33368810 967.0871918 1100.71966 833.4547237 0.757190731 -0.401271345 0.595216481 1

chr1_33428307_33431763 856.6496273 1085.709846 627.5894083 0.57804524 -0.790745688 0.317686111 1

chr1_33445663_33448389 170.5186761 199.130193 141.9071592 0.712635071 -0.488764611 0.767151001 1

chr1_33456916_33460018 263.0674206 366.2394505 159.8953906 0.436587021 -1.195658853 0.367022859 1

chr1_33489637_33494379 395.0354017 449.2937521 340.7770513 0.758472714 -0.398830815 0.71396808 1

chr1_33500502_33503568 699.132916 902.5901211 495.675711 0.549170326 -0.864674422 0.312334013 1

chr1_33515763_33517258 106.0209514 138.0902846 73.95161817 0.535530927 -0.900958201 0.67477019 1

chr1_33545854_33547686 308.5474826 381.249264 235.8457012 0.618612869 -0.692891248 0.569977785 1

chr1_33591770_33594334 789.6587693 1032.675172 546.6423667 0.529345899 -0.917717342 0.261489148 1

chr1_33645588_33649544 709.1473067 934.6110567 483.6835567 0.517523897 -0.950302612 0.264801564 1

chr1_33699779_33701259 99.97778463 66.04317959 133.9123897 2.027649039 1.019807962 0.646459961 1

chr1_33708392_33712325 415.0707232 523.3421656 306.7992808 0.58623077 -0.7704594 0.469053219 1

chr1_33720146_33727612 2846.483277 3586.344783 2106.621772 0.587400794 -0.76758288 0.167706444 1

chr1_33792063_33795697 337.9726033 296.1936539 379.7515528 1.282105635 0.358515133 0.759468695 1

chr1_33802862_33809874 1035.994989 1028.672555 1043.317424 1.014236667 0.020394338 0.979330466 1

chr1_33875407_33876621 84.00132601 86.05626432 81.9463877 0.952241982 -0.07059986 0.982250807 1

chr1_33895109_33896938 532.0426252 597.390579 466.6946714 0.781222014 -0.356195491 0.709086273 1

chr1_33937855_33939039 73.99739974 70.04579654 77.94900294 1.112829132 0.154232092 0.958561725 1

chr1_34124498_34127271 163.0706693 271.177298 54.96404053 0.202686733 -2.302676437 0.186932318 1

chr1_34134512_34135647 31.5193004 61.03990841 1.998692383 0.032744027 -4.932624447 0.369638332 1

chr1_34408029_34414052 309.6567046 549.3591757 69.9542334 0.127337881 -2.973266432 0.022315932 1

chr1_34426226_34430103 215.1092677 382.2499182 47.96861719 0.125490196 -2.994353437 0.054161131 1

chr1_34485707_34486718 31.51864637 60.03925417 2.998038574 0.049934641 -4.323815204 0.415478239 1

chr1_34628602_34631167 176.0765583 293.1916912 58.9614253 0.201101965 -2.314000915 0.167501254 1

chr1_34870061_34875192 1024.092436 1165.762185 882.4226871 0.756949143 -0.401731721 0.587301446 1

chr1_34890348_34892503 131.9908719 118.0771999 145.904544 1.235670765 0.3052944 0.874082303 1

chr1_35162143_35163576 59.01832525 87.05691855 30.97973194 0.355856059 -1.490634295 0.630754092 1

chr1_35219967_35222091 107.5546339 191.1249591 23.9843086 0.125490196 -2.994353437 0.183975898 1

chr1_35228111_35230246 104.0490739 179.1171083 28.98103955 0.161799394 -2.627721892 0.245600437 1

chr1_35242267_35244263 86.54024088 148.096827 24.98365479 0.16869811 -2.567484285 0.307354354 1

chr1_35246455_35248728 139.5579108 228.1491659 50.96665577 0.223391813 -2.162351782 0.252059818 1

chr1_35251045_35255535 271.6115689 442.2891724 100.9339653 0.228208086 -2.131578186 0.111514779 1

chr1_35308383_35311632 480.6717836 743.4860975 217.8574697 0.293021578 -1.770921185 0.082413885 1

chr1_35315904_35318245 249.0392946 309.202159 188.8764302 0.610850942 -0.711107713 0.599423229 1

chr1_35330757_35332988 504.1459548 727.4756298 280.8162798 0.38601469 -1.373272343 0.164581994 1

chr1_35394208_35396035 190.5873527 324.2119725 56.96273292 0.175695957 -2.508847098 0.121662112 1

chr1_35410556_35413772 253.1262804 446.2917894 59.96077149 0.134353293 -2.895896414 0.042629055 1

chr1_35449588_35451481 208.0569444 295.1929997 120.9208892 0.409633322 -1.287595017 0.388422625 1

chr1_35495842_35498358 817.183301 1097.717697 536.6489048 0.488876973 -1.032456642 0.201558472 1

chr1_35544020_35546330 723.1603901 968.6333007 477.6874795 0.493156161 -1.019883539 0.228409867 1

chr1_35654863_35660232 1731.770155 2145.402683 1318.137627 0.614401034 -0.702747451 0.263661711 1

chr1_35784301_35787495 266.5141185 288.18842 244.8398169 0.849582426 -0.235174172 0.858996164 1

chr1_35904439_35906849 188.9804198 159.1040236 218.8568159 1.375558022 0.460016995 0.768577324 1

chr1_35916016_35921095 1009.449529 932.6097482 1086.28931 1.164784426 0.220062971 0.767774729 1

chr1_35933772_35936185 111.533706 163.1066405 59.96077149 0.367616985 -1.443724668 0.491389821 1

chr1_35940104_35943171 311.569066 417.2728165 205.8653154 0.493359038 -1.019290156 0.402731565 1

chr1_35968586_35975972 1539.337805 2056.344455 1022.331154 0.497159487 -1.008219358 0.122865201 1

chr1_35988245_35991367 282.5939126 426.2787046 138.9091206 0.325864556 -1.617655657 0.210905036 1

chr1_36003214_36007910 494.5632189 591.3866536 397.7397842 0.672554549 -0.572276809 0.560905449 1

chr1_36008890_36011924 192.0078893 204.1334642 179.8823145 0.881199539 -0.182459355 0.908492433 1

chr1_36018651_36025694 2652.878265 3232.113183 2073.643347 0.641575103 -0.640309938 0.256370257 1

chr1_36038427_36039691 82.52846762 126.0824338 38.97450147 0.309119203 -1.693764814 0.501453086 1

chr1_36105678_36108538 995.708519 1314.859666 676.5573716 0.514547209 -0.958624646 0.202149204 1

chr1_36172554_36174619 433.5148082 456.2983317 410.7312847 0.900137599 -0.15178254 0.885749864 1

chr1_36183008_36185816 1085.666681 1340.876677 830.4566851 0.619338601 -0.691199728 0.34153386 1

chr1_36211192_36215683 470.0197212 500.3271181 439.7123243 0.878849673 -0.186311681 0.854272362 1

chr1_36233438_36236303 512.0772841 630.4121688 393.7423994 0.624579313 -0.679043308 0.483952627 1

chr1_36348073_36350086 674.5750298 789.5161924 559.6338672 0.708831399 -0.496485583 0.566083434 1

chr1_36395325_36398178 1250.713152 1577.031076 924.3952271 0.586161707 -0.770629374 0.266881367 1

chr1_36489509_36490646 65.50034102 66.04317959 64.95750245 0.983561101 -0.023913416 0.999461197 1

chr1_36490989_36492191 66.99870628 65.04252536 68.95488721 1.06015083 0.084269534 0.981840213 1

chr1_36553168_36556274 1155.781804 1587.037619 724.5259888 0.456527294 -1.13122698 0.114465971 1

chr1_36561903_36566202 221.5703548 329.2152437 113.9254658 0.346051612 -1.530940869 0.292399449 1

chr1_36652851_36654842 177.5454925 247.1615964 107.9293887 0.436675399 -1.195366839 0.460023647 1

chr1_36687341_36692318 1281.796219 1735.134446 828.4579928 0.477460404 -1.066547001 0.123278352 1

chr1_36702989_36706648 313.9719442 271.177298 356.7665904 1.315621156 0.395744113 0.743972633 1

chr1_36720345_36726496 1070.558764 1160.758914 980.3586139 0.844584178 -0.243686873 0.738421999 1

chr1_36770570_36773873 924.6921533 1218.79686 630.5874468 0.517385192 -0.950689332 0.217846348 1

chr1_36788251_36790547 433.5880587 568.3716062 298.8045113 0.525720335 -0.927632555 0.375015449 1

chr1_36824476_36827450 176.4709338 132.0863592 220.8555083 1.672053872 0.74162133 0.646325453 1

chr1_36836936_36844849 1709.186108 1994.303893 1424.068323 0.714067865 -0.4858669 0.440387507 1

chr1_36848353_36855834 2861.345936 3391.217207 2331.474665 0.687503785 -0.540560439 0.329429947 1

chr1_36871964_36875814 294.6481991 521.3408571 67.95554102 0.130347622 -2.939563831 0.027112699 1

chr1_36885836_36887580 166.5461442 237.155054 95.93723438 0.404533797 -1.305667858 0.436225301 1

chr1_36891386_36892906 90.51865899 119.0778541 61.95946387 0.520327347 -0.942508562 0.689479481 1

chr1_36914950_36917341 681.6195048 864.5652601 498.6737496 0.576791334 -0.793878606 0.357953336 1

chr1_36978869_36984315 431.0942714 575.3761858 286.812357 0.498477977 -1.004398329 0.338588858 1

chr1_36999955_37001385 76.03010128 122.0798168 29.98038574 0.245580199 -2.025733851 0.448404688 1

chr1_37045263_37048345 192.5592302 283.1851489 101.9333115 0.359952886 -1.47412001 0.344617794 1

chr1_37156980_37158201 67.01113271 84.05495584 49.96730957 0.594460006 -0.750348343 0.7926783 1

chr1_37168981_37172568 317.6266212 511.3343147 123.9189277 0.242344244 -2.04487028 0.098994036 1

chr1_37229311_37232595 282.0805051 405.2649657 158.8960444 0.3920794 -1.350782252 0.293357718 1

chr1_37235813_37240612 382.0144702 404.2643114 359.7646289 0.889924287 -0.168245496 0.880387815 1

chr1_37297806_37303997 765.6607262 1011.661433 519.6600196 0.513669893 -0.961086578 0.245471116 1

chr1_37898225_37901243 179.0634785 276.1805692 81.9463877 0.296713081 -1.752859562 0.282691792 1

chr1_37920236_37921730 165.0111537 182.119071 147.9032363 0.812123824 -0.300228384 0.85957951 1

chr1_37996542_38004419 1806.600125 1960.281649 1652.918601 0.84320465 -0.246045273 0.691467896 1

chr1_38018633_38020712 665.5939945 809.5292771 521.658712 0.644397586 -0.633977005 0.466378927 1

chr1_38070415_38072059 81.47745366 47.0307491 115.9241582 2.464858851 1.301505034 0.605708127 1

chr1_38077544_38080423 273.4931913 263.1720641 283.8143184 1.078436343 0.108941021 0.935283636 1

chr1_38099213_38100976 133.0274974 175.1144913 90.94050343 0.519320261 -0.945303581 0.616254737 1

chr1_38109553_38110829 58.48595103 37.02420674 79.94769532 2.159335806 1.11058762 0.721043456 1

chr1_38187040_38190335 241.0759181 357.2335623 124.9182739 0.349682357 -1.515883089 0.276683204 1

chr1_38217729_38219594 502.6364711 711.465162 293.8077803 0.412961584 -1.275920514 0.196150922 1

chr1_38258326_38261716 1520.310332 1995.304547 1045.316116 0.523888004 -0.932669668 0.154468026 1

chr1_38283075_38284172 71.50492046 79.05168466 63.95815626 0.809067593 -0.305667858 0.914225309 1

chr1_38324050_38329921 2010.722471 2351.537455 1669.907486 0.710134335 -0.493836133 0.41247451 1

chr1_38356004_38357573 115.5232425 151.0987897 79.94769532 0.529108774 -0.918363754 0.653043296 1

chr1_38379970_38382241 134.0006827 135.0883219 132.9130435 0.983897361 -0.023420271 0.994512931 1

chr1_38395423_38399125 775.6142928 950.6215244 600.6070611 0.63180461 -0.662449632 0.419293627 1

chr1_38469637_38472367 447.567133 550.3598299 344.7744361 0.626452763 -0.674722366 0.511749765 1

chr1_38477201_38480130 996.6771262 1267.828917 725.525335 0.572258075 -0.805262181 0.282680473 1

chr1_38511056_38514436 952.2297655 1303.85247 600.6070611 0.460640352 -1.118287297 0.144228299 1

chr1_38547735_38551107 207.5585794 297.1943082 117.9228506 0.396787043 -1.333563181 0.372747638 1

chr1_38588115_38590766 235.5441969 303.1982336 167.8901602 0.553730667 -0.85274367 0.540485724 1

chr1_38602657_38607728 687.5521418 767.5017992 607.6024844 0.791662619 -0.337042364 0.694874968 1

chr1_38638947_38645720 1050.854378 1593.041544 508.6672115 0.319305679 -1.646989884 0.028051633 1

chr1_38661722_38664828 190.5723102 301.1969251 79.94769532 0.265433305 -1.913578691 0.227985492 1

chr1_38672429_38674809 133.5265165 174.1138371 92.93919581 0.533784088 -0.905671794 0.63033316 1

chr1_38707546_38710538 323.0667794 425.2780504 220.8555083 0.519320261 -0.945303581 0.429383818 1

chr1_38774326_38775312 61.00916936 75.04906772 46.969271 0.625847495 -0.676116948 0.82428092 1

chr1_38904875_38909691 484.7201822 821.537128 147.9032363 0.180032321 -2.473672156 0.01755856 1

chr1_38928840_38931684 129.0379609 187.1223422 70.9535796 0.379182832 -1.39903445 0.468622074 1

chr1_39034990_39036097 47.51080153 64.04187112 30.97973194 0.48374183 -1.047690799 0.770034476 1

chr1_39106884_39108214 47.0248629 85.05561008 8.994115723 0.105743945 -3.241353044 0.394024531 1

chr1_39269295_39270546 69.03009979 115.0752372 22.9849624 0.199738562 -2.323815204 0.417036942 1

chr1_39283326_39284854 119.015068 142.0929015 95.93723438 0.675172604 -0.566671728 0.77850059 1

chr1_39323788_39327163 1032.22128 1370.896304 693.5462569 0.505907161 -0.983055435 0.185536824 1

chr1_39415194_39418129 290.5160857 315.2060844 265.8260869 0.843340595 -0.245812692 0.846124413 1

chr1_39423688_39425947 242.5173835 269.1759896 215.8587774 0.801924339 -0.31846197 0.817572287 1

chr1_39455736_39458877 887.5495685 963.6300295 811.4691075 0.842096118 -0.24794318 0.750650077 1

chr1_39490783_39493580 1204.101631 1359.889107 1048.314155 0.770882088 -0.375417888 0.592094885 1

chr1_39529791_39531425 93.9941339 85.05561008 102.9326577 1.2101807 0.275222482 0.908038525 1

chr1_39546601_39548172 196.0209706 228.1491659 163.8927754 0.718357986 -0.477225119 0.755547111 1

chr1_39568329_39574328 1648.976154 1613.054629 1684.897679 1.04453851 0.062865683 0.922054833 1

chr1_39605394_39611187 664.1106718 833.5449788 494.6763648 0.593460914 -0.75277508 0.388056667 1

chr1_39621314_39627406 863.5822645 989.6470397 737.5174893 0.745232856 -0.424236814 0.589815725 1

chr1_39657070_39658678 132.0189949 161.105332 102.9326577 0.638915276 -0.64630346 0.733144943 1

chr1_39669760_39671274 71.50949862 86.05626432 56.96273292 0.661924305 -0.59526185 0.828704999 1

chr1_39678919_39681501 163.5271769 205.1341184 121.9202354 0.594344014 -0.750629871 0.655665844 1

chr1_39734961_39736246 69.9921667 58.0379457 81.9463877 1.411945008 0.4976839 0.859253193 1

chr1_39849926_39851650 79.96142979 21.01373896 138.9091206 6.610395269 2.72473654 0.304451261 1

chr1_39859547_39862098 134.4735409 94.06149821 174.8855835 1.85926853 0.894735151 0.633117522 1

chr1_39873030_39877533 1180.090507 1318.862283 1041.318732 0.789558352 -0.340882202 0.628917285 1

chr1_39955958_39958025 335.9673707 286.1871116 385.7476299 1.347886101 0.430698591 0.71338322 1

chr1_39963912_39965381 91.02356427 127.083088 54.96404053 0.43250476 -1.209212083 0.607592036 1

chr1_40001914_40004145 132.4774646 98.06411515 166.890814 1.701854075 0.767107339 0.684843555 1

chr1_40024345_40026089 207.9686514 160.1046778 255.832625 1.597908497 0.676184796 0.64846516 1

chr1_40071697_40073058 63.97973899 33.0215898 94.93788819 2.875024757 1.523574379 0.604837703 1

chr1_40127270_40128963 89.01113742 106.069349 71.95292579 0.678357381 -0.559882563 0.815400128 1

chr1_40136997_40138326 140.032077 189.1236507 90.94050343 0.480852094 -1.056334894 0.564846165 1

chr1_40142129_40143628 79.52192675 113.0739287 45.96992481 0.406547516 -1.298504116 0.611865221 1

chr1_40203502_40205900 801.6502696 1031.674518 571.6260215 0.554075934 -0.85184439 0.294055827 1

chr1_40223601_40226042 108.0418805 172.1125286 43.97123243 0.255479556 -1.968720246 0.363203449 1

chr1_40239528_40245560 409.1675172 665.4350671 152.8999673 0.229774436 -2.121709797 0.05526742 1

chr1_40264558_40265888 131.0104924 147.0961727 114.924812 0.781290294 -0.356069403 0.853274484 1

chr1_40281147_40286908 645.5815638 770.5037619 520.6593658 0.675738902 -0.565462183 0.520848353 1

chr1_40309652_40312764 346.994188 338.2211319 355.7672442 1.051877635 0.072966885 0.951933657 1

chr1_40322425_40327342 455.5206991 487.3186131 423.7227852 0.869498463 -0.201744617 0.844334534 1

chr1_40347579_40352892 1185.142176 1402.917239 967.3671134 0.689539687 -0.536294506 0.446642285 1

chr1_40356198_40362200 580.6436821 800.523389 360.7639751 0.450660131 -1.149888272 0.21466906 1

chr1_40363482_40369283 1987.292773 2435.592411 1538.993135 0.631876306 -0.662285927 0.273951036 1

chr1_40423768_40428121 850.4292205 742.4854433 958.3729976 1.290763349 0.368224519 0.641790082 1

chr1_40504341_40507872 1845.358145 2393.564933 1297.151357 0.541932804 -0.883814116 0.153601344 1

chr1_40570238_40576144 1806.74728 2185.428852 1428.065708 0.65344873 -0.613854049 0.322700346 1

chr1_40625397_40629142 1148.683699 1429.934904 867.4324942 0.606623765 -0.721126078 0.312012994 1

chr1_40708825_40712531 276.4807655 247.1615964 305.7999346 1.237246964 0.307133502 0.812267121 1

chr1_40721236_40725601 1658.753788 2047.338567 1270.169009 0.620400079 -0.688729224 0.279080116 1

chr1_40731714_40733019 101.0327227 151.0987897 50.96665577 0.337306843 -1.567866507 0.481500552 1

chr1_40733435_40735568 179.0562842 265.1733726 92.93919581 0.350484647 -1.512576848 0.350876899 1

chr1_40767960_40774724 3531.389244 4892.198561 2170.579928 0.443681895 -1.172402412 0.028902723 1

chr1_40781698_40783167 322.6004614 476.3114165 168.8895064 0.35457791 -1.495825437 0.216497203 1

chr1_40803380_40806808 334.5592606 425.2780504 243.8404707 0.573367166 -0.802468803 0.494576285 1

chr1_40838988_40841722 1076.709844 1397.913968 755.5057208 0.54045223 -0.887760991 0.224382288 1

chr1_40852978_40859712 646.5900663 784.5129212 508.6672115 0.648386021 -0.625075108 0.477748104 1

chr1_40868618_40873022 310.5612175 404.2643114 216.8581236 0.536426584 -0.89854736 0.460684954 1

chr1_40887037_40888945 102.0216046 135.0883219 68.95488721 0.510442992 -0.97017825 0.658704525 1

chr1_40914625_40917129 931.1473542 1156.756297 705.5384112 0.609928308 -0.713288419 0.352375731 1

chr1_40973454_40975482 831.1349063 1037.678443 624.5913697 0.601912253 -0.732374909 0.359770961 1

chr1_40996134_40998332 410.506955 421.2754335 399.7384766 0.94887678 -0.075707343 0.945605147 1

chr1_41126319_41132466 1429.826336 1164.761531 1694.891141 1.45514004 0.541158002 0.415018715 1

chr1_41247692_41251203 784.1113515 954.6241414 613.5985616 0.642764555 -0.63763772 0.434924722 1

chr1_41314470_41316595 113.5088536 127.083088 99.93461915 0.786372292 -0.346715607 0.868688773 1

chr1_41342313_41344765 305.9771747 271.177298 340.7770513 1.256657743 0.329591778 0.788597386 1

chr1_41408055_41412881 812.9792452 781.5109585 844.4475318 1.080531914 0.111741683 0.890683349 1

chr1_41432724_41433798 63.50295668 68.04448806 58.9614253 0.86651288 -0.206706901 0.948560246 1

chr1_41444329_41448385 1211.519553 1241.811907 1181.227198 0.951212653 -0.072160189 0.918789361 1

chr1_41485582_41488126 141.4689642 94.06149821 188.8764302 2.008010013 1.005766463 0.581311566 1

chr1_41509261_41514761 1461.395342 1301.851161 1620.939523 1.245103565 0.316265748 0.631335414 1

chr1_41567131_41569211 112.9633989 57.03729147 168.8895064 2.961036578 1.566102313 0.45330317 1

chr1_41572419_41577748 527.3186216 250.1635591 804.4736842 3.21579085 1.685173579 0.084776114 1

chr1_41584267_41585980 70.97777842 37.02420674 104.9313501 2.834128246 1.502905042 0.585425634 1

chr1_41703322_41709354 1904.066463 2006.311744 1801.821183 0.898076378 -0.155089949 0.799953182 1

chr1_41733744_41735079 72.49445632 64.04187112 80.94704151 1.263970588 0.337962893 0.903702851 1

chr1_41806566_41809271 573.4350474 474.310108 672.5599869 1.417975235 0.503832336 0.58569373 1

chr1_41850483_41858366 1193.821707 921.6025516 1466.040863 1.59075174 0.6697087 0.341339703 1

chr1_42039156_42041023 193.0150838 216.141315 169.8888526 0.78600823 -0.347383676 0.822987055 1

chr1_42083238_42085361 166.9908794 153.1000981 180.8816607 1.181460122 0.240570935 0.887316831 1

chr1_42126764_42133506 1012.122519 1199.784429 824.460608 0.687173952 -0.541252744 0.466647994 1

chr1_42149102_42153277 454.5965655 602.3938502 306.7992808 0.509300154 -0.973411941 0.342211128 1

chr1_42160068_42162359 162.9791061 131.0857049 194.8725073 1.486603802 0.572020203 0.734813412 1

chr1_42276808_42280250 242.5232697 278.1818777 206.8646616 0.743630978 -0.427341225 0.755974804 1

chr1_42305540_42309198 291.5265503 332.2172064 250.8358941 0.75503583 -0.405382987 0.746821885 1

chr1_42343852_42351519 1971.160003 2216.449133 1725.870873 0.778664778 -0.360925725 0.551029499 1

chr1_42352763_42359429 899.7595123 1296.84789 502.6711343 0.387609941 -1.367322523 0.081928458 1

chr1_42390891_42394971 487.082511 613.4010468 360.7639751 0.588137202 -0.765775346 0.440103881 1

chr1_42409677_42414718 821.0080239 833.5449788 808.4710689 0.969918948 -0.044063902 0.957671423 1

chr1_42418452_42422482 252.0595699 343.224403 160.8947368 0.468774176 -1.093034998 0.418525594 1

chr1_42446007_42448175 234.4428232 147.0961727 321.7894737 2.187612823 1.129357424 0.420330809 1

chr1_42467688_42470387 174.9725685 133.0870134 216.8581236 1.629446164 0.704381687 0.664407407 1

chr1_42500236_42502540 495.998798 494.3231927 497.6744034 1.006779392 0.009747591 0.994401073 1

chr1_42546068_42548576 178.9470625 98.06411515 259.8300098 2.649593171 1.405770859 0.384920499 1

chr1_42610502_42614017 244.4833747 219.1432777 269.8234717 1.231265109 0.300141428 0.827374918 1

chr1_42674762_42676890 137.4702714 92.06018973 182.880353 1.986530264 0.990250773 0.592995454 1

chr1_42731881_42733439 98.49184579 86.05626432 110.9274273 1.289010488 0.366264002 0.87249971 1

chr1_42845426_42847447 513.4611955 454.2970233 572.6253677 1.260464715 0.333955732 0.730559292 1

chr1_42896213_42897343 59.00328273 64.04187112 53.96469434 0.842647059 -0.246999607 0.940642787 1

chr1_42920457_42923821 1859.067762 1963.283612 1754.851912 0.893835156 -0.161919306 0.792632523 1

chr1_42928534_42930134 147.9902213 133.0870134 162.8934292 1.223961865 0.291558609 0.871750499 1

chr1_43145830_43150537 1744.880687 2327.521754 1162.239621 0.499346405 -1.00188711 0.111940965 1

chr1_43157201_43159453 110.0039477 116.0758914 103.9320039 0.895379761 -0.159428387 0.943214463 1

chr1_43200095_43202730 195.5271837 237.155054 153.8993135 0.648939632 -0.623843818 0.683808931 1

chr1_43207791_43209917 126.5330552 177.1157998 75.95031055 0.428817252 -1.221565146 0.530103323 1

chr1_43222022_43223103 83.00459591 90.05888126 75.95031055 0.843340595 -0.245812692 0.924813053 1

chr1_43231186_43234245 1024.192502 1318.862283 729.5227198 0.553145487 -0.854269111 0.250238391 1

chr1_43281431_43283498 627.0727306 738.4828264 515.6626348 0.698273022 -0.51813686 0.560889359 1

chr1_43299395_43302786 286.0190278 315.2060844 256.8319712 0.814806515 -0.295470579 0.816369176 1

chr1_43311323_43313486 834.603841 993.6496566 675.5580255 0.679875468 -0.556657581 0.485039843 1

chr1_43388740_43391617 830.6496217 1059.692836 601.6064073 0.567717726 -0.816754307 0.307720648 1

chr1_43469555_43474985 548.0563631 634.4147858 461.6979405 0.727754067 -0.458477098 0.626481602 1

chr1_43516907_43520152 281.5265481 322.2106641 240.8424322 0.747468843 -0.419914651 0.742268755 1

chr1_43523811_43525094 88.51146432 106.069349 70.9535796 0.66893575 -0.580060445 0.809375899 1

chr1_43586473_43588099 126.5435195 193.1262676 59.96077149 0.310474449 -1.687453551 0.390359549 1

chr1_43636853_43638616 570.6260213 763.4991823 377.7528604 0.494765246 -1.015183932 0.275538125 1

chr1_43670771_43673890 252.0994653 404.2643114 99.93461915 0.247201191 -2.016242403 0.145001667 1

chr1_43692471_43694608 143.5154002 167.1092575 119.921543 0.717623576 -0.478700806 0.792040046 1

chr1_43699505_43703151 279.9686668 232.1517828 327.7855508 1.411945008 0.4976839 0.697157325 1

chr1_43735080_43737751 375.579544 497.3251554 253.8339326 0.510398338 -0.970304464 0.384606987 1

chr1_43749846_43752554 500.9987991 499.3264639 502.6711343 1.006698364 0.009631475 0.994448775 1

chr1_43758509_43760086 73.01113399 90.05888126 55.96338672 0.62140886 -0.686385284 0.799710702 1

chr1_43768866_43770308 98.99805911 96.06280668 101.9333115 1.061111111 0.085575732 0.974030246 1

chr1_43813597_43816188 1182.621574 1368.894995 996.3481529 0.727848488 -0.45828993 0.515605995 1

chr1_43822990_43827056 980.1741795 1246.815178 713.5331807 0.572284644 -0.805195199 0.285547101 1

chr1_43830302_43835315 2401.809539 2875.880275 1927.738803 0.670312607 -0.577094028 0.31712063 1

chr1_43853875_43857276 1136.629412 1334.872751 938.3860738 0.702977923 -0.508448713 0.476668806 1

chr1_43918180_43921469 535.3480543 303.1982336 767.4978751 2.531340193 1.339901408 0.163914767 1

chr1_43984460_43989816 501.6004997 655.4285247 347.7724746 0.530603203 -0.91429471 0.351484883 1

chr1_44046142_44049759 276.0628452 372.2433759 179.8823145 0.483238457 -1.049192824 0.416553913 1

chr1_44113167_44118166 1709.237122 2072.354923 1346.11932 0.64956022 -0.622464812 0.323477392 1

chr1_44171395_44174416 965.1957591 1264.826955 665.5645635 0.526209978 -0.926289491 0.222596908 1

chr1_44229076_44232135 197.0294731 242.1583252 151.9006211 0.627278129 -0.672822833 0.659138001 1

chr1_44247795_44251846 455.0883903 590.3859994 319.7907813 0.541663897 -0.884530159 0.387254971 1

chr1_44286301_44289721 338.5703798 446.2917894 230.8489702 0.517260178 -0.951037968 0.416438507 1

chr1_44321662_44328619 1277.134348 1482.969578 1071.299117 0.722401277 -0.469127651 0.495020519 1

chr1_44377773_44379677 100.514083 122.0798168 78.94834913 0.646694525 -0.628843699 0.777178953 1

chr1_44394952_44398172 400.0805303 523.3421656 276.818895 0.528944376 -0.91881208 0.396532618 1

chr1_44410060_44414799 1784.685143 2068.352306 1501.01798 0.725707112 -0.462540685 0.457159857 1

chr1_44434779_44437175 457.6613144 704.4605823 210.8620464 0.299324124 -1.740219539 0.094429428 1

chr1_44439051_44442236 1457.835498 1971.288845 944.382151 0.479068379 -1.061696505 0.11029677 1

chr1_44455746_44459237 1129.162439 1377.900883 880.4239947 0.638960324 -0.646201745 0.367309251 1

chr1_44470274_44472149 124.0072208 135.0883219 112.9261196 0.835942871 -0.258523744 0.897778981 1

chr1_44508978_44510423 103.0065623 113.0739287 92.93919581 0.821933021 -0.282907261 0.899718779 1

chr1_44616310_44624160 913.3245904 645.4219824 1181.227198 1.830162639 0.87197186 0.259896919 1

chr1_44731340_44736875 581.8124201 295.1929997 868.4318404 2.941912042 1.556754113 0.09594467 1

chr1_44740116_44741904 120.9954477 114.0745829 127.9163125 1.121339296 0.165222876 0.937251639 1

chr1_44747532_44750778 336.5278679 379.2479555 293.8077803 0.774711573 -0.368268803 0.75355988 1

chr1_44810696_44812029 64.49118449 51.03336605 77.94900294 1.527412534 0.611089767 0.835485735 1

chr1_44819661_44822173 687.5933452 830.5430161 544.6436744 0.655768171 -0.608742216 0.478577441 1

chr1_44870366_44873066 326.5298278 372.2433759 280.8162798 0.754388924 -0.4066196 0.732354592 1

chr1_44937995_44939240 65.52061571 97.06346092 33.97777051 0.350057274 -1.51433711 0.6014452 1

chr1_45032862_45034192 71.02028988 102.0667321 39.97384766 0.391644239 -1.352384357 0.622608357 1

chr1_45067307_45071075 372.8960902 214.1400066 531.6521739 2.48273166 1.31192834 0.244358134 1

chr1_45082294_45083803 109.5095067 124.0811253 94.93788819 0.765127556 -0.386227812 0.856452406 1

chr1_45092862_45099168 2099.639429 2313.512594 1885.766263 0.815109573 -0.294934084 0.620405941 1

chr1_45107602_45111672 422.4618301 364.238142 480.6855181 1.319701214 0.400211334 0.704568818 1

chr1_45146990_45148869 92.97124291 49.03205758 136.9104282 2.792263572 1.481435129 0.525806458 1

chr1_45158160_45163097 326.913739 195.1275761 458.6999019 2.350769231 1.23313292 0.30231897 1

chr1_45165263_45169416 391.4323924 288.18842 494.6763648 1.716503268 0.779472604 0.475625961 1

chr1_45177627_45181447 290.0412654 353.2309454 226.8515855 0.642218889 -0.638862996 0.611077745 1

chr1_45187175_45188744 93.54024237 155.1014066 31.97907813 0.206181742 -2.278011515 0.337984736 1

chr1_45195289_45199607 871.6614029 1118.731436 624.5913697 0.558303226 -0.840879203 0.285226064 1

chr1_45203719_45206684 759.1930989 1054.689565 463.6966329 0.439652243 -1.185565266 0.155045835 1

chr1_45239801_45244669 2155.390912 2753.800458 1556.981366 0.565393677 -0.822672346 0.166176547 1

chr1_45294310_45299101 332.4539625 262.1714099 402.7365152 1.536157262 0.619325917 0.598798392 1

chr1_45412351_45413960 68.51473015 91.0595355 45.96992481 0.504833728 -0.986119794 0.725229036 1

chr1_45450877_45453598 772.7137035 1099.719006 445.7084014 0.405292988 -1.302962881 0.116618388 1

chr1_45466118_45467345 65.00982424 80.0523389 49.96730957 0.624183007 -0.679959015 0.81576948 1

chr1_45475286_45479821 1517.335838 2031.3281 1003.343576 0.493934769 -1.017607568 0.121063456 1

chr1_45637875_45639344 106.5095061 121.0791626 91.93984962 0.759336682 -0.397188391 0.854721665 1

chr1_45670953_45673185 874.1689247 1132.740595 615.597254 0.543458278 -0.879758812 0.263310857 1

chr1_45711389_45713950 145.0758976 261.1707557 28.98103955 0.110965868 -3.171812111 0.099258781 1

chr1_45731291_45732930 120.5579068 209.1367354 31.97907813 0.152909904 -2.709246242 0.1945681 1

chr1_45762769_45766251 204.0726401 315.2060844 92.93919581 0.294852163 -1.761936317 0.248098763 1

chr1_45767958_45771793 1325.262546 1727.129212 923.3958809 0.534642038 -0.903354817 0.185802073 1

chr1_45804101_45807486 1290.28543 1727.129212 853.4416475 0.494138853 -1.017011599 0.140462331 1

chr1_45813083_45816710 449.5573231 537.3513249 361.7633213 0.67323426 -0.5708195 0.577933828 1

chr1_45929954_45931398 85.50230736 89.05822703 81.9463877 0.920143938 -0.120068536 0.965366835 1

chr1_45964338_45968841 875.6797164 1150.752372 600.6070611 0.521925547 -0.938084075 0.233100848 1

chr1_46013657_46021217 2038.392195 2638.725221 1438.05917 0.544982539 -0.875718089 0.14654721 1

chr1_46031440_46036610 803.0322188 852.5574093 753.5070284 0.883819694 -0.178176017 0.826348012 1

chr1_46039226_46043105 498.0014146 500.3271181 495.675711 0.990703268 -0.013475084 0.991325102 1

chr1_46048282_46053337 1569.868223 2133.394832 1006.341615 0.471709034 -1.084030864 0.095709091 1

chr1_46138909_46140086 45.48398618 21.01373896 69.9542334 3.328976035 1.735078485 0.64011112 1

chr1_46195348_46198025 263.0589183 353.2309454 172.8868911 0.489444352 -1.030783255 0.435485826 1

chr1_46213825_46218890 1459.813262 1939.26791 980.3586139 0.505530262 -0.984130639 0.138080309 1

chr1_46319504_46322478 139.5409062 202.1321557 76.94965675 0.380689834 -1.393312052 0.4509793 1

chr1_46336168_46340167 403.6560706 642.4200197 164.8921216 0.256673386 -1.961994382 0.076475959 1

chr1_46347107_46349036 110.0392649 170.1112202 49.96730957 0.29373318 -1.767421856 0.406759359 1

chr1_46361657_46363823 110.0255304 149.0974812 70.9535796 0.475887178 -1.071308511 0.61046682 1

chr1_46386459_46389279 227.0065888 237.155054 216.8581236 0.914414936 -0.129079126 0.930070928 1

chr1_46418887_46421045 132.0373075 189.1236507 74.95096436 0.396306671 -1.335310843 0.48300865 1

chr1_46424725_46427002 94.52847018 138.0902846 50.96665577 0.369082126 -1.437986224 0.533657594 1

chr1_46462007_46464554 272.5383186 331.2165522 213.860085 0.645680548 -0.63110753 0.625986026 1

chr1_46483256_46490494 573.6417185 790.5168466 356.7665904 0.451308017 -1.147815689 0.217774945 1

chr1_46530794_46531747 37.51341548 58.0379457 16.98888526 0.292720307 -1.772405263 0.677084375 1

chr1_46661946_46665526 952.9145269 822.5377822 1083.291272 1.317010957 0.397267348 0.600819769 1

chr1_46688128_46690004 109.4663413 58.0379457 160.8947368 2.772233491 1.471048773 0.488157701 1

chr1_46712035_46713173 140.4944709 132.0863592 148.9025825 1.127312339 0.172887292 0.92777733 1

chr1_46766124_46772797 2631.753342 2255.474649 3008.032036 1.333658101 0.415388862 0.461488639 1

chr1_46804591_46808056 1020.391978 855.559372 1185.224583 1.385321255 0.470220575 0.525807589 1

chr1_46812268_46814355 104.956857 39.02551521 170.8881987 4.378883861 2.130563186 0.335584959 1

chr1_46853754_46855126 71.99347518 62.04056265 81.9463877 1.320851782 0.401468585 0.885188369 1

chr1_46859359_46864618 1060.316774 780.5103043 1340.123243 1.716983409 0.779876099 0.287606222 1

chr1_46866833_46868256 205.9601487 145.0948643 266.8254331 1.838972279 0.878899732 0.555738513 1

chr1_46954831_46956398 107.4879236 89.05822703 125.9176201 1.413879709 0.499659383 0.815728606 1

chr1_46980809_46984670 477.408866 338.2211319 616.5966002 1.823057586 0.866360133 0.387109527 1

chr1_46997531_46999949 349.450042 273.1786065 425.7214776 1.55839977 0.64006537 0.577834795 1

chr1_47012941_47014313 61.48071949 32.02093556 90.94050343 2.84003268 1.505907531 0.618066149 1

chr1_47081223_47083289 636.8627915 427.2793589 846.4462242 1.981013608 0.98623879 0.267700824 1

chr1_47107538_47110376 220.4552466 152.0994439 288.8110493 1.898830409 0.92511106 0.521009169 1

chr1_47132504_47135052 764.4239699 648.4239451 880.4239947 1.357790688 0.441261096 0.592265983 1

chr1_47140674_47142103 56.96927314 10.00654236 103.9320039 10.38640523 3.376624514 0.316261575 1

chr1_47189497_47191428 290.4140582 159.1040236 421.7240928 2.650618654 1.406329124 0.26787844 1

chr1_47255400_47256806 68.49053133 54.03532876 82.94573389 1.535027838 0.61826482 0.826877692 1

chr1_47262920_47266586 302.4689986 255.1668302 349.771167 1.370754838 0.454970565 0.711997408 1

chr1_47287946_47289140 57.49314506 47.0307491 67.95554102 1.444917258 0.53098688 0.868117947 1

chr1_47340860_47342185 97.50034786 98.06411515 96.93658058 0.988502067 -0.016684111 0.999315555 1

chr1_47448247_47451482 230.9372633 135.0883219 326.7862046 2.419055919 1.274444119 0.368055621 1

chr1_47456858_47457856 59.50033974 60.03925417 58.9614253 0.98204793 -0.026134656 0.99949291 1

chr1_47477669_47479268 116.4885795 99.06476939 133.9123897 1.351766026 0.434845461 0.832097624 1

chr1_47488410_47490747 417.930109 311.2034675 524.6567505 1.685896223 0.753515733 0.47746745 1

chr1_47532638_47535976 246.4814131 218.1426235 274.8202027 1.259818912 0.333216374 0.807592491 1

chr1_47557173_47558287 64.49576265 58.0379457 70.9535796 1.222537751 0.289879015 0.924577956 1

chr1_47778279_47781540 455.4566049 389.2544979 521.658712 1.340148193 0.422392542 0.678944341 1

chr1_47797746_47801812 1423.850533 1195.781812 1651.919255 1.381455411 0.466188997 0.482929639 1

chr1_47972432_47976253 609.3376058 361.2361793 857.4390323 2.373624464 1.247091701 0.170787048 1

chr1_47999231_48004320 1137.275587 794.5194636 1480.03171 1.862801073 0.897473619 0.21095182 1

chr1_48034409_48036643 205.9575326 141.0922473 270.8228179 1.919473416 0.940710579 0.528529695 1

chr1_48042874_48045719 205.9948119 198.1295388 213.860085 1.07939526 0.110223257 0.943951395 1

chr1_48056821_48059400 111.4650336 58.0379457 164.8921216 2.841108857 1.50645411 0.473491458 1

chr1_48115660_48118533 138.9516321 65.04252536 212.8607388 3.272639517 1.710454698 0.359497404 1

chr1_48162002_48165622 158.4362667 61.03990841 255.832625 4.191235401 2.067375553 0.239462119 1

chr1_48174208_48176774 457.4055916 313.2047759 601.6064073 1.920808536 0.94171372 0.356634944 1

chr1_48190186_48191530 98.01702554 124.0811253 71.95292579 0.579886148 -0.786158418 0.726804849 1

chr1_48235223_48239360 291.4049021 146.0955185 436.7142857 2.98923807 1.579777801 0.214543577 1

chr1_48258116_48259538 56.48333451 31.02028132 81.9463877 2.641703563 1.401468585 0.660285568 1

chr1_48273448_48279531 1404.868188 1203.787046 1605.94933 1.33408092 0.415846177 0.533040542 1

chr1_48295617_48298326 139.9261254 27.01766438 252.8345864 9.35812152 3.226218963 0.10095225 1

chr1_48303582_48306650 124.9548992 56.03663723 193.8731612 3.459757236 1.790670811 0.366737018 1

chr1_48347034_48348714 117.5088544 131.0857049 103.9320039 0.792855361 -0.334870393 0.87065807 1

chr1_48358753_48363340 549.6037801 708.4631993 390.7443609 0.551537979 -0.858467862 0.362828087 1

chr1_48368469_48373776 362.4304241 256.1674845 468.6933638 1.829636438 0.871557003 0.441692558 1

chr1_48383500_48384896 87.50492388 95.06215244 79.94769532 0.841004472 -0.249814623 0.920769304 1

chr1_48405114_48410290 617.7829967 286.1871116 949.3788819 3.317336259 1.730025257 0.059242717 1

chr1_48468469_48471992 213.4454348 130.0850507 296.8058189 2.281628959 1.190064198 0.418463931 1

chr1_48508265_48510021 80.46829713 32.02093556 128.9156587 4.025980392 2.009340146 0.436062326 1

chr1_48559269_48560245 60.99216478 49.03205758 72.95227198 1.487848473 0.573227605 0.851475183 1

chr1_48680156_48682001 75.97843352 43.02813216 108.9287349 2.531570147 1.340032461 0.610898796 1

chr1_48712028_48713843 113.4506456 38.02486098 188.8764302 4.967182663 2.312427801 0.27748527 1

chr1_48907731_48910187 152.979104 121.0791626 184.8790454 1.526927024 0.610631114 0.726712668 1

chr1_48935677_48938970 417.9039481 271.177298 564.6305982 2.082145527 1.058070906 0.320521821 1

chr1_49241164_49243941 186.9490262 109.0713118 264.8267407 2.428014631 1.279777115 0.417146518 1

chr1_49811764_49813945 132.0288052 176.1151456 87.94246485 0.499346405 -1.00188711 0.597000618 1

chr1_50266442_50270426 501.2205127 838.54825 163.8927754 0.195448235 -2.355141539 0.02121146 1

chr1_50390744_50391660 36.50949113 51.03336605 21.98561621 0.430808663 -1.214880833 0.778821593 1

chr1_50488662_50490499 262.9784735 230.1504743 295.8064727 1.285274226 0.362076205 0.784300286 1

chr1_50561621_50563594 444.8058505 148.096827 741.5148741 5.006959901 2.323934901 0.030609743 1

chr1_50587396_50591779 390.8895538 222.1452404 559.6338672 2.519225107 1.332980041 0.227125982 1

chr1_50645352_50648727 235.4245108 120.0785083 350.7705132 2.921176471 1.546549515 0.273103106 1

chr1_50709531_50712725 198.9745357 160.1046778 237.8443936 1.485555556 0.571002559 0.706903052 1

chr1_50730545_50733243 102.9444301 18.01177625 187.877084 10.43079158 3.382776741 0.15014542 1

chr1_50736993_50739143 102.9437761 17.01112202 188.8764302 11.10311419 3.472892473 0.141223914 1

chr1_50749910_50756791 535.280036 199.130193 871.429879 4.376181561 2.129672595 0.030753715 1

chr1_50797533_50799875 443.8745227 252.1648675 635.5841778 2.520510426 1.333715922 0.200993008 1

chr1_50940648_50942552 76.96273719 20.01308472 133.9123897 6.69124183 2.742273986 0.31290791 1

chr1_50958255_50959492 68.98431822 45.02944063 92.93919581 2.063965142 1.045418605 0.70819352 1

chr1_51109300_51110725 60.99020271 46.03009487 75.95031055 1.650014209 0.722478448 0.812181411 1

chr1_51338767_51340268 86.94965888 10.00654236 163.8927754 16.37856209 4.0337368 0.131686699 1

chr1_51424430_51428017 686.4370337 590.3859994 782.4880679 1.325383848 0.406410243 0.63626502 1

chr1_51432955_51436707 791.8347017 539.3526333 1044.31677 1.936241163 0.953258655 0.243250793 1

chr1_51442838_51444495 271.4290966 163.1066405 379.7515528 2.328240908 1.219240345 0.350534989 1

chr1_51636201_51637520 52.98104469 24.01570167 81.9463877 3.412200436 1.770702394 0.596823529 1

chr1_51692817_51696195 921.2035979 468.3061826 1374.101013 2.93419362 1.552964074 0.047381134 1

chr1_51699891_51704922 1734.193961 1266.828263 2201.55966 1.737851707 0.797304981 0.205129591 1

chr1_51759559_51765857 1000.492693 989.6470397 1011.338346 1.021918225 0.031279754 0.967938058 1

chr1_51818467_51820289 204.9640726 150.0981354 259.8300098 1.731067538 0.791662013 0.596339065 1

chr1_51927747_51929459 56.97188923 14.00915931 99.93461915 7.133520075 2.834614158 0.389678159 1

chr1_51974911_51976859 88.97255009 47.0307491 130.9143511 2.783590599 1.47694704 0.537786787 1

chr1_51980874_51985445 1272.117996 1452.949951 1091.286041 0.751083023 -0.412955707 0.548513573 1

chr1_51999557_52001496 122.4990452 121.0791626 123.9189277 1.023453789 0.033445964 0.991056911 1

chr1_52057891_52059634 87.50623193 97.06346092 77.94900294 0.803072569 -0.316397733 0.898313363 1

chr1_52070978_52075660 284.9163461 157.1027151 412.7299771 2.627134591 1.393490113 0.276524731 1

chr1_52081745_52084422 496.9275098 386.2525352 607.6024844 1.573070541 0.653583367 0.505904276 1

chr1_52093941_52096072 148.4735439 108.0706575 188.8764302 1.747712418 0.805467813 0.649822095 1

chr1_52113394_52116156 102.9718991 60.03925417 145.904544 2.430152505 1.281046854 0.558782415 1

chr1_52194104_52197535 655.9085772 516.3375859 795.4795684 1.540619142 0.623510256 0.476322074 1

chr1_52233541_52235058 58.47679472 23.01504743 93.938542 4.081614095 2.029139786 0.52021564 1

chr1_52253944_52256589 105.4728806 64.04187112 146.9038901 2.293872549 1.197785235 0.578900427 1

chr1_52306653_52307688 51.48921965 35.02289827 67.95554102 1.94031746 0.956292715 0.778074459 1

chr1_52342332_52346256 1216.396598 1058.692182 1374.101013 1.297923076 0.376204882 0.590081268 1

chr1_52455646_52456891 111.972555 70.04579654 153.8993135 2.197124183 1.135616414 0.585669722 1

chr1_52497770_52500128 638.9608953 579.3788028 698.5429879 1.205675776 0.269841998 0.76071353 1

chr1_52519124_52523975 2084.836286 1835.199869 2334.472703 1.272053656 0.347159526 0.56061677 1

chr1_52829886_52833032 869.9171252 743.4860975 996.3481529 1.340103273 0.422344184 0.590475783 1

chr1_52833756_52834976 259.9653924 207.1354269 312.7953579 1.510100723 0.59464478 0.653690515 1

chr1_53101970_53104175 86.45260188 14.00915931 158.8960444 11.34229692 3.503640924 0.180671186 1

chr1_53128387_53129645 53.48464192 30.01962709 76.94965675 2.563311547 1.358008836 0.681196628 1

chr1_53139922_53141538 64.98170128 37.02420674 92.93919581 2.510227875 1.327818336 0.647857502 1

chr1_53162572_53165077 643.9818251 616.4030095 671.5606407 1.089483066 0.123643773 0.889696091 1

chr1_53191654_53193553 432.455292 364.238142 500.6724419 1.374574445 0.458985043 0.660178055 1

chr1_53307877_53309593 163.0517026 242.1583252 83.94508009 0.346653703 -1.528432924 0.369800611 1

chr1_53332087_53334891 223.0719902 333.2178607 112.9261196 0.338895759 -1.561086514 0.281735554 1

chr1_53386654_53387875 118.0059114 127.083088 108.9287349 0.857145798 -0.222387472 0.915263167 1

chr1_53392171_53394074 165.4735475 125.0817795 205.8653154 1.645845752 0.718829133 0.667401594 1

chr1_53479418_53481977 344.423226 227.1485116 461.6979405 2.032581843 1.023313445 0.378636009 1

chr1_53661707_53664033 699.4435767 613.4010468 785.4861065 1.280542494 0.356755129 0.675870109 1

chr1_53684849_53687687 820.954394 751.4913314 890.4174566 1.184867236 0.244725414 0.760655658 1

chr1_53702752_53705565 664.935394 566.3702977 763.5004903 1.348058847 0.430883476 0.620505364 1

chr1_53759793_53762714 154.9575217 90.05888126 219.8561621 2.441249092 1.287619508 0.459917376 1

chr1_53778587_53784023 541.329743 281.1838404 801.4756456 2.850361687 1.511144997 0.116316811 1

chr1_53790675_53794529 1246.978684 1214.794243 1279.163125 1.052987477 0.074488279 0.915314415 1

chr1_53832961_53836619 169.4244967 54.03532876 284.8136646 5.270878722 2.398043497 0.162274877 1

chr1_53976691_53979887 647.2440887 256.1674845 1038.320693 4.053288399 2.019092829 0.026272744 1

chr1_54037011_54043638 1640.571966 986.6450769 2294.498856 2.325556484 1.217575981 0.058772433 1

chr1_54057406_54060395 525.8267966 261.1707557 790.4828375 3.026689705 1.597740778 0.101759782 1

chr1_54149760_54155533 420.8732097 227.1485116 614.5979078 2.705709597 1.436007003 0.179747873 1

chr1_54204099_54205688 99.48138164 71.04645077 127.9163125 1.800460278 0.848365771 0.703561769 1

chr1_54276624_54277862 52.48529572 30.01962709 74.95096436 2.496732026 1.320040985 0.693272271 1

chr1_54302518_54304629 754.4723654 712.4658162 796.4789146 1.117918778 0.160815373 0.846910565 1

chr1_54353448_54357878 731.894859 571.3735689 892.416149 1.561878598 0.64328232 0.443012963 1

chr1_54382850_54386887 484.8372522 236.1543998 733.5201046 3.10610391 1.635106094 0.106068184 1

chr1_54421358_54423608 112.4820384 85.05561008 139.9084668 1.644905805 0.718004971 0.729462017 1

chr1_54467023_54468870 103.9751694 66.04317959 141.9071592 2.148702713 1.103465891 0.61175638 1

chr1_54478977_54485071 1213.312228 926.6058228 1500.018633 1.618831435 0.694952769 0.321021672 1

chr1_54517061_54520647 922.404383 776.5076873 1068.301079 1.375776565 0.460246186 0.549103308 1

chr1_54536092_54537538 68.47025663 23.01504743 113.9254658 4.950042626 2.307440949 0.422459831 1

chr1_54559293_54561527 127.9542458 58.0379457 197.8705459 3.409330629 1.769488515 0.365824312 1

chr1_54601363_54604506 160.4382292 66.04317959 254.8332788 3.858585859 1.948072208 0.26264385 1

chr1_54617989_54621567 266.383968 89.05822703 443.709709 4.982242785 2.316795326 0.088747689 1

chr1_54664528_54667130 587.4723297 545.3565588 629.5881006 1.15445224 0.207208489 0.821718706 1

chr1_54694006_54695291 78.00459484 85.05561008 70.9535796 0.83420223 -0.261530926 0.922950517 1

chr1_54762528_54766469 321.3722074 126.0824338 516.661981 4.097810976 2.034853437 0.098684829 1

chr1_54774724_54781254 342.3918325 177.1157998 507.6678653 2.866304789 1.519192027 0.197148937 1

chr1_54849547_54851462 103.964705 50.03271181 157.8966983 3.155869281 1.658037449 0.450076071 1

chr1_54861812_54863775 91.96208638 34.02224403 149.9019287 4.405997693 2.13946874 0.370832814 1

chr1_55007225_55008655 85.9738575 46.03009487 125.9176201 2.735549872 1.451830858 0.552791194 1

chr1_55046272_55048250 154.9522895 82.05364737 227.8509317 2.77685318 1.4734509 0.399568083 1

chr1_55322414_55328716 527.9948807 520.3402028 535.6495586 1.02942182 0.041834268 0.967141889 1

chr1_55348660_55356382 2310.797747 2002.309127 2619.286368 1.308132862 0.387509077 0.505145505 1

chr1_55380403_55382213 69.98235637 43.02813216 96.93658058 2.252865177 1.171760978 0.672146109 1

chr1_55387031_55390089 237.5226146 272.1779523 202.8672769 0.745347943 -0.424014034 0.760298263 1

chr1_55398864_55403734 526.0354297 580.379457 471.6914024 0.812729322 -0.29915315 0.755433789 1

chr1_55415513_55417019 238.4820654 211.1380438 265.8260869 1.259015581 0.332296137 0.811259242 1

chr1_55487601_55488748 81.49445824 73.04775925 89.94115723 1.231265109 0.300141428 0.908153867 1

chr1_55504685_55506137 142.018343 170.1112202 113.9254658 0.669711649 -0.578388032 0.750836691 1

chr1_55659577_55662426 122.4473774 42.02747792 202.8672769 4.827015251 2.271131385 0.264854202 1

chr1_55679220_55682719 1579.405832 1435.938829 1722.872834 1.199823279 0.262821928 0.683261698 1

chr1_55708693_55713606 460.8110861 172.1125286 749.5096436 4.354765162 2.122594921 0.043346785 1

chr1_55717056_55720624 217.4166587 90.05888126 344.7744361 3.82832244 1.936712346 0.192230965 1

chr1_55725308_55730184 550.3415173 308.2015048 792.4815299 2.571309736 1.362503405 0.152380271 1

chr1_55742280_55743609 60.4754871 23.01504743 97.93592677 4.255299801 2.089260779 0.49932857 1

chr1_55893421_55897235 217.4604782 157.1027151 277.8182412 1.768385996 0.822433214 0.570645123 1

chr1_56099154_56101661 139.9339737 39.02551521 240.8424322 6.171409418 2.625600008 0.170908537 1

chr1_56149080_56151984 202.9614561 144.09421 261.8287022 1.817066086 0.861610891 0.56642392 1

chr1_56157654_56163590 488.3284236 226.1478574 750.5089898 3.318665047 1.730603026 0.087089531 1

chr1_56165357_56166560 122.4722302 80.0523389 164.8921216 2.059803922 1.04250701 0.598053613 1

chr1_56183442_56185616 87.96927977 41.02682369 134.9117359 3.288378766 1.717376483 0.478982272 1

chr1_56247063_56252469 1262.174894 765.5004907 1758.849297 2.297646204 1.200156666 0.085274677 1

chr1_56298169_56300519 121.4526094 49.03205758 193.8731612 3.95400827 1.983315889 0.327864574 1

chr1_56353278_56354075 38.50556742 47.0307491 29.98038574 0.637463496 -0.649585366 0.878120616 1

chr1_56380470_56384876 388.1204231 572.3742231 203.8666231 0.356177156 -1.489333104 0.180245619 1

chr1_56664578_56669777 195.9202512 74.04841348 317.7920889 4.291679915 2.10154248 0.181955729 1

chr1_56854424_56856841 307.3977113 151.0987897 463.6966329 3.068830888 1.617689146 0.192813901 1

chr1_56897694_56899838 114.4650343 61.03990841 167.8901602 2.750498232 1.459692976 0.480267624 1

chr1_56957117_56959034 135.9607877 76.04972195 195.8718535 2.575576195 1.364895221 0.466417545 1

chr1_57012477_57014658 203.950992 129.0843965 278.8175874 2.15996352 1.111006947 0.459926789 1

chr1_57041989_57045849 1574.328656 1312.858358 1835.798954 1.398322174 0.483696797 0.45316866 1

chr1_57055394_57057346 86.46633635 35.02289827 137.9097744 3.937703081 1.97735433 0.423009428 1

chr1_57110055_57112374 623.3696559 424.2773962 822.4619156 1.938500432 0.954941056 0.287090105 1

chr1_57220285_57221853 51.47940931 20.01308472 82.94573389 4.144575163 2.051224227 0.550384819 1

chr1_57252444_57255544 210.9562256 144.09421 277.8182412 1.928031954 0.947128962 0.52064583 1

chr1_57283122_57287014 1177.715098 742.4854433 1612.944753 2.172358755 1.119262377 0.115982339 1

chr1_57301408_57303741 110.9529341 39.02551521 182.880353 4.686173957 2.22841051 0.300031882 1

chr1_57429066_57430757 86.45848808 23.01504743 149.9019287 6.513213981 2.703369625 0.285206535 1

chr1_57580446_57587256 471.3742015 279.1825319 663.5658712 2.376817298 1.24903101 0.218074539 1

chr1_58139752_58141579 76.46633421 25.01635591 127.9163125 5.11330719 2.354256701 0.38152153 1

chr1_58288977_58291559 152.4473838 72.04710501 232.8476626 3.231880901 1.692374034 0.340123074 1

chr1_58628499_58630024 61.46763905 12.00785083 110.9274273 9.237908497 3.207566256 0.313979008 1

chr1_58638582_58641296 171.4055304 27.01766438 315.7933965 11.68840474 3.547006136 0.047768052 1

chr1_58647785_58652331 237.3721894 42.02747792 432.7169009 10.29604731 3.364018682 0.025465987 1

chr1_58897962_58899519 53.46894538 6.003925417 100.9339653 16.81132898 4.071361872 0.261723937 1

chr1_58910639_58915633 192.9078241 52.03402028 333.781628 6.414680744 2.681377465 0.098293926 1

chr1_59010309_59013297 612.9621978 555.3631011 670.5612945 1.207428605 0.271937885 0.762847849 1

chr1_59041713_59043914 240.0746099 354.2315996 125.9176201 0.355466933 -1.492212736 0.284961363 1

chr1_59082008_59089783 1717.837516 1469.961073 1965.713959 1.337255792 0.419275452 0.504794697 1

chr1_59152134_59154155 100.983017 75.04906772 126.9169663 1.691119826 0.757978887 0.731748988 1

chr1_59164029_59166656 623.9164186 496.3245012 751.508336 1.514147164 0.598505432 0.502769068 1

chr1_59218573_59221522 124.4669985 74.04841348 174.8855835 2.361773538 1.239870637 0.527904998 1

chr1_59238381_59240304 159.9392101 67.04383383 252.8345864 3.771183299 1.915017275 0.271134055 1

chr1_59246891_59253145 2535.668953 2030.327445 3041.010461 1.497793111 0.582838359 0.306215046 1

chr1_59289053_59291924 170.4742026 131.0857049 209.8627002 1.60095794 0.678935407 0.680085352 1

chr1_59306696_59307741 41.48202326 14.00915931 68.95488721 4.922128852 2.299282425 0.566343154 1

chr1_59323142_59324862 79.47222105 37.02420674 121.9202354 3.292987105 1.719396862 0.505123718 1

chr1_59335058_59336980 115.9673237 66.04317959 165.8914678 2.511863735 1.328758202 0.516367599 1

chr1_59346830_59354004 598.8045754 300.1962709 897.41288 2.989420479 1.579865835 0.087548519 1

chr1_59367616_59371126 664.905309 520.3402028 809.4704151 1.555656109 0.637523175 0.464086333 1

chr1_59387645_59389340 69.96665983 19.01243049 120.9208892 6.360096319 2.669048614 0.353021358 1

chr1_59397200_59400128 422.9059112 279.1825319 566.6292906 2.029601518 1.021196504 0.334816569 1

chr1_59431207_59432705 60.97189008 18.01177625 103.9320039 5.770225127 2.528627607 0.417558589 1

chr1_59679437_59682010 150.9496726 74.04841348 227.8509317 3.077053524 1.621549539 0.362385198 1

chr1_59761340_59763940 642.4455265 559.3657181 725.525335 1.297050054 0.375234156 0.670742067 1

chr1_59891289_59894920 382.0301667 428.2800131 335.7803203 0.784020524 -0.351036673 0.750937314 1

chr1_60069975_60071563 74.9784333 42.02747792 107.9293887 2.568067227 1.36068297 0.608480446 1

chr1_60233965_60235954 94.47876448 62.04056265 126.9169663 2.045709467 1.032601267 0.653388489 1

chr1_60279435_60281849 719.9681069 671.4389925 768.4972213 1.144552565 0.194783722 0.818074278 1

chr1_60321738_60323717 167.4807422 138.0902846 196.8711997 1.425670171 0.511640253 0.758744297 1

chr1_60385339_60389122 428.9235711 312.2041217 545.6430206 1.747712418 0.805467813 0.442611712 1

chr1_60391511_60393042 266.4840334 242.1583252 290.8097417 1.20090747 0.264124996 0.84148564 1

chr1_60745808_60748155 112.9431242 26.01701014 199.8692383 7.682252388 2.941529362 0.178673959 1

chr1_61005542_61009831 160.9123954 27.01766438 294.8071265 10.91164367 3.447796533 0.061487847 1

chr1_61036427_61042030 697.7228438 274.1792607 1121.266427 4.089537713 2.031937768 0.021302103 1

chr1_61214467_61215656 41.47548304 4.002616945 78.94834913 19.72418301 4.301893639 0.323371379 1

chr1_61215710_61216998 43.47875358 11.0071966 75.95031055 6.900059418 2.786608785 0.479626463 1

chr1_61313211_61315541 161.9248221 47.0307491 276.818895 5.885912947 2.557266205 0.148113552 1

chr1_61436029_61437225 64.01178609 82.05364737 45.96992481 0.560242308 -0.835877158 0.775915911 1

chr1_61508377_61509488 36.47875208 4.002616945 68.95488721 17.22745098 4.106637347 0.384344193 1

chr1_61515225_61517270 1069.314159 785.5135755 1353.114743 1.722586071 0.78457607 0.283347534 1

chr1_61522091_61524029 606.363112 397.2597318 815.4664923 2.052728799 1.037543035 0.253418025 1

chr1_61532653_61534112 66.47418034 27.01766438 105.9306963 3.920793997 1.971145843 0.496944071 1

chr1_61589342_61593451 289.3761247 100.0654236 478.6868257 4.783738562 2.258138546 0.083613321 1

chr1_61597556_61600493 521.3506674 293.1916912 749.5096436 2.556380914 1.354102821 0.164315605 1

chr1_61648425_61651183 289.8856081 115.0752372 464.695979 4.038192668 2.013709746 0.119359359 1

chr1_61691623_61693834 157.9934936 148.096827 167.8901602 1.133651298 0.180976948 0.918758921 1

chr1_61706534_61708127 84.96993315 39.02551521 130.9143511 3.354583543 1.746133673 0.481156149 1

chr1_61776769_61778311 70.48987773 55.03598299 85.94377247 1.561592395 0.643017932 0.816527289 1

chr1_61785098_61790867 520.5416417 584.382074 456.7012095 0.781511326 -0.355661313 0.712082596 1

chr1_61802605_61805139 243.4650619 190.1243049 296.8058189 1.561114551 0.642576403 0.638851823 1

chr1_61868904_61870652 107.4728811 66.04317959 148.9025825 2.254624678 1.172887292 0.582602106 1

chr1_61887973_61892509 354.9471001 274.1792607 435.7149395 1.589160822 0.668265132 0.558388809 1

chr1_61906564_61908388 91.980399 62.04056265 121.9202354 1.965169724 0.974653918 0.676542514 1

chr1_61910435_61912895 128.5526763 209.1367354 47.96861719 0.229364856 -2.124283741 0.281538406 1

chr1_61917828_61920326 205.4859825 184.1203795 226.8515855 1.232082978 0.301099422 0.841581131 1

chr1_62034488_62035978 75.51865579 104.0680406 46.969271 0.451332328 -1.147737976 0.663575826 1

chr1_62189485_62192591 784.448173 705.4612366 863.4351095 1.223929912 0.291520945 0.7211171 1

chr1_62330635_62333339 168.4617757 110.071966 226.8515855 2.0609388 1.043301664 0.529649713 1

chr1_62359616_62360008 26.50164072 29.01897285 23.9843086 0.826504395 -0.274905604 0.966252417 1

chr1_62644367_62647996 292.9353144 194.1269218 391.7437071 2.017977225 1.012909892 0.419370004 1

chr1_62693800_62697681 281.9582029 218.1426235 345.7737823 1.58508125 0.664556793 0.601825985 1

chr1_62764663_62766375 78.98235829 52.03402028 105.9306963 2.035796883 1.025593627 0.689227023 1

chr1_62772416_62776371 378.4369678 282.1844946 474.689441 1.682195337 0.750345241 0.498777693 1

chr1_62784277_62785345 86.0235632 122.0798168 49.96730957 0.409300332 -1.288768257 0.597186267 1

chr1_62900580_62904388 1608.957833 1545.010141 1672.905525 1.08277964 0.114739666 0.858401648 1

chr1_63152550_63155088 954.9243377 839.5489042 1070.299771 1.274851013 0.350328655 0.644311753 1

chr1_63248465_63250767 564.947145 484.3166503 645.5776397 1.332966024 0.414640008 0.655814557 1

chr1_63787083_63790302 642.9249249 528.3454367 757.5044132 1.433729451 0.519772809 0.55567898 1

chr1_63832252_63834427 664.4442232 579.3788028 749.5096436 1.293643537 0.371440138 0.669697115 1

chr1_63987785_63990374 497.9575951 433.2832843 562.6319058 1.298531299 0.376880788 0.701130546 1

chr1_64058562_64060441 252.0007079 253.1655218 250.8358941 0.990798006 -0.01333713 0.995426716 1

chr1_64139465_64142683 197.3872234 25.01635591 369.7580909 14.78065359 3.885638161 0.021791357 1

chr1_64163697_64170591 450.7888472 128.0837422 773.4939522 6.038970588 2.594302647 0.016306266 1

chr1_64237335_64241212 542.3081605 249.1629048 835.4534161 3.353040922 1.74547009 0.071368817 1

chr1_64281799_64284138 87.45194807 14.00915931 160.8947368 11.48496732 3.521674847 0.175849032 1

chr1_64296295_64297622 44.97711884 10.00654236 79.94769532 7.989542484 2.99811289 0.439902639 1

chr1_64333288_64338634 184.8940879 23.01504743 346.7731284 15.06723501 3.913342787 0.025673878 1

chr1_64444103_64450597 987.7104794 545.3565588 1430.0644 2.622255801 1.390808428 0.067514087 1

chr1_64471490_64473258 72.46371726 17.01112202 127.9163125 7.519569396 2.910650049 0.305145977 1

chr1_64809264_64811435 202.0098535 217.1419693 186.8777378 0.86062468 -0.216543882 0.887739995 1

chr1_64869474_64871059 69.48987752 54.03532876 84.94442628 1.572016461 0.652616324 0.815464904 1

chr1_64935012_64937041 642.4514127 568.3716062 716.5312193 1.260673847 0.33419508 0.705082374 1

chr1_64970318_64973554 323.9490555 246.1609421 401.737169 1.632010202 0.706650076 0.553273282 1

chr1_65030127_65031615 86.00198046 89.05822703 82.94573389 0.931365205 -0.102581109 0.971098514 1

chr1_65209678_65212200 903.3736399 710.4645077 1096.282772 1.543050723 0.625785486 0.41924685 1

chr1_65363279_65364762 144.4683108 96.06280668 192.873815 2.007788671 1.005607427 0.576994147 1

chr1_65379216_65384672 640.3421906 399.2610403 881.4233409 2.207636739 1.1425028 0.199473569 1

chr1_65430714_65434231 940.3834582 762.498528 1118.268388 1.466584324 0.552460024 0.46914128 1

chr1_65446629_65449142 343.4166856 216.141315 470.6920562 2.177705156 1.122808638 0.335517512 1

chr1_65467546_65469477 255.4349794 156.1020609 354.767898 2.272666331 1.184385886 0.378349215 1

chr1_65531155_65535116 1473.949956 1397.913968 1549.985943 1.108784931 0.148979556 0.821190053 1

chr1_65612474_65615377 739.7326631 1095.716389 383.7489375 0.35022652 -1.513639763 0.074567408 1

chr1_65712521_65714739 99.53631952 155.1014066 43.97123243 0.283499895 -1.818579896 0.421152802 1

chr1_65719396_65721180 270.0000577 270.1766438 269.8234717 0.99869281 -0.00188711 1 1

chr1_65881794_65888114 1061.932209 958.6267583 1165.237659 1.215527993 0.281583118 0.700231642 1

chr1_66258390_66259169 42.50949241 57.03729147 27.98169336 0.490585942 -1.027422202 0.791097116 1

chr1_66735310_66740654 547.4029948 399.2610403 695.5449493 1.742080692 0.80081145 0.396402813 1

chr1_66801133_66803710 313.0288439 357.2335623 268.8241255 0.752516431 -0.410205011 0.735255329 1

chr1_66900906_66903355 124.9660176 73.04775925 176.8842759 2.421488047 1.275893882 0.515283183 1

chr1_66985780_66986923 39.98104191 11.0071966 68.95488721 6.264527629 2.647205729 0.524477427 1

chr1_67389264_67392319 745.9393355 653.4272163 838.4514547 1.283159675 0.359700709 0.665505198 1

chr1_67395092_67397782 646.9124994 513.3356232 780.4893756 1.520427066 0.604476613 0.492232865 1

chr1_67840421_67844623 455.9602022 395.2584233 516.661981 1.30714983 0.386424518 0.704917259 1

chr1_67859000_67860184 39.48136881 11.0071966 67.95554102 6.173737374 2.626144113 0.531147935 1

chr1_68017777_68018900 60.49576179 54.03532876 66.95619483 1.239118857 0.309314579 0.922723912 1

chr1_68109630_68116358 624.2545482 249.1629048 999.3461915 4.0108145 2.003895243 0.029643698 1

chr1_68149640_68155166 1723.286176 1396.913314 2049.659039 1.467277188 0.553141441 0.378875196 1

chr1_68164114_68168236 293.3964002 135.0883219 451.7044786 3.343771484 1.741476256 0.171813569 1

chr1_68213784_68217806 993.7366416 591.3866536 1396.08663 2.360700264 1.239214876 0.101337531 1

chr1_68256820_68259125 175.0222742 209.1367354 140.907813 0.673759264 -0.569694889 0.725916792 1

chr1_68296929_68299933 701.0740545 814.5325483 587.6155606 0.721414463 -0.471099749 0.580355972 1

chr1_68346189_68347077 46.00785811 58.0379457 33.97777051 0.585440613 -0.772405263 0.834144924 1

chr1_68362007_68364633 166.9385576 73.04775925 260.829356 3.570668815 1.836194328 0.279252582 1

chr1_68515342_68516951 131.4794264 100.0654236 162.8934292 1.627869281 0.702984855 0.711221038 1

chr1_68610213_68612161 94.9732054 54.03532876 135.911082 2.515226337 1.33068823 0.562830479 1

chr1_68638119_68644692 718.8163735 438.2865555 999.3461915 2.280120572 1.189110116 0.162603214 1

chr1_68960846_68963704 340.4539642 270.1766438 410.7312847 1.520232389 0.604291877 0.603675715 1

chr1_69346872_69347842 45.00589583 54.03532876 35.97646289 0.665795207 -0.58684961 0.876943346 1

chr1_70032574_70034554 153.5121322 172.1125286 134.9117359 0.783857729 -0.351336267 0.841737992 1

chr1_70670129_70672878 569.4252362 455.2976775 683.552795 1.50133161 0.58624267 0.527211049 1

chr1_70686036_70688571 671.9654805 619.4049722 724.5259888 1.169712904 0.226154476 0.794748385 1

chr1_70799552_70800702 37.4820224 10.00654236 64.95750245 6.491503268 2.698552609 0.536116052 1

chr1_70818610_70821493 877.4181079 752.4919857 1002.34423 1.332033097 0.413629929 0.597008123 1

chr1_71095920_71097362 46.47483008 8.00523389 84.94442628 10.61111111 3.407503827 0.377427595 1

chr1_71133234_71134566 50.9764661 15.00981354 86.94311866 5.792418301 2.534165791 0.470011497 1

chr1_71150544_71152764 151.4375733 56.03663723 246.8385093 4.404948646 2.1391252 0.235926639 1

chr1_71182068_71186271 270.4702998 225.1472032 315.7933965 1.402608569 0.488112447 0.707494483 1

chr1_71254600_71258093 138.9457459 56.03663723 221.8548545 3.959103641 1.985173835 0.291409332 1

chr1_71312836_71314190 63.48791417 45.02944063 81.9463877 1.819840232 0.863811799 0.769751978 1

chr1_71338017_71342590 702.3892935 533.3487079 871.429879 1.633883923 0.708305492 0.406129409 1

chr1_71456565_71459396 111.4539153 41.02682369 181.8810069 4.433221744 2.148355526 0.315202171 1

chr1_71512639_71514314 134.5507155 212.1386981 56.96273292 0.268516463 -1.89691755 0.320619277 1

chr1_71545345_71548156 707.9563319 641.4193654 774.4932984 1.207467907 0.271984844 0.749055302 1

chr1_72747919_72750639 471.9850584 449.2937521 494.6763648 1.101008778 0.138825971 0.891541788 1

chr1_72904252_72906370 83.46371962 28.01831861 138.9091206 4.957796452 2.309699041 0.36452295 1

chr1_73143848_73147694 181.9411769 92.06018973 271.8221641 2.952657005 1.562013776 0.331909916 1

chr1_73157173_73161431 825.4010922 674.4409552 976.3612291 1.447660053 0.533722861 0.504923164 1

chr1_74056048_74060835 510.4154133 381.249264 639.5815626 1.677594222 0.746393798 0.442565787 1

chr1_74082454_74084280 48.97384958 9.005888126 88.94181104 9.875962237 3.30392132 0.373210933 1

chr1_74662971_74664953 268.9686645 221.1445862 316.7927427 1.432514122 0.518549361 0.690879147 1

chr1_75117190_75119776 186.0301248 232.1517828 139.9084668 0.602659455 -0.730585088 0.642056838 1

chr1_75155611_75156453 29.98757999 11.0071966 48.96796338 4.448722519 2.153391116 0.67046655 1

chr1_75197564_75200087 544.4533539 473.3094537 615.597254 1.300623195 0.379203058 0.688271857 1

chr1_75408868_75410823 73.4643715 19.01243049 127.9163125 6.728035776 2.750185377 0.325456257 1

chr1_75436694_75438891 85.94965867 9.005888126 162.8934292 18.08743646 4.176916043 0.12377306 1

chr1_75518881_75520377 63.46633143 12.00785083 114.924812 9.5708061 3.258640441 0.297565882 1

chr1_75532957_75534540 59.47483286 21.01373896 97.93592677 4.660566449 2.220505312 0.479205311 1

chr1_75557223_75558681 71.97123841 28.01831861 115.9241582 4.137441643 2.048738964 0.458944163 1

chr1_75599985_75601854 208.423851 92.06018973 324.7875122 3.527990907 1.818846842 0.228925647 1

chr1_75668223_75669869 54.97581294 18.01177625 91.93984962 5.10442992 2.351749845 0.478295292 1

chr1_75838799_75839940 49.97908198 18.01177625 81.9463877 4.549600581 2.185739894 0.534223426 1

chr1_75926870_75928043 47.48202454 20.01308472 74.95096436 3.745098039 1.905003486 0.598232904 1

chr1_76080090_76082893 436.9346912 337.2204776 536.6489048 1.59138884 0.670286387 0.518967257 1

chr1_76137445_76139760 223.9791192 192.1256134 255.832625 1.331590414 0.41315039 0.773040058 1

chr1_76188743_76191386 634.9922875 623.4075892 646.5769859 1.037165728 0.052646439 0.954359692 1

chr1_77332814_77334094 62.01832589 90.05888126 33.97777051 0.377283951 -1.406277365 0.639128138 1

chr1_77504099_77505384 61.51538268 85.05561008 37.97515528 0.446474433 -1.163350532 0.699012211 1

chr1_77683987_77686120 334.4650813 281.1838404 387.7463223 1.378977973 0.463599412 0.693062554 1

chr1_77747059_77748507 139.0000297 139.0909388 138.9091206 0.99869281 -0.00188711 1 1

chr1_77906975_77908549 52.97712055 18.01177625 87.94246485 4.882498184 2.287619508 0.500075038 1

chr1_77912167_77915315 278.4683395 230.1504743 326.7862046 1.419880648 0.505769665 0.693227892 1

chr1_78070315_78072504 127.4578428 63.04121688 191.8744688 3.043635232 1.605795468 0.410873857 1

chr1_78146811_78150317 1226.938784 1133.74125 1320.136319 1.164407063 0.219595496 0.752806725 1

chr1_78223709_78226040 546.4356957 448.2930978 644.5782935 1.43785014 0.523913319 0.578559453 1

chr1_78244381_78246445 322.9928748 312.2041217 333.781628 1.069113457 0.096414964 0.937891795 1

chr1_78277213_78277812 35.00131553 37.02420674 32.97842432 0.89072602 -0.166946356 0.976391298 1

chr1_78442000_78446331 1104.450858 1029.673209 1179.228506 1.1452454 0.195656768 0.786612839 1

chr1_78469779_78471371 114.9961005 109.0713118 120.9208892 1.108640643 0.148791803 0.945776159 1

chr1_78702013_78703460 73.48857033 56.03663723 90.94050343 1.622875817 0.698552609 0.795335844 1

chr1_78956215_78958011 80.48399367 56.03663723 104.9313501 1.87254902 0.905003486 0.72111292 1

chr1_79695213_79698958 334.4467687 253.1655218 415.7280157 1.642119404 0.715559034 0.542274959 1

chr1_79719660_79723684 291.431063 186.1216879 396.740438 2.131618526 1.091949276 0.385784128 1

chr1_79856421_79859630 144.9725621 103.0673863 186.8777378 1.813160734 0.858506823 0.632967664 1

chr1_79982318_79983100 38.00589433 47.0307491 28.98103955 0.616214713 -0.698494966 0.86983665 1

chr1_80517107_80520907 384.8660078 180.1177625 589.614253 3.273493101 1.710830938 0.127504234 1

chr1_80548066_80551733 165.9379034 71.04645077 260.829356 3.671251036 1.876271768 0.270990431 1

chr1_80596663_80597993 72.46567933 20.01308472 124.9182739 6.241830065 2.64196908 0.346963763 1

chr1_81057472_81061718 1071.25726 700.4579654 1442.056554 2.058733894 1.041757363 0.155773624 1

chr1_81196069_81198114 128.9470518 48.03140334 209.8627002 4.369281046 2.127395907 0.280073067 1

chr1_81471478_81476249 377.0478243 450.2944063 303.8012422 0.674672476 -0.567740787 0.609112441 1

chr1_81563808_81565136 74.0314089 122.0798168 25.98300098 0.212836173 -2.232184729 0.414140926 1

chr1_81964442_81965432 56.5062253 66.04317959 46.969271 0.711190335 -0.491692377 0.879677172 1

chr1_81990705_81991830 47.50883946 61.03990841 33.97777051 0.556648452 -0.845161606 0.814231269 1

chr1_82135777_82139609 208.4088085 69.0451423 347.7724746 5.036885479 2.33253193 0.12941981 1

chr1_82143712_82146296 381.3617559 170.1112202 592.6122916 3.483675509 1.800610249 0.111152335 1

chr1_82240432_82242463 67.47091044 23.01504743 111.9267734 4.863199773 2.281905856 0.431498175 1

chr1_82265147_82269555 1301.980658 1272.832189 1331.129127 1.045800962 0.064608302 0.92561975 1

chr1_82627848_82628825 53.50491661 61.03990841 45.96992481 0.753112611 -0.409062491 0.904591513 1

chr1_83516371_83523766 906.5554588 991.6483481 821.4625694 0.828380918 -0.271633773 0.72557166 1

chr1_83599264_83601175 150.4473834 70.04579654 230.8489702 3.295686275 1.720578915 0.335946171 1

chr1_84157788_84159318 69.48333729 44.02878639 94.93788819 2.156268568 1.10853688 0.690136121 1

chr1_84303776_84306839 216.8895166 48.03140334 385.7476299 8.031154684 3.005607427 0.052401633 1

chr1_84325957_84327507 479.4494158 402.263003 556.6358287 1.383760934 0.468594717 0.638523217 1

chr1_84348749_84349849 42.97646439 7.004579654 78.94834913 11.27096172 3.494538717 0.392644912 1

chr1_84365861_84368639 80.95423576 11.0071966 150.9012749 13.70932858 3.777086011 0.170713773 1

chr1_84463814_84465926 556.3801059 373.2440301 739.5161817 1.981320857 0.986462531 0.294179 1

chr1_84483420_84488335 457.3624261 247.1615964 667.5632559 2.700918208 1.433449951 0.164811353 1

chr1_84542767_84544948 633.8745633 442.2891724 825.4599542 1.866335433 0.900208302 0.311938242 1

chr1_84568410_84569816 63.97777693 30.01962709 97.93592677 3.262396514 1.705932139 0.563676938 1

chr1_84582806_84584450 70.46306281 14.00915931 126.9169663 9.059570495 3.179442655 0.276246317 1

chr1_84596680_84598202 66.98039366 37.02420674 96.93658058 2.618194665 1.388572367 0.62650702 1

chr1_84598992_84599992 38.98561985 17.01112202 60.96011768 3.58354479 1.841387387 0.656846345 1

chr1_84608477_84610564 103.9810556 75.04906772 132.9130435 1.771015251 0.824576635 0.704228477 1

chr1_84689733_84691788 90.96143214 32.02093556 149.9019287 4.681372549 2.226931581 0.356065414 1

chr1_84725516_84727524 134.9575174 70.04579654 199.8692383 2.85340803 1.512686063 0.422906982 1

chr1_84763546_84769552 1211.280181 875.5724567 1546.987904 1.766830252 0.82116344 0.241990291 1

chr1_84872060_84879317 924.3788766 739.4834806 1109.274273 1.500066332 0.585026298 0.446258657 1

chr1_84898591_84900807 131.468308 83.05430161 179.8823145 2.16583983 1.114926555 0.557681974 1

chr1_84908138_84910034 88.47680113 53.03467452 123.9189277 2.336564311 1.224388746 0.609306313 1

chr1_84944110_84946396 507.4468057 426.2787046 588.6149068 1.38082175 0.465527094 0.63245832 1

chr1_84969300_84973182 1134.899523 981.6418058 1288.157241 1.31224774 0.392040113 0.58326051 1

chr1_85002917_85005140 65.46567783 13.00850507 117.9228506 9.065057818 3.180316222 0.29825413 1

chr1_85038230_85040622 229.4552486 161.105332 297.8051651 1.848512158 0.886364533 0.530196285 1

chr1_85068027_85070970 244.9699674 199.130193 290.8097417 1.460400039 0.546363613 0.689097385 1

chr1_85085891_85088574 368.9000135 216.141315 521.658712 2.413507625 1.271131385 0.261247678 1

chr1_85154604_85157625 775.9314936 671.4389925 880.4239947 1.311249428 0.390942143 0.633254526 1

chr1_85298666_85302208 733.8464617 499.3264639 968.3664596 1.939345357 0.955569741 0.255874874 1

chr1_85362838_85367469 517.8398753 273.1786065 762.5011441 2.791218368 1.480894996 0.130596514 1

chr1_85377151_85381135 230.8738231 38.02486098 423.7227852 11.14330925 3.478105832 0.023433199 1

chr1_85399132_85402516 215.4061939 72.04710501 358.7652827 4.979593319 2.316027923 0.125537199 1

chr1_85462018_85463882 265.4990757 264.1727184 266.8254331 1.010041592 0.014414703 0.994466109 1

chr1_85513201_85514888 301.9797899 271.177298 332.7822818 1.227176036 0.295342216 0.811581299 1

chr1_85576592_85579017 98.45456651 29.01897285 167.8901602 5.785530764 2.532449318 0.276726766 1

chr1_85599192_85604099 1562.57522 913.5973177 2211.553122 2.42070886 1.275429576 0.051258119 1

chr1_85611486_85613436 259.9137247 128.0837422 391.7437071 3.058496732 1.612822735 0.230938328 1

chr1_85677959_85683790 1096.375643 906.592738 1286.158548 1.4186729 0.504541989 0.485381524 1

chr1_85723809_85726329 452.4219411 333.2178607 571.6260215 1.715472335 0.77860586 0.44721646 1

chr1_85740460_85746105 1254.229176 840.5495584 1667.908794 1.984307501 0.988635612 0.155389314 1

chr1_85759196_85766488 1469.692924 1000.654236 1938.731612 1.937464052 0.954169543 0.149435737 1

chr1_85779069_85780716 109.4951183 102.0667321 116.9235044 1.1455594 0.196052268 0.929255649 1

chr1_85929371_85932347 1106.374337 914.5979719 1298.150703 1.419367572 0.505248251 0.483426441 1

chr1_86041769_86043875 841.8857262 667.4363756 1016.335077 1.52274451 0.606673903 0.445364843 1

chr1_86052937_86055628 160.4101062 23.01504743 297.8051651 12.93958511 3.693719455 0.04807632 1

chr1_86076779_86079367 115.4598023 54.03532876 176.8842759 3.273493101 1.710830938 0.408476752 1

chr1_86093474_86095864 189.9503349 114.0745829 265.8260869 2.330283224 1.220505312 0.434743425 1

chr1_86172667_86175257 555.973304 515.3369317 596.6096763 1.157707976 0.21127139 0.822362426 1

chr1_86283238_86284784 71.99347518 62.04056265 81.9463877 1.320851782 0.401468585 0.885188369 1

chr1_86416424_86417715 68.47941295 37.02420674 99.93461915 2.699169758 1.432515715 0.610849802 1

chr1_86569957_86571958 216.9843499 193.1262676 240.8424322 1.24707237 0.318545189 0.82754696 1

chr1_86742363_86744062 94.46110588 35.02289827 153.8993135 4.394248366 2.135616414 0.364063381 1

chr1_86859439_86863217 857.7993986 551.3604842 1164.238313 2.111573728 1.078318621 0.174816465 1

chr1_86887729_86890877 538.3264722 273.1786065 803.474338 2.9412052 1.556407441 0.10702461 1

chr1_86893684_86898321 1093.734047 687.4494603 1500.018633 2.182005689 1.125654863 0.123063236 1

chr1_87169418_87172253 1010.894265 849.5554466 1172.233083 1.379819395 0.464479445 0.532208396 1

chr1_87245875_87247531 442.3094471 151.0987897 733.5201046 4.854572999 2.279344404 0.034092704 1

chr1_87258170_87259820 402.3375615 154.1007524 650.5743707 4.221746881 2.077840083 0.062047319 1

chr1_87378216_87382004 1008.395899 849.5554466 1167.236352 1.373937812 0.458316706 0.538008941 1

chr1_87597099_87598457 146.4826997 120.0785083 172.8868911 1.439782135 0.525850522 0.769236567 1

chr1_87666588_87668352 96.98170812 69.0451423 124.9182739 1.809226106 0.855372718 0.705507616 1

chr1_87759338_87760897 82.50230672 86.05626432 78.94834913 0.917403861 -0.124371116 0.964884764 1

chr1_87821399_87825188 189.426463 77.05037619 301.8025498 3.916951023 1.969731089 0.216876432 1

chr1_87854883_87860994 592.8562419 373.2440301 812.4684537 2.176775482 1.122192612 0.22170608 1

chr1_87864163_87865762 102.9771312 68.04448806 137.9097744 2.026758939 1.019174506 0.640892733 1

chr1_87873514_87880609 824.7163307 391.2558064 1258.176855 3.215739766 1.685150661 0.039549137 1

chr1_87937849_87941105 204.0000436 204.1334642 203.8666231 0.99869281 -0.00188711 1 1

chr1_87994581_87996639 95.97320561 55.03598299 136.9104282 2.487653001 1.31478526 0.565001808 1

chr1_88141549_88145600 388.9732683 348.2276742 429.7188623 1.234016978 0.303362244 0.782390241 1

chr1_88218532_88222681 283.5056198 292.191037 274.8202027 0.940549736 -0.08842386 0.947050164 1

chr1_88299789_88301611 100.9895572 85.05561008 116.9235044 1.37467128 0.459086674 0.836949034 1

chr1_88316810_88317816 49.0065507 59.03859994 38.97450147 0.660152875 -0.59912794 0.866333165 1

chr1_88365613_88371035 520.9661023 469.3068368 572.6253677 1.220151344 0.287060107 0.766042071 1

chr1_88416258_88420886 361.9954993 355.2322539 368.7587447 1.038077879 0.053914682 0.964429916 1

chr1_88446723_88448770 199.4330054 97.06346092 301.8025498 3.109332255 1.636604788 0.287335366 1

chr1_88525645_88527398 70.01440346 92.06018973 47.96861719 0.521057118 -0.940486565 0.733986199 1

chr1_88759728_88760706 32.01570338 56.03663723 7.994769532 0.142670401 -2.809242032 0.567207182 1

chr1_89148012_89151536 972.9210714 852.5574093 1093.284733 1.282359078 0.358800293 0.634057231 1

chr1_89392412_89395490 207.9405284 117.0765456 298.8045113 2.55221496 1.351749845 0.366002559 1

chr1_89456892_89459933 718.9641825 664.4344129 773.4939522 1.164138909 0.219263215 0.795588608 1

chr1_89483035_89486609 305.0726617 416.2721623 193.8731612 0.465736551 -1.102413985 0.370909128 1

chr1_89590126_89594742 371.0988367 522.3415113 219.8561621 0.420905016 -1.248433393 0.268325066 1

chr1_89989153_89991847 1177.959049 1115.729473 1240.188624 1.111549576 0.152572296 0.829449071 1

chr1_90098014_90099746 237.0183633 265.1733726 208.863354 0.787648292 -0.344376527 0.805027474 1

chr1_90158354_90159815 50.51734239 77.05037619 23.9843086 0.311280876 -1.68371115 0.625579417 1

chr1_90355906_90357370 130.9934878 121.0791626 140.907813 1.163766002 0.218801006 0.911251898 1

chr1_90459259_90462710 838.4298719 731.4782467 945.3814972 1.29242599 0.370081668 0.64187641 1

chr1_91316219_91317608 178.0033082 183.1197252 172.8868911 0.944119433 -0.08295872 0.962264472 1

chr1_91358774_91359880 73.99674571 69.0451423 78.94834913 1.143430899 0.193369182 0.946551796 1

chr1_91442933_91444259 46.97908134 15.00981354 78.94834913 5.259782135 2.395003043 0.516264766 1

chr1_91556941_91558238 63.51342104 84.05495584 42.97188623 0.511235605 -0.967939778 0.742586997 1

chr1_91626106_91627455 76.51800198 104.0680406 48.96796338 0.470537959 -1.087616984 0.67760885 1

chr1_91627470_91628836 73.02486846 111.0726202 34.9771167 0.314903138 -1.667019959 0.539237093 1

chr1_91852614_91853400 59.50949605 74.04841348 44.97057862 0.607313196 -0.719487379 0.815965768 1

chr1_91869622_91870705 47.51930382 77.05037619 17.98823145 0.233460657 -2.098748649 0.56316571 1

chr1_91931279_91933482 126.0418844 190.1243049 61.95946387 0.325889233 -1.617546407 0.410508467 1

chr1_91965636_91967707 415.4618286 357.2335623 473.6900948 1.325995496 0.407075875 0.701862367 1

chr1_92202486_92203854 60.02748177 102.0667321 17.98823145 0.176239908 -2.50438745 0.426290699 1

chr1_92295234_92296888 96.51081201 113.0739287 79.94769532 0.707039158 -0.500137977 0.82697114 1

chr1_92328758_92331057 178.9745314 140.0915931 217.8574697 1.555107376 0.637014198 0.691395283 1

chr1_92350575_92352500 369.4480842 290.1897285 448.70644 1.546251972 0.628775435 0.574797221 1

chr1_92495061_92496351 95.52716235 137.0896304 53.96469434 0.393645341 -1.34503169 0.557356262 1

chr1_92545325_92547242 398.4108112 262.1714099 534.6502125 2.039315472 1.02808497 0.344505392 1

chr1_92763442_92765847 863.421375 743.4860975 983.3566524 1.322629509 0.403408995 0.608241393 1

chr1_92906600_92908804 404.9438407 319.2087014 490.67898 1.537172947 0.620279491 0.563917745 1

chr1_92950441_92952040 253.4591778 191.1249591 315.7933965 1.652287582 0.724464811 0.589360946 1

chr1_92980910_92982473 52.47744746 18.01177625 86.94311866 4.827015251 2.271131385 0.505642555 1

chr1_93249303_93251736 630.9223063 512.334969 749.5096436 1.462928922 0.548859676 0.536919008 1

chr1_93296729_93300026 907.9753413 870.5691855 945.3814972 1.085934941 0.118937673 0.878618946 1

chr1_93324689_93325872 47.98169764 20.01308472 75.95031055 3.79503268 1.924112309 0.592085034 1

chr1_93350485_93353754 258.8770992 71.04645077 446.7077476 6.287544877 2.652496792 0.057869664 1

chr1_93366638_93368376 60.97254411 19.01243049 102.9326577 5.413966288 2.436685904 0.433253833 1

chr1_93379785_93381111 63.48333601 38.02486098 88.94181104 2.339043688 1.225918808 0.677588361 1

chr1_93406846_93411055 238.9052179 94.06149821 383.7489375 4.079766375 2.028486539 0.153479366 1

chr1_93414055_93418314 195.8993225 42.02747792 349.771167 8.322440087 3.05700658 0.06154095 1

chr1_93425278_93428083 668.3265 403.2636572 933.3893429 2.3145883 1.210755602 0.167247324 1

chr1_93460146_93462735 159.9215515 40.02616945 279.8169336 6.990849673 2.805467813 0.118650877 1

chr1_93543274_93546533 960.4102773 823.5384364 1097.282118 1.3323994 0.414026609 0.58449386 1

chr1_93644106_93647068 666.4416075 577.3774943 755.5057208 1.30851259 0.387927806 0.655533578 1

chr1_93786057_93791208 455.9065723 313.2047759 598.6083687 1.911236401 0.934506236 0.36096481 1

chr1_93810018_93813254 1159.981935 1132.740595 1187.223275 1.048098109 0.067773768 0.924925923 1

chr1_93864168_93867393 251.5670911 354.2315996 148.9025825 0.420353754 -1.250324139 0.356600341 1

chr1_94017888_94019837 76.97385557 37.02420674 116.9235044 3.158028617 1.659024244 0.52777281 1

chr1_94100846_94106209 644.0138722 665.4350671 622.5926773 0.935617475 -0.096009287 0.914686395 1

chr1_94144229_94148422 1147.948578 1069.699379 1226.197777 1.146301289 0.196986286 0.782417798 1

chr1_94166242_94168935 153.5297908 199.130193 107.9293887 0.542004138 -0.883624228 0.612059412 1

chr1_94209527_94214251 492.9752526 455.2976775 530.6528277 1.165507434 0.220958206 0.823524541 1

chr1_94233243_94235249 98.48726763 79.05168466 117.9228506 1.491718375 0.576975192 0.797866448 1

chr1_94242069_94248575 853.6980243 1156.756297 550.6397515 0.476020535 -1.070904283 0.178502761 1

chr1_94263986_94265213 70.51015242 86.05626432 54.96404053 0.63869889 -0.646792151 0.815408016 1

chr1_94277644_94280645 183.5350294 237.155054 129.9150049 0.547806183 -0.868262545 0.583516859 1

chr1_94311047_94314381 1630.906824 1488.973504 1772.840144 1.190645864 0.251744374 0.69323698 1

chr1_94343234_94346061 701.9275537 591.3866536 812.4684537 1.373836303 0.458210112 0.590581241 1

chr1_94373744_94375754 557.9582619 494.3231927 621.5933311 1.257463417 0.330516429 0.724020911 1

chr1_94509703_94512687 141.4709263 97.06346092 185.8783916 1.915019204 0.937358859 0.607082762 1

chr1_94526548_94531349 367.0229693 402.263003 331.7829356 0.824791077 -0.277899369 0.80565419 1

chr1_94549116_94550189 51.00655113 61.03990841 40.97319385 0.671252545 -0.575072442 0.868208783 1

chr1_94606536_94610657 379.876471 191.1249591 568.627983 2.975163399 1.572968905 0.161979163 1

chr1_94701718_94703959 482.0471927 554.3624469 409.7319385 0.739104788 -0.436149176 0.661248656 1

chr1_94712294_94714016 290.5226259 325.2126268 255.832625 0.786662645 -0.346183017 0.783493531 1

chr1_94717599_94719611 121.4892346 105.0686948 137.9097744 1.312567694 0.39239183 0.844948248 1

chr1_94837404_94839542 121.9830215 96.06280668 147.9032363 1.539651416 0.622603755 0.753631517 1

chr1_94882768_94885227 705.4664687 654.4278705 756.505067 1.155979293 0.209115555 0.806364144 1

chr1_94891529_94892674 58.50622572 68.04448806 48.96796338 0.71964629 -0.474640107 0.881193949 1

chr1_94910432_94912418 68.03860186 127.083088 8.994115723 0.070773506 -3.820646795 0.213674589 1

chr1_95006486_95008287 258.9778186 225.1472032 292.8084341 1.300519971 0.379088554 0.775986932 1

chr1_95027762_95029305 89.03533624 143.0935558 34.9771167 0.244435303 -2.032475429 0.402733589 1

chr1_95044472_95046661 190.0163912 215.1406608 164.8921216 0.766438668 -0.383757745 0.806055335 1

chr1_95085431_95087461 109.0183359 137.0896304 80.94704151 0.590468012 -0.76006919 0.719123805 1

chr1_95118662_95121272 118.4669972 68.04448806 168.8895064 2.482045367 1.311529485 0.516630824 1

chr1_95284934_95287256 472.4376419 377.2466471 567.6286368 1.504661847 0.589439297 0.557100589 1

chr1_95319509_95321286 94.00786837 106.069349 81.9463877 0.772573684 -0.372255559 0.874026603 1

chr1_95347652_95352605 598.6018285 754.4932941 442.7103628 0.586765139 -0.769144934 0.397801427 1

chr1_95390842_95393078 236.4846811 213.1393523 259.8300098 1.219061647 0.285771083 0.838411836 1

chr1_95458943_95461589 238.0791876 359.2348708 116.9235044 0.325479272 -1.619362424 0.249425196 1

chr1_95467284_95469517 154.0379662 212.1386981 95.93723438 0.452238254 -1.144845063 0.511510931 1

chr1_95471800_95474300 213.5023347 217.1419693 209.8627002 0.966476913 -0.049192824 0.976518559 1

chr1_95477977_95481171 177.4970949 173.1131829 181.8810069 1.050647928 0.071279303 0.96820686 1

chr1_95536893_95539770 922.9419894 834.545633 1011.338346 1.211843075 0.277202892 0.718400249 1

chr1_95560401_95562738 139.942476 52.03402028 227.8509317 4.378883861 2.130563186 0.257882361 1

chr1_95581831_95584018 501.3578574 284.1858031 718.5299117 2.528380742 1.338213731 0.176285943 1

chr1_96394837_96395903 40.51210808 59.03859994 21.98561621 0.372393929 -1.42509854 0.722341254 1

chr1_96503027_96504422 98.53828138 157.1027151 39.97384766 0.254444028 -1.974579764 0.387311058 1

chr1_96539374_96541426 156.5579145 245.1602879 67.95554102 0.277188209 -1.851062207 0.292034614 1

chr1_96627772_96630252 121.5539828 204.1334642 38.97450147 0.190926567 -2.388910233 0.244760204 1

chr1_96630386_96631579 64.52453963 102.0667321 26.98234717 0.264359862 -1.919424949 0.515716657 1

chr1_96698778_96701996 165.0569352 252.1648675 77.94900294 0.309119203 -1.693764814 0.319192649 1

chr1_97186281_97189236 905.013928 926.6058228 883.4220333 0.953395728 -0.068852933 0.930323167 1

chr1_97330561_97331489 49.50426173 56.03663723 42.97188623 0.766853408 -0.382977277 0.91598529 1

chr1_97527041_97530730 461.4899614 446.2917894 476.6881333 1.068108678 0.095058447 0.927136913 1

chr1_97638955_97641238 97.03926209 157.1027151 36.97580909 0.235360726 -2.087054493 0.366780254 1

chr1_98519353_98520014 26.50818095 39.02551521 13.99084668 0.358505111 -1.479934406 0.788688227 1

chr1_99126408_99128361 265.0563026 351.2296369 178.8829683 0.509304881 -0.973398553 0.459451576 1

chr1_99729568_99730648 54.49118235 41.02682369 67.95554102 1.656368564 0.728023727 0.824425391 1

chr1_99921983_99923406 93.50557919 102.0667321 84.94442628 0.832244009 -0.264921515 0.911957186 1

chr1_100099358_100101283 105.0137569 126.0824338 83.94508009 0.665795207 -0.58684961 0.786549348 1

chr1_100108570_100113802 707.4409623 617.4036638 797.4782608 1.291664283 0.369231148 0.663768271 1

chr1_100125247_100126726 85.03598941 140.0915931 29.98038574 0.214005602 -2.224279531 0.375834329 1

chr1_100230527_100233766 326.4448049 242.1583252 410.7312847 1.696127046 0.762244237 0.521137938 1

chr1_100314573_100316741 778.5188061 807.5279687 749.5096436 0.92815317 -0.107565187 0.896571133 1

chr1_100408499_100409922 72.98758919 54.03532876 91.93984962 1.70147664 0.766787344 0.776462937 1

chr1_100451355_100452904 83.97516511 46.03009487 121.9202354 2.648707019 1.405288272 0.570801164 1

chr1_100502367_100506488 1286.97019 1241.811907 1332.128473 1.072729667 0.101286555 0.883358363 1

chr1_100623969_100624681 24.51145063 42.02747792 6.99542334 0.166448802 -2.58684961 0.665326421 1

chr1_100714090_100716362 440.4801465 410.2682369 470.6920562 1.147278814 0.198216041 0.849338227 1

chr1_100730599_100733173 738.4344287 638.4174027 838.4514547 1.313328006 0.393227277 0.637736728 1

chr1_100786980_100789908 231.4016191 81.05299314 381.7502452 4.709884612 2.235691716 0.124218027 1

chr1_100816112_100819605 858.8752654 668.4370298 1049.313501 1.569801573 0.650582211 0.409840579 1

chr1_100879542_100882996 172.9575255 108.0706575 237.8443936 2.200823045 1.138043152 0.487453077 1

chr1_100912831_100918525 366.9667234 316.2067387 417.726708 1.32105568 0.401691274 0.721192182 1

chr1_100980510_100981705 54.99216349 43.02813216 66.95619483 1.556102751 0.637937326 0.845518925 1

chr1_101004391_101006099 211.5173768 238.1557082 184.8790454 0.776294832 -0.365323412 0.804808355 1

chr1_101347358_101348804 60.98431651 37.02420674 84.94442628 2.294294294 1.198050461 0.692201963 1

chr1_101359319_101363372 1510.396661 1352.884527 1667.908794 1.232853773 0.302001694 0.643531403 1

chr1_101414405_101415998 106.9758241 70.04579654 143.9058516 2.054453782 1.038754875 0.626926187 1

chr1_101441501_101444414 190.4258092 77.05037619 303.8012422 3.942891096 1.979253863 0.213557549 1

chr1_101468184_101469424 74.00066985 75.04906772 72.95227198 0.972061002 -0.040881241 0.993416995 1

chr1_101490229_101492627 673.965481 621.4062807 726.5246812 1.169162115 0.225474986 0.795116562 1

chr1_101590062_101591533 65.01505642 88.05757279 41.97254004 0.476648841 -1.069001305 0.712576293 1

chr1_101722651_101726024 246.4774889 212.1386981 280.8162798 1.323739055 0.404618756 0.766860729 1

chr1_102246382_102247963 60.97319813 20.01308472 101.9333115 5.093333333 2.348610138 0.448753304 1

chr1_102262125_102263736 78.98105025 50.03271181 107.9293887 2.157176471 1.109144203 0.665489796 1

chr1_102554367_102556345 64.96338865 9.005888126 120.9208892 13.42687001 3.747051126 0.234458416 1

chr1_102699812_102701258 151.518018 179.1171083 123.9189277 0.691831891 -0.531506576 0.762513604 1

chr1_103255237_103256413 45.51210915 64.04187112 26.98234717 0.421323529 -1.246999607 0.735450366 1

chr1_103288137_103293320 335.1544209 571.3735689 98.93527296 0.173153394 -2.529877425 0.039841986 1

chr1_104067415_104069687 508.4716588 465.3042199 551.6390977 1.185545014 0.245550441 0.801419492 1

chr1_104599194_104601112 178.9451004 95.06215244 262.8280484 2.764802202 1.467176271 0.36516951 1

chr1_105614175_105616255 143.4598083 82.05364737 204.8659693 2.496732026 1.320040985 0.467780135 1

chr1_105630112_105631372 62.48660591 42.02747792 82.94573389 1.973607221 0.980834899 0.741934826 1

chr1_105680002_105682100 179.9038972 33.0215898 326.7862046 9.896137849 3.306865597 0.055630819 1

chr1_106565164_106566577 55.47417799 16.01046778 94.93788819 5.929738562 2.567968499 0.43934338 1

chr1_107097009_107099855 328.8607636 116.0758914 541.6456358 4.666306063 2.222280937 0.070166406 1

chr1_107201004_107204536 338.3460501 103.0673863 573.6247139 5.565530808 2.47651929 0.042851648 1

chr1_107260151_107262031 68.97385386 29.01897285 108.9287349 3.75370746 1.90831622 0.500135965 1

chr1_107421574_107423972 258.3610755 46.03009487 470.6920562 10.22574595 3.354134184 0.020169353 1

chr1_107598240_107601144 1048.92632 936.6123651 1161.240275 1.239830177 0.310142524 0.672757379 1

chr1_107683172_107685186 248.4290917 140.0915931 356.7665904 2.546666667 1.348610138 0.32406202 1

chr1_107703554_107707462 191.9215584 72.04710501 311.7960117 4.327668845 2.113590108 0.184273982 1

chr1_108076396_108080739 335.4369586 239.1563625 431.7175547 1.805168595 0.852133585 0.467933802 1

chr1_108111605_108117978 605.9366894 509.3330063 702.5403726 1.379334078 0.463971923 0.607598733 1

chr1_108243540_108246926 232.0320967 281.1838404 182.880353 0.65039425 -0.620613591 0.658191864 1

chr1_108251671_108252940 78.49118749 65.04252536 91.93984962 1.413534439 0.499307033 0.847963674 1

chr1_108268634_108272239 238.9464213 157.1027151 320.7901275 2.041913326 1.029921629 0.457447238 1

chr1_108308705_108310616 85.96862532 38.02486098 133.9123897 3.521706226 1.816274567 0.461352897 1

chr1_108506799_108509014 346.9176674 221.1445862 472.6907486 2.137473753 1.095906704 0.344724272 1

chr1_108660353_108661425 51.01505342 74.04841348 27.98169336 0.377883766 -1.403985553 0.680915072 1

chr1_108740953_108744018 1270.951874 1197.783121 1344.120628 1.122173626 0.166295911 0.809627317 1

chr1_108923139_108924514 46.52061165 78.05103043 14.99019287 0.19205631 -2.380398733 0.52141788 1

chr1_109062291_109063790 59.48333515 34.02224403 84.94442628 2.496732026 1.320040985 0.668120077 1

chr1_109074093_109078176 577.4370104 481.3146876 673.5593331 1.399415705 0.484824588 0.598862059 1

chr1_109101819_109104562 340.4781631 307.2008505 373.7554756 1.21664857 0.282912505 0.808929566 1

chr1_109126052_109128733 112.4460672 30.01962709 194.8725073 6.491503268 2.698552609 0.213894608 1

chr1_109202634_109204951 806.9622393 749.490023 864.4344556 1.153363526 0.205847305 0.799300827 1

chr1_109223504_109225606 100.9804009 71.04645077 130.9143511 1.842658566 0.881788772 0.689843069 1

chr1_109233772_109236852 1263.001578 1265.827609 1260.175547 0.995534889 -0.006456219 0.993826174 1

chr1_109288635_109290545 575.9634979 520.3402028 631.586793 1.213795877 0.279525826 0.762424597 1

chr1_109356309_109361123 492.9660963 441.2885182 544.6436744 1.234212203 0.303590465 0.758545294 1

chr1_109370072_109374067 328.0537 410.2682369 245.8391631 0.599215686 -0.738852704 0.532989153 1

chr1_109418651_109420819 345.4853584 323.2113183 367.7593985 1.13782958 0.186284492 0.873543447 1

chr1_109496233_109497473 55.4892205 39.02551521 71.95292579 1.843740573 0.882635673 0.78450908 1

chr1_109504172_109507114 715.4285376 606.3964672 824.460608 1.359606549 0.443189216 0.600099799 1

chr1_109583341_109585113 439.4579096 375.2453386 503.6704805 1.342243137 0.424646029 0.6821131 1

chr1_109617553_109620039 511.4356882 413.2701996 609.6011768 1.475066863 0.560780351 0.563203445 1

chr1_109631621_109636171 1094.877278 907.5933923 1282.161164 1.412704383 0.498459605 0.490846633 1

chr1_109676367_109683018 1296.691579 825.5397449 1767.843413 2.141439493 1.098580914 0.11132444 1

chr1_109781279_109785275 466.5619049 561.3670265 371.7567832 0.662234805 -0.594585259 0.555828141 1

chr1_109790873_109793634 613.9576199 549.3591757 678.556064 1.235177447 0.304718316 0.734916165 1

chr1_109823966_109827237 666.4533799 595.3892706 737.5174893 1.23871478 0.308844038 0.722708314 1

chr1_109840909_109843158 145.5297891 191.1249591 99.93461915 0.522875817 -0.935459748 0.602185509 1

chr1_109939000_109942472 673.8804581 491.32123 856.4396861 1.743135924 0.80168507 0.355468129 1

chr1_110008592_110010084 193.4715914 150.0981354 236.8450474 1.577934641 0.658037449 0.669159002 1

chr1_110025572_110027868 581.9994705 581.3801113 582.6188296 1.002130651 0.00307061 0.99948213 1

chr1_110035857_110037873 323.9745624 285.1864573 362.7626675 1.272019264 0.347120519 0.77147542 1

chr1_110074693_110075980 74.47941423 43.02813216 105.9306963 2.461893905 1.29976859 0.625791757 1

chr1_110088811_110092367 764.4592871 702.4592738 826.4593004 1.176522727 0.234529189 0.776424329 1

chr1_110185680_110186929 65.48006633 35.02289827 95.93723438 2.739271709 1.453792374 0.615869499 1

chr1_110281873_110283834 308.063506 405.2649657 210.8620464 0.520306625 -0.942566019 0.441019574 1

chr1_110305659_110306916 63.52126931 96.06280668 30.97973194 0.322494553 -1.6326533 0.581720774 1

chr1_110315008_110317447 179.5232562 215.1406608 143.9058516 0.668891929 -0.580154958 0.717410824 1

chr1_110324934_110327037 224.4611337 165.107949 283.8143184 1.718962171 0.781537796 0.583849051 1

chr1_110330285_110336779 484.8568728 266.1740268 703.5397188 2.643156912 1.402262074 0.163111521 1

chr1_110397723_110398900 54.99150947 42.02747792 67.95554102 1.616931217 0.693258309 0.831873264 1

chr1_110452773_110454544 302.507586 314.2054302 290.8097417 0.925540152 -0.111632516 0.930021074 1

chr1_110500543_110505403 693.9609071 634.4147858 753.5070284 1.187719841 0.248194574 0.772256149 1

chr1_110544803_110549320 481.8980756 326.213281 637.5828702 1.954496973 0.96679735 0.333224078 1

chr1_110576082_110578735 766.9681169 718.4697416 815.4664923 1.135004643 0.182698199 0.825057453 1

chr1_110630528_110632861 135.9620958 78.05103043 193.8731612 2.483928272 1.312623514 0.483228395 1

chr1_110647740_110649986 184.9915373 172.1125286 197.8705459 1.149658003 0.201204756 0.900800122 1

chr1_110692434_110694141 224.4317027 120.0785083 328.784897 2.738082789 1.453166069 0.313565324 1

chr1_110744945_110747315 167.9581785 104.0680406 231.8483164 2.227853193 1.155654167 0.487658816 1

chr1_110752010_110754390 470.8817227 290.1897285 651.5737169 2.245336939 1.166931955 0.249249788 1

chr1_110777025_110779589 482.8588345 267.1746811 698.5429879 2.614555335 1.386565604 0.168463341 1

chr1_110782154_110784029 312.8888831 143.0935558 482.6842105 3.373207185 1.754120933 0.156076637 1

chr1_110831646_110837628 634.245394 245.1602879 1023.3305 4.174128318 2.061474951 0.024630856 1

chr1_110854457_110856114 96.96274147 40.02616945 153.8993135 3.84496732 1.942971336 0.398980197 1

chr1_110877434_110885315 2021.35622 1802.178279 2240.534161 1.243236691 0.314100987 0.601450021 1

chr1_110896890_110901332 314.9418594 226.1478574 403.7358614 1.785273874 0.836145411 0.489232985 1

chr1_110923281_110928037 249.9032582 102.0667321 397.7397842 3.896860182 1.962312169 0.157109631 1

chr1_110948728_110951504 831.446221 749.490023 913.402419 1.21869857 0.285341337 0.720966002 1

chr1_110975171_110978440 185.9261353 73.04775925 298.8045113 4.090536306 2.032290006 0.207921543 1

chr1_111005321_111010044 370.3192421 94.06149821 646.5769859 6.873981366 2.781145941 0.019235252 1

chr1_111046409_111049914 173.9130522 41.02682369 306.7992808 7.478016898 2.902655731 0.092858777 1

chr1_111077846_111078620 49.49575944 43.02813216 55.96338672 1.300623195 0.379203058 0.91688719 1

chr1_111216773_111217967 127.5324014 177.1157998 77.94900294 0.440101916 -1.184090441 0.540793828 1

chr1_111218700_111220610 110.4689576 63.04121688 157.8966983 2.50465816 1.324613715 0.529197446 1

chr1_111505273_111508007 733.4625506 676.4422637 790.4828375 1.168588777 0.224767339 0.788918222 1

chr1_111652217_111653322 78.00655691 88.05757279 67.95554102 0.771717172 -0.373855887 0.88763109 1

chr1_111681482_111684042 885.9459056 803.5253517 968.3664596 1.205147364 0.269209568 0.730132774 1

chr1_111741933_111747980 1501.028444 1545.010141 1457.046747 0.943066138 -0.084569142 0.897835809 1

chr1_111888501_111889622 56.48922071 40.02616945 72.95227198 1.822614379 0.866009354 0.786022439 1

chr1_111963924_111967109 186.9267895 75.04906772 298.8045113 3.981455338 1.993295874 0.214918824 1

chr1_111990575_111993461 770.9589615 708.4631993 833.4547237 1.176426277 0.234410914 0.775820708 1

chr1_112117588_112121085 173.9516396 100.0654236 247.8378555 2.47675817 1.308453011 0.424650386 1

chr1_112134467_112137399 187.4330028 85.05561008 289.8103955 3.407304883 1.768631044 0.267203165 1

chr1_112161350_112163776 518.4775471 484.3166503 552.6384439 1.141068438 0.190385323 0.844565461 1

chr1_112190758_112192661 155.990223 141.0922473 170.8881987 1.211180642 0.276414053 0.874910952 1

chr1_112202008_112203977 101.9614345 43.02813216 160.8947368 3.739291686 1.902765014 0.394465292 1

chr1_112280282_112283263 590.8778242 404.2643114 777.491337 1.923225264 0.943527753 0.303333014 1

chr1_112296641_112299771 1010.501197 1012.662087 1008.340307 0.995732259 -0.006170225 0.99488488 1

chr1_112429068_112431232 114.4833469 89.05822703 139.9084668 1.570977455 0.651662476 0.751371897 1

chr1_112486580_112488116 56.47679429 21.01373896 91.93984962 4.375225646 2.129357424 0.510670786 1

chr1_112531429_112534461 498.8895769 330.215898 667.5632559 2.021596356 1.015494969 0.302637412 1

chr1_112936226_112940316 740.4043441 594.3886163 886.4200719 1.491314012 0.576584064 0.489576307 1

chr1_113002723_113009636 1018.688903 543.3552503 1494.022556 2.749623852 1.459234272 0.052924683 1

chr1_113044142_113045516 212.4709415 168.1099117 256.8319712 1.527762216 0.611420017 0.676898287 1

chr1_113050196_113052264 297.4310643 192.1256134 402.7365152 2.096214597 1.067786418 0.391581337 1

chr1_113071130_113073667 122.9496666 46.03009487 199.8692383 4.342142654 2.118407124 0.294742944 1

chr1_113085360_113087776 116.9431251 30.01962709 203.8666231 6.791111111 2.763647637 0.194287218 1

chr1_113135826_113138799 290.029493 335.2191691 244.8398169 0.730387279 -0.453266456 0.718563467 1

chr1_113160330_113163693 1566.965018 1513.989859 1619.940176 1.069980863 0.097584994 0.880500304 1

chr1_113209940_113212617 143.4271072 32.02093556 254.8332788 7.958333333 2.992466327 0.119267827 1

chr1_113216272_113219124 1276.39661 1118.731436 1434.061785 1.281864207 0.358243439 0.60232291 1

chr1_113240267_113244951 1379.235089 974.6372261 1783.832952 1.830253251 0.872043287 0.195698189 1

chr1_113254861_113259111 412.5108796 429.2806674 395.7410918 0.921870287 -0.117364327 0.914127717 1

chr1_113264777_113266703 76.98366591 52.03402028 101.9333115 1.958974359 0.970098514 0.709694753 1

chr1_113304555_113306407 109.4996964 109.0713118 109.9280811 1.00785513 0.011288279 1 1

chr1_113423724_113426110 144.4617706 86.05626432 202.8672769 2.357379541 1.237184053 0.493902177 1

chr1_113447187_113449079 96.49184536 84.05495584 108.9287349 1.295922814 0.373979792 0.871367543 1

chr1_113476918_113480399 292.4081724 152.0994439 432.7169009 2.84496044 1.508408592 0.234331686 1

chr1_113497788_113499653 288.0281846 331.2165522 244.8398169 0.739213712 -0.435936577 0.729841626 1

chr1_113562808_113565333 121.4565335 55.03598299 187.877084 3.413713607 1.771342029 0.379275614 1

chr1_113613774_113617314 790.410241 653.4272163 927.3932657 1.419275541 0.505154704 0.534648835 1

chr1_113643365_113648101 581.3977699 425.2780504 737.5174893 1.734200692 0.794270865 0.388398191 1

chr1_113683102_113684880 261.4532933 190.1243049 332.7822818 1.750340557 0.807635649 0.541376443 1

chr1_113719927_113721267 52.9784286 20.01308472 85.94377247 4.294379085 2.10244955 0.533224555 1

chr1_113747471_113753580 1317.815194 1035.677135 1599.953253 1.544837864 0.627455431 0.357192519 1

chr1_113758512_113763896 557.3395567 312.2041217 802.4749918 2.570353612 1.361966849 0.150385606 1

chr1_113779737_113783305 188.4199226 66.04317959 310.7966656 4.705961577 2.234489541 0.166192304 1

chr1_113788778_113791569 643.2970637 333.2178607 953.3762667 2.861119943 1.516579979 0.090807841 1

chr1_113794873_113799784 1610.703418 1157.756951 2063.649885 1.782455189 0.833865808 0.194668434 1

chr1_113808560_113810543 85.45783384 21.01373896 149.9019287 7.133520075 2.834614158 0.268242848 1

chr1_114117678_114120170 162.4101067 25.01635591 299.8038574 11.98431373 3.583075391 0.052331574 1

chr1_114300902_114303080 420.355878 200.1308472 640.5809087 3.200810458 1.678437247 0.119508084 1

chr1_114353406_114356140 702.9857619 681.4455349 724.5259888 1.063219218 0.088439087 0.918629933 1

chr1_114446299_114449048 569.9863874 549.3591757 590.6135992 1.075095539 0.104464872 0.911744013 1

chr1_114470824_114473825 874.9459033 792.5181551 957.3736515 1.208014788 0.272638116 0.7280256 1

chr1_114489301_114490434 56.99216392 45.02944063 68.95488721 1.531328976 0.614784251 0.847583936 1

chr1_114631428_114636677 464.4454885 381.249264 547.6417129 1.436440053 0.522497786 0.605398444 1

chr1_114780521_114781617 39.48071479 10.00654236 68.95488721 6.890980392 2.784709252 0.508946015 1

chr1_114888452_114889662 63.48791417 45.02944063 81.9463877 1.819840232 0.863811799 0.769751978 1

chr1_115051985_115055247 1278.429966 1171.766111 1385.093821 1.182056563 0.241299072 0.725692214 1

chr1_115123190_115125449 448.971319 405.2649657 492.6776724 1.21569273 0.281778629 0.784389202 1

chr1_115258249_115260236 433.9719699 391.2558064 476.6881333 1.218354145 0.284933549 0.785225894 1

chr1_115297562_115301861 1135.398542 980.6411515 1290.155933 1.315624917 0.395748237 0.5796474 1

chr1_115322280_115324251 523.4958608 517.3382401 529.6534815 1.023805009 0.03394097 0.973885463 1

chr1_115655997_115659845 213.4349704 114.0745829 312.7953579 2.742024997 1.455241723 0.325170097 1

chr1_115659971_115661907 145.4807375 116.0758914 174.8855835 1.506648636 0.591343007 0.74207583 1

chr1_115879782_115881356 106.9758241 70.04579654 143.9058516 2.054453782 1.038754875 0.626926187 1

chr1_116150778_116152008 48.98627601 28.01831861 69.9542334 2.496732026 1.320040985 0.706694145 1

chr1_116183540_116187348 1019.378243 833.5449788 1205.211507 1.44588659 0.531954397 0.473211944 1

chr1_116196900_116198688 149.9784493 117.0765456 182.880353 1.562057986 0.643448009 0.715530411 1

chr1_116236746_116238228 77.47287465 36.0235525 118.9221968 3.301234568 1.723005652 0.510903858 1

chr1_116294940_116299231 339.3839836 162.1059863 516.661981 3.187186315 1.672283358 0.159147188 1

chr1_116324580_116325935 65.97973942 35.02289827 96.93658058 2.767805789 1.468742716 0.610590861 1

chr1_116350992_116357461 643.8072011 349.2283285 938.3860738 2.687027361 1.426011012 0.110831981 1

chr1_116380731_116382784 172.9640658 118.0771999 227.8509317 1.929677634 0.948359855 0.561917253 1

chr1_116403825_116407013 149.9189333 26.01701014 273.8208565 10.52468577 3.395705255 0.075406674 1

chr1_116493678_116498589 434.8699426 236.1543998 633.5854854 2.682928991 1.423808871 0.177041293 1

chr1_116517976_116522216 824.2402024 427.2793589 1221.201046 2.858085748 1.515049201 0.062985994 1

chr1_116544122_116546912 189.4107665 53.03467452 325.7868584 6.142902947 2.61892059 0.108802598 1

chr1_116557237_116559658 137.457845 73.04775925 201.8679307 2.763506133 1.466499814 0.43208594 1

chr1_116608797_116611036 100.4467187 19.01243049 181.8810069 9.566425869 3.257980017 0.169384769 1

chr1_116666211_116667245 45.48529423 23.01504743 67.95554102 2.952657005 1.562013776 0.672989187 1

chr1_116680951_116682168 90.49511419 83.05430161 97.93592677 1.179179463 0.237783303 0.923151179 1

chr1_116693794_116696297 327.9327058 225.1472032 430.7182085 1.913051561 0.935875758 0.430711549 1

chr1_116736028_116739590 237.4670227 187.1223422 287.8117032 1.53809374 0.621143432 0.654162682 1

chr1_116759862_116765745 413.86863 213.1393523 614.5979078 2.883549664 1.52784587 0.157552085 1

chr1_116775500_116777309 66.47810447 33.0215898 99.93461915 3.02634185 1.597574961 0.57866729 1

chr1_116808301_116813748 360.3859502 186.1216879 534.6502125 2.872584159 1.522349161 0.185787738 1

chr1_116815395_116817430 124.9378946 30.01962709 219.8561621 7.323747277 2.872582008 0.163418729 1

chr1_116851300_116857768 1831.30582 1535.003598 2127.608042 1.386060622 0.470990357 0.445781289 1

chr1_116871149_116874603 553.401688 403.2636572 703.5397188 1.744614736 0.802908481 0.393024091 1

chr1_116959612_116962348 781.9471914 701.4586196 862.4357633 1.229489152 0.298059006 0.715438328 1

chr1_117007483_117011588 426.3957746 267.1746811 585.6168682 2.191887591 1.132173813 0.284151563 1

chr1_117025883_117028752 244.4290909 136.0889761 352.7692056 2.592195309 1.374174422 0.319131869 1

chr1_117122961_117125206 98.97059017 54.03532876 143.9058516 2.663180828 1.41315039 0.529504599 1

chr1_117129914_117133748 344.9843772 321.2100098 368.7587447 1.148030053 0.199160409 0.864786568 1

chr1_117200592_117202820 137.5232472 173.1131829 101.9333115 0.588824663 -0.764089995 0.679746274 1

chr1_117215604_117219359 501.9876809 483.3159961 520.6593658 1.077264916 0.107373074 0.914188621 1

chr1_117229346_117231249 345.854227 123.0804711 568.627983 4.61996918 2.207883227 0.065392886 1

chr1_117235520_117238117 430.8673256 228.1491659 633.5854854 2.77706685 1.473561906 0.164635197 1

chr1_117238884_117240112 157.9673327 108.0706575 207.8640078 1.923408376 0.943665106 0.582457225 1

chr1_117248918_117254866 1157.341647 915.5986262 1399.084668 1.528054573 0.611696069 0.389392639 1

chr1_117262239_117263862 69.97385408 30.01962709 109.9280811 3.661873638 1.872582008 0.503960069 1

chr1_117394397_117398943 473.9222727 355.2322539 592.6122916 1.668238976 0.738325971 0.46186829 1

chr1_117420502_117424115 332.5527199 413.2701996 251.8352403 0.609371884 -0.714605157 0.543883236 1

chr1_117441487_117445807 193.9444496 109.0713118 278.8175874 2.556287102 1.354049878 0.382300191 1

chr1_117451831_117454574 761.4520922 688.4501145 834.4540699 1.212076303 0.277480523 0.73700472 1

chr1_117481549_117483236 55.97581315 19.01243049 92.93919581 4.888338493 2.289344188 0.484050508 1

chr1_117593442_117595608 230.9640782 176.1151456 285.8130108 1.622875817 0.698552609 0.619290579 1

chr1_117601272_117603823 372.5219894 406.2656199 338.7783589 0.833883898 -0.262081564 0.815394272 1

chr1_117662854_117667031 789.4730269 748.4893687 830.4566851 1.109510328 0.149923097 0.854756775 1

chr1_117670105_117673526 203.9581862 140.0915931 267.8247793 1.91178338 0.934919064 0.533102532 1

chr1_117679398_117681347 102.0111402 119.0778541 84.94442628 0.713352007 -0.487313937 0.825767297 1

chr1_117687781_117693241 1485.72759 1069.699379 1901.755802 1.777841364 0.830126599 0.207391913 1

chr1_117704536_117707726 265.9948247 258.168793 273.8208565 1.060627248 0.084917718 0.951007964 1

chr1_117722660_117725690 193.5029845 198.1295388 188.8764302 0.953297683 -0.069001305 0.96760576 1

chr1_117729133_117733801 307.4166779 180.1177625 434.7155933 2.413507625 1.271131385 0.3020315 1

chr1_117739160_117741316 111.4683038 63.04121688 159.8953906 2.536362693 1.342761062 0.521546575 1

chr1_117773398_117776730 420.5076112 432.2826301 408.7325923 0.945521665 -0.080817579 0.941076214 1

chr1_117779512_117781715 204.9941576 196.1282303 213.860085 1.090409497 0.124870033 0.936192531 1

chr1_117807964_117815482 1790.002345 1794.173046 1785.831644 0.995350838 -0.006722963 0.992374877 1

chr1_117826514_117827755 132.9967583 128.0837422 137.9097744 1.076715686 0.106637347 0.958659743 1

chr1_117908147_117911657 1268.519565 1298.849199 1238.189931 0.953297683 -0.069001305 0.921176897 1

chr1_118080390_118084845 734.819647 459.3002944 1010.339 2.199735145 1.137329829 0.17751271 1

chr1_118103262_118109471 1701.224039 1279.836768 2122.611311 1.658501587 0.729880392 0.248196477 1

chr1_118129486_118136355 2064.71725 1633.067714 2496.366786 1.528636422 0.61224531 0.306783053 1

chr1_118146935_118150779 1186.919155 1063.695453 1310.142857 1.231689816 0.300638979 0.669434866 1

chr1_118155147_118157626 378.8712386 182.119071 575.6234063 3.160698125 1.660243252 0.141383808 1

chr1_118195269_118200277 1742.640006 1192.77985 2292.500163 1.921980962 0.942594046 0.134517723 1

chr1_118294551_118297854 247.9065278 105.0686948 390.7443609 3.718941799 1.89489217 0.172624006 1

chr1_118300391_118302627 200.9222143 82.05364737 319.7907813 3.897337797 1.962488981 0.204637018 1

chr1_118362957_118365270 239.0334063 290.1897285 187.877084 0.647428443 -0.627207348 0.649923737 1

chr1_118622564_118626417 178.9640671 124.0811253 233.8470088 1.884629981 0.9142813 0.569015803 1

chr1_118641143_118646426 484.891536 319.2087014 650.5743707 2.038084701 1.02721401 0.303089513 1

chr1_118652986_118654685 78.98105025 50.03271181 107.9293887 2.157176471 1.109144203 0.665489796 1

chr1_118658148_118662633 758.40631 615.4023553 901.4102647 1.464749455 0.550653913 0.505324286 1

chr1_119097267_119102403 366.9915762 354.2315996 379.7515528 1.07204313 0.100362949 0.930941458 1

chr1_119177323_119179961 284.9699759 239.1563625 330.7835894 1.383126863 0.467933489 0.712171417 1

chr1_119369436_119370509 43.48725587 24.01570167 62.95881006 2.621568627 1.390430313 0.715354108 1

chr1_119505503_119507486 159.9555607 92.06018973 227.8509317 2.475021313 1.307440949 0.445471367 1

chr1_119543750_119545147 68.510152 84.05495584 52.96534815 0.630127607 -0.666284078 0.813283388 1

chr1_119682162_119684433 507.4984735 505.3303893 509.6665577 1.008580858 0.01232675 0.992159838 1

chr1_119871258_119872981 114.516048 139.0909388 89.94115723 0.646635633 -0.628975086 0.759813164 1

chr1_119931337_119934605 250.0275224 292.191037 207.8640078 0.711397618 -0.49127195 0.716444265 1

chr1_120084303_120086398 94.52650812 135.0883219 53.96469434 0.399477124 -1.323815204 0.565891634 1

chr1_120172397_120175925 517.8948132 357.2335623 678.556064 1.899474561 0.925600391 0.339075205 1

chr1_120189366_120192253 744.9739984 705.4612366 784.4867603 1.112019654 0.153182287 0.85489505 1

chr1_120201714_120205637 548.4180375 423.2767419 673.5593331 1.591297764 0.670203819 0.476955642 1

chr1_120215727_120217598 108.5291272 153.1000981 63.95815626 0.417753855 -1.259274952 0.553359928 1

chr1_120217805_120219871 124.9895624 109.0713118 140.907813 1.29188703 0.369479918 0.851724421 1

chr1_120238787_120240584 176.9660287 125.0817795 228.8502779 1.829605229 0.871532394 0.589314116 1

chr1_120254007_120257756 1359.350847 1131.739941 1586.961752 1.402231815 0.487724874 0.469387835 1

chr1_120288778_120290507 202.9758446 166.1086032 239.843086 1.44389322 0.529964055 0.724500352 1

chr1_120308035_120312303 430.0027081 434.2839385 425.7214776 0.980283726 -0.028728722 0.98047194 1

chr1_120331987_120334900 505.4481133 426.2787046 584.617522 1.371444352 0.455696085 0.640243706 1

chr1_120402270_120405353 131.9555547 64.04187112 199.8692383 3.120915033 1.64196908 0.391734728 1

chr1_120408964_120414892 614.8137352 330.215898 899.4115723 2.723707665 1.445571867 0.112776828 1

chr1_120434797_120438159 352.4990944 351.2296369 353.7685518 1.007228646 0.01039122 0.995565391 1

chr1_120510253_120513068 523.9046248 378.2473013 669.5619483 1.770169796 0.823887752 0.391833252 1

chr1_120538695_120540953 124.482695 98.06411515 150.9012749 1.538802188 0.621807786 0.751154464 1

chr1_120610020_120613149 1544.540553 1607.050703 1482.030402 0.922205129 -0.116840405 0.85755004 1

chr1_120622410_120624821 137.9941433 129.0843965 146.9038901 1.138045296 0.18655798 0.92256681 1

chr1_120638965_120640899 91.98301509 66.04317959 117.9228506 1.785541691 0.83636182 0.720422736 1

chr1_120793918_120798878 364.3885671 194.1269218 534.6502125 2.75412708 1.46159513 0.200972251 1

chr1_120808545_120810342 155.9869529 136.0889761 175.8849297 1.29242599 0.370081668 0.831738216 1

chr1_120834124_120838681 867.9609443 808.5286229 927.3932657 1.147013525 0.197882403 0.801689478 1

chr1_120856268_120857332 38.98496583 16.01046778 61.95946387 3.869934641 1.952309201 0.638356383 1

chr1_120904320_120906932 226.5559674 312.2041217 140.907813 0.451332328 -1.147737976 0.420942173 1

chr1_120924825_120926532 260.4611414 201.1315015 319.7907813 1.589958703 0.668989294 0.613379892 1

chr1_121137381_121139440 505.0857849 636.4160943 373.7554756 0.587281621 -0.767875605 0.431710333 1

chr1_121156285_121157686 95.50034744 96.06280668 94.93788819 0.98828976 -0.016994002 0.99932373 1

chr1_121260389_121262190 359.9974609 356.2329081 363.7620137 1.021135346 0.0301741 0.981400592 1

chr1_121328041_121329550 113.4728823 72.04710501 154.8986597 2.149963689 1.104312294 0.593100433 1

chr1_121351479_121355448 363.4448129 279.1825319 447.7070938 1.603635767 0.681346501 0.54636694 1

chr1_121478224_121485629 2421.359576 2207.443245 2635.275907 1.193813663 0.255577671 0.656671031 1

chr1_142618433_142619684 105.4820369 78.05103043 132.9130435 1.702899279 0.767993107 0.721514059 1

chr1_142890172_142891496 103.9594729 42.02747792 165.8914678 3.947214441 1.980834899 0.371068766 1

chr1_143466482_143468591 582.9824661 556.3637554 609.6011768 1.095688155 0.13183725 0.887111581 1

chr1_143730297_143732224 87.48399517 63.04121688 111.9267734 1.775453885 0.828187889 0.731049523 1

chr1_143741945_143746242 1763.475197 1726.128558 1800.821837 1.043272142 0.06111554 0.922738055 1

chr1_143911851_143916638 854.0054148 862.5639516 845.446878 0.980155589 -0.028917316 0.972350884 1

chr1_143941624_143945216 245.9889342 229.1498201 262.8280484 1.146970346 0.197828092 0.886465744 1

chr1_143953286_143958020 350.8915074 185.1210337 516.661981 2.79094153 1.4807519 0.203134757 1

chr1_144026748_144031181 598.0759945 714.4671247 481.6848643 0.674187584 -0.568778037 0.531284049 1

chr1_144156296_144158903 226.9928543 216.141315 237.8443936 1.100411523 0.138043152 0.924982617 1

chr1_144310947_144312375 99.02094989 131.0857049 66.95619483 0.510781819 -0.969220921 0.664612616 1

chr1_144326750_144328310 79.99020677 65.04252536 94.93788819 1.459627954 0.545600686 0.83162863 1

chr1_144338306_144342151 1357.961703 1299.849853 1416.073553 1.089413174 0.123551218 0.855230682 1

chr1_144491154_144492540 100.515391 124.0811253 76.94965675 0.620156019 -0.689296879 0.756111636 1

chr1_144518072_144522595 1440.389451 1271.831534 1608.947368 1.265063277 0.339209548 0.608355201 1

chr1_144526132_144531864 433.3434544 194.1269218 672.5599869 3.46453743 1.792662743 0.093054747 1

chr1_144532861_144534596 140.0176885 167.1092575 112.9261196 0.675762201 -0.56541244 0.758174552 1

chr1_144567157_144568701 75.45979377 14.00915931 136.9104282 9.772922502 3.288790051 0.242347117 1

chr1_144572509_144573994 131.0019901 134.0876677 127.9163125 0.953975222 -0.0679763 0.97560874 1

chr1_144592961_144596430 815.0047525 822.5377822 807.4717227 0.981683444 -0.02667021 0.975146938 1

chr1_144706196_144710715 502.5763011 619.4049722 385.7476299 0.622771284 -0.683225671 0.48477936 1

chr1_144821150_144824130 303.9882926 286.1871116 321.7894737 1.124402395 0.169158432 0.892298221 1

chr1_144867603_144869583 72.47091151 28.01831861 116.9235044 4.173109244 2.061122688 0.454453945 1

chr1_144940069_144942263 128.5356717 183.1197252 73.95161817 0.403842994 -1.308133582 0.498205748 1

chr1_144972315_144973196 47.00785832 59.03859994 34.9771167 0.592444888 -0.755247142 0.835415212 1

chr1_145038631_145040803 272.9863239 252.1648675 293.8077803 1.165141612 0.220505312 0.866303215 1

chr1_145138778_145140613 627.026949 668.4370298 585.6168682 0.876098783 -0.190834548 0.831292853 1

chr1_145144220_145145689 70.49249382 59.03859994 81.9463877 1.388013737 0.473021846 0.865798752 1

chr1_145178958_145180996 109.9869431 90.05888126 129.9150049 1.442556282 0.528627607 0.802403665 1

chr1_145195308_145197334 143.0019927 146.0955185 139.9084668 0.95765064 -0.062428651 0.97649401 1

chr1_145244774_145249635 741.4998316 741.4847891 741.5148741 1.000040574 5.85E-05 1 1

chr1_145252949_145255904 253.9738934 214.1400066 293.8077803 1.372035917 0.456318249 0.733982163 1

chr1_145280581_145282285 103.496425 98.06411515 108.9287349 1.110790983 0.151587371 0.948391082 1

chr1_145381619_145383810 358.5540335 441.2885182 275.8195489 0.625032235 -0.677997498 0.550828502 1

chr1_145395123_145398228 799.499844 799.5227348 799.4769532 0.999942739 -8.26E-05 1 1

chr1_145469604_145471408 261.0301409 307.2008505 214.8594312 0.699410274 -0.515789105 0.696740114 1

chr1_145514668_145517636 738.1074175 902.5901211 573.6247139 0.635531788 -0.653963806 0.434014545 1

chr1_145541044_145545661 874.032234 923.6038601 824.460608 0.892656087 -0.163823638 0.835122464 1

chr1_145574257_145577060 559.0360908 614.4017011 503.6704805 0.819773903 -0.286702031 0.759413994 1

chr1_145588354_145589884 242.5121513 261.1707557 223.8535469 0.857115669 -0.222438184 0.872974758 1

chr1_145609088_145612168 855.7150293 1184.774616 526.6554429 0.44451952 -1.169681324 0.14215634 1

chr1_145652321_145653474 65.00524608 73.04775925 56.96273292 0.779801236 -0.358821654 0.904755958 1

chr1_145726184_145728045 62.02355807 98.06411515 25.98300098 0.264959317 -1.916157236 0.526949077 1

chr1_145825776_145828016 443.066805 545.3565588 340.7770513 0.62487018 -0.6783716 0.511362383 1

chr1_145968273_145970805 555.6031273 713.4664704 397.7397842 0.557475089 -0.843020755 0.369261212 1

chr1_146008127_146009075 33.5127606 53.03467452 13.99084668 0.263805648 -1.922452642 0.678275733 1

chr1_146009086_146009888 57.51276573 77.05037619 37.97515528 0.492861387 -1.020746137 0.745591351 1

chr1_146038847_146041817 233.9823914 207.1354269 260.829356 1.25922137 0.33253193 0.81292572 1

chr1_146076343_146077533 71.99805333 69.0451423 74.95096436 1.085535664 0.118407124 0.970238352 1

chr1_146081193_146083830 520.0890583 656.429179 383.7489375 0.58460067 -0.774476613 0.422024366 1

chr1_146232511_146235339 251.9863194 231.1511286 272.8215103 1.180273321 0.23912099 0.860596158 1

chr1_146417528_146420282 271.9935179 262.1714099 281.815626 1.074928903 0.104241241 0.93839169 1

chr1_146548140_146549310 45.01897627 74.04841348 15.98953906 0.215933581 -2.211340475 0.558385419 1

chr1_146555332_146557384 492.0962465 639.418057 344.7744361 0.539200344 -0.891106679 0.367604392 1

chr1_146574163_146576703 162.0366599 218.1426235 105.9306963 0.485602926 -1.04215098 0.538589154 1

chr1_146584510_146587622 240.5069187 251.1642133 229.849624 0.915136838 -0.127940613 0.928506814 1

chr1_146642495_146645716 1061.090482 1199.784429 922.3965348 0.768801888 -0.379316215 0.603899387 1

chr1_146696502_146697516 49.51407207 71.04645077 27.98169336 0.393850686 -1.344279307 0.699509013 1

chr1_146707348_146713320 888.6208572 1073.701995 703.5397188 0.655246727 -0.609889852 0.433855955 1

chr1_146713367_146715794 639.0563826 725.4743213 552.6384439 0.761761551 -0.392588624 0.657115645 1

chr1_146975739_146978302 167.4840123 143.0935558 191.8744688 1.340902235 0.423204054 0.799993601 1

chr1_146987185_146989260 169.4944771 161.105332 177.8836221 1.104144846 0.142929443 0.934008022 1

chr1_146993991_146995814 76.98562798 55.03598299 98.93527296 1.797647059 0.846109797 0.745593669 1

chr1_147070120_147073224 808.0708072 916.5992804 699.542334 0.763193196 -0.389879786 0.628914607 1

chr1_147082615_147086017 411.5815138 536.3506706 286.812357 0.534747829 -0.903069373 0.398587674 1

chr1_147140730_147143705 941.1754793 1209.790972 672.5599869 0.555930737 -0.847022944 0.268430026 1

chr1_147147960_147149426 49.52257436 84.05495584 14.99019287 0.178338002 -2.487313937 0.486060977 1

chr1_147228422_147231357 144.5389452 204.1334642 84.94442628 0.416122004 -1.264921515 0.484343651 1

chr1_147399519_147401721 409.0517552 488.3192673 329.7842432 0.675345548 -0.566302233 0.596615605 1

chr1_147509956_147512351 539.0844842 668.4370298 409.7319385 0.612970138 -0.706111303 0.457049709 1

chr1_147550593_147551832 71.01636574 96.06280668 45.96992481 0.478540305 -1.063287654 0.698168572 1

chr1_147580694_147583497 217.4951413 210.1373896 224.8528931 1.070028011 0.097648564 0.949199323 1

chr1_147623017_147625537 557.5861232 689.4507688 425.7214776 0.617479154 -0.695537662 0.4574358 1

chr1_147734694_147737043 621.6378046 832.5443246 410.7312847 0.493344646 -1.019332244 0.256879673 1

chr1_147805591_147808048 797.1499416 1026.671246 567.6286368 0.55288257 -0.854955006 0.293341624 1

chr1_147843820_147844984 56.50360921 62.04056265 50.96665577 0.821505376 -0.283658078 0.933075049 1

chr1_147860351_147861481 38.9843118 15.00981354 62.95881006 4.194509804 2.068502218 0.61941504 1

chr1_147874097_147875284 138.0294605 183.1197252 92.93919581 0.507532412 -0.978428137 0.596553976 1

chr1_147917831_147919434 86.98563011 65.04252536 108.9287349 1.674731021 0.743929402 0.758562057 1

chr1_147992773_147995435 425.5259249 465.3042199 385.7476299 0.829022419 -0.270516978 0.797718443 1

chr1_147999578_148001437 336.0425833 401.2623487 270.8228179 0.674927061 -0.567196495 0.628190949 1

chr1_148015466_148018425 332.9601759 272.1779523 393.7423994 1.446635909 0.532701869 0.650689428 1

chr1_148246815_148248583 330.0295016 375.2453386 284.8136646 0.759006536 -0.397815786 0.736694884 1

chr1_148328289_148331109 223.4971047 219.1432777 227.8509317 1.039734981 0.056215845 0.971829747 1

chr1_148526513_148528036 53.97188859 11.0071966 96.93658058 8.806654783 3.138594114 0.363655151 1

chr1_148531780_148533360 157.5173653 184.1203795 130.9143511 0.71102586 -0.492026064 0.775265825 1

chr1_148555151_148557962 719.5874659 853.5580635 585.6168682 0.686089082 -0.543532186 0.51937617 1

chr1_148587290_148589956 228.9823904 202.1321557 255.832625 1.265670096 0.339901408 0.810863632 1

chr1_148597904_148599681 256.5173865 283.1851489 229.849624 0.811658468 -0.301055301 0.822640374 1

chr1_148603804_148606688 254.0334095 305.1995421 202.8672769 0.664703739 -0.589216625 0.660313831 1

chr1_148759924_148761798 232.5173813 259.1694472 205.8653154 0.7943271 -0.33219487 0.813705158 1

chr1_148853842_148855181 86.52323629 122.0798168 50.96665577 0.417486339 -1.260199105 0.603939713 1

chr1_148927396_148929545 461.5363969 517.3382401 405.7345537 0.784273271 -0.350571663 0.729936472 1

chr1_149151081_149151530 31.51210615 50.03271181 12.99150049 0.259660131 -1.945303581 0.688613838 1

chr1_149205186_149206394 52.51341869 73.04775925 31.97907813 0.43778315 -1.191711668 0.721528926 1

chr1_149215313_149216480 70.01309542 90.05888126 49.96730957 0.554829339 -0.849884016 0.758972657 1

chr1_149378791_149380456 210.9882727 193.1262676 228.8502779 1.18497748 0.244859641 0.86973108 1

chr1_149398260_149399326 101.5186613 130.0850507 72.95227198 0.560804424 -0.834430364 0.704854533 1

chr1_149399358_149400719 130.5448284 199.130193 61.95946387 0.311150524 -1.68431542 0.382982961 1

chr1_149512874_149515505 280.5383203 339.2217861 221.8548545 0.654011221 -0.612612706 0.631355112 1

chr1_149574667_149579370 1301.44763 1221.798822 1381.096437 1.130379577 0.176807306 0.796250189 1

chr1_149604913_149606421 167.0320828 216.141315 117.9228506 0.545582183 -0.874131562 0.59955074 1

chr1_149763619_149765444 305.489274 289.1890743 321.7894737 1.112730398 0.154104086 0.901860971 1

chr1_149782744_149785703 578.1557809 816.5338568 339.7777051 0.416122004 -1.264921515 0.174084227 1

chr1_149801884_149806381 1016.653586 1251.81845 781.4887217 0.624282796 -0.679728386 0.360429763 1

chr1_149820085_149826617 2529.471437 3251.125614 1807.81726 0.556058878 -0.846690445 0.13874447 1

chr1_149855910_149861702 2225.324217 2721.779523 1728.868911 0.635198001 -0.654721723 0.265607637 1

chr1_149870613_149872372 446.0439149 513.3356232 378.7522066 0.737825683 -0.438648087 0.670149359 1

chr1_149898610_149901038 727.5966239 875.5724567 579.6207911 0.661990663 -0.595117226 0.478753813 1

chr1_149907557_149912817 802.4965745 797.5214263 807.4717227 1.012476526 0.017888459 0.984030501 1

chr1_149980642_149983545 672.0583517 761.4978738 582.6188296 0.765095806 -0.38628768 0.655820261 1

chr1_149993227_149994740 100.4898841 85.05561008 115.9241582 1.362921953 0.446702949 0.84188048 1

chr1_150038250_150040941 825.5685219 930.6084397 720.5286041 0.774255394 -0.369118566 0.644665279 1

chr1_150121223_150123482 310.9451286 227.1485116 394.7417456 1.737813481 0.797273246 0.512059147 1

chr1_150253724_150254714 74.52127166 107.0700033 41.97254004 0.392010262 -1.351036673 0.612330888 1

chr1_150264951_150267866 1024.60192 1180.771999 868.4318404 0.73547801 -0.443245887 0.549222913 1

chr1_150292698_150296247 1224.01465 1246.815178 1201.214122 0.96342597 -0.053754282 0.939588719 1

chr1_150359204_150360845 115.9719018 73.04775925 158.8960444 2.175235026 1.121171287 0.582853447 1

chr1_150361149_150362786 119.4879261 101.0660779 137.9097744 1.364550573 0.448425864 0.824244947 1

chr1_150458726_150461130 944.0557938 1029.673209 858.4383785 0.833699829 -0.262400055 0.730793449 1

chr1_150476223_150482837 859.622159 1046.684331 672.5599869 0.642562391 -0.638091551 0.418687009 1

chr1_150504925_150506063 87.99282457 77.05037619 98.93527296 1.284033613 0.36068297 0.883028585 1

chr1_150520579_150525700 896.565921 997.6522735 795.4795684 0.797351532 -0.326712183 0.673925865 1

chr1_150575419_150579812 867.8412582 625.4088977 1110.273619 1.77527634 0.828043612 0.293334778 1

chr1_150594700_150599337 540.9994617 540.3532876 541.6456358 1.002391673 0.003446335 0.999337985 1

chr1_150620795_150624921 513.4847403 490.3205758 536.6489048 1.094485794 0.130253229 0.89445206 1

chr1_150668575_150670814 184.5284894 228.1491659 140.907813 0.617612659 -0.695225771 0.659628838 1

chr1_150847876_150850336 365.0314711 413.2701996 316.7927427 0.76655114 -0.383546051 0.734005852 1

chr1_151008730_151010151 86.50426965 93.06084397 79.94769532 0.85909059 -0.219117826 0.931682755 1

chr1_151019536_151021467 255.0569545 342.2237488 167.8901602 0.490585942 -1.027422202 0.443892622 1

chr1_151118219_151119833 143.0091869 157.1027151 128.9156587 0.820581991 -0.285280603 0.876999096 1

chr1_151137237_151139894 586.0531011 667.4363756 504.6698267 0.756131738 -0.403290483 0.659836987 1

chr1_151161045_151163460 504.5416383 568.3716062 440.7116704 0.775393538 -0.366999383 0.707057909 1

chr1_151167664_151172878 1099.573813 1212.792934 986.354691 0.813291917 -0.298154819 0.679868214 1

chr1_151226137_151228821 499.0465423 570.3729147 427.72017 0.749895654 -0.415238232 0.671984489 1

chr1_151281273_151285957 391.3885729 221.1445862 561.6325596 2.53966226 1.344636651 0.222947239 1

chr1_151298665_151301403 598.0459095 668.4370298 527.6547891 0.789385934 -0.341197283 0.707459512 1

chr1_151318292_151321016 448.5416263 512.334969 384.7482837 0.75097018 -0.413172474 0.687606663 1

chr1_151339824_151347441 1181.851136 954.6241414 1409.07813 1.476055412 0.561746882 0.425875946 1

chr1_151429528_151433093 642.6672401 898.5875042 386.7469761 0.43039434 -1.216268988 0.172013403 1

chr1_151508984_151516087 1166.60522 1327.868172 1005.342269 0.757109998 -0.401425175 0.570740169 1

chr1_151541145_151543828 134.4892374 118.0771999 150.9012749 1.277988257 0.35387458 0.851993343 1

chr1_151577946_151580274 168.00069 169.1105659 166.890814 0.986873961 -0.019062253 0.994873842 1

chr1_151678199_151679405 75.51342361 96.06280668 54.96404053 0.572167756 -0.805489897 0.760374426 1

chr1_151689212_151694048 458.0995094 610.3990841 305.7999346 0.500983607 -0.997164699 0.329125067 1

chr1_151694797_151699080 470.62927 668.4370298 272.8215103 0.408148409 -1.292834261 0.203107171 1

chr1_151703457_151708145 524.5736897 637.4167485 411.7306309 0.645936323 -0.630536145 0.511266633 1

chr1_151710953_151714565 299.5239359 336.2198234 262.8280484 0.781714908 -0.355285543 0.774640836 1

chr1_151734820_151737217 672.0629299 768.5024534 575.6234063 0.749019608 -0.416924609 0.630433351 1

chr1_151754454_151755718 76.99151418 64.04187112 89.94115723 1.404411765 0.489965987 0.85268867 1

chr1_151762136_151764583 391.5455383 461.3016029 321.7894737 0.697568514 -0.519593172 0.634024214 1

chr1_151811482_151813882 558.4553189 490.3205758 626.5900621 1.277919168 0.353796584 0.705255619 1

chr1_151881219_151882863 180.0020006 183.1197252 176.8842759 0.965948784 -0.049981398 0.978714026 1

chr1_151918351_151919821 92.51735138 119.0778541 65.95684864 0.553896853 -0.852310753 0.714338811 1

chr1_152000819_152002919 169.4663541 118.0771999 220.8555083 1.870433145 0.9033724 0.584576281 1

chr1_152017661_152024779 1619.566076 1721.125286 1518.006865 0.881985104 -0.181173805 0.77717028 1

chr1_152060409_152064198 235.9922022 224.1465489 247.8378555 1.105695612 0.144954279 0.919389922 1

chr1_152081296_152085571 788.6705415 1049.686294 527.6547891 0.502678555 -0.992291953 0.225497472 1

chr1_152626420_152629407 116.5703323 224.1465489 8.994115723 0.04012605 -4.63931703 0.046783443 1

chr1_152699660_152701599 76.54547092 146.0955185 6.99542334 0.047882532 -4.384356746 0.135951725 1

chr1_153093725_153097712 860.0819367 985.6444227 734.5194507 0.745217478 -0.424266584 0.590348139 1

chr1_153233511_153234696 56.53173217 105.0686948 7.994769532 0.076090881 -3.716132627 0.279820136 1

chr1_153264439_153265939 43.52191905 77.05037619 9.993461915 0.129700365 -2.946745555 0.457079933 1

chr1_153345726_153349964 434.8869472 262.1714099 607.6024844 2.317577209 1.212617402 0.247963212 1

chr1_153361877_153365765 307.9523222 235.1537455 380.750899 1.61915728 0.695243131 0.56903021 1

chr1_153410913_153414576 184.9287511 76.04972195 293.8077803 3.863364293 1.949857722 0.22702199 1

chr1_153450400_153452494 117.9771345 83.05430161 152.8999673 1.840963855 0.880461302 0.662659194 1

chr1_153460970_153464945 257.409473 119.0778541 395.7410918 3.323381117 1.732651747 0.201759714 1

chr1_153492693_153495153 312.9824083 286.1871116 339.7777051 1.187257187 0.24763249 0.839274952 1

chr1_153507067_153514353 1005.837364 757.4952568 1254.17947 1.655692836 0.727435049 0.329775657 1

chr1_153535833_153542050 1669.067721 1773.159307 1564.976136 0.882591953 -0.180181501 0.776467675 1

chr1_153557803_153561356 346.5115195 364.238142 328.784897 0.902664656 -0.147737976 0.899992693 1

chr1_153621257_153622932 103.9836717 79.05168466 128.9156587 1.630776868 0.705559398 0.745576988 1

chr1_153640528_153645445 1116.556158 1202.786392 1030.325923 0.85661588 -0.223279673 0.75633012 1

chr1_153650412_153652235 179.5454929 249.1629048 109.9280811 0.441189595 -1.180529328 0.462870556 1

chr1_153660343_153662466 125.4951217 118.0771999 132.9130435 1.125645286 0.170752277 0.933490716 1

chr1_153698520_153702313 1475.6563 1715.121361 1236.191239 0.720760214 -0.472408719 0.472321092 1

chr1_153739693_153742079 114.0281473 157.1027151 70.9535796 0.45163815 -1.146760739 0.578082404 1

chr1_153754774_153757393 940.0695274 1046.684331 833.4547237 0.796280883 -0.328650672 0.666745349 1

chr1_153852358_153855453 331.4971278 327.2139353 335.7803203 1.026179769 0.037283488 0.977463629 1

chr1_153915987_153920170 1716.60076 1871.223422 1561.978097 0.83473629 -0.260607601 0.678562145 1

chr1_153929209_153932371 1088.591469 1228.803402 948.3795357 0.771791105 -0.373717678 0.606101164 1

chr1_153947668_153951437 1694.660271 1940.268564 1449.051978 0.746830622 -0.421147013 0.504537139 1

chr1_153957471_153960374 496.0197268 526.3441283 465.6953252 0.884773478 -0.176619954 0.85858075 1

chr1_153962039_153965603 1089.703307 1400.915931 778.4906832 0.555701214 -0.847618703 0.243824268 1

chr1_153982211_153983337 45.01832225 73.04775925 16.98888526 0.232572298 -2.104248827 0.576516292 1

chr1_154076833_154078770 112.9961 107.0700033 118.9221968 1.110695742 0.151463667 0.945344255 1

chr1_154129099_154131827 205.4173101 79.05168466 331.7829356 4.19703814 2.069371574 0.177668789 1

chr1_154165131_154168317 996.788964 1438.940792 554.6371363 0.385448199 -1.375391109 0.069602646 1

chr1_154190759_154195217 2324.321622 2816.841675 1831.801569 0.650303347 -0.620815246 0.285844874 1

chr1_154243626_154246660 1228.10883 1394.912005 1061.305655 0.760840577 -0.394333905 0.571086832 1

chr1_154300153_154302297 627.2225017 967.6326465 286.812357 0.296406243 -1.754352262 0.054451741 1

chr1_154323654_154327778 757.6254073 949.6208702 565.6299444 0.595637651 -0.747493144 0.366840018 1

chr1_154357083_154358351 73.01178802 91.0595355 54.96404053 0.603605545 -0.728322036 0.787525848 1

chr1_154376817_154381722 1103.563349 1200.785083 1006.341615 0.838069717 -0.254857832 0.724104203 1

chr1_154388848_154394629 795.0747286 909.5947008 680.5547564 0.748195604 -0.418512606 0.606125848 1

chr1_154404179_154407797 429.5278878 472.3087995 386.7469761 0.81884347 -0.288340403 0.783676463 1

chr1_154491760_154493021 63.00328359 68.04448806 57.96207911 0.851826221 -0.231368956 0.942024532 1

chr1_154528522_154533438 1474.696849 1776.161269 1173.232429 0.660543864 -0.598273726 0.36325491 1

chr1_154539966_154541068 48.01047462 64.04187112 31.97907813 0.499346405 -1.00188711 0.778356251 1

chr1_154579245_154582618 1484.61706 1664.087995 1305.146126 0.784301149 -0.350520381 0.593061817 1

chr1_154683827_154687483 446.4722995 404.2643114 488.6802876 1.208813823 0.273592063 0.791075969 1

chr1_154702357_154704404 143.0294616 188.1229964 97.93592677 0.520595188 -0.941766117 0.603218616 1

chr1_154739478_154741719 89.46829906 41.02682369 137.9097744 3.36145385 1.749085343 0.466753163 1

chr1_154841708_154843185 71.9941292 63.04121688 80.94704151 1.284033613 0.36068297 0.897356513 1

chr1_154927563_154929885 414.5442352 482.3153419 346.7731284 0.718975944 -0.475984593 0.654556605 1

chr1_154932734_154936051 1738.107631 1903.244357 1572.970905 0.826468183 -0.274968815 0.660775087 1

chr1_154954609_154957920 690.011266 707.462545 672.5599869 0.950665151 -0.07299082 0.933659997 1

chr1_154993751_154996714 267.9987493 266.1740268 269.8234717 1.013710747 0.019646052 0.991171668 1

chr1_155032965_155038264 1127.048639 1201.785738 1052.31154 0.875623255 -0.191617824 0.789521707 1

chr1_155063173_155067529 567.4932541 557.3644096 577.6220987 1.036345502 0.051505056 0.957612736 1

chr1_155111282_155113833 738.5586929 828.5417076 648.5756783 0.782791828 -0.353299399 0.672314621 1

chr1_155130969_155133313 140.5068973 151.0987897 129.9150049 0.859801757 -0.217924036 0.907869181 1

chr1_155144544_155153195 3668.136167 3877.535165 3458.737169 0.891993759 -0.164894478 0.754327425 1

chr1_155176348_155180514 1520.598756 1672.093229 1369.104282 0.818796619 -0.28842295 0.657894088 1

chr1_155192644_155198705 1289.097725 1438.940792 1139.254658 0.791731435 -0.336916961 0.623016424 1

chr1_155212996_155215695 654.5076612 666.4357213 642.5796011 0.964203419 -0.052590549 0.953795919 1

chr1_155223115_155226150 806.5423569 871.5698398 741.5148741 0.850780787 -0.233140642 0.77321981 1

chr1_155230503_155233211 752.5658902 853.5580635 651.5737169 0.763361914 -0.389560887 0.638422113 1

chr1_155241920_155244547 916.6032045 1074.70265 758.5037593 0.705780115 -0.502709312 0.513881163 1

chr1_155246851_155248806 484.510895 501.3277724 467.6940176 0.932910649 -0.100189183 0.921342363 1

chr1_155277190_155280548 1188.088547 1323.865555 1052.31154 0.794877951 -0.331194735 0.637960974 1

chr1_155292061_155297186 1897.545206 1967.286228 1827.804184 0.929099263 -0.106095356 0.862792275 1

chr1_155578861_155581269 614.0805761 737.4821721 490.67898 0.665343514 -0.587828704 0.51312148 1

chr1_155619406_155623587 433.4762209 397.2597318 469.69271 1.182331539 0.24163464 0.817663602 1

chr1_155656717_155661385 1940.142338 2158.411188 1721.873488 0.797750446 -0.325990584 0.591779012 1

chr1_155714357_155716680 533.087099 666.4357213 399.7384766 0.599815502 -0.737409287 0.439638969 1

chr1_155798663_155800333 55.5153814 79.05168466 31.97907813 0.404533797 -1.305667858 0.685355441 1

chr1_155825895_155828030 429.0511055 507.3316978 350.7705132 0.691402715 -0.532401826 0.611215981 1

chr1_155879817_155881964 297.5173952 324.2119725 270.8228179 0.835326394 -0.259588071 0.835561292 1

chr1_155902635_155905663 805.0956594 951.6221787 658.5691402 0.692048961 -0.531053985 0.511014062 1

chr1_155969867_155974085 540.9661065 489.3199215 592.6122916 1.211093735 0.27631053 0.771066014 1

chr1_155989158_155991178 465.513507 486.3179588 444.7090552 0.914440948 -0.129038087 0.899941824 1

chr1_156029555_156033929 804.5521668 884.5783448 724.5259888 0.819063674 -0.287952484 0.721842063 1

chr1_156039301_156041183 102.4663398 51.03336605 153.8993135 3.015660643 1.592474089 0.471259722 1

chr1_156049733_156053750 1297.106229 1459.954531 1134.257927 0.776913187 -0.364174695 0.59442662 1

chr1_156162181_156165064 1064.575767 1180.771999 948.3795357 0.803185998 -0.316193977 0.665130677 1

chr1_156181310_156184643 1197.567293 1300.850507 1094.28408 0.841206637 -0.249467863 0.722502682 1

chr1_156264311_156266394 564.5560396 650.4252536 478.6868257 0.735959779 -0.442301172 0.634527311 1

chr1_156304597_156309647 1685.571976 1796.174354 1574.969598 0.876846724 -0.189603419 0.764421253 1

chr1_156326179_156327639 119.9843291 96.06280668 143.9058516 1.498039216 0.583075391 0.771129409 1

chr1_156348329_156350130 91.51800518 119.0778541 63.95815626 0.5371121 -0.896704873 0.701979035 1

chr1_156390609_156392348 223.5271897 265.1733726 181.8810069 0.685894685 -0.543941019 0.703751585 1

chr1_156456436_156461875 920.2160241 1250.817795 589.614253 0.471383007 -1.085028345 0.161362634 1

chr1_156487283_156488685 64.49380058 55.03598299 73.95161817 1.343695781 0.426206543 0.886489165 1

chr1_156528568_156531498 256.4722589 214.1400066 298.8045113 1.395369861 0.480647578 0.718960743 1

chr1_156539868_156542872 188.5016754 191.1249591 185.8783916 0.97254902 -0.040157126 0.983115668 1

chr1_156560373_156562947 1024.597995 1174.768073 874.4279176 0.744340894 -0.425964596 0.564894531 1

chr1_156569488_156572415 995.6319984 1197.783121 793.480876 0.662457888 -0.594099349 0.427114029 1

chr1_156585570_156587190 77.01636703 102.0667321 51.96600196 0.509137511 -0.973872733 0.708537833 1

chr1_156607667_156611527 306.0844343 435.2845928 176.8842759 0.406364661 -1.29915315 0.292780337 1

chr1_156629219_156631002 98.00786923 110.071966 85.94377247 0.780796197 -0.356982068 0.876203918 1

chr1_156642683_156644647 77.9771259 43.02813216 112.9261196 2.624471804 1.392027098 0.591470042 1

chr1_156709655_156712208 546.6050875 707.462545 385.7476299 0.545255198 -0.874996477 0.354871463 1

chr1_156715472_156723351 3686.583522 4579.994439 2793.172605 0.609863755 -0.713441118 0.176630351 1

chr1_156735387_156739310 1264.182743 1544.009487 984.3559986 0.637532351 -0.649429542 0.34709678 1

chr1_156774042_156776269 90.03402841 142.0929015 37.97515528 0.267255823 -1.903706716 0.428430307 1

chr1_156782988_156784632 232.0530254 313.2047759 150.9012749 0.48179749 -1.053501217 0.453991344 1

chr1_156821378_156823501 113.5062375 123.0804711 103.9320039 0.844423189 -0.243961897 0.90880865 1

chr1_156859510_156860373 59.50949605 74.04841348 44.97057862 0.607313196 -0.719487379 0.815965768 1

chr1_157014041_157016818 691.592038 832.5443246 550.6397515 0.661393917 -0.596418319 0.486496671 1

chr1_157080945_157083318 104.5395907 165.107949 43.97123243 0.266318083 -1.908777705 0.386192119 1

chr1_157122984_157126450 323.0543529 406.2656199 239.843086 0.590360282 -0.760332431 0.524219394 1

chr1_157167566_157169236 53.0222481 87.05691855 18.98757764 0.218105326 -2.196903092 0.51585879 1

chr1_157329148_157330800 58.53434869 111.0726202 5.996077149 0.053983395 -4.211340475 0.22154702 1

chr1_157962915_157963908 49.03140355 97.06346092 0.999346191 0.010295802 -6.601799952 0.142309038 1

chr1_157970972_157972877 65.54023638 127.083088 3.997384766 0.031454892 -4.990571796 0.135810607 1

chr1_158115197_158116655 44.52649742 85.05561008 3.997384766 0.046997309 -4.411278046 0.290375801 1

chr1_158146600_158148029 45.52780568 88.05757279 2.998038574 0.034046346 -4.876356227 0.247573747 1

chr1_159823784_159825539 423.0092468 437.2859012 408.7325923 0.93470334 -0.097419546 0.92826634 1

chr1_159851155_159852739 82.50492281 90.05888126 74.95096436 0.832244009 -0.264921515 0.918881004 1

chr1_159926101_159932349 1553.189345 1078.705267 2027.673423 1.879728861 0.910524577 0.161675799 1

chr1_159974288_159975861 76.48726292 57.03729147 95.93723438 1.682008944 0.750185377 0.774808519 1

chr1_160000127_160003002 798.098928 949.6208702 646.5769859 0.680879082 -0.554529485 0.493989103 1

chr1_160030290_160033310 196.059558 287.1877658 104.9313501 0.365375418 -1.452548519 0.347000766 1

chr1_160053209_160054786 249.5965217 397.2597318 101.9333115 0.2565911 -1.962456965 0.15732793 1

chr1_160064965_160069894 893.1009104 1047.684985 738.5168355 0.704903521 -0.504502282 0.516354778 1

chr1_160092540_160096197 575.6619936 823.5384364 327.7855508 0.39802095 -1.329083725 0.154595229 1

chr1_160127427_160130685 258.5912914 398.260386 118.9221968 0.298604132 -1.743693967 0.198037822 1

chr1_160173802_160176729 640.0557288 725.4743213 554.6371363 0.764516565 -0.387380333 0.661189543 1

chr1_160213417_160216232 159.9980722 157.1027151 162.8934292 1.036859415 0.052220296 0.97939688 1

chr1_160230525_160233669 1196.122558 1383.904809 1008.340307 0.7286197 -0.456762092 0.515434215 1

chr1_160253334_160256096 701.5449506 770.5037619 632.5861392 0.82100331 -0.284540056 0.738729729 1

chr1_160311623_160314483 1125.089188 1261.824992 988.3533834 0.78327295 -0.352412959 0.622930791 1

chr1_160331013_160333203 154.4872796 135.0883219 173.8862373 1.287204067 0.364240789 0.835292051 1

chr1_160520008_160520810 48.49968336 48.03140334 48.96796338 1.019498911 0.027860234 1 1

chr1_160746395_160754095 1799.237795 1398.914622 2199.560967 1.57233396 0.652907675 0.293627262 1

chr1_160796540_160798645 114.4702665 69.0451423 159.8953906 2.315809416 1.211516529 0.556262699 1

chr1_160802668_160803996 99.00786944 111.0726202 86.94311866 0.78275923 -0.35335948 0.876731575 1

chr1_160959401_160960977 108.4833456 83.05430161 133.9123897 1.612347429 0.68916265 0.745273922 1

chr1_160982502_160985204 767.8739379 575.3761858 960.37169 1.669119636 0.739087365 0.369750368 1

chr1_161006049_161010029 1442.017967 1469.961073 1414.074861 0.961981162 -0.055919452 0.93358671 1

chr1_161013360_161017803 1870.065802 1971.288845 1768.842759 0.897302677 -0.156333379 0.799343935 1

chr1_161041658_161045808 256.5422393 321.2100098 191.8744688 0.597348971 -0.743354096 0.57744749 1

chr1_161064431_161069551 1208.016609 1233.806673 1182.226545 0.958194319 -0.061609836 0.930897007 1

chr1_161121976_161125495 1450.537262 1507.985934 1393.088591 0.923807417 -0.114335965 0.86322499 1

chr1_161128198_161131316 1190.054538 1273.832843 1106.276234 0.86846264 -0.203464307 0.772757599 1

chr1_161145691_161150966 1258.638595 1470.961727 1046.315462 0.711313859 -0.491441822 0.476847722 1

chr1_161196495_161198914 251.9941677 243.1589794 260.829356 1.072670056 0.101206383 0.942694707 1

chr1_161226308_161227265 48.00785853 60.03925417 35.97646289 0.599215686 -0.738852704 0.836653742 1

chr1_161358838_161360934 479.0131829 499.3264639 458.6999019 0.918637275 -0.122432771 0.903925204 1

chr1_161366753_161370769 1733.08801 1868.221459 1597.95456 0.855334657 -0.225439098 0.719330835 1

chr1_161409795_161411061 83.47810811 50.03271181 116.9235044 2.336941176 1.22462142 0.621708125 1

chr1_161416191_161421199 626.9340778 526.3441283 727.5240274 1.382221228 0.466988541 0.600222662 1

chr1_161423664_161425933 403.4690202 356.2329081 450.7051324 1.265197914 0.339363083 0.753112487 1

chr1_161438325_161442656 617.9621989 560.3663723 675.5580255 1.205564893 0.26970931 0.763982139 1

chr1_161493426_161495144 282.0249132 320.2093556 243.8404707 0.761503268 -0.393077867 0.758149883 1

chr1_161499138_161501748 254.0150968 277.1812234 230.8489702 0.832844907 -0.263880234 0.845381608 1

chr1_161507243_161508336 51.0085132 64.04187112 37.97515528 0.592973856 -0.753959596 0.826108018 1

chr1_161509235_161510464 79.49968999 79.05168466 79.94769532 1.011334492 0.016260237 1 1

chr1_161575357_161576661 104.5153919 128.0837422 80.94704151 0.631985294 -0.662037107 0.760239373 1

chr1_161581661_161582630 74.50688317 85.05561008 63.95815626 0.75195694 -0.411278046 0.879579093 1

chr1_161679531_161681674 200.472901 159.1040236 241.8417783 1.52002302 0.604093172 0.689532349 1

chr1_161695099_161697779 581.225762 926.6058228 235.8457012 0.254526461 -1.974112443 0.037224877 1

chr1_161718917_161721016 791.0361404 846.5534839 735.5187969 0.868839135 -0.202839007 0.803670715 1

chr1_161731965_161737543 1182.949893 1106.723585 1259.176201 1.137751303 0.186185239 0.792096021 1

chr1_161992933_161994562 164.5069024 175.1144913 153.8993135 0.878849673 -0.186311681 0.914274283 1

chr1_162038429_162041700 887.5691892 993.6496566 781.4887217 0.78648316 -0.34651222 0.656547966 1

chr1_162113601_162116878 269.0602276 361.2361793 176.8842759 0.489663788 -1.030136586 0.430703011 1

chr1_162120638_162125018 659.146642 883.5776906 434.7155933 0.491994759 -1.023285146 0.243942707 1

chr1_162128063_162131718 963.8321224 1471.962382 455.7018633 0.309587982 -1.691578627 0.028803871 1

chr1_162147849_162151114 234.9876238 216.141315 253.8339326 1.174388768 0.231910075 0.869606546 1

chr1_162160267_162165173 607.9818174 580.379457 635.5841778 1.095118323 0.131086756 0.885752626 1

chr1_162208976_162211608 173.5101745 189.1236507 157.8966983 0.834886053 -0.260348786 0.875180786 1

chr1_162252198_162257957 977.5070763 988.6463854 966.3677672 0.977465534 -0.032882262 0.966526485 1

chr1_162349883_162354028 394.3826874 215.1406608 573.6247139 2.66627755 1.414826968 0.198935585 1

chr1_162466657_162468859 847.5279772 890.5822703 804.4736842 0.903312036 -0.146703662 0.853995775 1

chr1_162529632_162532998 1565.050694 1643.074256 1487.027133 0.905027346 -0.14396671 0.82403202 1

chr1_162536313_162542081 1063.403759 916.5992804 1210.208238 1.320324229 0.400892252 0.583187943 1

chr1_162546959_162550372 190.9189421 67.04383383 314.7940503 4.695346795 2.231231718 0.163887998 1

chr1_162553147_162558422 370.9104783 234.1530913 507.6678653 2.168102341 1.116432858 0.321073034 1

chr1_162648143_162649304 69.9980529 67.04383383 72.95227198 1.088127988 0.121848259 0.969897724 1

chr1_162664761_162669504 639.4841132 615.4023553 663.5658712 1.078263457 0.108709722 0.903472661 1

chr1_162759310_162761771 446.0367206 502.3284266 389.7450147 0.775876885 -0.36610035 0.722451112 1

chr1_162815520_162816759 52.50687846 63.04121688 41.97254004 0.665795207 -0.58684961 0.862656858 1

chr1_162843785_162844864 47.52257393 82.05364737 12.99150049 0.158329348 -2.658999396 0.471482073 1

chr1_162882109_162885070 232.6148307 408.2669284 56.96273292 0.13952326 -2.841422437 0.055798435 1

chr1_163259261_163260404 70.01701955 96.06280668 43.97123243 0.457734205 -1.127417992 0.683701186 1

chr1_163290537_163293012 509.0798996 631.4128231 386.7469761 0.612510488 -0.707193548 0.467231923 1

chr1_163513236_163515321 124.9581693 61.03990841 188.8764302 3.094310511 1.629617977 0.40943104 1

chr1_163551467_163555715 217.9006352 66.04317959 369.7580909 5.598732422 2.485100232 0.100433509 1

chr1_163735435_163736436 46.01309029 66.04317959 25.98300098 0.39342444 -1.345841511 0.713366336 1

chr1_163762824_163767806 471.4297934 364.238142 578.6214449 1.588580047 0.667737788 0.50651322 1

chr1_163940166_163942761 159.4238405 43.02813216 275.8195489 6.410214318 2.680372593 0.134638588 1

chr1_163986354_163987337 41.98627451 21.01373896 62.95881006 2.996078431 1.583075391 0.686008002 1

chr1_164223823_164228840 366.3270895 102.0667321 630.5874468 6.178187876 2.627183743 0.026781835 1

chr1_164599649_164601909 193.9895771 178.1164541 209.8627002 1.178233091 0.236624977 0.879714573 1

chr1_164651554_164654269 449.3408417 206.1347727 692.5469107 3.359680183 1.748323905 0.095619975 1

chr1_164704123_164707315 340.5566458 427.2793589 253.8339326 0.594070196 -0.751294682 0.518864707 1

chr1_165141483_165145207 232.3878849 61.03990841 403.7358614 6.614293368 2.725587036 0.065376318 1

chr1_165227096_165229830 117.466343 66.04317959 168.8895064 2.557258863 1.354598207 0.505333373 1

chr1_165252304_165259507 825.4311772 720.4710501 930.3913043 1.291365287 0.368897152 0.644885304 1

chr1_165423796_165426660 191.0085431 204.1334642 177.8836221 0.871408433 -0.198579021 0.90039597 1

chr1_165566960_165568523 255.4853391 233.152437 277.8182412 1.191573396 0.252867819 0.851458301 1

chr1_165598909_165602532 1203.618962 1385.906117 1021.331808 0.736941554 -0.44037789 0.529748265 1

chr1_165666671_165668688 710.5508387 788.5155382 632.5861392 0.802249428 -0.31787724 0.707881284 1

chr1_165795745_165799939 2152.645326 2375.553157 1929.737496 0.812331852 -0.29985888 0.612326631 1

chr1_165863232_165867008 277.4101313 140.0915931 414.7286695 2.960410831 1.5657974 0.229401796 1

chr1_165884106_165885690 94.48661275 74.04841348 114.924812 1.552022611 0.634149576 0.783339729 1

chr1_166135056_166137233 588.6417217 805.5266602 371.7567832 0.461507734 -1.115573271 0.225737427 1

chr1_166458907_166460022 34.01374174 55.03598299 12.99150049 0.236054664 -2.082807105 0.65062582 1

chr1_166572514_166573567 42.01374345 63.04121688 20.98627002 0.332897603 -1.58684961 0.685168945 1

chr1_166699137_166700175 52.50360835 58.0379457 46.969271 0.809285553 -0.305279253 0.931344994 1

chr1_166807246_166814586 1445.91921 1322.8649 1568.973521 1.186042143 0.246155273 0.709834104 1

chr1_166844117_166846374 828.0315702 876.573111 779.4900294 0.889247023 -0.169343855 0.832989538 1

chr1_166944123_166945419 138.0196502 168.1099117 107.9293887 0.642016807 -0.63931703 0.729520924 1

chr1_167012707_167014068 85.99871035 84.05495584 87.94246485 1.046249611 0.065227086 0.983676617 1

chr1_167027783_167028970 58.99412642 50.03271181 67.95554102 1.358222222 0.441719542 0.889155685 1

chr1_167035621_167036565 67.49380122 58.0379457 76.94965675 1.3258508 0.406918436 0.888473449 1

chr1_167051700_167052869 54.00458971 61.03990841 46.969271 0.769484624 -0.378035595 0.911688012 1

chr1_167077635_167079515 142.0078786 154.1007524 129.9150049 0.843052372 -0.246305837 0.894713469 1

chr1_167188317_167192371 1283.060444 1375.899575 1190.221314 0.865049554 -0.209145315 0.760907241 1

chr1_167219966_167220719 38.50229731 42.02747792 34.9771167 0.832244009 -0.264921515 0.954374864 1

chr1_167266454_167267695 63.98366313 39.02551521 88.94181104 2.279068208 1.188444103 0.685332982 1

chr1_167423660_167425289 306.0360367 361.2361793 250.8358941 0.694381982 -0.526198582 0.667421282 1

chr1_167477909_167482449 223.4075036 82.05364737 364.7613599 4.445400925 2.15231354 0.144691802 1

chr1_167521602_167524123 877.4540791 807.5279687 947.3801895 1.173185606 0.230431276 0.768783041 1

chr1_167555725_167557133 50.47548496 13.00850507 87.94246485 6.760382102 2.757104791 0.438460719 1

chr1_167567913_167572664 359.387258 187.1223422 531.6521739 2.841200937 1.506500866 0.190789068 1

chr1_167632114_167633963 262.9111092 127.083088 398.7391304 3.137625444 1.64967314 0.218345691 1

chr1_167651343_167654045 366.3866055 193.1262676 539.6469434 2.79427004 1.48247145 0.193736531 1

chr1_167682552_167686691 948.9478812 869.5685313 1028.327231 1.182571809 0.241927791 0.750716275 1

chr1_167764519_167766709 125.5140883 147.0961727 103.9320039 0.706558179 -0.501119736 0.79797407 1

chr1_167788938_167789818 59.99870479 58.0379457 61.95946387 1.067568177 0.094328206 0.981022474 1

chr1_167794228_167795944 59.97254389 18.01177625 101.9333115 5.659259259 2.500613231 0.427211886 1

chr1_167822777_167824860 142.5108218 159.1040236 125.9176201 0.791416944 -0.337490141 0.854244311 1

chr1_167894667_167899550 492.7810079 158.1033693 827.4586466 5.233655994 2.3878191 0.0205374 1

chr1_168025199_168026051 74.50884524 88.05757279 60.96011768 0.692275698 -0.530581391 0.843524915 1

chr1_168104582_168107209 616.8654033 411.2688911 822.4619156 1.99981553 0.999866927 0.267407167 1

chr1_168138857_168141834 233.9294156 126.0824338 341.7763975 2.710737628 1.438685482 0.308206816 1

chr1_168157455_168160245 129.4447628 45.02944063 213.860085 4.749339143 2.247726781 0.254684884 1

chr1_168193788_168196817 1300.455478 1232.806019 1368.104936 1.109748748 0.150233081 0.826559694 1

chr1_168211430_168214409 222.3976931 66.04317959 378.7522066 5.734917806 2.519772809 0.092850433 1

chr1_168309814_168312459 146.4343021 46.03009487 246.8385093 5.362546178 2.422918166 0.191836149 1

chr1_168365385_168367550 164.49513 157.1027151 171.8875449 1.094109321 0.129756896 0.941511129 1

chr1_168600887_168601929 40.01832118 68.04448806 11.9921543 0.176239908 -2.50438745 0.54496366 1

chr1_168678420_168682105 215.0072402 226.1478574 203.8666231 0.901474926 -0.14964073 0.920770716 1

chr1_168704316_168705365 42.51014644 58.0379457 26.98234717 0.464908722 -1.104980603 0.775539165 1

chr1_168773029_168773959 53.99935753 53.03467452 54.96404053 1.036379332 0.051552149 0.993732292 1

chr1_168811721_168813206 74.02617672 114.0745829 33.97777051 0.29785575 -1.747314282 0.517079808 1

chr1_168829527_168831072 89.51735073 116.0758914 62.95881006 0.542393509 -0.882588181 0.710190184 1

chr1_168849179_168850365 74.03206292 123.0804711 24.98365479 0.202986344 -2.300545425 0.401023629 1

chr1_168872177_168880544 2011.116846 2190.432123 1831.801569 0.836274062 -0.257952278 0.668429287 1

chr1_168882072_168884471 199.9706118 155.1014066 244.8398169 1.578578958 0.658626424 0.663478088 1

chr1_168907240_168910901 376.1740504 642.4200197 109.9280811 0.171115591 -2.546956883 0.029243317 1

chr1_168941626_168944489 142.5258644 182.119071 102.9326577 0.565194283 -0.823181223 0.650070063 1

chr1_169041471_169044674 248.5317732 297.1943082 199.8692383 0.672520411 -0.572350041 0.672856681 1

chr1_169046507_169049740 204.4853282 182.119071 226.8515855 1.245622352 0.316866738 0.833650105 1

chr1_169073761_169080281 1875.594907 2021.321557 1729.868257 0.855810522 -0.224636678 0.714362295 1

chr1_169137066_169138129 49.48921922 33.0215898 65.95684864 1.997385621 0.99811289 0.774598493 1

chr1_169213969_169216804 197.0347053 250.1635591 143.9058516 0.575247059 -0.797746393 0.601008484 1

chr1_169275541_169279377 386.5533855 468.3061826 304.8005884 0.650857494 -0.619586397 0.572493587 1

chr1_169336296_169338632 505.0354252 559.3657181 450.7051324 0.805743216 -0.311607959 0.749800003 1

chr1_169452178_169457675 1774.057933 1863.218188 1684.897679 0.904294349 -0.145135646 0.816167516 1

chr1_169465661_169468992 180.97911 149.0974812 212.8607388 1.427661534 0.51365399 0.747851746 1

chr1_169495753_169501858 2027.087418 2161.41315 1892.761687 0.87570564 -0.191482091 0.750191018 1

chr1_169555167_169556393 76.50295946 81.05299314 71.95292579 0.887726943 -0.171812111 0.952006289 1

chr1_169580042_169581290 58.48660505 38.02486098 78.94834913 2.07622979 1.053966125 0.734736428 1

chr1_169710420_169711424 41.51407036 63.04121688 19.98692383 0.317045337 -1.657238938 0.674900599 1

chr1_169747602_169751866 399.5193791 429.2806674 369.7580909 0.86134345 -0.215339487 0.843352892 1

chr1_169762637_169765270 1178.07154 1287.842002 1068.301079 0.829528061 -0.26963731 0.702579918 1

chr1_169784284_169786858 146.5134388 167.1092575 125.9176201 0.753504755 -0.408311479 0.820551749 1

chr1_169857261_169864806 4448.29984 4143.709192 4752.890487 1.147013525 0.197882403 0.697648033 1

chr1_169894308_169895569 86.50426965 93.06084397 79.94769532 0.85909059 -0.219117826 0.931682755 1

chr1_169912781_169915216 196.9418341 108.0706575 285.8130108 2.644686517 1.403096725 0.362016475 1

chr1_170042694_170044786 524.5115576 542.354596 506.6685191 0.934201577 -0.098194214 0.920137596 1

chr1_170066457_170067681 63.49903255 62.04056265 64.95750245 1.047016656 0.066284393 0.987804999 1

chr1_170135336_170139782 270.8797178 87.05691855 454.7025171 5.223048606 2.38489213 0.078106367 1

chr1_170499792_170502605 1239.596733 1387.907426 1091.286041 0.786281578 -0.346882041 0.617232174 1

chr1_170530994_170533307 189.5029836 194.1269218 184.8790454 0.952361701 -0.070418491 0.967294774 1

chr1_170632999_170634190 67.02094305 99.06476939 34.9771167 0.353073216 -1.501960713 0.59909148 1

chr1_171186208_171190366 844.8562958 625.4088977 1064.303694 1.701772549 0.767038226 0.334745173 1

chr1_171255072_171256854 89.52062085 121.0791626 57.96207911 0.478712256 -1.062769352 0.654596724 1

chr1_171282066_171284562 223.5042989 230.1504743 216.8581236 0.942244956 -0.085825928 0.955083178 1

chr1_171371238_171374585 747.4874064 728.476284 766.4985289 1.052194211 0.073401018 0.931032096 1

chr1_171393591_171394828 67.99609041 62.04056265 73.95161817 1.191988193 0.253369946 0.93231356 1

chr1_171453233_171456372 1092.545034 1161.759568 1023.3305 0.880845338 -0.183039366 0.800880911 1

chr1_171613807_171615021 72.4911862 59.03859994 85.94377247 1.455721724 0.541734596 0.842965461 1

chr1_171710009_171713090 1149.541122 1212.792934 1086.28931 0.895692314 -0.158924868 0.823845137 1

chr1_171749227_171752364 1141.068916 1246.815178 1035.322654 0.830373797 -0.268167175 0.70712894 1

chr1_172008727_172010851 119.0229162 154.1007524 83.94508009 0.544741533 -0.876356227 0.662535269 1

chr1_172058928_172061068 83.96077662 24.01570167 143.9058516 5.992156863 2.583075391 0.313469989 1

chr1_172133217_172134510 54.97973707 24.01570167 85.94377247 3.578649237 1.839415144 0.573860063 1

chr1_172213946_172215774 171.4343074 71.04645077 271.8221641 3.825978091 1.935828612 0.248778753 1

chr1_172246971_172249511 139.9346277 40.02616945 239.843086 5.992156863 2.583075391 0.177220941 1

chr1_172366873_172370663 319.3610886 107.0700033 531.6521739 4.965463319 2.31192834 0.064008797 1

chr1_172400358_172401962 93.97712932 59.03859994 128.9156587 2.183582586 1.126697097 0.625476085 1

chr1_172412162_172413953 667.0217254 700.4579654 633.5854854 0.904530345 -0.144759191 0.868806159 1

chr1_172415359_172420804 889.4756644 852.5574093 926.3939195 1.08660591 0.119828799 0.878636752 1

chr1_172437428_172441290 355.4775123 321.2100098 389.7450147 1.213365097 0.279013717 0.807732921 1

chr1_172464471_172471691 1120.768062 766.501145 1475.034979 1.924374136 0.944389315 0.190569194 1

chr1_172497581_172503921 1791.046165 1862.217534 1719.874796 0.923562776 -0.114718068 0.854021847 1

chr1_172606889_172613933 826.595337 972.6359176 680.5547564 0.699701444 -0.515188625 0.519569299 1

chr1_172901217_172904345 295.4840397 271.177298 319.7907813 1.179268263 0.237891944 0.849877777 1

chr1_172945021_172946696 63.98300911 38.02486098 89.94115723 2.365325077 1.242038473 0.672061903 1

chr1_173088983_173094557 543.3205871 269.1759896 817.4651846 3.036917171 1.602607561 0.096147516 1

chr1_173158845_173161075 131.4833506 106.069349 156.8973521 1.479195955 0.564813185 0.766597505 1

chr1_173349952_173351301 93.98497759 71.04645077 116.9235044 1.645733223 0.718730491 0.755659166 1

chr1_173388951_173390041 45.98889146 29.01897285 62.95881006 2.169574037 1.117411819 0.760438339 1

chr1_173445176_173448184 1230.049315 1305.853778 1154.244851 0.883900533 -0.178044065 0.798503556 1

chr1_173475241_173477604 145.9784485 113.0739287 178.8829683 1.582000116 0.661749705 0.71188557 1

chr1_173629436_173631348 72.47221955 30.01962709 114.924812 3.82832244 1.936712346 0.480621459 1

chr1_173683099_173685773 881.0250413 919.6012431 842.4488394 0.916102328 -0.12641934 0.872383889 1

chr1_173792141_173795368 1087.077407 1205.788355 968.3664596 0.803098202 -0.316351685 0.662705011 1

chr1_173844542_173846181 78.01375115 99.06476939 56.96273292 0.575004951 -0.798353715 0.757679024 1

chr1_173894937_173896016 46.50949327 61.03990841 31.97907813 0.523904425 -0.932624447 0.797991792 1

chr1_173989373_173994266 1716.534049 1769.15669 1663.911409 0.940511046 -0.088483207 0.888732594 1

chr1_174158377_174160837 119.9477039 40.02616945 199.8692383 4.993464052 2.320040985 0.261011366 1

chr1_174207579_174208868 67.48791502 49.03205758 85.94377247 1.75280779 0.809667801 0.775431787 1

chr1_174710124_174712095 98.4833435 73.04775925 123.9189277 1.696409705 0.762484642 0.734052566 1

chr1_174736524_174738028 59.97908412 28.01831861 91.93984962 3.281419234 1.714319924 0.577913599 1

chr1_174768479_174769649 54.50687889 65.04252536 43.97123243 0.67603821 -0.564823304 0.864498987 1

chr1_174817721_174818963 57.51734389 84.05495584 30.97973194 0.368565204 -1.440008222 0.647774993 1

chr1_174843306_174845774 288.510199 304.1988878 272.8215103 0.896852425 -0.157057482 0.902766267 1

chr1_174958675_174960679 298.0288407 342.2237488 253.8339326 0.741719222 -0.431054938 0.728478547 1

chr1_174967314_174971579 2244.096621 2392.564279 2095.628964 0.87589244 -0.191174378 0.744243631 1

chr1_174991348_174993998 891.0243894 928.6071312 853.4416475 0.919055668 -0.121775845 0.876574108 1

chr1_174998241_174999339 69.99936095 69.0451423 70.9535796 1.027640428 0.039335553 0.99442929 1

chr1_175098138_175099967 173.0046152 180.1177625 165.8914678 0.921016703 -0.118700775 0.945210616 1

chr1_175124156_175128344 376.0824872 502.3284266 249.8365479 0.497356977 -1.007646379 0.366562514 1

chr1_175186334_175192318 776.4815264 748.4893687 804.4736842 1.074796407 0.104063404 0.900081414 1

chr1_175195370_175197606 116.5225887 151.0987897 81.9463877 0.542336493 -0.882739844 0.664089138 1

chr1_175237638_175239592 85.03402734 137.0896304 32.97842432 0.240561042 -2.055525073 0.410676298 1

chr1_175286280_175289317 303.5546758 387.2531894 219.8561621 0.567732347 -0.816717152 0.506775754 1

chr1_176146610_176149196 135.939205 43.02813216 228.8502779 5.318619851 2.411051924 0.212083939 1

chr1_176712080_176712993 32.01374131 53.03467452 10.99280811 0.207275866 -2.270375945 0.638416453 1

chr1_176792536_176793504 27.01570231 51.03336605 2.998038574 0.058746636 -4.089349951 0.487694618 1

chr1_176823151_176824213 36.01505021 59.03859994 12.99150049 0.220050958 -2.184090441 0.621301981 1

chr1_176954257_176957640 147.5579125 236.1543998 58.9614253 0.249673203 -2.00188711 0.271893573 1

chr1_177016515_177018535 71.03729446 128.0837422 13.99084668 0.109232026 -3.194532187 0.271861284 1

chr1_177133171_177134140 36.02093642 68.04448806 3.997384766 0.058746636 -4.089349951 0.390198353 1

chr1_177139703_177142179 222.6095963 390.2551521 54.96404053 0.140841294 -2.82785771 0.062416629 1

chr1_177149987_177152336 192.5781968 312.2041217 72.95227198 0.23366851 -2.09746477 0.186622873 1

chr1_177177745_177178684 27.01504829 50.03271181 3.997384766 0.079895425 -3.645743299 0.527365844 1

chr1_177242867_177246007 196.0811407 320.2093556 71.95292579 0.224705882 -2.153890203 0.171895575 1

chr1_177269576_177272396 123.5637936 221.1445862 25.98300098 0.117493272 -3.089349951 0.139941891 1

chr1_177353417_177354616 39.01832097 67.04383383 10.99280811 0.163964491 -2.608544681 0.537061629 1

chr1_177615990_177617712 57.02682711 98.06411515 15.98953906 0.163051887 -2.616596954 0.423170483 1

chr1_177840251_177844836 394.1662059 648.4239451 139.9084668 0.215766965 -2.212454095 0.050085983 1

chr1_177905841_177906846 38.51733983 65.04252536 11.9921543 0.184374057 -2.439292422 0.565307983 1

chr1_177927070_177927921 39.00981868 54.03532876 23.9843086 0.443863471 -1.171812111 0.776161485 1

chr1_178001151_178004265 194.0595575 285.1864573 102.9326577 0.360931086 -1.470204691 0.343922547 1

chr1_178062001_178064226 550.0426291 615.4023553 484.6829029 0.787587013 -0.344488773 0.71444885 1

chr1_178498948_178500448 93.52323779 129.0843965 57.96207911 0.449024674 -1.15513337 0.617883705 1

chr1_178505341_178507433 102.5068892 113.0739287 91.93984962 0.813095032 -0.298504116 0.894265488 1

chr1_178510465_178513449 819.5972976 968.6333007 670.5612945 0.692275698 -0.530581391 0.508548307 1

chr1_178598286_178599229 40.51014601 56.03663723 24.98365479 0.445845005 -1.165385842 0.771351496 1

chr1_178614030_178615230 56.48725865 37.02420674 75.95031055 2.051369016 1.036587038 0.74474826 1

chr1_178621706_178627755 525.5887324 661.4324502 389.7450147 0.589243867 -0.763063257 0.426781569 1

chr1_178693378_178696311 913.1054929 1074.70265 751.508336 0.699270944 -0.516076536 0.503367973 1

chr1_178984305_178987929 267.5474738 340.2224403 194.8725073 0.5727797 -0.803947732 0.538638975 1

chr1_179050220_179053277 721.5423388 786.5142297 656.5704478 0.834785212 -0.260523051 0.757602297 1

chr1_179103132_179104280 94.9993663 94.06149821 95.93723438 1.019941594 0.028486539 0.995248751 1

chr1_179197528_179199775 667.5737203 780.5103043 554.6371363 0.710608346 -0.492873462 0.570514191 1

chr1_179262276_179264076 212.0209741 244.1596336 179.8823145 0.736740598 -0.440771351 0.764698952 1

chr1_179333803_179335942 488.5305165 535.3500164 441.7110166 0.825088266 -0.277379631 0.779835499 1

chr1_179367310_179368403 49.50426173 56.03663723 42.97188623 0.766853408 -0.382977277 0.91598529 1

chr1_179554942_179556147 112.9915219 100.0654236 125.9176201 1.258352941 0.331536624 0.874934448 1

chr1_179560289_179561737 255.4663725 204.1334642 306.7992808 1.502934769 0.587782394 0.660203666 1

chr1_179716278_179718393 87.9673177 38.02486098 137.9097744 3.626831785 1.858709834 0.445197809 1

chr1_179849620_179853188 1223.171615 1485.971541 960.37169 0.646292115 -0.629741704 0.366930519 1

chr1_179922548_179925143 800.6208383 985.6444227 615.597254 0.62456322 -0.679080483 0.402138254 1

chr1_180096675_180098615 109.5029665 114.0745829 104.9313501 0.919848641 -0.120531606 0.958418846 1

chr1_180134229_180141248 978.6313408 1179.771345 777.491337 0.659018665 -0.601608768 0.424137044 1

chr1_180197915_180201615 545.5409931 608.3977756 482.6842105 0.793369453 -0.333935244 0.723797706 1

chr1_180216587_180219167 125.4715769 82.05364737 168.8895064 2.058281524 1.041440322 0.593399039 1

chr1_180470185_180473127 1180.62746 1375.899575 985.3553448 0.716153535 -0.481659176 0.494707363 1

chr1_180524006_180526802 265.6318423 467.3055283 63.95815626 0.136865824 -2.869165849 0.039528537 1

chr1_180529928_180533287 150.0602021 242.1583252 57.96207911 0.239356128 -2.062769352 0.254197077 1

chr1_180553731_180556827 219.5847428 349.2283285 89.94115723 0.257542559 -1.957117239 0.185672185 1

chr1_180891690_180893473 124.510818 141.0922473 107.9293887 0.764956195 -0.38655096 0.845104233 1

chr1_180920343_180924047 355.0118483 373.2440301 336.7796665 0.902304228 -0.148314149 0.898408961 1

chr1_180930766_180931610 40.00981889 55.03598299 24.98365479 0.453951277 -1.139390633 0.77830631 1

chr1_180990462_180992951 602.0779574 721.4717043 482.6842105 0.669027223 -0.57986318 0.522194877 1

chr1_181001943_181004046 139.5474465 212.1386981 66.95619483 0.315624615 -1.663718374 0.371063721 1

chr1_181100161_181106963 764.4998365 764.4998365 764.4998365 1 3.20E-16 1 1

chr1_181109775_181112168 207.518684 236.1543998 178.8829683 0.757483106 -0.400714382 0.788116231 1

chr1_181997697_181999957 509.5697624 616.4030095 402.7365152 0.653365589 -0.614037622 0.527463788 1

chr1_182064249_182067104 169.024235 206.1347727 131.9136973 0.639939082 -0.643993517 0.697089444 1

chr1_182069238_182072512 395.6272919 590.3859994 200.8685845 0.340232635 -1.555406563 0.158806272 1

chr1_182109136_182116825 1139.526078 1179.771345 1099.280811 0.931774463 -0.101947304 0.88727401 1

chr1_182148374_182153767 812.5456283 882.5770364 742.5142203 0.841302447 -0.249303555 0.757212383 1

chr1_182186144_182189417 432.5468551 504.3297351 360.7639751 0.715333541 -0.483312006 0.643309118 1

chr1_182196487_182198799 148.4872783 129.0843965 167.8901602 1.300623195 0.379203058 0.832180242 1

chr1_182204213_182206447 162.009191 176.1151456 147.9032363 0.839809863 -0.251865363 0.883856422 1

chr1_182228040_182229275 66.49249297 55.03598299 77.94900294 1.416327986 0.502155396 0.862670274 1

chr1_182283276_182285553 141.9751775 104.0680406 179.8823145 1.728506787 0.789526269 0.664153603 1

chr1_182285934_182287845 130.502317 134.0876677 126.9169663 0.946522291 -0.079291613 0.970845603 1

chr1_182308454_182311122 249.0680716 353.2309454 144.9051978 0.410227925 -1.285502393 0.345952075 1

chr1_182358246_182362304 1507.561474 1602.047432 1413.075515 0.882043494 -0.181078297 0.781862133 1

chr1_182428833_182430672 73.47679793 38.02486098 108.9287349 2.864671483 1.518369702 0.573422529 1

chr1_182452802_182454872 88.97124205 45.02944063 132.9130435 2.951692084 1.56154223 0.515461145 1

chr1_182557191_182559022 287.5245873 325.2126268 249.8365479 0.768225239 -0.380398733 0.763632688 1

chr1_182569528_182574321 778.4959154 772.5050704 784.4867603 1.015510176 0.022204697 0.980031735 1

chr1_182600930_182606475 646.9746315 608.3977756 685.5514874 1.126814585 0.172250143 0.845740467 1

chr1_182652649_182658691 807.4933055 797.5214263 817.4651846 1.025007176 0.035634009 0.966337733 1

chr1_182673080_182674175 69.00786303 81.05299314 56.96273292 0.70278383 -0.508847098 0.857338621 1

chr1_182685380_182688157 131.4499954 55.03598299 207.8640078 3.776874629 1.917192895 0.321995034 1

chr1_182700589_182701501 60.00851512 73.04775925 46.969271 0.642994001 -0.637122817 0.836352733 1

chr1_182750326_182752592 142.4800828 112.0732745 172.8868911 1.542623716 0.625386196 0.730680186 1

chr1_182758007_182759460 258.5775569 377.2466471 139.9084668 0.370867357 -1.431024806 0.286792315 1

chr1_182807628_182810638 708.0681697 812.5312398 603.6050997 0.742870022 -0.428818287 0.61342409 1

chr1_182921734_182922781 51.01243733 70.04579654 31.97907813 0.456545285 -1.131170126 0.740127259 1

chr1_183028357_183029779 73.00720986 84.05495584 61.95946387 0.737130408 -0.440008222 0.872561988 1

chr1_183118263_183119578 96.0019826 99.06476939 92.93919581 0.938165973 -0.092084919 0.972313721 1

chr1_183128005_183130560 273.9987506 272.1779523 275.8195489 1.013379469 0.019174506 0.991298339 1

chr1_183154550_183156108 213.0281685 256.1674845 169.8888526 0.663194444 -0.592496173 0.685994077 1

chr1_183187154_183188631 69.47941316 38.02486098 100.9339653 2.654420365 1.40839686 0.613494728 1

chr1_183196990_183198555 84.46829799 36.0235525 132.9130435 3.689615105 1.883470325 0.45056122 1

chr1_183202982_183204250 53.48071778 24.01570167 82.94573389 3.453812636 1.788189821 0.591018133 1

chr1_183317290_183319977 273.4107844 137.0896304 409.7319385 2.988788703 1.579560907 0.228764431 1

chr1_183343990_183345031 48.99216221 37.02420674 60.96011768 1.646493552 0.719396862 0.838761975 1

chr1_183429566_183431502 99.47484142 61.03990841 137.9097744 2.259337833 1.17590001 0.5984533 1

chr1_183439174_183442909 1099.652949 1333.872097 865.4338018 0.648813184 -0.62412496 0.388046576 1

chr1_183603663_183605513 495.0556978 580.379457 409.7319385 0.705972504 -0.5023161 0.609593186 1

chr1_183667737_183670363 181.5481095 255.1668302 107.9293887 0.422975779 -1.241353044 0.437966122 1

chr1_183686704_183690313 297.5664469 399.2610403 195.8718535 0.490585942 -1.027422202 0.409326961 1

chr1_183711839_183713436 67.47287251 26.01701014 108.9287349 4.186827552 2.065857497 0.473485868 1

chr1_183773262_183775463 405.0033567 410.2682369 399.7384766 0.974334449 -0.037511019 0.974556739 1

chr1_183848625_183853528 478.3205732 204.1334642 752.5076822 3.686351403 1.882193603 0.066416066 1

chr1_184005524_184007126 378.5121804 397.2597318 359.7646289 0.905615647 -0.14302921 0.899052762 1

chr1_184019433_184022149 843.0041044 849.5554466 836.4527623 0.984577011 -0.022424041 0.979065121 1

chr1_184124991_184127073 86.53239261 136.0889761 36.97580909 0.271703191 -1.879896585 0.44477714 1

chr1_184181081_184183638 168.9797615 138.0902846 199.8692383 1.447380885 0.533444623 0.747517111 1

chr1_184354786_184362363 1245.956447 1179.771345 1312.141549 1.112199881 0.153416088 0.825334359 1

chr1_184373157_184379759 927.8863986 754.4932941 1101.279503 1.459627954 0.545600686 0.476780626 1

chr1_184392741_184397462 671.9066185 529.346091 814.4671461 1.53862881 0.621645227 0.473396423 1

chr1_184411869_184413739 110.4846541 87.05691855 133.9123897 1.538216513 0.621258585 0.767487787 1

chr1_184424977_184428019 319.4709643 275.179915 363.7620137 1.32190612 0.402619722 0.737554437 1

chr1_184473020_184474331 55.01309221 75.04906772 34.9771167 0.466056645 -1.101422783 0.733911924 1

chr1_184557884_184560952 204.5127972 224.1465489 184.8790454 0.824813259 -0.277860571 0.854295692 1

chr1_184590542_184594742 429.0341009 481.3146876 376.7535142 0.78275923 -0.35335948 0.736233912 1

chr1_184600911_184603465 304.5402875 366.2394505 242.8411245 0.663066538 -0.592774444 0.629064238 1

chr1_184722662_184725392 820.5829093 947.6195617 693.5462569 0.731882588 -0.450315872 0.574485021 1

chr1_184769980_184774299 163.5664182 265.1733726 61.95946387 0.233656431 -2.097539349 0.22524696 1

chr1_184796079_184799405 169.5324104 219.1432777 119.921543 0.547228937 -0.869783574 0.598463329 1

chr1_184805961_184809238 229.0491007 304.1988878 153.8993135 0.505916753 -0.983028082 0.487178272 1

chr1_184830340_184831834 66.50949755 81.05299314 51.96600196 0.641136125 -0.641297394 0.823736432 1

chr1_184847424_184849587 133.0085307 146.0955185 119.921543 0.820843406 -0.284821073 0.882232086 1

chr1_184872400_184876308 393.0432495 459.3002944 326.7862046 0.711487035 -0.491090627 0.652217191 1

chr1_184896030_184901320 860.5377903 918.6005889 802.4749918 0.873584234 -0.194981275 0.805173719 1

chr1_184934936_184936016 48.98823808 31.02028132 66.95619483 2.158465106 1.110005771 0.751616863 1

chr1_185013911_185015888 616.592022 757.4952568 475.6887872 0.627975928 -0.671218836 0.454711812 1

chr1_185124884_185127639 976.0970041 1124.735362 827.4586466 0.735691857 -0.442826472 0.556369899 1

chr1_185226083_185227504 143.0078789 155.1014066 130.9143511 0.844056504 -0.244588513 0.895047608 1

chr1_185457696_185458834 38.51930189 68.04448806 8.994115723 0.132179931 -2.919424949 0.498281137 1

chr1_185493830_185494854 35.01766609 62.04056265 7.994769532 0.128863588 -2.95608342 0.52244792 1

chr1_185553603_185555123 89.04710865 161.105332 16.98888526 0.105452036 -3.245341146 0.201713004 1

chr1_185555142_185556795 131.0719705 241.1576709 20.98627002 0.087023025 -3.522459023 0.088237799 1

chr1_185702894_185704087 57.01440068 79.05168466 34.9771167 0.44245884 -1.176384841 0.710011342 1

chr1_186047957_186049004 55.99608784 50.03271181 61.95946387 1.238379085 0.308453011 0.927162415 1

chr1_186343183_186346249 797.5979469 947.6195617 647.5763321 0.683371638 -0.549257722 0.498194511 1

chr1_186546375_186547710 74.4990349 73.04775925 75.95031055 1.039734981 0.056215845 0.988610774 1

chr1_187126326_187127154 32.01504936 55.03598299 8.994115723 0.16342246 -2.613321822 0.592179092 1

chr1_188737723_188739214 90.04383875 157.1027151 22.9849624 0.146305316 -2.772945902 0.262743942 1

chr1_189686662_189689110 133.57099 242.1583252 24.98365479 0.103170745 -3.276894157 0.105083701 1

chr1_189743980_189745248 38.02224489 72.04710501 3.997384766 0.055482934 -4.171812111 0.364292573 1

chr1_190421462_190422496 42.48398554 18.01177625 66.95619483 3.717356572 1.894277079 0.627507203 1

chr1_190636682_190637775 58.49576137 52.03402028 64.95750245 1.248366013 0.320040985 0.921748179 1

chr1_191121706_191122685 35.01439598 57.03729147 12.99150049 0.227772044 -2.134337406 0.635844071 1

chr1_191496620_191498928 97.54874552 172.1125286 22.9849624 0.133546132 -2.904589908 0.221356968 1

chr1_191669852_191672549 89.53631738 145.0948643 33.97777051 0.234176245 -2.094333358 0.387871507 1

chr1_192777021_192778783 235.5265383 276.1805692 194.8725073 0.705598181 -0.503081253 0.718109047 1

chr1_193026888_193030561 927.6398321 1141.746484 713.5331807 0.624948875 -0.678189922 0.377040564 1

chr1_193072980_193075565 512.5579906 601.393196 423.7227852 0.704568638 -0.505187836 0.602161149 1

chr1_193080015_193082827 189.4486998 111.0726202 267.8247793 2.411258317 1.269786215 0.417490942 1

chr1_193090007_193092763 724.6116658 895.5855414 553.6377901 0.61818527 -0.693888816 0.410003756 1

chr1_193198712_193199964 48.48725694 29.01897285 67.95554102 2.341762452 1.227594737 0.72813321 1

chr1_195691857_195692747 48.50426152 55.03598299 41.97254004 0.762638146 -0.3909294 0.915402833 1

chr1_195732165_195733115 56.01570851 80.0523389 31.97907813 0.399477124 -1.323815204 0.679458484 1

chr1_195969691_195972696 199.0687149 304.1988878 93.938542 0.30880633 -1.695225771 0.271633109 1

chr1_196960342_196962128 158.4526173 86.05626432 230.8489702 2.68253534 1.423597177 0.409585405 1

chr1_197169017_197171883 1060.993033 1050.686948 1071.299117 1.019617803 0.028028468 0.970756103 1

chr1_197189226_197193372 681.3343511 428.2800131 934.388689 2.18172378 1.125468459 0.194995387 1

chr1_197453048_197457715 1447.476438 1411.923127 1483.029748 1.050361538 0.070885994 0.915449276 1

chr1_197588745_197590410 67.01178673 85.05561008 48.96796338 0.575717032 -0.796568202 0.78000838 1

chr1_197632180_197633435 58.99020228 44.02878639 73.95161817 1.679619727 0.748134637 0.809711445 1

chr1_197730845_197734459 198.4500097 122.0798168 274.8202027 2.251151827 1.170663361 0.443063274 1

chr1_197741283_197746284 1924.352276 1699.110893 2149.593658 1.265128525 0.339283956 0.577588229 1

chr1_197767682_197769342 73.47679793 38.02486098 108.9287349 2.864671483 1.518369702 0.573422529 1

chr1_197807208_197808334 45.478754 13.00850507 77.94900294 5.992156863 2.583075391 0.496112258 1

chr1_197870501_197873173 1446.533992 1498.980046 1394.087937 0.930024346 -0.104659611 0.874905283 1

chr1_197907321_197909795 224.441513 135.0883219 313.7947041 2.3228855 1.215918042 0.396693941 1

chr1_197945703_197946484 30.00850871 43.02813216 16.98888526 0.394832041 -1.340689023 0.789144933 1

chr1_198125096_198129572 1538.585025 1669.091266 1408.078784 0.843620006 -0.245334787 0.705531271 1

chr1_198624348_198628412 224.9222195 106.069349 343.7750899 3.241040819 1.696457191 0.241867838 1

chr1_198636819_198639192 147.0019935 150.0981354 143.9058516 0.958745098 -0.060780799 0.976768266 1

chr1_198650593_198652263 91.96731856 42.02747792 141.9071592 3.376532835 1.755542587 0.457834403 1

chr1_198848854_198851859 236.9320324 133.0870134 340.7770513 2.560558258 1.356458384 0.332696277 1

chr1_198858593_198862688 260.4382506 166.1086032 354.767898 2.135758721 1.094748674 0.41030814 1

chr1_198871278_198873908 153.4369197 57.03729147 249.8365479 4.380231625 2.131007161 0.234281606 1

chr1_198940313_198941242 46.49183467 34.02224403 58.9614253 1.733025759 0.793293099 0.828340163 1

chr1_199210895_199213030 252.4742201 213.1393523 291.8090879 1.369100003 0.453227829 0.736483158 1

chr1_199241841_199243979 100.9921733 89.05822703 112.9261196 1.268003231 0.342558422 0.879155409 1

chr1_199997768_199998790 52.00197319 55.03598299 48.96796338 0.889744504 -0.168536979 0.965359351 1

chr1_200007963_200009469 168.001998 171.1118744 164.8921216 0.963650957 -0.05341741 0.978064802 1

chr1_200076463_200077691 53.02028603 84.05495584 21.98561621 0.261562403 -1.934772914 0.564496459 1

chr1_200098472_200099575 57.49249104 46.03009487 68.95488721 1.498039216 0.583075391 0.854771892 1

chr1_200153367_200155663 135.5042801 142.0929015 128.9156587 0.907263187 -0.140406974 0.943453609 1

chr1_200264606_200265617 60.01701741 86.05626432 33.97777051 0.394832041 -1.340689023 0.661448749 1

chr1_200270211_200272165 203.047133 275.179915 130.9143511 0.475740939 -1.071751916 0.476677322 1

chr1_200337922_200340134 199.5271846 241.1576709 157.8966983 0.654744664 -0.610995698 0.686886631 1

chr1_200377862_200380341 704.5410271 767.5017992 641.5802549 0.835933226 -0.25854039 0.761589104 1

chr1_200385264_200386851 82.98170512 55.03598299 110.9274273 2.015543672 1.011169043 0.68436627 1

chr1_200445660_200449517 267.4205935 146.0955185 388.7456685 2.660900707 1.411914677 0.285150613 1

chr1_200707320_200709847 843.6522406 1076.703958 610.600523 0.567101587 -0.818320902 0.304013789 1

chr1_200859716_200861192 182.5481097 256.1674845 108.9287349 0.425224673 -1.233702785 0.439416579 1

chr1_200875000_200879030 373.4827483 347.22702 399.7384766 1.151230905 0.203177228 0.856773103 1

chr1_200940612_200942795 130.9843315 107.0700033 154.8986597 1.446704539 0.532770309 0.7801284 1

chr1_200961058_200962686 92.98497738 70.04579654 115.9241582 1.654976657 0.726810869 0.754514432 1

chr1_201082927_201084328 144.5513717 223.1458947 65.95684864 0.295577244 -1.75839289 0.336403683 1

chr1_201095628_201097165 95.50427157 102.0667321 88.94181104 0.871408433 -0.198579021 0.934367832 1

chr1_201122380_201124800 365.5363764 421.2754335 309.7973194 0.735379504 -0.443439127 0.693974931 1

chr1_201137207_201144761 1130.872708 936.6123651 1325.13305 1.414814815 0.500613231 0.484215983 1

chr1_201164251_201165979 60.47483308 22.0143932 98.93527296 4.494117647 2.168037892 0.48428031 1

chr1_201221839_201225058 241.5585867 331.2165522 151.9006211 0.458614221 -1.124647003 0.415300058 1

chr1_201235718_201238458 141.0431956 207.1354269 74.95096436 0.361845221 -1.466555377 0.42553901 1

chr1_201250627_201256779 627.611645 798.5220805 456.7012095 0.571933101 -0.806081691 0.366534359 1

chr1_201267343_201270808 425.5344272 478.3127249 372.7561294 0.779314683 -0.359722097 0.732582198 1

chr1_201314997_201316571 88.01898547 117.0765456 58.9614253 0.503614323 -0.98960878 0.680006918 1

chr1_201321757_201324537 195.5369941 252.1648675 138.9091206 0.550866272 -0.86022596 0.574468428 1

chr1_201347672_201355178 1598.590924 1738.136408 1459.04544 0.839430917 -0.252516494 0.694041579 1

chr1_201363570_201370715 2134.133877 2339.529604 1928.73815 0.824412799 -0.278561192 0.638561667 1

chr1_201416861_201418922 133.4728866 92.06018973 174.8855835 1.899687411 0.925762046 0.622880237 1

chr1_201427877_201432998 1521.155983 1760.150802 1282.161164 0.728438247 -0.457121422 0.482763851 1

chr1_201435032_201439559 1589.517018 1616.056592 1562.977443 0.967155143 -0.048180762 0.941143588 1

chr1_201448781_201452440 407.5174187 434.2839385 380.750899 0.876732629 -0.189791154 0.860774377 1

chr1_201479653_201482980 456.0622297 551.3604842 360.7639751 0.654315979 -0.611940591 0.548374517 1

chr1_201484416_201488108 219.9941609 211.1380438 228.8502779 1.083889354 0.11621749 0.93846467 1

chr1_201507443_201510268 322.0510826 400.2616945 243.8404707 0.609202614 -0.715005962 0.549715408 1

chr1_201528279_201529238 64.01178609 82.05364737 45.96992481 0.560242308 -0.835877158 0.775915911 1

chr1_201565913_201568549 211.9732304 171.1118744 252.8345864 1.477598135 0.56325395 0.701525607 1

chr1_201616912_201618616 157.0301186 203.13281 110.9274273 0.546083261 -0.87280716 0.612071004 1

chr1_201645062_201645970 46.50229902 50.03271181 42.97188623 0.858875817 -0.219478545 0.95696778 1

chr1_201672501_201673750 64.50688103 75.04906772 53.96469434 0.719058824 -0.475818298 0.872699916 1

chr1_201707553_201712576 1133.891675 968.6333007 1299.150049 1.341219683 0.423545561 0.553496217 1

chr1_201797833_201799542 394.5455389 464.3035656 324.7875122 0.699515438 -0.515572197 0.63548372 1

chr1_201847212_201848102 45.98823744 28.01831861 63.95815626 2.282726424 1.190757968 0.745182659 1

chr1_201857145_201858795 214.0484434 288.18842 139.9084668 0.485475672 -1.042529094 0.476787964 1

chr1_201865219_201866755 77.00328658 82.05364737 71.95292579 0.876901004 -0.189514113 0.946214269 1

chr1_201907175_201914054 954.6745011 1221.798822 687.5501797 0.562735998 -0.82946984 0.275913961 1

chr1_201923284_201925824 704.100216 857.5606805 550.6397515 0.642100045 -0.639129995 0.452787867 1

chr1_201950991_201953592 832.6090728 999.653582 665.5645635 0.665795207 -0.58684961 0.462159765 1

chr1_202098799_202103421 586.0687977 691.4520772 480.6855181 0.695182694 -0.524535926 0.566900509 1

chr1_202166516_202172449 519.4056049 375.2453386 663.5658712 1.76835207 0.822405536 0.394405456 1

chr1_202190334_202192685 194.9889233 178.1164541 211.8613926 1.189454359 0.250299914 0.872254596 1

chr1_202235392_202240708 349.1321872 551.3604842 146.9038901 0.266438917 -1.908123273 0.106104246 1

chr1_202250588_202254618 320.5023576 324.2119725 316.7927427 0.977116114 -0.033398082 0.980531505 1

chr1_202304688_202305782 58.02748134 100.0654236 15.98953906 0.15979085 -2.645743299 0.413239601 1

chr1_202309669_202312225 267.0307962 314.2054302 219.8561621 0.699721077 -0.515148145 0.693849225 1

chr1_202317341_202319128 470.5383609 529.346091 411.7306309 0.777809902 -0.362510494 0.718781485 1

chr1_202347012_202351334 650.9255807 537.3513249 764.4998365 1.422718808 0.50865055 0.562217357 1

chr1_202440935_202441380 30.00654664 40.02616945 19.98692383 0.499346405 -1.00188711 0.842483148 1

chr1_202451646_202454699 163.5324091 213.1393523 113.9254658 0.534511645 -0.903706716 0.591552916 1
[truncated: 3,840,553 more chars]
